# N─H or O─H Bond Activation With a Bicyclic Si_4_ Ring Compound

**DOI:** 10.1002/chem.71315

**Published:** 2026-06-25

**Authors:** Michael Quest, Florian Schreiner, Alexander Hepp, Constantin G. Daniliuc, Kasperi M. Salonen, Janne Körkkö, Heikki M. Tuononen, Felicitas Lips

**Affiliations:** ^1^ Institut für Anorganische und Analytische Chemie Universität Münster Fachbereich Chemie und Pharmazie Münster Germany; ^2^ Organisch‐Chemisches Institut Universität Münster Fachbereich Chemie und Pharmazie Münster Germany; ^3^ Department of Chemistry NanoScience Centre University of Jyväskylä Jyväskylä Finland

**Keywords:** amine, functionalized clusters, ring compounds, silicon, siliconoid cluster

## Abstract

The bicyclic silicon ring compound Si_4_{N(SiMe_3_)Mes}_4_ (Mes = 2,4,6‐trimethylphenyl), a heavier analogue of cyclobutadiene, was reacted with ammonia, water, and selected alcohols. This resulted in E─H (E = N, O) bond activation, yielding saturated Si_4_ rings with ─NH_2_/─H or ─OH/─H functionalities in the case of ammonia or water, respectively. With methanol, a mixture of a saturated Si_4_ ring compound with ─OMe/─H functionalities and an eight‐vertex methoxy‐functionalized siliconoid cluster was obtained; an ethoxy‐functionalized analogue was obtained from the reaction with ethanol. In contrast, the reaction between Si_4_{N(SiMe_3_)Mes}_4_ and phenol gave a simple 1,3‐addition product with only a trace of side products. The observed reactivity was rationalized with the help of mechanistic calculations performed using density functional theory.

## Graphical abstract

The bicyclic Si_4_ ring compound with zwitterionic character reacts with NH_3_, H_2_O, and selected alcohols to give functionalized saturated silicon rings, siliconoid clusters with unsubstituted Si atoms, or simple 1,3‐addition products. The experimentally observed reactivity could be rationalized based on results from mechanistic calculations employing density functional theory.

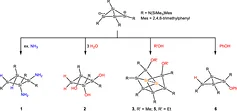

## 1 Introduction

The synthesis and characterization of silicon analogues of cyclobutadiene with a formula Si_4_R_4_ have attracted recurring interest (Figure 1). The first crystallographically characterized example from this class of compounds was the tetrahedral Si_4_(Si*^t^* Bu_3_)_4_ (**A**) by Wiberg et al. in 1993 [1]; Sekiguchi and coworkers reported Si_4_{SiMe(Dis)_2_}_4_ (**B**, Dis = CH(SiMe_3_)_2_) 10 years later [2]. A further increase to the steric bulk of the employed R group was described to afford a planar rhombic charge‐separated Si_4_(EMind)_4_ (**C**, EMind = 1,1,7,7‐tetraethyl‐3,3,5,5‐tetramethyl‐*s*‐hydrindacen‐4‐yl) by Matsuo, Tamao, and coworkers in 2011 [3]. Recently, we reported that the reductive debromination of a tribromosilane {(SiMe_3_)_2_N}SiBr_3_ with 1.5 equivalents of activated magnesium gives Si_4_{N(SiMe_3_)_2_}_4_ (**D**), an amido analogue of **C** [4]. Further studies on this topic showed that structurally new types of heavier analogues of cyclobutadiene with a folded charge‐separated bicyclic silicon ring, Si_4_{N(SiMe_3_)Dipp}_4_ (**E**, Dipp = 2,6‐diisopropylphenyl) [5] and Si_4_{N(SiMe_3_)Mes}_4_ (**F**, Mes = 2,4,6‐trimethylphenyl) [6], can be obtained by adjusting the steric and electronic properties of the employed tribromosilane.

**FIGURE 1:**
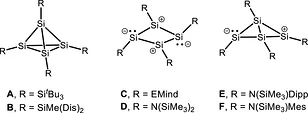
Crystallographically characterized examples of silicon analogues of cyclobutadiene with a general formula Si_4_R_4_. Dis = bis(trimethylsilyl)methyl, EMind = 1,1,7,7‐tetraethyl‐3,3,5,5‐tetramethyl‐*s*‐hydrindacen‐4‐yl, Dipp = 2,6‐diisopropylphenyl, Mes = 2,4,6‐trimethylphenyl.

In contrast to the instability of **C** in solution that prevents further exploration of its reaction chemistry [3], the three compounds **D**–**F** display rich reactivity with a wide array of reagents (Scheme 1). For example, the bis(trimethylsilyl)amido‐substituted compound **D** undergoes cycloadditions with alkenes and alkynes [4]. In the case of folded bicyclic rings, the sterically very bulky diisopropylphenyl substituents in **E** allow reactivity with chalcogens and chalcogen transfer agents, yielding cage compounds Si_x_Ch_y_ (Ch = S, Se, Te) [7], while reductive abstraction of amido ligands is observed in reactions with *N*‐heterocyclic carbenes [8] or potassium graphite [9]. Lowering the steric demand of the amido substituent to mesityl proved necessary to probe the reactivity related to the zwitterionic nature of the two tricoordinate silicon atoms, allowing the characterization of Lewis acid and/or base adducts of **F** [6]. In addition, the abstraction of one or two amido ligands from **F** with potassium graphite gave access to dianionic cubane‐type Si_8_ species through head‐to‐tail dimerization of four‐membered silylene intermediates [10].

**SCHEME 1:**
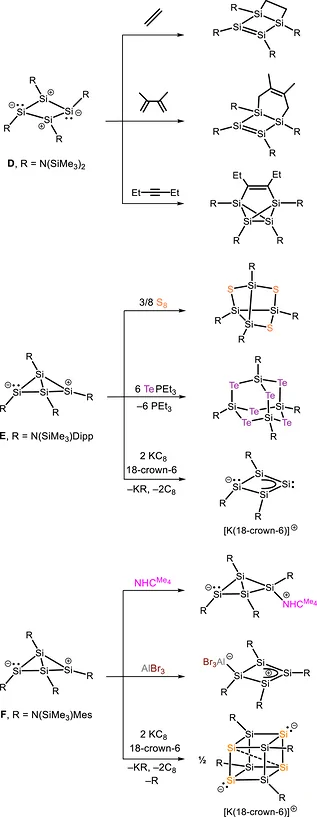
Selected examples of reactivity observed for cyclobutadiene analogues **D**–**F** with various reagents [4, 5, 6, 7, 8, 9, 10]. Dipp = 2,6‐diisopropylphenyl, Mes = 2,4,6‐trimethylphenyl, NHC^Me4^ = 1,3,4,5‐tetramethylimidazol‐2‐ylidene.

In this contribution, we report the results of our investigations of the reactivity of **F** with ammonia, water, and selected alcohols, leading to E─H (E = N, O) bond activation. We demonstrate that the products from these reactions depend on the type of reagent used, the reaction conditions, and the employed stoichiometry, with experimental results supported by density functional theory (DFT) calculations. To the best of our knowledge, prior investigations of similar reactivity of unsaturated Si_4_ ring compounds are limited to water and methanol, with no examples involving ammonia or other types of alcohols. Specifically, the unsaturated bicyclic Si_4_R_6_/Si_4_R_4_R’_2_ ring compounds **G** and **H** undergo hydrolysis involving the weak transannular Si─Si bond (Scheme 2a,b) [11, 12], whereas Si_4_ ring compounds with Si─OH and Si─H functionalities have been observed as side products in the reductive coupling of chlorosilanes [13]. Considering reactivity with methanol, the iodo functionalities in the cyclodisilene **I** can be replaced by methoxy groups with concomitant release of HI and retention of the Si═Si double bond [14], while the homocyclic silylene **J** reacts with MeOH to give a fully saturated Si_4_ ring compound with Si─OMe and Si─H functionalities (Scheme 2c,d) [9].

**SCHEME 2:**
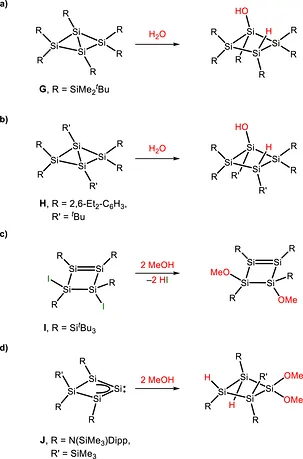
Reactions of various unsaturated Si_4_ ring compounds with water (a and b) and methanol (c and d) [9, 11, 12, 14]. Dipp = 2,6‐diisopropylphenyl.

## 2 Results and Discussion

We commenced our investigations by reacting Si_4_{N(SiMe_3_)Mes}_4_ with an excess of ammonia in THF at −78 °C and 1 atm. This resulted in the clean formation of a yellow‐colored product (89% isolated yield). Results from multinuclear NMR spectroscopic analyses were consistent with the formation of a four‐membered silicon ring compound **1** (Scheme 3). For example, the ^1^H NMR spectrum shows a broad signal at 0.26 ppm and a singlet at 5.79 ppm that can be assigned to Si─NH_2_ and Si─H functionalities, respectively. Similarly, signals in the ^29^Si{^1^H} IG NMR spectrum at −16.2 and −32.3 ppm are consistent with Si─NH_2_ and Si─H units, respectively, while those at 6.3 and 7.1 ppm can be assigned to the SiMe_3_ groups of N(SiMe_3_)Mes substituents. The presence of Si─H functionalities in **1** was further confirmed with a ^29^Si{^1^H} DEPT‐90 NMR experiment that revealed a singlet at −32.3 ppm. FT‐IR spectroscopy showed a band at 2111 cm^−1^ that is consistent with isolated Si─H stretching vibrations, while the band at around 3300–3500 cm^−1^ is characteristic for symmetric and asymmetric N─H stretching vibrations.

**SCHEME 3:**
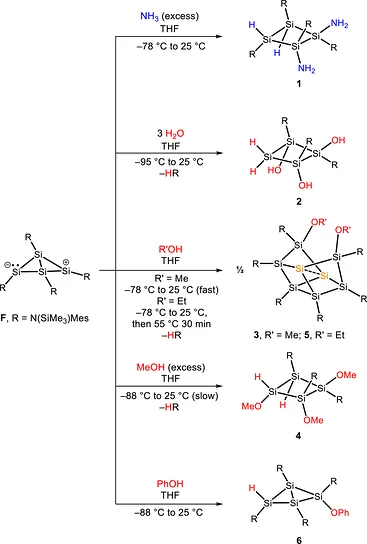
Synthesis of **1**–**6**. Mes = 2,4,6‐trimethylphenyl.

The molecular structure of **1** was determined by single‐crystal X‐ray diffraction. The crystal structure of **1** (Figure 2) shows two independent molecules in the asymmetric unit with very similar metric parameters. The Si_4_ ring in **1** deviates only slightly from planarity, and the arrangement of Si–NH_2_ and Si–H groups is antiperiplanar. The Si─Si (2.406(1)–2.421(1) Å), Si─NH_2_ (1.720(2)–1.725(2) Å), Si─N(SiMe_3_)Mes (1.750(2)–1.770(2) Å), and Si─H (1.41(3)–1.54(4) Å) bond lengths are in the expected range for amido‐substituted silicon ring compounds [4, 5, 6, 7, 8, 9, 10] and indicative of single bonds between the respective elements [15].

**FIGURE 2:**
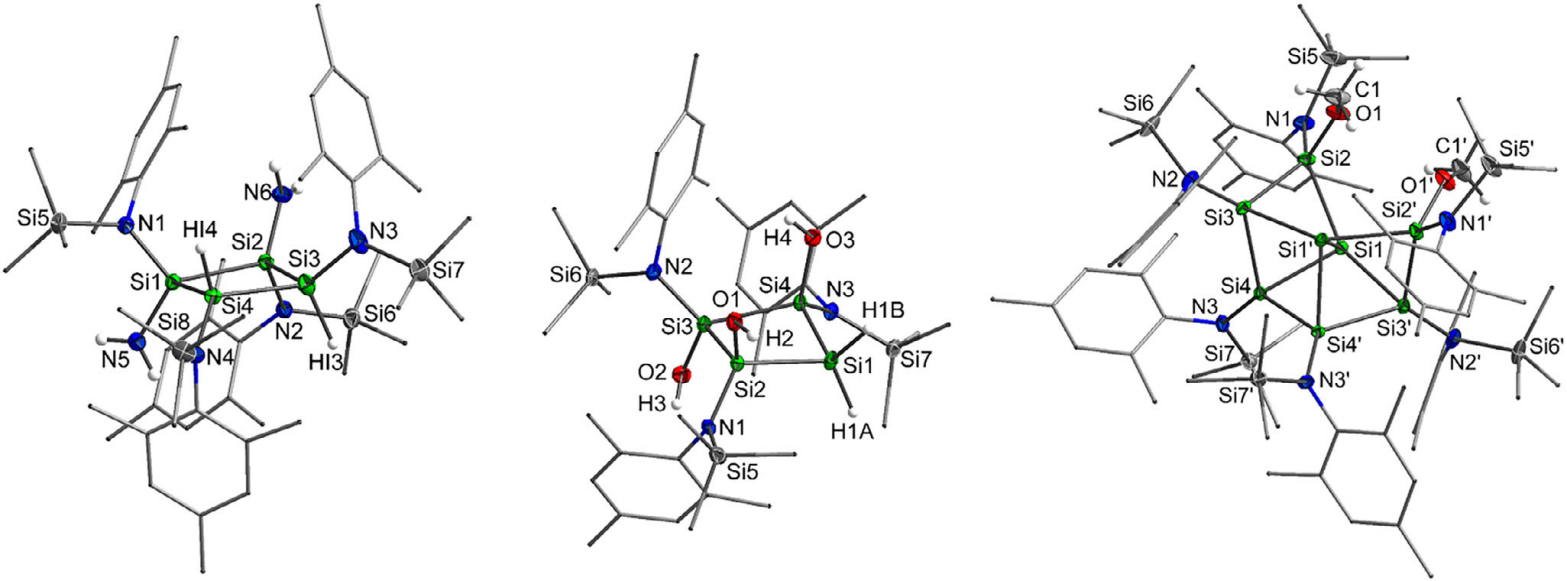
Molecular structures of **1** (left), **2** (middle), and **3** (right) with thermal ellipsoids drawn at 50% probability level and C─H hydrogen atoms omitted for clarity.

The mechanism for the formation of **1** was investigated with DFT using the PBE1PBE hybrid functional [16, 17, 18, 19]. with D3BJ empirical dispersion correction [20, 21] and def2‐SVP basis sets [22]. Various possibilities were considered, but by far the lowest energy pathway was found to involve two formal 1,3‐additions that generate the observed Si─NH_2_ and Si─H functionalities in an antiperiplanar arrangement (Scheme 4). The first addition proceeds through coordination of ammonia to the Lewis acidic Si^+^ center in **F**, followed by a transfer of one of its protons to a second molecule of NH_3_ that, in turn, passes it to the Lewis basic Si^−^ center. The formation of [NH_4_]^+^ is mediated by the two flanking amido ligands and represents the highest point on the calculated pathway with a barrier height of only 58 kJ mol^−1^ (the Gibbs energy difference between the transition state and the minimum preceding it). The addition of a second molecule of NH_3_ proceeds in an analogous manner and is associated with very small activation barriers throughout. Hence, the formation of **1** is expected to be facile even at low temperatures, as observed experimentally. The high basicity of NH_3_ together with the vast stoichiometric excess employed most likely prevents any side reactions involving proton transfer to the amido ligand (*cf*. formation of **2**–**5**). It should be noted that the calculated energetics did not change upon inclusion of a continuum solvent model or by increasing the size of the employed basis set (Supporting Information).

**SCHEME 4:**
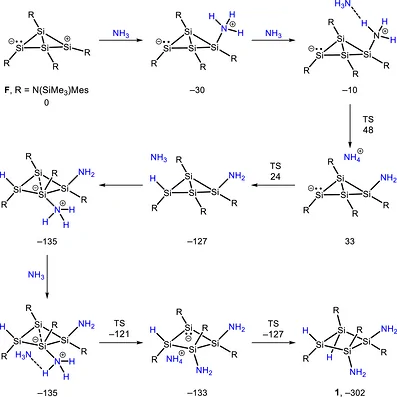
Calculated mechanism for the formation of **1**. Gibbs energies (kJ mol^−1^) are reported relative to **F** + 3 NH_3_ and refer to gas phase calculations at room temperature. Mes = 2,4,6‐trimethylphenyl.

Next, **F** was reacted with one equivalent of water at −95 °C. However, it was soon realized that unreacted **F** remained in solution, and three equivalents of H_2_O are required to effect complete conversion. Employing a strict 1:3 stoichiometric ratio of reagents resulted in almost quantitative conversion of **F** to a single product, **2**, according to NMR spectroscopic and subsequent X‐ray crystallographic analyses (Scheme 3). For example, the ^1^H NMR spectrum of **2** shows two singlets at 3.81 and 3.71 ppm that can be assigned to the SiH_2_ unit. Both signals show ^29^Si satellites with ^1^ *J*_H,Si_ coupling constants of 186.6 and 187.2 Hz, and ^2^ *J*_H,Si_ coupling constants of 10.2 and 19.3 Hz, respectively. In the ^29^Si{^1^H} IG NMR spectrum of **2**, the Si atom of the SiH_2_ unit resonates at −93.3 ppm, while the three hydroxy‐substituted Si atoms resonate at 3.7 and −1.6 ppm.

Single crystal X‐ray diffraction study performed on isolated pale yellow crystals of **2** (13% yield) confirmed that the reaction of **F** with H_2_O results in the addition of three ─OH and two ─H functionalities to the Si_4_ ring with simultaneous removal of one of its amido ligands as an amine (Figure 2); the presence of HN(SiMe_3_)Mes in solution could be detected by NMR spectroscopy. The Si_4_ ring in **2** is slightly folded with torsion angles *ca*. ±14 °. The Si─Si (2.3545(9)–2.4243(9) Å), Si─OH (1.6412(18)–1.6775(19) Å), Si─N(SiMe_3_)Mes (1.728(2)–1.738(2) Å), and Si─H (1.41(4)–1.44(3) Å) bond lengths are in the expected range for amido‐substituted silicon ring compounds [4, 5, 6, 7, 8, 9, 10] and indicative of single bonds between the respective elements [15].

The ─OH and ─H functionalities in **2** were also characterized by FT‐IR spectroscopy, which showed Si–H stretches at 2158 and 2122 cm^−1^ and O─H vibrations at 3588, 3572, and 3556 cm^−1^. The deuterium analogue of **2**, dubbed **2’**, was synthesized using three equivalents of D_2_O in place of H_2_O. The FT‐IR spectrum of **2’** revealed complete loss of signals assignable to Si─H and O─H functionalities along with the emergence of new ones at around 1550 and 2650 cm^−1^ (*ν*_SiH_/*ν*_SiD_ ≈ 1.36), corresponding to Si─D and O─D stretching vibrations, respectively. This demonstrates that all ─OH/─OD and ─H/─D functionalities in **2/2’** arise from the added molecules of H_2_O/D_2_O. Furthermore, the presence of a SiD_2_ unit in **2’** was observed in the ^29^Si{^1^H} IG NMR spectrum that shows a quintet at −94.4 ppm with a ^1^ *J*_D,Si_ coupling constant of 29.2 Hz.

A computational analysis of the mechanism for the formation of **2** showed that a formal 1,3‐addition of a single equivalent of water to **F** requires the combined action of three molecules of H_2_O and takes place in an almost barrierless manner through two transition states connected by an oxonium intermediate (Scheme 5). It should be noted that the sequential buildup of the intermediate **F** + 3 H_2_O can take place starting either from the Lewis basic Si^−^ or the Lewis acidic Si^+^ center, and only the former is shown in Scheme 5 due to its slightly lower‐energy intermediates.

**SCHEME 5:**
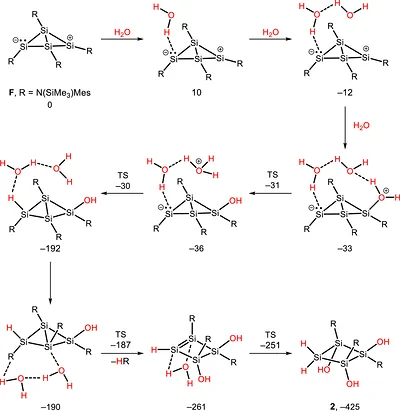
Calculated mechanism for the formation of **2**. Gibbs energies (kJ mol^−1^) are reported relative to **F** + 3 H_2_O and refer to gas phase calculations at room temperature. Mes = 2,4,6‐trimethylphenyl.

In the next step, the initial 1,3‐addition product is converted into a cyclodisilene through hydrogen bond‐assisted oxidative addition involving two H_2_O molecules. One of the amido ligands is simultaneously lost as an amine. The addition of H_2_O to the Si═Si double bond in the cyclodisilene constitutes the third and final step on the pathway to **2**. In principle, the Si═Si bond can be attacked from either side of the Si_4_ ring, and two different relative orientations of the Si═Si and O─H units are possible. However, the pathway leading to **2** was found to have a clear energetic preference over possible alternatives.

Interestingly, the reaction of **F** with MeOH at −78 °C resulted in the formation of a mixture of two products, denoted **3** and **4**, whose relative proportions could be affected by adjusting the employed stoichiometry and/or the reaction conditions. When two equivalents of methanol were used, and the reaction mixture was rapidly warmed to room temperature, a methoxy‐functionalized eight‐vertex siliconoid cluster **3** with two unsubstituted silicon atoms and six amine substituents could be obtained almost quantitatively based on NMR spectroscopy in solution (Scheme 3). Compound **3** could be isolated in the form of yellow crystals in moderate (54%) yield and its structure determined by single crystal X‐ray diffraction (Figure 2).

The structure of **3** features a distance of 2.4586(7) Å between the two unsubstituted Si atoms, with all other Si─Si bonds in the range of typical single bonds (2.3472(5)–2.3950(5) Å) [15]. In the ^29^Si{^1^H}IG NMR spectrum of **3**, the unsubstituted Si atoms appear at −136.2 ppm, while the other Si atoms resonate between 16.3 and −58.5 ppm. These data are comparable to those reported for the eleven‐vertex siliconoid cluster **K** (Figure 3) that has been obtained in very low yield via thermolysis or photolysis of a six‐vertex siliconoid species [23]. Another point for comparison is offered by the borderline silicon clusters **L** or **M** (Figure 3) with eight or six unsubstituted silicon atoms, respectively [24, 25]. These clusters feature Si─Si bond lengths of 2.361(1)–2.384(1) Å for **L** and 2.212(2)–2.368(2) Å for **M**, and display their most shielded ^29^Si NMR signals at −71.8 and −84.6 ppm, respectively.

**FIGURE 3:**
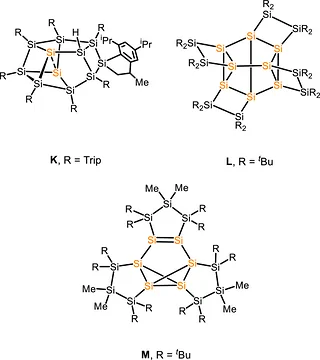
Silicon clusters **K**, **L**, and **M** with unsubstituted Si atoms. Trip = 2,4,6‐triisopropylphenyl.

When the reaction between **F** and MeOH was carried out using an excess of methanol (1:4 stoichiometry) and the reaction mixture was allowed to slowly warm to room temperature (6–8 h), the formation of compound **4** was preferred over **3** based on NMR spectroscopy. Even though **4** could not be crystallized and characterized by single‐crystal X‐ray crystallography, its atomic composition could be assigned based on ESI mass spectrometry, and a tentative structure for it was deduced based on data from 2D NMR experiments (Supporting Information). However, the ROE cross signals did not allow us to distinguish between **4** and its isomer **4’** that differ with respect to the relative orientation of their ─OMe/─H and ─R/─H functionalities (Figure 4).

**FIGURE 4:**
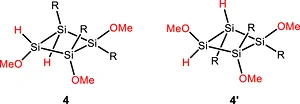
Structural suggestions for **4** based on 2D NMR data.

A plausible pathway for the formation of **3** was formulated based on DFT calculations (Scheme 6). It involves the facile conversion of **F** to a methoxy‐functionalized silylene intermediate through oxidative addition and combined action of two methanol molecules, leading to the loss of one of the amido ligands as an amine. Next, the methoxy‐functionalized silylene intermediate forms a weakly bound head‐to‐head dimer (*via* a double donor‐acceptor interaction) in a barrierless manner and, ultimately, the siliconoid cluster **3** through a single transition state. This postulation is consistent with our previous findings related to the formation of anionic cubane‐type clusters that involve the dimerization of two anionic Si_4_ rings, though in a head‐to‐tail fashion (Scheme 1) [10].

**SCHEME 6:**
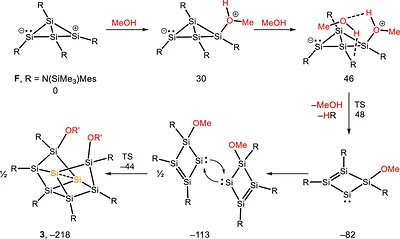
Calculated mechanism for the formation of **3**. Gibbs energies (kJ mol^−1^) are reported relative to **F** + 2 MeOH and refer to gas phase calculations at room temperature. Mes = 2,4,6‐trimethylphenyl.

Given the apparent similarities in the structures of **2** and **4**, a mechanism for the formation of the latter was conceived based on Scheme 5 and investigated computationally. The results show that, similar to the reaction with H_2_O, a formal 1,3‐addition of a single equivalent of methanol to **F** requires the combined action of three molecules of MeOH and involves a very low‐barrier transition state (Scheme 7). It should, however, be noted that the addition of the first equivalent of MeOH involves only a single transition state, whereas the H_2_O reaction mechanism was found to involve two transition states linked by an oxonium intermediate (Scheme 5).

**SCHEME 7:**
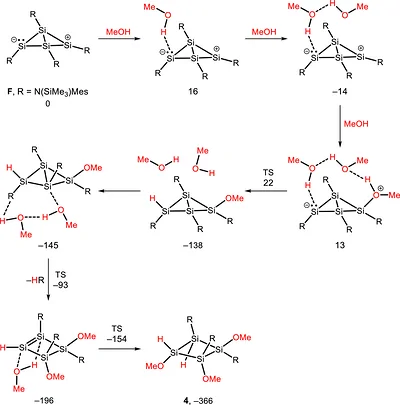
Calculated mechanism for the formation of **4**. Gibbs energies (kJ mol^−1^) are reported relative to **F** + 3 MeOH and refer to gas phase calculations at room temperature. Mes = 2,4,6‐trimethylphenyl.

The next step on the pathway converts the initial 1,3‐addition product into a cyclodisilene through oxidative addition involving two hydrogen‐bonded MeOH molecules with simultaneous loss of one of the amido ligands. Subsequent addition of methanol to the Si═Si double bond in the cyclodisilene intermediate can, in principle, yield **4** or any of its three isomers, depending on the relative orientation of the attacking MeOH and the Si═Si double bond (*cf*. mechanism for the formation of **2**). Interestingly, the two isomers with a SiH_2_ unit were found to be preferred in terms of both calculated reaction energy and barrier height, though **4** or **4’** is the experimentally observed product. At this point, it is assumed that the outcome of the final reaction step is affected by temperature (slow heating of the reaction mixture), which allows the MeOH molecule present at the end of the previous reaction step to directly attack the Si═Si double bond, thereby leading to the formation of a single isomer (Scheme 7). Thus, we tentatively assign **4** as the product from the reaction between **F** and MeOH with 1:4 stoichiometry.

The mechanisms outlined in Schemes 6 and 7 are consistent with experimental evidence, that is, both low **F**:methanol ratio and low temperature favor the formation of the saturated Si_4_ ring species **4**, whereas the opposite conditions prefer the siliconoid cluster **3**. It should also be noted that the silylene intermediate formed on the pathway leading to **3** (Scheme 6) could, in principle, undergo oxidative addition with MeOH to yield a cyclodisilene and, ultimately, **4**. However, our recent results indicate that the transition states involved in such a process are much higher in energy than those leading to **3** [9]. Consequently, it is unlikely that compounds **3** and **4** are formed from a common intermediate, giving further, albeit indirect, support for the mechanisms in Schemes 6 and 7.

The reaction of **F** with ethanol was found to proceed in a slightly different manner compared to the same reaction with methanol. First, when using two equivalents of EtOH, the ethoxy‐substituted siliconoid cluster **5** could be isolated in moderate (30%) yield (Scheme 3), but the reaction needed to be driven forward by heating at 55 °C for 30 min. Second, much smaller amounts of a side product, presumably the ethoxy analogue of **4**, were formed in the reaction. The cluster **5** showed similar structural and NMR spectroscopic properties to cluster **4** (Supporting Information). For example, a single‐crystal X‐ray structural study showed that the bond length between unsubstituted Si atoms in **5** is 2.4420(7) Å with all other Si─Si bond lengths between 2.3658(5) and 2.4102(5) Å. The ^29^Si{^1^H}IG NMR spectrum of **5** shows signals at −140.3 ppm for the unsubstituted Si atoms, while those for ligand‐substituted atoms appear between 17.9 and −60.1 ppm.

The mechanism for the formation of **5** was assumed to be analogous to that involving **3** and, therefore, not examined computationally. The increased size and greater fluxionality of ethyl compared to methyl are expected to affect the dimerization of the silylene intermediate into **5** (Scheme 6), which rationalizes the heating required to drive the reaction forward. In a similar manner, the use of EtOH over MeOH makes the 1,3‐addition pathway disfavored due to increased sterics (Scheme 7), which is consistent with the fact that the reaction between **F** and EtOH gave a single main product under all conditions.

As the last experiment, compound **F** was reacted with two equivalents of PhOH. Detailed 2D NMR spectroscopic analyses (Supporting Information) showed that the main (90%) product from this reaction is the formal 1,3‐addition species **6** with ─OPh and ─H functionalities in *anti*‐arrangement (Scheme 3). The side product obtained in low (10%) yield was found to be structurally similar to **6** but with one of its SiMe_3_ groups replaced by a hydrogen atom (Supporting Information). Furthermore, a siliconoid cluster analogous to **3** and **5** but with ─OPh and ─H functionalities was also formed in trace quantities as yellow‐colored single crystals suitable for X‐ray crystallographic structure determination (Supporting Information). All attempts to selectively alter the product distribution by changing the reaction conditions or using a different stoichiometry were met with failure.

A computational analysis of the formation of **6** indicated that a formal 1,3‐addition of a single equivalent of PhOH to **F** does not take place in the same manner as observed in reactions with H_2_O (Scheme 5) or MeOH (Scheme 7). In addition, such a reaction would lead to the formation of a *syn*‐isomer rather than the experimentally observed *anti*‐arrangement of ─OPh and ─H functionalities in **6**. Furthermore, given the fact that **6** is formed even when using one equivalent of PhOH, the mechanism is likely to involve ionic intermediates. Such a mode of action is understandable, given the higher acidity of phenol in low‐polar aprotic solvents, such as THF [26], compared to water, methanol, or ethanol. Consequently, a computational analysis of a proton transfer from PhOH to the Lewis basic Si^−^ center in **F** gave a barrier height of only *ca*. 50 kJ mol^−1^. Next, the nucleophilic phenoxide ion could attack the Lewis acidic Si^+^ center in [H–**F**]^+^, yielding **6**. Calculations indicated that this step has no energy barrier associated with it and should be very facile.

## 3 Conclusion

The results reported in this contribution demonstrate the ability of the bicyclic silicon ring compound Si_4_{N(SiMe_3_)Mes}_4_ (Mes = 2,4,6‐trimethylphenyl), a heavier analogue of cyclobutadiene, to effect E─H (E = N, O) bond activation with ammonia, water, and selected alcohols. In the case of NH_3_ and H_2_O, the saturated Si_4_ ring compounds **1** and **2** with multiple amino or hydroxy functionalities, respectively, were obtained. The amino‐functionalized Si_4_ ring species **1** is unprecedented, which is also the case for the three hydroxy groups present in the ring structure of **2**. The reactions of Si_4_{N(SiMe_3_)Mes}_4_ with two equivalents of methanol and ethanol gave the alkoxy‐functionalized clusters **3** and **5**. These compounds feature unsubstituted Si atoms and are unique examples of siliconoid clusters obtained through a synthetic route other than thermolysis or reductive oligomerization. When phenol was used as the reactant, the formal 1,3‐addition product **6** with ─OPh and ─H functionalities was formed almost exclusively. The experimental observations were rationalized based on density functional theory calculations. The results indicated that compound **1** forms via two formal 1,3‐additions of NH_3_ to Si_4_{N(SiMe_3_)Mes}_4_, whereas compounds **2** and **4** involve a reaction sequence combining formal 1,3‐addition, oxidative addition with loss of amido ligand, and addition to a Si═Si double bond. The mechanism for the formation of siliconoid clusters **3** and **5** was found to involve oxidative addition with concomitant ligand loss and subsequent head‐to‐head dimerization of the resulting silylene intermediate. The mechanism for the formation of **6** presumably takes place via ionic intermediates, leading to formal 1,3‐addition with an *anti*‐arrangement of the attached ─OPh and ─H functionalities.

## Conflicts of Interest

The authors have nothing to report.

## Supporting information




**Supporting File 1**: Further experimental and computational details along with complete characterization data for **1**–**6** are provided in the Supporting Information [27]. The authors have cited additional references within the Supporting Information [28, 29, 30, 31, 32, 33, 34, 35, 36, 37, 38, 39, 40].

## Data Availability

The data that support the findings of this study are available from the corresponding author upon reasonable request.
