# Supplementary material for: N─H or O─H Bond Activation With a Bicyclic Si4 Ring Compound
Source: Chemistry. 2026 Jun 25;32(37):e71315. doi: 10.1002/chem.71315 (PMC13628535; doi:10.1002/chem.71315)
Supplement: Supplementary file 1 [file CHEM-32-e71315-s001.pdf]

Table of Contents

1. General Experimental Procedures (S2)

2. Details for the Synthesis and Characterization of 1 to 7 (S3–S61)

   2.1 $H_2(NH_2)_2Si_4\{N(SiMe_3)Mes\}_4$ (1) (S3–S12)

   2.2 $H_2(OH)_3Si_4\{N(SiMe_3)Mes\}_3$ (2) (S12–S23)

   2.3 $D_2(OD)_3Si_4\{N(SiMe_3)Mes\}_3$ (2') (S24–S34)

   2.4 Comparison of IR data for 2 and 2' (S35–S37)

   2.5 $(OMe)_2Si_8\{N(SiMe_3)Mes\}_6$ (3) (S38–S47)

   2.6 $H_2(OMe)_3Si_4\{N(SiMe_3)Mes\}_3$ (4) (S48–S58)

   2.7 $(OEt)_2Si_8\{N(SiMe_3)Mes\}_6$ (5) (S59–S68)

   2.8 $(H(OPh)Si_4\{N(SiMe_3)Mes\}_4$ (6) and $(H)(OPh)Si_8\{N(SiMe_3)Mes\}_6$ (7) (S69–S79)

3. Details of Single Crystal X-ray Diffraction Analyses (S62–S91)

   3.1 Refinement details (S80–S91)

4. Computational Details (S91)

   4.1 Further discussion of the formation of 6 (S91)

5. Cartesian Coordinates and Gibbs Energies (S92–S259)

6. References (S260)

1. General Experimental Procedures

All reactions were performed with modified Schlenk techniques with additional manipulations done using an MBraun Glovebox. All solvents were dried over sodium benzophenone or CaH_2, distilled. and stored over 3 Å molecular sieves prior to use. Silicon tetrabromide was purchased from Aldrich and was used without further purification. The starting material $Si_4\{N(SiMe_3)Mes\}_4$ (**F**, Mes = 2,4,6-trimethylphenyl) was prepared according to literature procedures.[6] NH_3 (99.98 %, Westfalen AG) was passed over a column with molecular sieves and Sicapent® prior to use. Methanol and ethanol were dried with CaH_2, distilled three times, and stored over 3 Å molecular sieves prior to use. H_2O and D_2O were degassed in vacuum and stored under argon prior to use. Phenol was dried in vacuum for 16 h prior to use.

All NMR data were obtained on Bruker Avance I and III spectrometers and were referenced to deuterated solvents (C_6D_6, THF-d_8, or PhMe-d_8) according to an IUPAC recommendation. Additionally, the ^{1}H and ^{13}C NMR spectra were referenced internally to residual solvent resonances at 300 K. ^{1}H chemical shifts are given relative to tetramethylsilane (TMS; δ = 0 ppm). ^{13}C, ^{15}N and ^{29}Si NMR chemical shifts are referenced according to the unified chemical shift scale with TMS as the primary reference. All ^{13}C NMR spectra were recorded proton decoupled. Further explanation of the experiments: ^{13}C DEPT-135 = ^{13}C NMR measurement with Distortionless Enhancement of Polarization Transfer using a 135° decoupler pulse; ^{29}Si DEPT-19.5 = ^{29}Si NMR measurement with Distortionless Enhancement Polarization Transfer method, pulse angle 19.5°, coupling to 9 protons as polarization source with coupling $^{2}J_{Si\text{–}H}$ = 7 Hz; ^{29}Si DEPT-90 = ^{29}Si NMR measurement with Distortionless Enhancement Polarization Transfer method, pulse angle 90°; ^{29}Si{^{1}H} IG = Inverse-Gated proton decoupled ^{29}Si NMR measurement; ^{29}Si{/} = ^{29}Si proton coupled NMR measurement.

IR spectra were recorded as KBr-pellets on a Shimadzu IR PRESTIGE 21 or a Bruker Invenio R spectrometer. UV-Visible spectra were recorded as dilute solutions in quartz cuvettes with 1 cm diameter using an Agilent Cary 60 spectrometer. Mass spectrometry was performed with a Varian MAT 212 Micromass Quattro LC-Z, a Thermo Fisher LTQ Orbitrap XL, or a Bruker Impact II QTOF device. CHN elemental analyses werre performed with a Vario EL III CHN instrument. Melting points were measured in glass capillaries sealed under argon gas by using a Stuart Melting Point Apparatus SMP3 or a Büchi Melting Point 560 instrument and are uncorrected.

2. Details for the Synthesis and Characterization of 1 to 7

2.1 $H_2(NH_2)_2Si_4\{N(SiMe_3)Mes\}_4$ (1)

Tetrasilatetraamide $Si_4\{N(SiMe_3)Mes\}_4$ **F** (500.3 mg, 0.533 mmol, 1 eq.) was dissolved in THF (20 mL) and cooled to −78 °C. Ammonia (1 atm, gaseous) was bubbled into the solution using a Pasteur pipette for 10 s. The solution turned yellow immediately. Afterwards, the ammonia stream was removed, and the reaction mixture was allowed to reach room temperature. All volatiles were removed *in vacuo* and the yellow residue was dissolved in benzene. From a concentrated benzene solution yellow single crystals suited for X-ray diffraction studies were obtained at room temperature.

Yield: 0.4617 g (0.475 mmol, 89 %).

Melting Point: 162 °C.

^{1}H NMR (C_6D_6, 400.10 MHz, 300 K): δ (ppm) = 6.84 (s, 2H, *meta*–Ar–C*H*), 6.78 (s, 2H, *meta*–Ar–C*H*), 6.73 (s, 2H, *meta*–Ar–C*H*), 6.63 (s, 2H, *meta*–Ar–C*H*), 5.79 (s, 2H, Si–*H*), 2.60 (s, 6H, *ortho*–Ar–CH_3), 2.41 (s, 6H, *ortho*–Ar–CH_3), 2.22 (s, 6H, *para*–Ar–CH_3), 2.13 (s, 6H, *ortho*–Ar–CH_3), 2.10 (s, 6H, *para*–Ar–CH_3), 1.91 (s, 6H, *ortho*–Ar–CH_3), 0.31 (s, 18H, Si–(CH_3)$_3$), 0.26 (br, 4H, NH_2), 0.22 (s, 18H, Si– (CH_3)$_3$).

^{13}C{^{1}H} NMR (C_6D_6, 100.62 MHz, 300 K): δ (ppm) = 144.3 (s, *ipso*–Ar–*C*), 143.8 (s, *ipso*–Ar–*C*), 138.0 (s, *ortho*–Ar–*C*), 137.3 (s, *ortho*–Ar–*C*), 137.1 (s, *ortho*–Ar–*C*), 136.6 (s, *ortho*–Ar–*C*), 133.4 (s, *para*–Ar–*C*), 133.0 (s, *para*–Ar–*C*), 130.0 (s, *meta*–Ar–*C*H), 129.4 (s, *meta*–Ar–*C*H), 129.2 (s, *meta*–Ar–*C*H), 129.0 (s, *meta*–Ar–*C*H), 22.1 (s, *ortho*–Ar–*C*H_3), 22.1 (s, *ortho*–Ar–*C*H_3), 22.0 (s, *ortho*–Ar–*C*H_3), 22.0 (s, *ortho*–Ar–*C*H_3), 21.9 (s, *para*–Ar–*C*H_3), 20.8 (s, *para*–Ar–*C*H_3), 3.9 (s, Si–(*C*H_3)$_3$), 2.7 (s, Si–(*C*H_3)$_3$).

^{13}C{^{1}H} Dept-135 NMR (C_6D_6, 100.62 MHz, 300 K): δ (ppm) = 130.0 (s, *meta*–Ar–*C*H), 129.4 (s, *meta*–Ar–*C*H), 129.2 (s, *meta*–Ar–*C*H), 129.0 (s, *meta*–Ar–*C*H), 22.1 (s, *ortho*–Ar–*C*H_3), 22.1 (s, *ortho*–Ar–*C*H_3), 22.0 (s, *ortho*–Ar–*C*H_3), 22.0 (s, *ortho*–Ar–*C*H_3), 21.9 (s, *para*–Ar–*C*H_3), 20.8 (s, *para*–Ar–*C*H_3), 3.9 (s, Si–(*C*H_3)$_3$), 2.7 (s, Si–(*C*H_3)$_3$).

^{1}H,^{15}N HMBC NMR (C_6D_6, 40.55 MHz, 300 K): δ (ppm) = 58.5 (s, Ar–*N*–Si–(CH_3)$_3$), 28.1 (s, Ar–*N*–Si–(CH_3)$_3$), 22.8 (d, Si–*N*H_2).

^{29}Si{^{1}H} IG NMR (C_6D_6, 79.49 MHz, 300 K): δ (ppm) = 7.1 (s, *Si*–(CH_3)$_3$), 6.3 (s, *Si*–(CH_3)$_3$), −16.2 (s, *Si*–NH_2), −32.3 (s, *Si*–H).

^{29}Si{^{1}H} DEPT-19.5 NMR (C_6D_6, 79.49 MHz, 300 K): δ (ppm) = 7.1 (s, *Si*–(CH_3)$_3$), 6.3 (s, *Si*–(CH_3)$_3$), −16.2 (s, *Si*–NH_2).

^{29}Si{^{1}H} DEPT-90 NMR (C_6D_6, 79.48 MHz, 300 K): δ (ppm) = −32.3 (s, *Si*–H).

MS (ESI, positive mode, dissolved in THF): m/z = $[M+H]^+$ 971.51281 (971.51409 calc., Δ = 1.32 ppm), $[M+Na]^+$ 993.49457 (993.49603 calc., Δ = 1.47 ppm)

IR (KBr-pellet): $\tilde{\nu}$ (cm^{-1}) = 3456 w, 3431 w, 3353 w, 2949 m, 2915 m, 2860 w, 2111 w, 1732 vw, 1653 vw, 1607 vw, 1559 vw, 1539 vw, 1534 w, 1524 vw, 1475 m, 1445 w, 1373 vw, 1343 vw, 1303 w, 1249 s, 1207 s, 1177 vw, 1153 m, 1126 w, 1112 vw, 1083 vw, 1067 vw, 1057 vw,

1035 vw, 1022 vw, 1008 vw, 911 vs, 901 vs, 872 vs, 831 s, 756 m, 729 m, 698 vw, 681 w, 653 w, 619 s, 604 s, 588 m, 567 s, 548 m, 526 s, 515 s, 502 s.

UV-vis (THF, *c* = 0.51 mmol/mL): λ_{max} = 435 nm (shoulder), ε = $0.1 \cdot 10^3$ $L \cdot mol^{-1} \cdot cm^{-1}$.

CHN for $C_{48}H_{87}N_6Si_8$: calc.: C 59.3 %, H 9.0 %, N 8.7 %; found: C 59.0 %, H 9.1 %, N 8.8 %.

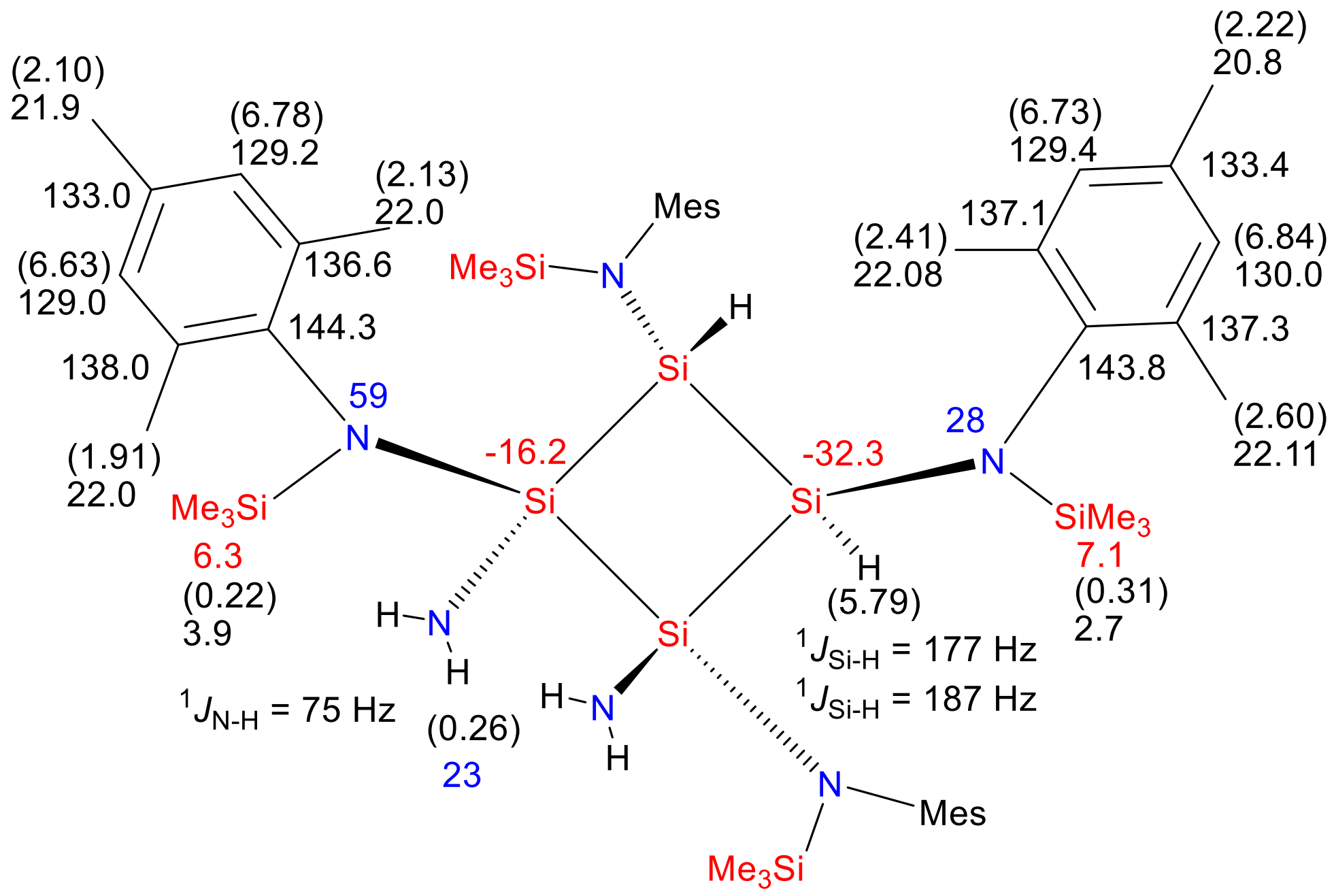

Figure S1. Assignment of chemical shifts to **1** (in C_6D_6 at 300 K). Color coding: ^{1}H black in brackets, ^{13}C black, ^{15}N blue, and ^{29}Si red.

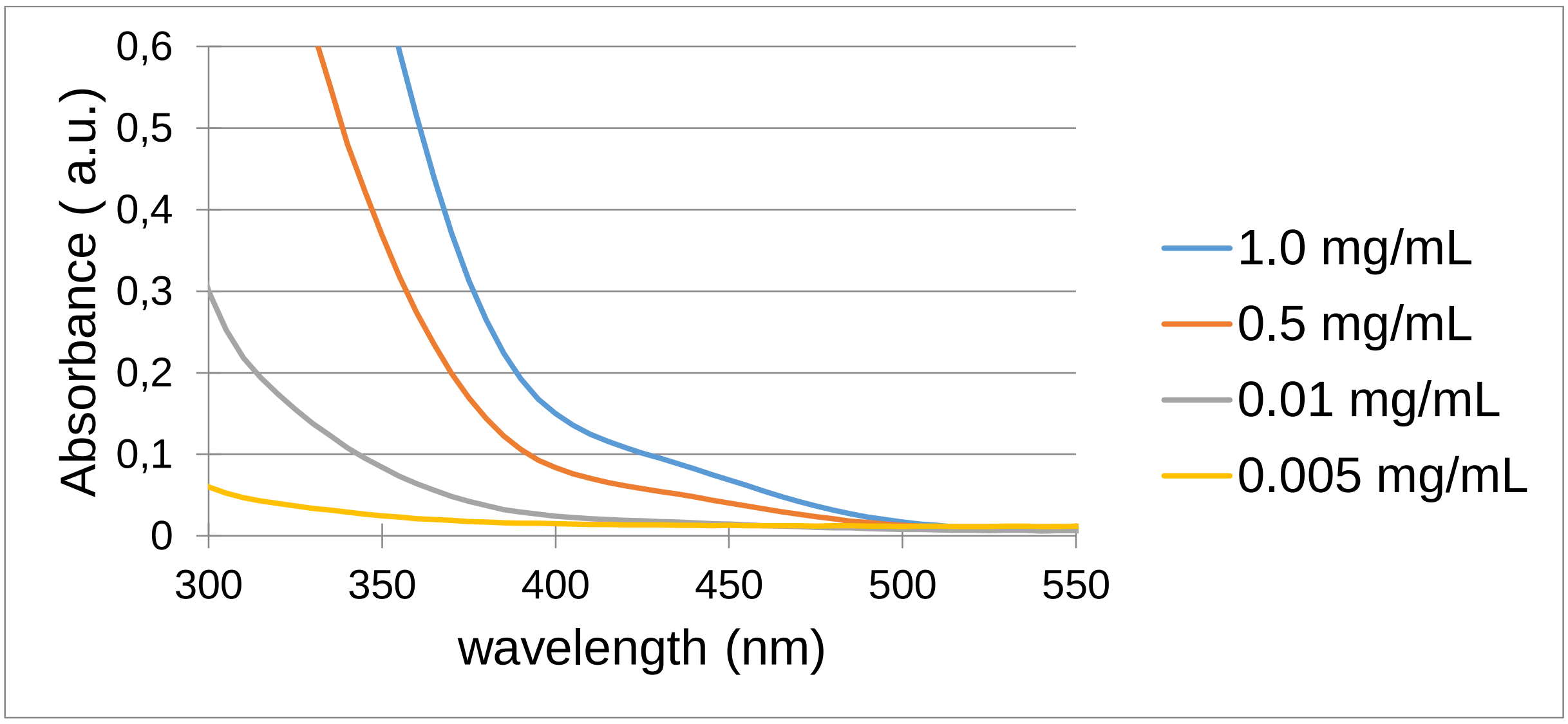

Figure S2. UV-vis spectra of **1** in THF.

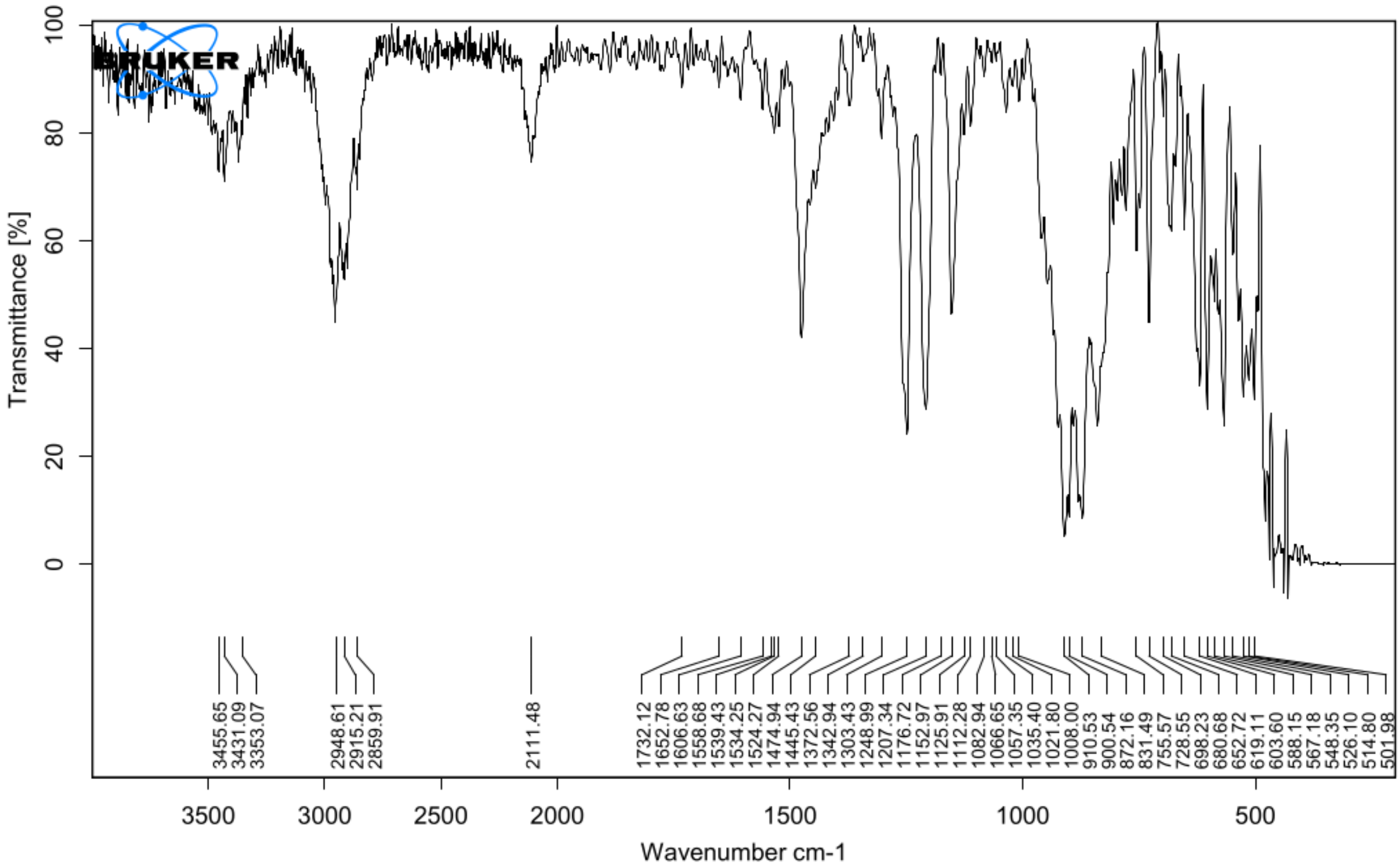

Figure S3. FT-IR spectrum of **1** (KBr-pellet).

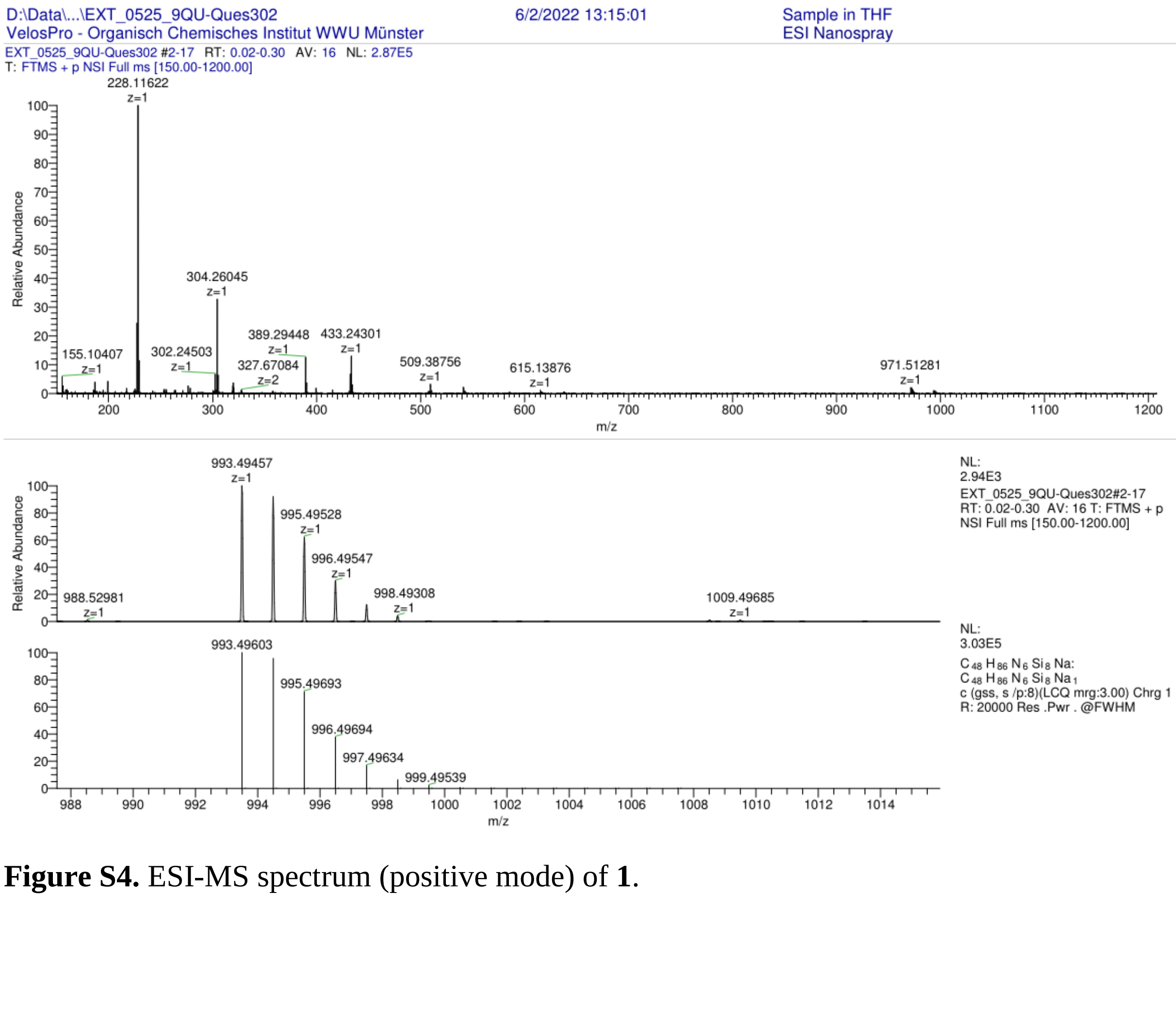

Figure S4. ESI-MS spectrum (positive mode) of **1**.

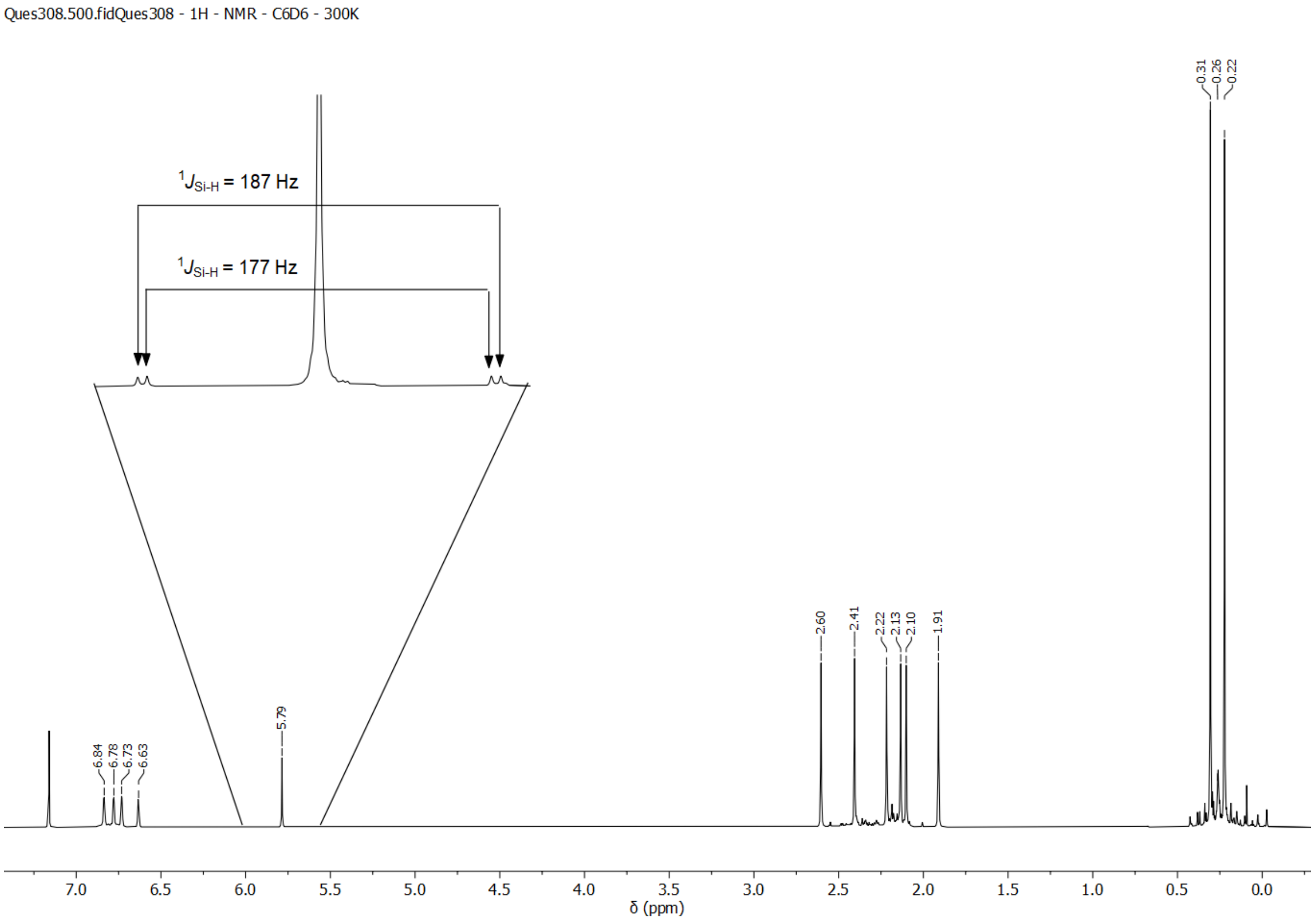

Figure S5. ^{1}H NMR spectrum of **1** in C_6D_6 at 300 K and 500 MHz.

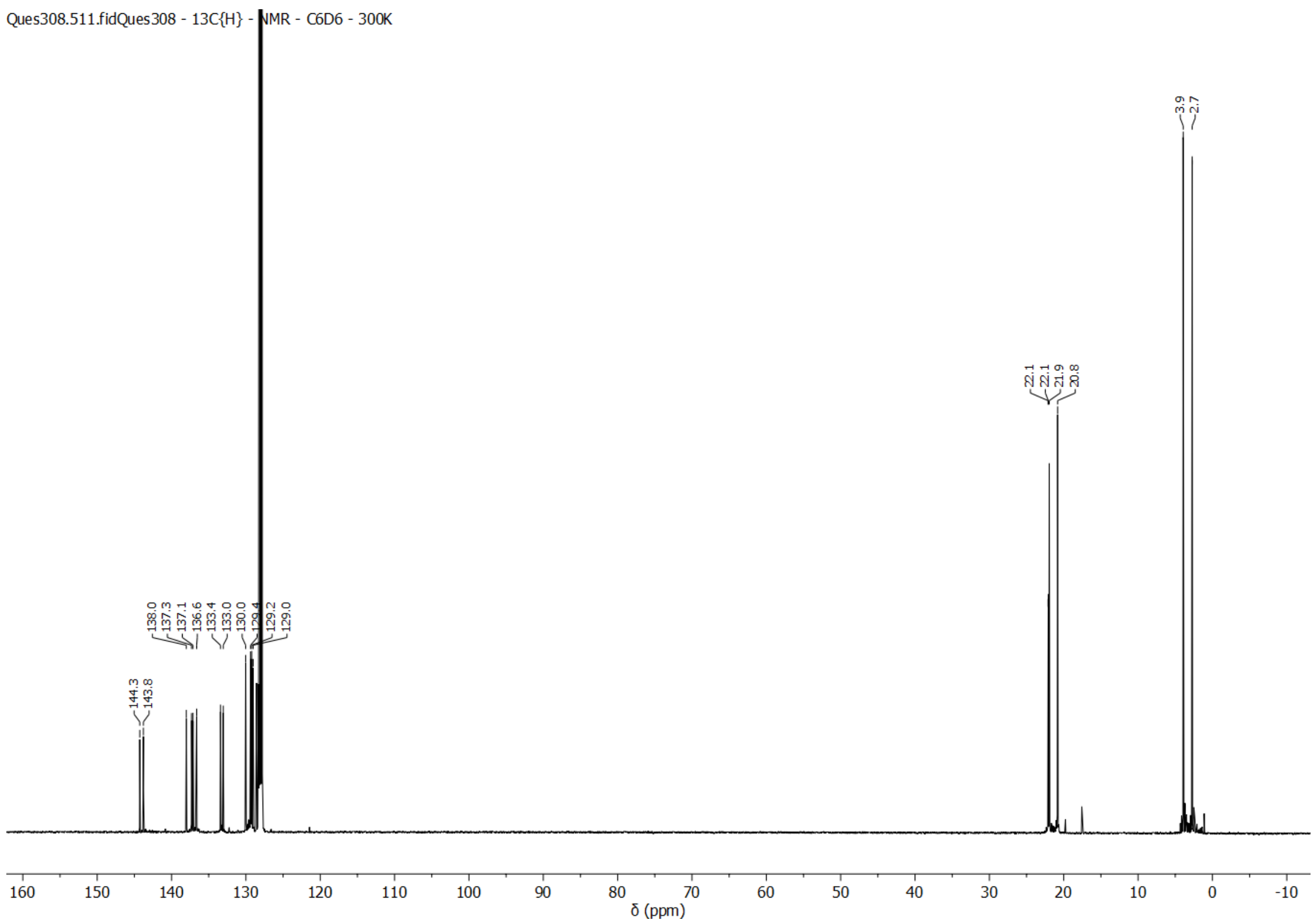

Figure S6. ^{13}C{^{1}H} NMR spectrum of **1** in C_6D_6 at 300 K and 126 MHz.

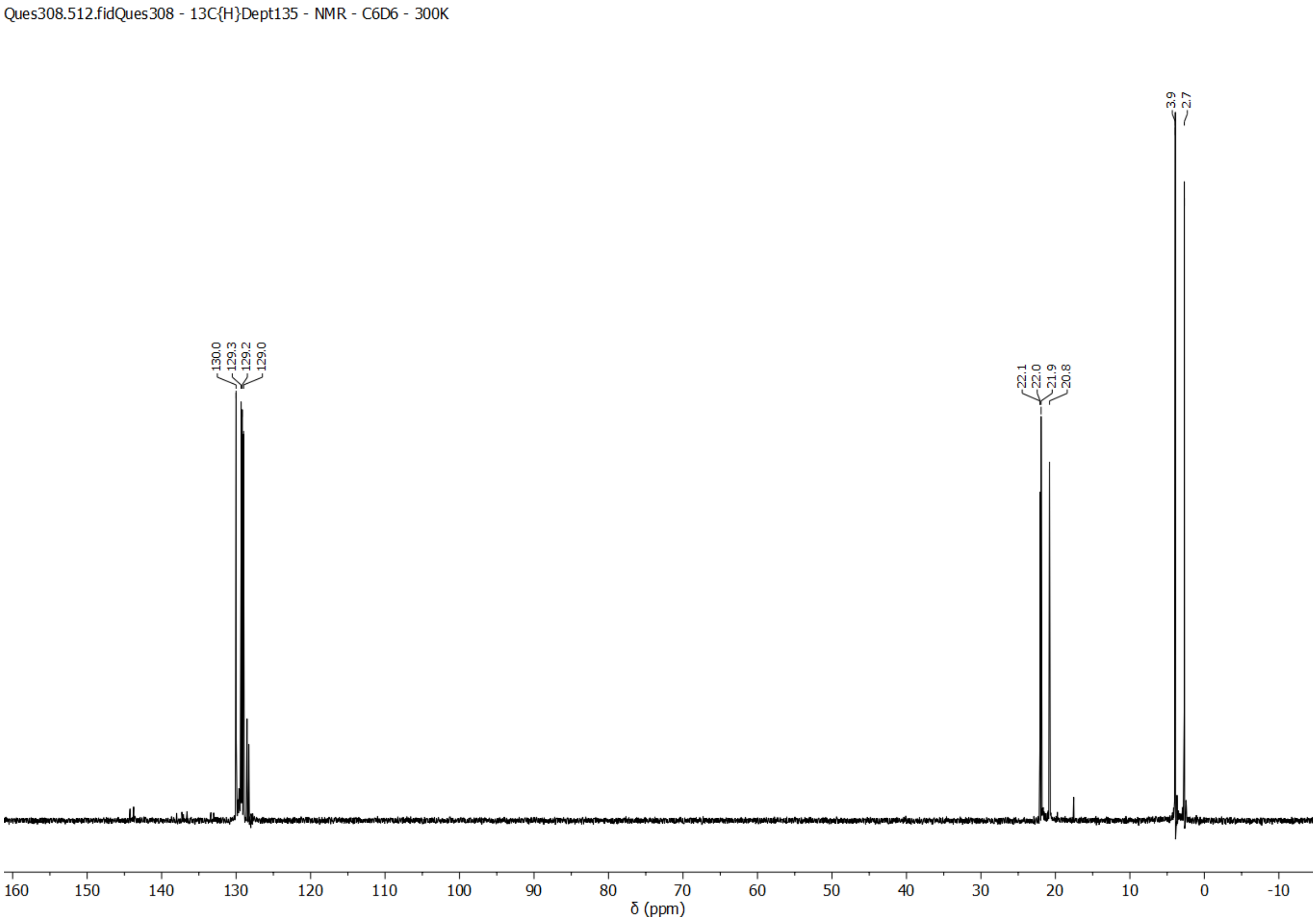

Figure S7. $^{13}C\{^{1}H\}$ Dept-135 NMR spectrum of **1** in C_6D_6 at 300 K and 126 MHz.

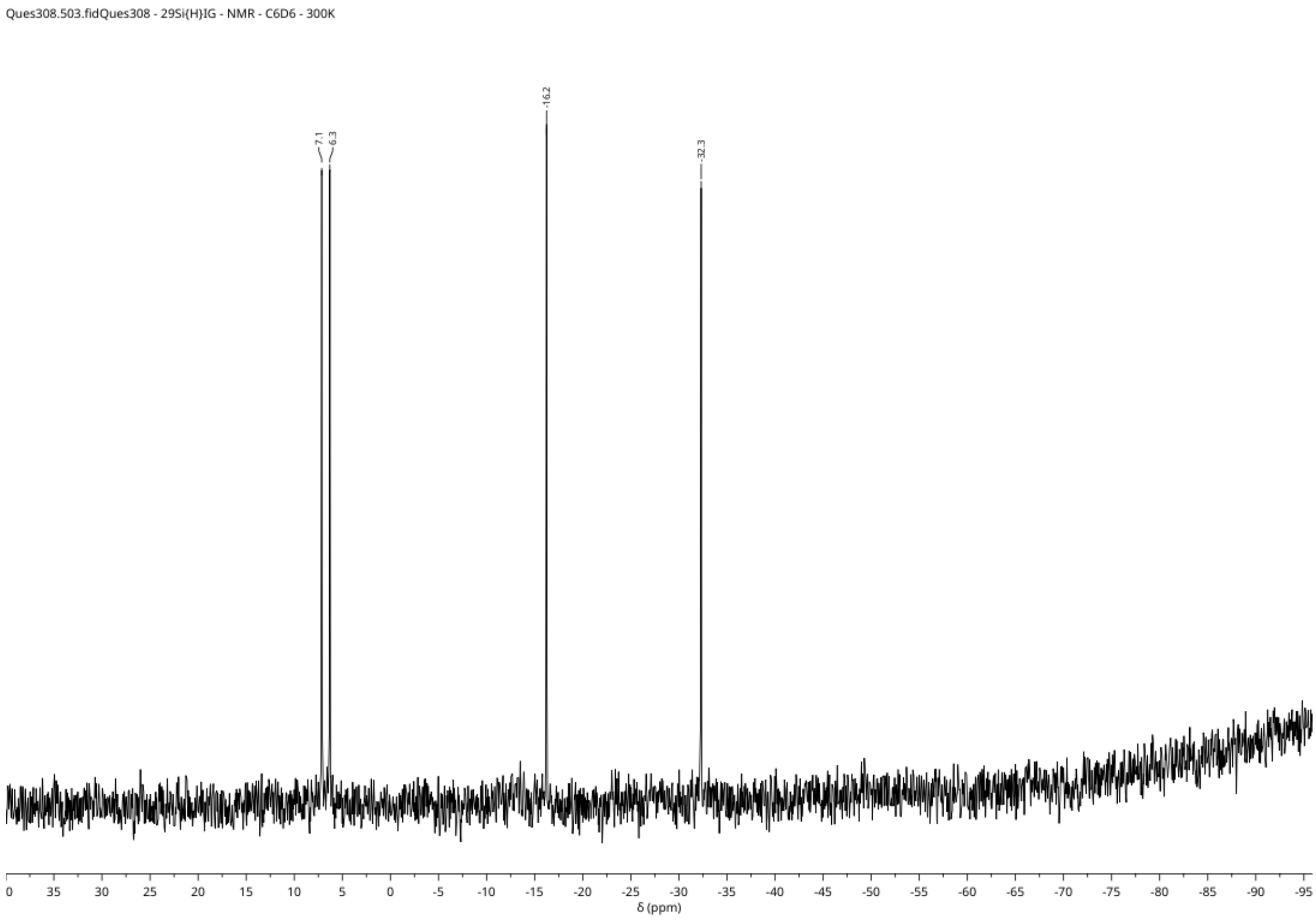

Figure S8. $^{29}Si\{^{1}H\}$ IG NMR spectrum of **1** in C_6D_6 at 300 K and 99 MHz.

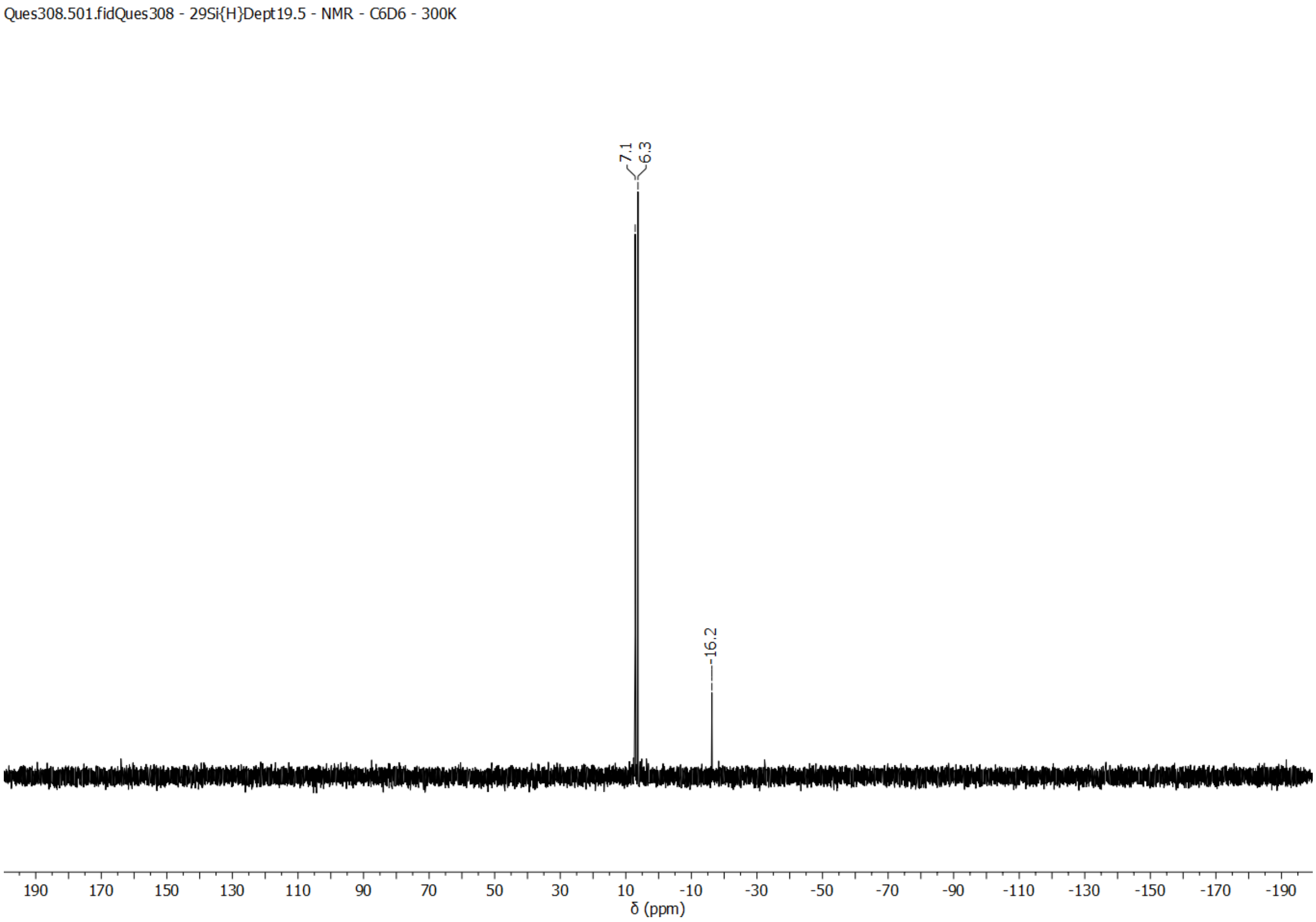

Figure S9. $^{29}Si\{^1H\}$ DEPT-19.5 NMR spectrum of **1** in C_6D_6 at 300 K and 99 MHz.

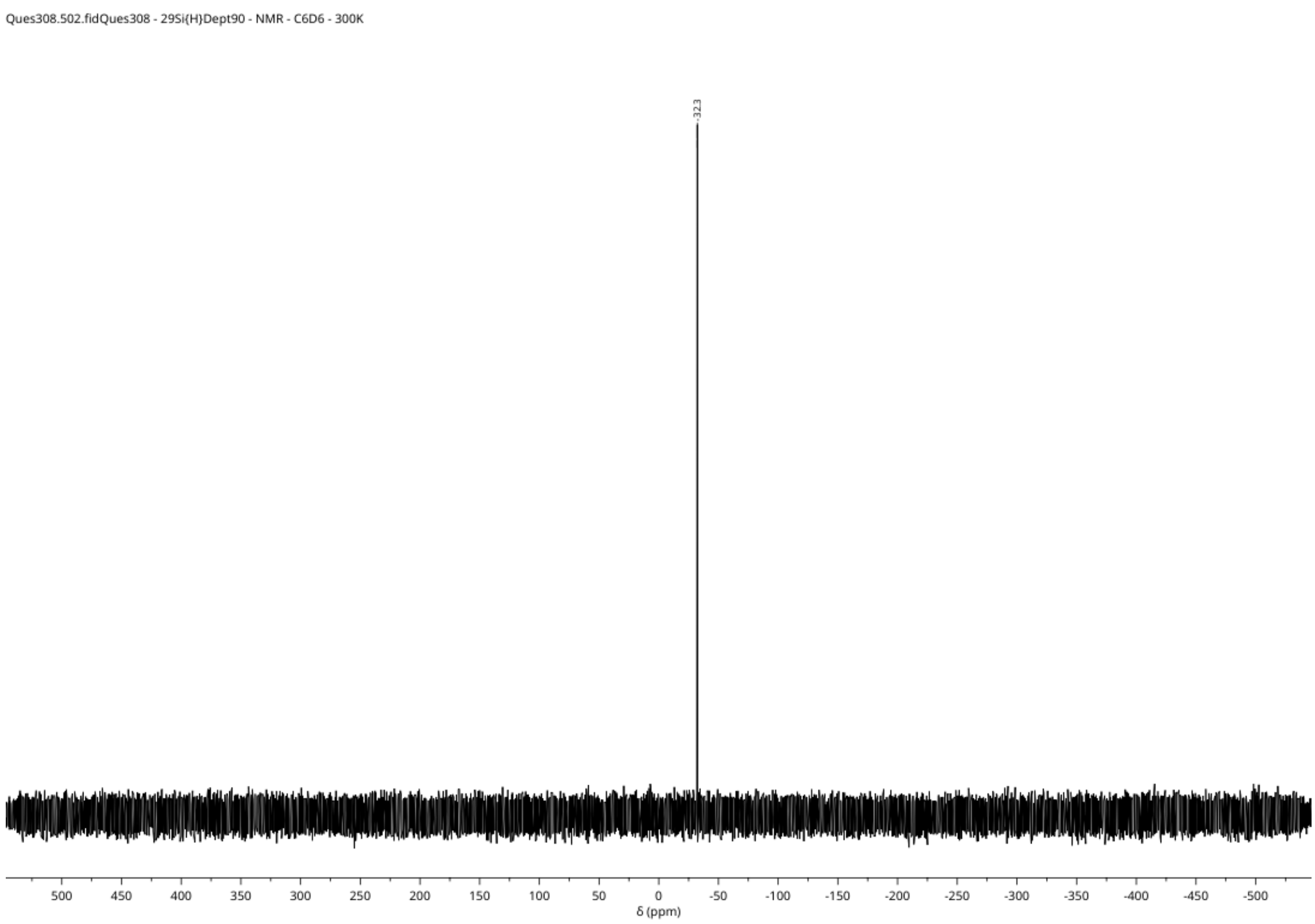

Figure S10. $^{29}Si\{^1H\}$ DEPT-90 NMR spectrum of **1** in C_6D_6 at 300 K and 99 MHz.

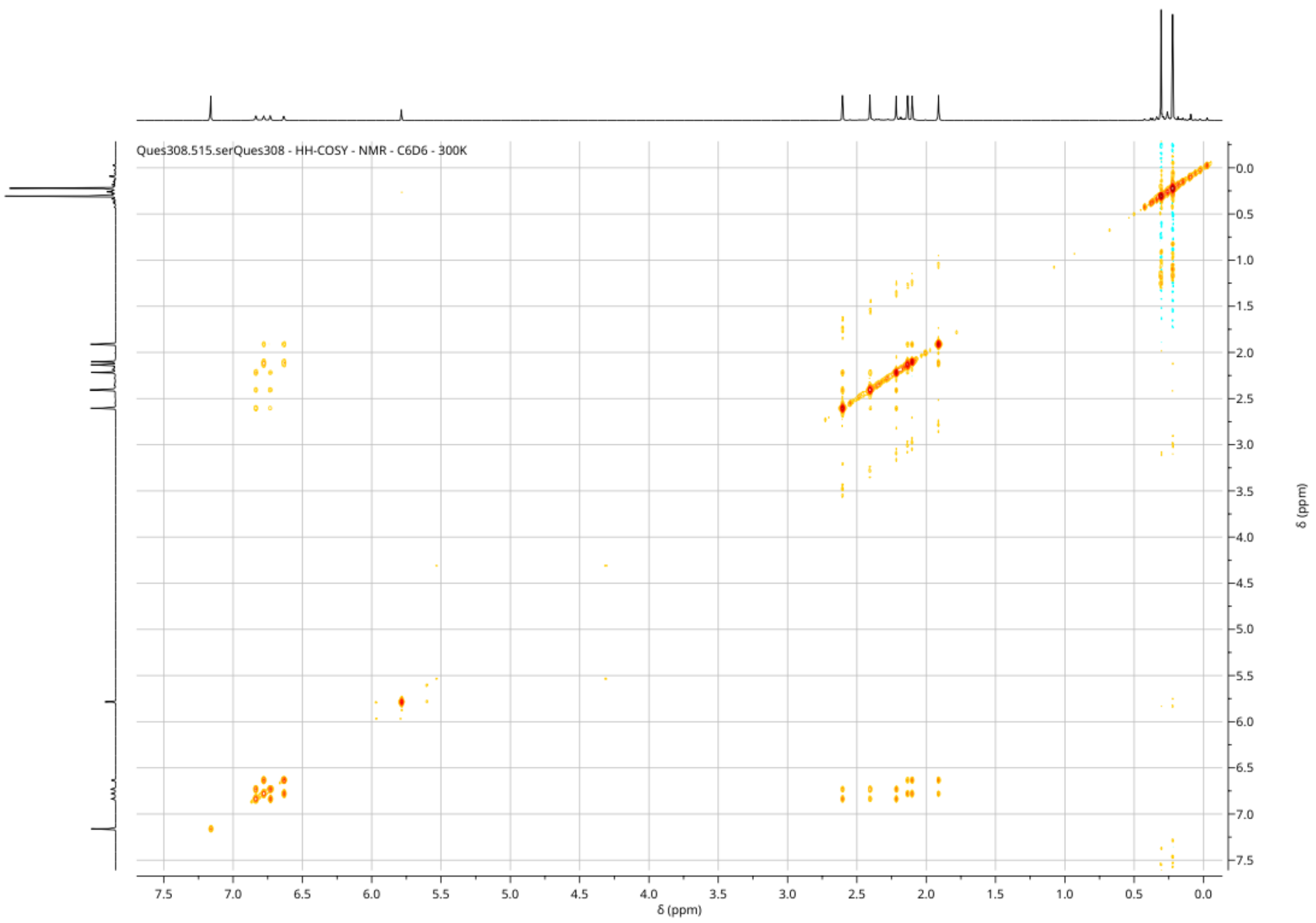

Figure S11. ^{1}H,^{1}H COSY NMR spectrum of **1** in C_6D_6 at 300 K and 500 MHz.

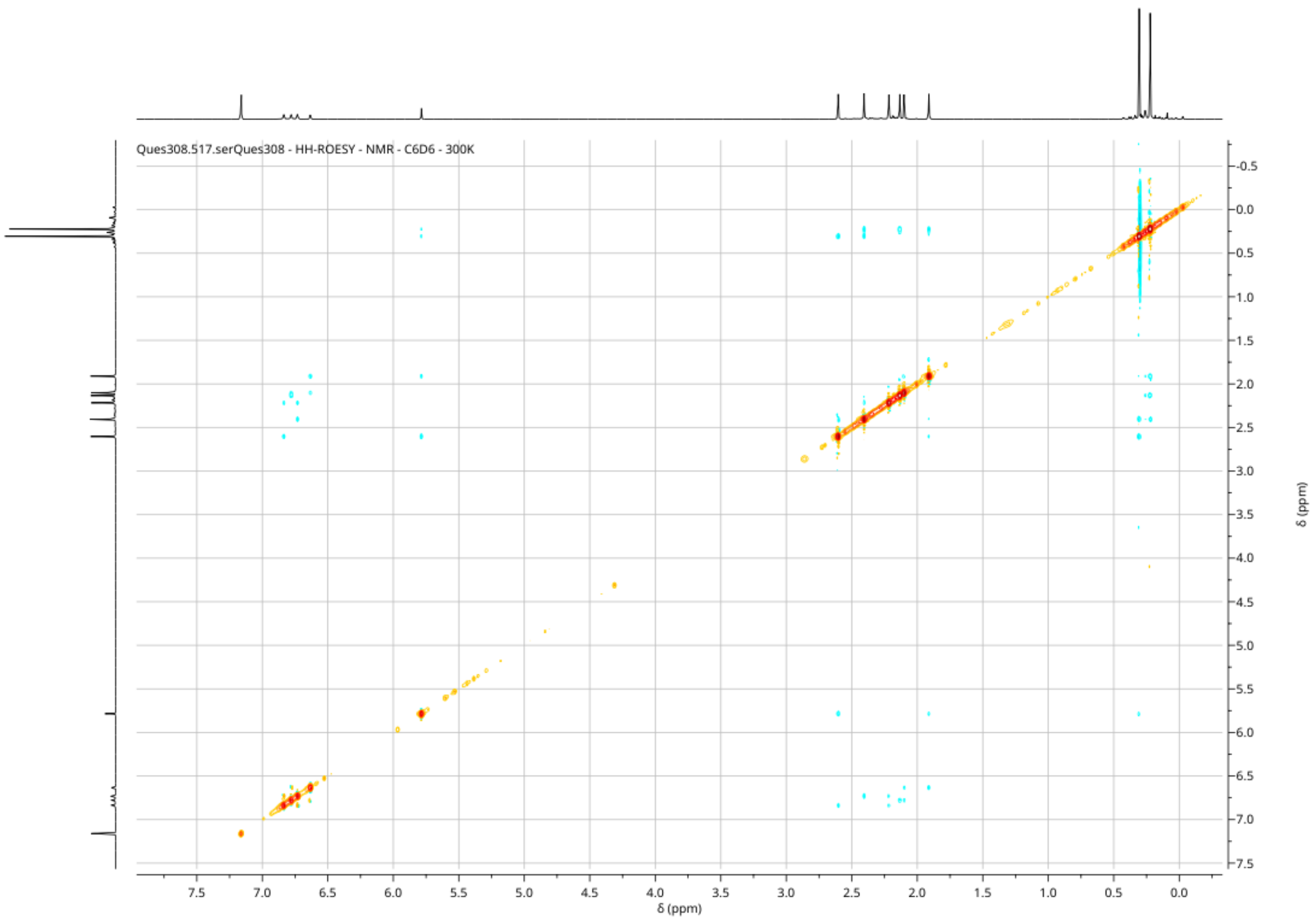

Figure S12. ^{1}H,^{1}H ROESY NMR spectrum of **1** in C_6D_6 at 300 K and 500 MHz.

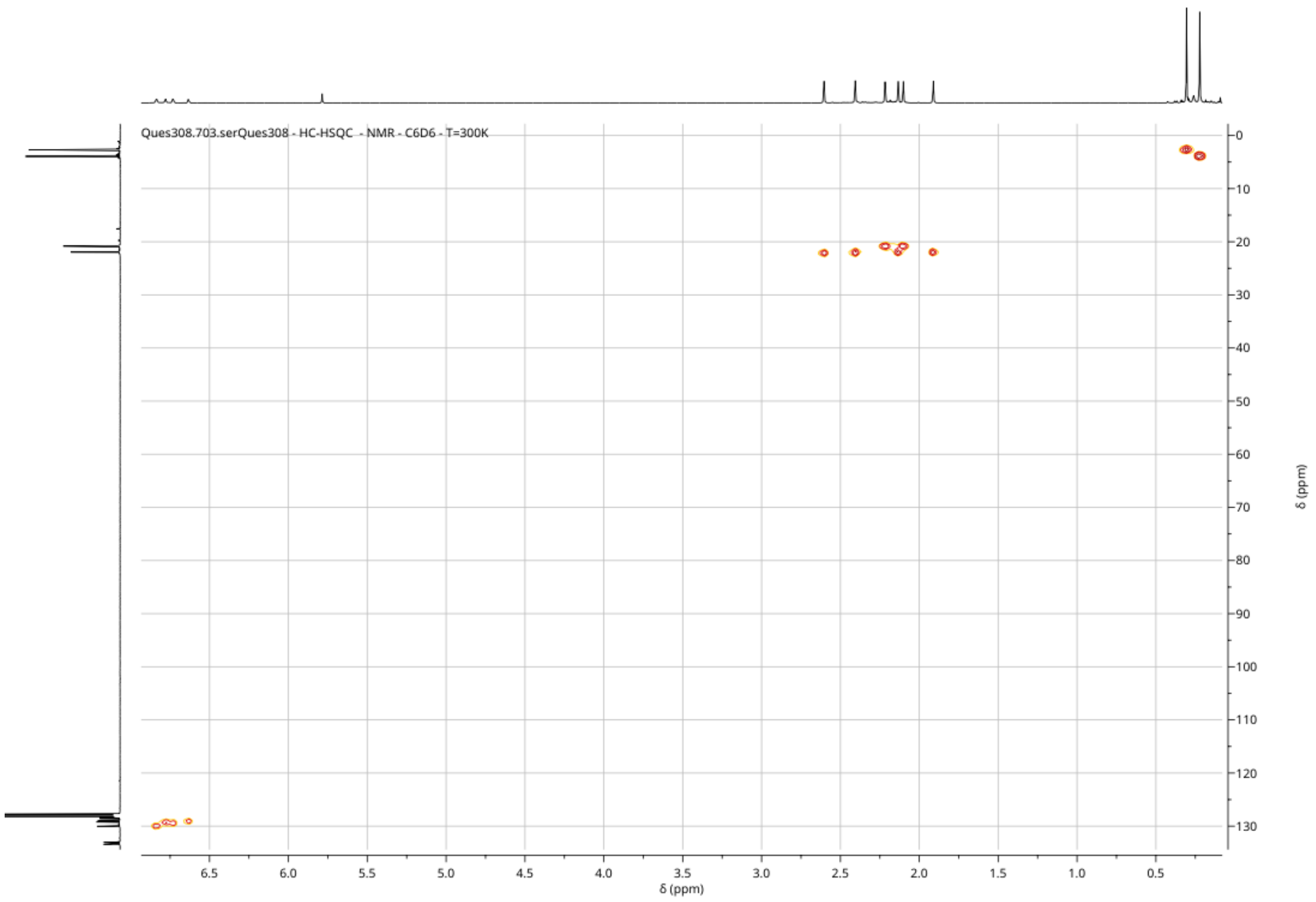

Figure S13. $^{1}H,^{13}C$ HSQC NMR spectrum of **1** in C_6D_6 at 300 K and 400 MHz (^{1}H) and 100 MHz (^{13}C).

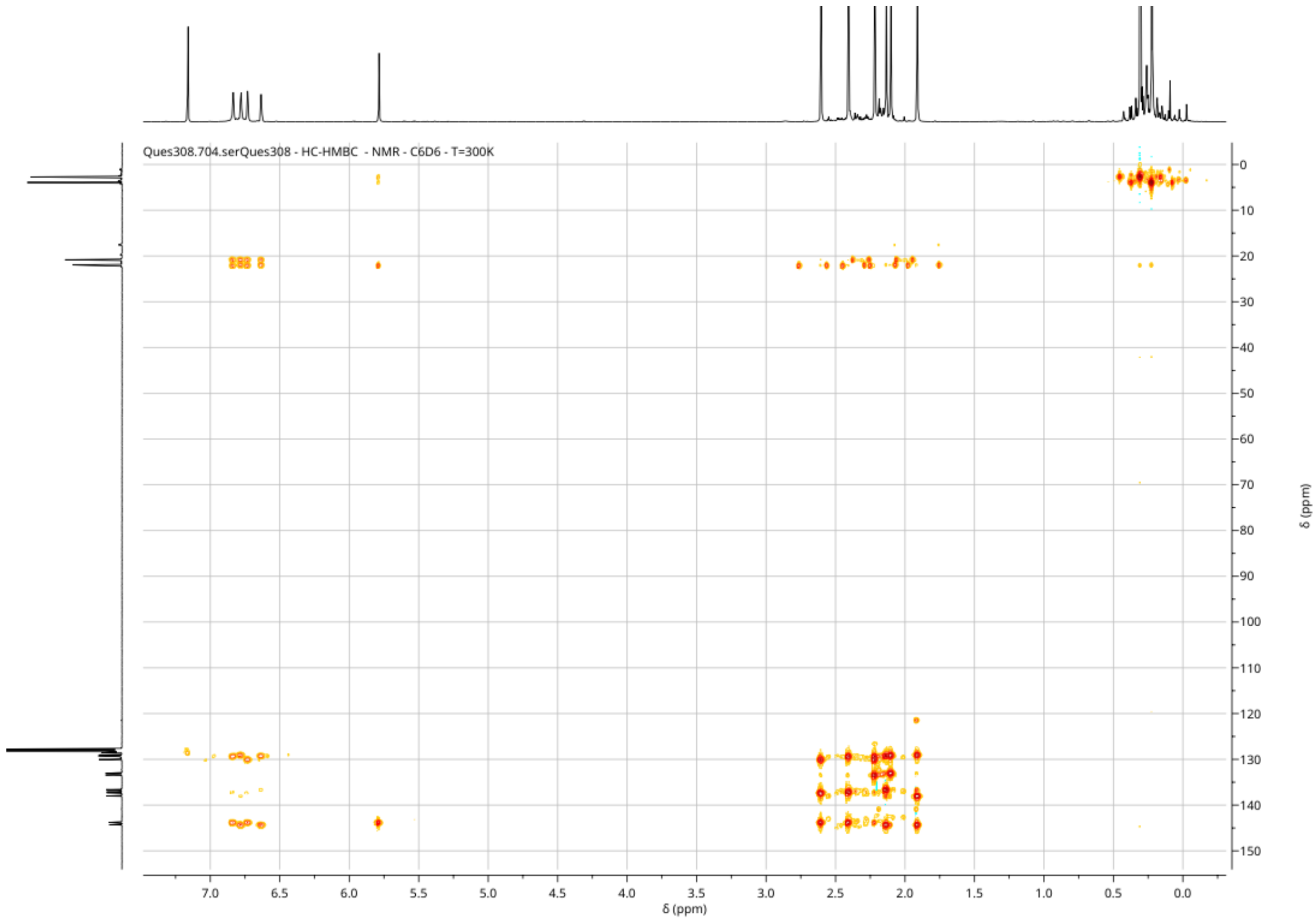

Figure S14. $^{1}H,^{13}C$ HMBC NMR spectrum of **1** in C_6D_6 at 300 K and 400 MHz (^{1}H) and 100 MHz (^{13}C).

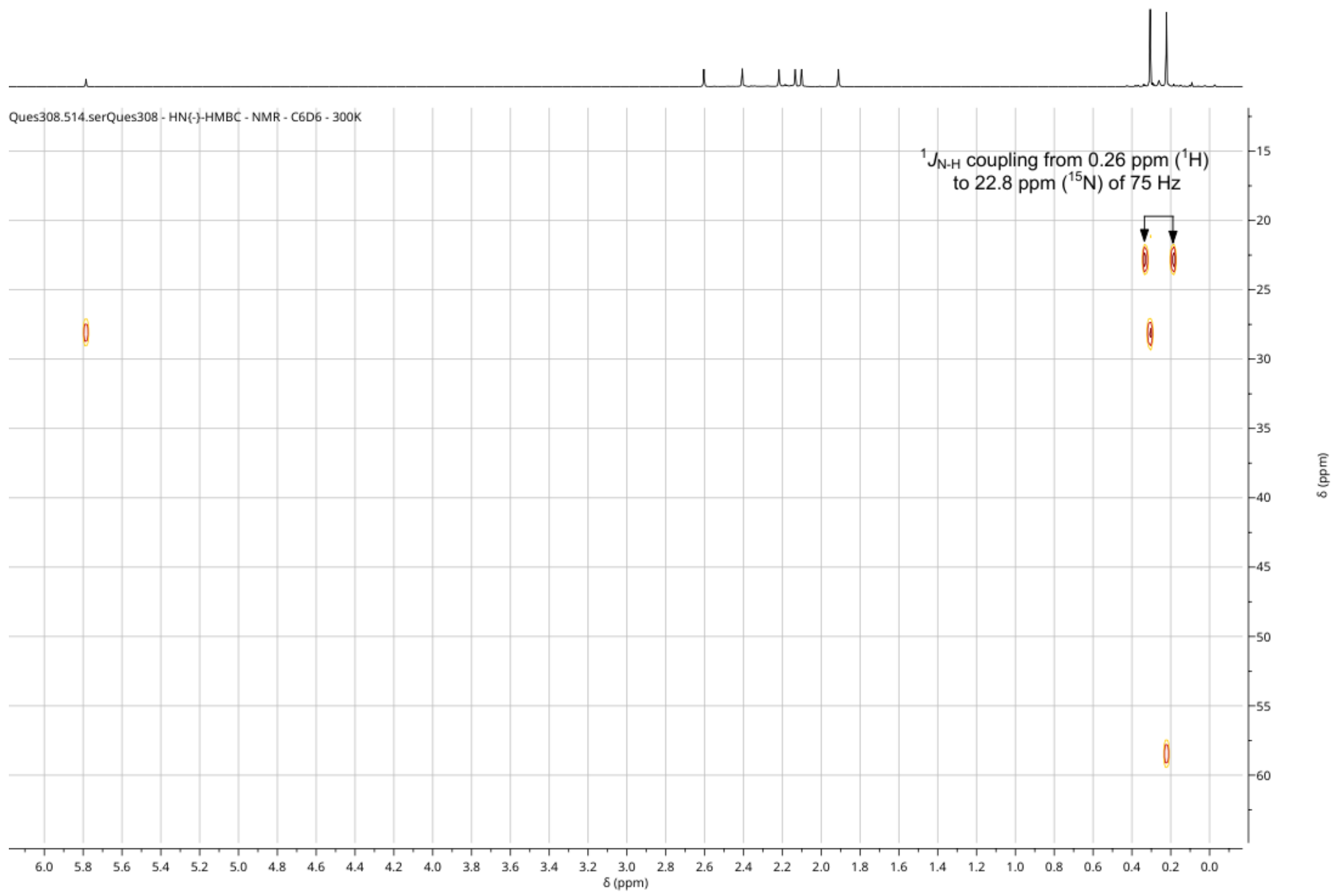

Figure S15. ^{1}H,^{15}N HMBC NMR spectrum of **1** in C_6D_6 at 300 K and 500 MHz (^{1}H) and 70 MHz (^{15}N).

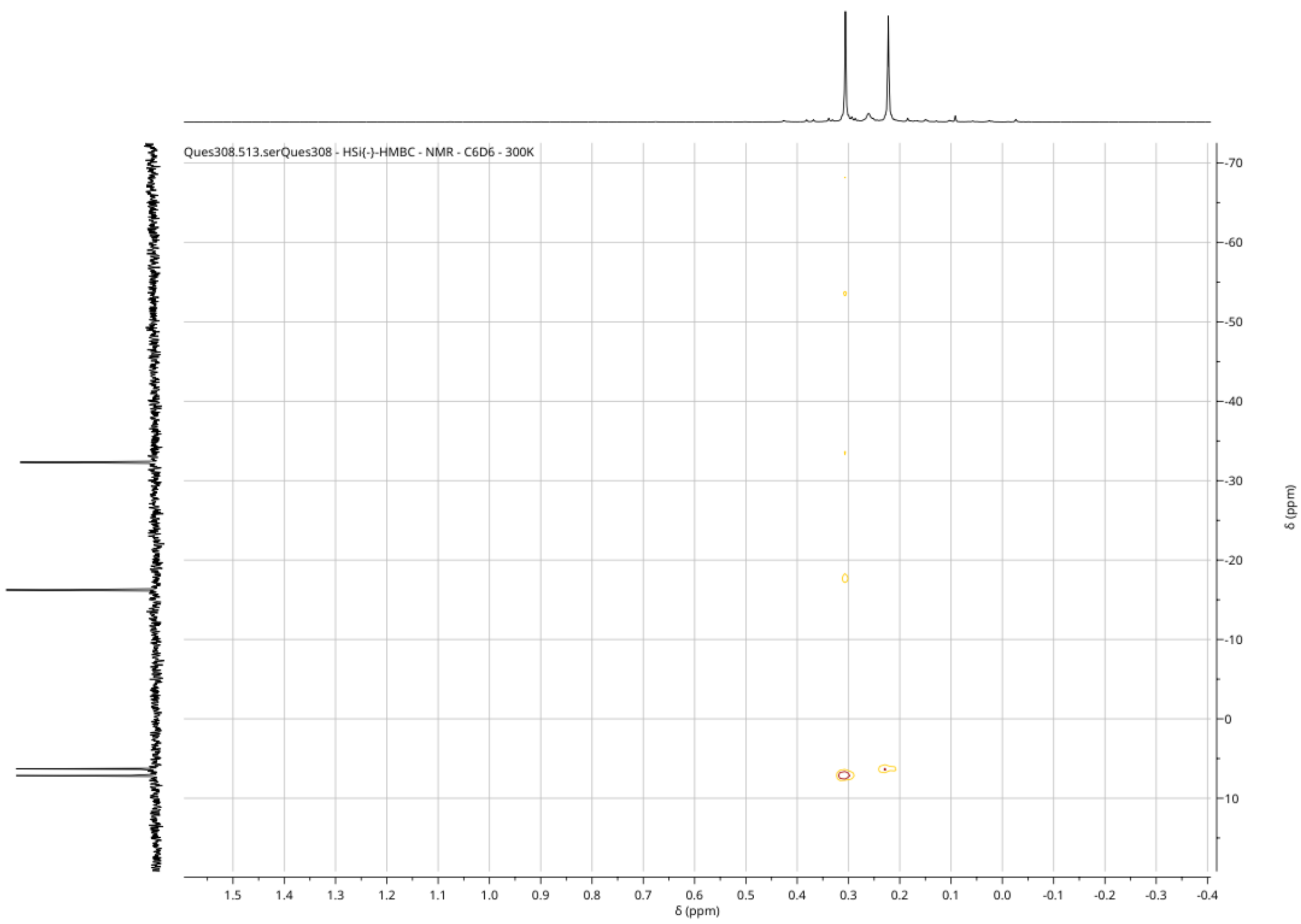

Figure S16. ^{1}H,^{29}Si HMBC NMR spectrum of **1** in C_6D_6 at 300 K and 500 MHz (^{1}H) and 100 MHz (^{29}Si).

2.2 $H_2(OH)_3Si_4\{N(SiMe_3)Mes\}_3$ (2)

Under vigorous stirring H_2O (31.0 mg, 1.72 mmol, 3.0 eq., 3.10 mL solution in THF v/v 1:100) was slowly added dropwise to a cooled (−95 °C) solution of $Si_4\{N(SiMe_3)Mes\}_4$ **F** (538.7 mg, 0.574 mmol, 1.0 eq.) in THF (30 mL) using an acetone liquid nitrogen cooling bath. During the addition, a color change from orange to yellow was observed. After the addition, the mixture was stirred for 30 min at −95 °C. The mixture was then allowed to warm to room temperature, and all volatiles were removed *in vacuo*. The yellow residue was washed with 3×2 mL *n*-hexane and dried *in vacuo*. $H_2(OH)_3Si_4\{N(SiMe_3)Mes\}_3$ **2** was obtained as yellow crystals from a saturated *n*-hexane solution.

Yield: 60.3 mg (0.0769 mmol, 13.4 %).

Melting point: 167.7–168.9 °C.

^{1}H NMR (C_6D_6, 300 K, 500.2 MHz): δ (ppm) = 6.81 (s, 2H, Mes–C$H_{arom.}$), 6.78 (s, 2H, Mes–C$H_{arom.}$), 6.70 (s, 2H, Mes-C$H_{arom.}$), 3.81 (s, 1H, SiH_2, $^1J_{H,Si}$ = 186.6 Hz, $^2J_{H,Si}$ = 10.2 Hz), 3.72 (s, 1H, SiH_2, $^1J_{H,Si}$ = 187.2 Hz, $^2J_{H,Si}$ = 19.3 Hz), 2.40 (s, 6H, *ortho*–Mes–CH_3), 2.27 (s, 6H, *ortho*–Mes–CH_3), 2.14 (s, 6H, *para*–Mes–CH_3), 2.09 (s, 3H, *para*–Mes–CH_3), 2.03 (s, 6H, *ortho*–Mes–CH_3), 1.82 (s, 2H, O*H*), 1.56 (s, 1H, O*H*), 0.28 (s, 18H, Si(CH_3)$_3$), −0.06 (s, 9H, Si(CH_3)$_3$).

^{13}C{^{1}H} NMR (C_6D_6, 300 K, 125.8 MHz): δ (ppm) = 142.63 (s, 1C, Mes–C_{ipso}), 142.56 (s, 2C, Mes–C_{ipso}), 136.3 (s, 2C, Mes–C_{ortho}), 136.0 (s, 2C, Mes–C_{ortho}), 135.9 (s, 2C, Mes–C_{ortho}), 133.2 (s, 1C, Mes–C_{para}), 132.9 (s, 2C, Mes–C_{para}), 129.2 (s, 2C, Mes–C_{meta}), 129.0 (s, 2C, Mes–C_{meta}), 128.6 (s, 2C, Mes–C_{meta}), 20.7 (s, 2C, *ortho*–Mes–*C*H_3), 20.2 (s, 2C, *ortho*–Mes–*C*H_3), 19.9 (s, 2C, *para*–Mes–*C*H_3), 19.8 (s, 1C, *para*–Mes–*C*H_3), 19.6 (s, 2C, *ortho*–Mes–*C*H_3), 2.0 (s, 6C, Si(*C*H_3)$_3$), 1.5 (s, 3C, Si(*C*H_3)$_3$).

^{13}C{^{1}H} DEPT-135 NMR (C_6D_6, 300 K, 125.8 MHz): δ (ppm) = 129.2 (s, 2 C, *meta*-Ar–*C*), 129.0 (s, 2 C, *meta*-Ar–*C*), 128.6 (s, 2 C, *meta*-Ar–*C*), 20.7 (s, 2 C, *ortho*–Ar–*C*H_3), 20.2 (s, 2 C, *ortho*–Ar–*C*H_3), 19.9 (s, 2 C, *para*–Ar–*C*H_3), 19.8 (s, 1 C, *para*–Ar–*C*H_3), 19.6 (s, 2 C, *ortho*–Ar–*C*H_3), 2.0 (s, 6 C, Si(*C*H_3)$_3$), 1.5 (s, 3 C, Si(*C*H_3)$_3$).

^{29}Si{^{1}H} IG NMR (C_6D_6, 300 K, 99.38 MHz): δ (ppm) = 7.6 (s, 2Si, (H_3C)$_3$*Si*), 6.7 (s, 1Si, (H_3C)$_3$*Si*), 3.7 (s, 1Si, *Si*), −1.6 (s, 2Si, *Si*), −93.3 (s, 1 Si, H_2*Si*).

^{29}Si{^{1}H} DEPT-19.5 NMR (C_6D_6, 79.49 MHz, 300 K): δ (ppm) = 7.6 (s, 2Si, (H_3C)$_3$*Si*), 6.7 (s, 1Si, (H_3C)$_3$*Si*), −1.6 (s, 2Si, *Si*).

^{29}Si{^{1}H} DEPT-90 NMR (C_6D_6, 300 K, 99.38 MHz): δ (ppm) = −93.3 (s, 1 Si, H_2*Si*).

^{1}H,^{15}N HMBC NMR (C_6D_6, 300 K, 500.2 MHz, 50.70 MHz): δ (ppm) = 65 (s, Mes–*N*–Si(CH_3)$_3$), 67 (s, Mes–*N*–Si(CH_3)$_3$).

IR (KBr-pellet): $\tilde{\nu}$ (cm^{-1}) = 3588 w, 3572 s, 3556 m, 3350 s, 2953 m, 2158 m, 2122 s, 1477 m, 1437 s, 1376 s, 1302 s, 1249 w, 1208 w, 1202 w, 1151 m, 1033 s, 920 vw, 872 vw, 832 w, 776 m, 756 m, 719 m, 667 s, 617 w, 610 w, 567 w, 537 w, 527 w, 472 m, 418 w.

UV-vis (*c* = 1.3 mmol/L, 0.13 mmol/L, 0.025 mmol/L in THF): λ_{max} = 334 nm, ε = 239(2) L·mol^{-1} cm^{-1}.

ESI-MS (positive mode, solution in THF): *m/z* = $[M+H]^+$ 784.3452 (784.3484 calc., Δ = 4.02 ppm), $[M+Na]^+$ 806.3296 (806.3303 calc., Δ = 0.87 ppm).

CHN for $C_{36}H_{65}N_3O_3Si_7$: calc.: C 55.1 %, H 8.4 %, N 5.4 %; found: C 55.0 %, H 8.3 %, N 5.2 %.

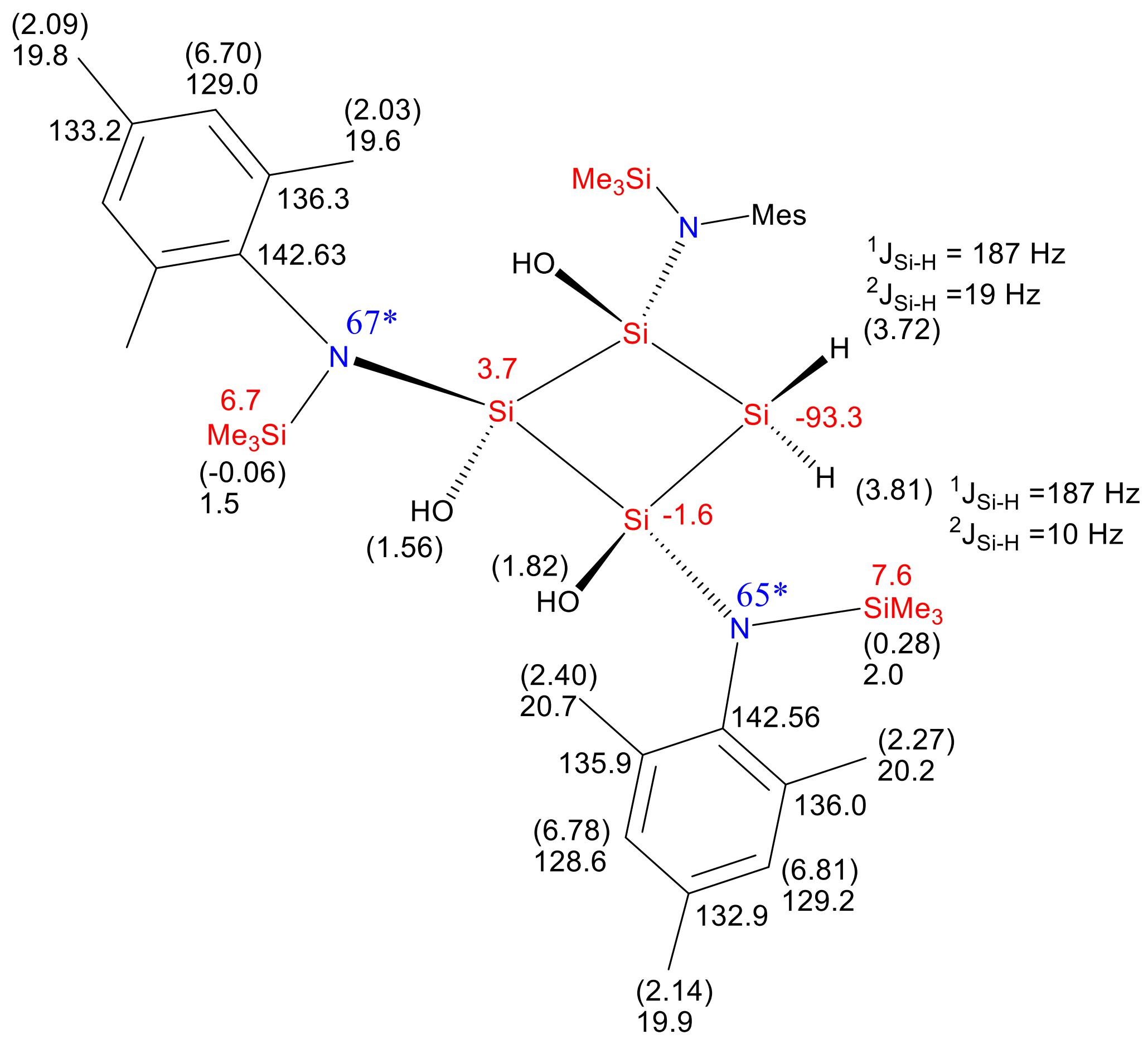

Figure S17. Assignment of chemical shifts to **2** (in C_6D_6 at 300 K). Color coding: ^{1}H black in brackets, ^{13}C black, ^{15}N blue, and ^{29}Si red.

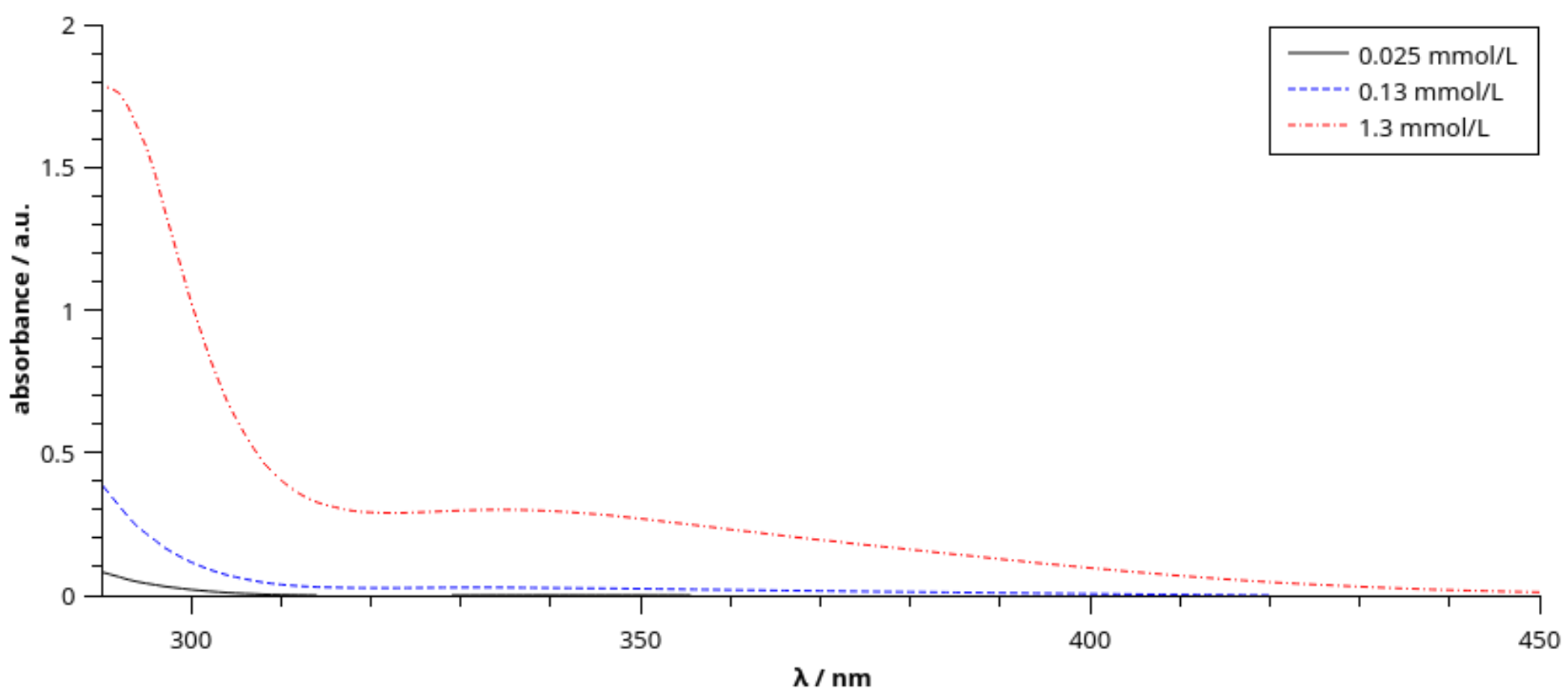

Figure S18. UV-Vis spectra of **2** in THF.

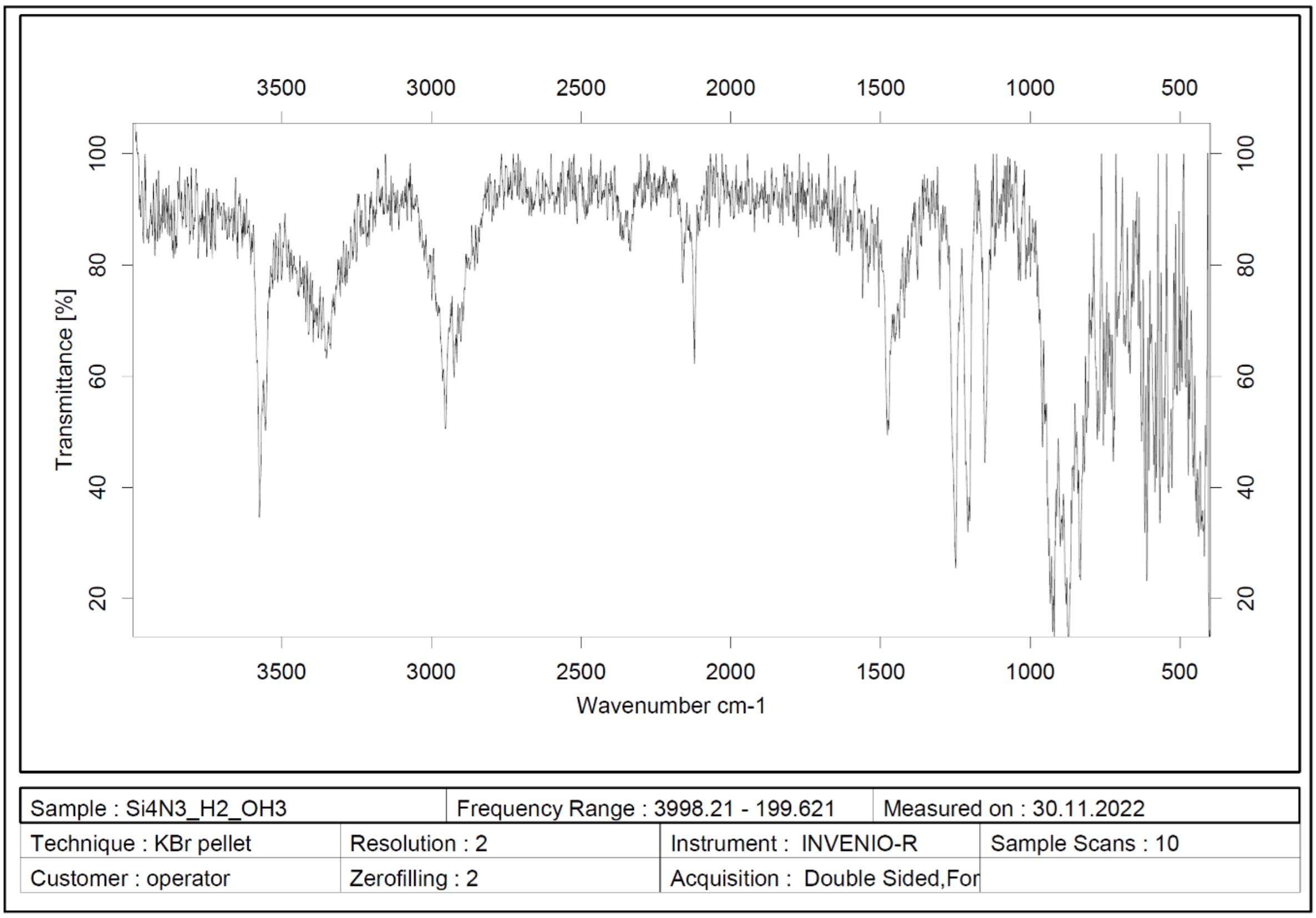

Figure S19. FT-IR spectrum of **2** (KBr-pellet).

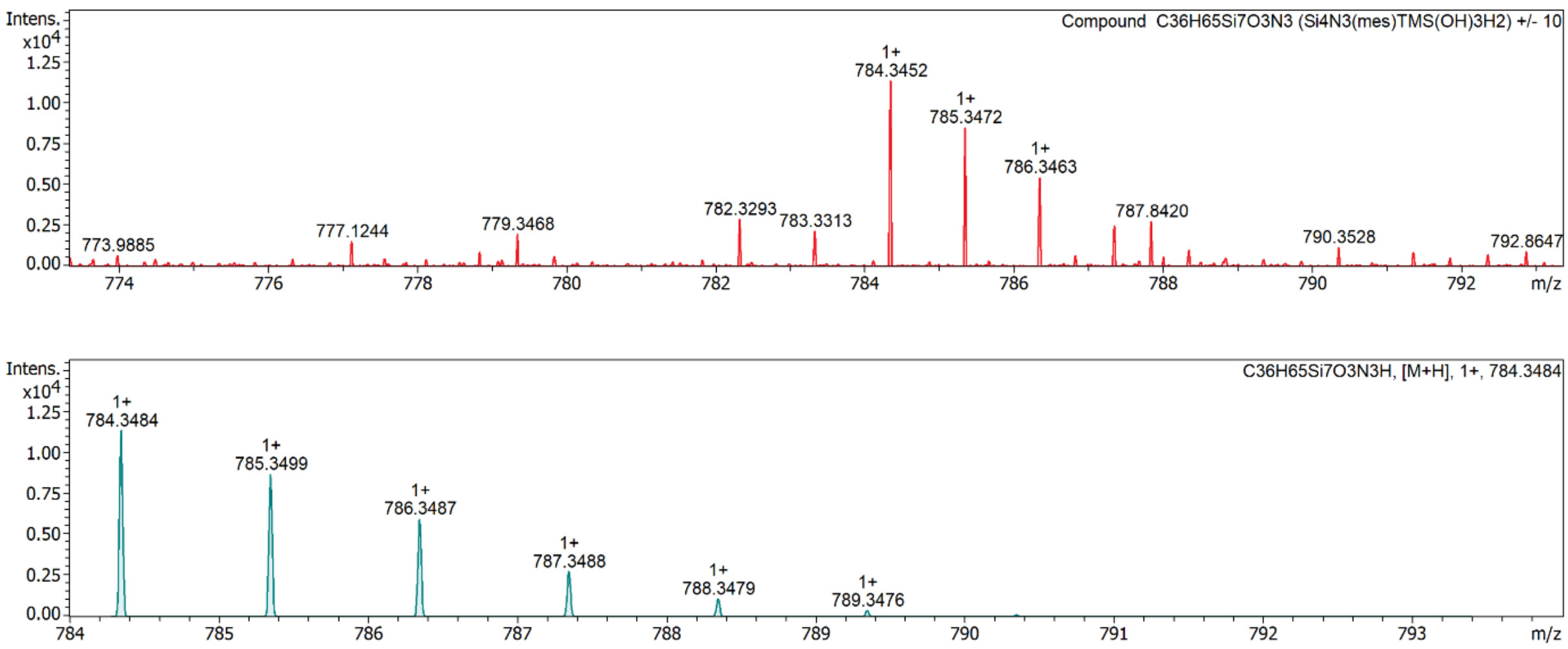

Figure S20. ESI-MS spectrum (positive mode) of **2**.

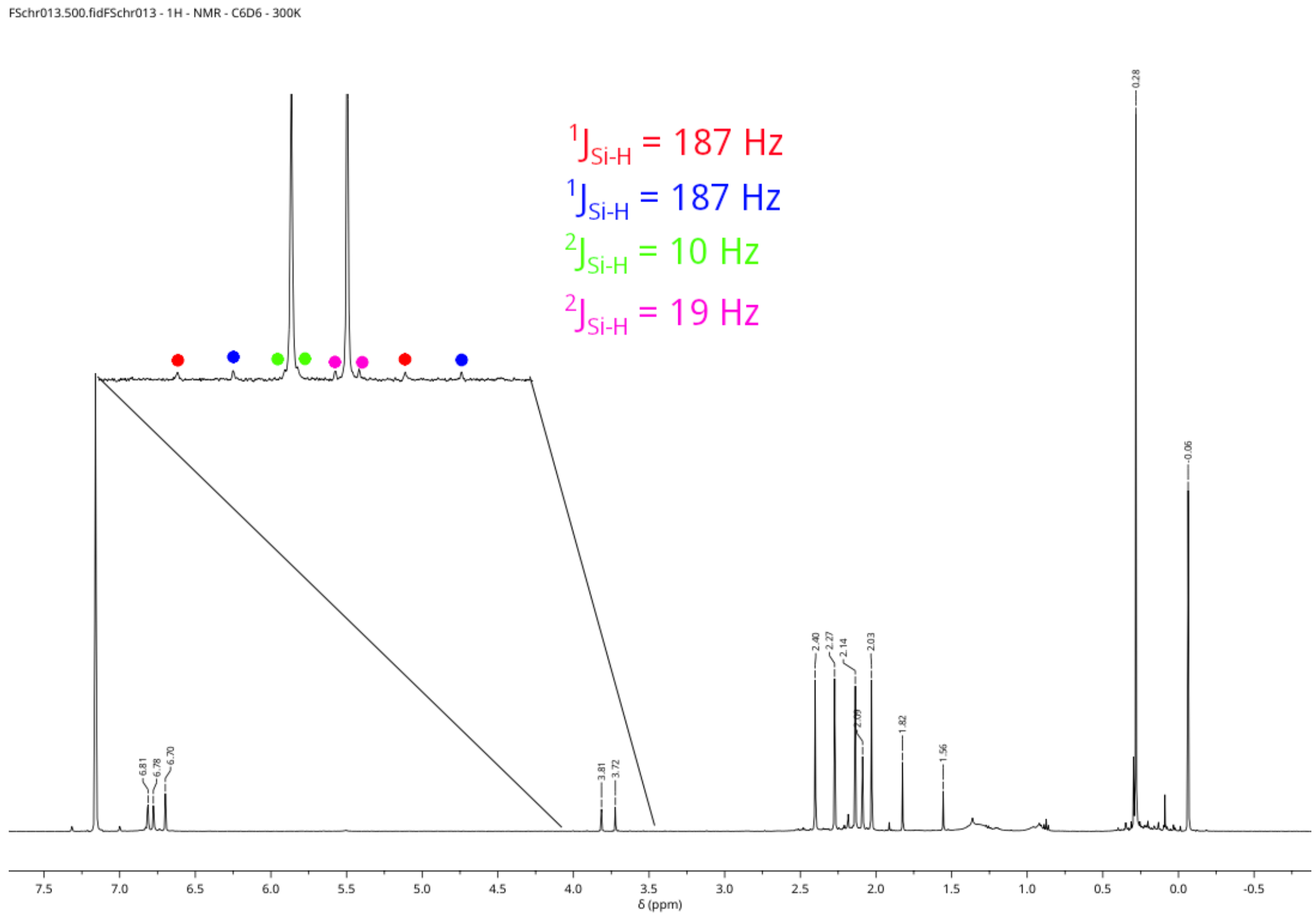

Figure S21. ^{1}H NMR spectrum of **2** in C_6D_6 at 300 K and 500 MHz.

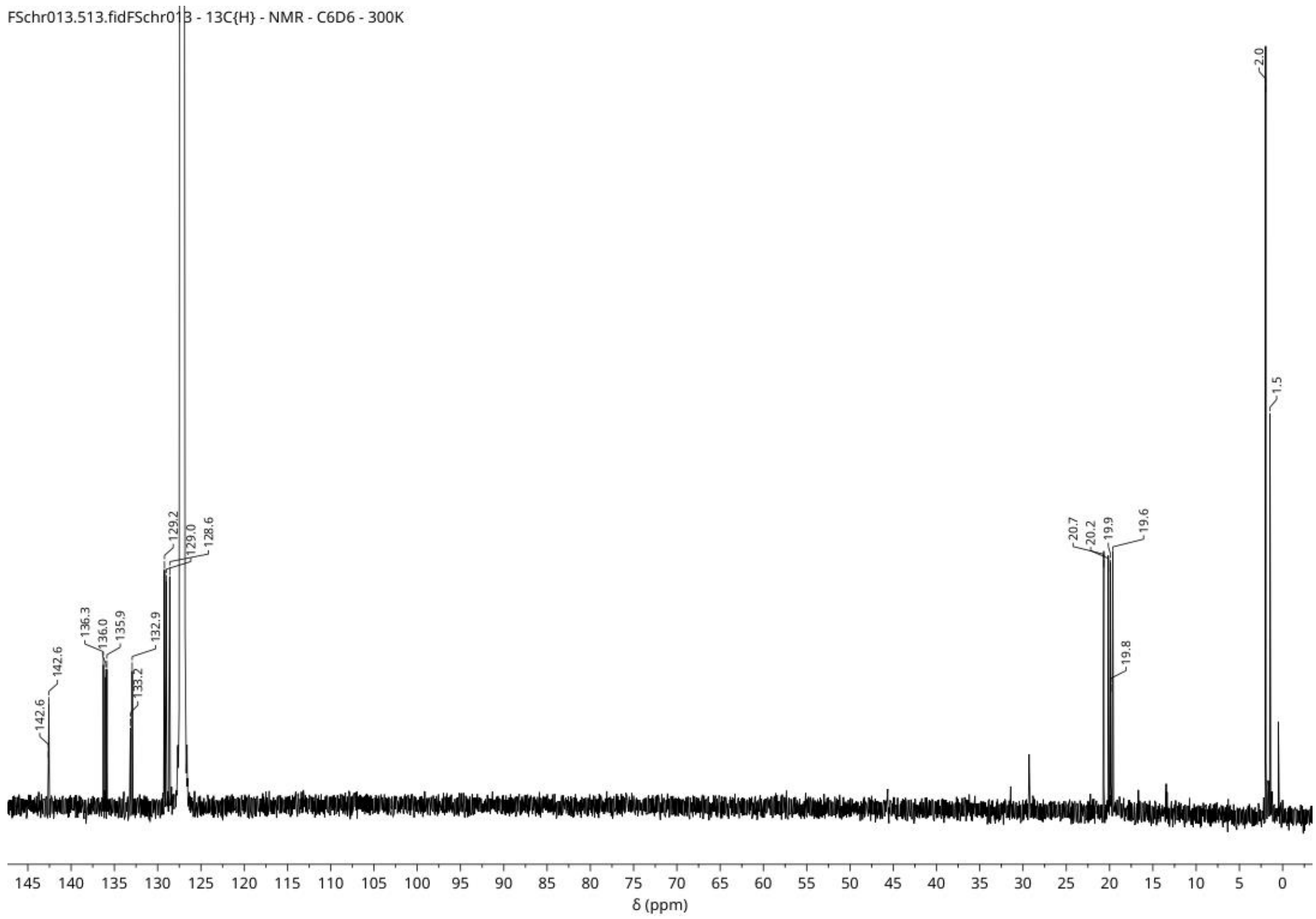

Figure S22. ^{13}C{^{1}H} NMR spectrum of **2** in C_6D_6 at 300 K and 126 MHz.

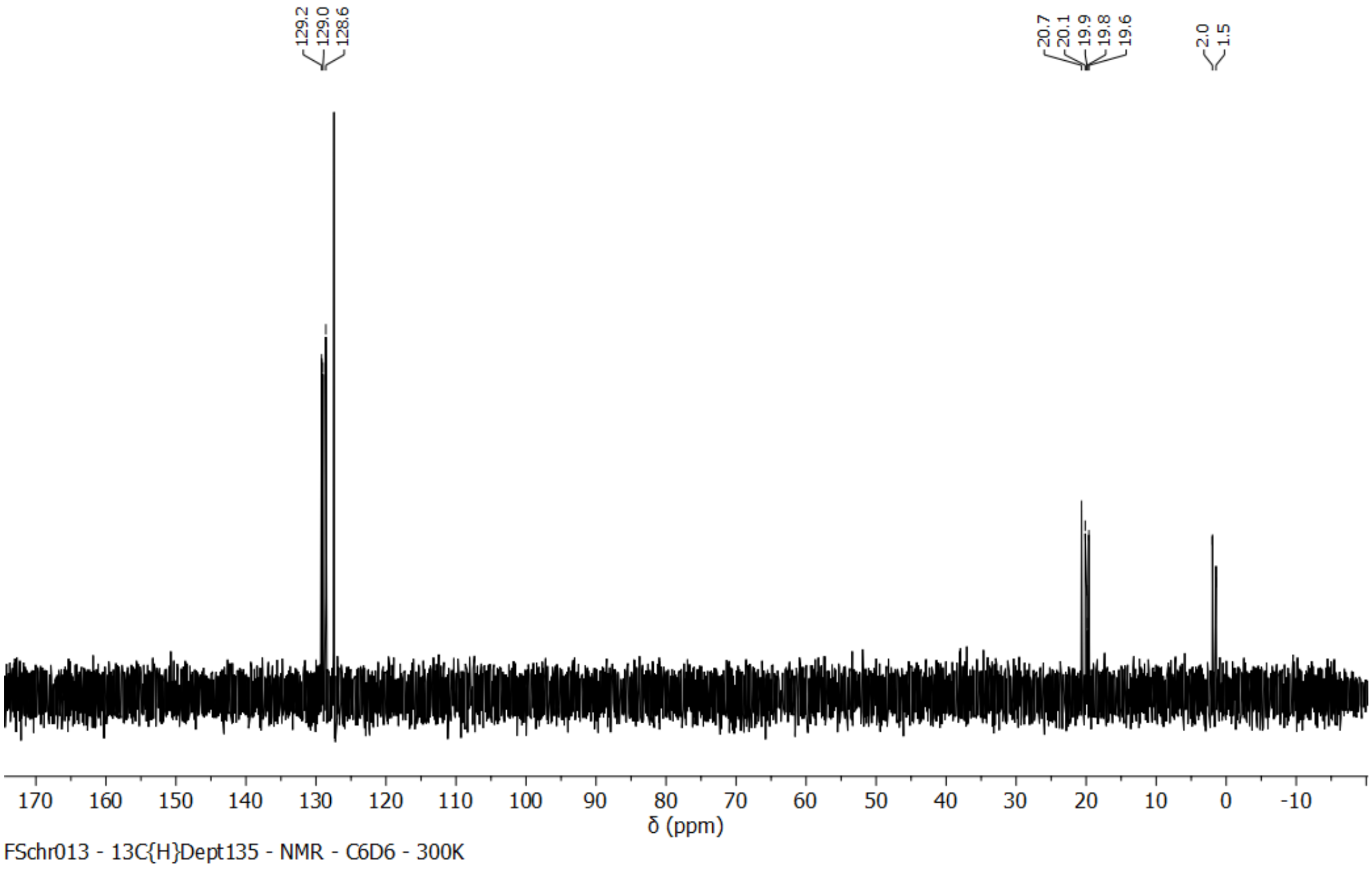

Figure S23. $^{13}C\{^{1}H\}$ DEPT-135 NMR spectrum of **2** in C_6D_6 at 300 K and 126 MHz.

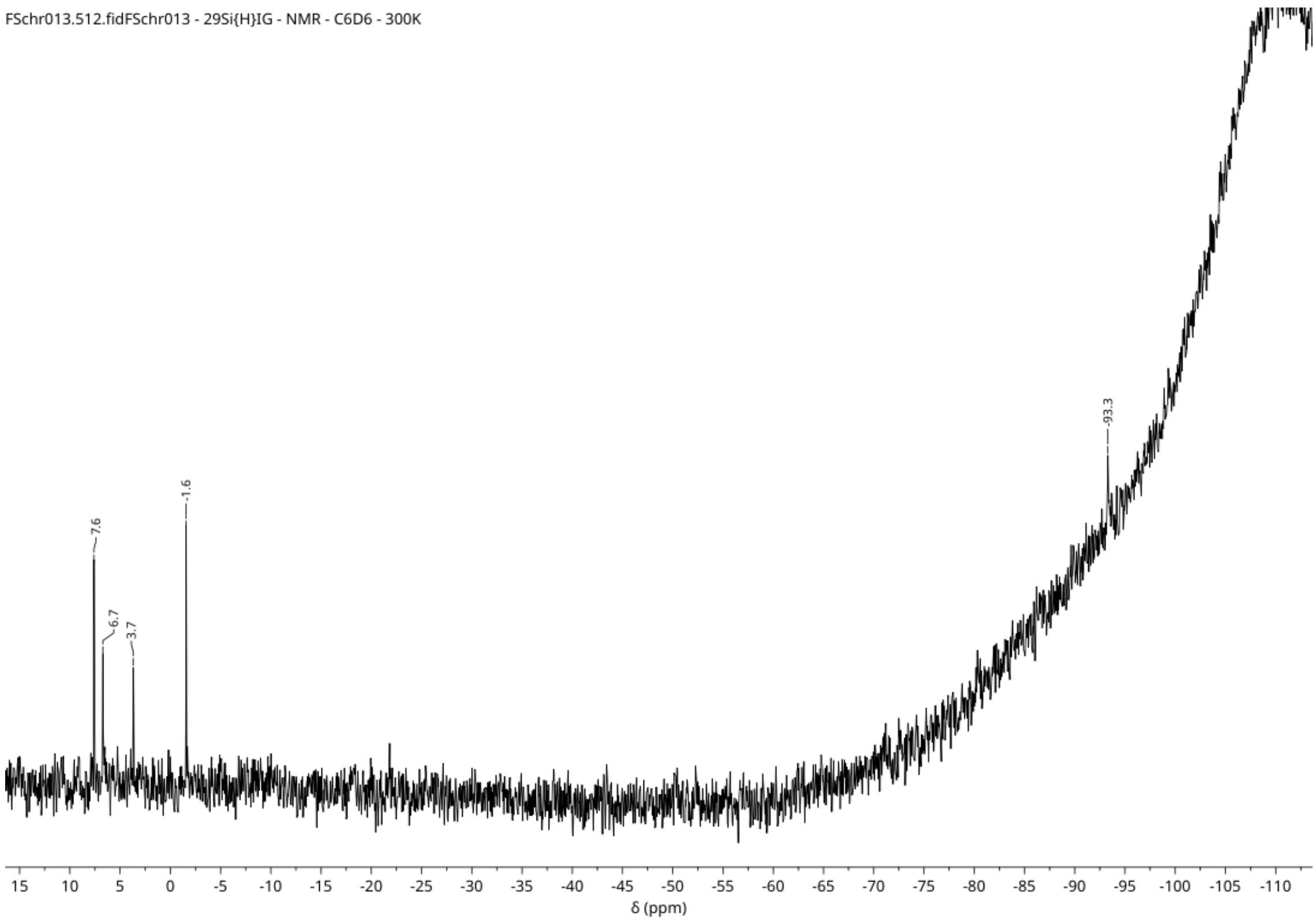

Figure S24. $^{29}Si\{^{1}H\}$ IG NMR spectrum of **2** in C_6D_6 at 300 K and 99 MHz.

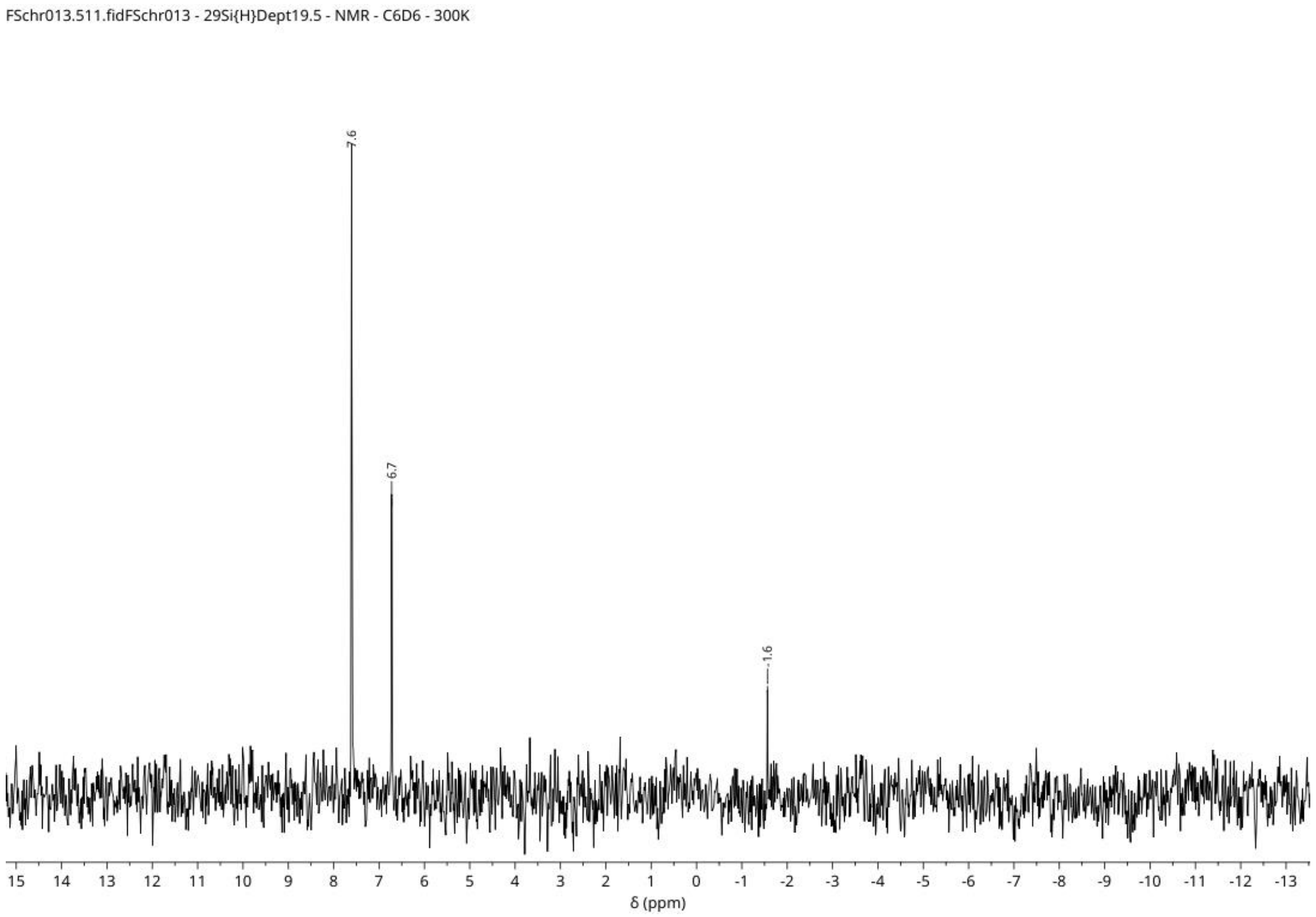

Figure S25. $^{29}Si\{^{1}H\}$ DEPT-19.5 NMR spectrum of **2** in C_6D_6 at 300 K and 99 MHz.

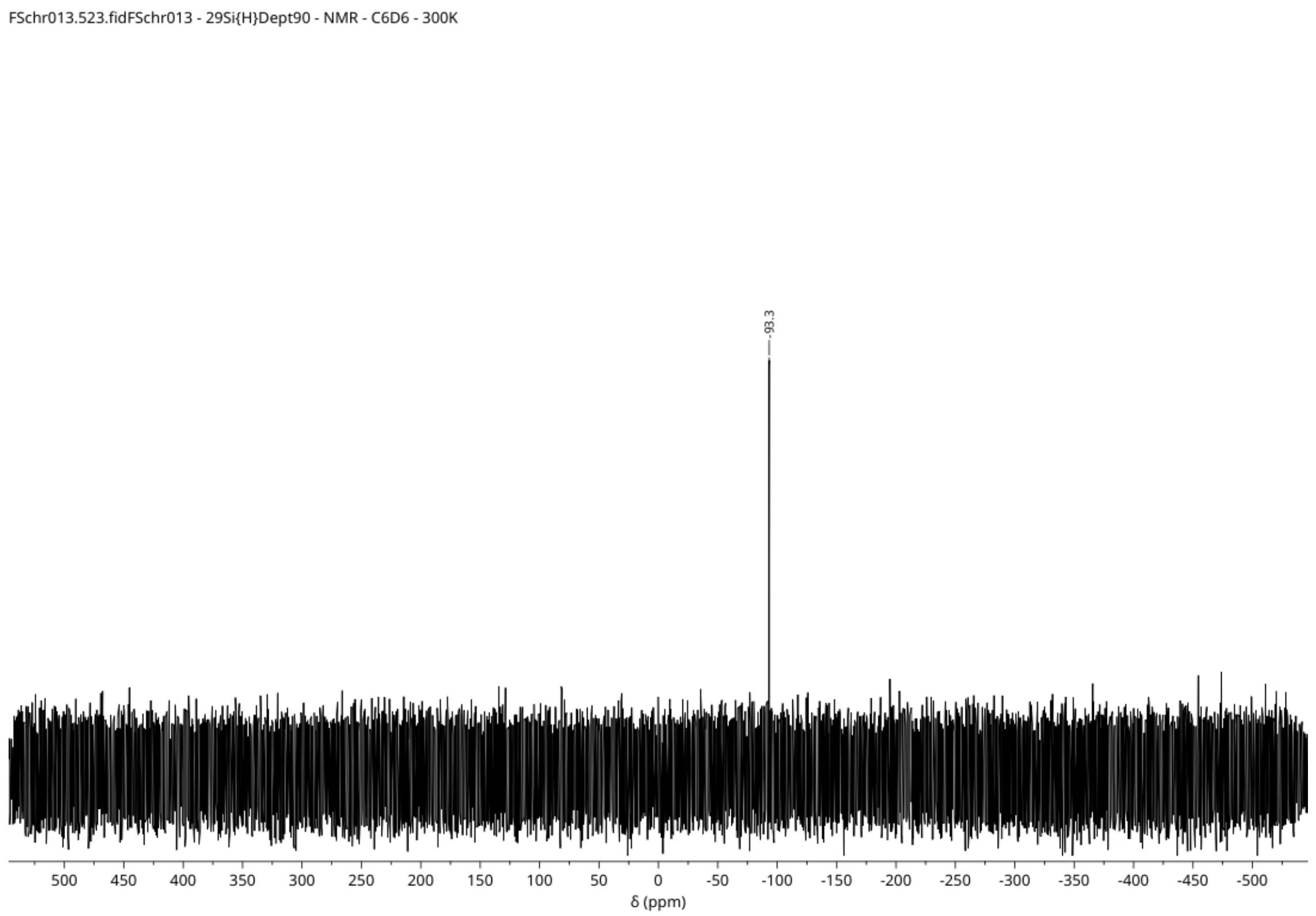

Figure S26. $^{29}Si\{^{1}H\}$ DEPT-90 NMR spectrum of **2** in C_6D_6 at 300 K and 99 MHz.

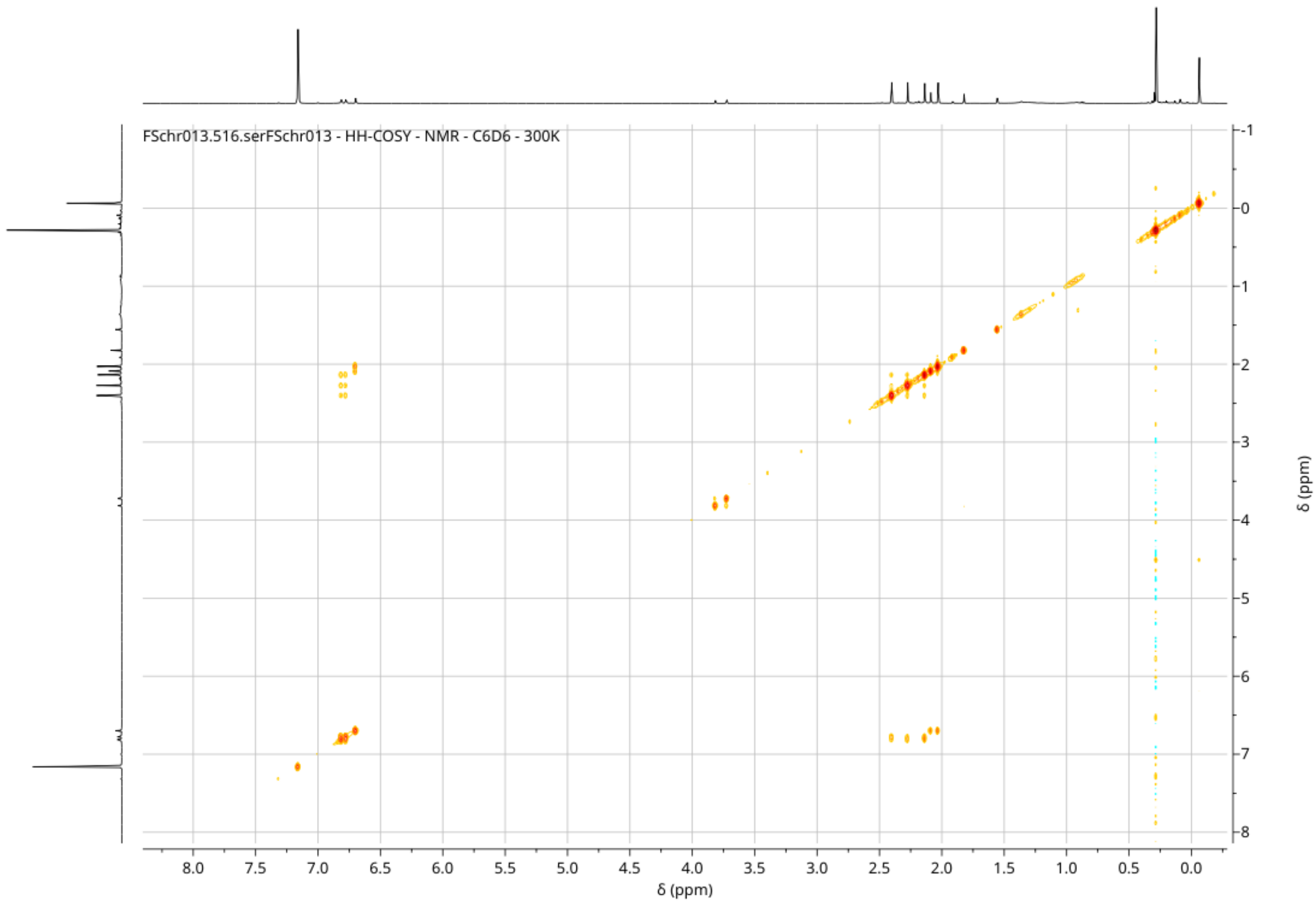

Figure S27. $^{1}H,^{1}H$ COSY NMR spectrum of **2** in C_6D_6 at 300 K and 500 MHz.

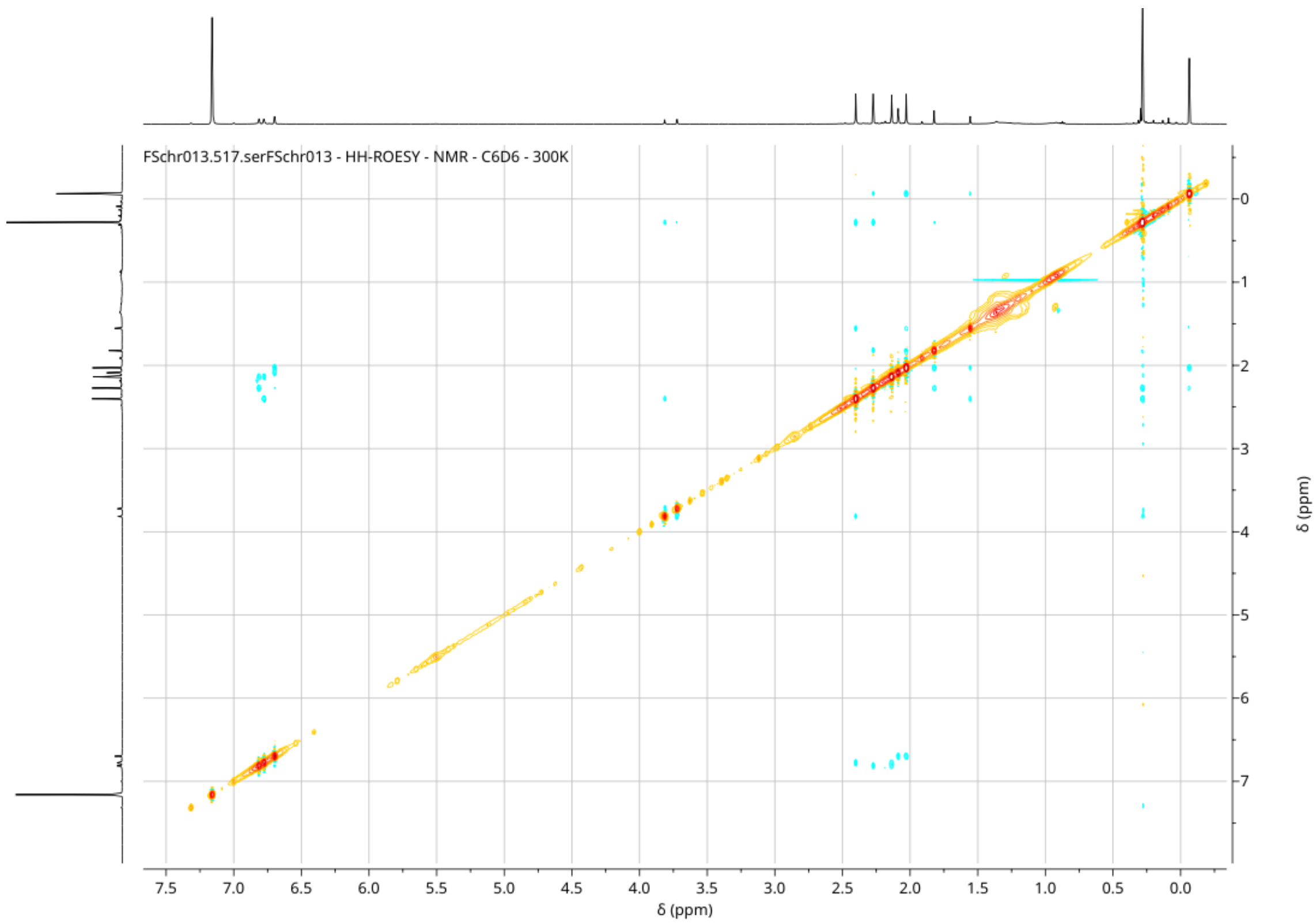

Figure S28. $^{1}H,^{1}H$ ROESY NMR spectrum of **2** in C_6D_6 at 300 K and 500 MHz.

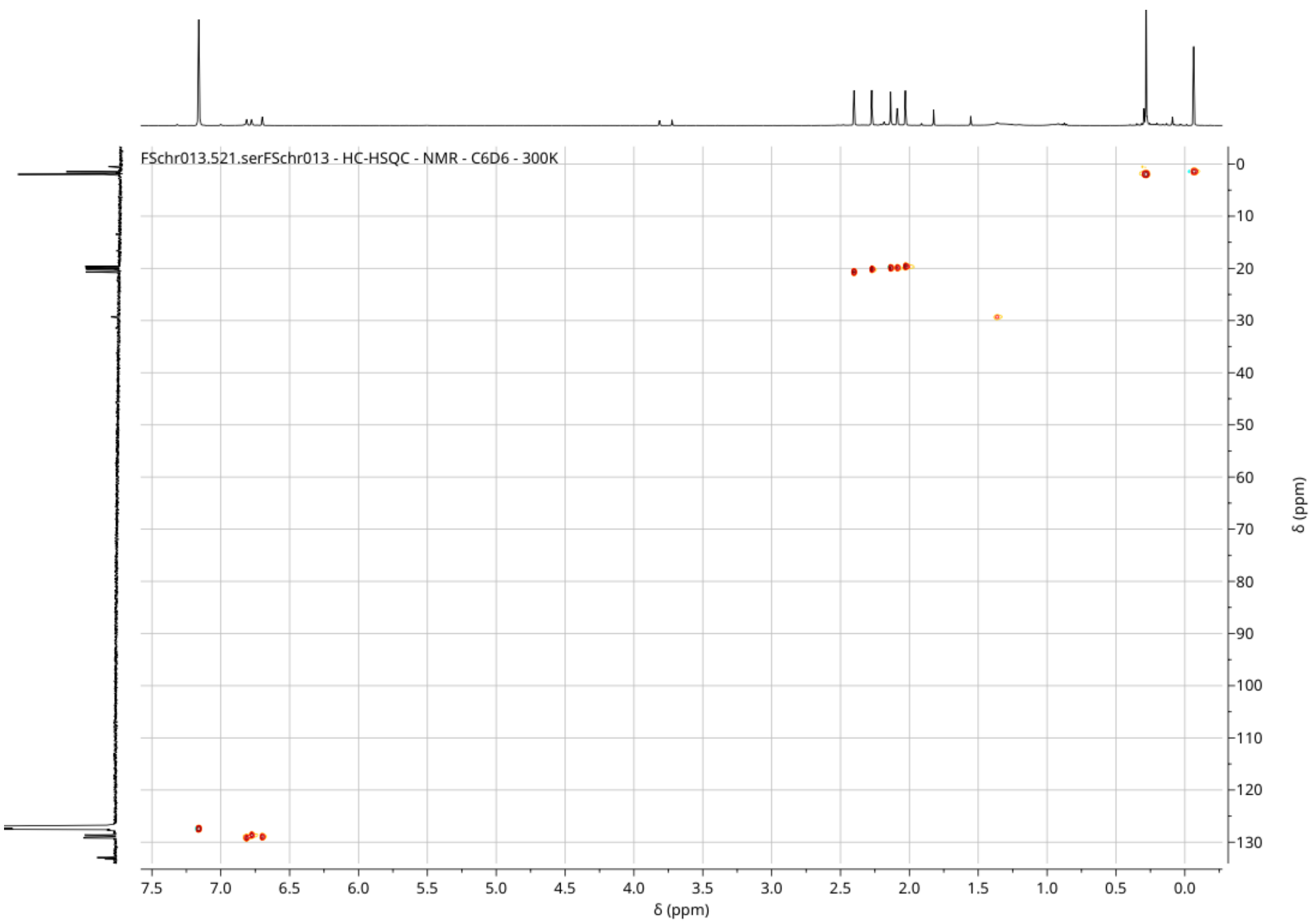

Figure S29. $^{1}H,^{13}C$ HSQC NMR spectrum of **2** in C_6D_6 at 300 K and 500 MHz (^{1}H) and 126 MHz (^{13}C).

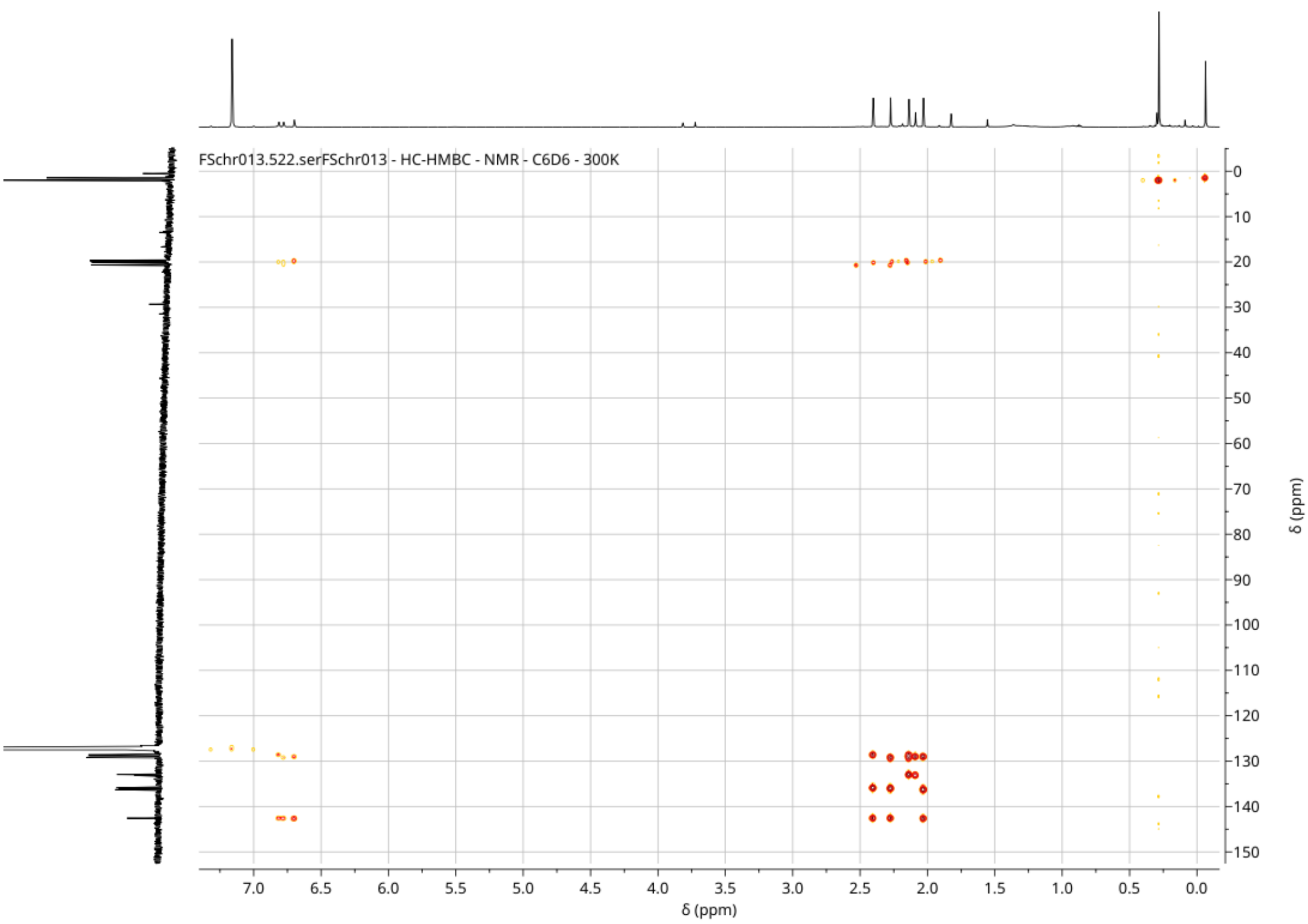

Figure S30. $^{1}H,^{13}C$ HMBC NMR spectrum of **2** in C_6D_6 at 300 K and 500 MHz (^{1}H) and 126 MHz (^{13}C).

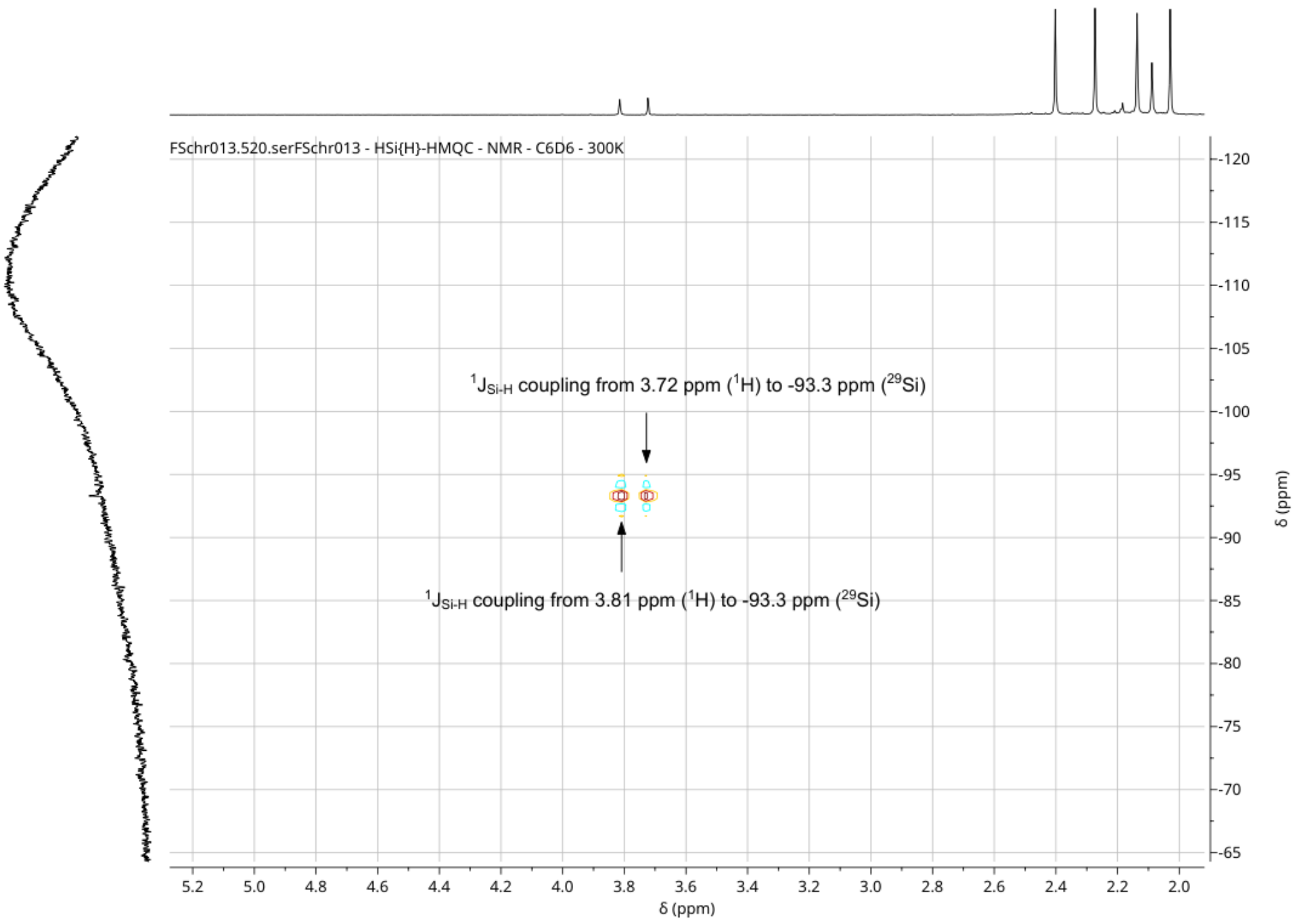

Figure S31. $^{1}H,^{29}Si$ HMQC NMR spectrum of **2** in C_6D_6 at 300 K and 500 MHz (^{1}H) and 99 MHz (^{29}Si).

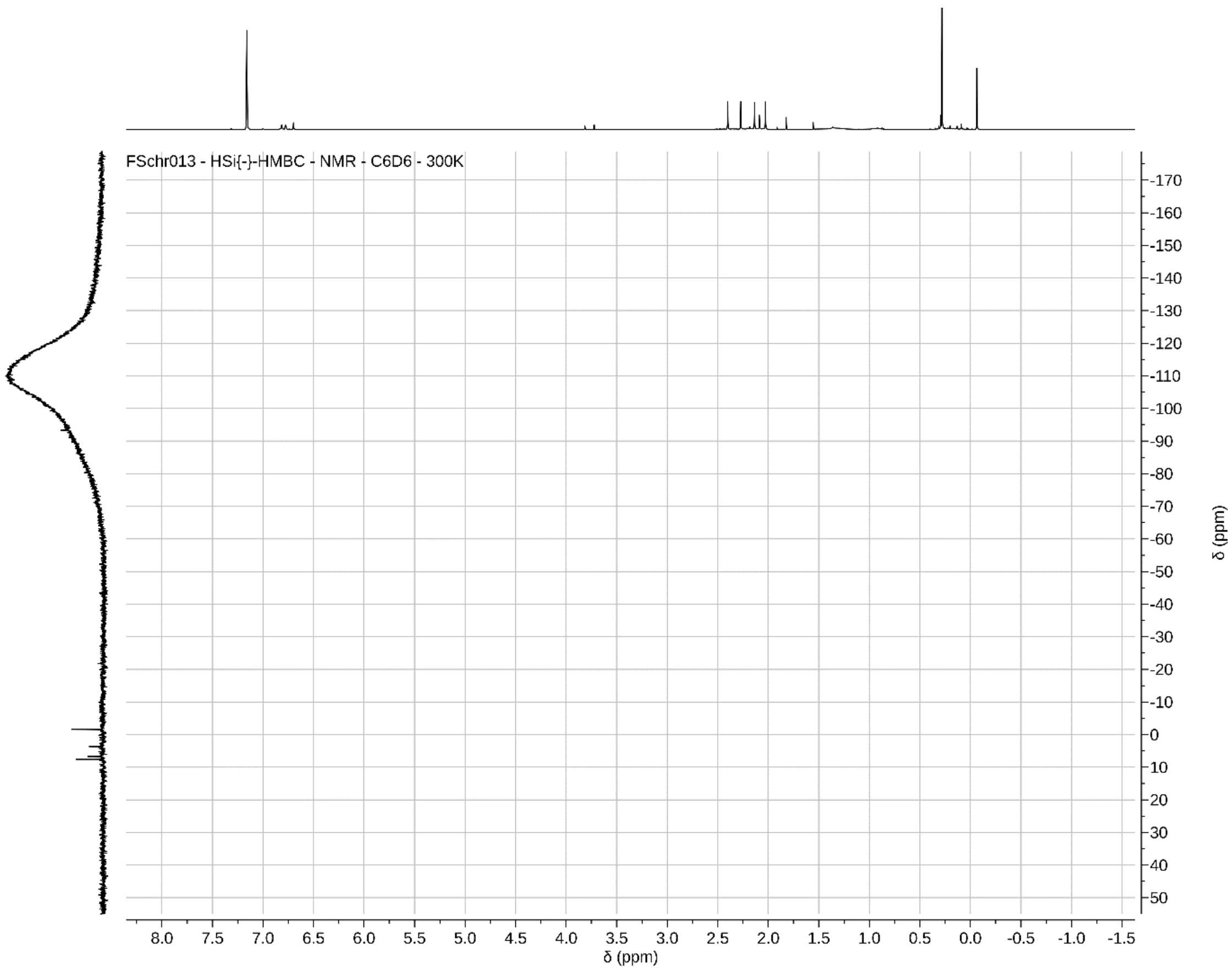

Figure S32. $^{1}H,^{29}Si$ HMBC NMR spectrum of **2** in C_6D_6 at 300 K and 500 MHz (^{1}H) and 99 MHz (^{29}Si).

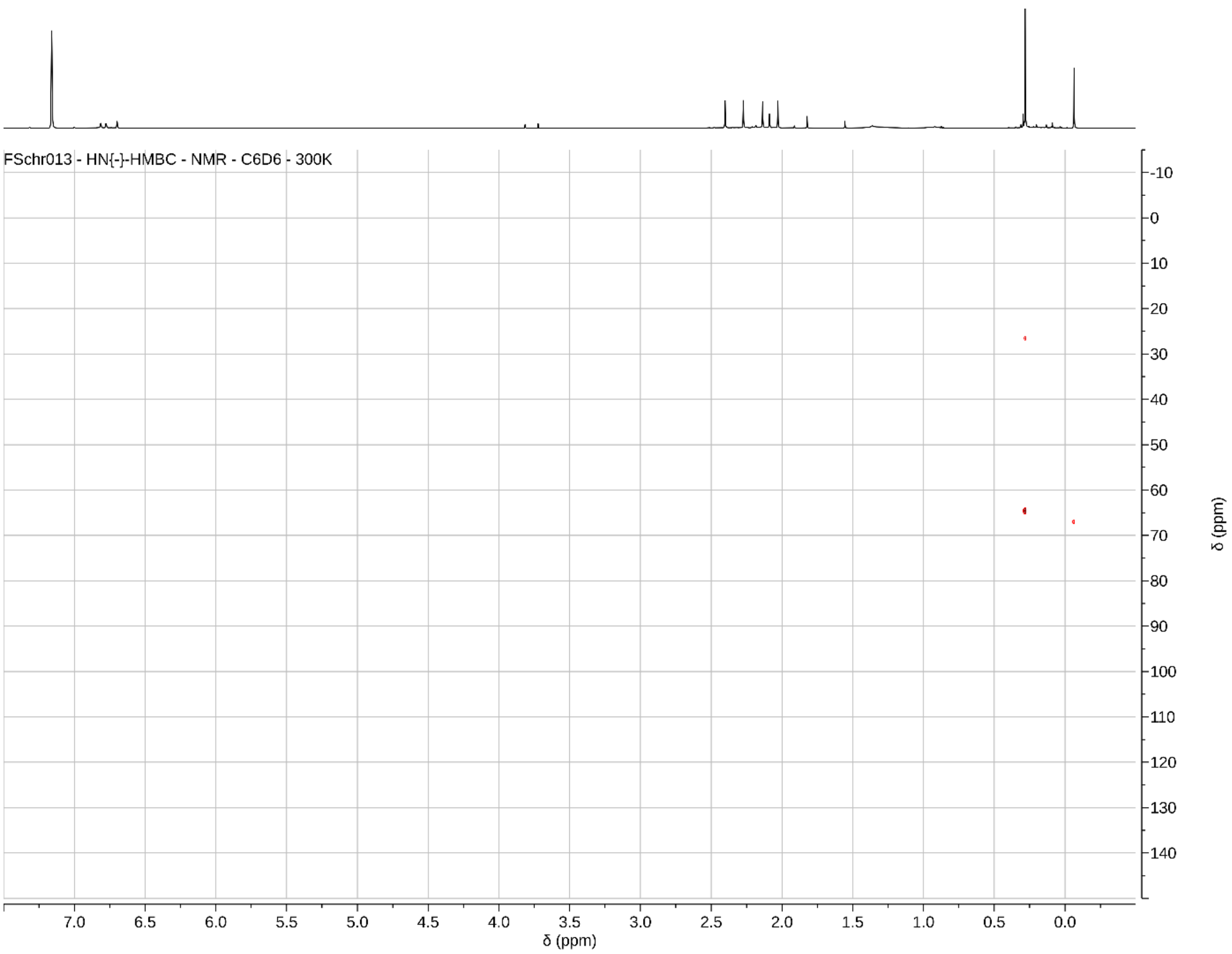

Figure S33. $^{1}H,^{15}N$ HMBC NMR spectrum of **2** in C_6D_6 at 300 K and 500 MHz (^{1}H) and 50.7 MHz (^{15}N).

2.3 $D_2(OD)_3Si_4\{N(SiMe_3)Mes\}_3$ (2')

Under vigorous stirring D_2O (36.3 mg, 1.73 mmol, 3.0 eq., 3.63 mL solution in THF v/v 1:100) was slowly added dropwise to a cooled (−95 °C) solution of $Si_4\{N(SiMe_3)Mes\}_4$ **F** (540.0 mg, 0.5758 mmol, 1.0 eq.) in THF (30 mL) using an acetone liquid nitrogen cooling bath. During the addition, a color change from orange to yellow was observed. After the addition, the mixture was stirred for 30 min at −95 °C. The mixture was then allowed to warm to room temperature, and all volatiles were removed *in vacuo*. The yellow residue was washed with 3×1 mL *n*-hexane and dried *in vacuo*. The product **2'** was obtained as yellow crystals from a saturated *n*-hexane solution.

Yield: 52.3 mg (0.0662 mmol, 11.5 %).

Melting point: 171.8–173.3 °C.

^{1}H NMR (C_6D_6, 300 K, 500.2 MHz): δ (ppm) = 6.80 (s, 2H, Mes–C$H_{arom.}$), 6.76 (s, 2H, Mes–C$H_{arom.}$), 6.69 (s, 2H, Mes–C$H_{arom.}$), 2.39 (s, 6H, *ortho*–Mes–CH_3), 2.26 (s, 6H, *ortho*–Mes–CH_3), 2.13 (s, 6H, *para*–Mes–CH_3), 2.09 (s, 3H, *para*–Mes–CH_3), 2.01 (s, 3H, *ortho*–Mes–CH_3), 0.27 (s, 18H, Si(CH_3)$_3$), −0.08 (s, 9H, Si(CH_3)$_3$).

^{13}C{^{1}H} NMR (C_6D_6, 300 K, 125.8 MHz): δ (ppm) = 143.5 (s, 2C, Mes–C_{ipso}), 143.4 (s, 1C, Mes–C_{ipso}), 137.2 (s, 2C, Mes–C_{ortho}), 136.9 (s, 2C, Mes–C_{ortho}), 136.8 (s, 2C, Mes–C_{ortho}), 134.0 (s, 1C, Mes–C_{para}), 133.8 (s, 2C, Mes–C_{para}), 130.1 (s, 2C, Mes–C_{meta}), 129.9 (s, 2C, Mes–C_{meta}), 129.5 (s, 2C, Mes–C_{meta}), 21.6 (s, 2C, *ortho*–Mes–CH$_3$), 21.1 (s, 2C, *ortho*–Mes–CH$_3$), 20.83 (s, 2C, *para*–Mes–CH$_3$), 20.80 (s, 1C, *para*–Mes–CH$_3$), 20.5 (s, 2C, *ortho*–Mes–CH$_3$), 2.9 (s, 6C, Si(CH$_3$)$_3$), 2.4 (s, 3C, Si(CH$_3$)$_3$).

^{13}C{^{1}H} DEPT-135 NMR (C_6D_6, 300 K, 125.8 MHz): 130.1 (s, 2C, Mes–C_{meta}), 129.9 (s, 2C, Mes–C_{meta}), 129.5 (s, 2C, Mes–C_{meta}), 21.6 (s, 2C, *ortho*–Mes–CH$_3$), 21.1 (s, 2C, *ortho*–Mes–CH$_3$), 20.83 (s, 2C, *para*–Mes–CH$_3$), 20.80 (s, 1C, *para*–Mes–CH$_3$), 20.5 (s, 2C, *ortho*–Mes–CH$_3$), 2.9 (s, 6C, Si(CH$_3$)$_3$), 2.4 (s, 3C, Si(CH$_3$)$_3$).

^{29}Si{^{1}H} NMR (C_6D_6, 300 K, 99.38 MHz): δ (ppm) = 7.6 (s, 2Si, (H$_3$C)$_3$*Si*), 6.7 (s, 1Si, (H$_3$C)$_3$*Si*), 3.7 (s, 1Si, *Si*), −1.6 (s, 2Si, *Si*), −94.4 (quint, 1Si, D$_2$*Si*, $^1J_{D,Si}$ = 29.2 Hz).

^{29}Si{^{1}H} DEPT-19.5 NMR (C_6D_6, 300 K, 99.38 MHz): δ (ppm) = 7.6 (s, 2 Si, (H$_3$C)$_3$*Si*), 6.7 (s, 1 Si, (H$_3$C)$_3$*Si*).

^{29}Si{^{1}H} DEPT-90 NMR (C_6D_6, 300 K, 99.38 MHz): δ (ppm) = 7.6 (s, 2 Si, (H$_3$C)$_3$*Si*), 6.7 (s, 1 Si, (H$_3$C)$_3$*Si*).

^{1}H,^{15}N HMBC NMR (C_6D_6, 300 K, 500.2 MHz, 50.70 MHz): δ (ppm) = 64 (s, Mes–*N*–Si(CH$_3$)$_3$), 66 (s, Mes–*N*–Si(CH$_3$)$_3$).

IR (KBr-pellet): $\tilde{\nu}$ (cm^{-1}) = 2850 vs, 2810 vs, 2684 w, 2642 m, 2611 w, 2501 vs, 2481 vs, 2363 s, 2359 s, 2206 vs, 1560 w, 1556 w, 1482 vs, 1476 s, 1457 w, 1248 m, 1208 m, 1154 s, 1148 s, 959 s, 948 s, 926 w, 888 m, 874 w, 871 w, 828 m, 621 w, 608 w, 593 m, 576 m, 570 m, 566 m, 526 m, 488 m, 472 m, 465 m, 444 m, 433 w, 409 w, 399 w, 393 w, 384 w, 379 w, 374 w, 354 w, 342 vw.

UV-vis (c = 1.3 mmol/L, 0.13 mmol/L, 0.025 mmol/L in THF): λ_{max} = 338 nm, ε = 185(4) $L \cdot mol^{-1} \cdot cm^{-1}$.

ESI-MS (positive mode, solution in THF): m/z = $[M+Na]^+$ 811.3580 (811.3617 calc., Δ = 4.54 ppm).

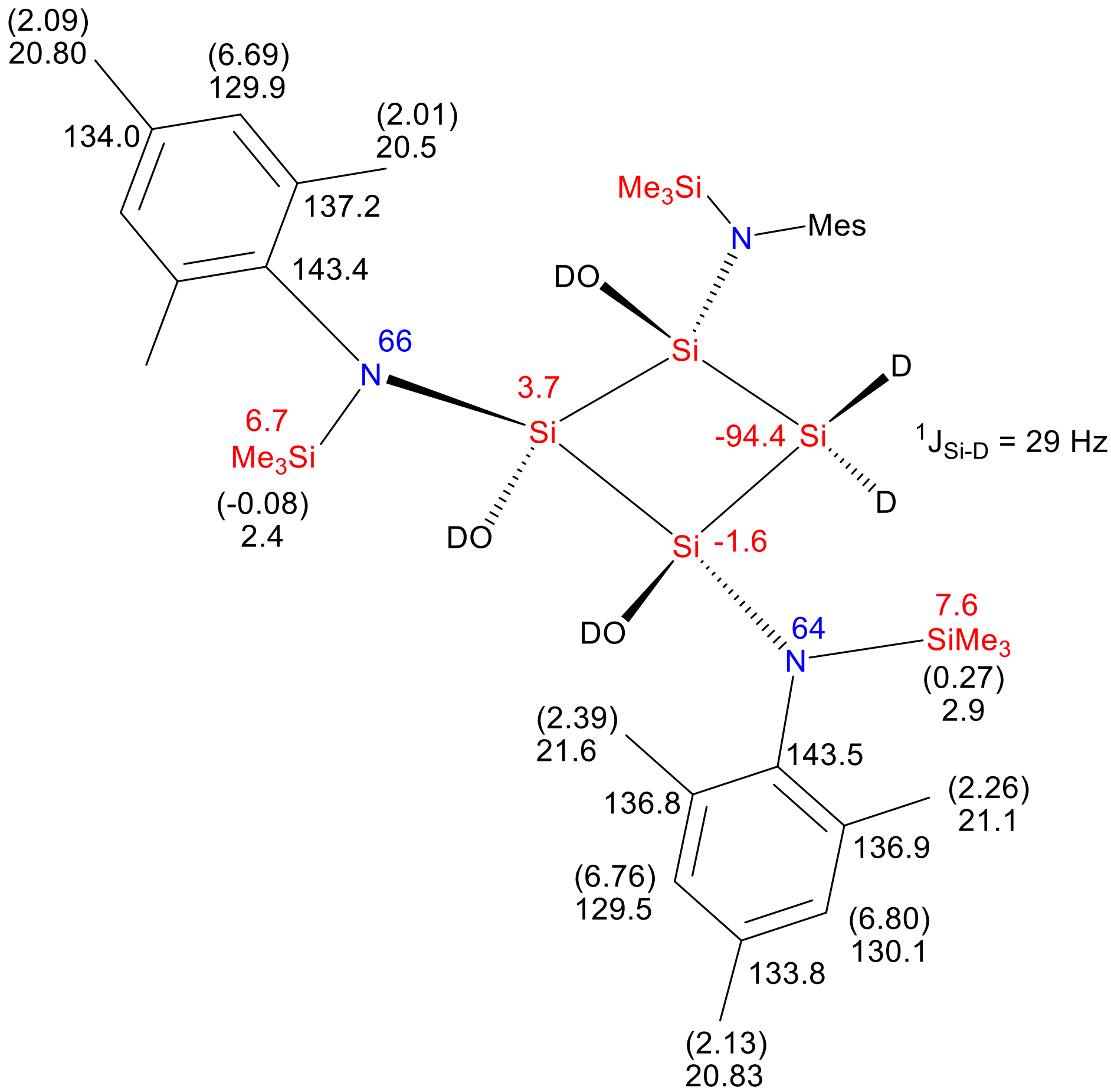

Figure S34. Assignment of chemical shifts to **2'** (in C_6D_6 at 300 K): Color code: ^{1}H black in brackets, ^{13}C black, ^{15}N blue, and ^{29}Si red.

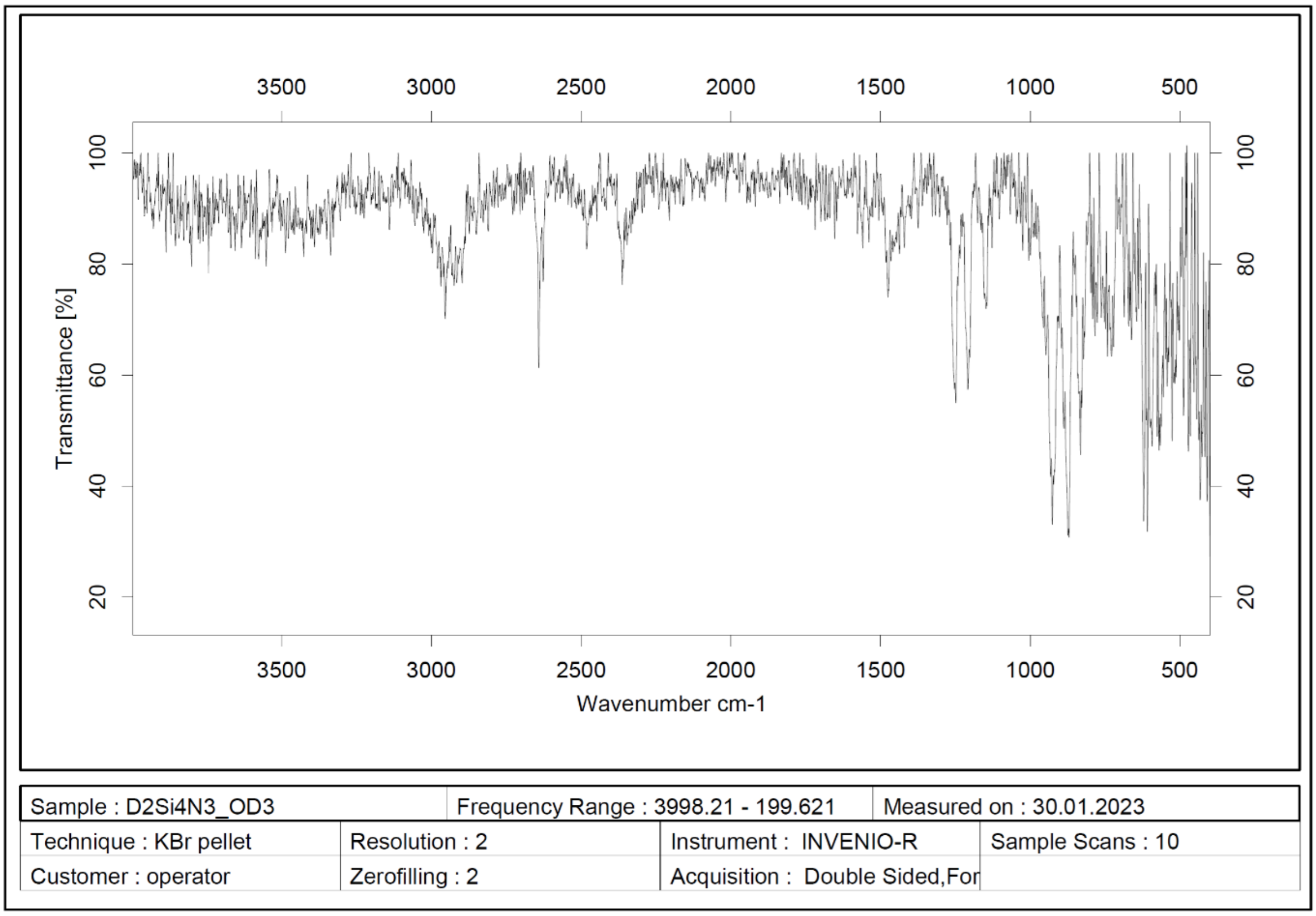

Figure S35. FT-IR spectrum of **2’** (KBr-pellet).

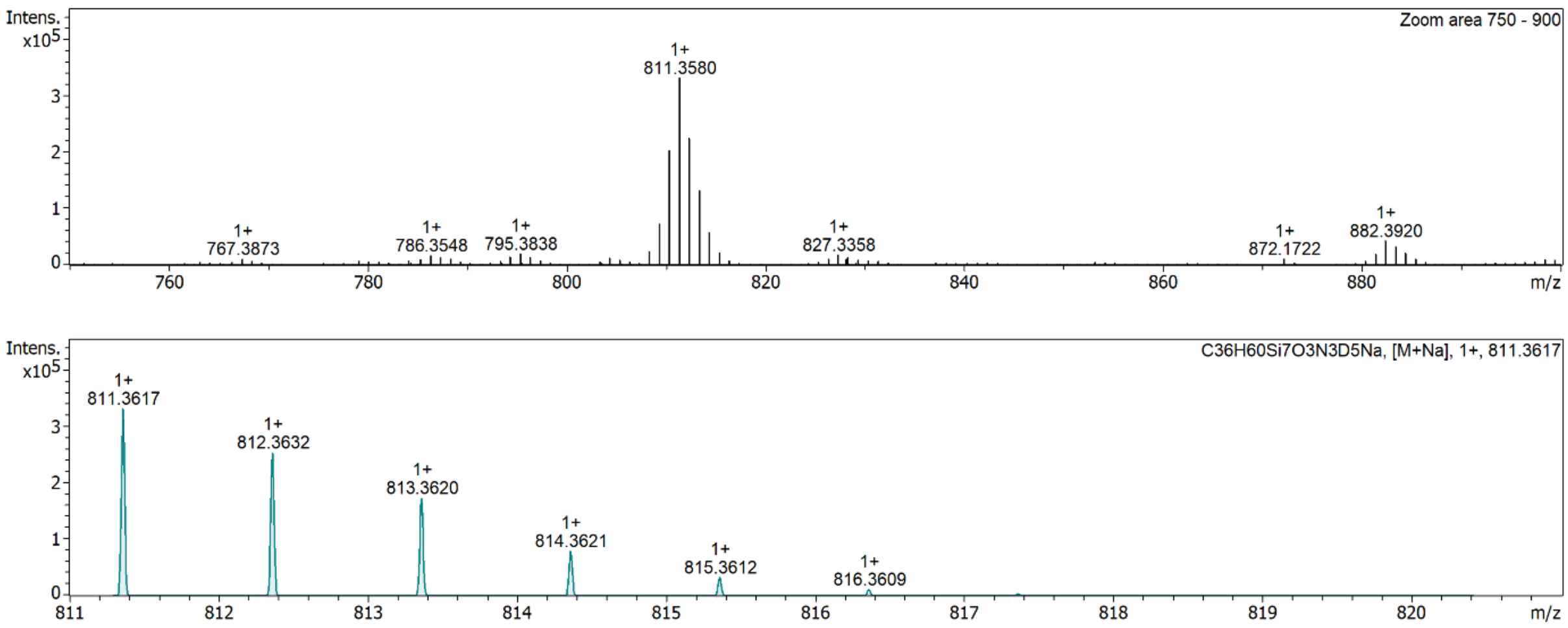

Figure S36. ESI-MS spectrum (positive mode) of **2’**.

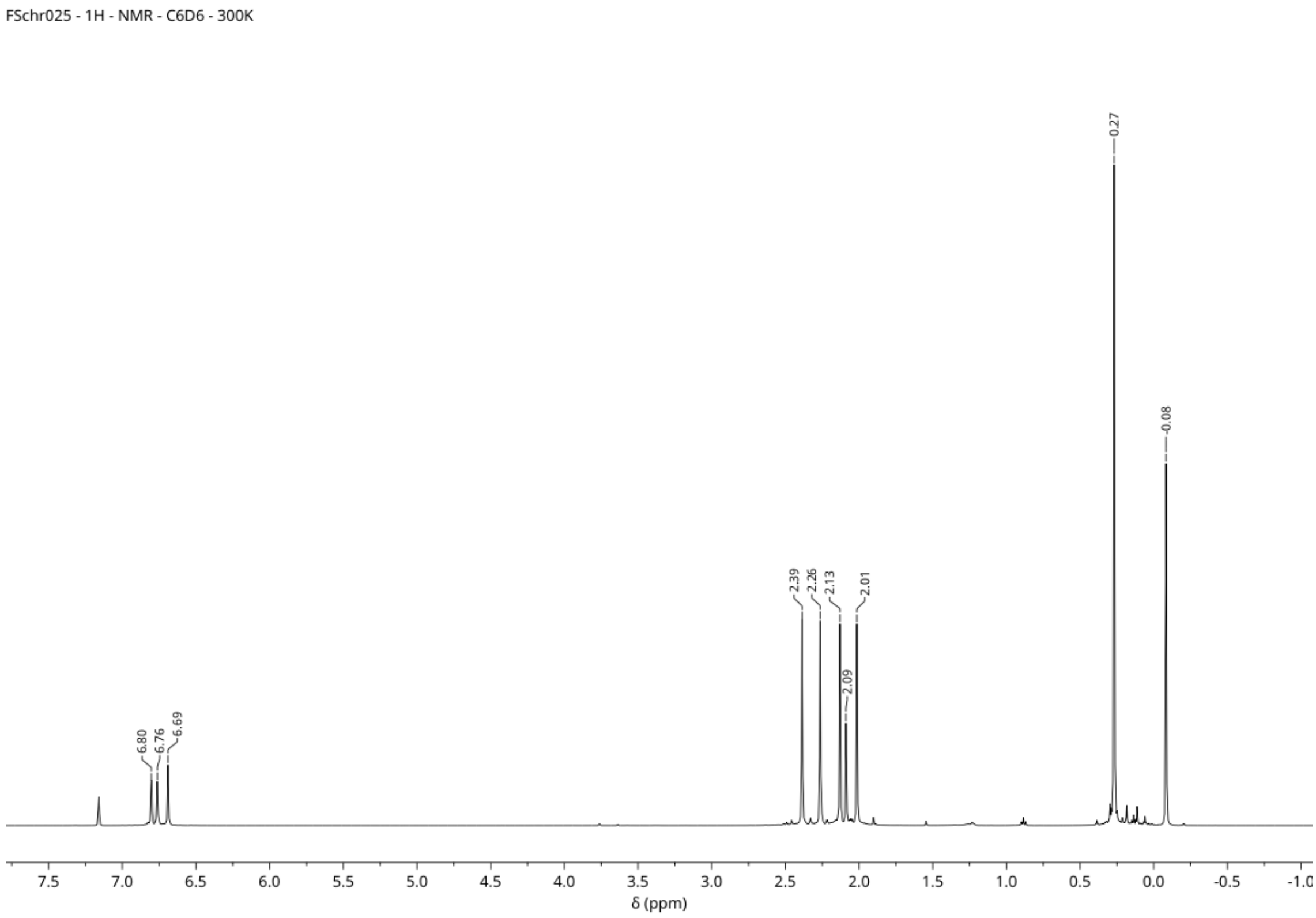

Figure S37. ^{1}H NMR spectrum of **2’** in C_6D_6 at 300 K and 500 MHz.

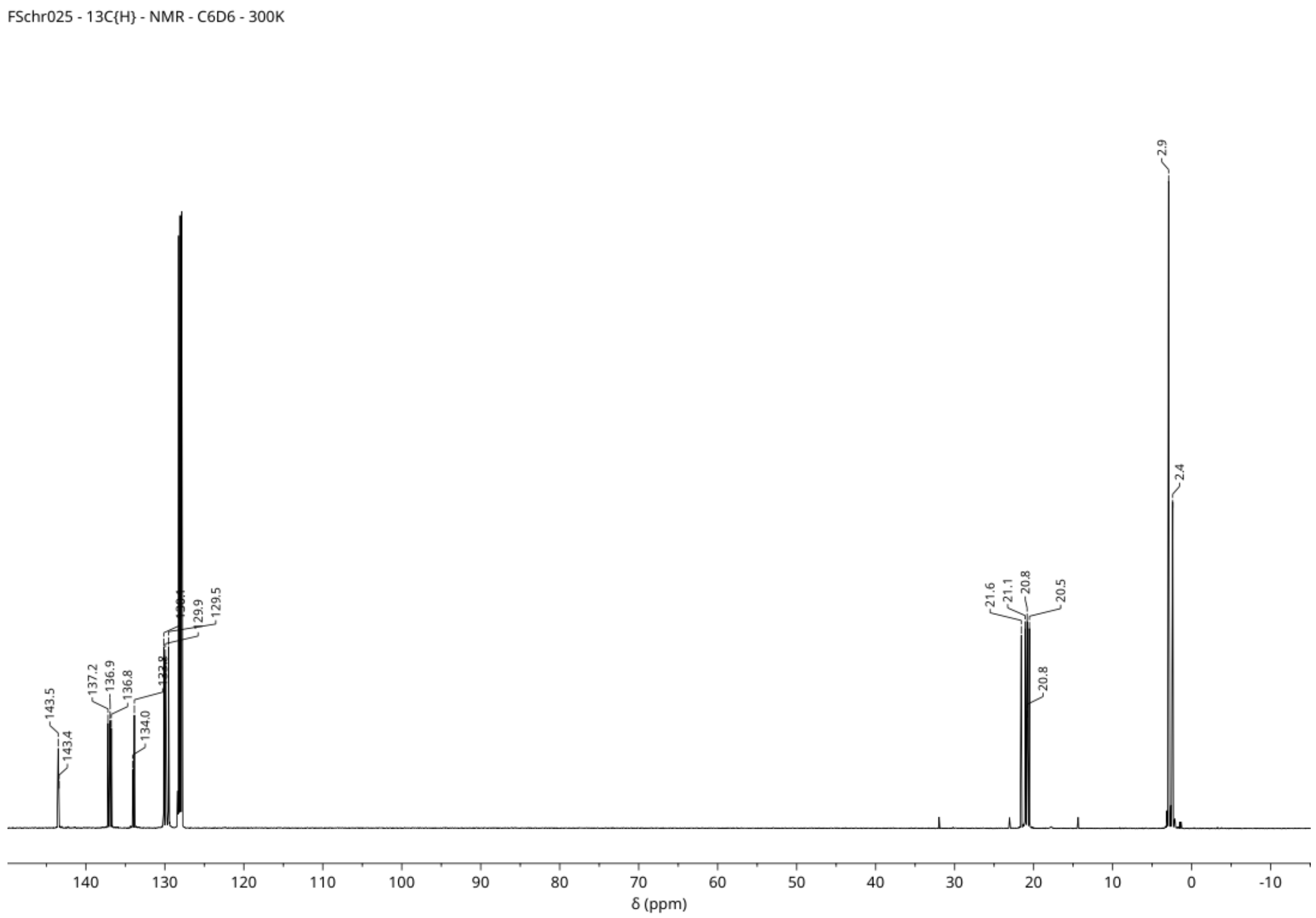

Figure S38. $^{13}C\{^{1}H\}$ NMR spectrum of **2’** in C_6D_6 at 300 K and 126 MHz.

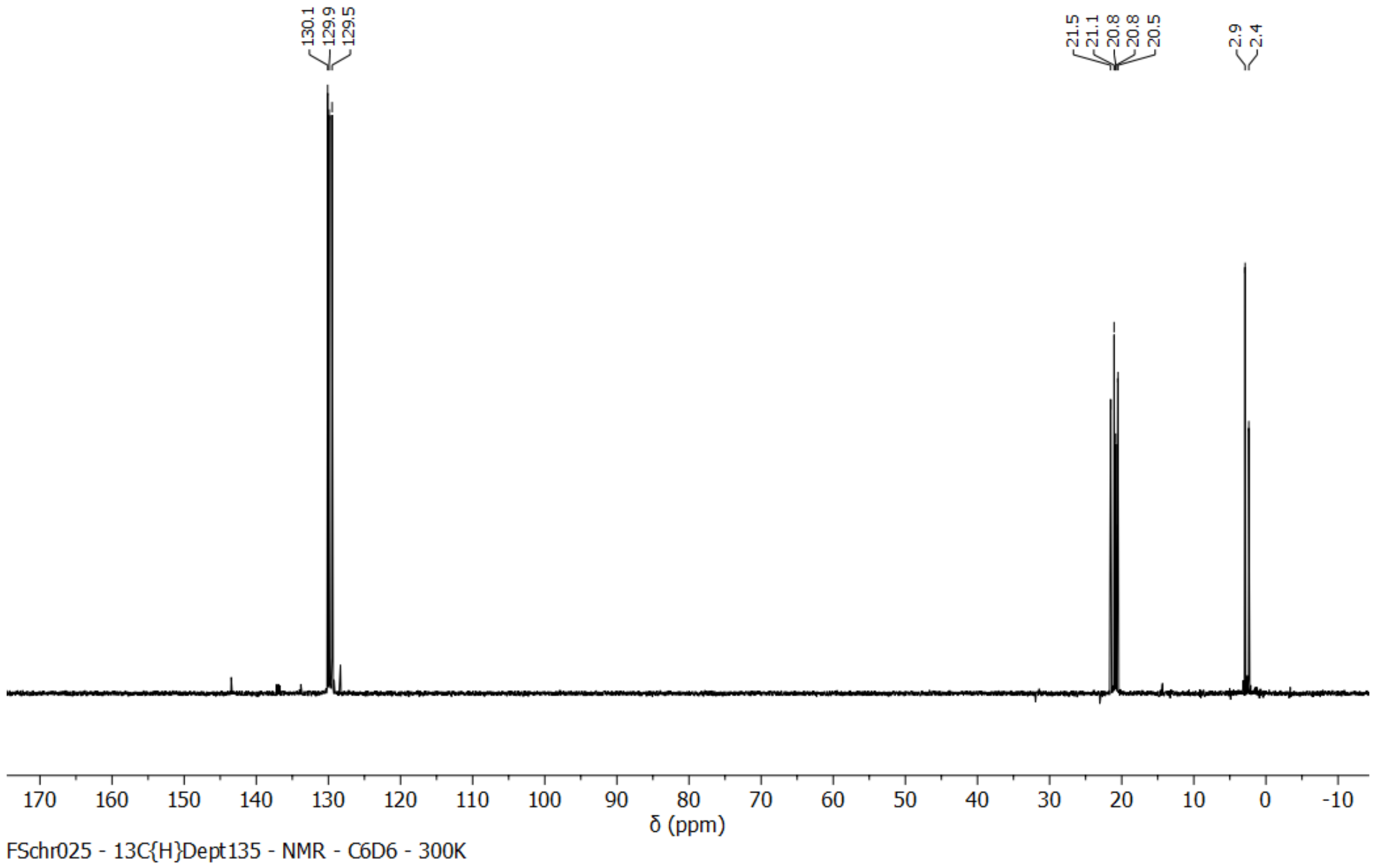

Figure S39. $^{13}C\{^{1}H\}$ DEPT-135 NMR spectrum of **2’** in C_6D_6 at 300 K and 126 MHz.

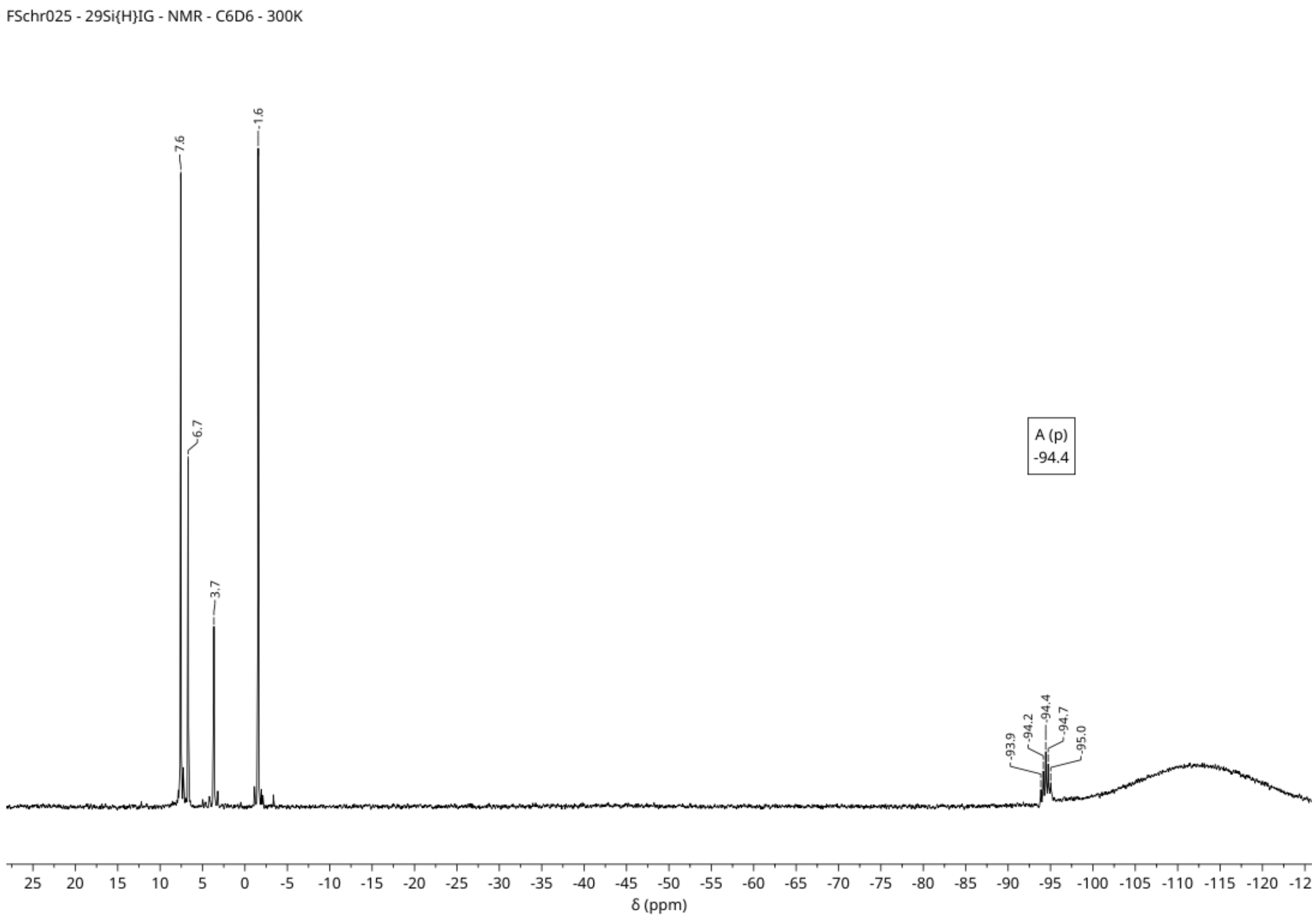

Figure S40. $^{29}Si\{^{1}H\}$ IG NMR spectrum of **2’** in C_6D_6 at 300 K and 99 MHz.

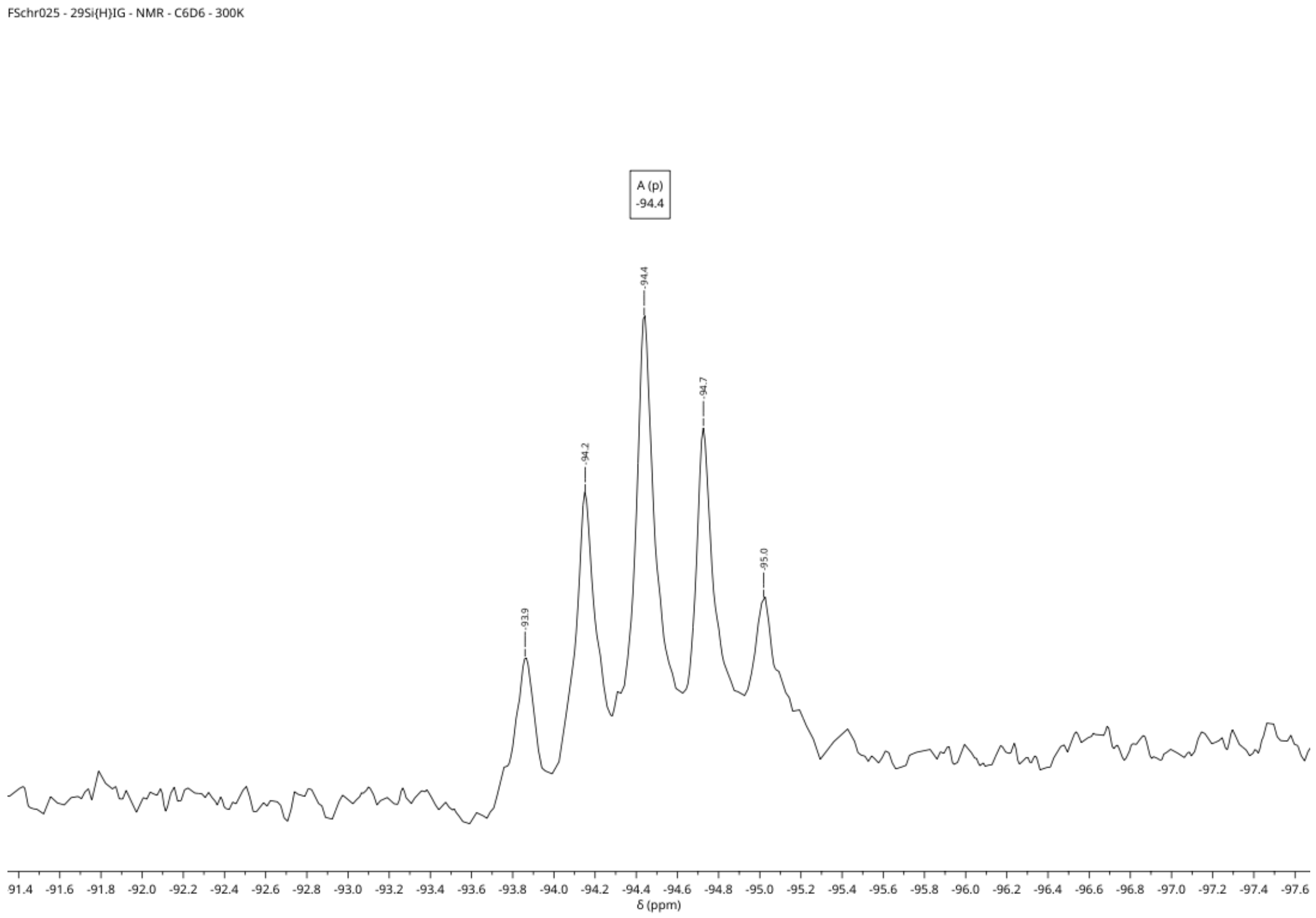

Figure S41. Zoom into the $^{29}Si\{^{1}H\}$IG NMR spectrum of **2’** in C_6D_6 at 300 K and 99 MHz. $^{1}J_{Si–D}$ = 29 Hz.

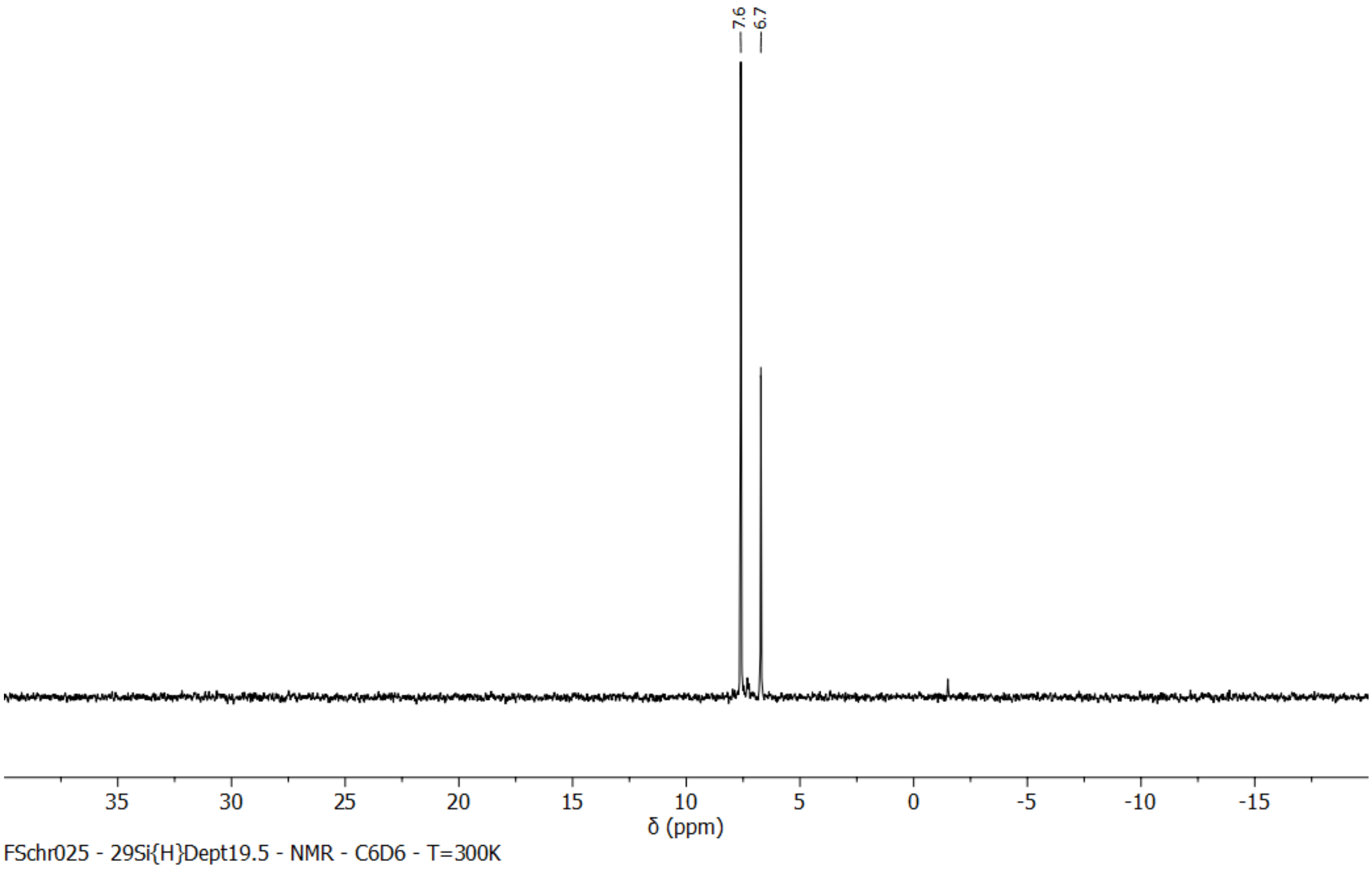

Figure S42. $^{29}Si\{^{1}H\}$ DEPT-19.5 NMR spectrum of **2’** in C_6D_6 at 300 K and 99 MHz.

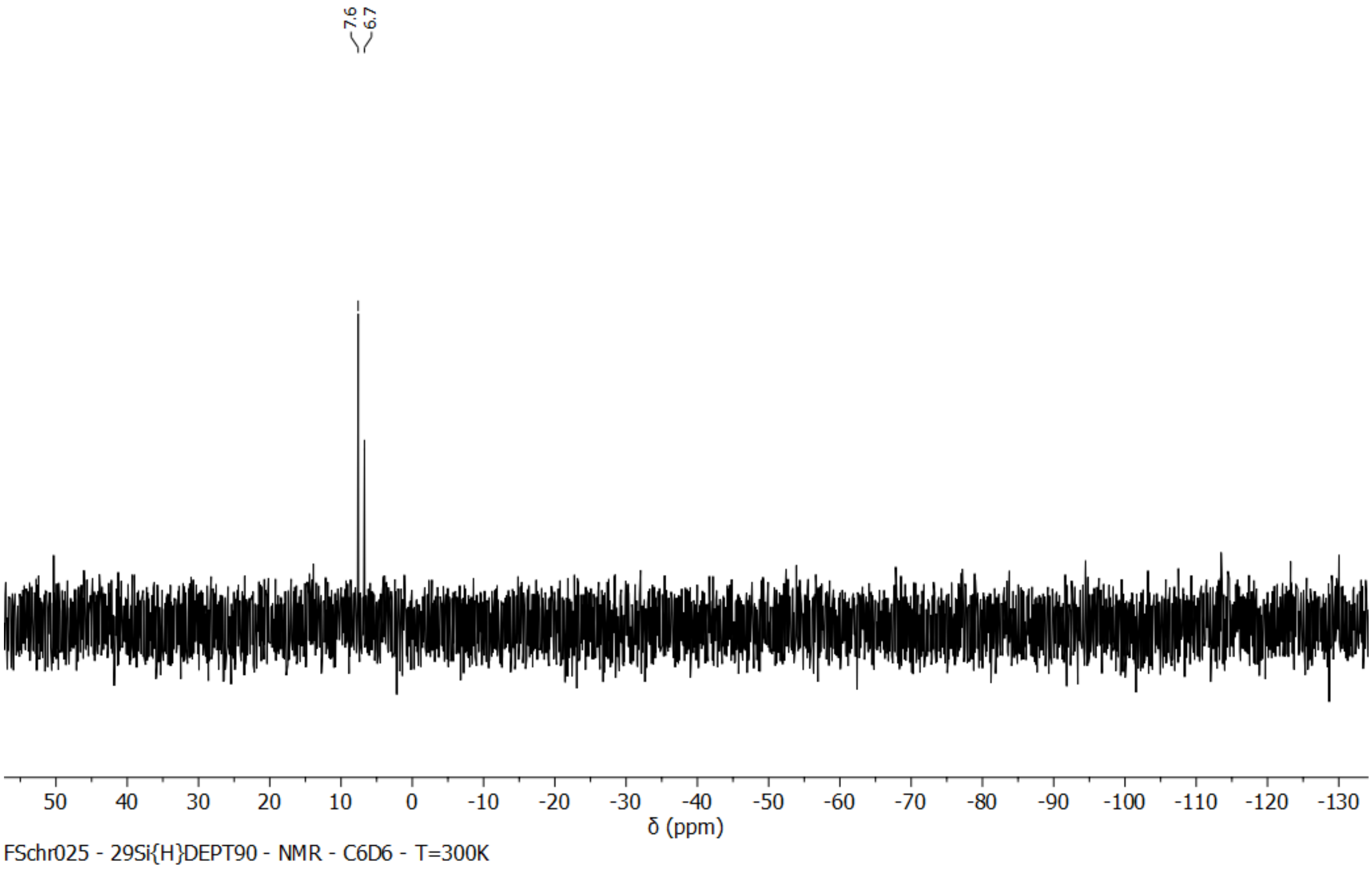

Figure S43. $^{29}Si\{^1H\}$ DEPT-90 NMR spectrum of **2’** in C_6D_6 at 300 K and 99 MHz.

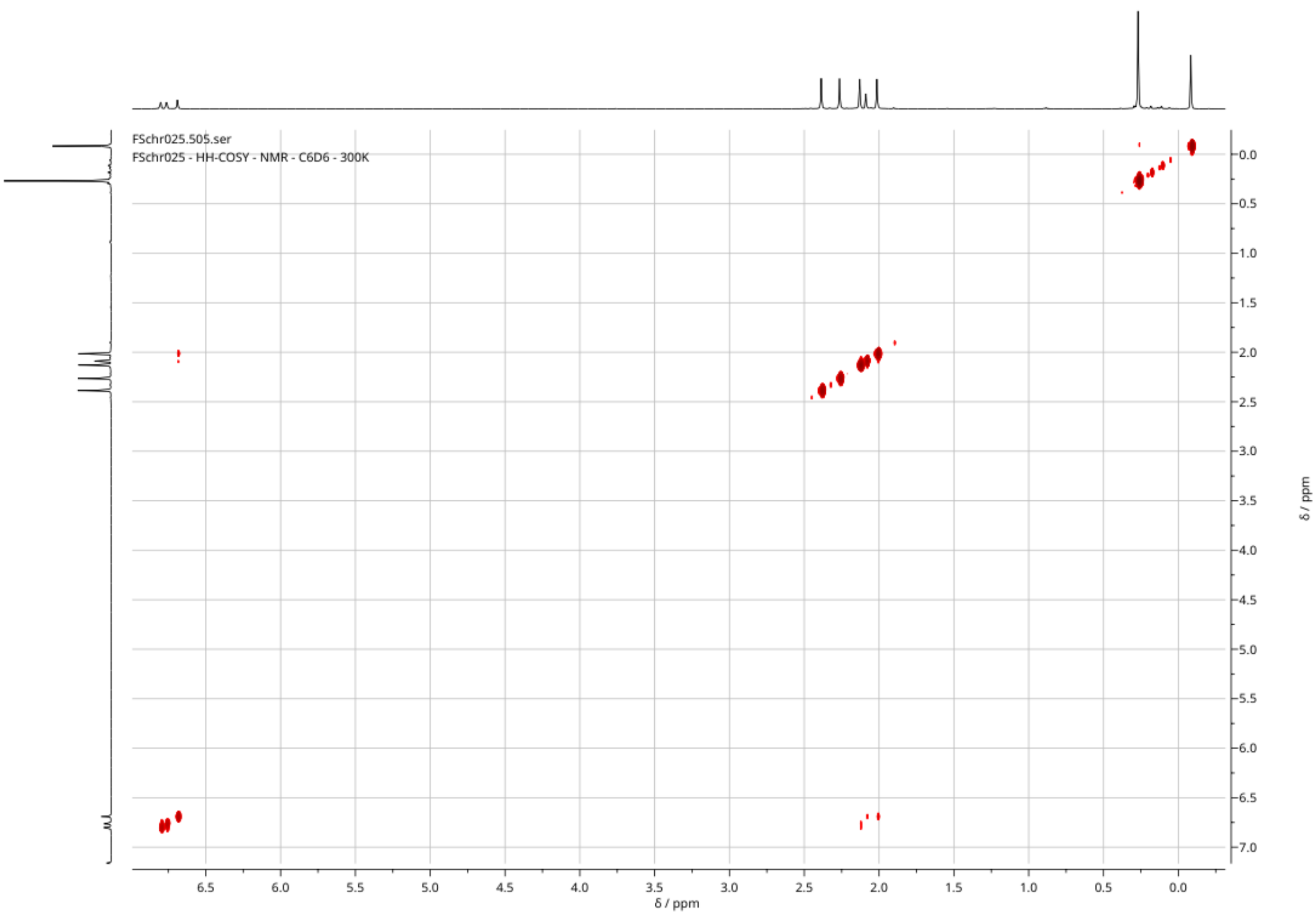

Figure S44. $^1H,^1H$ COSY NMR spectrum of **2’** in C_6D_6 at 300 K and 500 MHz.

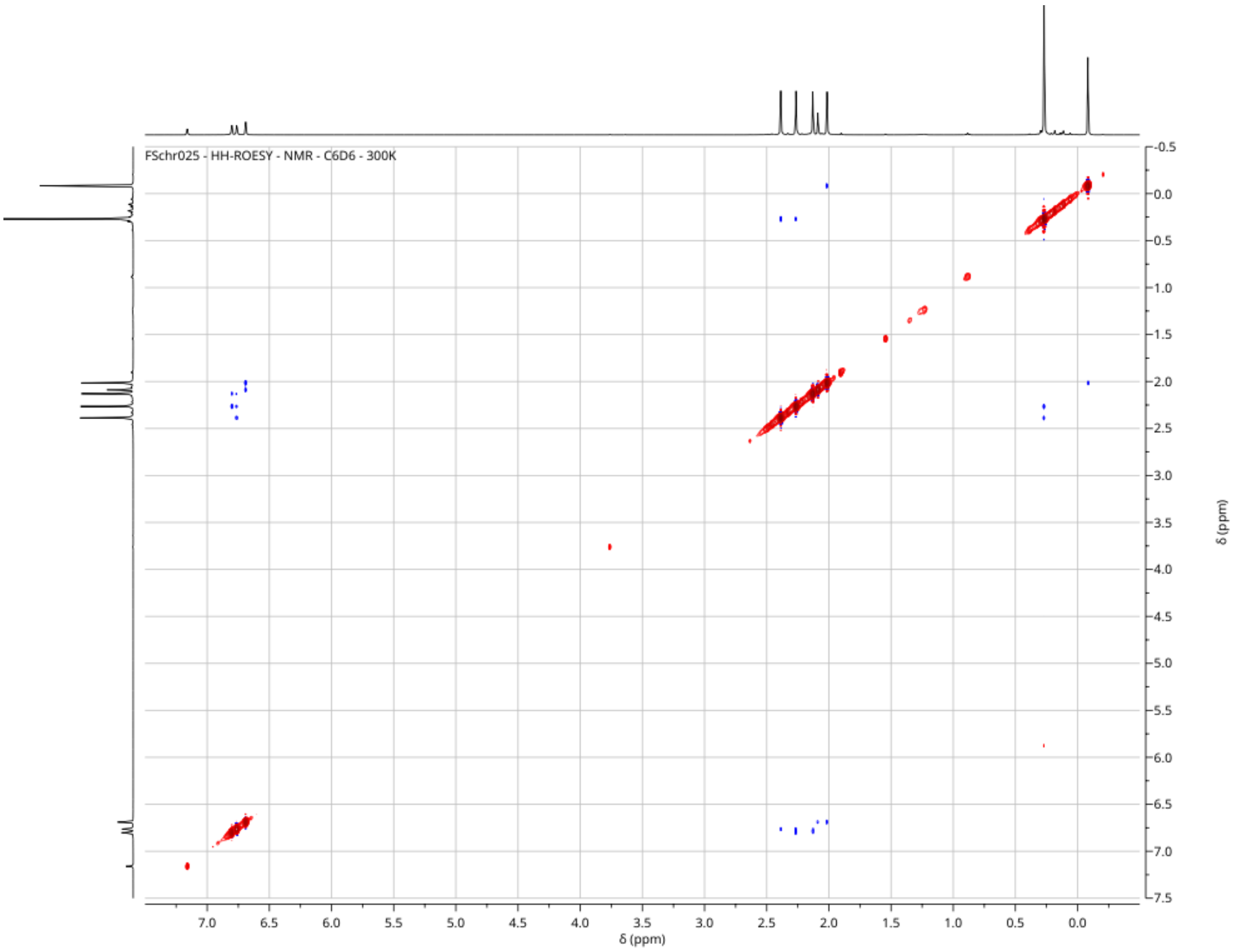

Figure S45. ^{1}H,^{1}H ROESY NMR spectrum of **2'** in C_6D_6 at 300 K and 500 MHz.

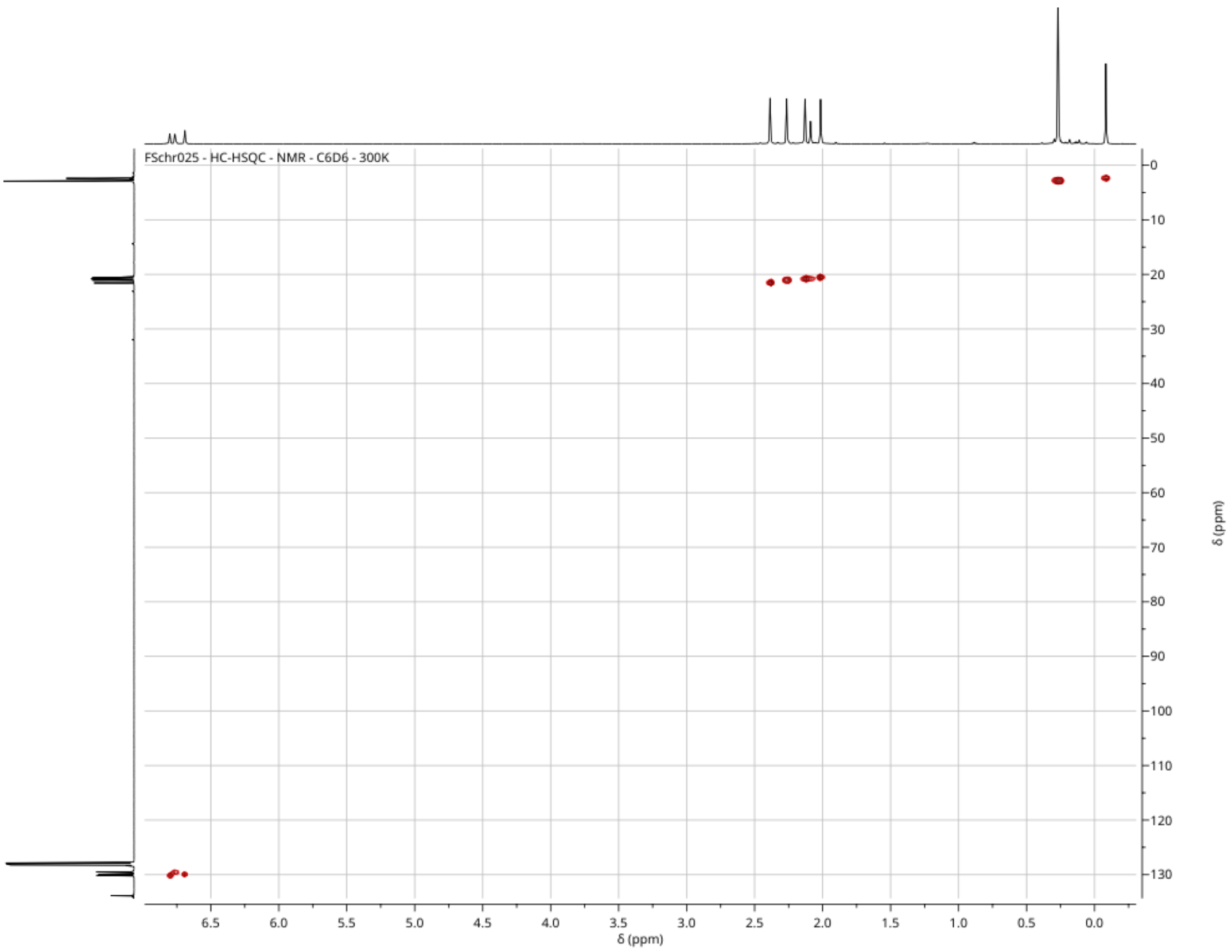

Figure S46. ^{1}H,^{13}C HSQC NMR spectrum of **2'** in C_6D_6 at 300 K and 500 MHz (^{1}H) and 126 MHz (^{13}C).

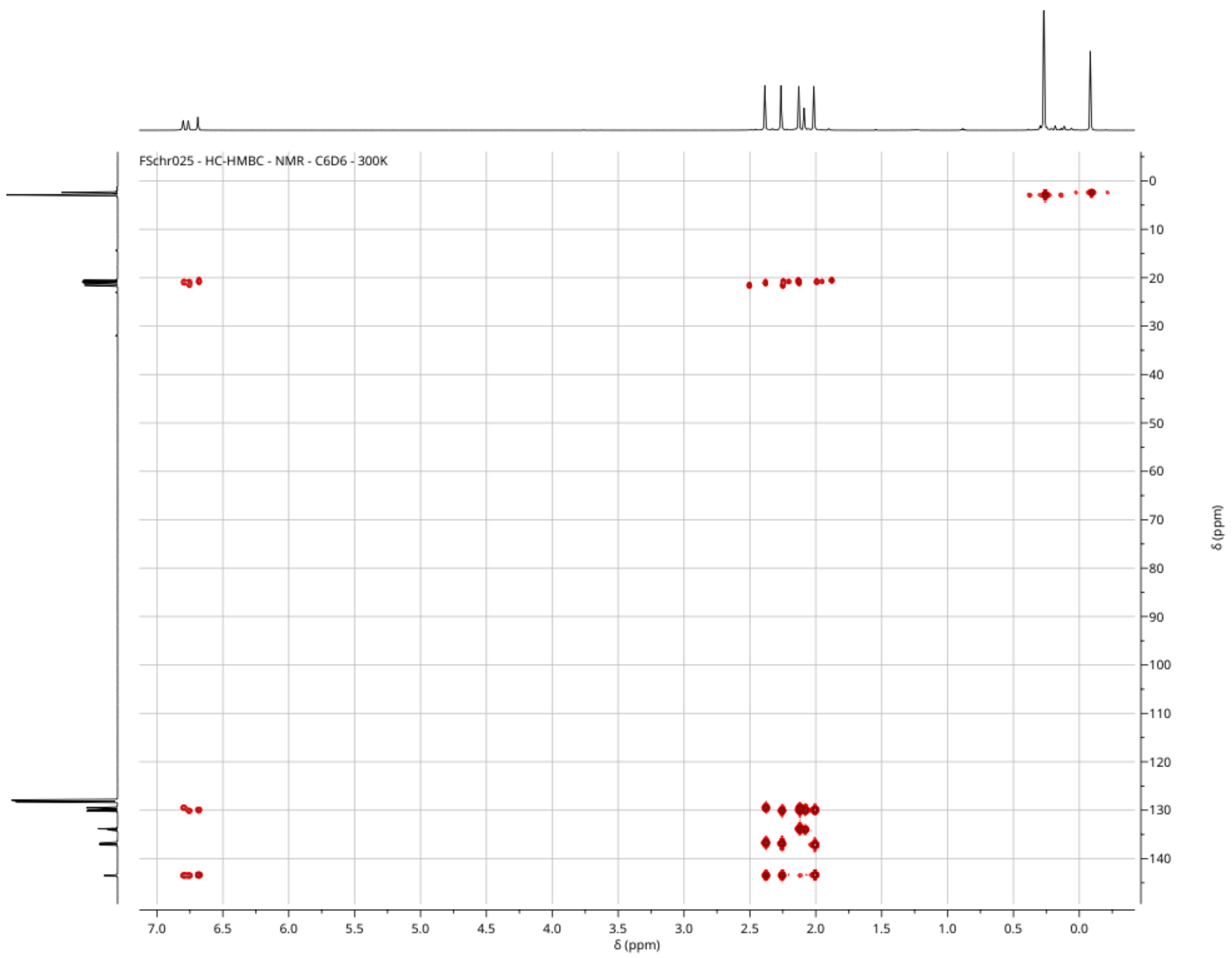

Figure S47. $^{1}H,^{13}C$ HMBC NMR spectrum of **2’** in C_6D_6 at 300 K and 500 MHz (^{1}H) and 126 MHz (^{13}C).

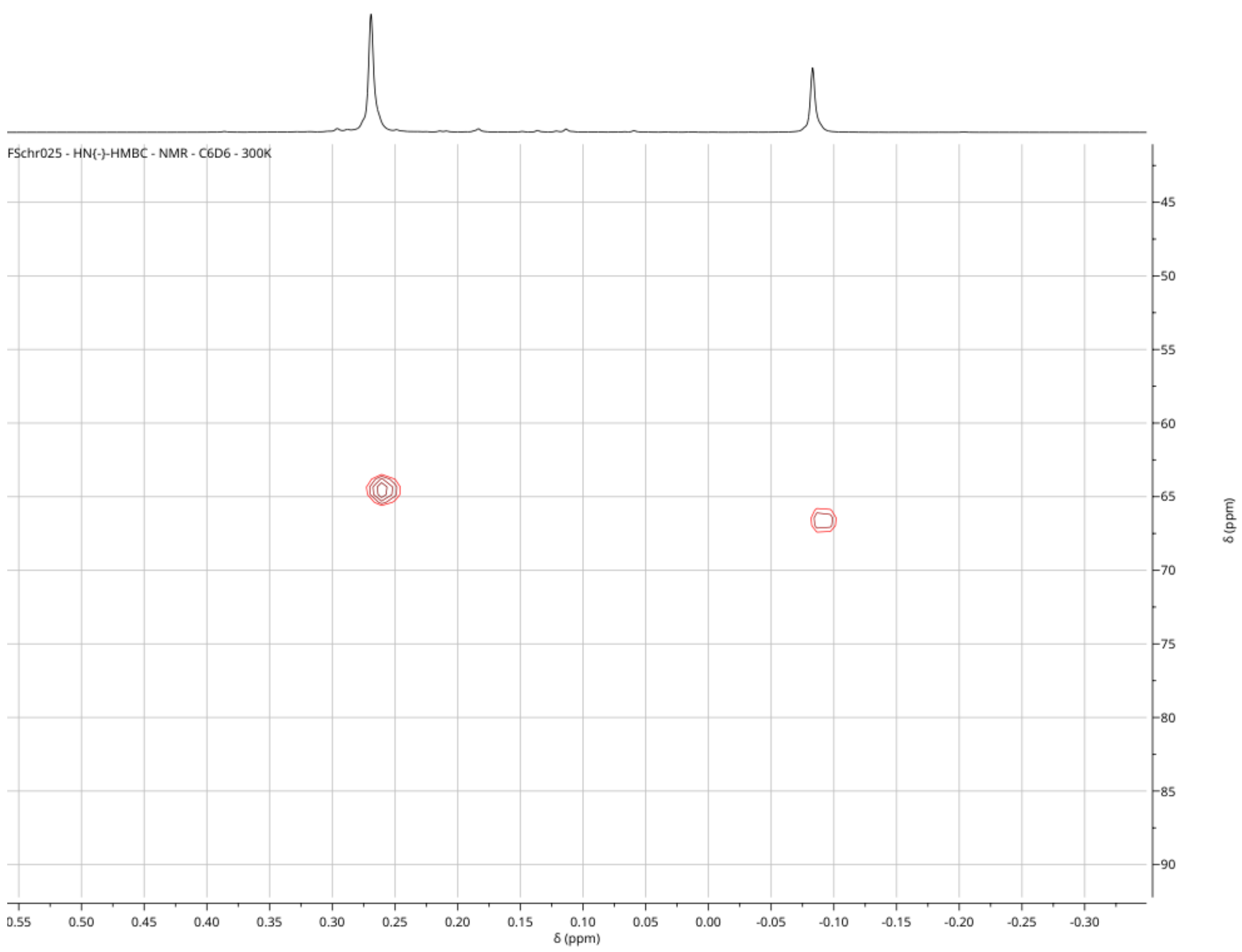

Figure S48. $^{1}H,^{15}N$ HMBC NMR spectrum of **2’** in C_6D_6 at 300 K and 500 MHz (^{1}H) and 50 MHz (^{15}N).

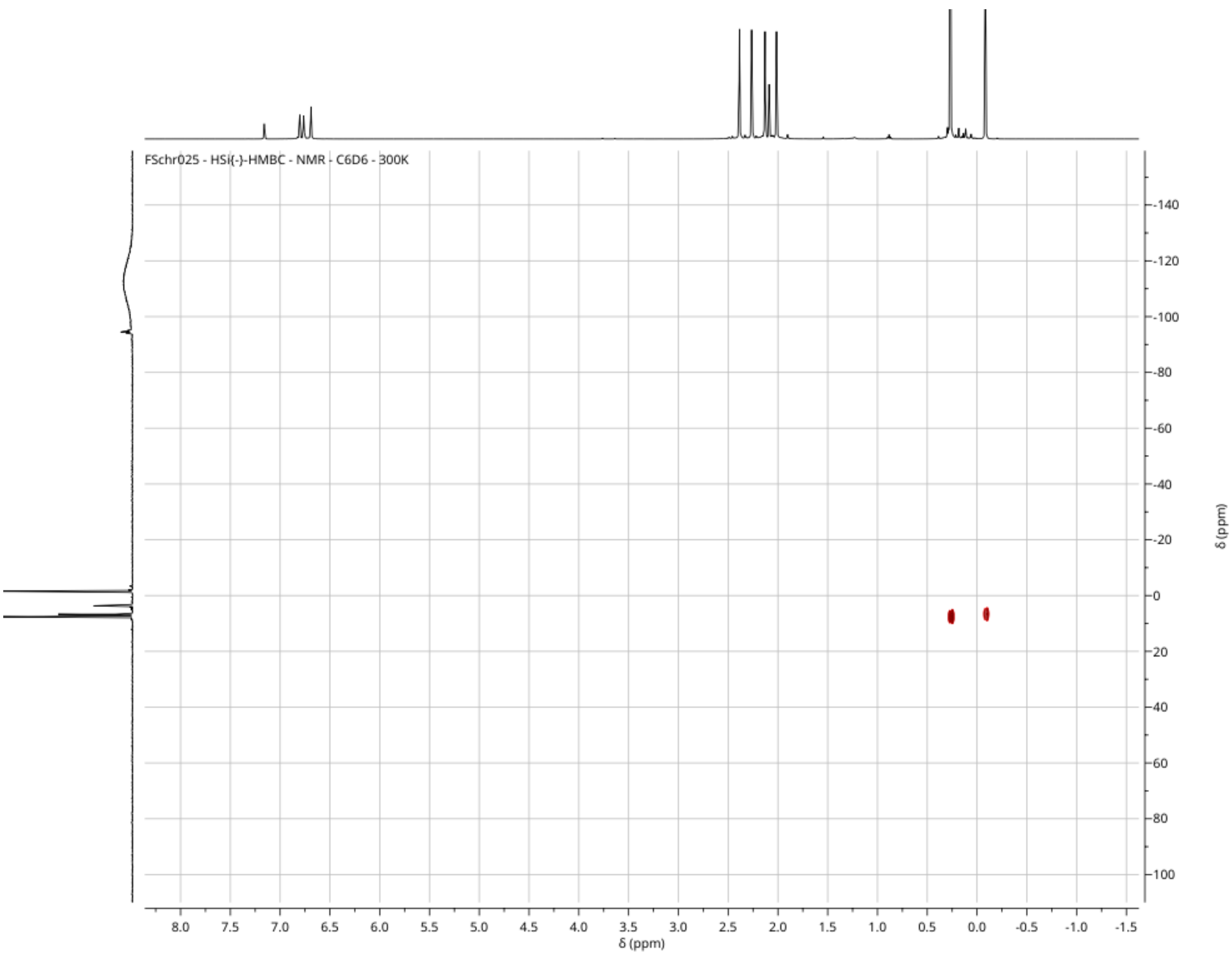

Figure S49. $^{1}H,^{29}Si$ HMBC NMR spectrum of **2’** in C_6D_6 at 300 K and 500 MHz (^{1}H) and 79 MHz (^{29}Si).

2.4 Comparison of IR data for 2 and 2’

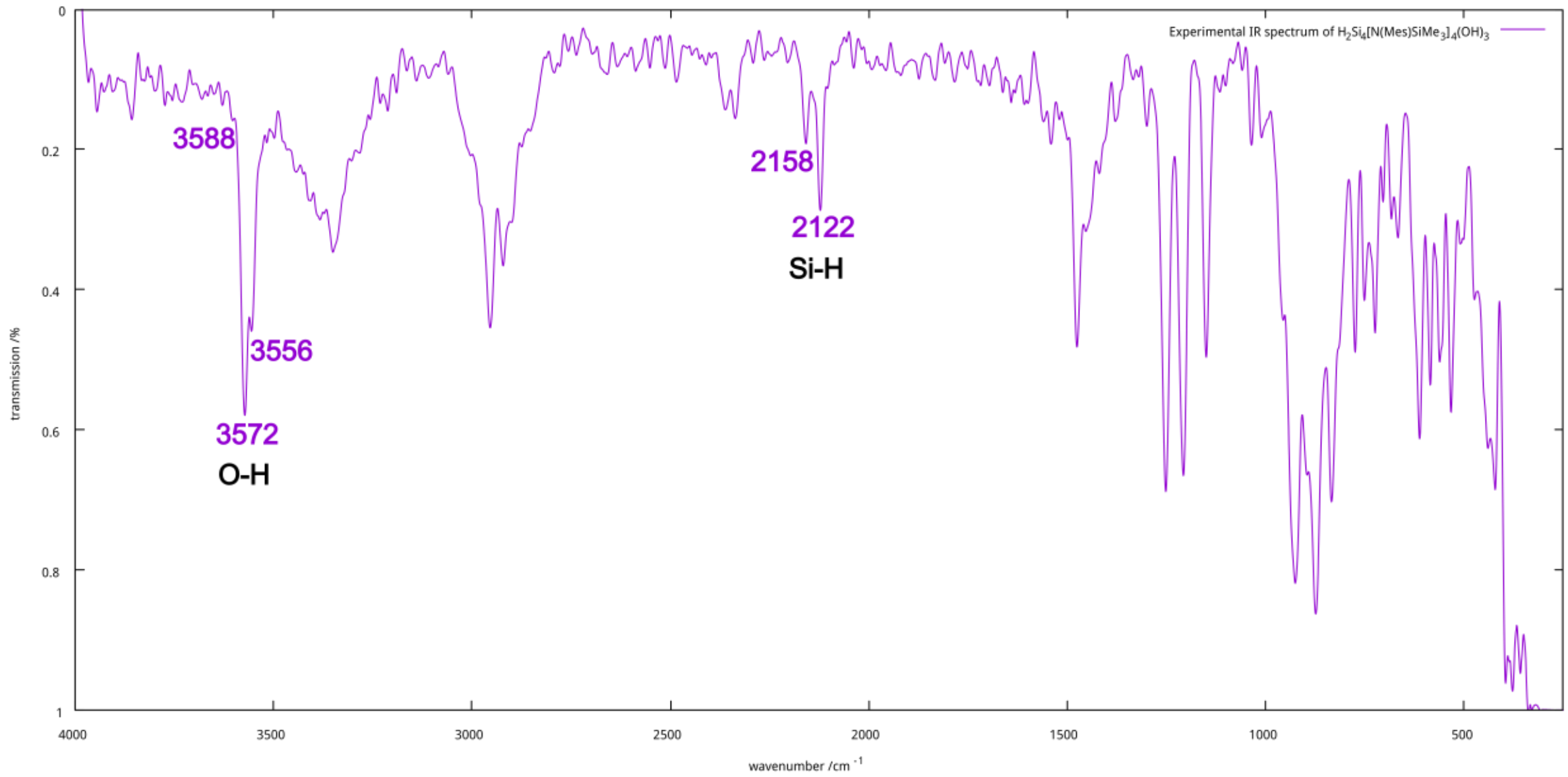

Figure S50. FT-IR spectrum of **2** (KBr-pellet) with Si–H and O–H bands labeled in the spectrum.

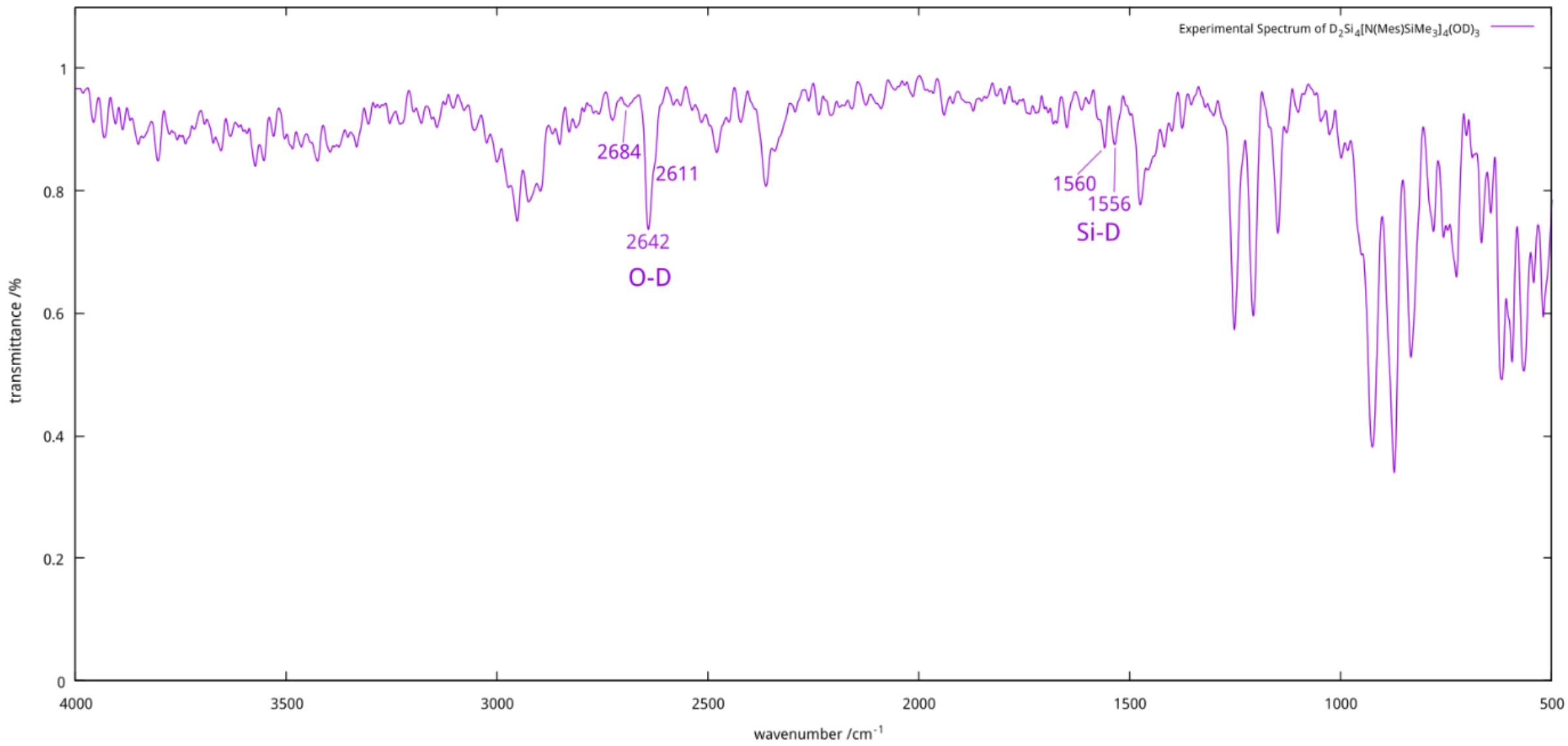

Figure S51. FT-IR spectrum of **2’** (KB0-pellet) with Si–D and O–D bands labeled in the spectrum.

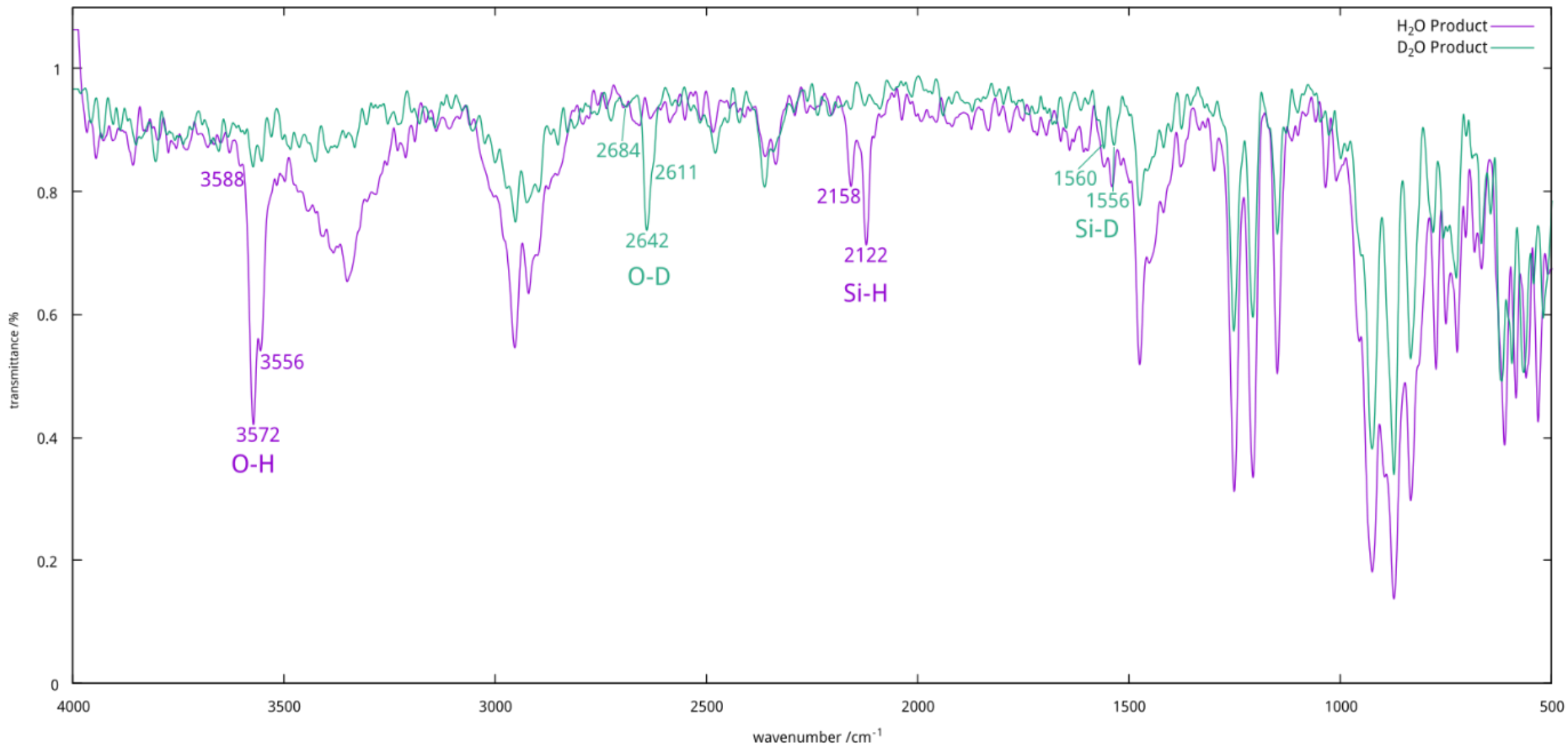

Figure S52. Overlay of FT-IR spectra of **2** (violet) and **2’** (green).

2.5 $(OMe)_2Si_8\{N(SiMe_3)Mes\}_6$ (3)

Methanol (0.11 mmol, 2.0 eq., 0.45 mL solution v/v 1:100 in THF) was slowly added dropwise to a cooled (−78 °C) solution of $Si_4\{N(SiMe_3)Mes\}_4$ **F** (52.8 mg, 0.06 mmol, 1.0 eq.) under stirring in THF (15 mL) using an acetone dry ice cooling bath. The solution was allowed to warm to room temperature and stirred for 16 h. All volatiles were removed *in vacuo* and the residue was washed with 3×3 mL DME. The resulting solid was dried *in vacuo*. $(OMe)_2Si_8\{N(SiMe_3)Mes\}_6$ (**3**) was obtained as a yellow solid.

Yield: 0.0229 g (0.02 mmol, 54.4 %).

Melting point: 251.9–252.9 °C.

^{1}H NMR (THF-d_8, 300 K, 500.2 MHz): δ (ppm) = 6.77 (s (overlapped), 2H, Mes–C$H_{arom.}$), 6.76 (s (overlapped), 2H, Mes–C$H_{arom.}$), 6.72 (s (overlapped), 2H, Mes–C$H_{arom.}$), 6.71 (s (overlapped), 2H, Mes–C$H_{arom.}$), 6.62 (s (overlapped), 2H, Mes–C$H_{arom.}$), 6.59 (s (overlapped), 2H, Mes–C$H_{arom.}$), 3.76 (s, 6H, OCH_3), 2.55 (s, 6H, *ortho*–Mes–CH_3), 2.50 (s, 6H, *ortho*–Mes–CH_3), 2.36 (s, 6H, *ortho*–Mes–CH_3), 2.19 (s, 6H, *para*–Mes–CH_3), 2.17 (s, 6H, *para*–Mes–CH_3), 2.17 (s, 6H, *para*–Mes–CH_3), 2.13 (s, 6H, *ortho*–Mes–CH_3), 1.89 (s, 6H, *ortho*–Mes–CH_3), 1.55 (s, 6H, *ortho*–Mes–CH_3), 0.06 (s, 18H, Si(CH_3)$_3$), –0.17 (s, 18H, Si(CH_3)$_3$), −0.18 (s, 18H, Si(CH_3)$_3$).

^{13}C{^{1}H} NMR (THF-d_8, 300 K, 125.8 MHz): δ (ppm) = 145.1 (s, 2C, Mes–C_{ipso}), 142.9 (s, 2C, Mes–C_{ipso}), 141.2 (s, 2C, Mes–C_{ipso}), 139.0 (s, 2C, Mes–C_{ortho}), 137.8 (s, 2C, Mes–C_{ortho}), 137.7 (s, 2C, Mes–C_{ortho}), 137.5 (s, 2C, Mes–C_{ortho}), 137.5 (s, 2C, Mes–C_{ortho}), 136.9 (s, 2C, Mes–C_{ortho}), 134.1 (s, 2C, Mes–C_{para}), 133.8 (s, 2C, Mes–C_{para}), 133.5 (s, 2C, Mes–C_{para}), 131.1 (s, 2C, Mes–C_{meta}), 131.0 (s, 2C, Mes–C_{meta}), 130.9 (s, 2C, Mes–C_{meta}), 130.6 (s, 2C, Mes–C_{meta}), 130.4 (s, 2C, Mes–C_{meta}), 129.4 (s, 2C, Mes–C_{meta}), 55.0 (s, 2C, O(CH$_3$)), 25.3 (s, 2C, *ortho*–Mes–CH$_3$), 24.3 (s, 2C, *ortho*–Mes–CH$_3$), 23.6 (s, 2C, *ortho*–Mes–CH$_3$), 23.5 (s, 2C, *ortho*–Mes–CH$_3$), 22.8 (s, 2C, *ortho*–Mes–CH$_3$), 22.5 (s, 2C, *ortho*–Mes–CH$_3$), 20.9 (s, 2C, *para*–Mes–CH$_3$), 20.9 (s, 2C, *para*–Mes–CH$_3$), 20.7 (s, 2C, *para*–Mes–CH$_3$), 4.9 (s, 6C, Si(CH$_3$)$_3$), 4.6 (s, 6C, Si(CH$_3$)$_3$), 4.1 (s, 6C, Si(CH$_3$)$_3$).

^{13}C{^{1}H} DEPT-135 NMR (THF-d_8, 300K, 125.8 MHz): δ (ppm) = 131.1 (s, 2C, Mes–C_{meta}), 131.0 (s, 2C, Mes–C_{meta}), 130.9 (s, 2C, Mes–C_{meta}), 130.6 (s, 2C, Mes–C_{meta}), 130.4 (s, 2C, Mes–C_{meta}), 129.4 (s, 2C, Mes–C_{meta}), 55.0 (s, 2C, O(CH$_3$)), 25.3 (s, 2C, *ortho*–Mes–CH$_3$), 24.3 (s, 2C, *ortho*–Mes–CH$_3$), 23.6 (s, 2C, *ortho*–Mes–CH$_3$), 23.5 (s, 2C, *ortho*–Mes–CH$_3$), 22.8 (s, 2C, *ortho*–Mes–CH$_3$), 22.5 (s, 2C, *ortho*–Mes–CH$_3$), 20.9 (s, 2C, *para*–Mes–CH$_3$), 20.9 (s, 2C, *para*–Mes–CH$_3$), 20.7 (s, 2C, *para*–Mes–CH$_3$), 4.9 (s, 6C, Si(CH$_3$)$_3$), 4.6 (s, 6C, Si(CH$_3$)$_3$), 4.1 (s, 6C, Si(CH$_3$)$_3$).

^{29}Si{^{1}H} IG NMR (THF-d_8, 300 K, 99.38 MHz): δ (ppm) = 16.3 (s, 2Si, *Si*), 9.1 (s, 2Si, (H$_3$C)$_3$*Si*), 7.9 (s, 2Si, (H$_3$C)$_3$*Si*), 5.5 (s, 2Si, (H$_3$C)$_3$*Si*), −1.4 (s, 2Si, *Si*), −58.5 (s, 2Si, *Si*), −136.2 (s, 2Si, *Si*).

^{29}Si{^{1}H} DEPT-19.5 NMR (THF-d_8, 300K, 99.38MHz): δ (ppm) = 9.1 (s, 2 Si, (H$_3$C)$_3$*Si*), 7.9 (s, 2 Si, (H$_3$C)$_3$*Si*), 5.5 (s, 2 Si, (H$_3$C)$_3$*Si*).

^{1}H,^{15}N HMBC NMR (THF-*d*$_8$, 300K, 500.2MHz, 50.70MHz): δ (ppm) = 54 (s, Mes–*N*–Si(CH$_3$)$_3$), 66 (s, Mes–*N*–Si(CH$_3$)$_3$), 70 (s, Mes–*N*–Si(CH$_3$)$_3$).

IR (KBr-pellet): $\tilde{\nu}$ (cm^{-1}) = 2976 w, 2955 w, 2795 s, 2714 s, 2341 vs, 1937 s, 1830 s, 1715 s, 1586 s, 1518 s, 1472 w, 1435 m, 1403 s, 1374 s, 1362 s, 1312 s, 1251 vw, 1213 w, 1199 vw, 1145 w, 1124 w, 1084 s, 1066 s, 1033 s, 961 m, 908 vw, 873 vw, 820 vw, 789 m, 759 w, 724 vw, 682 w, 654 m, 632 w, 589 vw.

UV-vis (*c* = 0.079 mmol/L and *c* = 0.016 mmol/L in THF): λ_{max} = 315 nm, ε = 9002 L·mol^{-1}·cm^{-1}.

ESI-MS (positive mode, solution in THF): *m*/*z* = [M]$^+$ 1522.6710 (1522.6707 calc., Δ = 0.23 ppm).

CHN for $C_{74}H_{126}N_6O_2Si_{14}$: calc.: C 58.28 %, H 8.33 %, N 5.51 %; found: C 57.45 %, H 8.15 %, N 5.17 %.

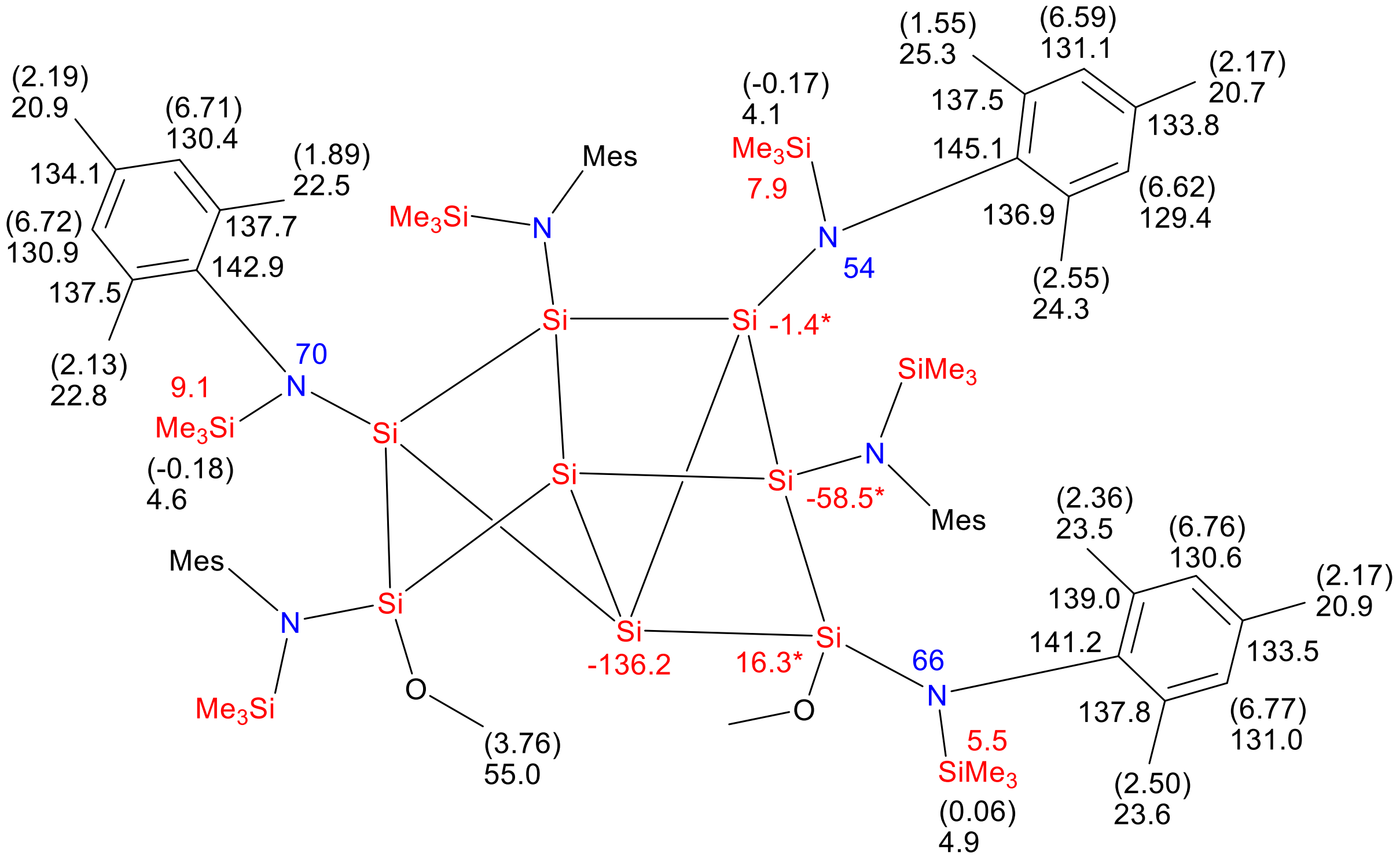

Figure S53. Assignment of chemical shifts to **3** (in THF-d_8 at 300 K). Color code: ^{1}H black in brackets, ^{13}C black, ^{15}N blue, and ^{29}Si red.

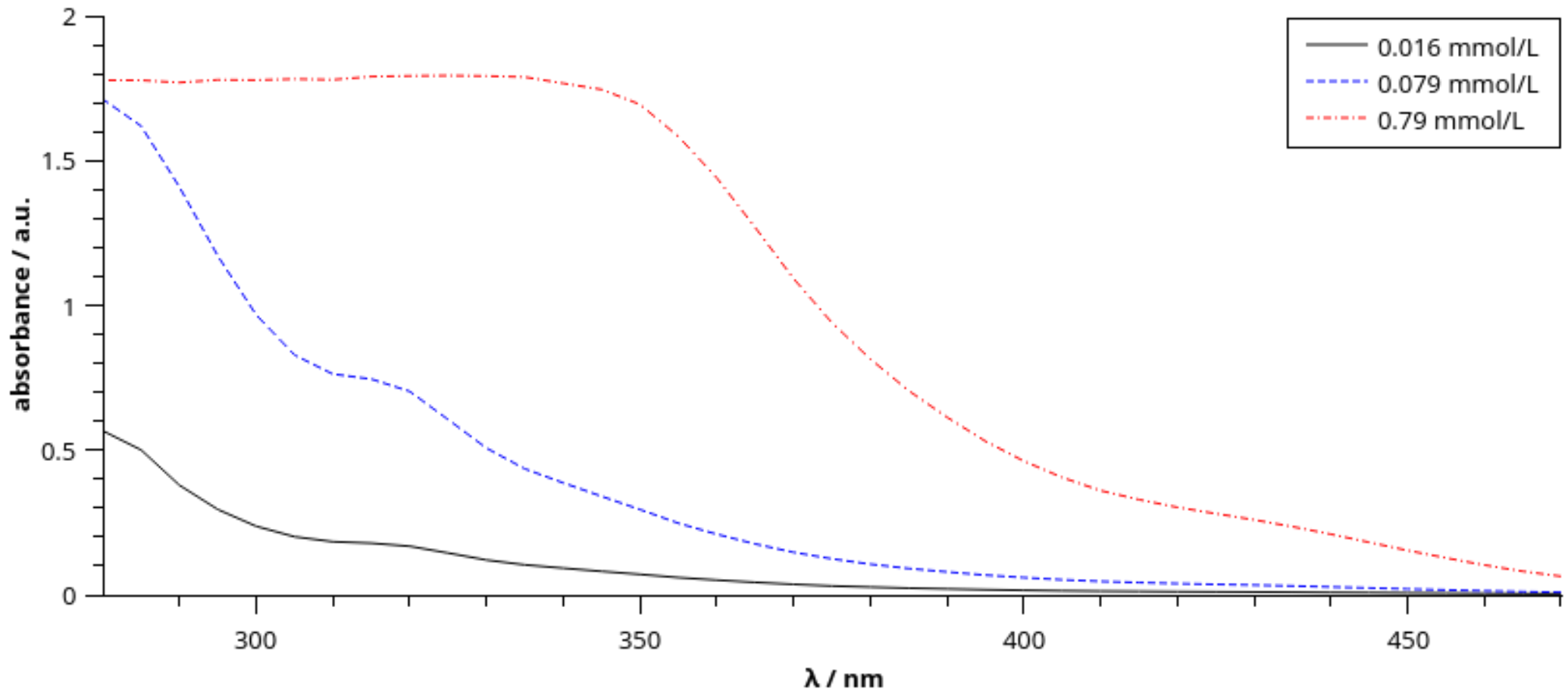

Figure S54. UV-vis spectra of **3** in THF.

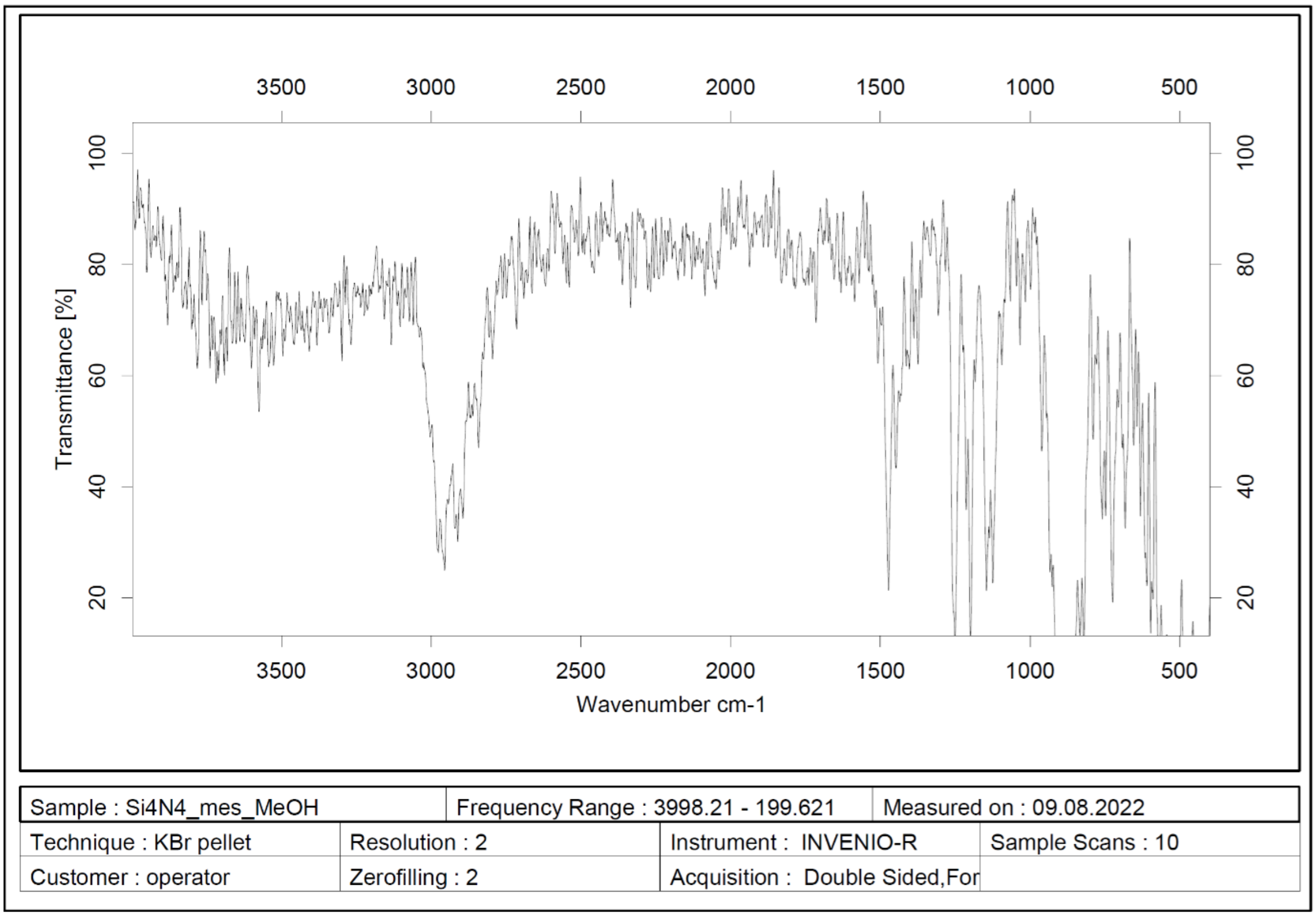

Figure S55. FT-IR spectrum of **3** (KBr-pellet).

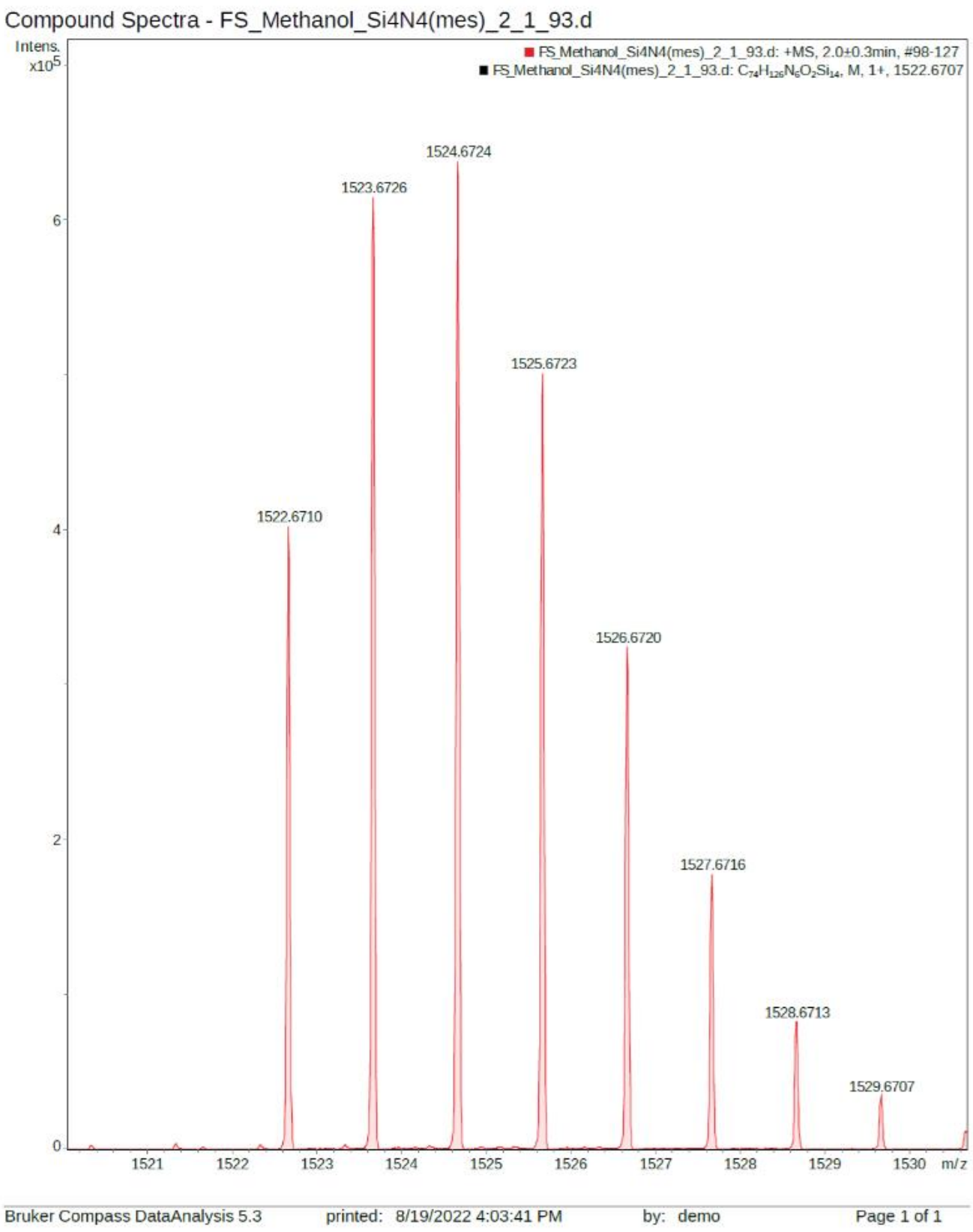

Figure S56. ESI-MS spectrum (positive mode) of **3**.

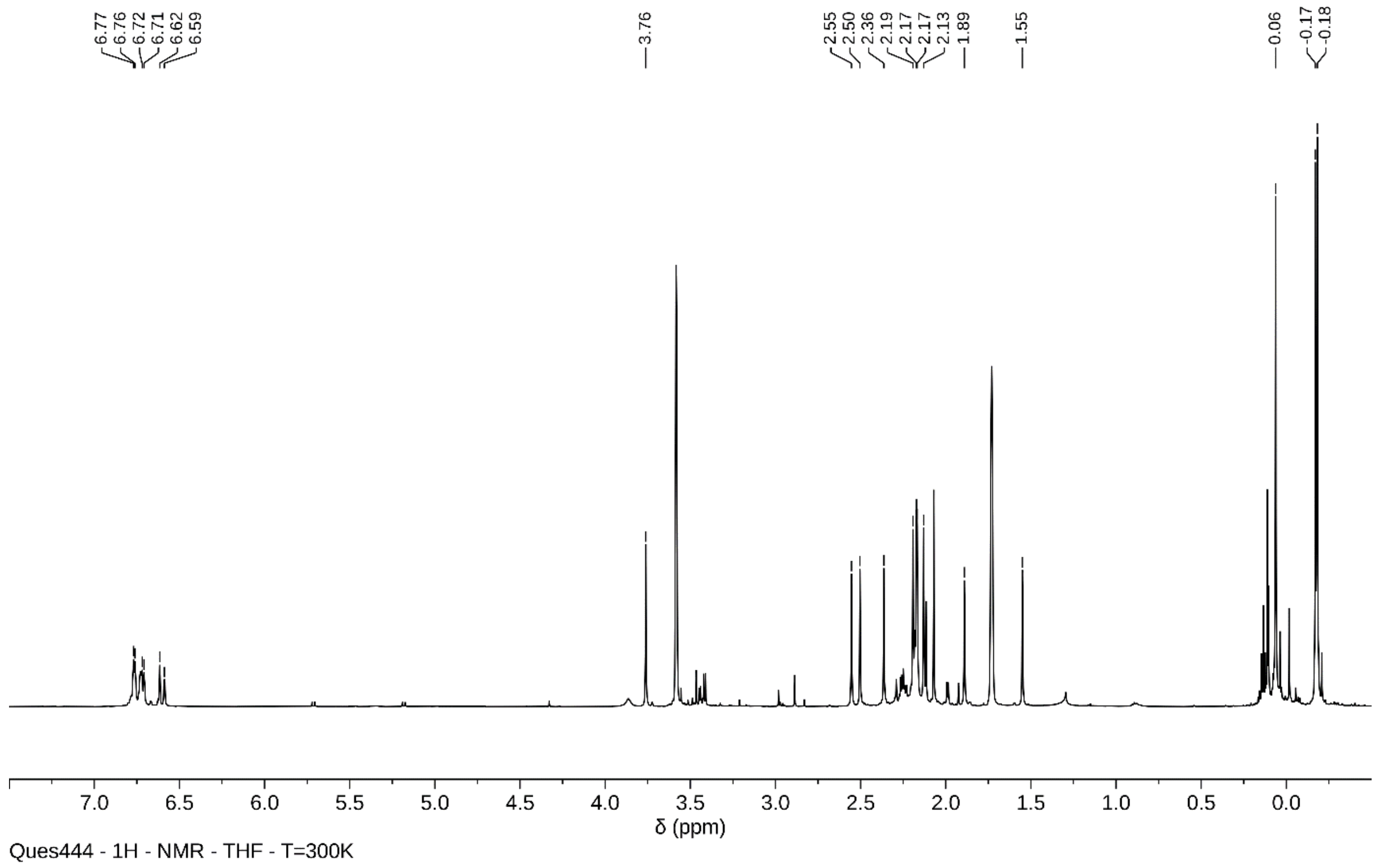

Figure S57. ^{1}H NMR spectrum of **3** in THF-d_8 at 300 K and 500 MHz.

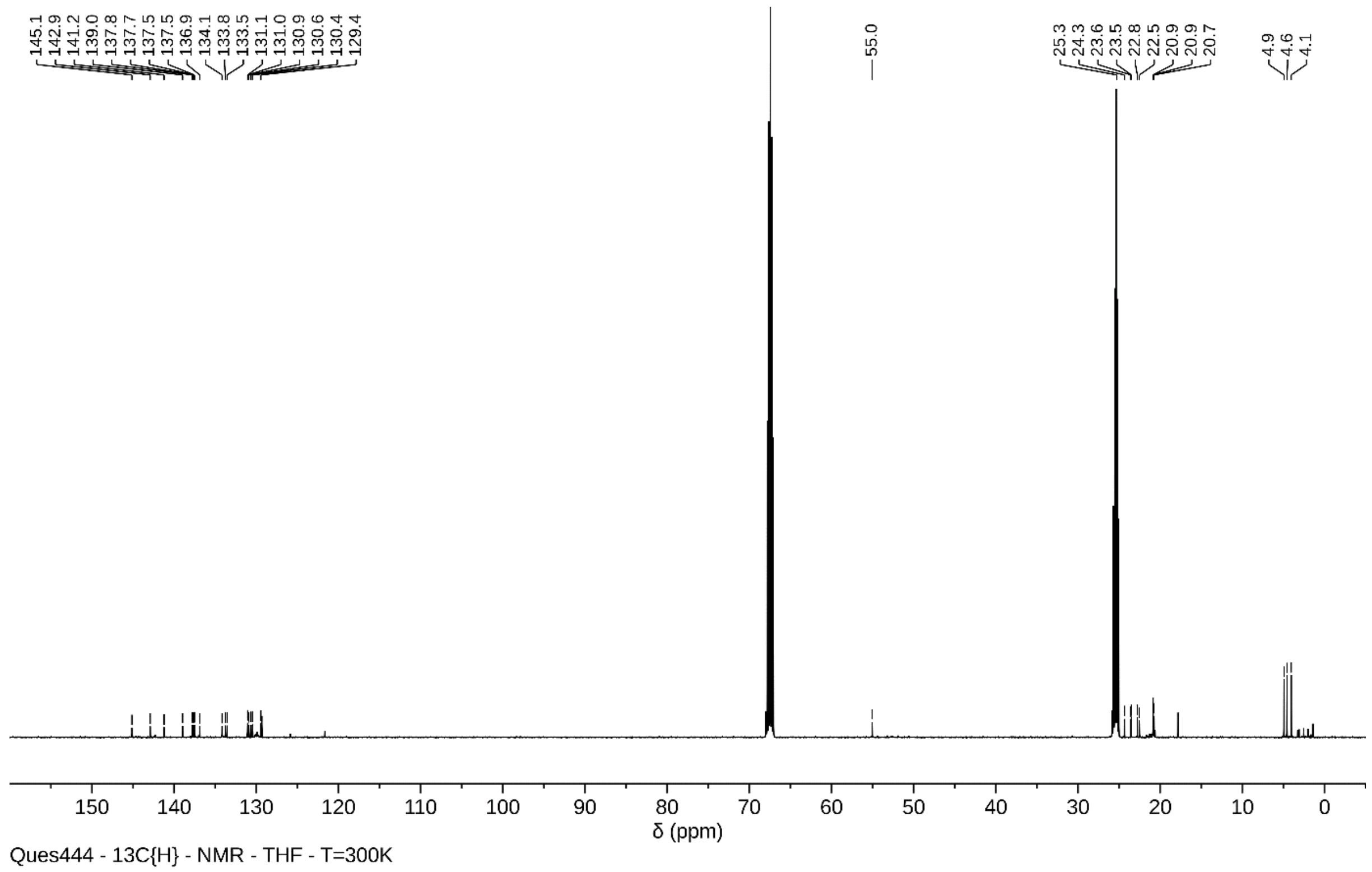

Figure S58. $^{13}C\{^{1}H\}$ NMR spectrum of **3** in THF-d_8 at 300 K and 126 MHz.

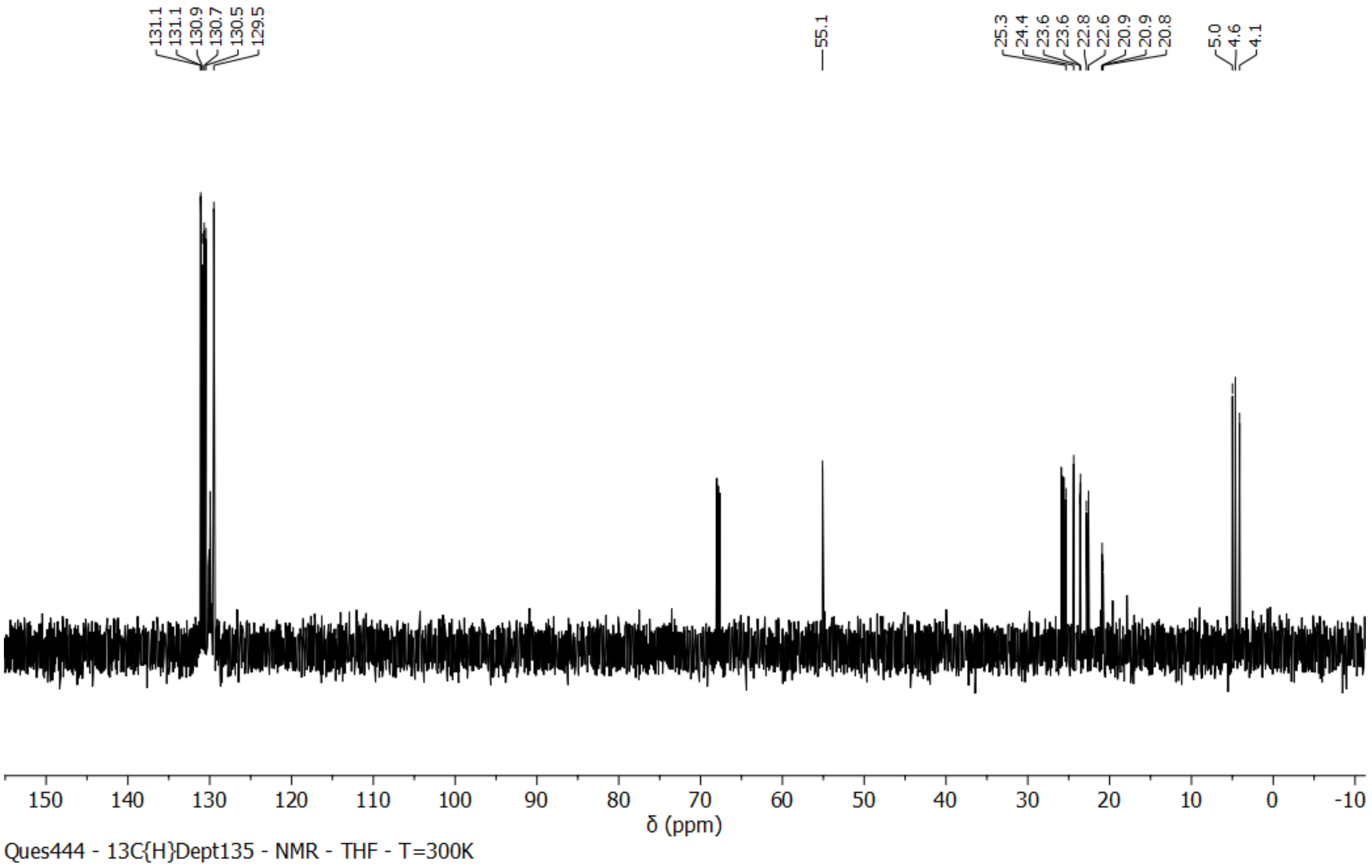

Figure S59. $^{13}C\{^{1}H\}$ DEPT-135 NMR spectrum of **3** in THF-d_8 at 300 K and 126 MHz.

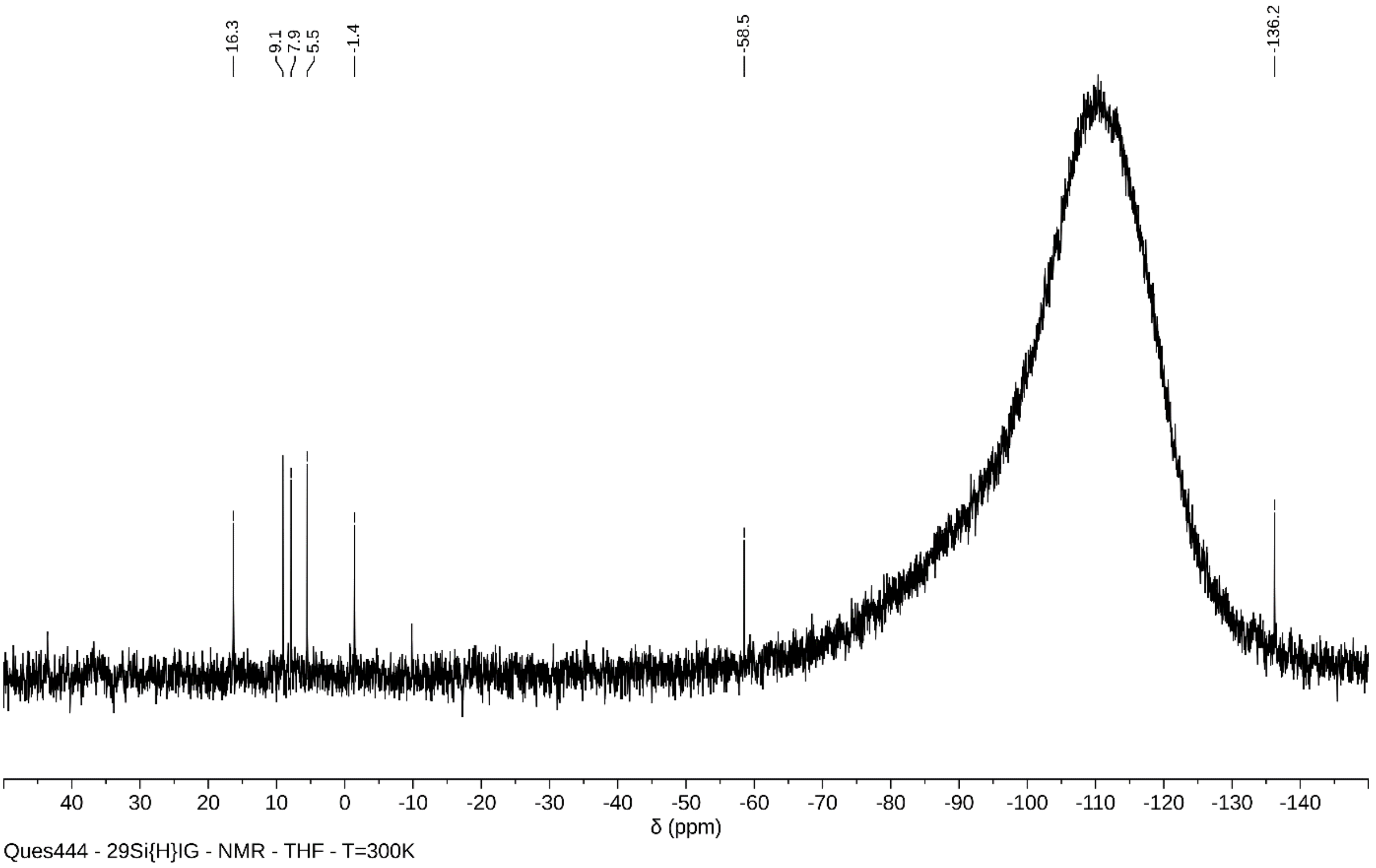

Figure S60. $^{29}Si\{^{1}H\}$ IG NMR spectrum of **3** in THF-d_8 at 300 K and 99 MHz.

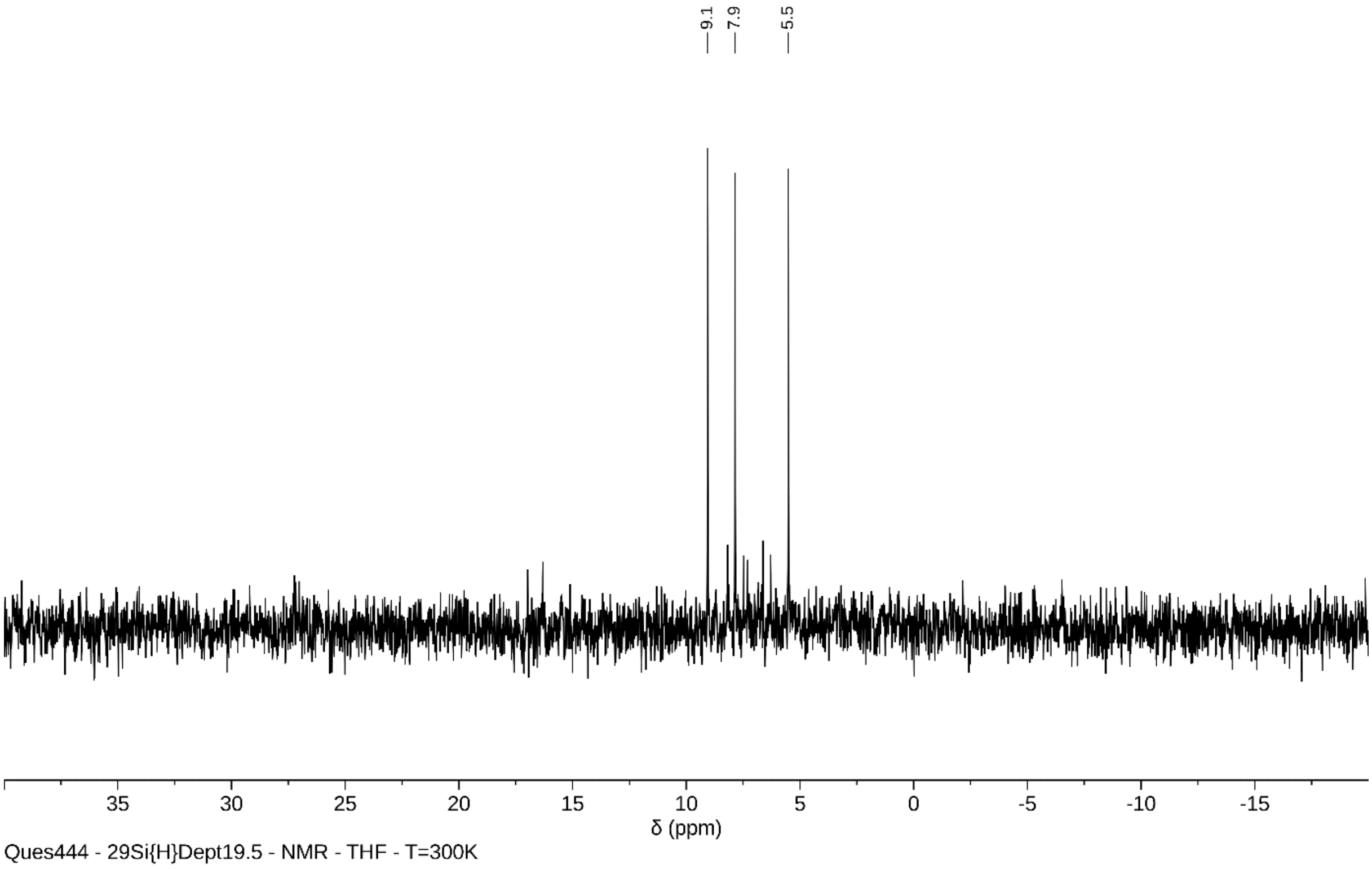

Figure S61. $^{29}Si\{^{1}H\}$ DEPT-19.5 NMR spectrum of **3** in THF-d_8 at 300 K and 99 MHz.

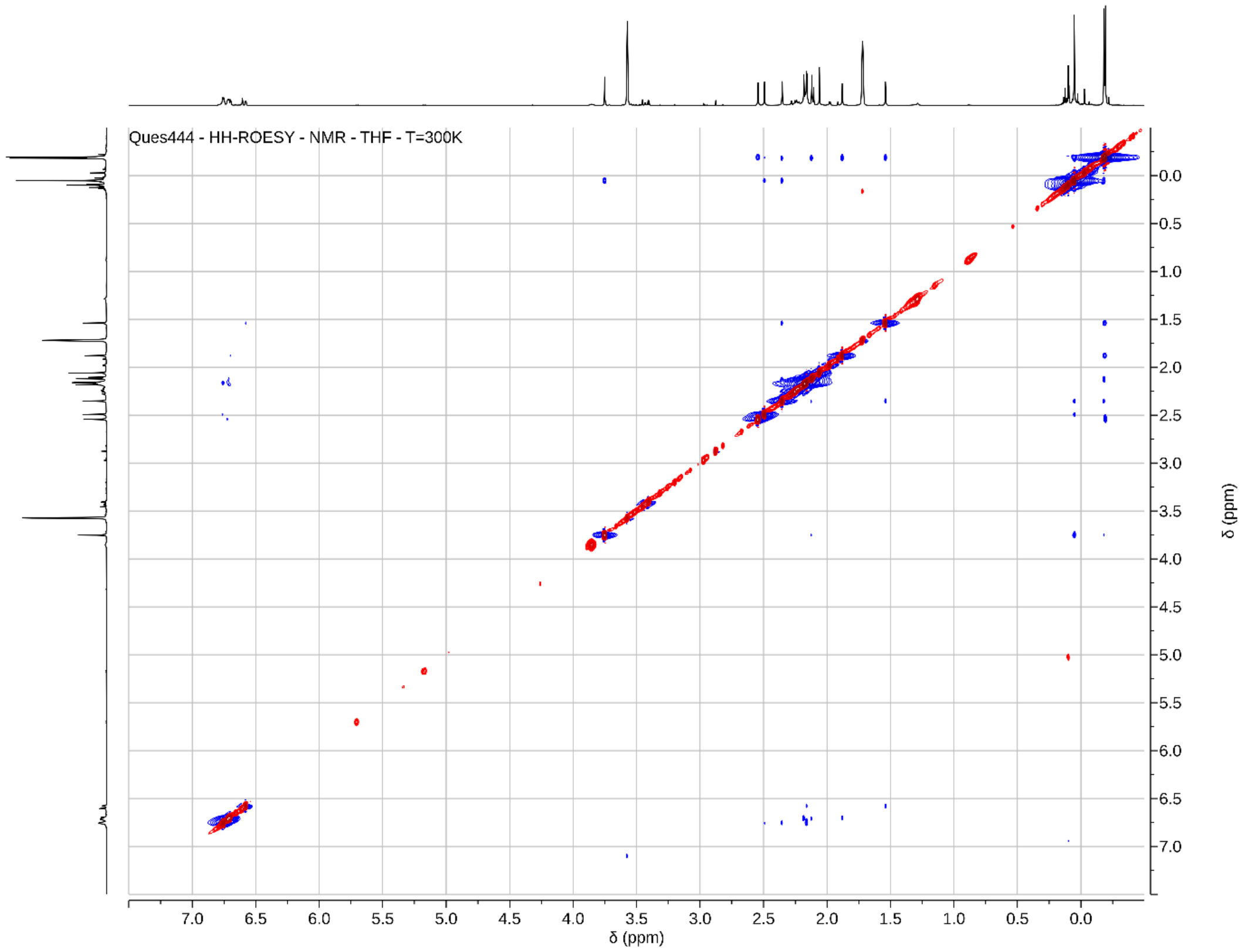

Figure S62. $^{1}H,^{1}H$ ROESY NMR spectrum of **3** in THF-d_8 at 300 K and 500 MHz.

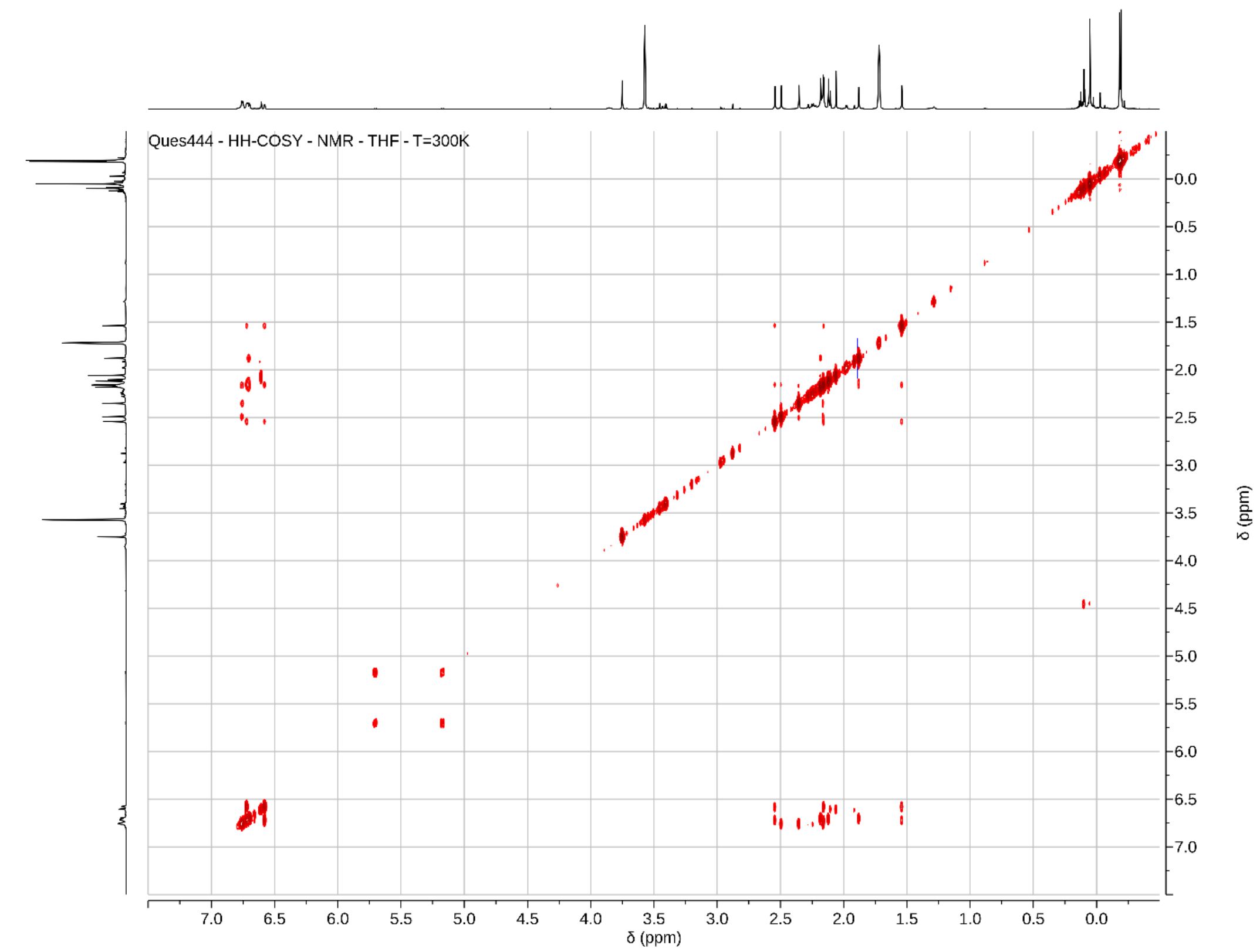

Figure S63. $^{1}H,^{1}H$ COSY NMR spectrum of **3** in THF-d_8 at 300 K and 500 MHz.

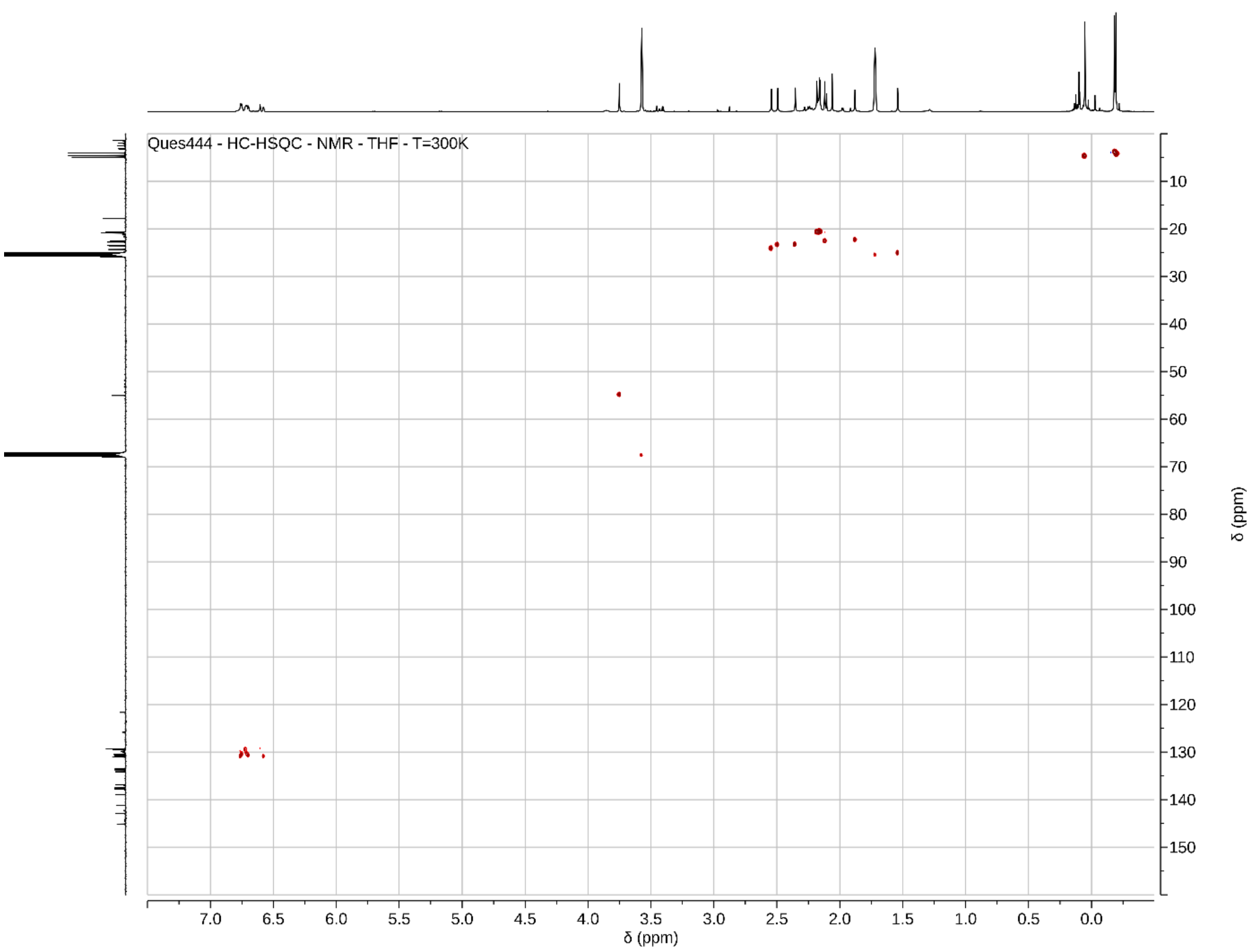

Figure S64. $^{1}H,^{13}C$ HSQC NMR spectrum of **3** in THF-d_8 at 300 K and 500 MHz (^{1}H) and 126 MHz (^{13}C).

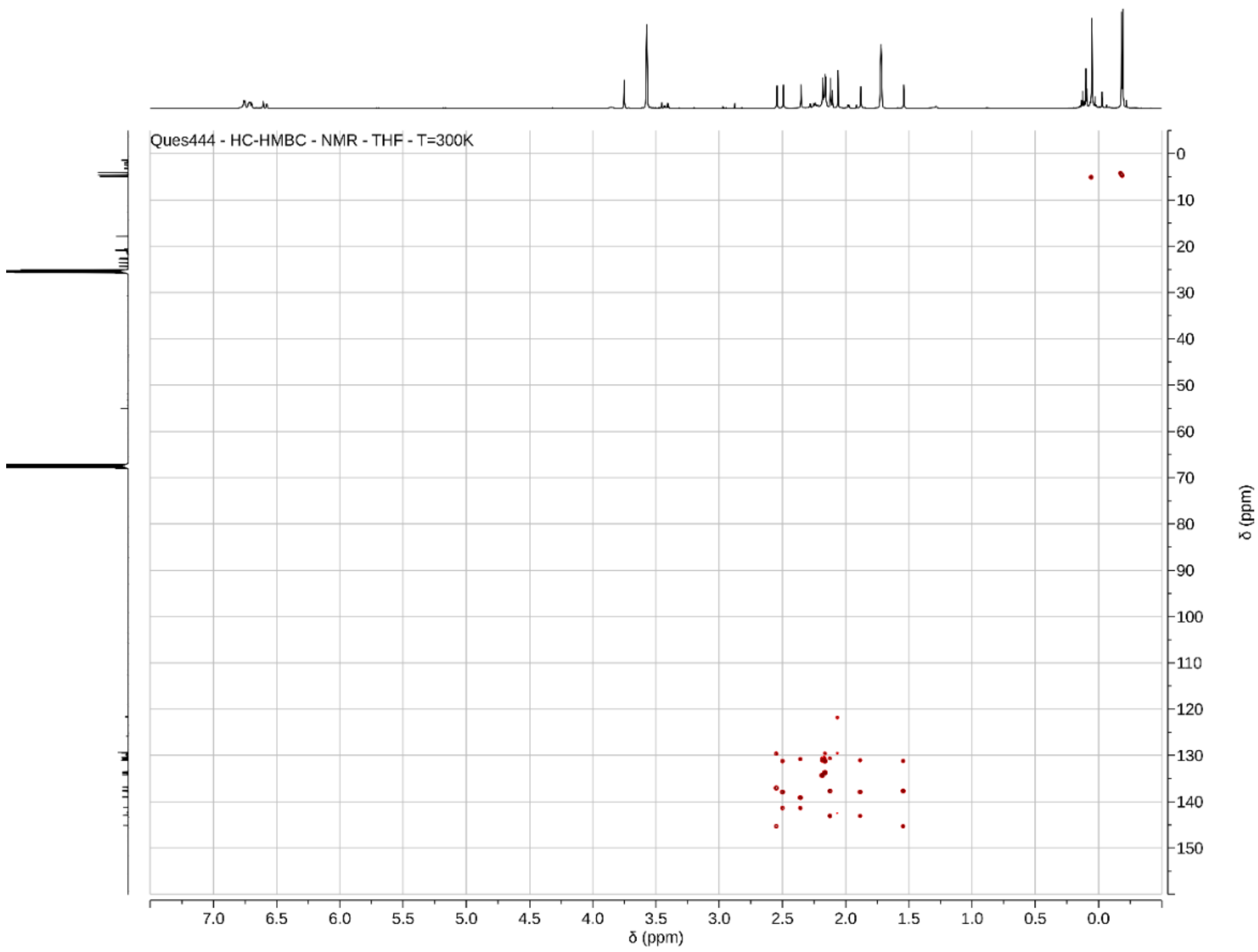

Figure S65. $^{1}H,^{13}C$ HMBC NMR spectrum of **3** in THF-d_8 at 300 K and 500 MHz (^{1}H) and 126 MHz (^{13}C).

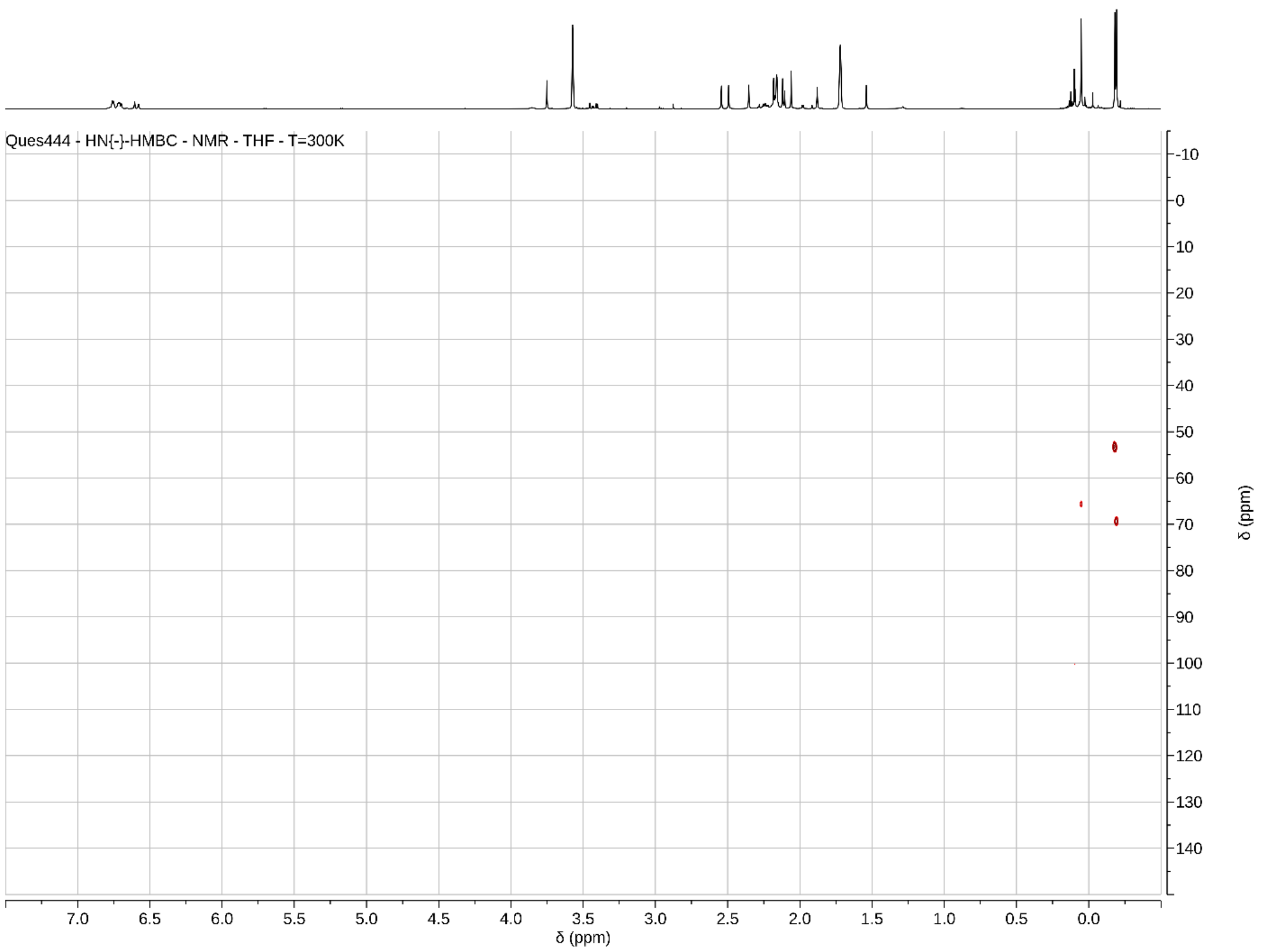

Figure S66. $^{1}H,^{15}N$ HMBC NMR spectrum of **3** in THF-d_8 at 300 K and 500 MHz (^{1}H) and 51 MHz (^{15}N).

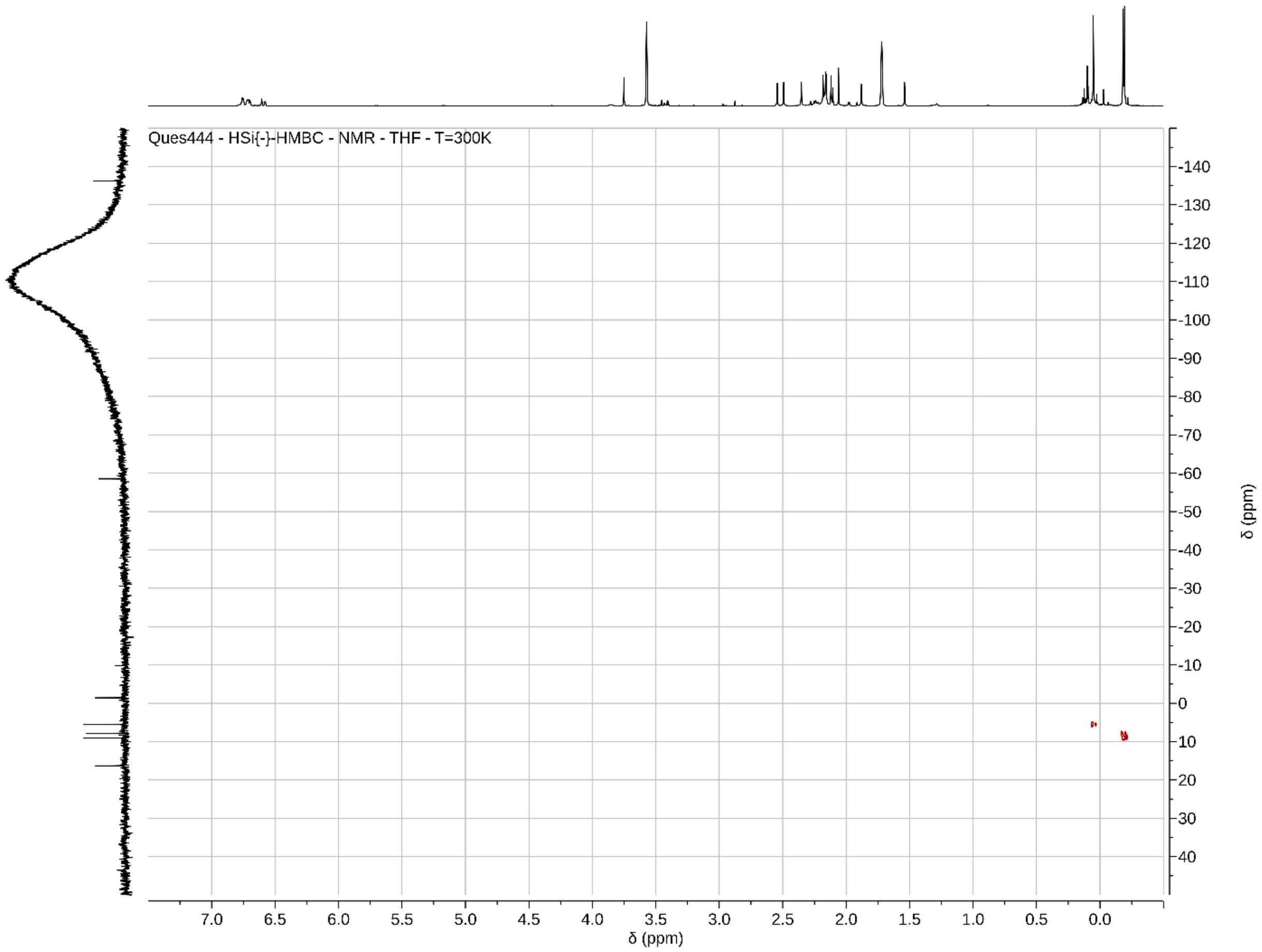

Figure S67. ^{1}H,^{29}Si HMBC NMR spectrum of **3** in THF-d_8 at 300 K and 500 MHz (^{1}H) and 99 MHz (^{29}Si).

2.6 $H_2(OMe)_3Si_4\{N(SiMe_3)Mes\}_3$ (4)

Tetrasilatetraamide $Si_4\{N(SiMe_3)Mes\}_4$ **F** (267.0 mg, 0.28 mmol, 1 eq.) was dissolved in THF (5 mL) and cooled to −88 °C using an *i*PrOH dry ice cooling bath. To this solution MeOH (37 mg, 1.14 mmol, 0.05 mL, 4 eq.) was added dropwise. The solution was slowly allowed to reach room temperature and was stirred for 18 h. The color changed from a deep red to a light yellow. All volatile compounds were removed in vacuo.

Yield: approx. 46 % (by ^{1}H NMR spectroscopy).

^{1}H NMR (C_6D_6, 500.23 MHz, 300 K): *δ* (ppm) = 6.81 (m, 1H, *meta*–Ar–*H*), 6.80 (m, 1H, *meta*–Ar–*H*), 6.79 (m, 1H, *meta*–Ar–*H*), 6.75 (s, 1H, *meta*–Ar–*H*), 6.75 (s, 1H, *meta*–Ar–*H*), 6.00 (d, 1H, Si–*H*, $^1J_{Si–H}$ = 193 Hz, $^3J_{H–H}$ = 7.6 Hz), 5.46 (d, 1H, Si–*H*, $^3J_{H–H}$ = 7.6 Hz), 3.49 (s, 3H, O–CH_3), 3.46 (s, 3H, O–CH_3), 3.01 (s, 3H, O–CH_3), 2.43 (s, 3H, *para*–Ar–CH_3), 2.39 (s, 3H, *ortho*–Ar–CH_3), 2.37 (m, 3H, *para*–Ar–CH_3), 2.34 (m, 3H, *para*–Ar–CH_3), 2.20 (m, 3H, *ortho*–Ar–CH_3), 2.17 (s, 3H, *ortho*–Ar–CH_3), 2.16 (s, 3H, *ortho*–Ar–CH_3), 2.12 (s, 3H, *ortho*–Ar–CH_3), 0.29 (s, 9H, Si–(CH_3)$_3$), 0.14 (s, 9H, Si–(CH_3)$_3$), 0.07 (s, 9H, Si–(CH_3)$_3$).

^{13}C{^{1}H} NMR (C_6D_6, 125.80 MHz, 300 K): *δ* (ppm) = 143.8 (s, *ipso*–Ar–*C*), 142.5 (s, *ipso*–Ar–*C*), 142.4 (s, *ipso*–Ar–*C*), 137.5 (s, *ortho*–Ar–*C*), 137.3 (s, *ortho*–Ar–*C*), 137.2 (s, *ortho*–Ar–*C*), 137.0 (s, *ortho*–Ar–*C*), 137.0 (s, *ortho*–Ar–*C*), 136.5 (s, *ortho*–Ar–*C*), 133.6 (s, *para*–Ar–*C*), 133.5 (s, *para*–Ar–*C*), 133.4 (s, *para*–Ar–*C*), 129.7 (s, *meta*–Ar–*C*H), 129.6 (s, *meta*–Ar–*C*H), 129.5 (s, *meta*–Ar–*C*H), 129.5 (s, *meta*–Ar–*C*H), 129.5 (s, *meta*–Ar–*C*H), 129.5 (s, *meta*–Ar–*C*H), 55.5 (s, O–*C*H_3), 53.0 (s, O–*C*H_3), 51.5 (s, O–*C*H_3), 21.1 (s, *ortho*/*para*–Ar–*C*H_3), 21.1 (s, *ortho*/*para*–Ar–*C*H_3), 21.1 (s, *ortho*/*para*–Ar–*C*H_3), 21.0 (s, *para*–Ar–*C*H_3), 20.9 (s, *ortho*–Ar–*C*H_3), 20.9 (s, *ortho*–Ar–*C*H_3), 20.85 (s, *ortho*–Ar–*C*H_3), 20.8 (s, *ortho*–Ar–*C*H_3), 20.6 (s, *ortho*–Ar–*C*H_3), 3.1 (s, Si–(*C*H_3)$_3$), 2.5 (s, Si–(*C*H_3)$_3$), 2.0 (s, Si–(*C*H_3)$_3$).

^{13}C{^{1}H} DEPT-135 NMR (C_6D_6, 125.80 MHz, 300 K): *δ* (ppm) = 129.7 (s, *meta*–Ar–*C*H), 129.6 (s, *meta*–Ar–*C*H), 129.5 (s, *meta*–Ar–*C*H), 129.5 (s, *meta*–Ar–*C*H), 129.5 (s, *meta*–Ar–*C*H), 129.5 (s, *meta*–Ar–*C*H), 55.5 (s, O–*C*H_3), 53.0 (s, O–*C*H_3), 51.5 (s, O–*C*H_3), 21.1 (s, *ortho*/*para*–Ar–*C*H_3), 21.1 (s, *ortho/para*–Ar–*C*H_3), 21.1 (s, *ortho/para*–Ar–*C*H_3), 21.0 (s, *para*–Ar–*C*H_3), 20.9 (s, *ortho*–Ar–*C*H_3), 20.9 (s, *ortho*–Ar–*C*H_3), 20.85 (s, *ortho*–Ar–*C*H_3), 20.8 (s, *ortho*–Ar–*C*H_3), 20.6 (s, *ortho*–Ar–*C*H_3), 3.1 (s, Si–(*C*H_3)$_3$), 2.5 (s, Si–(*C*H_3)$_3$), 2.0 (s, Si–(*C*H_3)$_3$).

^{1}H,^{15}N HMBC NMR (C_6D_6, 50.70 MHz, 300 K): *δ* (ppm) = 57.9 (s, *N*), 53.3 (s, N), 23.9 (s, *N*).

^{29}Si{^{1}H} IG NMR (C_6D_6, 99.38 MHz, 300 K): *δ* (ppm) = 8.2 (s, *Si*–(CH_3)$_3$), 7.6 (s, *Si*–(CH_3)$_3$), 7.3 (s, *Si*–(CH_3)$_3$), −0.5 (s, H_3CO–*Si*–H), −9.6 (s, *Si*–OCH_3), −9.7 (s, *Si*–OCH_3), −46.3 (s, *Si*–H).

^{29}Si{^{1}H} DEPT-19.5 NMR (C_6D_6 , 99.38 MHz, 300 K): *δ* (ppm) = 8.2 (s, *Si*–(CH_3)$_3$), 7.6 (s, *Si*–(CH_3)$_3$), 7.3 (s, *Si*–(CH_3)$_3$).

^{29}Si{^{1}H} DEPT-90 NMR (C_6D_6, 99.38 MHz, 300 K): *δ* (ppm) = −0.5 (s, CH_3O–*Si*–H), −46.3 (s, *Si*–H).

ESI-MS (positive mode, solution in THF): *m*/*z* = $[MNa]^+$ 848.3782 (848.3772 calc.).

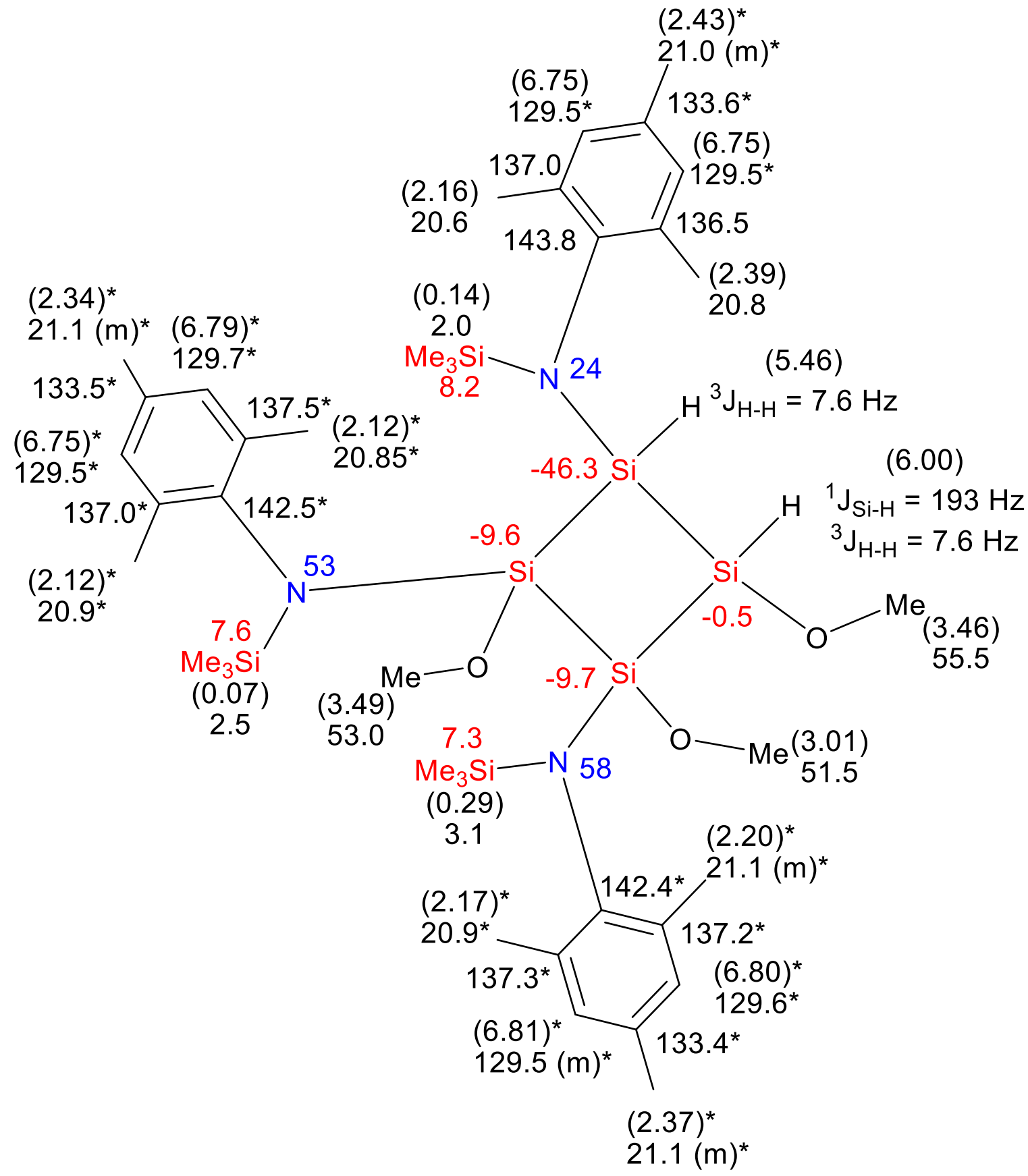

Figure S68. Assignment of chemical shifts to **4** (in C_6D_6 at 300 K). Color code: ^{1}H black in brackets, ^{13}C black, ^{15}N blue, and ^{29}Si red.

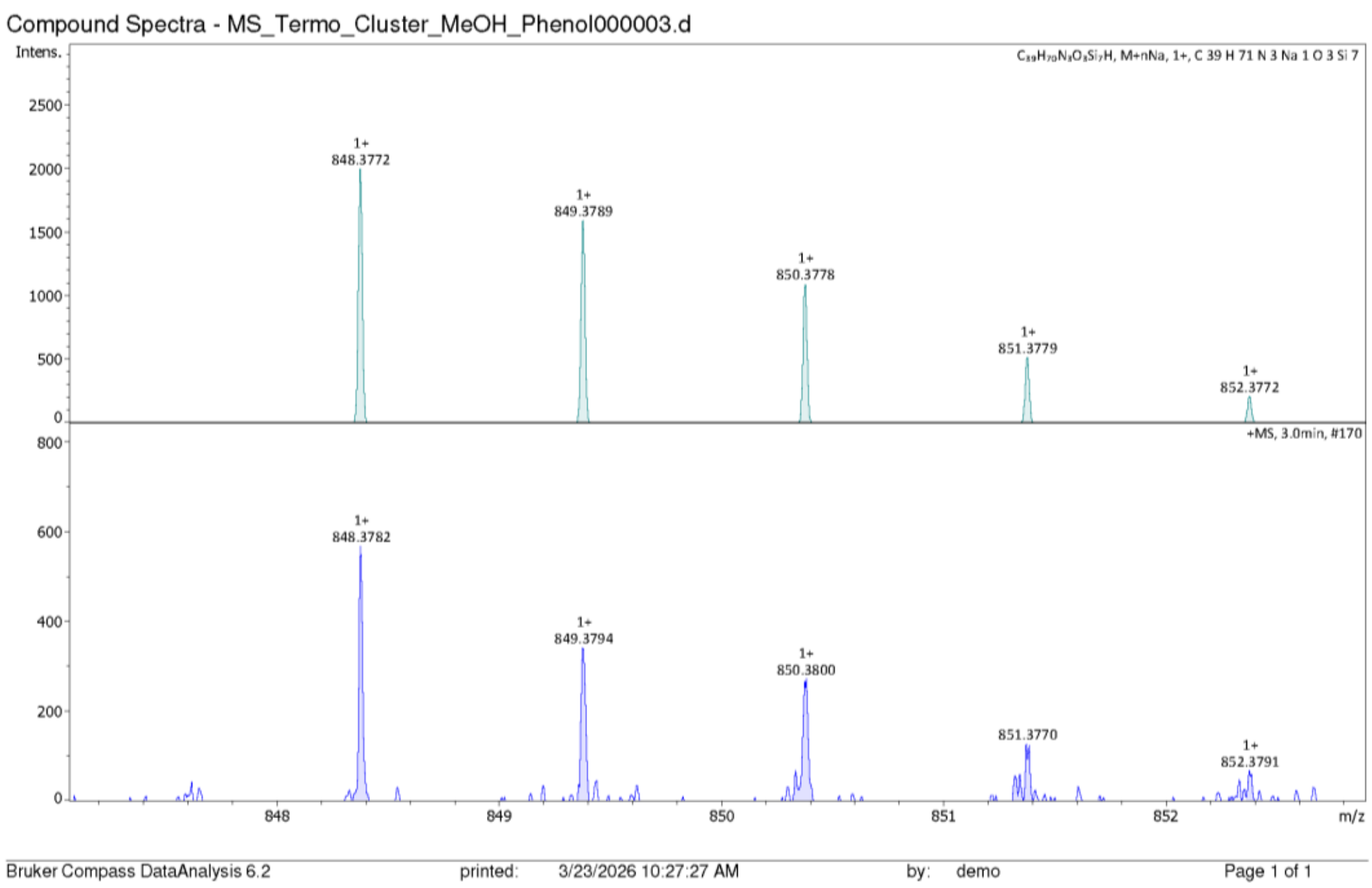

Figure S69. ESI-MS spectrum (positive mode) of **4** in THF.

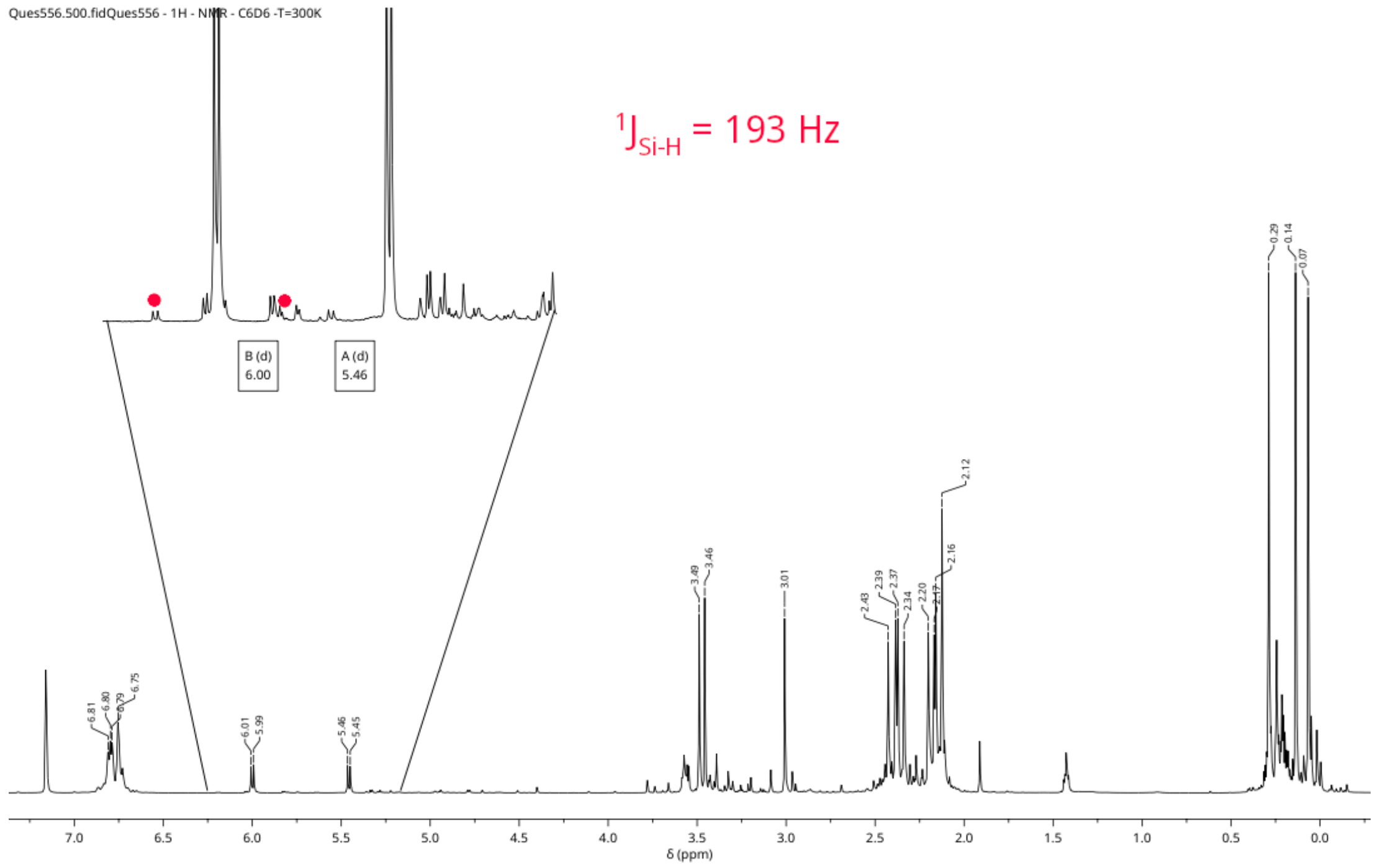

Figure S70. ^{1}H NMR spectrum of **4** in C_6D_6 at 300 K and 500 MHz.

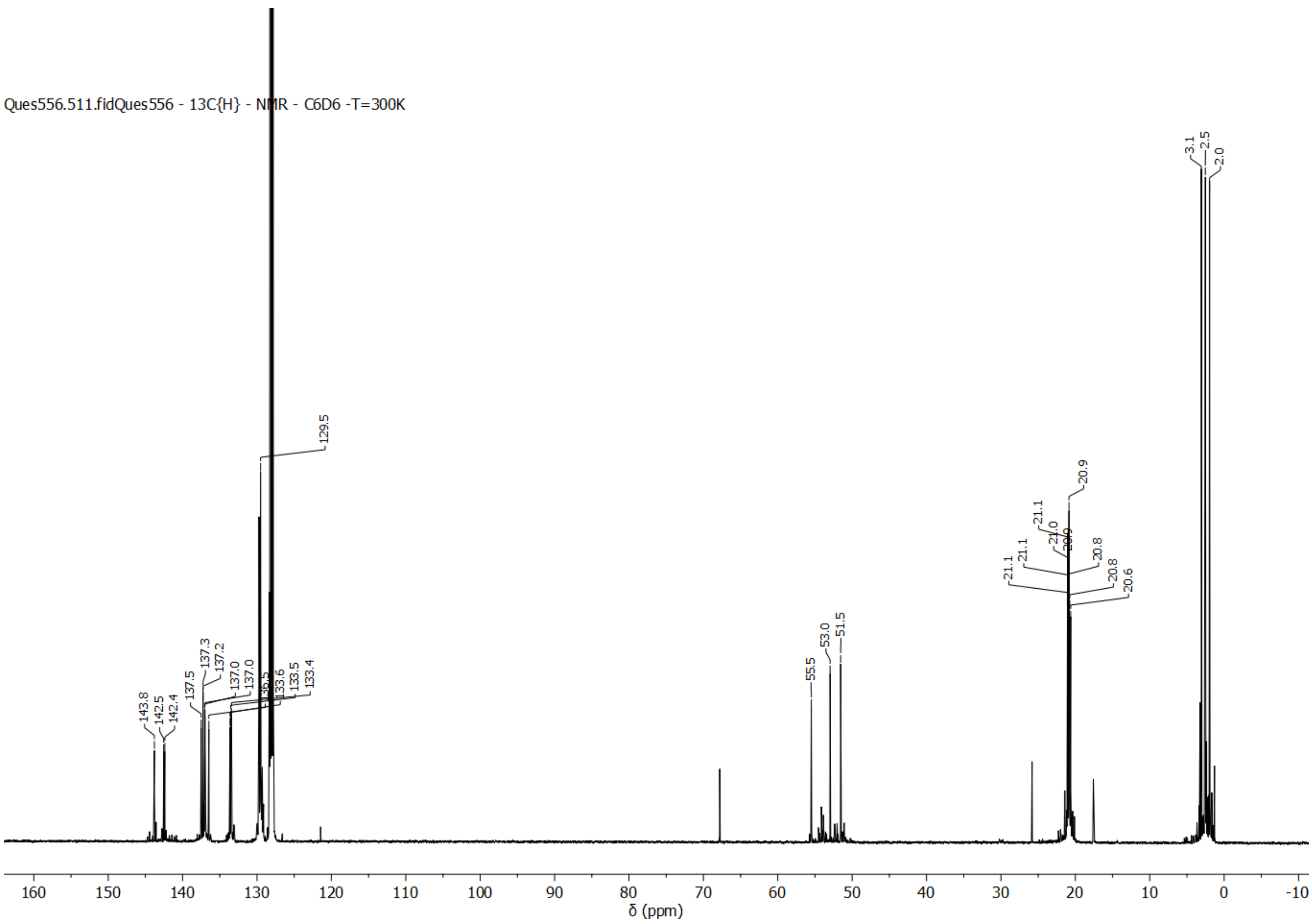

Figure S71. $^{13}C\{^1H\}$ NMR spectrum of **4** in C_6D_6 at 300 K and 126 MHz.

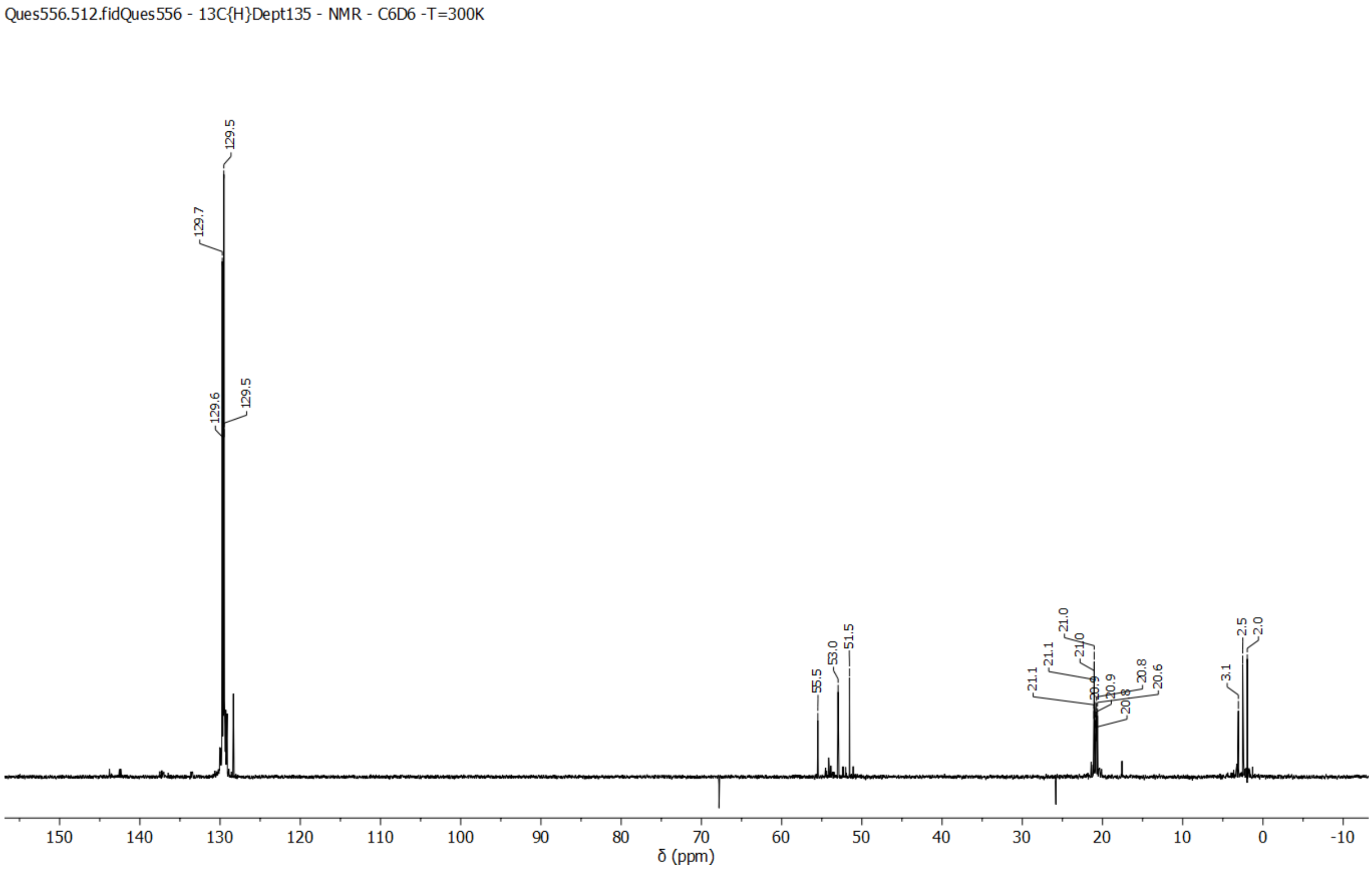

Figure S72. $^{13}C\{^{1}H\}$ DEPT-135 NMR spectrum of **4** in C_6D_6 at 300 K and 126 MHz.

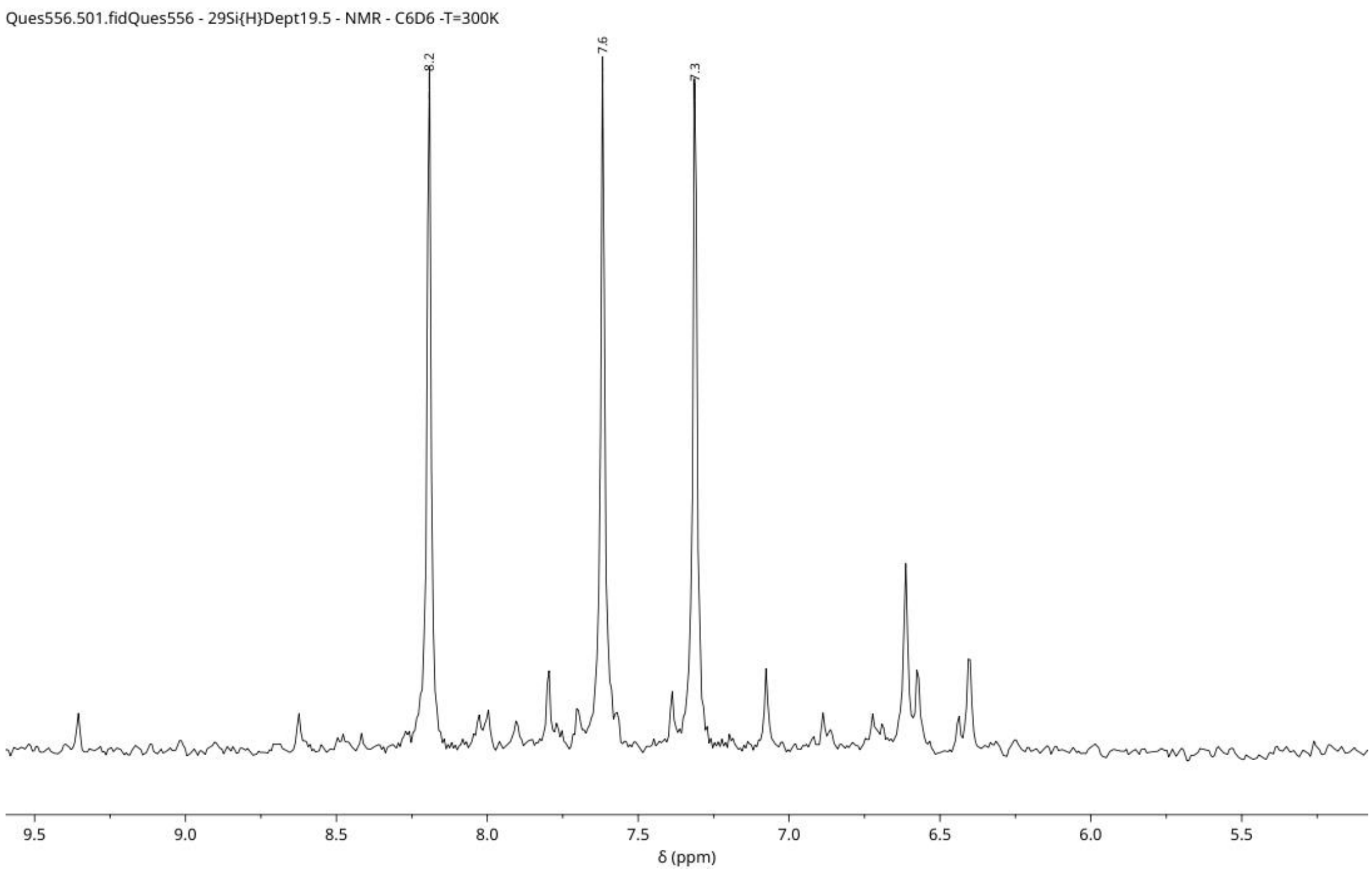

Figure S73. $^{29}Si\{^{1}H\}$ DEPT-19.5 NMR spectrum of **4** in C_6D_6 at 300 K and 99 MHz.

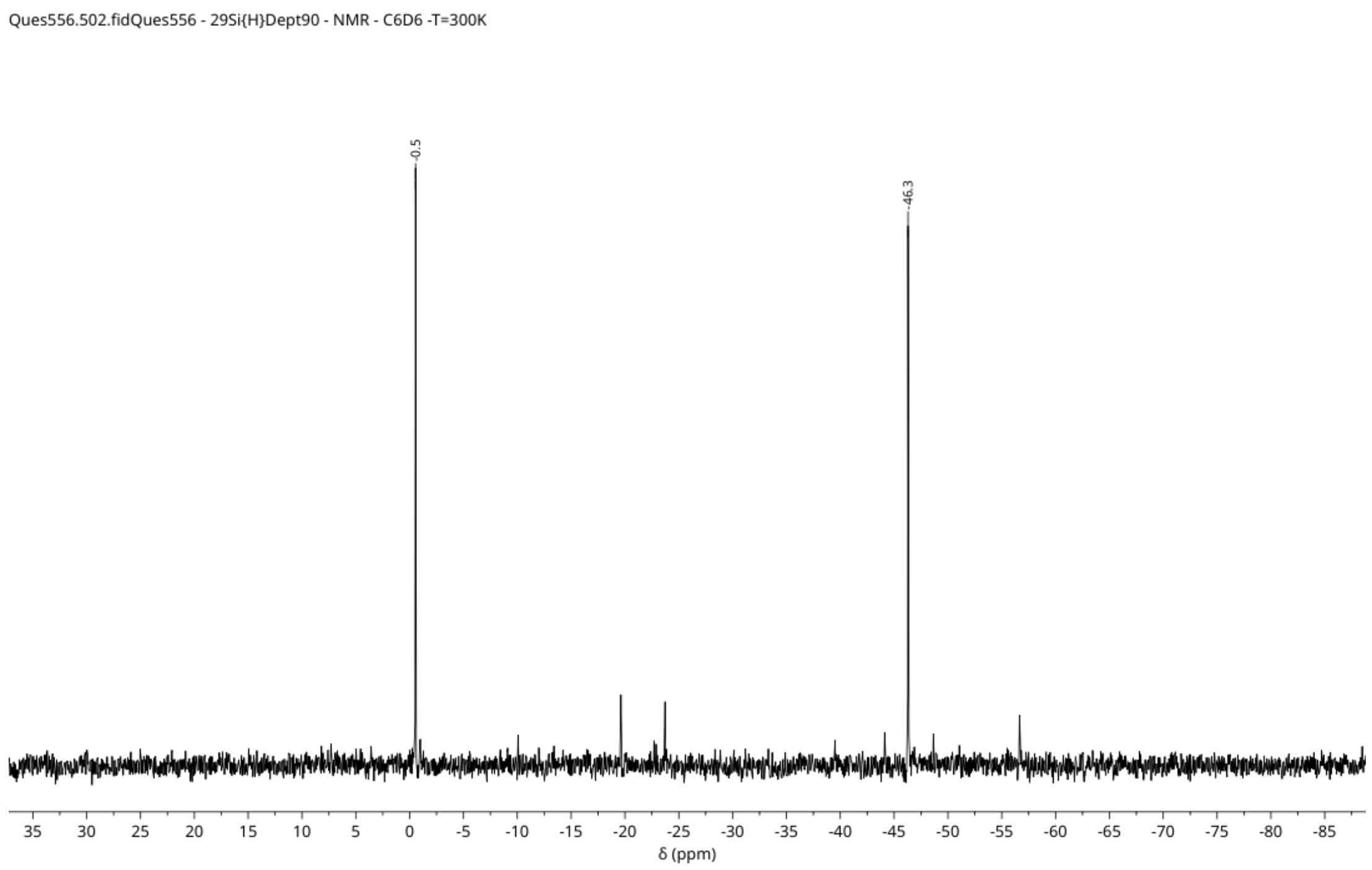

Figure S74. $^{29}Si\{^1H\}$ DEPT-90 NMR spectrum of **4** in C_6D_6 at 300 K and 99 MHz.

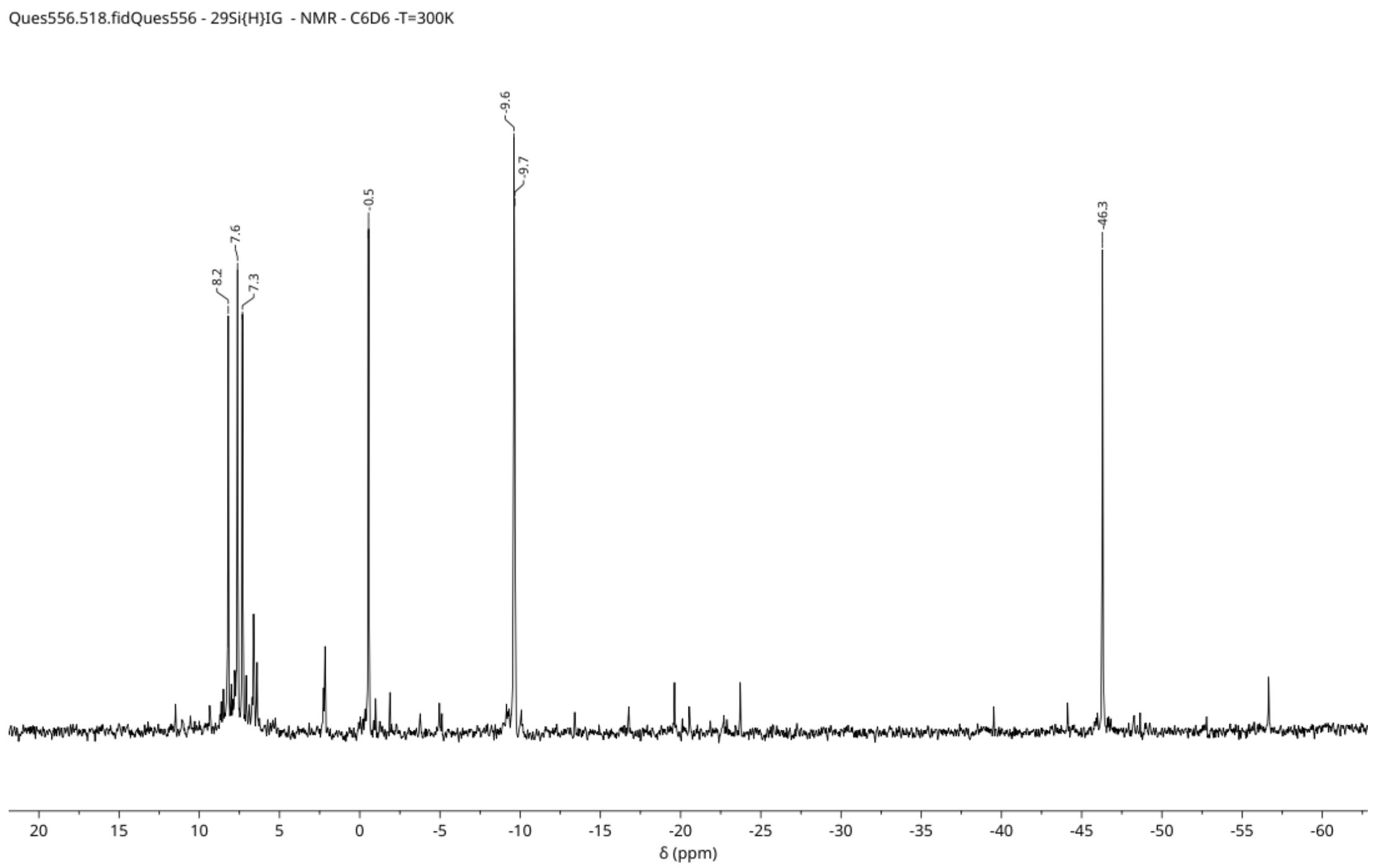

Figure S75. $^{29}Si\{^1H\}$ IG NMR spectrum of **4** in C_6D_6 at 300 K and 99 MHz.

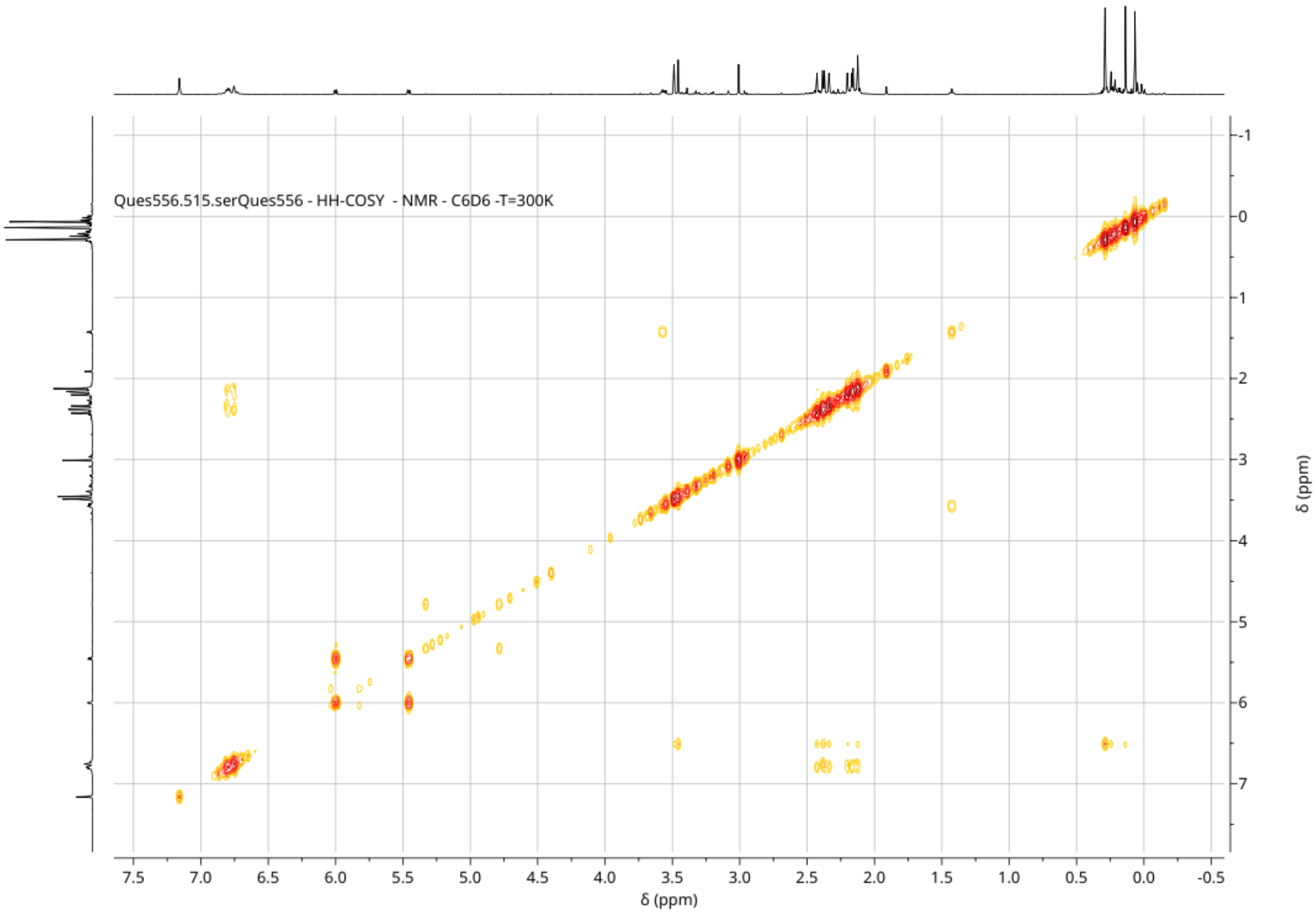

Figure S76. $^{1}H,^{1}H$ COSY NMR spectrum of **4** in C_6D_6 at 300 K and 500 MHz.

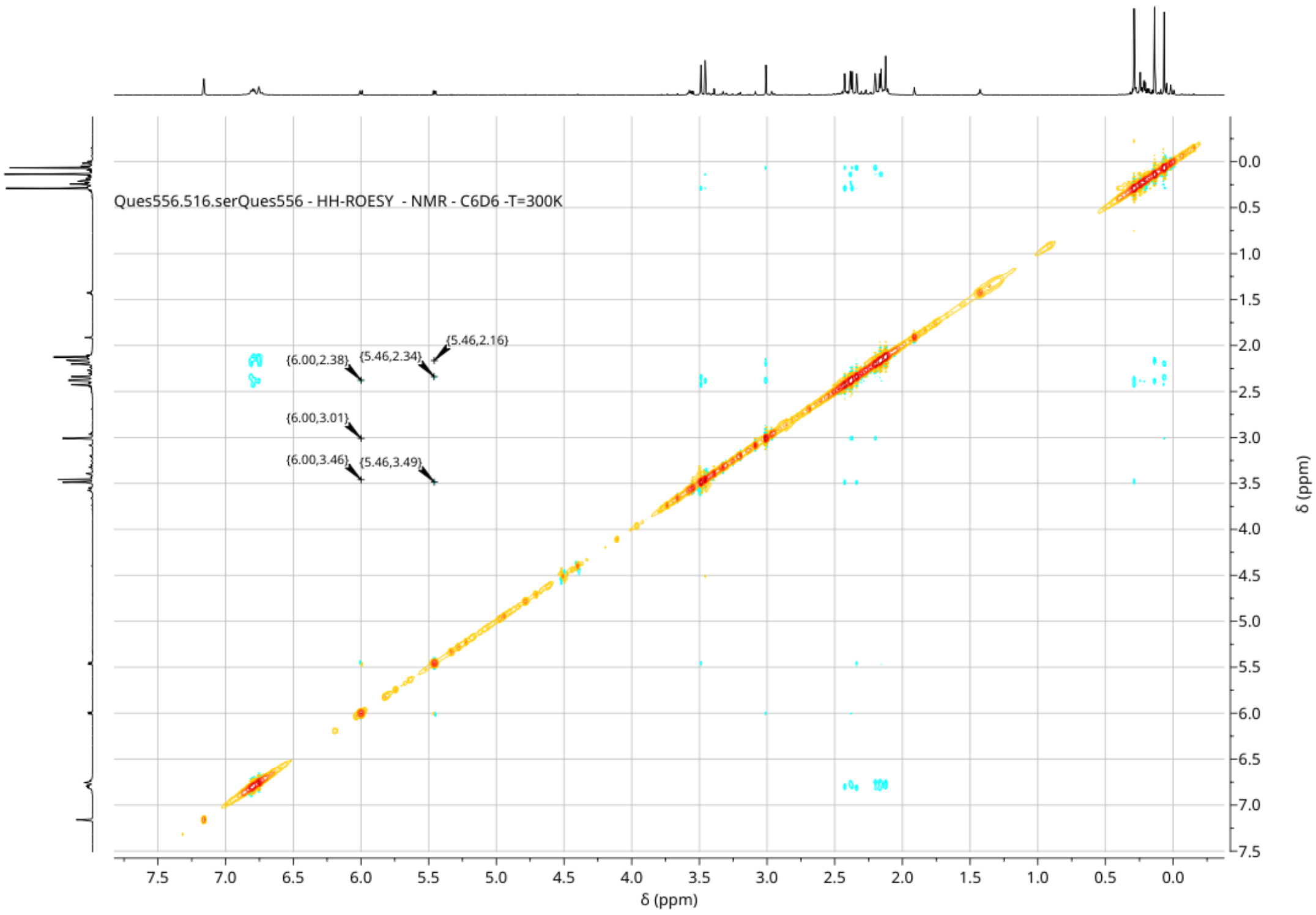

Figure S77. $^{1}H,^{1}H$ ROESY NMR spectrum of **4** in C_6D_6 at 300 K and 500 MHz.

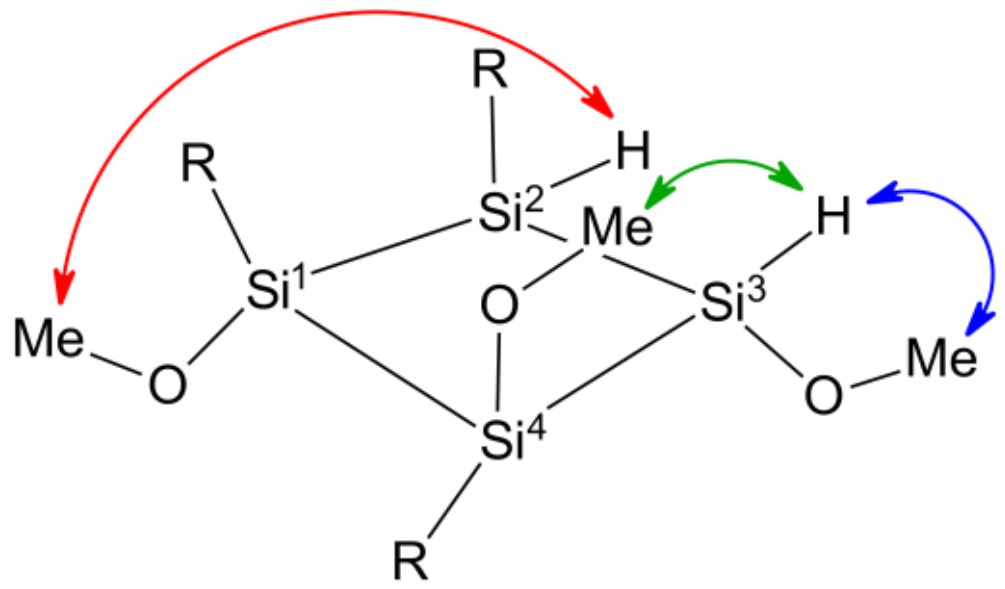

R = N(SiMe$_3$)Mes

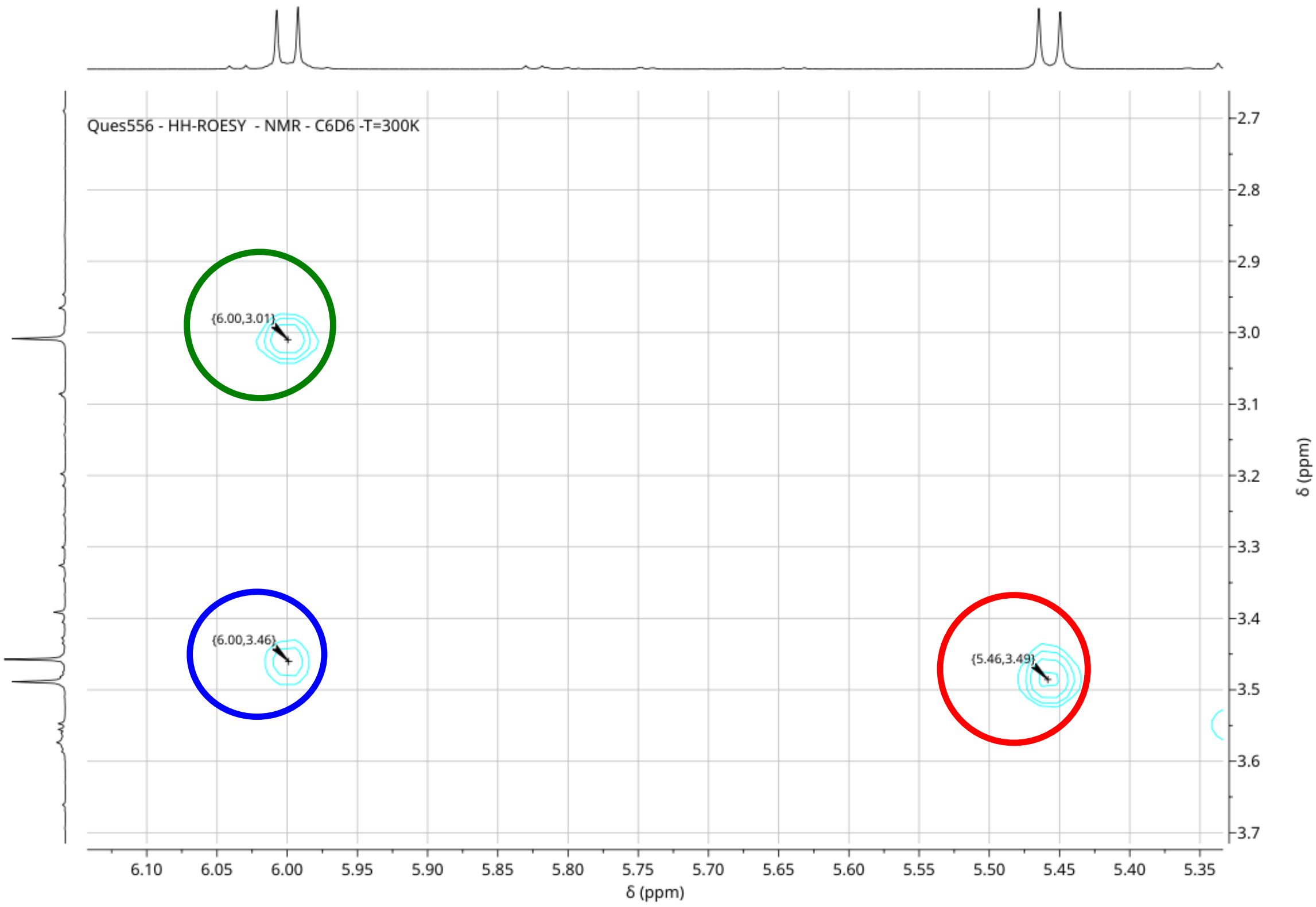

Figure S78. Zoom into the $^1H,^1H$ ROESY NMR spectrum of **4** in C_6D_6 at 300 K and 500 MHz.

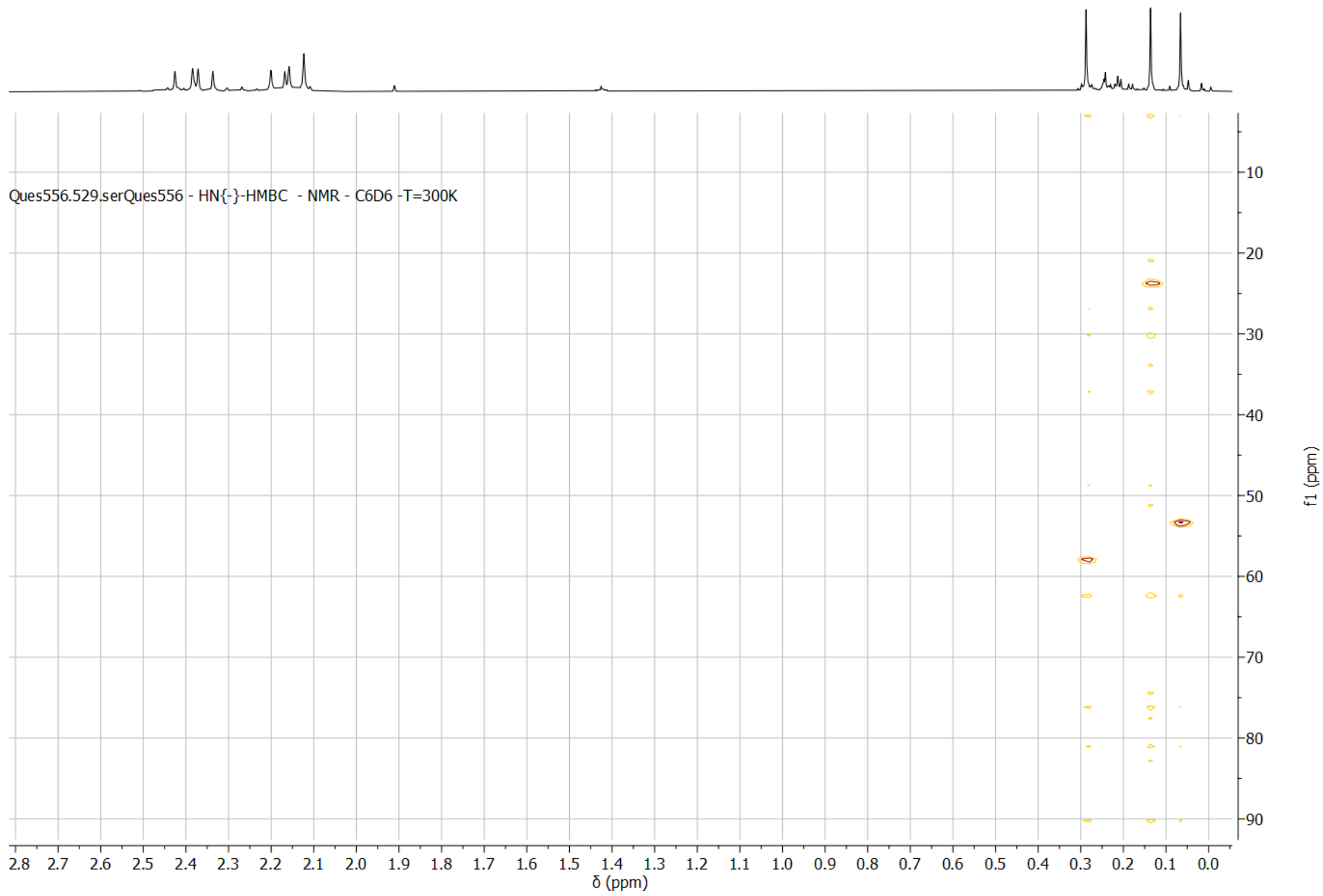

Figure S79. ^{1}H,^{15}N HMBC NMR spectrum of **4** in C_6D_6 at 300 K and 500 MHz (^{1}H) and 70 MHz (^{15}N).

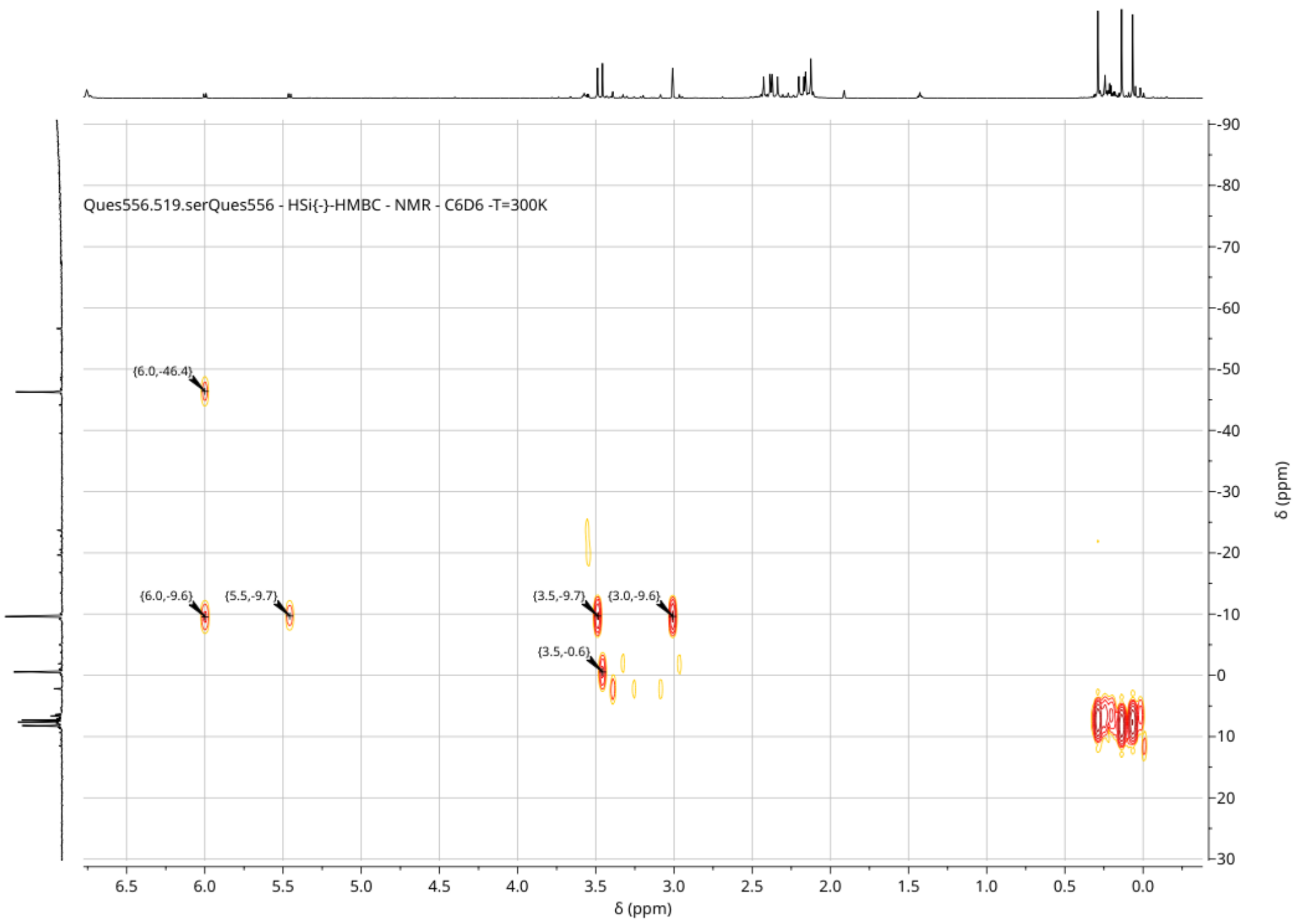

Figure S80. ^{1}H,^{29}Si HMBC NMR spectrum of **4** in C_6D_6 at 300 K and 500 MHz (^{1}H) and 99 MHz (^{29}Si).

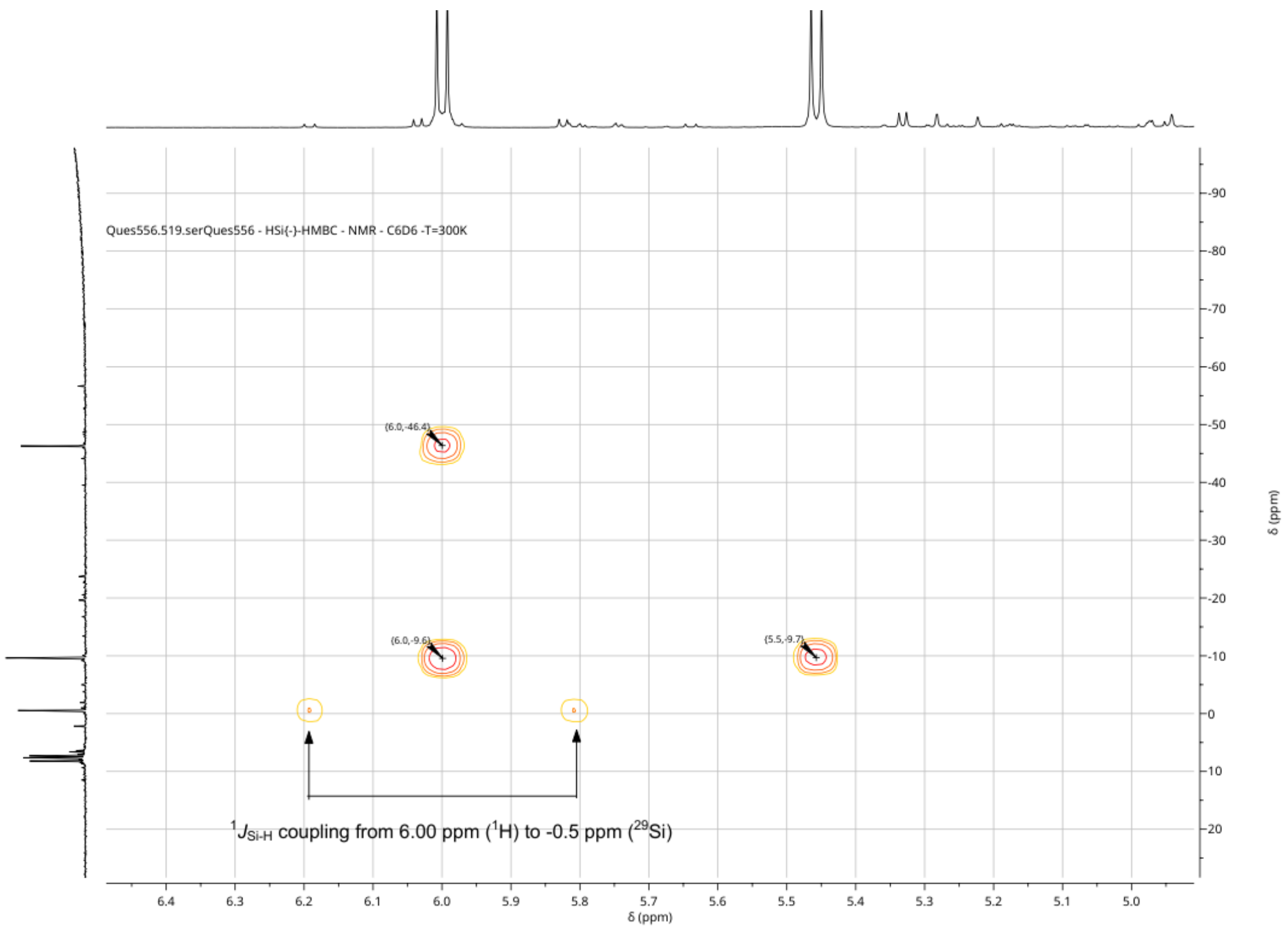

Figure S81. Zoom into the $^{1}H,^{29}Si$ HMBC NMR spectrum of **4** in C_6D_6 at 300 K and 500 MHz (^{1}H) and 99 MHz (^{29}Si).

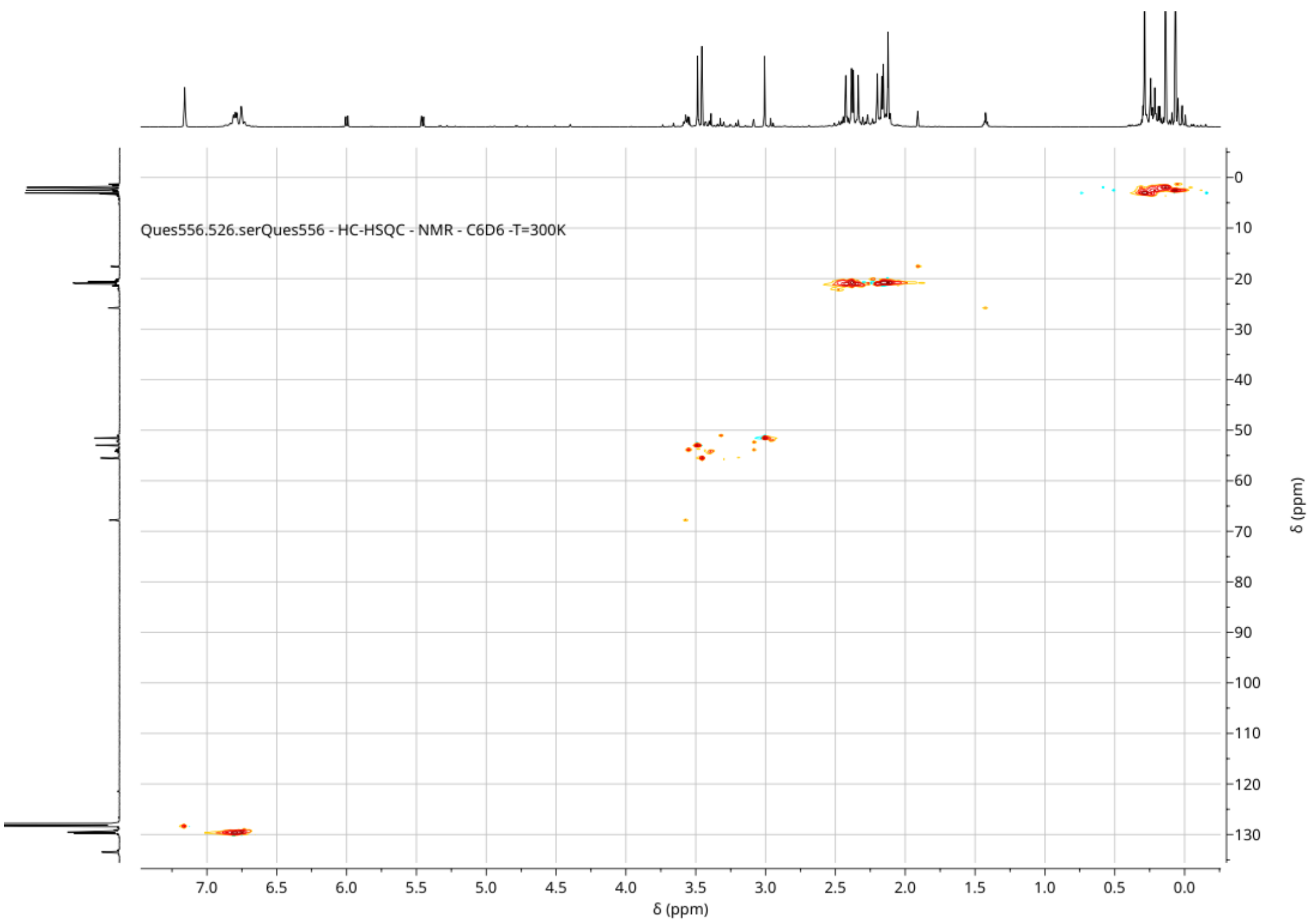

Figure S82. $^{1}H,^{13}C$ HSQC NMR spectrum of **4** in C_6D_6 at 300 K and 500 MHz (^{1}H) and 126 MHz (^{13}C).

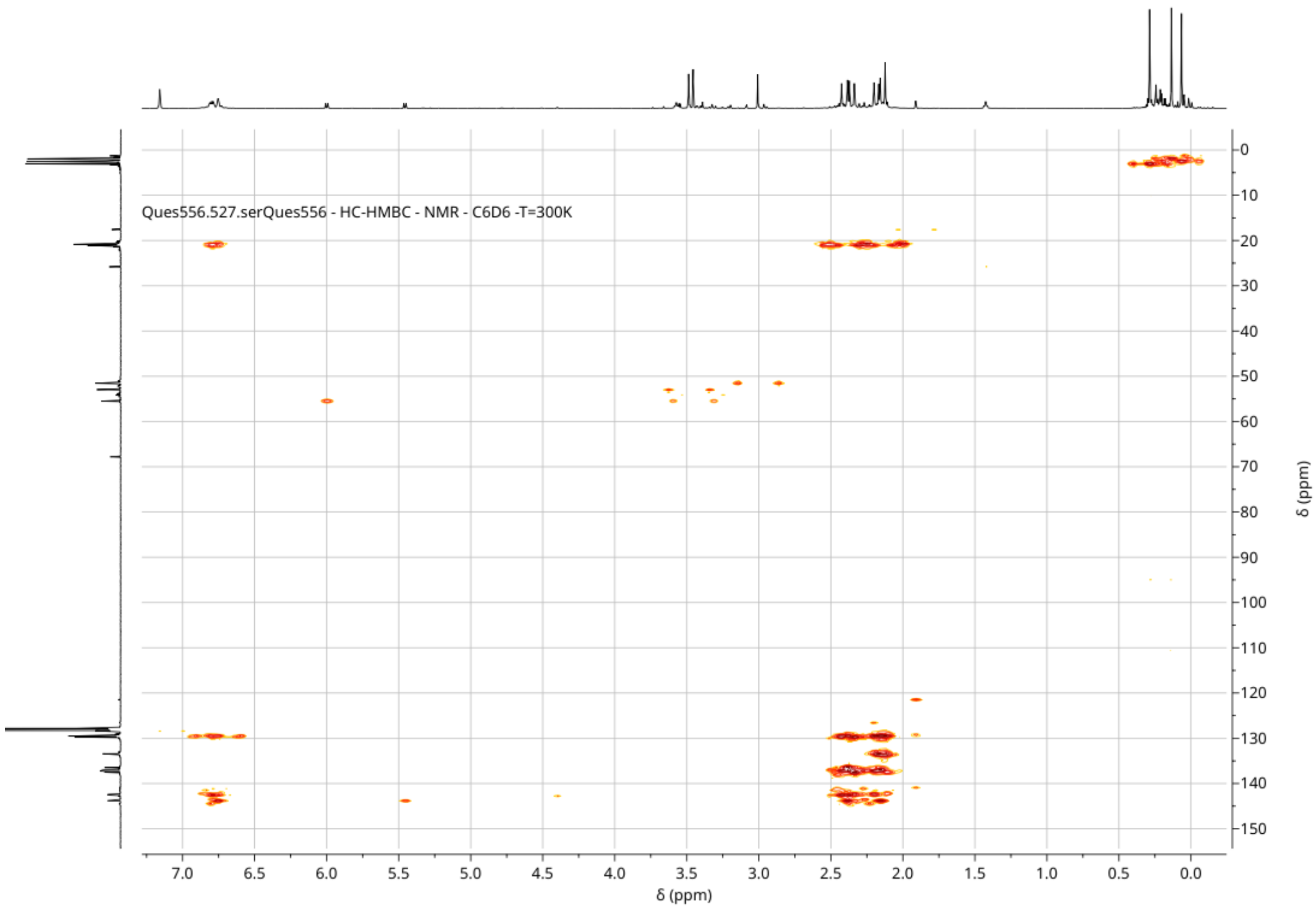

Figure S83. ^1H,^{13}C HMBC NMR spectrum of **4** in C_6D_6 at 300 K and 500 MHz (^1H) and 126 MHz (^{13}C).

2.7 $(OEt)_2Si_8\{N(SiMe_3)Mes\}_6$ (5)

Ethanol (25.9 mg, 1.13 mmol, 2.0 eq., 3.28 mL solution v/v 1:100 in THF) was slowly added dropwise to a cooled (−78 °C) solution of $Si_4\{N(SiMe_3)Mes\}_4$ **F** (528.0 mg, 0.563 mmol, 1eq.) in THF (20 mL) using an *iso*-propanol liquid nitrogen cooling bath. The reaction was allowed to warm to room temperature over 2 h and subsequently was heated to 55 °C for 30 min to drive the reaction to completion. After cooling back to room temperature all volatiles were removed *in vacuo*. The residue was washed with 3×2 mL DME and dried *in vacuo*. The product $(OEt)_2Si_8\{N(SiMe_3)Mes\}_6$ (**5**) was obtained as a yellow solid. Single crystals were obtained from a concentrated $CDCl_3$ solution (0.6 mL) at 25 °C inside an NMR tube.

Yield: 100 mg (0.0656 mmol, 30 %).

Melting point: 250 °C.

^{1}H NMR ($CDCl_3$, 300 K, 400.1 MHz): δ (ppm) = 6.86 (s, 2H, Mes–CH_{arom}), 6.73 (s, 2H, Mes–C$H_{arom.}$), 6.71 (s, overlapped, 2H, Mes–C$H_{arom.}$), 6.71–6.69 (m, overlapped, 2H, Mes–C$H_{arom.}$), 6.71–6.69 (m, overlapped, 2H, Mes–C$H_{arom.}$), 6.59 (s, 2H, Mes–C$H_{arom.}$), 4.32 (dq, 2H, O(CH_2)CH$_3$, $^2J_{H,H}$ = 9.6 Hz, $^3J_{H,H}$ = 6.6 Hz), 3.34 (dq, 2H, O(CH_2)CH$_3$, $^2J_{H,H}$ = 9.6 Hz, $^3J_{H,H}$ = 6.8 Hz), 2.54 (s, 6H, *ortho*–Mes–CH_3), 2.35 (s, 6H, *ortho*–Mes–CH_3), 2.30 (s, 6H, *ortho*–Mes–CH_3), 2.20 (s, 6H, *para*–Mes–CH_3), 2.18 (s, overlapped, 6H, *para*–Mes–CH_3), 2.17 (s, overlapped, 6H, *para*–Mes–CH_3), 2.07 (s, 6H, *ortho*–Mes–CH_3), 1.97 (s, 6H, *ortho*–Mes–CH_3), 1.56 (s, 6H, *ortho*–Mes–CH_3), 1.20 (t, 6H, O(CH$_2$)CH_3, $^3J_{H,H}$ = 6.7 Hz), 0.05 (s, 18H, Si(CH_3)$_3$), −0.18 (s, 18H, Si(CH_3)$_3$), −0.28 (s, 18H, Si(CH_3)$_3$).

^{13}C{^{1}H} NMR ($CDCl_3$, 300 K, 100.6 MHz): δ (ppm) = 144.2 (s, 2C, Mes–C_{ipso}), 142.1 (s, 2C, Mes–C_{ipso}), 140.7 (s, 2C, Mes–C_{ipso}), 138.3 (s, 2C, Mes–C_{ortho}), 137.1 (s, 2C, Mes–C_{ortho}), 136.9 (s, 2C, Mes–C_{ortho}), 136.8 (s, 2C, Mes–C_{ortho}), 136.5 (s, 2C, Mes–C_{ortho}), 136.2 (s, 2C, Mes–C_{ortho}), 133.0 (s, 2C, Mes–C_{para}), 132.9 (s, 2C, Mes–C_{para}), 132.7 (s, 2C, Mes–C_{para}), 130.3 (s, 2C, Mes–C_{meta}), 130.1 (s, 2C, Mes–C_{meta}), 129.9 (s, 2C, Mes–C_{meta}), 129.8 (s, 2C, Mes–C_{meta}), 129.7 (s, 2C, Mes–C_{meta}), 128.6 (s, 2C, Mes–C_{meta}), 63.0 (s, 2C, O(CH$_2$)CH$_3$), 24.8 (s, 2C, *ortho*–Mes–CH$_3$), 24.1 (s, 2C, *ortho*–Mes–CH$_3$), 22.8 (s, 2C, *ortho*–Mes–CH$_3$), 22.7 (s, 2C, *ortho*–Mes–CH$_3$), 22.3 (s, 2C, *ortho*–Mes–CH$_3$), 21.9 (s, 2C, *ortho*–Mes–CH$_3$), 20.9 (s, 2C, *para*–Mes–CH$_3$), 20.8 (s, 2C, *para*–Mes–CH$_3$), 20.7 (s, 2C, *para*–Mes–CH$_3$), 18.8 (s, 2C, O(CH$_2$)CH$_3$), 5.3 (s, 6C, Si(CH$_3$)$_3$), 4.4 (s, 6C, Si(CH$_3$)$_3$), 3.6 (s, 6C, Si(CH$_3$)$_3$).

^{13}C{^{1}H} DEPT-135 NMR ($CDCl_3$, 300K, 100.6 MHz): δ (ppm) = 130.3 (s, 2C, Mes–C_{meta}), 130.1 (s, 2C, Mes–C_{meta}), 129.9 (s, 2C, Mes–C_{meta}), 129.8 (s, 2C, Mes–C_{meta}), 129.7 (s, 2C, Mes–C_{meta}), 128.6 (s, 2C, Mes–C_{meta}), 63.0 (s, 2C, O(CH$_2$)CH$_3$), 24.8 (s, 2C, *ortho*–Mes–CH$_3$), 24.1 (s, 2C, *ortho*–Mes–CH$_3$), 22.8 (s, 2C, *ortho*–Mes–CH$_3$), 22.7 (s, 2C, *ortho*–Mes–CH$_3$), 22.3 (s, 2C, *ortho*–Mes–CH$_3$), 21.9 (s, 2C, *ortho*–Mes–CH$_3$), 20.9 (s, 2C, *para*–Mes–CH$_3$), 20.8 (s, 2C, *para*–Mes–CH$_3$), 20.7 (s, 2C, *para*–Mes–CH$_3$), 18.8 (s, 2C, O(CH$_2$)CH$_3$), 5.3 (s, 6C, Si(CH$_3$)$_3$), 4.4 (s, 6C, Si(CH$_3$)$_3$), 3.6 (s, 6C, Si(CH$_3$)$_3$).

^{29}Si{^{1}H} IG NMR (CDCl$_3$, 300 K, 79.49 MHz): δ (ppm) = 17.9 (s, 2Si, *Si*), 9.1 (s, 2Si, (H$_3$C)$_3$*Si*), 7.5 (s, 2Si, (H$_3$C)$_3$*Si*), 7.0 (s, 2Si, (H$_3$C)$_3$*Si*), −0.7 (s, 2Si, *Si*), −60.1 (s, 2Si, *Si*), −140.3 (s, 2 Si, *Si*).

^{29}Si{^{1}H} DEPT-19.5 NMR (CDCl$_3$, 300K, 79.49 MHz): δ (ppm) = 9.1 (s, 2 Si, (H$_3$C)$_3$*Si*), 7.5 (s, 2 Si, (H$_3$C)$_3$*Si*), 7.0 (s, 2 Si, (H$_3$C)$_3$*Si*).

^{1}H,^{15}N HMBC NMR (CDCl$_3$, 300K, 400.1MHz, 40.55 MHz): δ (ppm) = 54 (s, Mes–*N*–Si(CH$_3$)$_3$), 65 (s, Mes–*N*–Si(CH$_3$)$_3$), 69 (s, Mes–*N*–Si(CH$_3$)$_3$).

IR (KBr-pellet): $\tilde{\nu}$ (cm^{-1}) = 2956 m, 2910 m, 2852 s, 2733 s, 2417 vs, 2400 s, 2356 vs, 2082 vs, 2027 vs, 1961 vs, 1943 vs, 1894 s, 1825 s, 1745 s, 1684 s, 1671 s, 1645 s, 1636 s, 1582 s, 1566 s, 1541 s, 1475 w, 1372 m, 1348 s, 1295 s, 1250 w, 1212 m, 1198 w, 1148 w, 1136 m, 1109 w, 1084 w, 959 m, 901 vw, 865 vw, 782 m, 764 m, 725 w, 687 w, 635 vw.

UV-vis: (c = 0.064 mmol/L, 0.013 mmol/L in THF) λ_{max} = 315 nm, ε = 8556 L·mol^{-1}·cm^{-1}.

ESI-MS (positive mode, solution in THF): m/z = [M]$^+$ 1550.6965 (1550.7020 calc.).

CHN for $C_{76}H_{130}N_6O_2Si_{14}$: calc.: C 58.77 %, H 8.44 %, N 5.41 %; found: C 57.85 %, H 8.25 %, N 5.27 %.

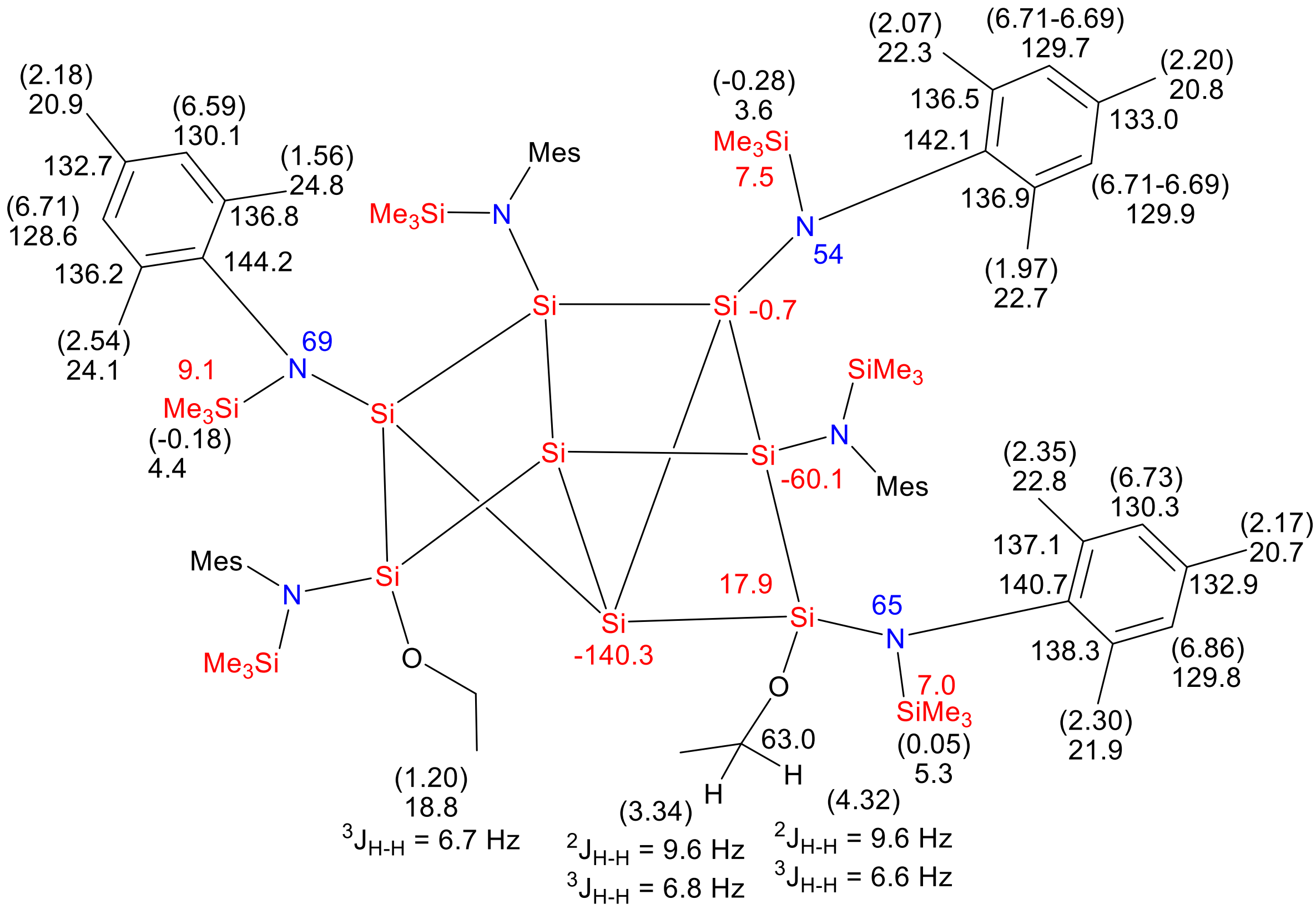

Figure S84. Assignment of chemical shifts to **5** (in $CDCl_3$ at 300 K). Color code: ^{1}H black in brackets, ^{13}C black, ^{15}N blue, and ^{29}Si red.

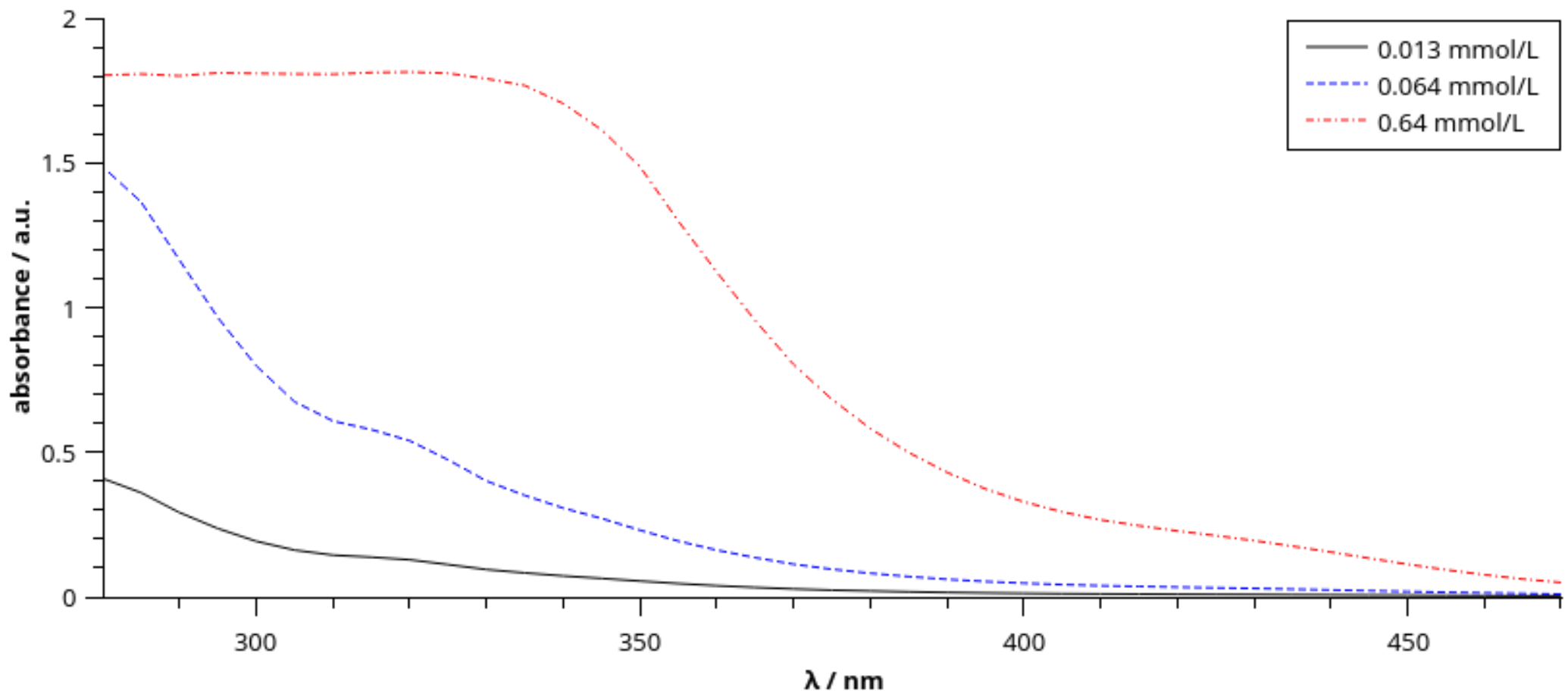

Figure S85. UV-vis spectrum of **5** in THF.

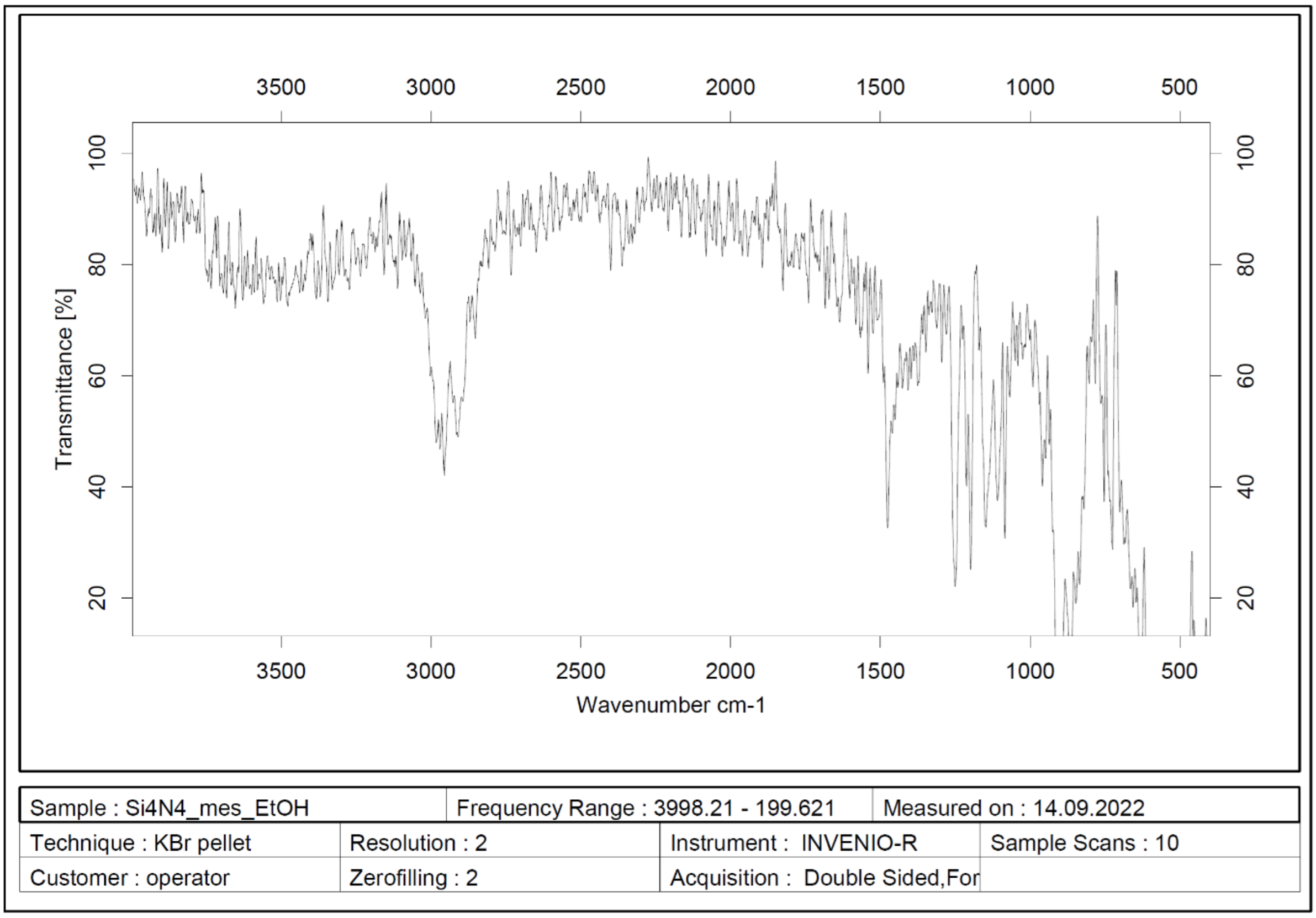

Figure S86. FT-IR spectrum of **5** (KBr-pellet).

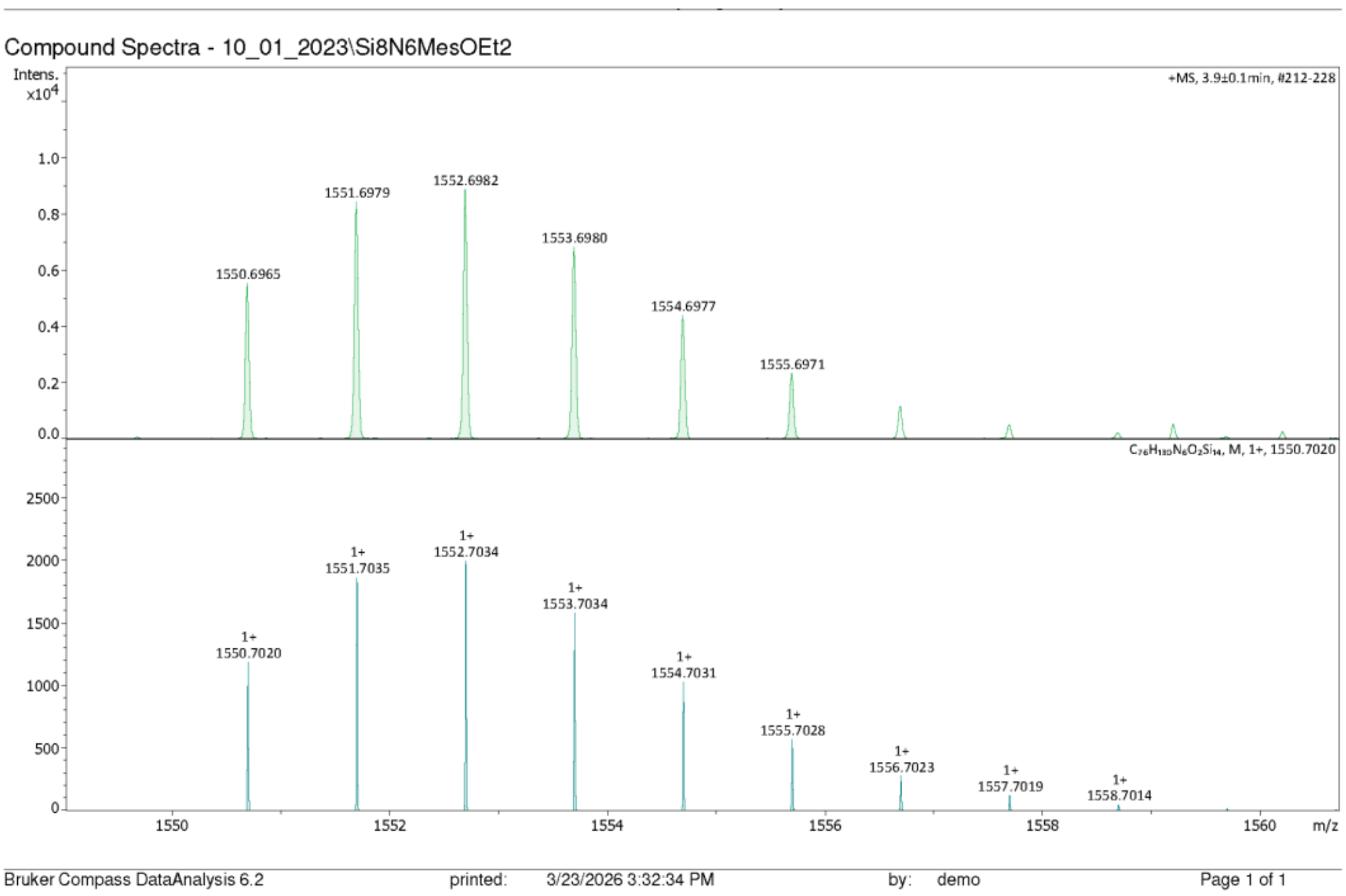

Figure S87. ESI-MS spectrum (positive mode) of **5**.

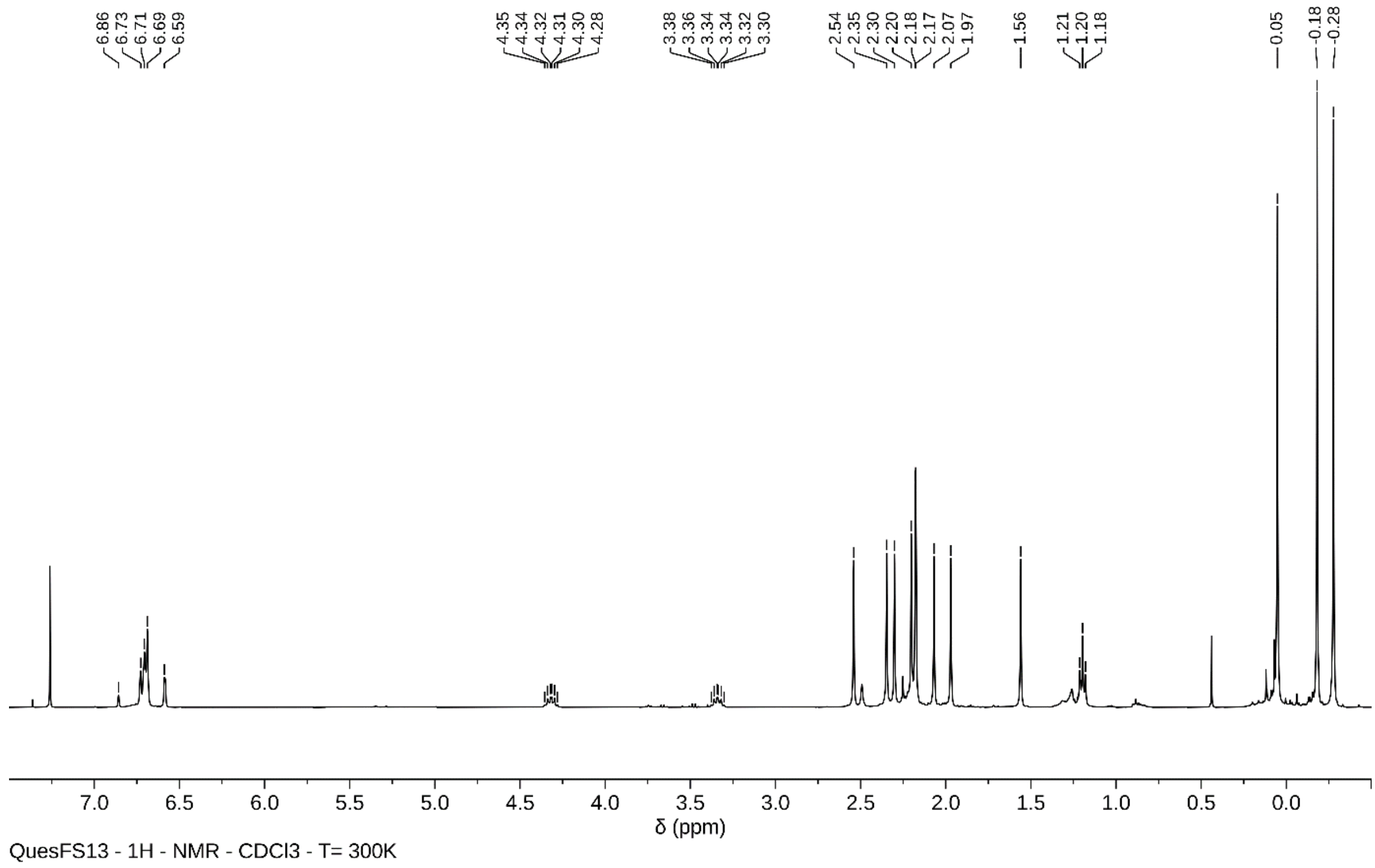

Figure S88. ^{1}H NMR spectrum of **5** in $CDCl_3$ at 300 K and 400 MHz.

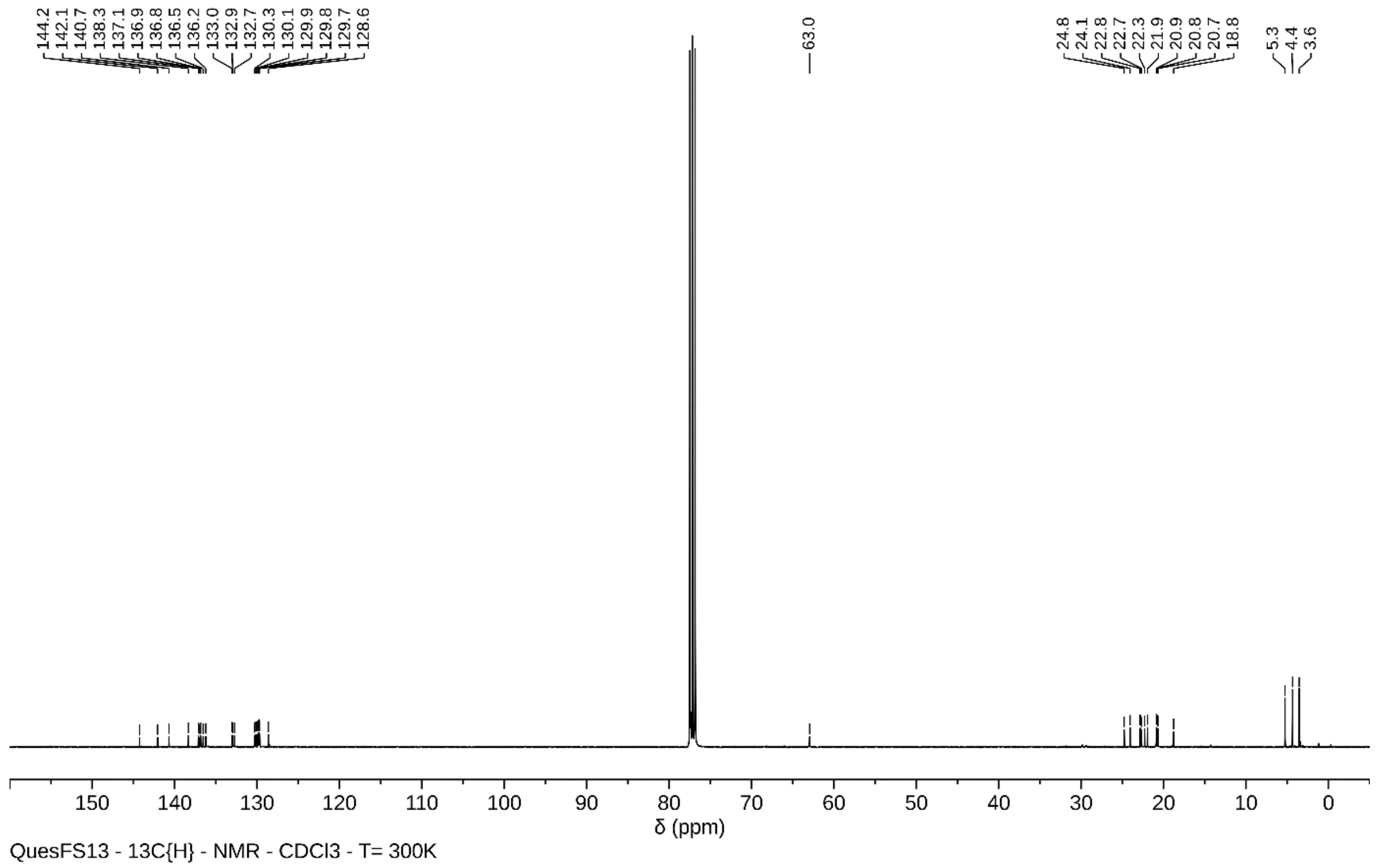

Figure S89. $^{13}C\{^{1}H\}$ NMR spectrum of **5** in $CDCl_3$ at 300 K and 100 MHz.

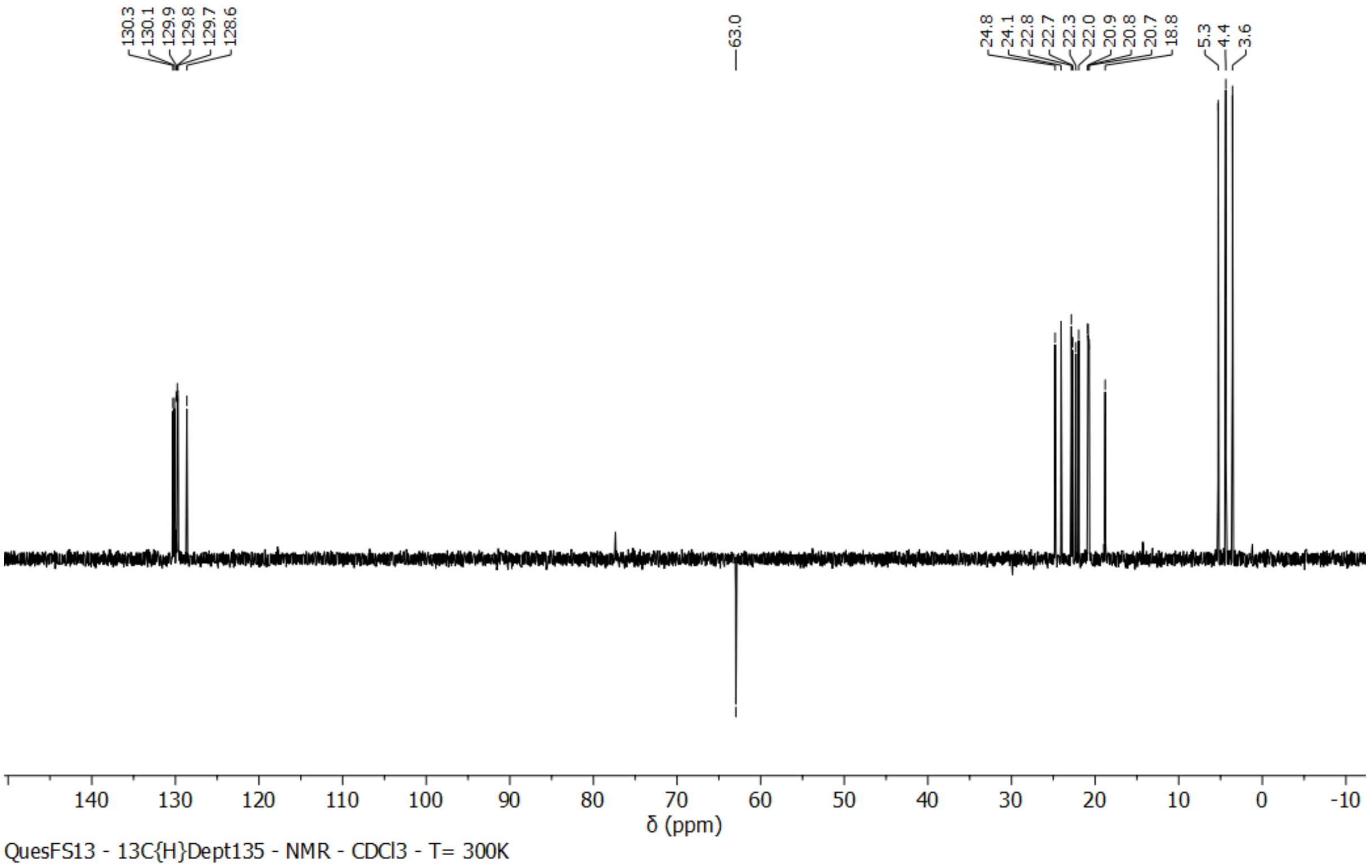

Figure S90. $^{13}C\{^{1}H\}$ DEPT-135 NMR spectrum of **5** in $CDCl_3$ at 300 K and 100 MHz.

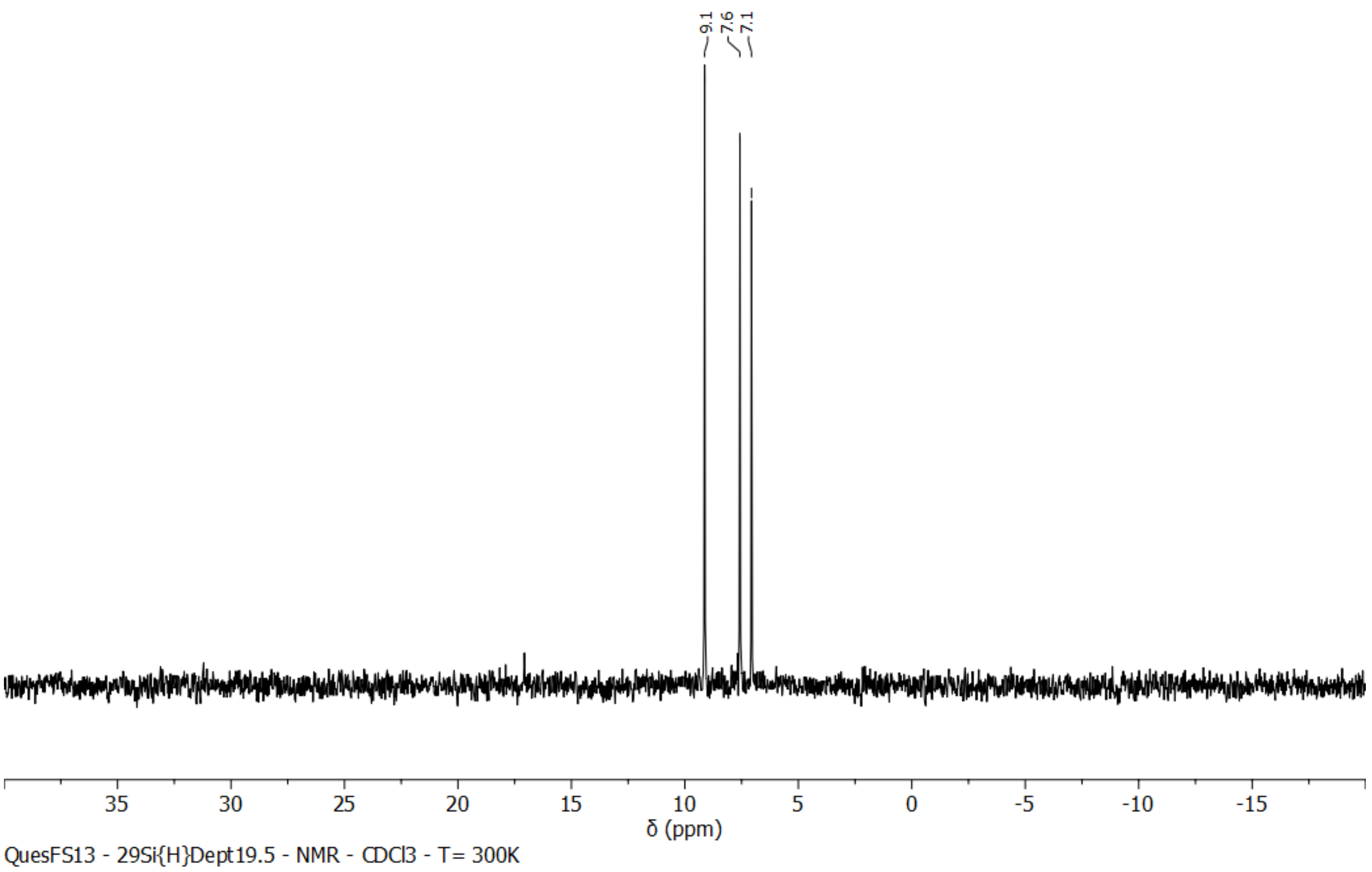

Figure S91. $^{29}Si\{^{1}H\}$ DEPT-19.5 NMR spectrum of **5** in $CDCl_3$ at 300 K and 79 MHz.

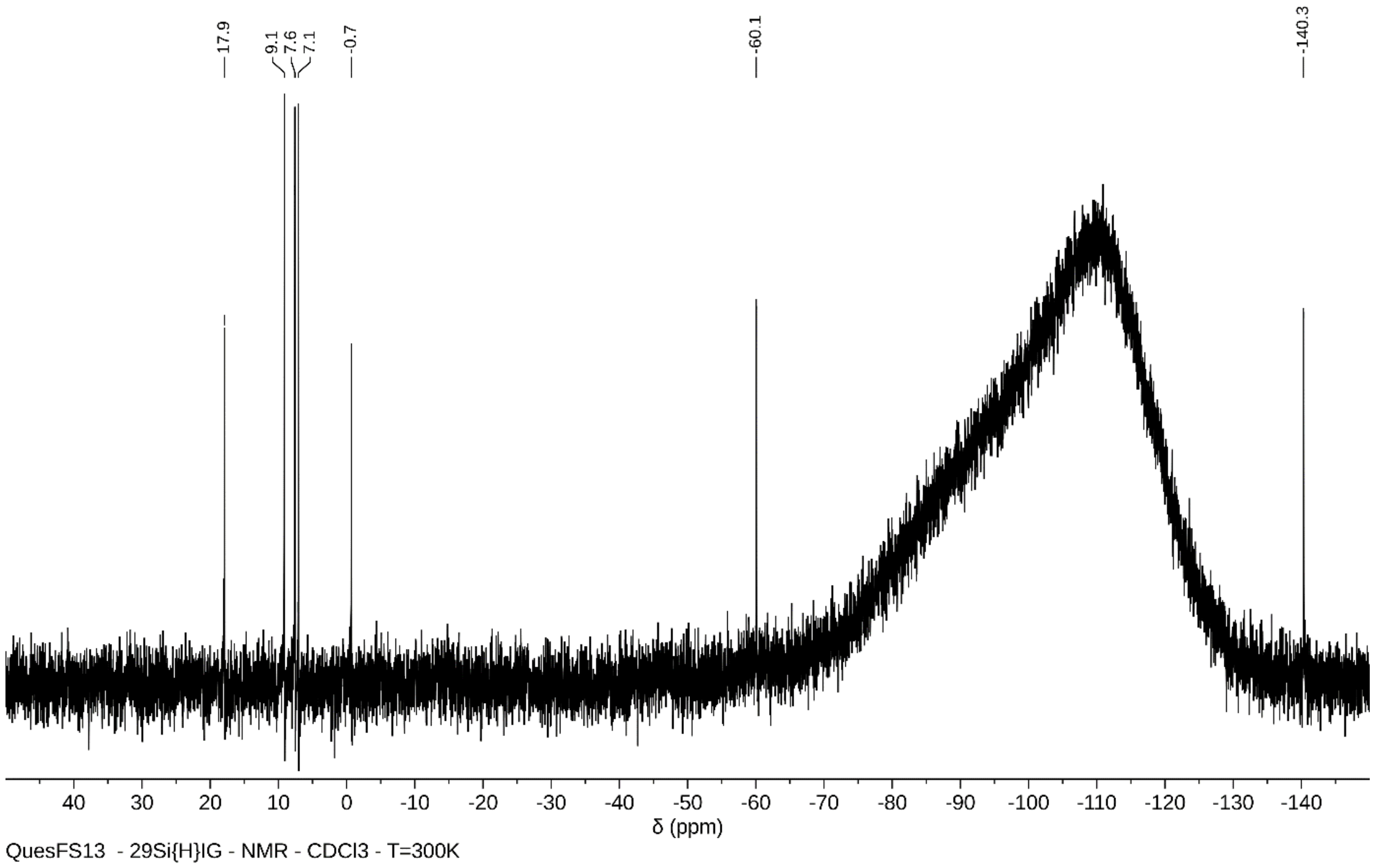

Figure S92. $^{29}Si\{^{1}H\}$ IG NMR spectrum of **5** in $CDCl_3$ at 300 K and 79 MHz.

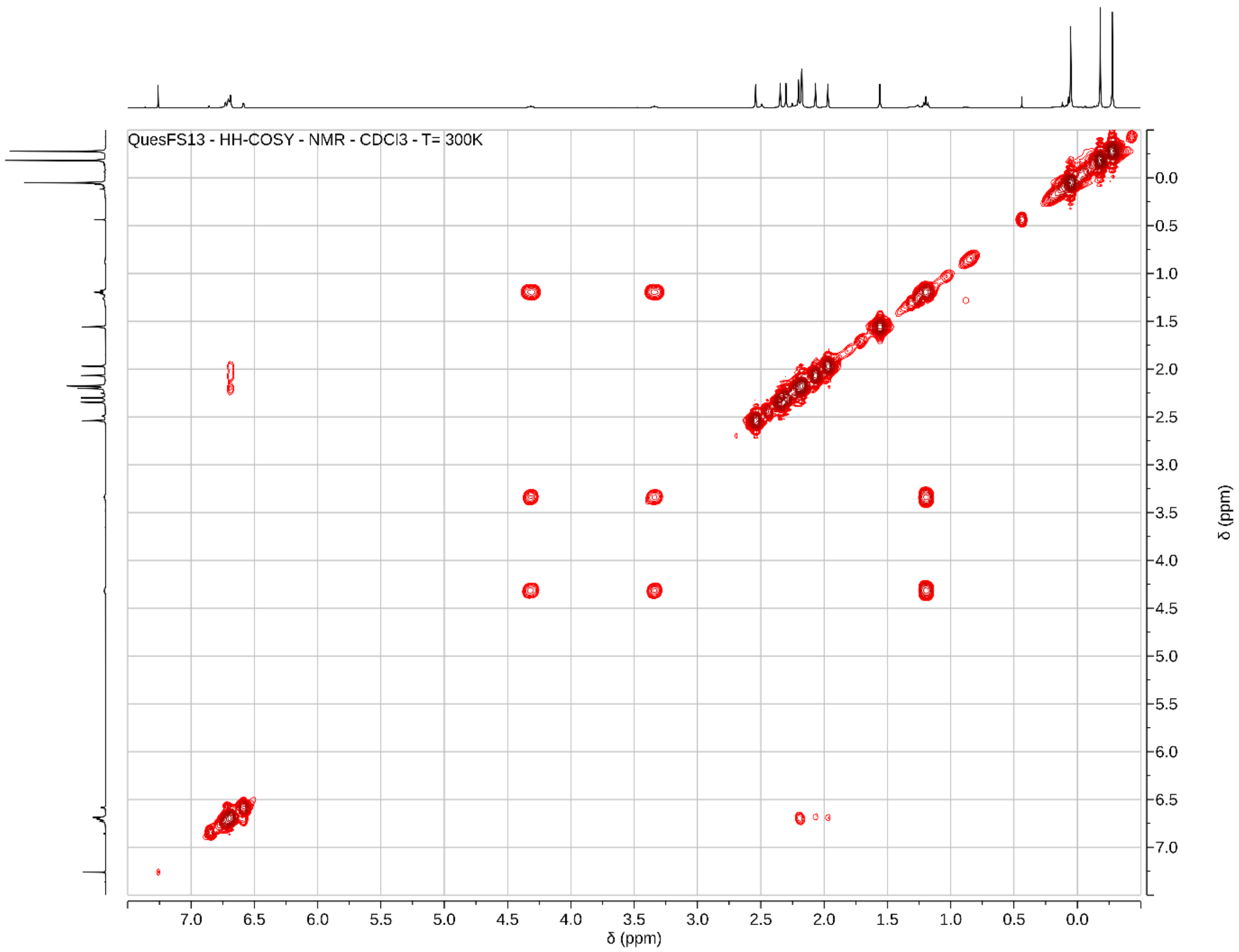

Figure S93. $^{1}H,^{1}H$ COSY NMR spectrum of **5** in $CDCl_3$ at 300 K and 400 MHz.

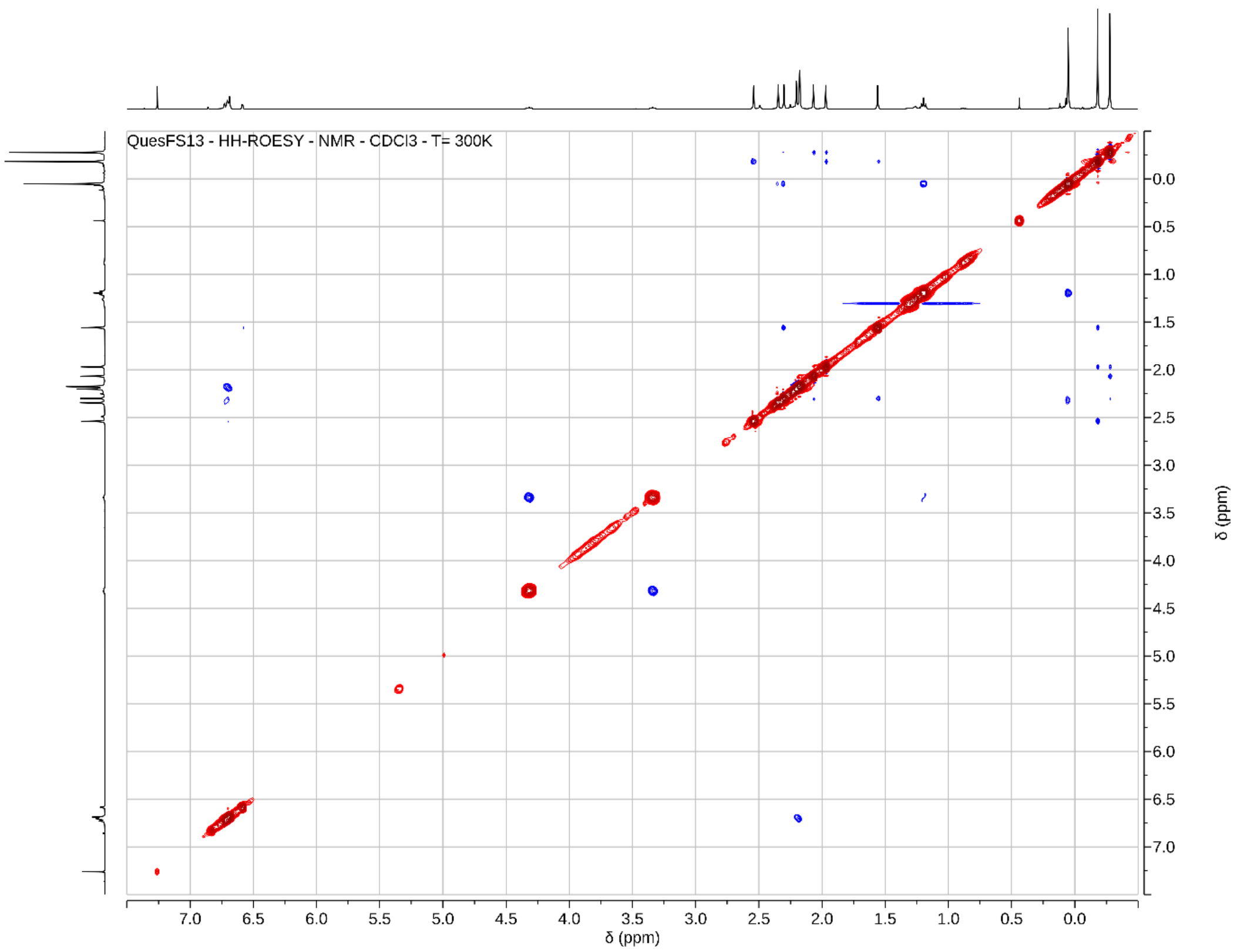

Figure S94. $^{1}H,^{1}H$ ROESY NMR spectrum of **5** in $CDCl_3$ at 300 K and 400 MHz.

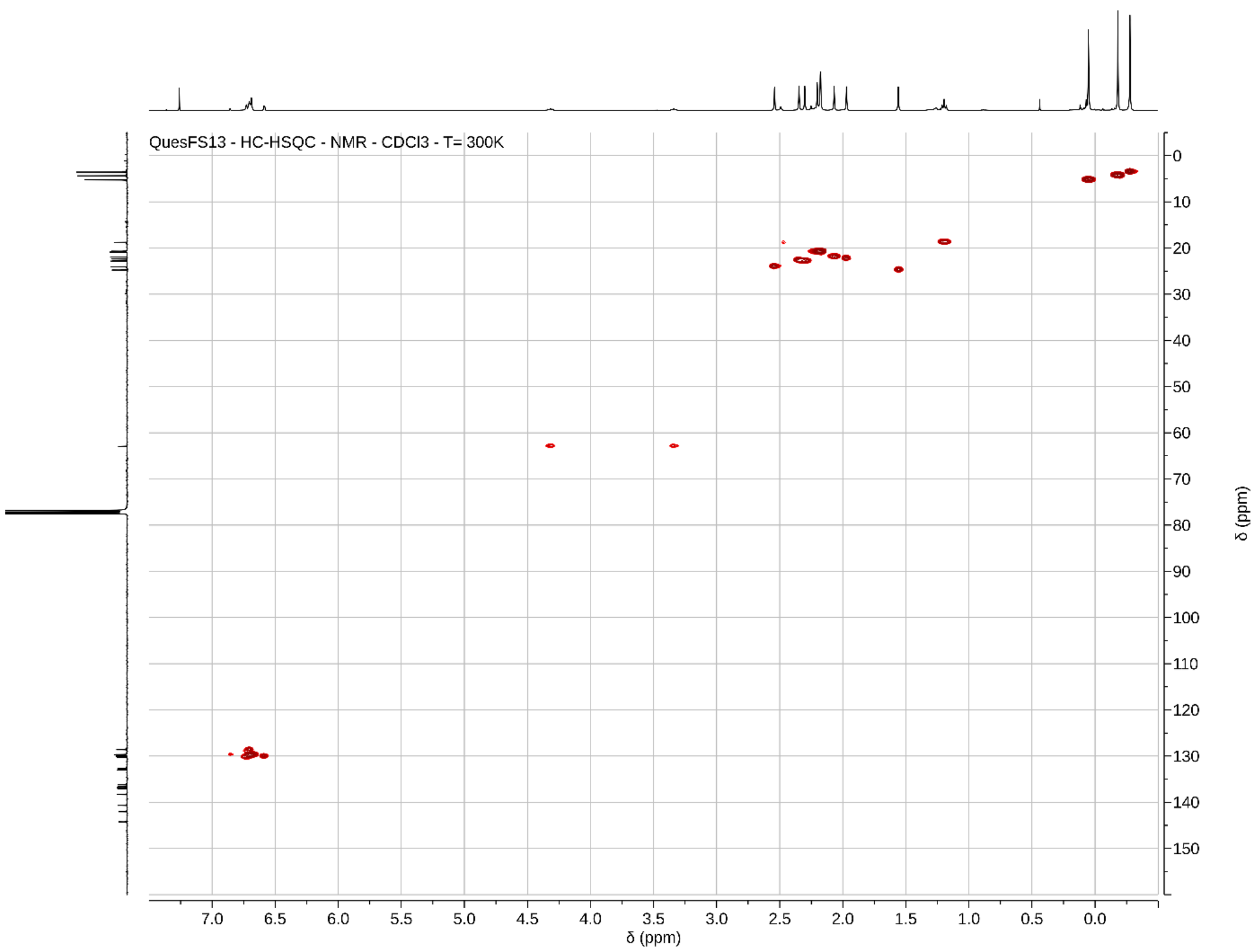

Figure S95. $^{1}H,^{13}C$ HSQC NMR spectrum of **5** in $CDCl_3$ at 300 K and 400 MHz (^{1}H) and 100 MHz (^{13}C).

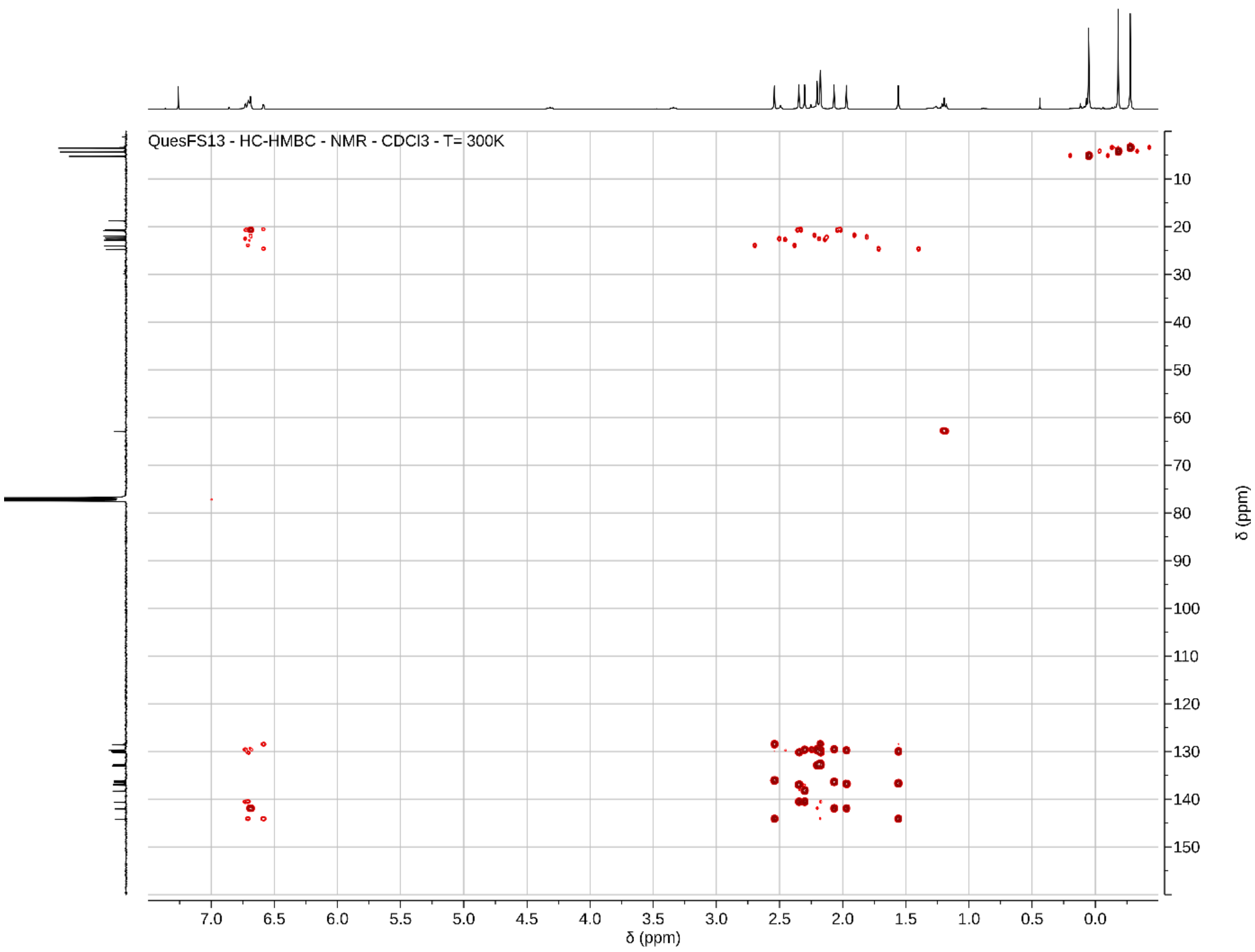

Figure S96. $^{1}H,^{13}C$ HMBC NMR spectrum of **5** in $CDCl_3$ at 300 K and 400 MHz (^{1}H) and 100 MHz (^{13}C).

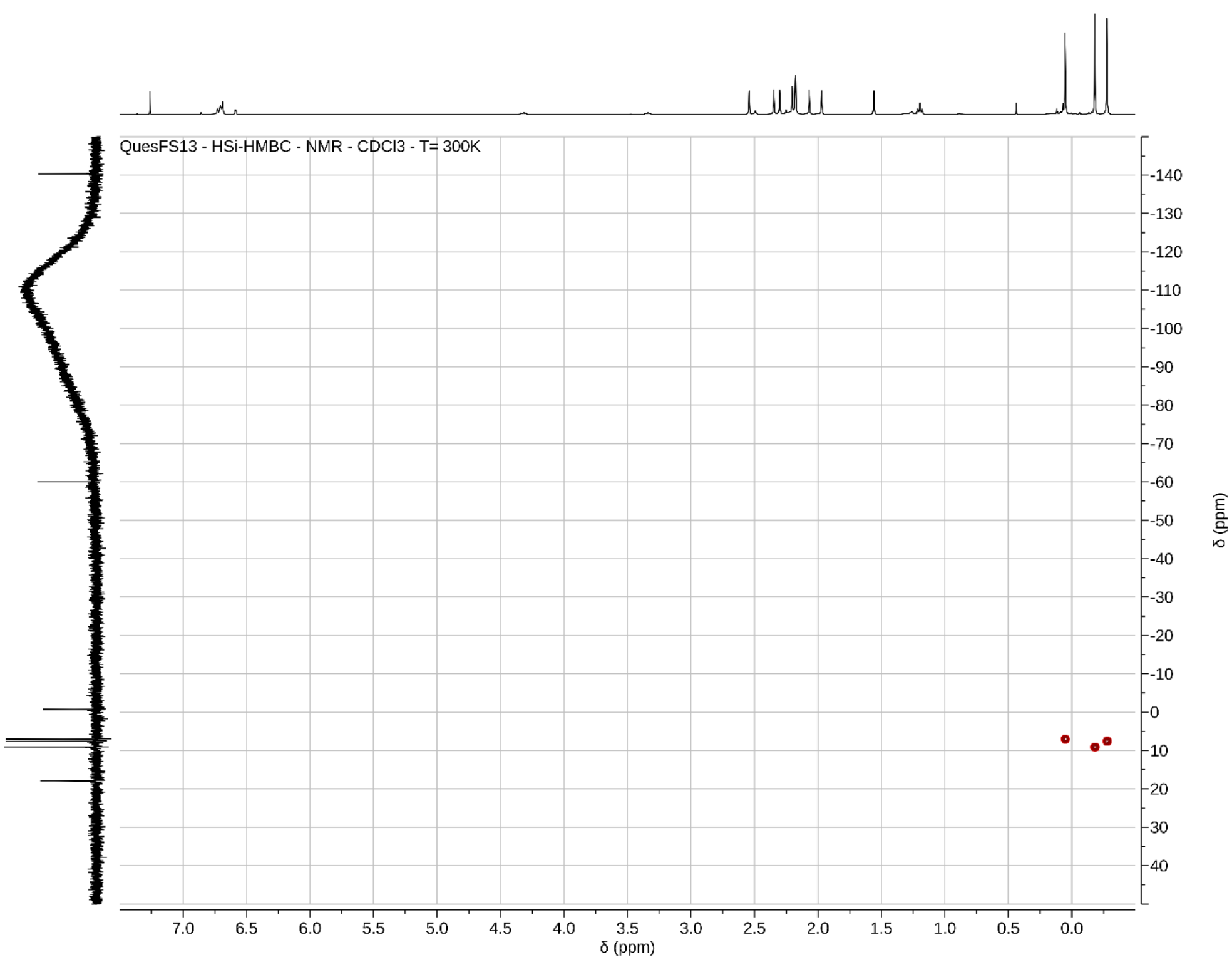

Figure S97. $^{1}H,^{29}Si$ HMBC NMR spectrum of **5** in $CDCl_3$ at 300 K and 400 MHz (^{1}H) and 79 MHz (^{29}Si).

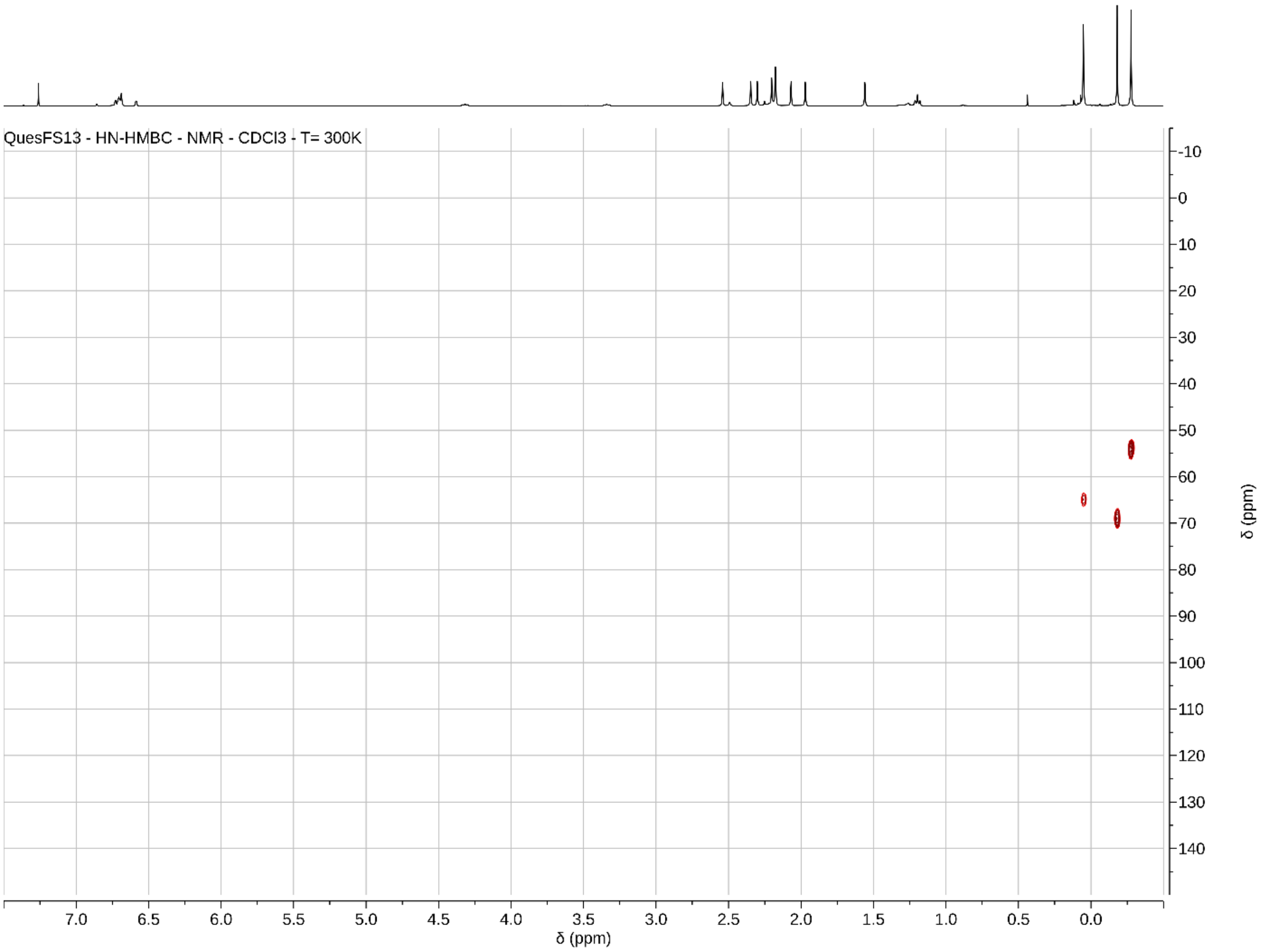

Figure S98. $^{1}H,^{15}N$ HMBC NMR spectrum of **5** in $CDCl_3$ at 300 K and 400 MHz (^{1}H) and 40 MHz (^{15}N).

2.8 $H(OPh)Si_4\{N(SiMe_3)Mes\}_4$ (6) and $(H)(OPh)Si_8\{N(SiMe_3)Mes\}_6$ (7)

Phenol (107.0 mg, 1.14 mmol, 2.0 eq., 2.27 mL solution in THF 0.5 M) was added dropwise to a cooled (−89 °C) solution of $Si_4\{N(SiMe_3)Mes\}_4$ **F** (533.0 mg, 0.568 mmol, 1.0 eq.) under stirring in toluene (15 mL) using an *iso*-propanol liquid nitrogen cooling bath. The solution was allowed to warm to room temperature and was stirred for 16 h. A change in color from red to yellow was observed at a temperature of approx. 0 °C. All volatiles were removed *in vacuo* to yield yellow foam. The residue was extracted with 3×0.5 mL *n*-pentane and filtered using a filter cannula. After filtration all volatiles were removed. The product $H(OPh)Si_4\{N(SiMe_3)Mes\}_4$ (**6**) was obtained as a yellow solid together with a minor component (see below). A few single crystals of $(H)(OPh)Si_8\{N(SiMe3)Mes\}_6$ (**7**) could also be obtained as a DME solvate. Complete characterization of **6** and **7** proved impossible. Compound **6** was characterized by NMR spectroscopy and ESI-MS, whereas the structure of **7**·DME was determined by single crystal X-ray diffraction (see below).

Data for 6:

^{1}H NMR (C_6D_6, 300 K, 400.1 MHz): δ (ppm) = 7.01 (m, 2H, *meta*–OPh–*H*), 6.83–6.81 (m, 2H, *ortho*–OPh–*H*), 6.79 (s o.l., 1H, *para*–OPh–*H*), 6.78 (s, 2H, *meta*-Ar–C*H*), 6.63 (s, 2H, *meta*-Ar–C*H*), 6.59 (s, 2H, *meta*-Ar–C*H*), 6.40 (s, 2H, *meta*-Ar–C*H*), 5.60 (s, 1H, Si*H*, $^{1}J_{H,Si}$ = 228 Hz, $^{3}J_{H,Si}$ = 43.8 Hz), 2.51 (s, 6H, *ortho*–Ar–CH_3), 2.51 (s, 6H, *ortho*–Ar–CH_3), 2.26 (s, 6H, *ortho*–Ar–CH_3), 2.15 (s, 6H, *para*–Ar–CH_3), 2.07 (s, 3H, *para*–Ar–CH_3), 2.00 (s, 3H, *para*–Ar–CH_3), 1.82 (s, 6H, *ortho*–Ar–CH_3), 0.32 (s, 18H, Si(CH_3)$_3$), 0.30 (s, 9H, Si(CH_3)$_3$), −0.16 (s, 9H, Si(CH_3)$_3$).

^{13}C{^{1}H} NMR (C_6D_6, 300 K, 100.6 MHz): δ (ppm) = 155.1 (s, 1C, *ipso*-OPh–*C*), 144.1 (s, 2C, *ipso*-Ar–*C*), 140.5 (s, 1C, *ipso*-Ar–*C*), 139.8 (s, 1C, *ipso*-Ar–*C*), 137.2 (s, 2C, *ortho*-Ar–*C*), 137.0 (s, 2C, *ortho*-Ar–*C*), 135.8 (s, 2C, *ortho*-Ar–*C*), 135.2 (s, 2C, *ortho*-Ar–*C*), 133.4 (s, 2C, *para*-Ar–*C*), 133.3 (s, 1C, *para*-Ar–*C*), 132.9 (s, 1C, *para*-Ar–*C*), 129.9 (s, 2C, *meta*-Ar–*C*), 129.7 (s, 2C, *meta*-Ar–*C*), 129.6 (s, 2C, *meta*-Ar–*C*), 129.3 (s, 2C, *meta*-Ar–*C*), 128.3 (s, 2C, *meta*-OPh–*C*), 120.9 (s, 1C, *para*-OPh–*C*), 119.8 (s, 2C, *ortho*-OPh–*C*), 21.7 (s, 2C, *ortho*–Ar–*C*H_3), 21.6 (s, 2C, *ortho*–Ar–*C*H_3), 21.5 (s, 2C, *ortho*–Ar–*C*H_3), 21.4 (s, 2C, *ortho*–Ar–*C*H_3), 21.1 (s, 1C, *para*–Ar–*C*H_3), 20.9 (s ol, 1C, *para*–Ar–*C*H_3), 20.9 (s o.l., 2C, *para*–Ar–*C*H_3), 2.9 (s, 3C, Si(*C*H_3)$_3$), 2.7 (s, 6C, Si(*C*H_3)$_3$), 1.8 (s, 3C, Si(*C*H_3)$_3$).

^{13}C{^{1}H} DEPT-135 NMR (C_6D_6, 300 K, 100.6 MHz): δ (ppm) = 129.9 (s, 2C, *meta*-Ar–*C*), 129.7 (s, 2C, *meta*-Ar–*C*), 129.6 (s, 2C, *meta*-Ar–*C*), 129.3 (s, 2C, *meta*-Ar–*C*), 128.3 (s, 2C, *meta*-OPh–*C*), 120.9 (s, 1C, *para*-OPh–*C*), 119.8 (s, 2C, *ortho*-OPh–*C*), 21.7 (s, 2C, *ortho*–Ar–*C*H_3), 21.6 (s, 2C, *ortho*–Ar–*C*H_3), 21.5 (s, 2C, *ortho*–Ar–*C*H_3), 21.4 (s, 2C, *ortho*–Ar–*C*H_3), 21.1 (s, 1C, *para*–Ar–*C*H_3), 20.9 (s, 1C, *para*–Ar–*C*H_3), 20.9 (s, 2C, *para*–Ar–*C*H_3), 2.8 (s, 3C, Si(*C*H_3)$_3$), 2.7 (s, 6C, Si(*C*H_3)$_3$), 1.8 (s, 3C, Si(*C*H_3)$_3$).

^{29}Si{^{1}H} IG NMR (C_6D_6, 300K, 79.49 MHz): δ (ppm) = 43.3 (s, 2 Si, *Si*), 10.0 (s, 2 Si, (H_3C)$_3$*Si*), 7.6 (s, 1 Si, (H_3C)$_3$*Si*), 6.9 (s, 1 Si, (H_3C)$_3$*Si*), −30.4 (s, 1 Si, *Si*), −100.7 (s, 1 Si, H*Si*).

^{29}Si{^{1}H} DEPT-19.5 NMR (C_6D_6, 300K, 79.49 MHz): δ (ppm) = 10.0 (s, 2Si, $(H_3C)_3$*Si*), 7.6 (s, 1Si, $(H_3C)_3$*Si*), 6.9 (s, 1Si, $(H_3C)_3$*Si*).

^{29}Si{^{1}H} DEPT-90 NMR (C_6D_6, 300K, 79.49 MHz): δ (ppm) = −100.7 (s, 1 Si, H*Si*).

^{1}H,^{15}N HMBC NMR (C_6D_6, 300K, 400.1MHz, 40.55 MHz): δ (ppm) = 40 (s, Mes–*N*–Si$(CH_3)_3$), 54 (s, Mes–*N*–Si$(CH_3)_3$), 68 (s, Mes–*N*–Si$(CH_3)_3$).

ESI-MS (positive mode, solution in THF): *m*/*z* = $[M+H]^+$ 1031.5057 (1031.5029 calc.).

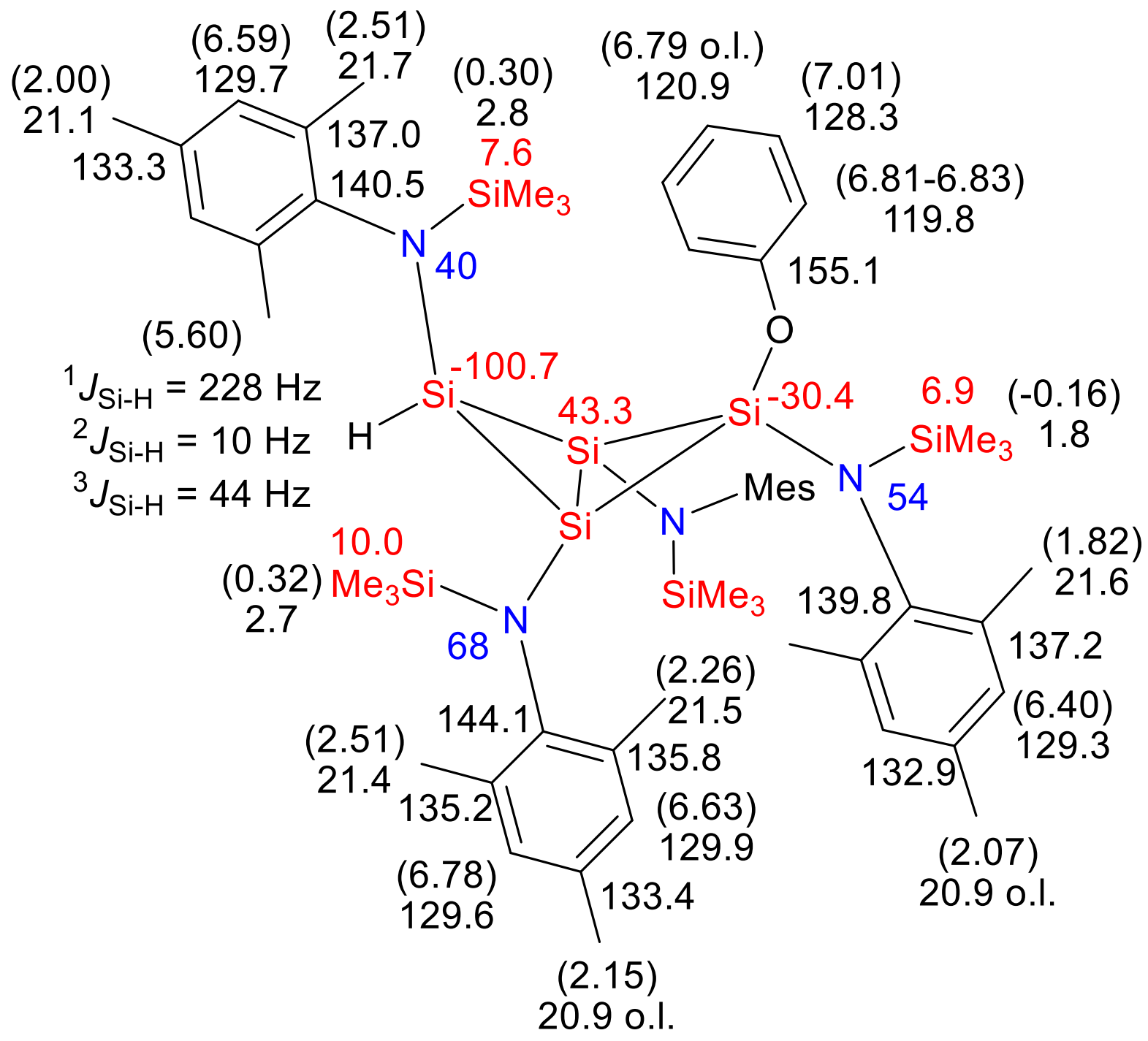

Figure S99. Assignment of chemical shifts to **6** (in C_6D_6 at 300 K). Color code: ^{1}H black in brackets, ^{13}C black, ^{15}N blue, and ^{29}Si red.

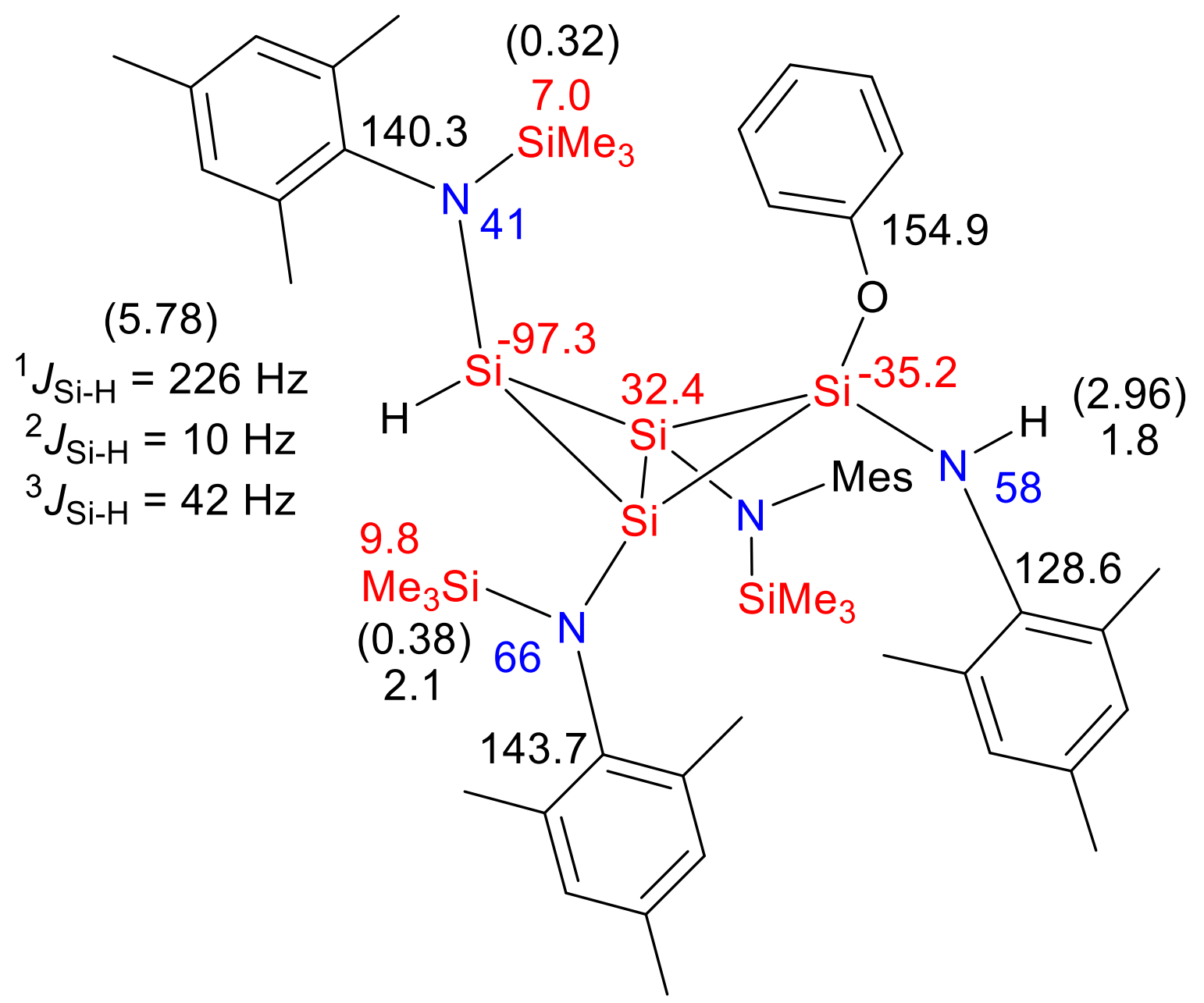

Figure S100. Assignment of chemical shifts to the minor component observed together with **6** (in C_6D_6 at 300 K). Color code: ^{1}H black in brackets, ^{13}C black, ^{15}N blue, and ^{29}Si red.

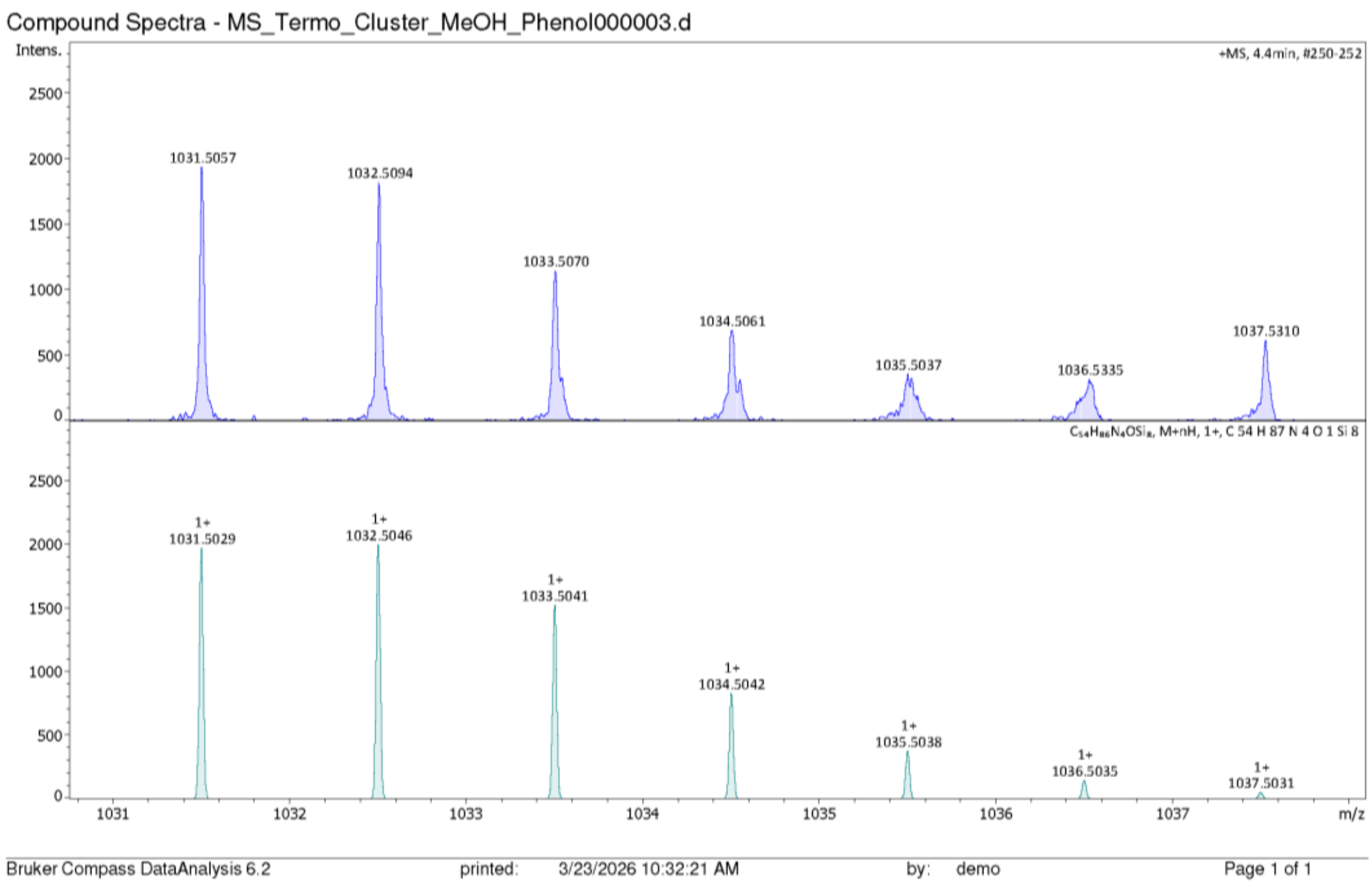

Figure S101. ESI-MS spectrum (positive mode) of **6**.

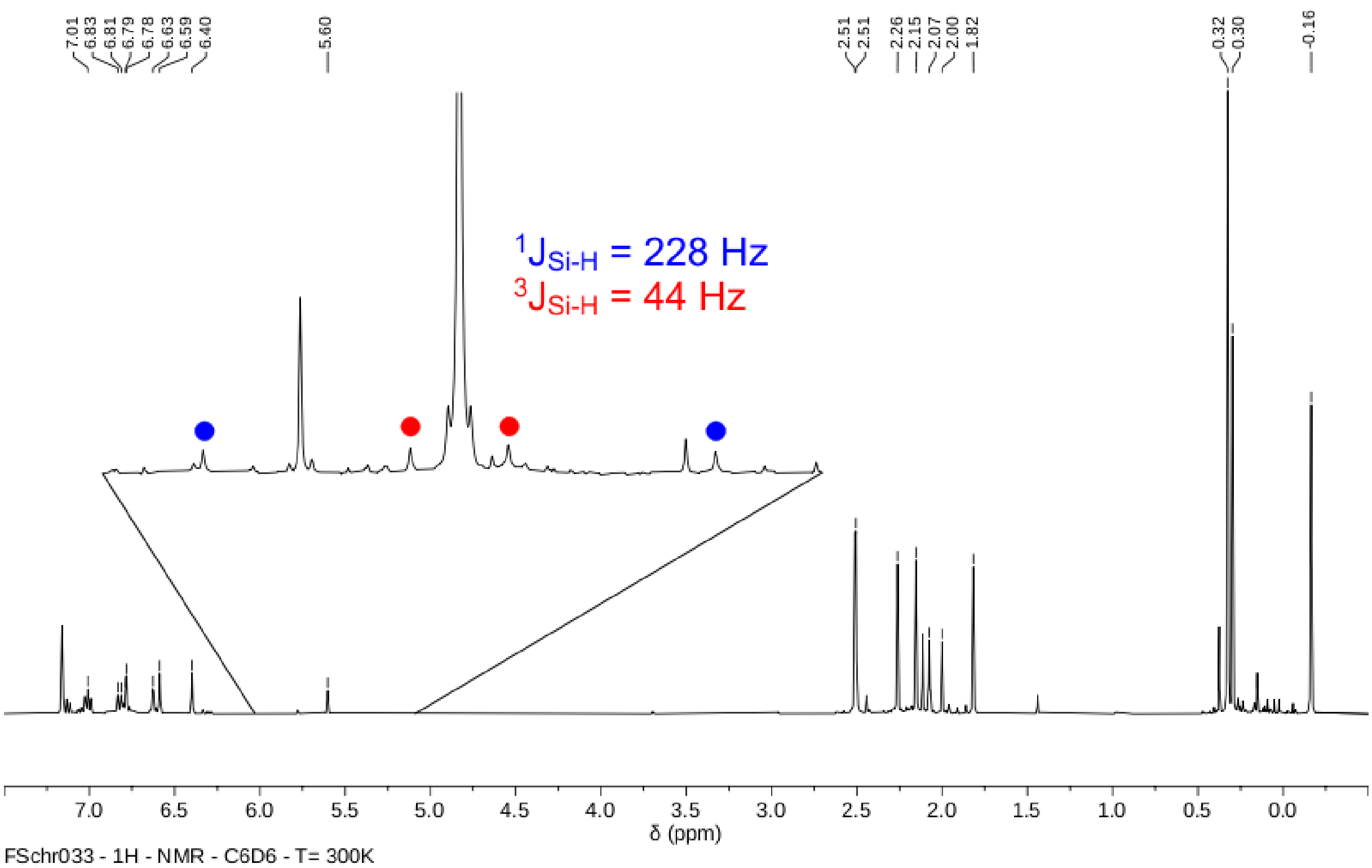

Figure S102. ^{1}H NMR spectrum of **6** in C_6D_6 at 300 K and 400 MHz.

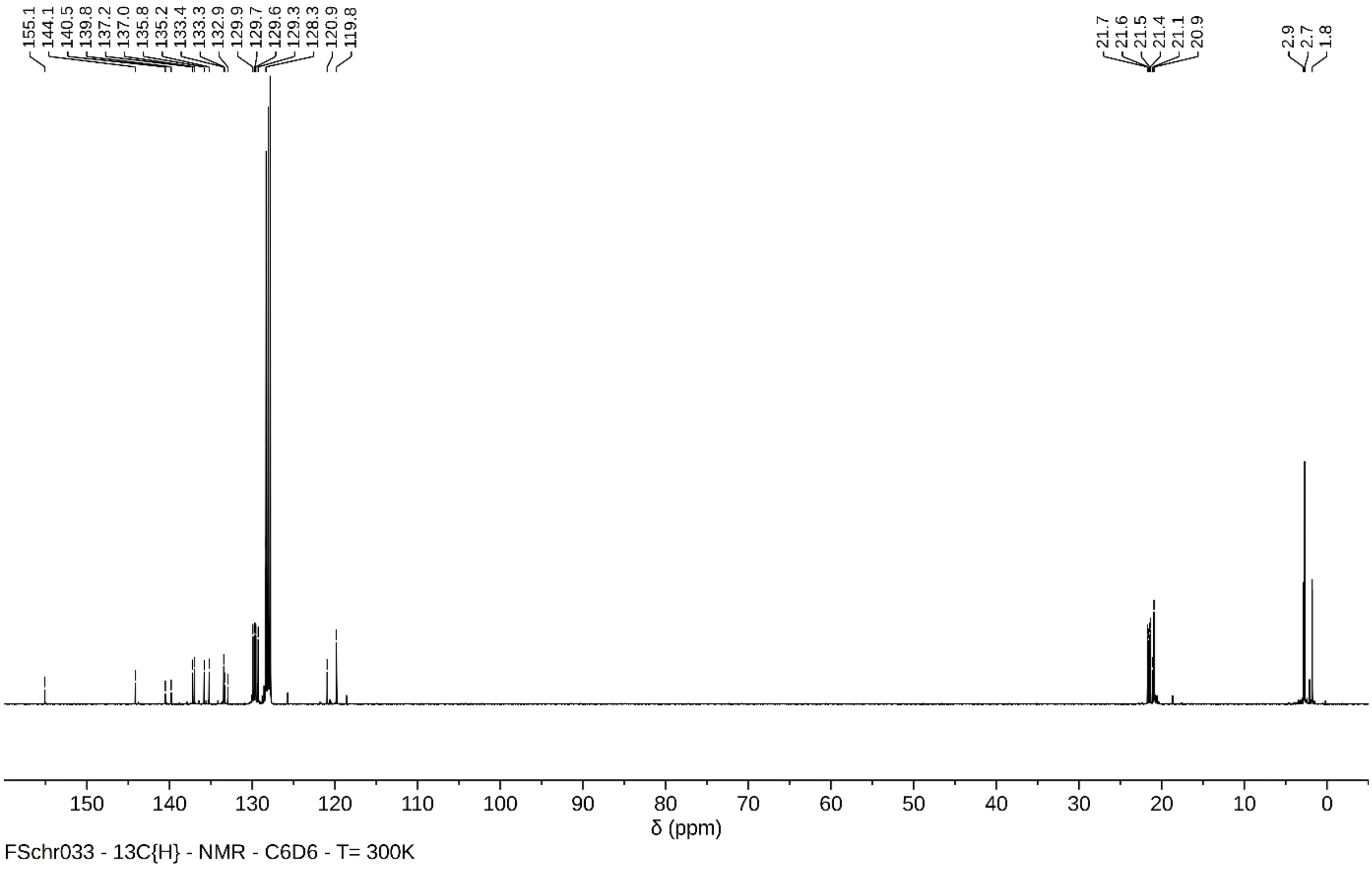

Figure S103. $^{13}C\{^{1}H\}$ NMR spectrum of **6** in C_6D_6 at 300 K and 100 MHz.

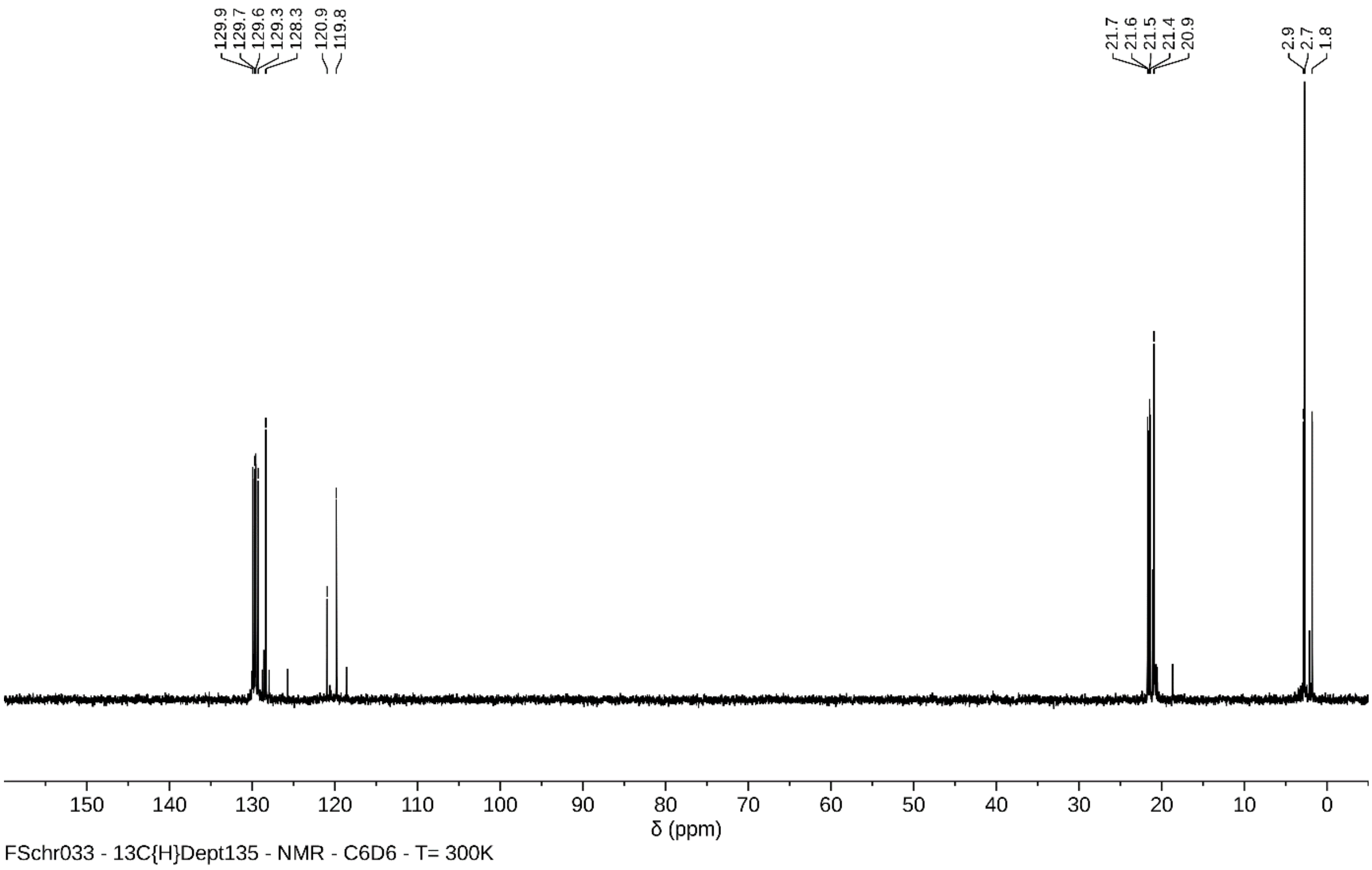

Figure S104. $^{13}C\{^{1}H\}$ DEPT-135 NMR spectrum of **6** in C_6D_6 at 300 K and 100 MHz.

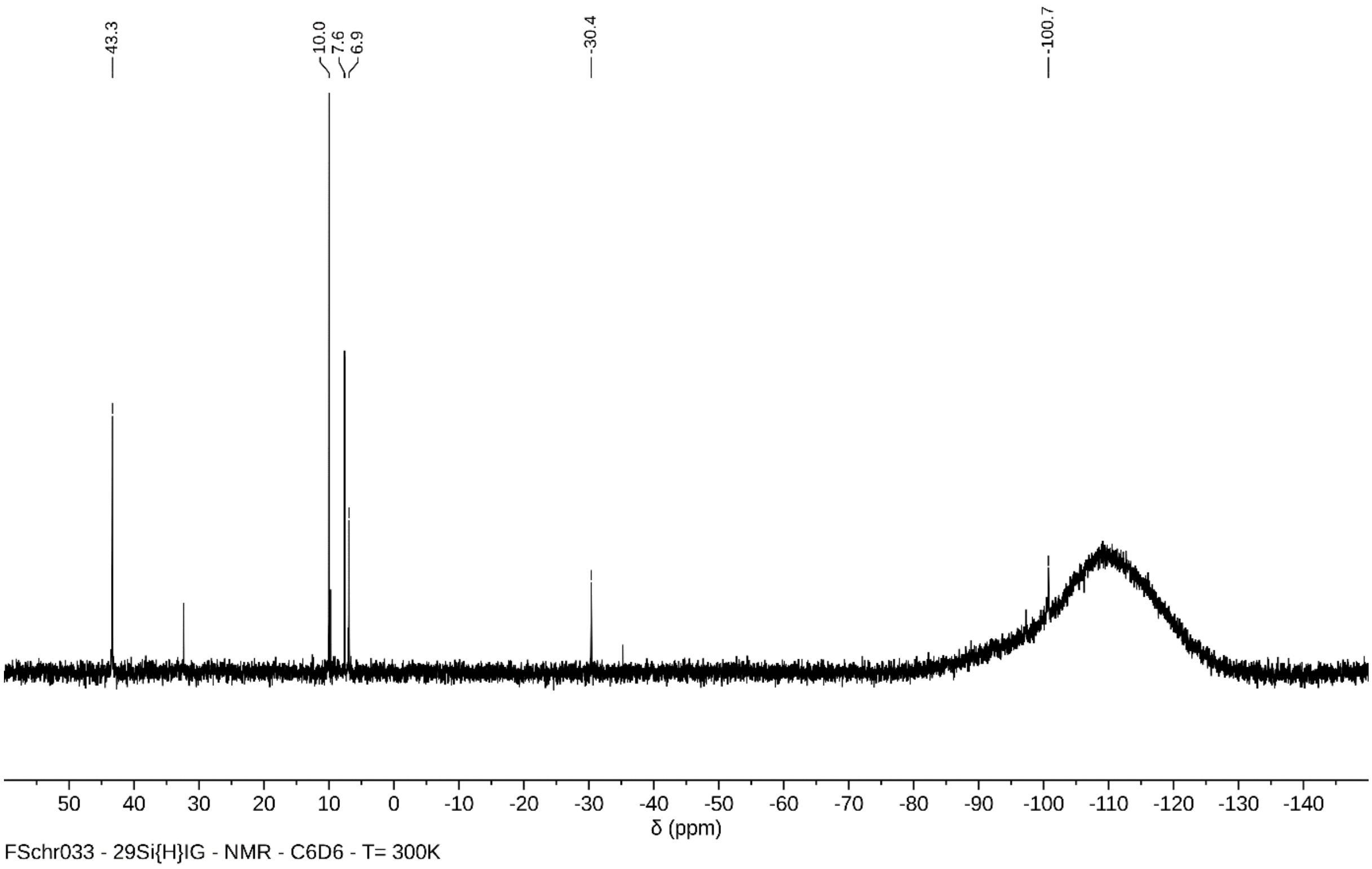

Figure S105. $^{29}Si\{^{1}H\}$ IG NMR spectrum of **6** in C_6D_6 at 300 K and 79 MHz.

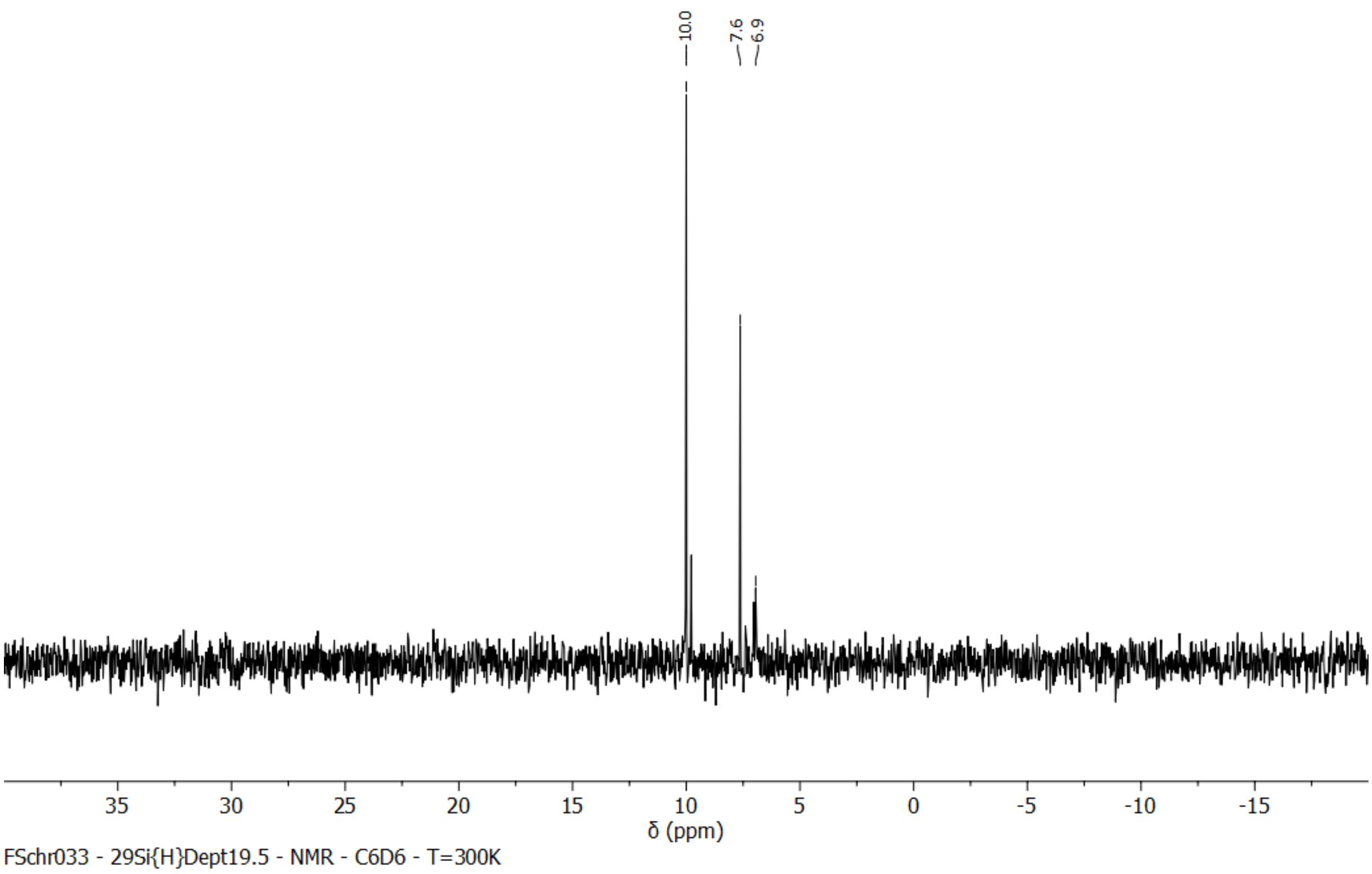

Figure S106. $^{29}Si\{^{1}H\}$ DEPT-19.5 NMR spectrum of **6** in C_6D_6 at 300 K and 79 MHz.

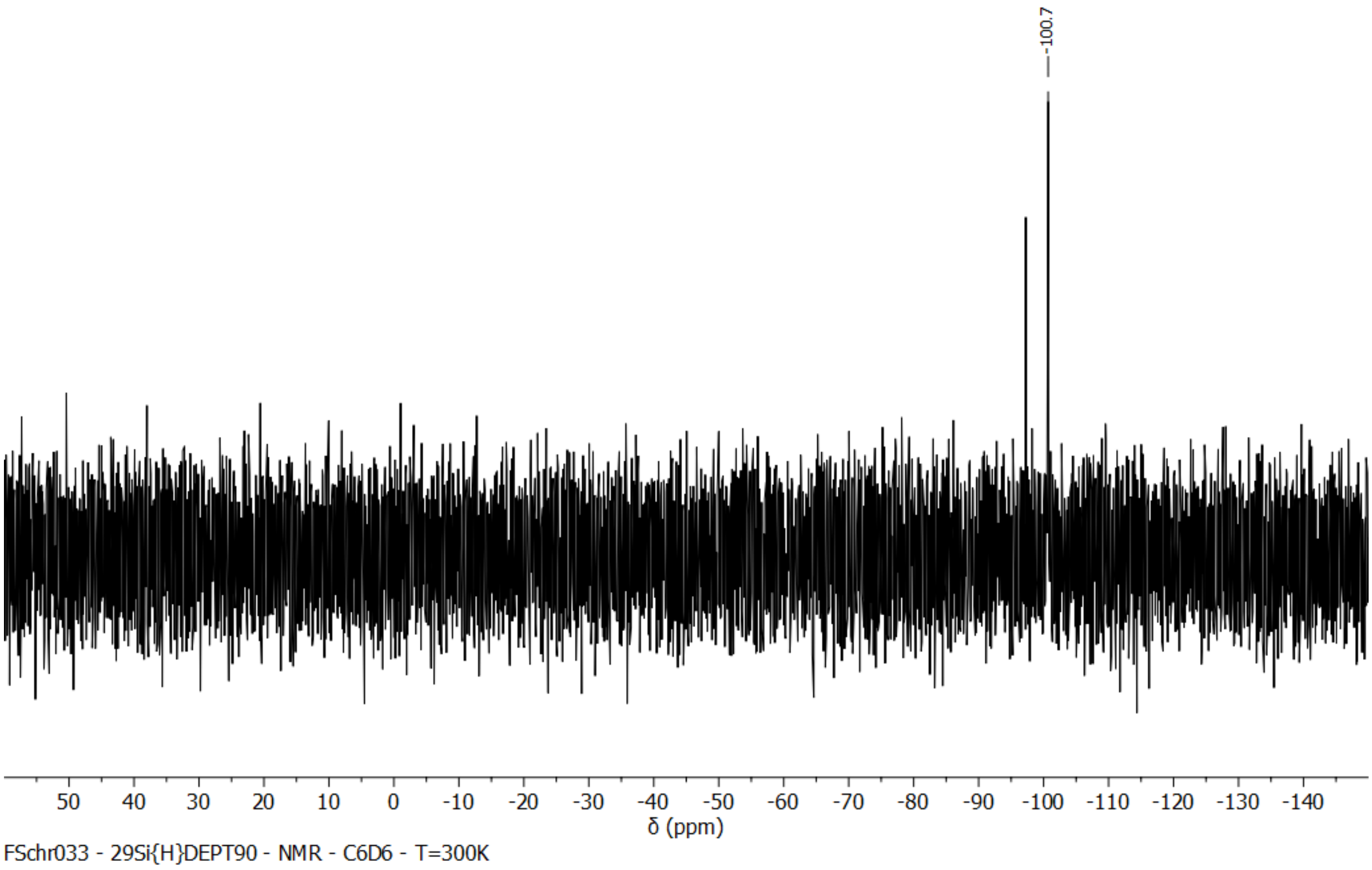

Figure S107. $^{29}Si\{^{1}H\}$ DEPT-90 NMR spectrum of **6** in C_6D_6 at 300 K and 79 MHz.

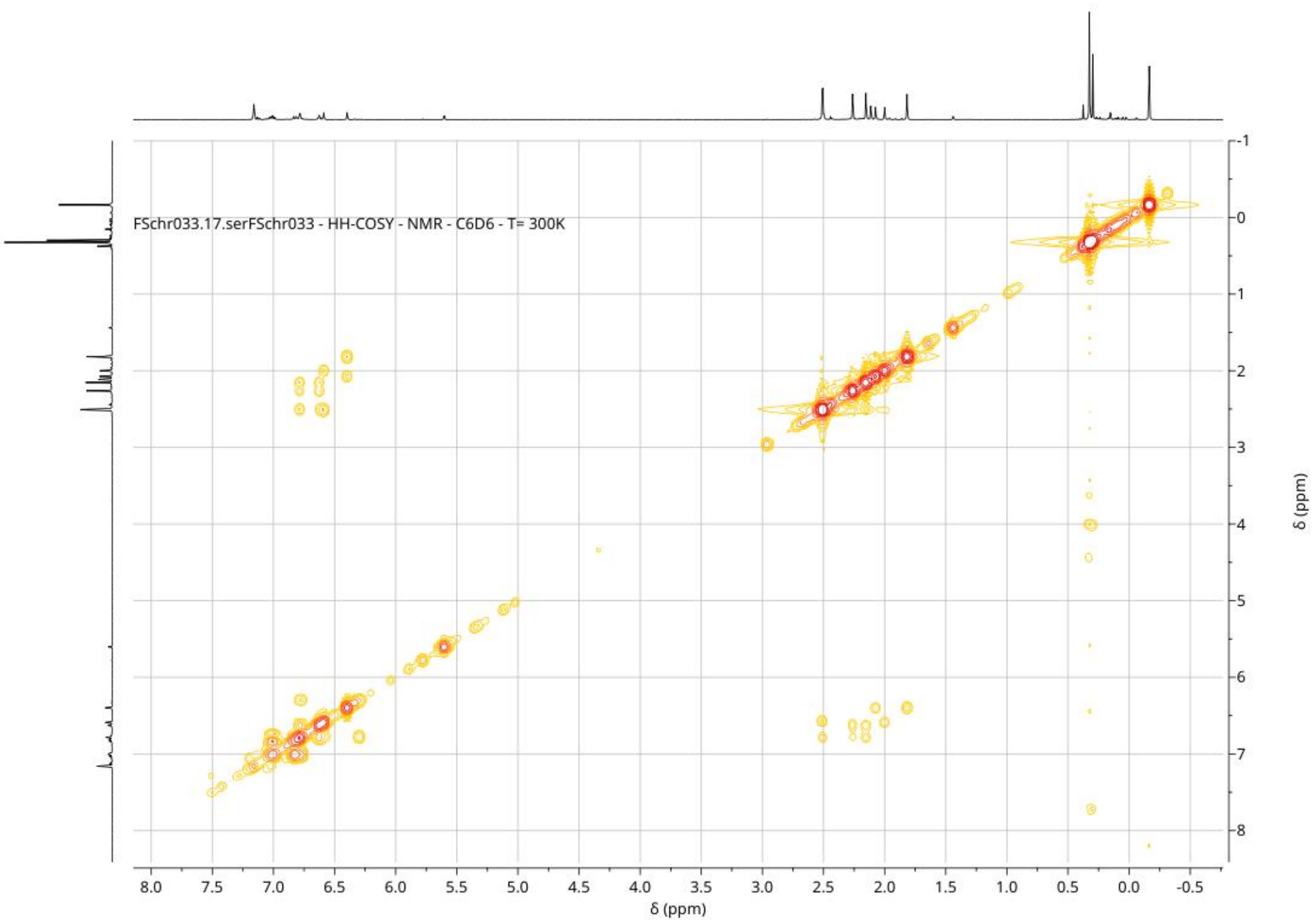

Figure S108. $^{1}H,^{1}H$ COSY NMR spectrum of **6** in C_6D_6 at 300 K and 400 MHz.

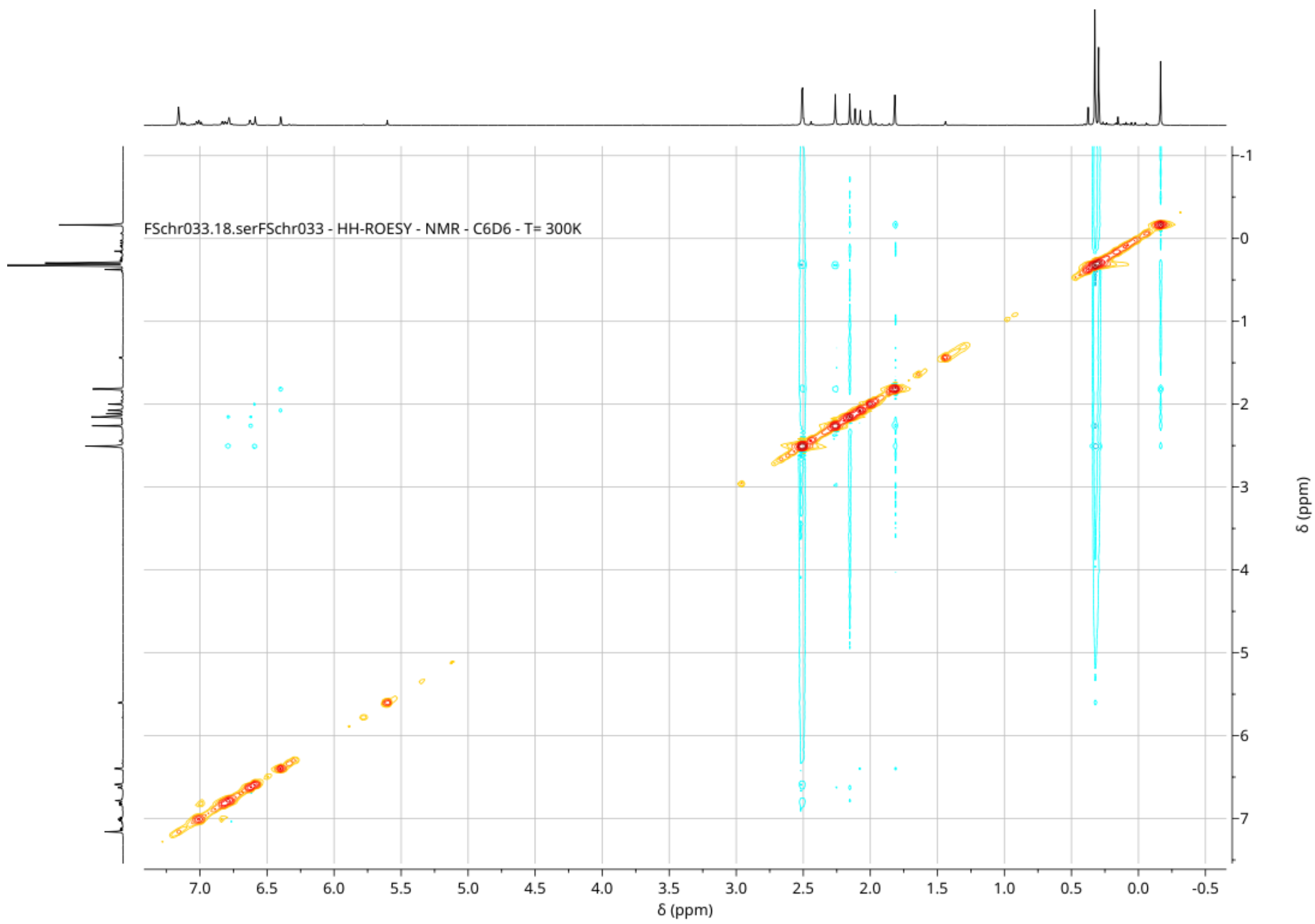

Figure S109. $^{1}H,^{1}H$ ROESY NMR spectrum of **6** in C_6D_6 at 300 K and 400 MHz.

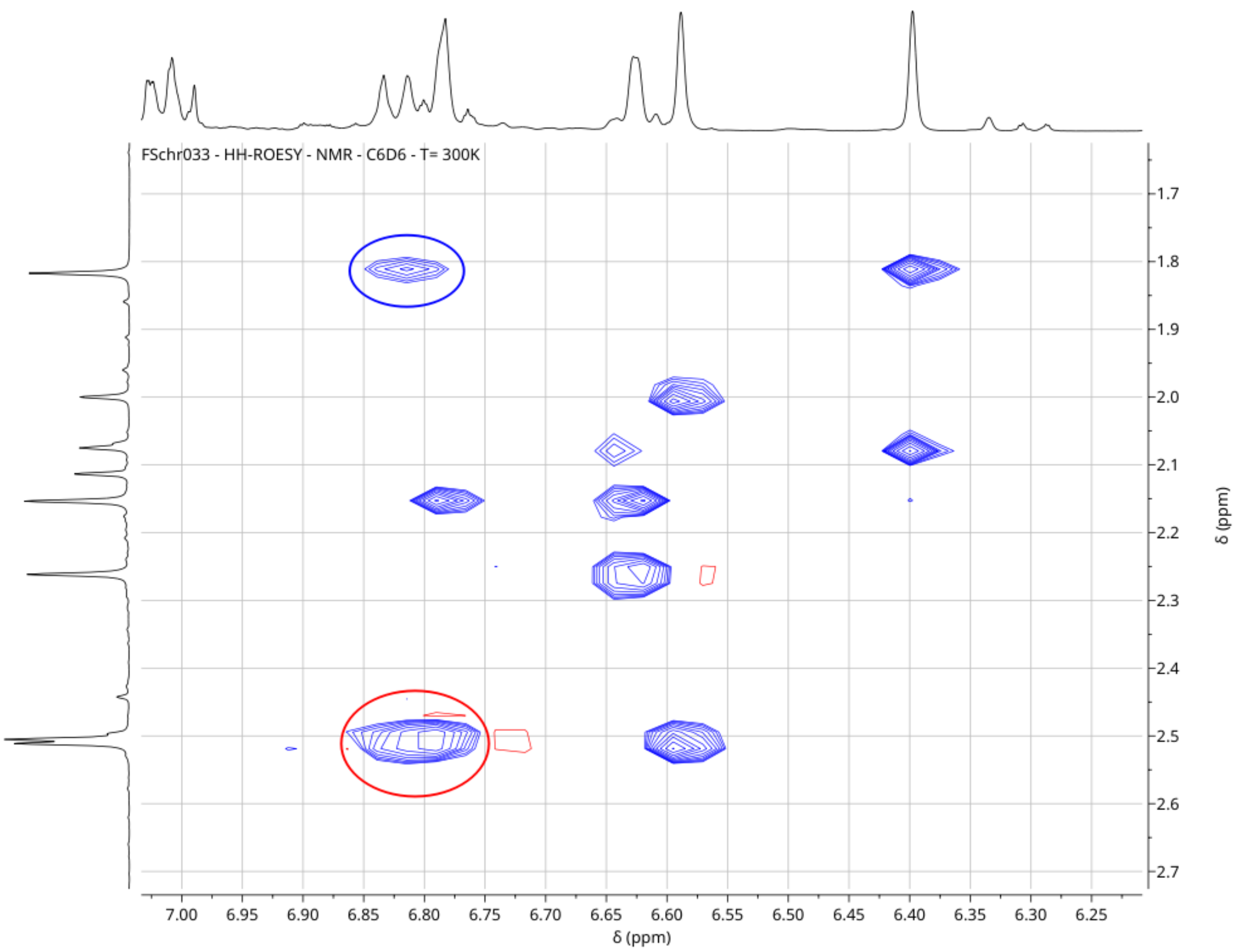

Figure S110. Zoom into the $^{1}H,^{1}H$ ROESY NMR spectrum of **6** in C_6D_6 at 300 K and 400 MHz.

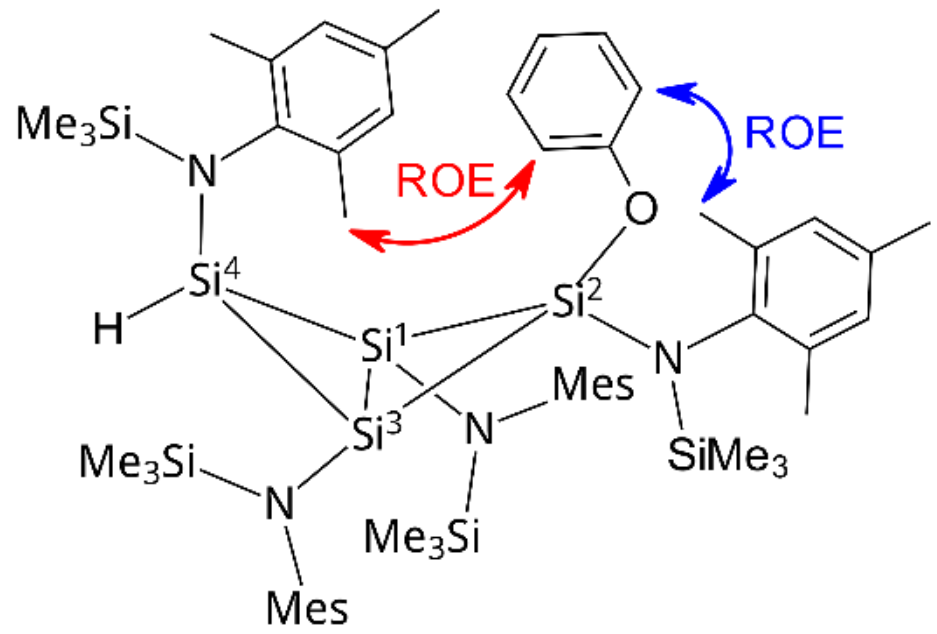

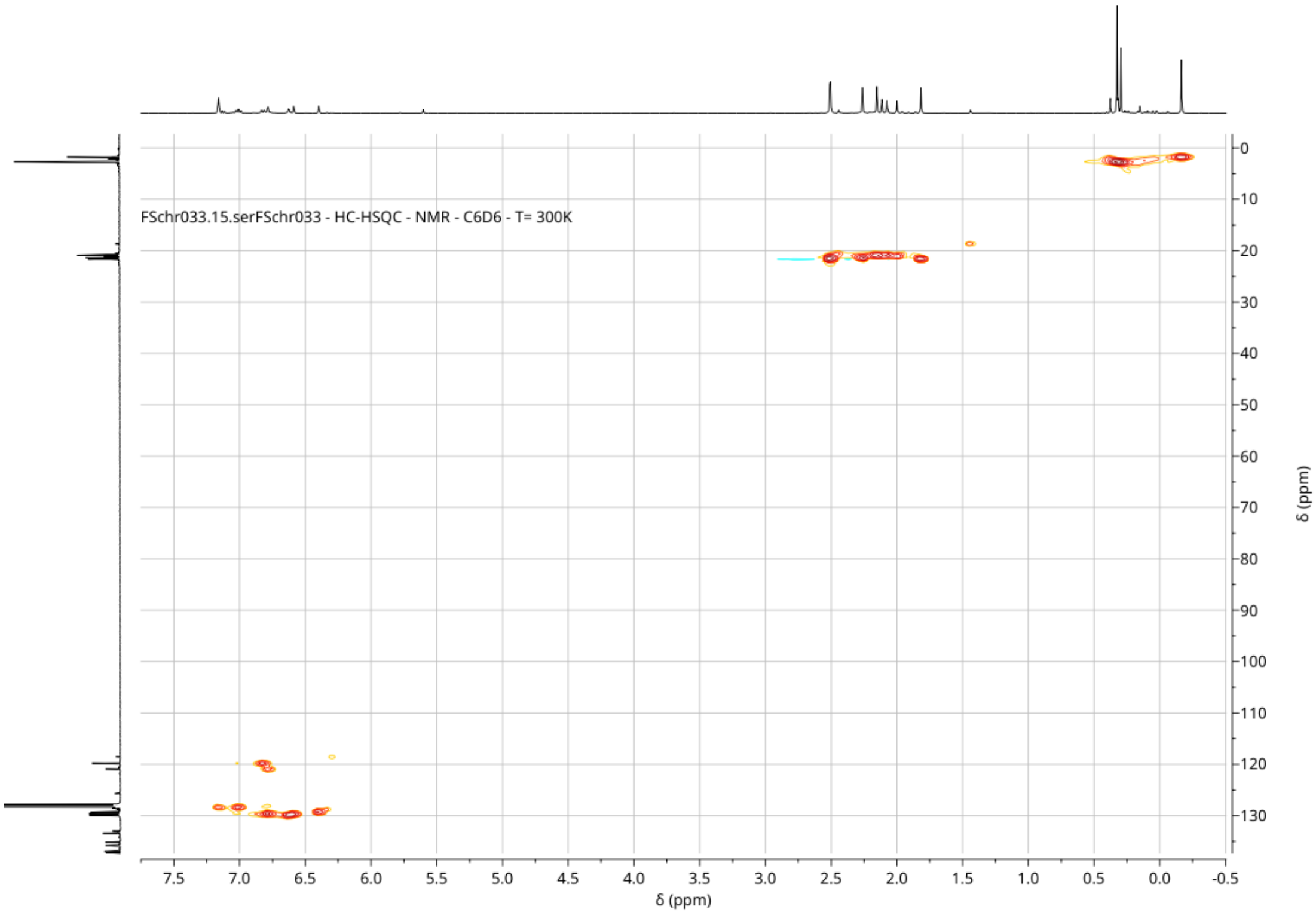

Figure S111. ^{1}H,^{13}C HSQC NMR spectrum of **6** in C_6D_6 at 300 K and 400 MHz (^{1}H) and 100 MHz (^{13}C).

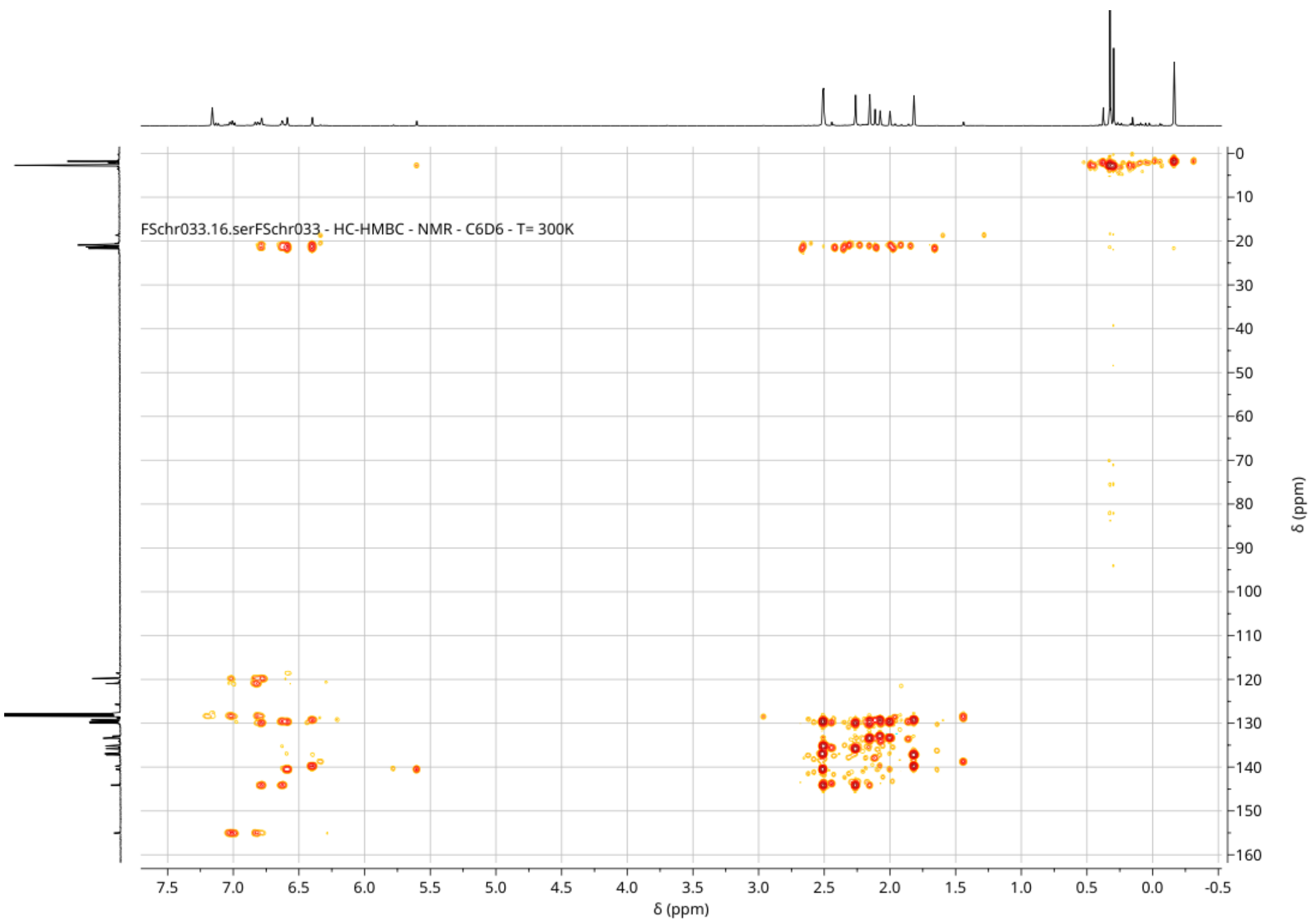

Figure S112. $^{1}H,^{13}C$ HMBC NMR spectrum of **6** in C_6D_6 at 300 K and 400 MHz (^{1}H) and 100 MHz (^{13}C).

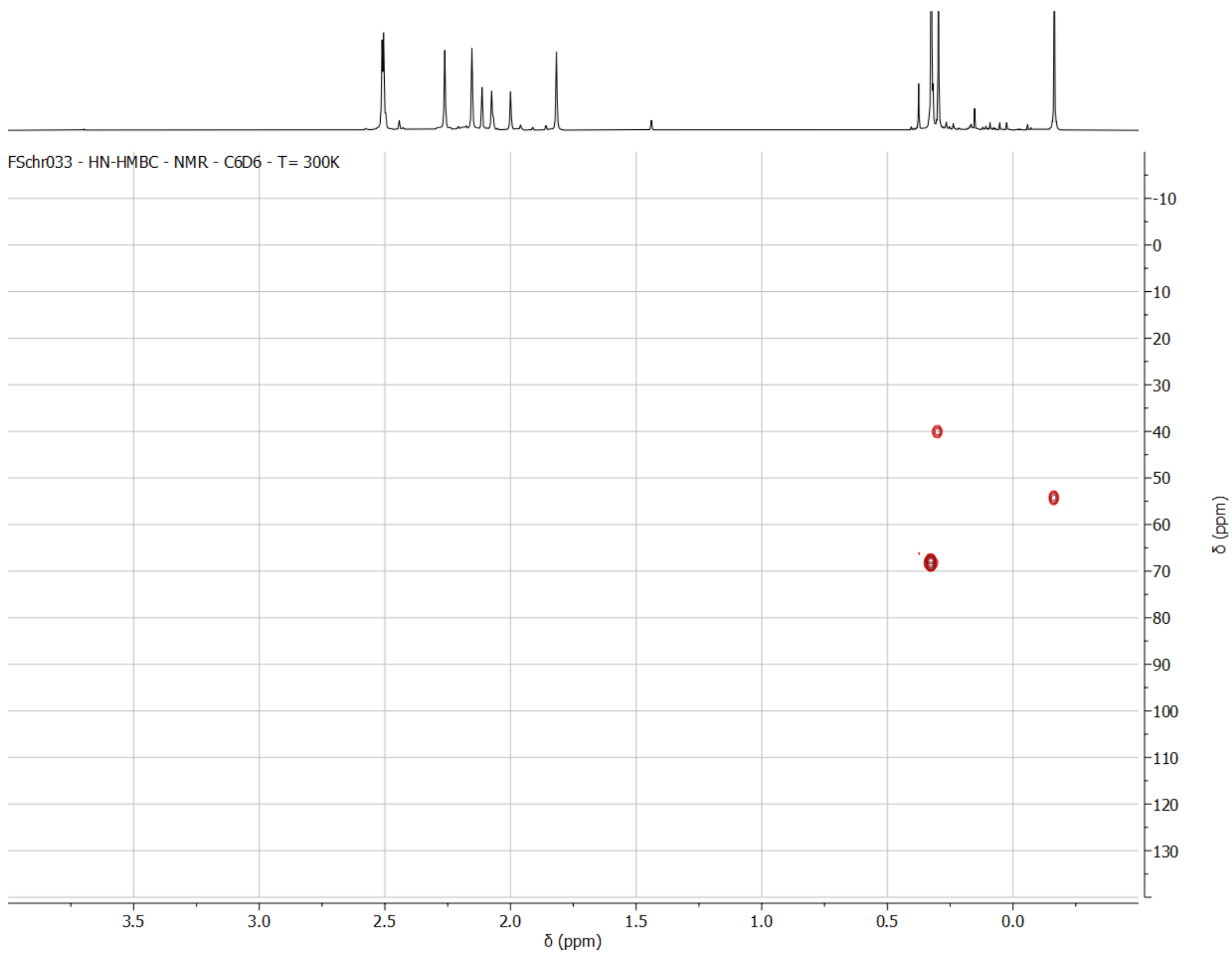

Figure S113. $^{1}H,^{15}N$ HMBC NMR spectrum of **6** in C_6D_6 at 300 K and 400 MHz (^{1}H) and 40 MHz (^{15}N).

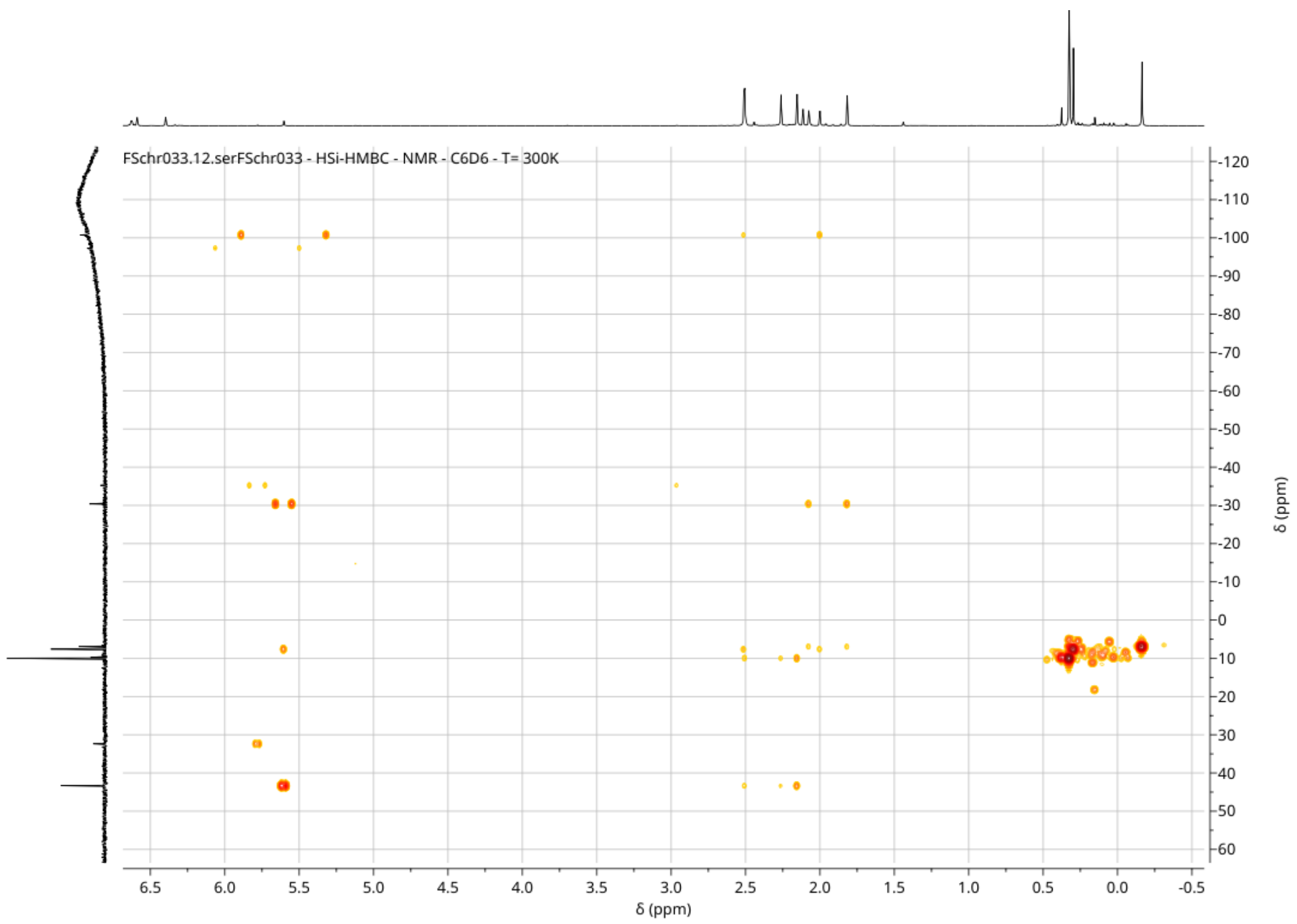

Figure S114. $^{1}H,^{29}Si$ HMBC NMR spectrum of **6** in C_6D_6 at 300 K and 400 MHz (^{1}H) and 79 MHz (^{29}Si).

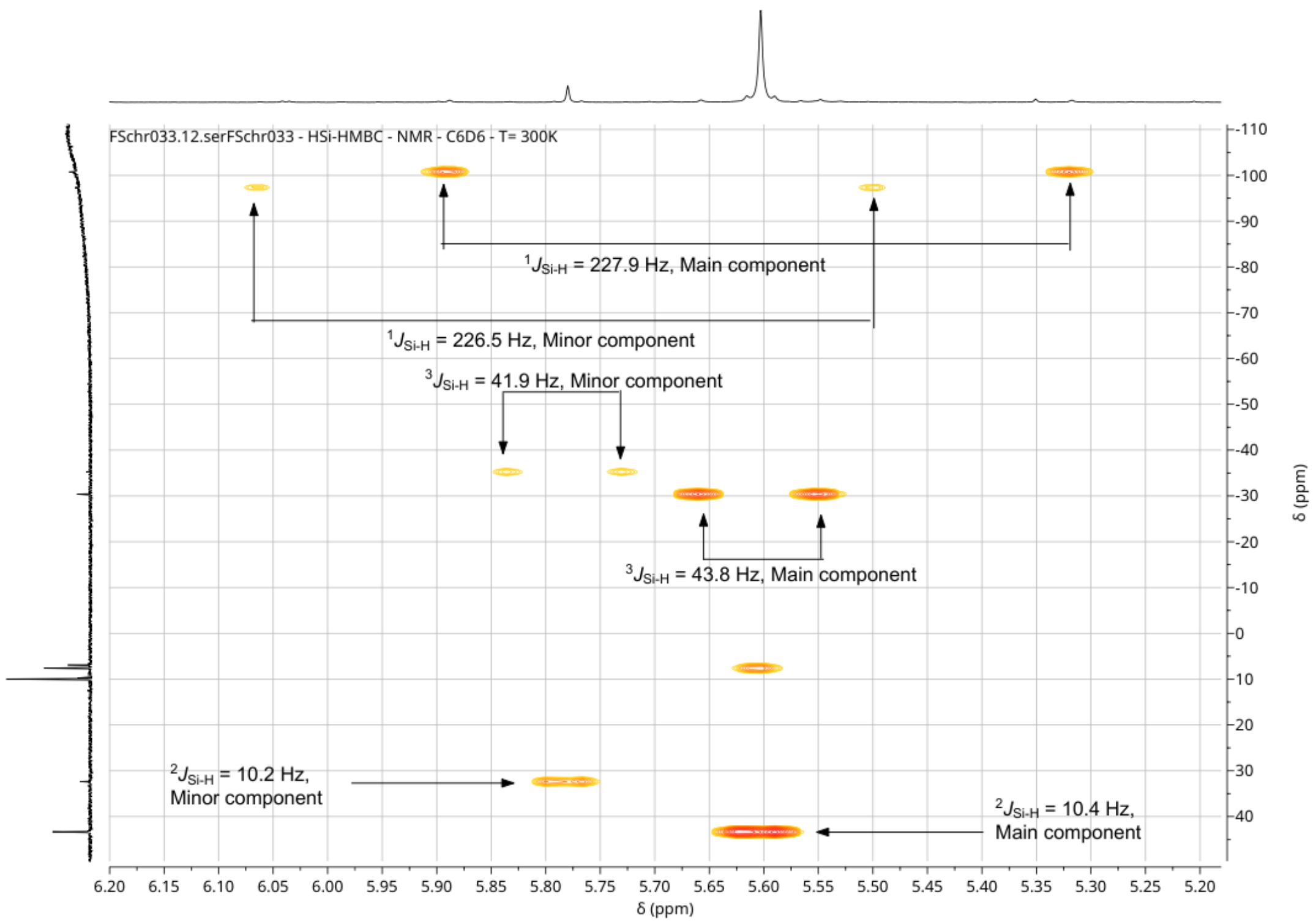

Figure S115. Zoom into the $^{1}H,^{29}Si$ HMBC NMR spectrum of **6** in C_6D_6 at 300 K and 400 MHz (^{1}H) and 79 MHz (^{29}Si).

3. Details of Single Crystal X-ray Diffraction Analyses

Crystals of **1**($\cdot 2C_6H_6$), **2**, **3**, **5**$\cdot 2CHCl_3$, and **7**·DME were removed from a Schlenk tube under an argon atmosphere and covered with a layer of hydrocarbon oil. A suitable crystal was selected, attached to a glass fiber, and quickly placed in a low temperature argon stream. The data were collected at 100 K on a Bruker Venture with Mo K_α radiation (λ = 0.71073 Å), corrected for Lorentz and polarization effects with Saint[28] and absorption using Blessing's method,[29] as incorporated into the program SADABS,[30, 31] and merged. Crystal structures were solved by using the SHELXTL version 6.1 program package to determine the space groups based upon intensity statistics.[32] The structures were determined by direct methods with most of the non-hydrogen atoms from the molecule of interest being located directly using the program SHELXT.[33] Refinement of the structures were achieved using the program SHELXL with all non-hydrogen atoms refined anisotropically.[34] Difference-Fourier least-squares refinement cycles were required to locate the remaining non-hydrogen atoms. Structures were visualized using Diamond.[35]

3.1. Refinement details

Compound **1**($\cdot 2C_6H_6$) contains two independent molecules of **1** in the asymmetric unit. One of them has two disordered mesityl groups that were refined on split positions with ratios 46:54 and 65:35, respectively. In addition, one $SiMe_3$ group is disordered over three positions that were refined accordingly with a 46:28:26 ratio. Furthermore, two solvent molecules (C_6H_6) are included in the asymmetric unit that were treated with the Platon squeeze-routine[36, 37] and nine reflections were omitted. For compounds **2** and **3**, no restraints or constraints were used in the refinement, but two and four reflections were omitted, respectively. Compound **5**$\cdot 2CHCl_3$ includes two disordered solvent molecules that were refined with split positions with ratios 85:15 and 82:18, respectively, and two reflections were omitted. In compound **7**·DME, the hydride substituent and the phenyl group are disordered over two positions that were refined with split positions with a 93:7 ratio. In addition, three $SiMe_3$ groups are disordered over two positions and were refined with split positions with ratios 86:14, 89:11, and 92:8, respectively. Furthermore, 16 reflections were omitted.

Table S1. Crystal data and structure refinement for **1** and **2**.

**Compound**	**1**	**2**
CCDC number	2543842	2543843
Empirical formula	$C_{48}H_{86}N_6Si_8$	$C_{36}H_{65}N_3O_3Si_7$
Formula weight (g·mol^{-1})	971.94	784.54
Crystal color, shape	yellow, plate	pale yellow, plate
Crystal size (mm)	0.106 × 0.326 × 0.381	0.070 × 0.095 × 0.159
Crystal system	monoclinic	triclinic
Space group	*C*2/*c*	*P*-1
a (Å)<br>*b* (Å)<br>*c* (Å)	45.110(3)<br>12.5606(9)<br>42.591(3)	13.1103(5)<br>13.2345(5)<br>15.5761(6)
α (°)<br>*c* (°)<br>γ (°)	90<br>92.748(3)<br>90	97.4930(10)<br>100.6140(10)<br>119.4290(10)
V (Å^3)	24105(3)	2235.22(15)
Z	16	2
T (K)	100(2)	100(2)
Completeness to θ 25.24° (%)	99.9	98.4
ρ_{calc} (g·cm^{-3})	1.071	1.166
μ(Mo) (mm^{-1})	0.213	0.249
2Θ range (°)	1.915–27.134	1.972–27.173
Reflections measured	154374	28240
Independent reflections	26592	9725
R(int)	0.0420	0.0533
Ind. reflections ($I > 2\sigma(I)$)	21673	7877
Parameters	1370	476
Restraints	598	6
R_1 ($I > 2\sigma(I)$)	0.0642	0.0521
wR_2 (all data)	0.1586	0.1582
GooF (all data)	1.067	1.010
Max. peak / hole (e$^-$·Å^{-3})	1.917 / −0.765	0.723 / −0.772
Absorption correction type	multiscan	multiscan
Min. / Max. transmission	0.6403 / 0.7421	0.5582 / 0.7455

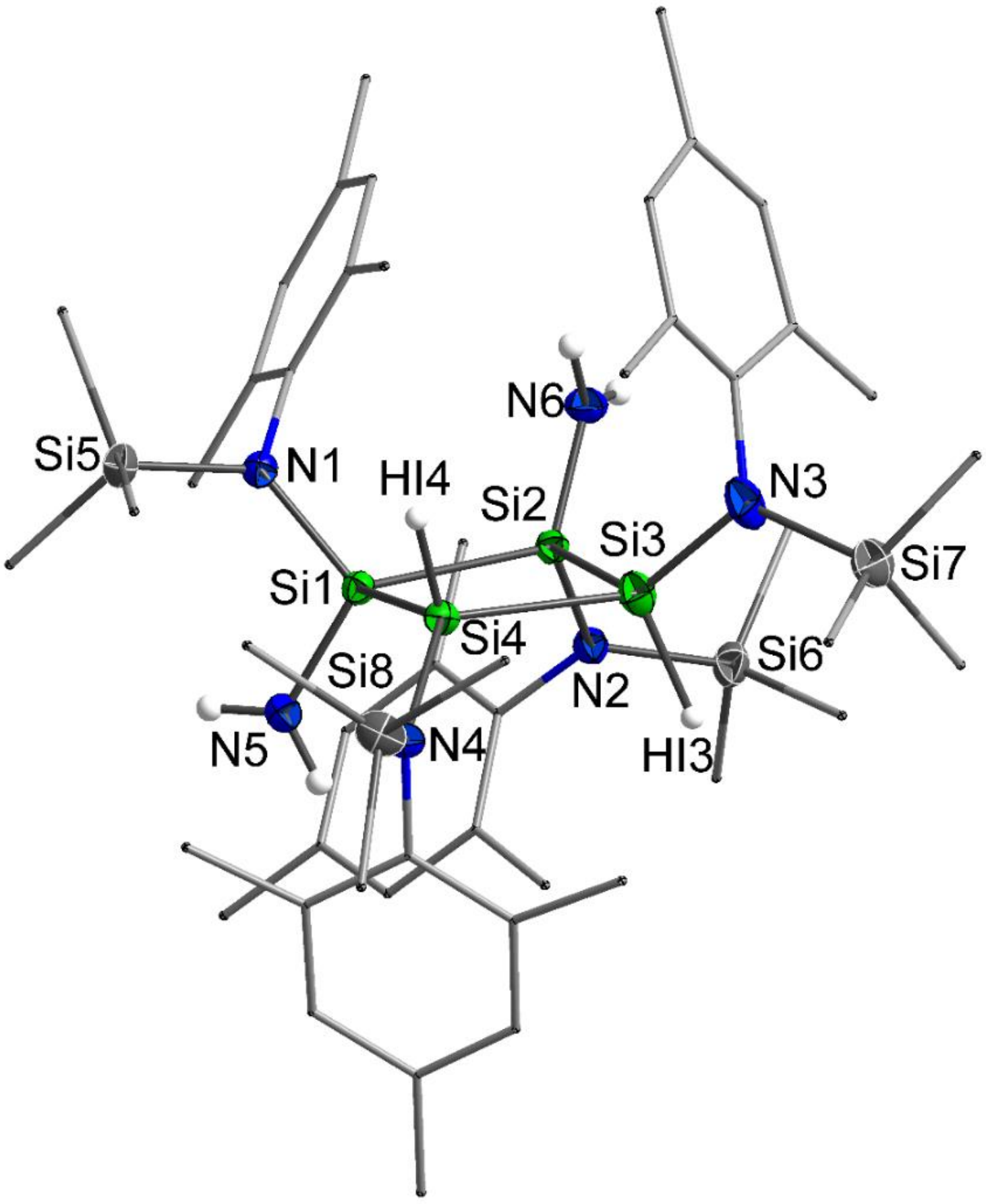

Figure S116. Molecular structure of first independent molecule of **1** with thermal ellipsoids drawn at 50 % probability level and C–H hydrogen atoms omitted for clarity. Selected bond lengths (Å) and angles (°): Si1–Si2 2.4127(9), Si2–Si3 2.4059(10), Si3–Si4 2.4211(10), Si1–Si4 2.4084(10), Si3–HI3 1.54(4), Si4–HI4 1.41(3), Si1–N1 1.750(2), Si2–N2 1.754(2), Si1–N5 1.725(2), Si3–N3 1.770(2), Si2–N6 1.720(2), Si4–N4 1.758(2), Si5–N1 1.759(2), Si6–N2 1.763(2), Si7–N3 1.788(7), Si8–N4 1.753(2), Si5–C10 1.860(3), Si5–C11 1.864(3), Si5–C12 1.870(3), Si6–C22 1.858(3), Si6–C23 1.867(3), Si6–C24 1.870(3), Si7–C34 1.856(10), Si7–C35 1.863(8), Si7–C36 1.842(9), Si8–C46 1.861(3), Si8–C48 1.862(3), Si8–C47 1.865(4), N5-Si1-N1 107.03(11), N5-Si1-Si4 109.77(8), N1-Si1-Si4 115.91(8), N5-Si1-Si2 120.10(8), N1-Si1-Si2 113.56(8), Si4-Si1-Si2 90.04(3), N6-Si2-N2 109.74(11), N6-Si2-Si3 111.23(9), N2-Si2-Si3 113.91(8), N6-Si2-Si1 119.09(8), N2-Si2-Si1 111.77(8), Si3-Si2-Si1 89.97(3), N3-Si3-Si2 121.99(9), N3-Si3-Si4 122.51(9), Si2-Si3-Si4 89.90(3), N3-Si3-HI3 106.2(14), Si2-Si3-HI3 107.3(14), Si4-Si3-HI3 107.4(14), N4-Si4-Si1 122.10(8), N4-Si4-Si3 121.51(8), Si1-Si4-Si3 89.71(3), N4-Si4-HI4 105.6(12), Si1-Si4-HI4 108.7(12), Si3-Si4-HI4 108.0(12), N1-Si5-C10 109.28(13), N1-Si5-C11 109.53(13), C10-Si5-C11 108.68(15), N1-Si5-C12 113.53(13), C10-Si5-C12 107.33(17), C11-Si5-C12 108.38(18), N2-Si6-C22 112.02(14), N2-Si6-C23 112.93(13), C22-Si6-C23 107.20(16), N2-Si6-C24 107.35(12), C22-Si6-C24 109.12(16), C23-

Si6-C24 108.11(16), C25-N3-Si7 121.0(3), Si3-N3-Si7 122.2(3), N3-Si7-C36 114.9(4), N3-Si7-C34 107.0(7), C36-Si7-C34 109.5(6), N3-Si7-C35 110.1(4), C36-Si7-C35 108.2(5), C34-Si7-C35 107.0(6), N4-Si8-C46 107.83(13), N4-Si8-C48 112.21(13), C46-Si8-C48 110.10(17), N4-Si8-C47 112.51(13), C46-Si8-C47 108.95(18), C48-Si8-C47 105.21(16).

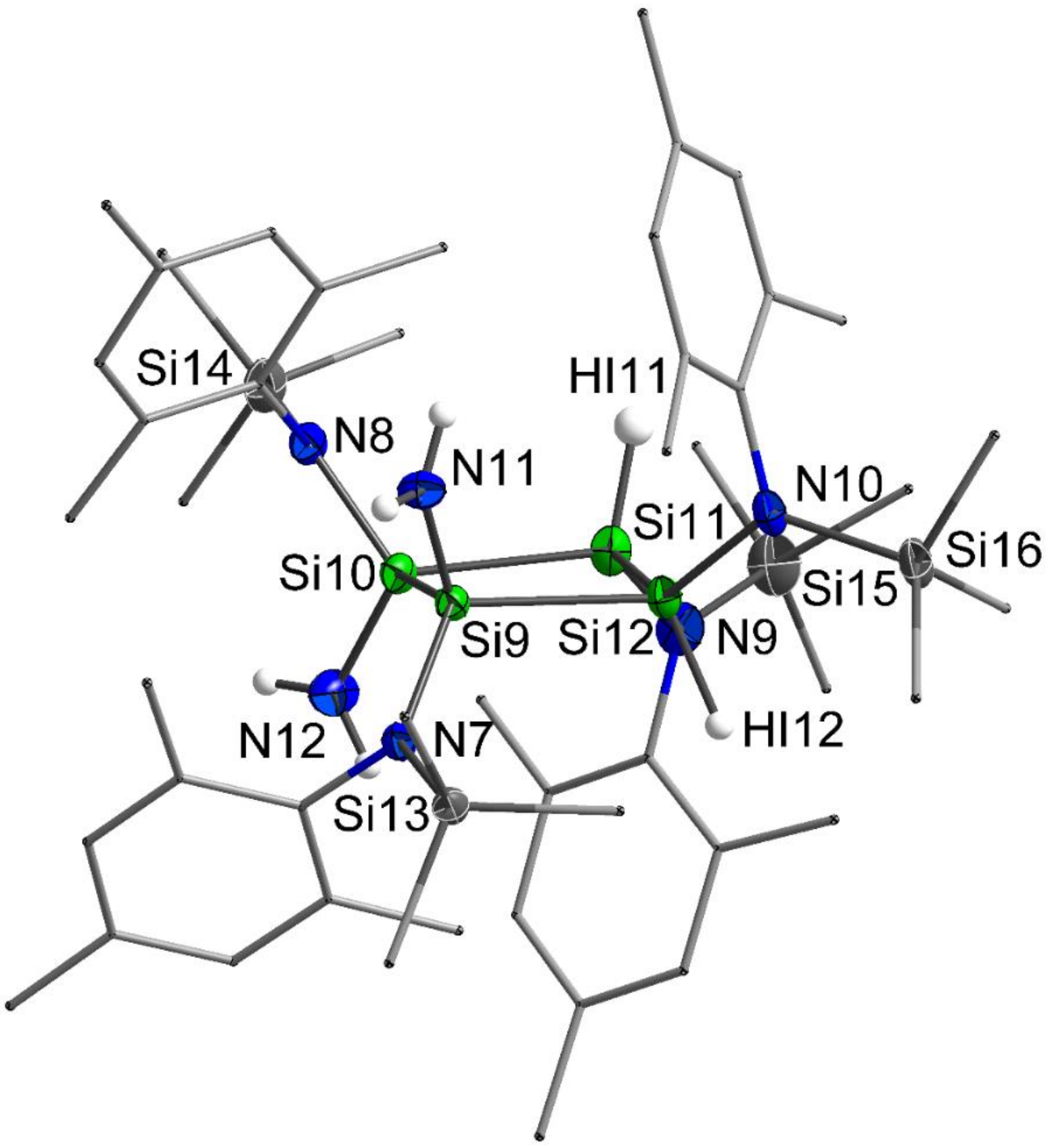

Figure S117. Molecular structure of second independent molecule of **1** with thermal ellipsoids drawn at 50 % probability level and C–H hydrogen atoms omitted for clarity. Selected bond lengths (Å) and angles (°): Si9–Si10 2.4082(10), Si10–Si11 2.4152(11), Si11–Si12 2.4155(10), Si9–Si12 2.4066(10), Si11–HI11 1.42(3), Si12–HI12 1.53(3), Si9–N7 1.753(2), Si9–N11 1.724(2), Si10–N8 1.759(2), Si10–N12 1.724(3), Si11–N9 1.757(2), Si12–N10 1.761(2), Si13–N7 1.761(2), Si14–N8 1.758(2), Si15–N9 1.740(3), Si16–N10 1.756(2), Si13–C58 1.859(3), Si13–C59 1.863(3), Si13–C60 1.864(3), Si14–C70 1.860(4), Si14–C71 1.878(4), Si14–C72 1.858(4), Si15–C82 1.869(4), Si15–C83 1.872(4), Si15–C84 1.872(4), Si16–C94 1.863(3), Si16–C95 1.864(3), Si16–C96 1.867(4), N11-Si9-N7 108.72(11), N11-Si9-Si12 109.37(9), N7-Si9-Si12 115.60(8), N11-Si9-Si10 119.57(8), N7-Si9-Si10 112.69(8), Si12-Si9-Si10 90.06(3), N12-Si10-N8 108.33(12), N12-Si10-Si9 118.53(9), N8-Si10-Si9 112.61(8), N12-Si10-Si11 111.27(10), N8-Si10-Si11 115.50(9), Si9-Si10-Si11 89.94(3), N9-Si11-Si10 122.96(10), N9-

Si11-Si12 120.41(9), Si10-Si11-Si12 89.68(3), N9-Si11-HI11 104.7(11), Si10-Si11-HI11 108.5(12), Si12-Si11-HI11 109.8(11), N10-Si12-Si9 121.64(8), N10-Si12-Si11 123.45(8), Si9-Si12-Si11 89.97(3), N10-Si12-HI12 103.6(10), Si9-Si12-HI12 111.6(10), Si11-Si12-HI12 105.7(10), N7-Si13-C58 109.97(12), N7-Si13-C59 109.36(12), C58-Si13-C59 109.45(14), N7-Si13-C60 113.04(12), C58-Si13-C60 106.25(15), C59-Si13-C60 108.68(14), N8-Si14-C72 111.57(13), N8-Si14-C70 108.26(14), C72-Si14-C70 108.86(18), N8-Si14-C71 112.08(14), C72-Si14-C71 107.15(19), C70-Si14-C71 108.85(18), N9-Si15-C82 110.56(15), N9-Si15-C84 107.77(16), C82-Si15-C84 110.41(18), N9-Si15-C83 113.29(17), C82-Si15-C83 107.1(2), C84-Si15-C83 107.6(2), N10-Si16-C94 107.49(13), N10-Si16-C95 111.64(13), C94-Si16-C95 109.54(15), N10-Si16-C96 113.51(13), C94-Si16-C96 108.53(18), C95-Si16-C96 106.06(19).

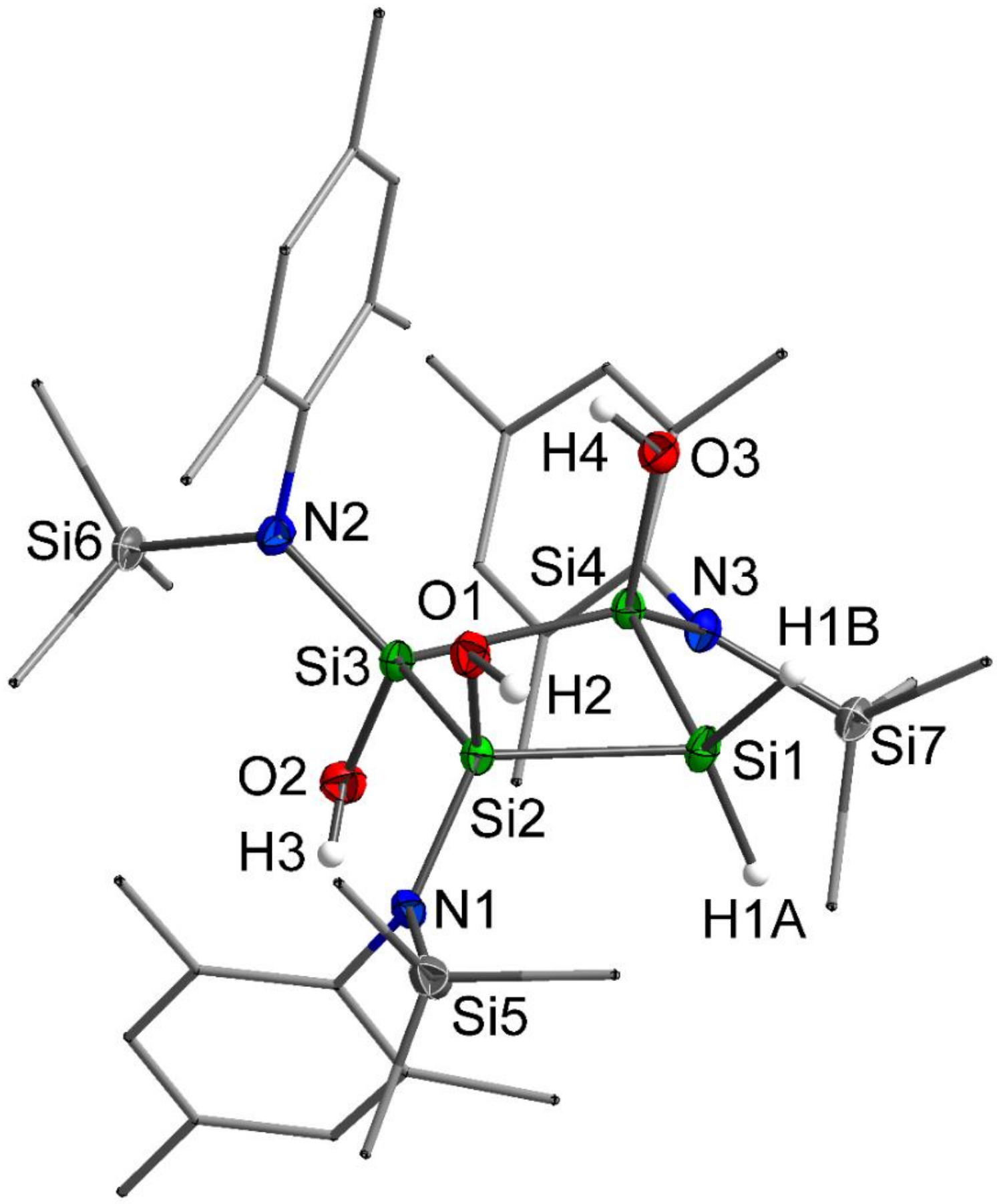

Figure S118. Molecular structure of **2** with thermal ellipsoids drawn at 50 % probability level and C–H hydrogen atoms omitted for clarity. Selected bond lengths (Å) and angles (°): Si1–H1A 1.41(4), Si1–H1B 1.44(3), Si1–Si4 2.3545(9), Si1–Si2 2.3874(10), Si2–Si3 2.4243(9), Si3–Si4 2.4054(9), Si2–O1 1.6412(18), Si3–O2 1.6511(19), Si4–O3 1.6775(19), Si2–N1 1.738(2), Si3–N2 1.738(2), Si4–N3 1.728(2), Si5–N1 1.758(2), Si6–N2 1.762(2), Si7–N3 1.757(2), Si5–C12 1.858(3), Si5–C11 1.863(3), Si5–C10 1.870(3), Si6–C22 1.862(3), Si6–C23

1.863(3), Si6–C24 1.869(3), Si7–C34 1.864(3), Si7–C35 1.867(3), Si7–C36 1.868(3), Si4-Si1-Si2 89.09(3), Si1-Si2-Si3 89.69(3), Si4-Si3-Si2 87.06(3), Si1-Si4-Si3 90.93(3), Si4-Si1-H1A 114.8(14), Si2-Si1-H1A 115.7(15), Si4-Si1-H1B 109.0(13), Si2-Si1-H1B 112.5(13), H1A-Si1-H1B 113(2), O1-Si2-N1 107.52(10), O1-Si2-Si1 107.73(7), O1-Si2-Si3 115.62(7), O2-Si3-N2 105.23(10), O3-Si4-N3 110.00(10), O2-Si3-Si4 109.99(8), O2-Si3-Si2 109.80(8), O3-Si4-Si1 103.70(7), O3-Si4-Si3 107.61(8), N1-Si2-Si1 119.21(8), N1-Si2-Si3 116.36(7), N2-Si3-Si4 119.29(8), N2-Si3-Si2 124.44(8), N3-Si4-Si1 116.97(8), N3-Si4-Si3 124.72(8).

Table S2. Crystal data and structure refinement for **3** and **5**·$2CHCl_3$.

**Compound**	**3**	**5·2CHCl₃**
CCDC number	2543844	2543845
Empirical formula	$C_{74}H_{126}N_6O_2Si_{14}$	$C_{80}H_{134}Cl_{12}N_6O_2Si_{14}$
Formula weight (g·mol^{-1})	1525.06	2030.58
Crystal color, shape	pale yellow, plate	yellow, block
Crystal size (mm)	0.165 × 0.229 × 0.234	0.206 × 0.295 × 0.333
Crystal system	monoclinic	monoclinic
Space group	*C*2/*c*	*C*2/*c*
a (Å)<br>*b* (Å)<br>*c* (Å)	26.5830(6)<br>13.7098(3)<br>25.0101(6)	34.0470(7)<br>14.1756(3)<br>25.5561(5)
α (°)<br>*c* (°)<br>γ (°)	90<br>105.2290(10)<br>90	90<br>122.4360(10)<br>90
V (Å^3)	8794.8(4)	10410.0(4)
Z	4	4
T (K)	100(2)	100(2)
Completeness to θ 25.24° (%)	99.5	99.9
ρ_{calc} (g·cm^{-3})	1.152	1.296
μ(Mo) (mm^{-1})	0.248	0.525
2Θ range (°)	1.975–27.119	1.888–27.123
Reflections measured	59801	70436
Independent reflections	9684	11497
R(int)	0.0232	0.0201
Ind. reflections ($I > 2\sigma(I)$)	8602	10639
Parameters	452	607
Restraints	0	96
R_1 ($I > 2\sigma(I)$)	0.0316	0.0351
wR_2 (all data)	0.0870	0.0970
GooF (all data)	1.042	1.082
Max. peak / hole (e$^-$·Å^{-3})	0.398 / −0.235	1.276 / −0.808
Absorption correction type	multiscan	multiscan
Min. / Max. transmission	0.5664 / 0.7455	0.6067 / 0.7455

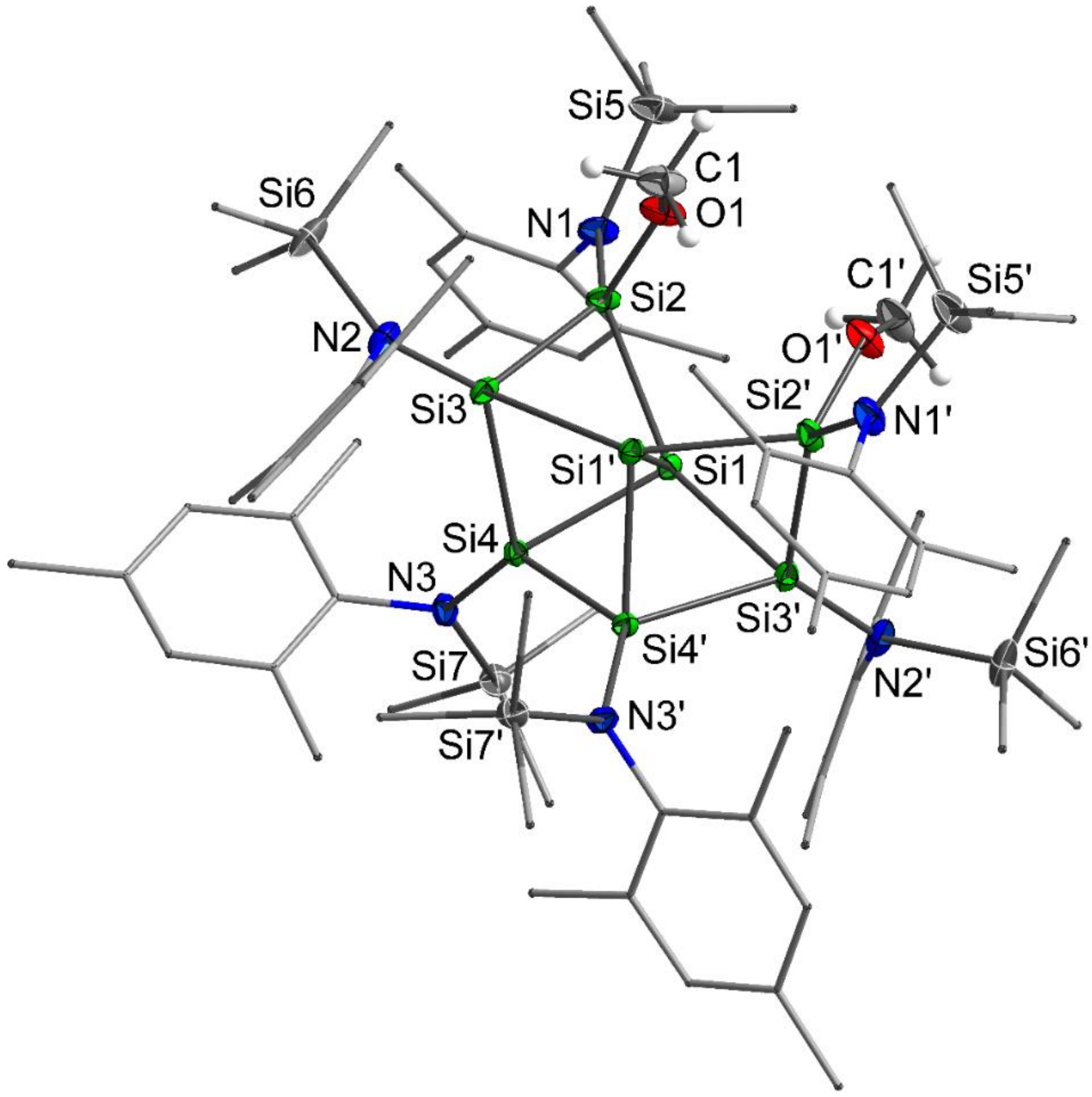

Figure S119. Molecular structure of **3** with thermal ellipsoids drawn at 50 % probability level and most C–H hydrogen atoms omitted for clarity. Selected bond lengths (Å) and angles (°): O1–C1 1.4130(18), Si1–Si4 2.3472(5), Si1–Si3' 2.3817(5), Si1–Si2 2.3827(5),Si1–Si1' 2.4586(7), Si2–Si3 2.3950(5), Si3–Si4 2.3917(5), Si4–Si4' 2.3753(7), Si2–O1 1.6450(10), Si2–N1 1.7416(12), Si3–N2 1.7466(12), Si4–N3 1.7493(11), Si5–N1 1.7805(12), Si6–N2 1.7605(12), Si7–N3 1.7640(12), Si5–C13 1.866(2), Si5–C11 1.867(2), Si5–C12 1.8706(17), Si6–C24 1.862(2), Si6–C23 1.8653(18), Si6–C25 1.8653(17), Si7–C37 1.8522(15), Si7–C36 1.8620(17), Si7–C35 1.8639(16), Si4-Si1-Si3' 102.101(17), Si4-Si1-Si2 97.783(18), Si3'-Si1-Si2 144.41(2), Si4-Si1-Si1' 74.540(14), Si3'-Si1-Si1' 74.970(18), Si2-Si1-Si1' 82.402(17), Si1-Si2-Si3 76.151(16), Si1'-Si3-Si4 75.173(15), Si1'-Si3-Si2 83.780(17), Si4-Si3-Si2 96.237(18), Si1-Si4-Si4' 76.128(13), Si1-Si4-Si3 76.882(16), Si4-Si4'-Si3' 100.978(17), O1-Si2-N1 94.69(5), O1-Si2-Si1 118.58(4), O1-Si2-Si3 112.95(4), N1-Si2-Si1 121.07(5), N1-Si2-Si3 135.57(5), N2-Si3-Si1' 120.78(4), N2-Si3-Si4 134.09(5), N2-Si3-Si2 126.36(4), N3-Si4-Si1 125.29(4), N3-Si4-Si4' 130.28(4), N3-Si4-Si3 126.04(4).

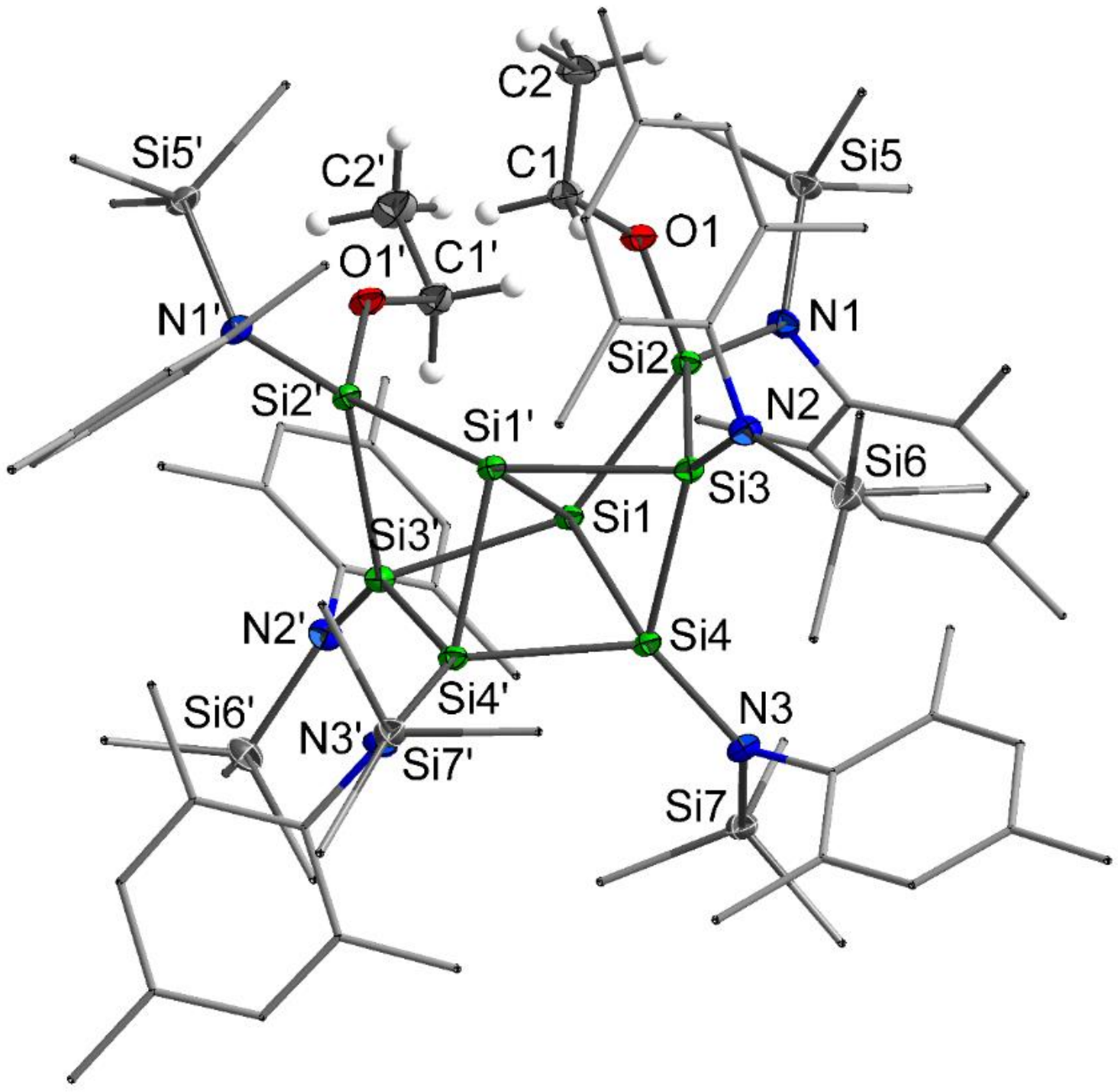

Figure S120. Molecular structure of **5** with thermal ellipsoids drawn at 50 % probability level and most C–H hydrogen atoms omitted for clarity. Selected bond lengths (Å) and angles (°): O1–C1 1.4316(18), C1–C2 1.516(2), Si2–O1 1.6489(11), Si1–Si4 2.3658(5), Si1–Si2 2.4012(5), Si1–Si3' 2.4102(5), Si1–Si1' 2.4420(7), Si2–Si3 2.4051(5), Si3–Si4 2.3768(5), Si4–Si4' 2.3836(7), Si2–N1 1.7441(12), Si3–N2 1.7531(13), Si4–N3 1.7540(12), Si6–N2 1.7666(13), Si5–N1 1.7760(13), Si7–N3 1.7655(13), Si5–C13 1.8667(18), Si5–C14 1.8683(18), Si5–C12 1.8765(17), Si6–C25 1.8547(18), Si6–C24 1.8688(17), Si6–C26 1.8732(17), Si7–C36 1.8596(17), Si7–C38 1.8603(16), Si7–C37 1.8654(17), N1–C3 1.4475(19), N2–C15 1.4504(18), N3–C27 1.4469(19), Si4-Si1-Si2 97.757(19), Si4-Si1-Si3' 101.792(19), Si2-Si1-Si3' 144.16(2), Si4-Si1-Si1' 74.490(14), Si2-Si1-Si1' 81.575(18), Si3'-Si1-Si1' 75.22(2), Si1-Si2-Si3 76.070(17), Si4-Si3-Si2 97.349(19), Si4-Si3-Si1' 74.881(17), Si2-Si3-Si1' 82.153(17), Si1-Si4-Si3 77.278(17), Si1-Si4-Si4' 75.583(15), Si3-Si4-Si4' 102.258(19), O1-Si2-N1 95.61(6), O1-Si2-Si1 122.93(4), O1-Si2-Si3 111.20(4), N1-Si2-Si1 118.06(4), N1-Si2-Si3 135.99(5), N2-Si3-Si4 133.45(5), N2-Si3-Si2 126.74(5), N2-Si3-Si1' 120.47(5), N3-Si4-Si1 127.12(5), N3-Si4-Si3 125.25(5), N3-Si4-Si4' 129.32(4).

Table S3. Crystal data and structure refinement for **7**.

**Compound**	**7·DME**
CCDC number	2543846
Empirical formula	$C_{80}H_{131}N_6O_2Si_{14}$
Formula weight (g·mol^{-1})	1602.16
Crystal color, shape	yellow, rod
Crystal size (mm)	0.078 × 0.140 × 0.313
Crystal system	triclinic
Space group	*P*-1
a (Å)<br>*b* (Å)<br>*c* (Å)	12.7015(7)<br>15.6017(10)<br>23.9509(16)
α (°)<br>*c* (°)<br>γ (°)	87.026(2)<br>79.545(3)<br>74.519(2)
V (Å^3)	4498.1(5)
Z	2
T (K)	100(2)
Completeness to θ 25.24° (%)	98.7
ρ_{calc} (g·cm^{-3})	1.183
μ(Mo) (mm^{-1})	0.246
2Θ range (°)	1.868–26.732
Reflections measured	56386
Independent reflections	18877
R(int)	0.0300
Ind. reflections ($I > 2\sigma(I)$)	16592
Parameters	1129
Restraints	595
R_1 ($I > 2\sigma(I)$)	0.0398
wR_2 (all data)	0.1056
GooF (all data)	1.009
Max. peak / hole (e$^-$·Å^{-3})	0.593 / −0.462
Absorption correction type	multiscan
Min. / Max. transmission	0.6821 / 0.7455

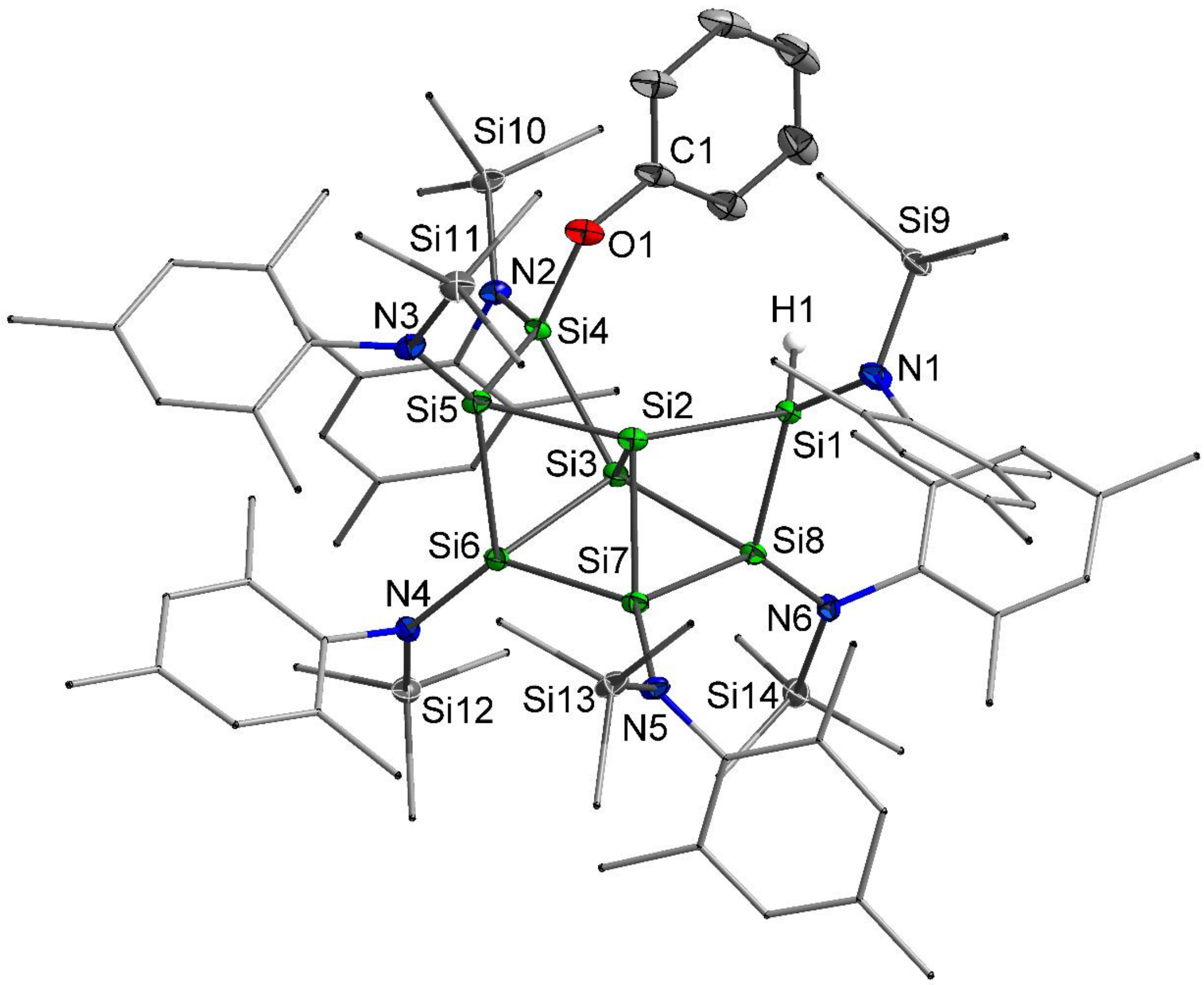

Figure S121. Molecular structure of **7** with thermal ellipsoids drawn at 50 % probability level and C–H hydrogen atoms omitted for clarity. Selected bond lengths (Å) and angles (°): Si1–H01 1.40(2), Si4–O1 1.6773(19), O1–C1 1.3641(17), Si2–Si1 2.3515(13), Si2–Si7 2.3670(6), Si2–Si5 2.3729(7), Si2–Si3 2.4548(7), Si3–Si4 2.3831(13), Si3–Si6 2.3856(7), Si3–Si8 2.3886(7), Si1–Si8 2.3708(13), Si4–Si5 2.3823(17),Si5–Si6 2.3889(6), Si6–Si7 2.3612(7), Si7–Si8 2.3755(7), Si1–N1 1.7457(19), Si4–N2 1.736(2), Si5–N3 1.7441(15), Si6–N4 1.7519(15), Si7–N5 1.7497(15), Si8–N6 1.7484(15), Si1-Si2-Si7 97.57(4), Si1-Si2-Si5 143.94(3), Si7-Si2-Si5 102.86(2), Si1-Si2-Si3 83.38(3), Si7-Si2-Si3 73.70(2), Si5-Si2-Si3 74.45(2), Si4-Si3-Si6 97.25(4), Si4-Si3-Si8 145.44(4), Si6-Si3-Si8 102.45(2), Si4-Si3-Si2 84.53(4), Si6-Si3-Si2 73.63(2), Si8-Si3-Si2 74.35(2), Si2-Si1-Si8 76.61(4), Si5-Si4-Si3 75.61(4), Si2-Si5-Si4 86.37(4), Si2-Si5-Si6 75.07(2), Si4-Si5-Si6 97.18(3), Si7-Si6-Si3 75.09(2), Si7-Si6-Si5 102.55(2), Si3-Si6-Si5 75.44(2), Si6-Si7-Si2 75.70(2), Si6-Si7-Si8 103.58(2), Si2-Si7-Si8 76.22(2), Si1-Si8-Si7 96.81(3), Si1-Si8-Si3 84.42(3), Si7-Si8-Si3 74.77(2), Si2-Si1-H01 110.9(10), Si8-Si1-H01 107.8(10), N1-Si1-H01 100.2(10), O1-Si4-N2 102.88(10), O1-Si4-Si5 102.07(8), O1-Si4-Si3 121.85(10), C1-O1-Si4 134.05(13), N1-Si1-Si2 125.45(8), N1-Si1-Si8 134.30(8), N2-Si4-Si5 136.69(10), N2-Si4-Si3 118.07(8), N3-Si5-Si2 117.65(6), N3-Si5-Si4 124.90(6), N3-Si5-Si6 135.30(6), N4-Si6-Si7 128.78(5), N4-Si6-Si3

131.05(6), N4-Si6-Si5 124.67(5), N5-Si7-Si6 130.01(6), N5-Si7-Si2 130.49(6), N5-Si7-Si8 122.28(5), N6-Si8-Si1 123.48(6), N6-Si8-Si7 135.54(5), N6-Si8-Si3 122.72(5), Si5-N3-Si11 123.87(9), Si6-N4-Si12 127.21(9), Si7-N5-Si13 124.86(8), Si8-N6-Si14 125.12(9).

4. Computational Details

The geometries of all investigated compounds **F**, **1**–**4**, and **6** along with those of the associated transition states, were optimized in the gas phase with density functional theory using the PBE1PBE functional,[16–19] def2-SVP basis sets,[20] and Grimme's D3 dispersion correction with Becke Johnson damping.[21, 22] The Gaussian 16-C.01 program suite was used for all calculations.[38] The structures were confirmed to be either minima or first order transition states on the potential energy hypersurface via calculation of the associated vibrational frequencies (all positive or one imaginary, respectively). The nature of all transition states found was further confirmed by following the reaction coordinate to both forward and reverse directions. In the mechanism for the formation of **1** from **F** and NH_3, one of the transition states was found to be lower in Gibbs free energy than the preceding minimum, though the ordering of the structures on the potential energy surface based on total (electronic) energy is as expected. For this reason, all minima and transition states on the pathway to **1** were recalculated using either bigger def2-TZVP basis sets[20] or implicit conductor-like polarizable continuum model (CPCM) for THF.[39, 40] These changes had, however, negligible impact on the calculated energetics. It is therefore believed that the negative Gibbs activation energy arises in this single instance from the 'loose' nature of the transition state and the harmonic approximation that overemphasizes the entropy contribution in particular. Implicit solvent model was, however, used in all calculations probing the formation of **6** owing to the involvement of ionic intermediates that are stabilized by the solvent environment.

4.1 Further discussion of the formation of 6

It is not immediately evident why the 1,3-*anti* isomer of **6** would be preferred over a mixture of products if the reaction involves ionic intermediates. In fact, DFT calculations showed that the 1,3-*syn* isomer of **6** is thermodynamically favored over 1,3-*anti* by 14 kJ mol^{-1} and could easily form through a 1,2-*syn* intermediate. At this point, we attribute the experimentally observed *anti*-arrangement of substituents in **6** to stabilization of the phenoxide ion through explicit interactions with solvent molecules that are not modelled by calculations. Furthermore, once the Lewis acidic (H^+) and basic (PhO^-) moieties are separated, steric effects are likely to favor their attack on opposite sides of the Si_4 ring. In this respect, 1,3,4,5-tetramethyl-imidazol-2-ylidene (NHC^{Me4}) is known to coordinate to the Lewis acidic Si^+ center in **F** exclusively from a single side, with simultaneous coordination of NHC^{Me4} and BH_3 giving the 1,3-*anti* isomer.[6]

5. Cartesian Coordinates and Gibbs Energies

```
140
F, G=-4406.094921
 Si      -1.460573558307      0.632923789807     -0.761891992206
 Si       0.304829898006      0.335627037749      0.591109213078
 Si      -0.118071289260     -1.486651537540     -0.629079787654
 Si      -2.483011033400     -1.493495148139     -0.751488465693
 Si      -3.234104544101      1.861799427176     -2.917274689223
 Si       1.360114335333      2.765678800583      2.249026002580
 Si       0.373178519620     -4.149058812009     -2.082988010435
 Si      -4.608154395930     -2.790745309815      1.011758701559
 N       -1.949671053684      2.030075481744     -1.673593448365
 N        1.579537535860      1.202612759195      1.383594380550
 N        0.967228754882     -2.614930989257     -1.370251006531
 N       -3.161684477337     -1.774701281918      0.912130964966
 C       -1.205345068192      3.242790969787     -1.579760849718
 C        0.028656929074      3.378930052792     -2.247314503964
 C        0.734033405096      4.578137791856     -2.126624769441
 H        1.692317578043      4.676060241132     -2.645990236321
 C        0.250434790897      5.652430308897     -1.374693778260
 C       -0.982295716479      5.499025211164     -0.739247504757
 H       -1.385214783817      6.325582853621     -0.146230382225
 C       -1.720504677366      4.315295192447     -0.827090525336
 C        0.558840838610      2.268440102869     -3.101669892720
 H        1.549384053344      2.518230112613     -3.505184589562
 H       -0.114032273918      2.061767526467     -3.948854367130
 H        0.646483913921      1.327912941053     -2.536146389082
 C        1.051483297596      6.913148618021     -1.227337488574
 H        0.415674611505      7.765823811314     -0.949438763851
 H        1.578626484144      7.167954694428     -2.158701182583
 H        1.816912255140      6.801925173769     -0.440506595876
 C       -3.041006807726      4.189397496528     -0.127840533347
 H       -3.189934293227      5.012079545011      0.584764065346
 H       -3.119286362253      3.235928486915      0.413919135051
 H       -3.879206696079      4.211439379016     -0.843032756182
 C       -3.301085725135      3.494697616571     -3.838250230768
 H       -3.572005501632      4.336552315641     -3.184202608219
 H       -4.050116048364      3.432022302354     -4.643707155116
 H       -2.327485181843      3.729911677389     -4.295211669488
 C       -4.879704570463      1.461074764670     -2.112144592219
 H       -4.834816569257      0.478930663551     -1.616581512297
 H       -5.671686311330      1.413419093004     -2.876986766816
 H       -5.169093808416      2.214098927215     -1.364332499410
 C       -2.784800218719      0.480833097050     -4.106780397212
 H       -1.836783042668      0.683227682853     -4.628067437769
 H       -3.574028429834      0.360738773463     -4.866691703803
 H       -2.688342587157     -0.476203827082     -3.568195330914
 C        2.917626389747      0.684022594521      1.414905555479
 C        3.349705162117     -0.130082946398      2.481254675303
 C        4.702125187201     -0.473561804592      2.558529026959
 H        5.035313762652     -1.094298694194      3.396715359047
 C        5.635079731851     -0.038019346104      1.617545224140
 C        5.167237246938      0.706969648972      0.534699248686
 H        5.869573958987      1.019421355080     -0.243224259926
 C        3.821611205246      1.050936224194      0.396221357325
 C        2.403091893089     -0.653501989000      3.521341346783
 H        2.429889575202     -1.754436489137      3.539930933265
 H        1.368783375839     -0.347154978423      3.328942515362
 H        2.680037558040     -0.306578483349      4.528915686209
 C        7.085160448964     -0.404430941153      1.747615849378
```

H	7.705485725674	0.140120211074	1.021982234930
H	7.242144111894	-1.482842565653	1.576857037696
H	7.467728271568	-0.182186490489	2.755733016316
C	3.347147985052	1.728361759881	-0.853225937966
H	4.175179036072	2.230245839430	-1.373193245587
H	2.549637052383	2.456955670374	-0.667860330834
H	2.940178253515	0.968666562307	-1.540981004379
C	-0.463565366091	3.156650597762	2.273031765644
H	-0.823380651252	3.385799290717	1.260997694279
H	-0.633399670543	4.047962903380	2.898700904112
H	-1.058023365496	2.326136214975	2.682105143875
C	2.020526539768	2.606642189700	3.997918338992
H	1.435453734805	1.890844079175	4.592087235386
H	1.957362640247	3.590541318856	4.490659026425
H	3.075171798247	2.292528600643	4.006999456260
C	2.308158637259	4.133625082988	1.385954620765
H	3.375981621117	3.887071108790	1.284177385715
H	2.228011707963	5.057943400101	1.981132424778
H	1.889865828969	4.334405196557	0.388515020580
C	2.347224508269	-2.276075017243	-1.493130898904
C	3.243325957245	-2.628922864197	-0.466924755198
C	4.601934119769	-2.363372846970	-0.637495653871
H	5.295021533786	-2.644340130119	0.159766076512
C	5.094513536310	-1.736303000323	-1.783526000058
C	4.179803248470	-1.357773297128	-2.767450213185
H	4.539663819878	-0.845088171718	-3.665165697968
C	2.812442826960	-1.618312846460	-2.647030970275
C	2.738597698825	-3.292108729526	0.778364574786
H	3.542529952355	-3.397879456366	1.519330846339
H	2.327283758403	-4.292509013991	0.567380899134
H	1.924265723323	-2.709392801523	1.236656510015
C	6.563489978784	-1.484397978220	-1.962425945215
H	6.751240459796	-0.499242030123	-2.416123190529
H	7.017916521785	-2.237909298569	-2.627333349665
H	7.099020827957	-1.525426942495	-1.003944515853
C	1.865837461852	-1.228477934961	-3.743082891894
H	2.296683170618	-0.438999185037	-4.374733659998
H	0.904385753844	-0.874138270041	-3.343283572252
H	1.637406154972	-2.086028533030	-4.397470305357
C	1.889947321754	-5.068236586912	-2.691908176372
H	2.426482573276	-4.495952213359	-3.463790560644
H	1.590013703888	-6.034880393170	-3.126947468993
H	2.598989952428	-5.264165527917	-1.873235423083
C	-0.517181657584	-5.142463262876	-0.764795587885
H	0.133810709614	-5.351842944200	0.097534613361
H	-0.859727625239	-6.104393474520	-1.179120426081
H	-1.404500797659	-4.601938742861	-0.402958196681
C	-0.817038893556	-3.817172482771	-3.493943301874
H	-1.668412078266	-3.216001322290	-3.135366742349
H	-1.211962925252	-4.767611488138	-3.888235762478
H	-0.336625626287	-3.276139837824	-4.322367191136
C	-2.649264358783	-1.168748004711	2.081241343758
C	-3.147532939594	0.078807273613	2.515838015774
C	-2.634163955704	0.658291541180	3.677200039521
H	-3.036975597999	1.622161342995	4.004582436204
C	-1.625156034116	0.049260680142	4.428207549716
C	-1.141093092466	-1.180629058779	3.981647385123
H	-0.357756960625	-1.686471953690	4.553959026249
C	-1.635822823490	-1.801342029732	2.830147896498
C	-4.226822668747	0.768557650940	1.739449374223
H	-5.193888486476	0.250284871012	1.842303755800
H	-3.997244707792	0.778100009565	0.663020563759
H	-4.364208601888	1.803478135207	2.082555629589

C	-1.100314403539	0.690287292721	5.680511073123
H	-0.944067696504	1.771730431402	5.544922894118
H	-0.143403842743	0.245181272697	5.989734202819
H	-1.805933128149	0.569353407319	6.519426071555
C	-1.109949597222	-3.134311801722	2.402084997469
H	-0.256748852809	-3.447578402143	3.019761527349
H	-0.788168264207	-3.108896142041	1.348289914660
H	-1.885226022010	-3.914488268143	2.462767385946
C	-5.993219407132	-2.089519559755	-0.054730727565
H	-6.287055515885	-1.078686005540	0.265863180983
H	-6.884794465629	-2.736269530844	-0.013902402880
H	-5.669286146647	-2.027875690201	-1.106070487701
C	-5.136136620309	-2.866356896256	2.812426935036
H	-4.336884047103	-3.287260080021	3.441852242972
H	-6.029938940629	-3.501163958548	2.920764916038
H	-5.373571759282	-1.867078515126	3.207870419146
C	-4.247302044700	-4.526102781687	0.377768028339
H	-3.894390595647	-4.489179093106	-0.665176714047
H	-5.157526917832	-5.147204282075	0.403304212448
H	-3.473940908808	-5.027629963874	0.979336435545

140
F(THF), G=-4406.105297

Si	-1.462070337232	0.622839940087	-0.775812642342
Si	0.298730469018	0.338546668362	0.587119706210
Si	-0.093329085832	-1.488675196454	-0.638313094509
Si	-2.458193830663	-1.515374526681	-0.755025173809
Si	-3.244548515968	1.843203973409	-2.929404644649
Si	1.317241837503	2.774730426166	2.259965571068
Si	0.424650791242	-4.146864787866	-2.094334119939
Si	-4.583499910182	-2.832807338258	0.996163491349
N	-1.965424734835	2.017964480183	-1.679608488056
N	1.559626193935	1.220588460631	1.380457957226
N	1.002512659592	-2.609441123037	-1.373880156942
N	-3.142480257398	-1.808503286297	0.903508242699
C	-1.236367314132	3.240074295137	-1.575955483640
C	0.000114560770	3.393862064145	-2.235459685257
C	0.690051702276	4.602122061080	-2.106053763182
H	1.650848538754	4.713675569337	-2.617891232988
C	0.187715768734	5.667785755070	-1.353177373541
C	-1.048047360381	5.496783923780	-0.725966823282
H	-1.465066527655	6.315824603957	-0.132134052380
C	-1.770489179855	4.303360855110	-0.822317623324
C	0.551577364622	2.293729107329	-3.090049721934
H	1.539555575700	2.560294151398	-3.488709184278
H	-0.114170797968	2.077887216427	-3.940486638957
H	0.652524426413	1.353641466967	-2.525897254644
C	0.971713934989	6.937871066270	-1.193554870914
H	0.317400906911	7.790454705169	-0.962078397187
H	1.543584120887	7.176561503038	-2.102262134508
H	1.697850289195	6.847685147195	-0.367737702044
C	-3.093624249539	4.159939336442	-0.131319626963
H	-3.252678577365	4.974407451111	0.588313948195
H	-3.167635101894	3.200159970170	0.399631452018
H	-3.928549074627	4.183418681744	-0.850249949684
C	-3.335136682950	3.477519153318	-3.842857736808
H	-3.621508651381	4.311284025296	-3.184970347483
H	-4.084589186666	3.401764616352	-4.646849758979
H	-2.365266817930	3.724699472115	-4.301653421285
C	-4.884373427652	1.412813538460	-2.128070393514
H	-4.821632330920	0.436963292335	-1.621921647489
H	-5.669740970012	1.344605463962	-2.898182799086
H	-5.189730606453	2.168294009907	-1.388971753228

C	-2.765354150947	0.477173230481	-4.125552631334
H	-1.833424274296	0.716908990153	-4.660009174790
H	-3.562520189440	0.330798291648	-4.872447183821
H	-2.623255819801	-0.474911362732	-3.588483307477
C	2.904418831534	0.717139201817	1.414903240987
C	3.337044747420	-0.108240575894	2.473139967122
C	4.691235546057	-0.445476539478	2.551522289847
H	5.023764545745	-1.078553612607	3.380548937019
C	5.626365284663	0.007663792433	1.619972716926
C	5.159641650032	0.769159552884	0.547596461711
H	5.864318568580	1.097556034403	-0.221718247652
C	3.812070781775	1.108526212782	0.408314677275
C	2.390839571333	-0.650481355512	3.503840334524
H	2.424082792730	-1.751207693416	3.509371900677
H	1.355439228311	-0.346510778346	3.314502252817
H	2.663617566238	-0.315558660384	4.516437538185
C	7.077008229831	-0.356652558415	1.748889162371
H	7.694749715717	0.181317658663	1.016365455021
H	7.231283320728	-1.436469824027	1.587055621467
H	7.461534607139	-0.128154012513	2.754948049552
C	3.344394548133	1.804963017280	-0.833977274274
H	4.160900296523	2.367868985019	-1.307863384684
H	2.503654412844	2.483560373040	-0.651667473274
H	3.000727900973	1.051396652678	-1.562311926300
C	-0.511848658883	3.139483516122	2.281337144247
H	-0.880062737910	3.353984236971	1.268971146203
H	-0.690438272530	4.033026994904	2.901341176507
H	-1.092080976907	2.304335237368	2.701086601474
C	1.972888166865	2.607313565902	4.008846910968
H	1.397607588251	1.875069218958	4.592406076968
H	1.888492051873	3.586155769836	4.508416239394
H	3.032646580057	2.311045048693	4.020499703900
C	2.250934241101	4.160861505442	1.411033986778
H	3.323656179819	3.932241850565	1.318892643222
H	2.148829710591	5.079175431079	2.012119533210
H	1.842172514821	4.361360258664	0.409614133698
C	2.381403707852	-2.260764867251	-1.490579003131
C	3.276658363996	-2.616111057617	-0.464452909753
C	4.634591703179	-2.336598154725	-0.627362196498
H	5.327491532769	-2.618185717300	0.169873261578
C	5.126049150618	-1.694090512218	-1.765661509370
C	4.211002577292	-1.315091967390	-2.750784133823
H	4.569359462582	-0.790333021126	-3.642139623589
C	2.845142020438	-1.587781260785	-2.637284058243
C	2.774191770279	-3.294888801745	0.773438195978
H	3.576039660612	-3.399055774475	1.516734995207
H	2.373463987542	-4.297653351577	0.553261516609
H	1.952719652338	-2.723992520367	1.233688291397
C	6.591782158384	-1.416159993074	-1.933928488394
H	6.769242502623	-0.376957292380	-2.251209582893
H	7.034950898555	-2.066914649510	-2.705811527694
H	7.143389168419	-1.585078105066	-0.999156078574
C	1.900219817982	-1.193633140182	-3.733341947626
H	2.329285398391	-0.396273717992	-4.355937824481
H	0.935809661906	-0.847410608718	-3.333398337057
H	1.678975369157	-2.046595514769	-4.395998400669
C	1.948189385140	-5.063203625091	-2.687766887966
H	2.486677504788	-4.490411658153	-3.458112831543
H	1.648822149948	-6.029634305266	-3.123782739136
H	2.647710735222	-5.259498720111	-1.860941164865
C	-0.480122483828	-5.142261475137	-0.788069428231
H	0.166076207281	-5.356546218702	0.076766894187
H	-0.819377225714	-6.100887121986	-1.212777657996

H	-1.368147167514	-4.600814045089	-0.429150033875
C	-0.745188352816	-3.813719967115	-3.522958643466
H	-1.586680005249	-3.189729542325	-3.180881987267
H	-1.153210938608	-4.763289382533	-3.905772448233
H	-0.242575403857	-3.295070306097	-4.352668373454
C	-2.639803820916	-1.202230631754	2.077459102540
C	-3.152615010920	0.038585640839	2.515598814784
C	-2.651119084324	0.617289101646	3.683314839092
H	-3.063028969701	1.576814369092	4.012106819806
C	-1.638969927344	0.014402983527	4.436414717602
C	-1.139623048790	-1.208494656834	3.985324903266
H	-0.350536893967	-1.706933512606	4.556273750821
C	-1.622923650195	-1.828408537423	2.827630869303
C	-4.232014661974	0.724717612246	1.735803972677
H	-5.195775699853	0.198102765509	1.826742010438
H	-3.992784966094	0.744108228745	0.661502991356
H	-4.380429719370	1.755933474515	2.084923279799
C	-1.121526433165	0.657871479466	5.690784443568
H	-0.966614377126	1.739067001245	5.552799841562
H	-0.166293455262	0.213475624759	6.005512701177
H	-1.832048005688	0.540096812839	6.526004249433
C	-1.079350129087	-3.153387756938	2.395652984734
H	-0.221835519802	-3.456487593837	3.012247036527
H	-0.758084977784	-3.120290484048	1.341928856276
H	-1.844252072003	-3.944128950966	2.454261144052
C	-5.975121337929	-2.139714466007	-0.068024540662
H	-6.272454099568	-1.130809526674	0.255713412020
H	-6.861128939056	-2.793783013653	-0.020042112514
H	-5.657763738264	-2.075914194131	-1.121186288840
C	-5.119554926092	-2.918233380669	2.794232833669
H	-4.320074777546	-3.338577132449	3.424047286181
H	-6.009024777835	-3.560980758257	2.891809155094
H	-5.369339684861	-1.921732907445	3.189515207900
C	-4.214824094000	-4.567846961993	0.364763572939
H	-3.863032705533	-4.535782725533	-0.678705292165
H	-5.124333880585	-5.189915979571	0.396303370675
H	-3.438938893266	-5.061870256742	0.969524276997

35

RH, G=-812.807967

Si	-2.344946891065	-0.025077665241	0.189257835087
N	-0.939883722151	0.377506174046	-0.805065600992
C	0.413271096523	0.179795779605	-0.485517458599
C	1.258976584396	1.296460452706	-0.296421330103
C	2.601957152382	1.096292295648	0.017575669291
H	3.242983502644	1.972108528915	0.160521983884
C	3.146179022581	-0.182161085034	0.168883374911
C	2.297664468825	-1.269758040099	-0.034713159002
H	2.702604358764	-2.283488723945	0.046880109394
C	0.949138400433	-1.118093614296	-0.371003414672
C	0.702367029361	2.684058682711	-0.422527201810
H	1.462487413215	3.439290195203	-0.180620979594
H	-0.156944441224	2.835747855988	0.251792307290
H	0.346466351190	2.899799470985	-1.445952417135
C	4.586489568963	-0.371328126864	0.549692904886
H	4.932063951198	-1.390272389252	0.322647039152
H	4.740951106473	-0.205166008412	1.629332383278
H	5.241905141042	0.335077720166	0.017737773899
C	0.104680358590	-2.324465233972	-0.653260228437
H	0.732084847316	-3.199842007136	-0.871607040127
H	-0.558857444955	-2.140186188351	-1.511343886127
H	-0.540598957661	-2.595276437036	0.198968432886
C	-1.771019056192	-0.385238587686	1.937411984946

```
 H        -1.060278970831     -1.224480731081      1.972158529940
 H        -2.625510246411     -0.636753211530      2.585300069605
 H        -1.262635078883      0.494530343427      2.361782946744
 C        -3.348917264481     -1.455748925453     -0.501628467401
 H        -3.605630934327     -1.269934309143     -1.556604341001
 H        -4.291066938382     -1.562068186703      0.060363733922
 H        -2.815161250498     -2.415187197852     -0.449170713614
 C        -3.437087443087      1.506622328701      0.144997344687
 H        -2.930021629102      2.376289963773      0.591652974815
 H        -4.372801232856      1.337893090039      0.701193129418
 H        -3.715044396687      1.767605623443     -0.889435797614
 H        -1.080752129827      1.234241137771     -1.330686835460

4
NH3, G=-56.424926
 N         0.000000000000      0.000000000000      0.121572790368
 H         0.000000000000      0.936051458008     -0.283669844191
 H        -0.810644341884     -0.468025729004     -0.283669844191
 H         0.810644341884     -0.468025729004     -0.283669844191

3
H2O, G=-76.272658
 O         0.000000000000      0.000000000000      0.119645474026
 H         0.000000000000      0.754088572674     -0.478581896105
 H         0.000000000000     -0.754088572674     -0.478581896105

6
MeOH, G=-115.471293
 C         0.648945108248     -0.019699305749      0.000002420196
 H         1.088756593372      0.989153974293     -0.000937109632
 H         1.036618711746     -0.545178694598      0.895728742833
 H         1.036463235975     -0.546763643046     -0.894849526287
 O        -0.740598332520      0.122226896432      0.000002999907
 H        -1.130722530421     -0.756830973614      0.000019372649

13
PhOH(THF), G=-306.833678
 C        -0.270075380889      1.195969194330     -0.000010471739
 C         1.124007823993      1.220127993474     -0.000028632812
 C         1.855508816376      0.032807793102     -0.000038732108
 C         1.173810576515     -1.186337810854     -0.000016996319
 C        -0.217548883351     -1.223647295836      0.000017798319
 C        -0.947828772082     -0.028672390379      0.000016924941
 H        -0.839716028189      2.130063150625     -0.000015318094
 H         1.640611969437      2.183256641709     -0.000043651018
 H         2.947239016901      0.055925233201     -0.000062050377
 H         1.733370851619     -2.125245395587     -0.000019331651
 H        -0.760346615637     -2.171366121170      0.000044866746
 O        -2.294286112100     -0.113457971686      0.000026838974
 H        -2.674115380708      0.773545361692      0.000241430914

144
NH3_Int_1, G=-4462.531320
 C         3.575859402430      2.858268569086      0.260590634413
 C         3.691627756347      1.540634008958      0.753194763539
 C         3.767066619776      1.331996926961      2.145212209090
 C         3.798159480296      2.432853722957      3.005363531208
 C         3.745885012547      3.744231791731      2.531343585425
 C         3.614679945277      3.930824705317      1.153574148242
 N         3.845131317005      0.454926515939     -0.144637237864
 Si        5.507761850236      0.023702936894     -0.610031434093
 C         5.741945260791      0.142583995065     -2.476125846697
 C         3.862493080686     -0.060479595260      2.686715016456
```

C	3.764575295101	4.914095123137	3.471854983244
C	3.449270428225	3.096844582135	-1.212319709606
C	5.921320825189	-1.745821454235	-0.109703215612
C	6.681225444926	1.214283115341	0.246876398382
Si	2.591444690531	-0.569631483939	-0.937496709735
Si	0.463112882623	0.354685615663	-1.254355578513
N	-0.208803097133	0.704154990705	-2.855909656795
C	-1.569128172454	1.100380006418	-2.914607422441
C	-1.899871288523	2.466582182939	-2.818334956807
C	-3.243818737755	2.847919163323	-2.848067171851
C	-4.280523708356	1.918380266033	-2.944573639892
C	-3.933623566888	0.569429707167	-3.059204993907
C	-2.600391883616	0.146511040428	-3.064743362711
C	-0.810377012610	3.485991509062	-2.683957080662
C	-5.719337752534	2.343086440686	-2.901952317167
C	-2.263462521301	-1.302295592938	-3.246644259652
Si	0.836972857715	-1.403802059555	0.383017442587
N	0.566990734887	-3.128064472365	0.687448564429
C	-0.796861660426	-3.504870002315	0.671439114973
C	-1.423852293103	-3.873153483641	-0.538414877173
C	-2.802729142190	-4.124941335596	-0.541588766101
C	-3.572975522324	-4.047344116176	0.621215639573
C	-2.918517437728	-3.732403732930	1.817534367983
C	-1.549688422064	-3.461189642006	1.865403547028
C	-0.613659030191	-4.018583774553	-1.790285984202
C	-5.052191340628	-4.305378952156	0.601962249728
C	-0.868355741665	-3.118167352910	3.155017300300
Si	-0.860158206873	0.121610745418	0.608506733933
N	-1.657297721002	1.079081431309	1.878256801449
C	-3.052047879367	1.343237100774	1.803912024106
C	-3.546129448441	2.414958589362	1.031286048960
C	-4.926219222342	2.573476882626	0.888620493650
C	-5.842270768232	1.712780837643	1.498851571343
C	-5.335336325230	0.692818381961	2.306634021172
C	-3.960905484568	0.500250759038	2.481051877472
C	-2.601217050444	3.403581865574	0.423573341785
C	-7.318586197491	1.876691207109	1.280871760924
C	-3.451371096265	-0.573732664808	3.394478065967
Si	1.882673597472	-4.318108381391	0.813370720066
C	2.812682983461	-4.541517970537	-0.802303003909
C	1.080324890505	-5.940327629626	1.321112616466
C	3.098107731018	-3.726224850924	2.112396030679
Si	-0.679752325477	1.901941562324	3.125498300132
C	-1.882588580094	2.768065224744	4.280095169898
C	0.338763694503	0.632080324139	4.058327266013
C	0.462434385173	3.150995455167	2.339881795366
Si	0.718921314627	0.426004936501	-4.352957855117
C	1.194717635651	-1.381903004912	-4.527701929666
C	-0.399689673745	0.954754378041	-5.768739495324
C	2.286972782456	1.455005270373	-4.365387095347
H	-3.284523671889	-4.397311300547	-1.485836107412
H	-3.495262194020	-3.693019058498	2.746438525016
H	0.098482731190	-3.187743482660	-1.910231152910
H	-1.255184345415	-4.075888948517	-2.679641605729
H	-0.013912895965	-4.941969431161	-1.754769305988
H	-5.314398817745	-5.173538009255	1.227579075417
H	-5.413271304687	-4.506091931178	-0.416455602280
H	-5.615220631805	-3.444072387101	0.996425406148
H	-1.573052501700	-3.135365808700	3.997504648860
H	-0.403633341158	-2.120762547611	3.104636383898
H	-0.053344366809	-3.825582558436	3.371189846272
H	0.563425423812	-5.862149892144	2.289756840049
H	1.846771658950	-6.726948537099	1.406872673709

H	0.340932173908	-6.270420533543	0.574674934643
H	3.620684070835	-2.824165800154	1.761102100301
H	3.860596241228	-4.497549528633	2.306630689080
H	2.597413421022	-3.484704348759	3.062395309364
H	2.234772673640	-5.105870360990	-1.548873437277
H	3.756873383675	-5.081149908128	-0.623470508096
H	3.054004810625	-3.553988931960	-1.229522347182
H	-5.295715457431	3.404529220652	0.280085969359
H	-6.029844175208	0.026494866752	2.827632262362
H	-1.741889343169	2.908822428738	-0.048543262290
H	-2.196359638645	4.071213599662	1.201050930071
H	-3.104673991853	4.026468927350	-0.325381421710
H	-7.901772610955	1.379032789018	2.068761216790
H	-7.629206635872	1.439716505175	0.316483670147
H	-7.607420484244	2.938175051572	1.261441475687
H	-3.010578231550	-0.137158165294	4.305237982883
H	-2.651910723121	-1.163042157316	2.927578620693
H	-4.258357867288	-1.252756114184	3.702162339447
H	-0.299164473712	-0.092272709564	4.587387867780
H	0.985811332168	1.131517181297	4.797198388363
H	0.991058914508	0.080777906970	3.364133895233
H	1.155576503350	2.662439318366	1.637796612281
H	1.074830097759	3.641769610904	3.110253383641
H	-0.087815969389	3.928548897236	1.791717428467
H	-2.489239755461	3.513203246980	3.742922204286
H	-1.316952282515	3.295194487796	5.065029723349
H	-2.573965005526	2.068345761732	4.772925379714
H	-3.488750091798	3.912376750978	-2.782100031347
H	-4.724359500189	-0.180744003272	-3.163881198273
H	-0.162691073584	3.268230256411	-1.818891297709
H	-1.222450653592	4.497207596169	-2.563709929408
H	-0.150752032420	3.483222189354	-3.565852740905
H	-6.328466017573	1.786231045654	-3.629743142254
H	-5.829554102974	3.415823853362	-3.115524148375
H	-6.151676159597	2.158564791236	-1.904021849066
H	-1.395775857145	-1.586980734547	-2.636241139304
H	-1.987397810270	-1.513730067876	-4.292564438518
H	-3.115053577058	-1.951254322416	-2.995175651195
H	-1.326210716044	0.361839838157	-5.807643862022
H	0.127192291946	0.824411743258	-6.727444623965
H	-0.688137492265	2.013671994353	-5.680433929546
H	2.077722068187	2.527152407251	-4.231723208599
H	2.810857764444	1.323660665321	-5.326137126262
H	2.969325194727	1.133007397986	-3.564856160424
H	1.780212139433	-1.693594888236	-3.646638483726
H	1.823495508959	-1.526294259647	-5.421304114622
H	0.317965340692	-2.040390630457	-4.616062570112
H	3.878016745386	2.254735100882	4.082778018209
H	3.545055401885	4.948224286508	0.755383127429
H	3.681797983624	-0.081759072546	3.770530613412
H	4.855963922646	-0.500900806956	2.502482399374
H	3.131440327430	-0.715194788512	2.189984424913
H	2.740780808559	5.252585666396	3.710335035410
H	4.293047446914	5.774869144155	3.035490100186
H	4.254371520676	4.660095330306	4.423304466626
H	4.367928941950	2.815842447337	-1.751409974225
H	3.238021219252	4.153762308416	-1.427928120574
H	2.642375833389	2.481262244604	-1.637301925599
H	5.881400501753	-1.881618918815	0.981768366593
H	6.933935477335	-2.017149790307	-0.450620025445
H	5.209666178950	-2.452254880744	-0.564660320312
H	6.474871815231	2.256615200981	-0.040696332544
H	7.721409275161	0.978472464335	-0.029605768493

H	6.592366366872	1.153271826016	1.342211453707
H	5.039590908070	-0.527372352085	-2.996333692366
H	6.766704379743	-0.152184052262	-2.756066563909
H	5.568357100293	1.165684118487	-2.843449810459
N	-2.499711842508	-0.717554049384	-0.035736261578
H	-3.250165779645	-0.726605737637	0.663147061601
H	-2.858673883744	-0.218249843900	-0.861383448635
H	-2.300473588537	-1.686281853352	-0.316375276762

148
NH3_Int_2, G=-4518.948636

C	3.208825225440	1.056491211250	2.624157735440
C	3.340025118081	-0.141882950664	1.892572157775
C	3.101035137876	-1.371466971532	2.540855156781
C	2.712323307583	-1.378599688541	3.882053013296
C	2.553199952263	-0.200365928277	4.613893217538
C	2.818739109366	1.006118627973	3.964194515010
N	3.771229159524	-0.115559001168	0.544257624279
Si	5.516694856686	-0.150943807367	0.232478676092
C	6.104097490126	1.370049261658	-0.710863762158
C	3.325609495042	-2.655994046801	1.805859921367
C	2.079221547793	-0.227388583675	6.037849423709
C	3.545865722425	2.364291047968	1.978622315850
C	6.022707021545	-1.635856346813	-0.810523718860
C	6.397071035409	-0.234780424816	1.892080458016
Si	2.671722224485	-0.072548144019	-0.903939762808
Si	0.707084365048	1.219329813742	-0.721120181670
N	0.460322076102	2.805529789540	-1.509287020622
C	-0.583804287403	3.679226210612	-1.080865465624
C	-0.481950019328	4.335144989631	0.167878587434
C	-1.458333619942	5.256923330582	0.546799984212
C	-2.547318410792	5.564036008887	-0.270820320586
C	-2.638145397491	4.902960469286	-1.494297440514
C	-1.686071645640	3.967577257290	-1.912839810724
C	0.675116093324	4.058871097663	1.073635630417
C	-3.599486624923	6.537627652142	0.172892709284
C	-1.865555998754	3.302903770052	-3.245434769554
Si	0.666363597226	-1.276784243949	-0.712409318742
N	0.339181171878	-2.855357981657	-1.458712383793
C	-0.828698756054	-3.597478088036	-1.127036491223
C	-1.935801273125	-3.652805645835	-1.998666908622
C	-3.027271622866	-4.466945481178	-1.671345889019
C	-3.054712081688	-5.243892924419	-0.515407217651
C	-1.952866517076	-5.168769296520	0.341413606088
C	-0.853110029991	-4.357465464792	0.065324460607
C	-1.937075754058	-2.912024252474	-3.302555070233
C	-4.238053070825	-6.102336659807	-0.176124561474
C	0.308701623885	-4.307207599667	1.005948048733
Si	-1.044469297121	-0.000131389961	0.098473519733
N	-2.231649284485	0.027461622847	1.415446826567
C	-3.625185416356	0.082533588320	1.131264938313
C	-4.258246128699	1.325990553893	0.929558943470
C	-5.608391032328	1.354733072252	0.567883045032
C	-6.360245277863	0.187533347420	0.414619133219
C	-5.719340675093	-1.032267872698	0.643285953053
C	-4.371150101897	-1.106414848273	1.003626318293
C	-3.489067227720	2.596546239032	1.106212301238
C	-7.817105942038	0.243765965526	0.056112613199
C	-3.721719522167	-2.430173584915	1.253631400609
Si	1.522065399365	-3.604162171186	-2.568024568573
C	1.777749989350	-2.486195315006	-4.058213302053
C	0.843648258113	-5.279009335152	-3.083136746439
C	3.178782129918	-3.876200795639	-1.736611695015

Si	-1.702924225655	0.000586702838	3.119083338780
C	-3.255017168661	0.088470341830	4.172034327458
C	-0.746335049513	-1.572390066335	3.466524417490
C	-0.595642490741	1.470123314938	3.468852400254
Si	1.753364025364	3.520144750360	-2.524667530090
C	2.083976491021	2.413582324838	-4.007856097156
C	1.185964937558	5.224736583388	-3.077096867814
C	3.366442450746	3.730462691063	-1.591806229455
H	-3.876865738443	-4.504644678984	-2.360557696944
H	-1.947583231383	-5.762410045548	1.260780529512
H	-1.399603742805	-1.960644358637	-3.231353397781
H	-2.962814940250	-2.719282027017	-3.650192315794
H	-1.435463516081	-3.496857304717	-4.091675220652
H	-3.925248750797	-7.083768105979	0.211096386571
H	-4.880282748377	-6.270504398303	-1.052329086658
H	-4.860713376622	-5.633435667863	0.605166835071
H	0.128135592626	-4.925162775614	1.895946367898
H	0.503759124706	-3.277014398704	1.341696597949
H	1.233702298658	-4.654194386323	0.521545488228
H	0.670216386887	-5.914917631961	-2.200999257485
H	1.581892668838	-5.784867663114	-3.726044935964
H	-0.103859032437	-5.216088329008	-3.637043185071
H	3.588654881646	-2.928195433141	-1.362050533887
H	3.897804775050	-4.289086732610	-2.462704362386
H	3.103611923738	-4.580696160885	-0.894480001668
H	0.849670424830	-2.353727024718	-4.636119675387
H	2.538188092580	-2.905936338913	-4.736048807763
H	2.142934332819	-1.501980205987	-3.718520163000
H	-6.086063440082	2.325945428186	0.405255322609
H	-6.284797718370	-1.963346421121	0.537234987353
H	-2.579547737851	2.627291165641	0.487101150265
H	-3.146270217095	2.711033866442	2.146331309728
H	-4.100419103614	3.470519038659	0.850256075087
H	-8.129759359428	-0.647844178974	-0.506960198902
H	-8.050550695233	1.131339428936	-0.549985268904
H	-8.445179918928	0.293429140631	0.961569163912
H	-3.452375775415	-2.545297167642	2.315481674677
H	-2.783985048968	-2.549057663612	0.690768424055
H	-4.389616478131	-3.257182205741	0.983613021877
H	-1.328250932320	-2.471529334034	3.216606346498
H	-0.457653179710	-1.625659710394	4.528341076471
H	0.182117329462	-1.581838114198	2.876005630673
H	0.338071554223	1.387158076593	2.892885586595
H	-0.318973048167	1.499828731169	4.534907274632
H	-1.084311990213	2.420651524418	3.208435659125
H	-3.825247646807	1.009191828879	3.975164060885
H	-2.975991358562	0.081451200460	5.237849423158
H	-3.926878982608	-0.763743863737	3.989756137885
H	-1.356415874912	5.757117229454	1.514749694356
H	-3.479065019826	5.124614889493	-2.158834171792
H	0.756607103815	2.984759712903	1.305176727476
H	0.573942092960	4.598535229136	2.024879614730
H	1.629131873549	4.349316398309	0.611018121011
H	-4.164498031470	6.939789425498	-0.680273256191
H	-3.160506948885	7.383861893729	0.721996664035
H	-4.325414575009	6.056021898202	0.850696864804
H	-2.214780213681	2.265271617913	-3.135315242625
H	-0.927817794168	3.263474066562	-3.817346829425
H	-2.609913201105	3.838275977104	-3.850770657584
H	0.285518237277	5.206650655366	-3.707609924936
H	1.996835333448	5.694964562966	-3.656746728377
H	0.972678692503	5.867434988446	-2.208963772188
H	3.284946515477	4.471228214432	-0.782320614940

H	4.151416710985	4.073633479141	-2.285124403206
H	3.697937053216	2.775451004011	-1.161770439901
H	2.378367328369	1.407800114148	-3.664598262897
H	2.920766697949	2.824918074675	-4.595196726954
H	1.217171540636	2.323757423345	-4.680769330894
H	2.531038578575	-2.340930059164	4.371337148158
H	2.720095924405	1.945261008737	4.518024765771
H	2.943663146782	-3.515347173529	2.374167485313
H	4.399049336914	-2.825009151365	1.619962990054
H	2.841681724583	-2.635623562379	0.820107466721
H	0.978980228212	-0.149781687383	6.092946636954
H	2.491011264070	0.611494987606	6.618448518600
H	2.362921001703	-1.163158107462	6.541769632543
H	4.637186925150	2.488439013480	1.879063221450
H	3.167116651742	3.211704765156	2.566702036827
H	3.132990967668	2.417349949044	0.963373548443
H	5.768523728406	-2.590932682894	-0.327292167184
H	7.111409259583	-1.618462474244	-0.983823560135
H	5.524463139824	-1.608048139603	-1.792072524957
H	6.165852568740	0.638529124887	2.520603747723
H	7.487230020614	-0.266930573334	1.735295576631
H	6.104775950638	-1.131780029064	2.459122246316
H	5.611360128611	1.429171815519	-1.693808358082
H	7.191573972235	1.308614451626	-0.881668792446
H	5.894789725494	2.304327268704	-0.169195561911
N	-2.346701943295	-0.071427610305	-1.338489767501
H	-2.840591780801	-0.969088209165	-1.331091458352
H	-3.068149888812	0.651426905622	-1.257076039954
H	-1.832524540129	0.028445756732	-2.259331686303
H	-0.266274408802	1.145323978471	-3.352562545360
H	0.003642515233	-0.412664341289	-3.694269173226
H	-1.018070797276	0.456221105571	-4.641942391811
N	-0.730970285499	0.295358929625	-3.676564299060

148
NH3_TS_1, G=-4518.926618

C	3.555693865331	0.898554936625	2.589965224203
C	3.601766168311	-0.293470837663	1.838312523320
C	3.512623713257	-1.531111942807	2.509018157036
C	3.406244008973	-1.553951524092	3.900932182730
C	3.360068498844	-0.384463545813	4.660575314268
C	3.428215812462	0.831157538743	3.979496274955
N	3.825587244960	-0.252330499204	0.433009337618
Si	5.507928284667	-0.337871968898	-0.132073431684
C	5.986483776407	1.187019635592	-1.130495194661
C	3.539916987697	-2.810787815525	1.733567083095
C	3.191334973457	-0.431309027724	6.151436857637
C	3.679681513477	2.225436082011	1.909498971455
C	5.786991646785	-1.817088850701	-1.266938851878
C	6.619079784144	-0.468319316883	1.375552456206
Si	2.562102176293	-0.126890284445	-0.839625830789
Si	0.735528482261	1.253160109920	-0.318018318683
N	0.365339909081	2.533977394332	-1.570247576129
C	-0.829863788547	3.298056283501	-1.344324055973
C	-0.913192802195	4.202921454212	-0.261335762539
C	-2.064117860721	4.978083092253	-0.100293478290
C	-3.148024934230	4.894834230935	-0.973623858005
C	-3.044637248930	4.004621281630	-2.042535431800
C	-1.910168543171	3.215033455443	-2.246708262344
C	0.227186728979	4.387831286234	0.692242713538
C	-4.391910988938	5.703379072075	-0.750074593458
C	-1.857913366905	2.291623186307	-3.424488303244
Si	0.585127231207	-1.324619735374	-0.420916684776

N	0.048983498450	-2.410001996420	-1.808547915311
C	-1.272208866681	-2.955462474135	-1.677255005382
C	-2.271592935086	-2.658241584748	-2.628176132578
C	-3.568390777261	-3.146805461599	-2.445325736946
C	-3.914219188909	-3.951625066860	-1.360227907877
C	-2.894389555514	-4.304749576084	-0.477632045248
C	-1.587382021103	-3.832086209904	-0.613485909599
C	-1.985559037935	-1.808278078716	-3.829411701109
C	-5.328513067600	-4.396598489481	-1.133263493040
C	-0.540779779911	-4.294157865272	0.355012240266
Si	-1.142662058104	0.048922818247	0.279033678662
N	-1.947360790171	0.096818896198	1.851354236993
C	-3.362503148803	-0.072687317304	1.843699033847
C	-4.195261887652	1.043337530923	1.618719269849
C	-5.577685860590	0.865470918715	1.552193273073
C	-6.170253239502	-0.390591067174	1.711378411752
C	-5.331628956590	-1.474763414800	1.970188173759
C	-3.941494104969	-1.339076902095	2.046314262615
C	-3.590830656008	2.404705076812	1.467689941909
C	-7.657271308281	-0.563513602312	1.593013791396
C	-3.086690689678	-2.520352952042	2.385520643537
Si	1.228306533093	-3.445557370360	-2.683741541365
C	2.039133773250	-2.532292005906	-4.123652140912
C	0.279587558479	-4.908753316666	-3.382050576022
C	2.637431578784	-4.052047685583	-1.607131798508
Si	-1.106119959560	0.269268474868	3.409978471937
C	-2.430620011831	0.376520316205	4.739386414923
C	0.027805651419	-1.190766826772	3.702469645024
C	-0.073115389019	1.835339683698	3.447447697544
Si	1.665949396034	3.433121420822	-2.425304863036
C	2.283411597450	2.534223216254	-3.968379374563
C	0.936895921794	5.067803695166	-2.996847767634
C	3.189910518725	3.739474926758	-1.377764929219
H	-4.333834560826	-2.885331527347	-3.182338228955
H	-3.120023829136	-4.975232680162	0.356311890782
H	-1.045548675293	-2.097162779398	-4.323138001101
H	-1.922683904244	-0.747971478422	-3.540083377352
H	-2.791878295839	-1.897430458170	-4.570011661971
H	-5.369893408085	-5.386018074381	-0.654416497036
H	-5.895564129991	-4.446148910351	-2.074138491322
H	-5.852420172166	-3.688188913264	-0.468972164036
H	-0.988084962818	-4.896432590539	1.157033047952
H	-0.005018744797	-3.449206834900	0.824857326800
H	0.221754246843	-4.912120457914	-0.144255383359
H	-0.180166107738	-5.514208540885	-2.586546097363
H	0.970865365731	-5.553555641412	-3.948111095787
H	-0.527353067436	-4.593624731367	-4.060648412469
H	3.241988182377	-3.210063984996	-1.242863956764
H	3.292423731753	-4.701351324527	-2.210909183171
H	2.294761472432	-4.628262044839	-0.736289574977
H	1.323757361794	-2.155715682802	-4.872169412049
H	2.698485816349	-3.245429569396	-4.645029070889
H	2.675132532703	-1.707534088064	-3.763334237707
H	-6.212293433738	1.739332946600	1.373276269072
H	-5.770557364255	-2.465665374915	2.125040472420
H	-2.869329546198	2.457422179037	0.638379229089
H	-3.024378359803	2.691187060743	2.367751221898
H	-4.361859983308	3.166255044638	1.289831016405
H	-7.988989194098	-1.515109238692	2.032816550666
H	-7.976532322734	-0.559049234829	0.537083766962
H	-8.199764526004	0.251069627335	2.096655354546
H	-2.778201400508	-2.488214104325	3.443426249571
H	-2.167171449654	-2.533947457004	1.786706564108

H	-3.629816469837	-3.461038937285	2.227560704252
H	-0.517758618800	-2.144965696620	3.716332226919
H	0.563843391315	-1.081391457828	4.657249534083
H	0.788105939857	-1.240991098576	2.907700112124
H	0.748808977685	1.771761278063	2.719290297649
H	0.375174315566	1.960432440155	4.446335664471
H	-0.676358059400	2.726516251946	3.218090549773
H	-3.098073328162	1.235608925645	4.569734269722
H	-1.953134958370	0.504712768304	5.724125781578
H	-3.058251739842	-0.526085704572	4.778975661297
H	-2.110282706205	5.672033568689	0.744412373326
H	-3.871753146856	3.925262199262	-2.754675872461
H	0.647318250539	3.425738494016	1.028152456876
H	-0.093146370665	4.942850071596	1.584103495130
H	1.050986197583	4.951763843256	0.226777210831
H	-4.912671671759	5.915282355289	-1.695229213042
H	-4.168137962537	6.661759884605	-0.259220000227
H	-5.102188835799	5.163385299359	-0.100535528996
H	-1.880147779142	1.251399966848	-3.060976235928
H	-0.950285543717	2.450769830736	-4.028171637931
H	-2.726049253935	2.439387026675	-4.081012860253
H	0.065708289812	4.920337695835	-3.653205651100
H	1.701214372842	5.625722084120	-3.561457430264
H	0.608033808801	5.693056568546	-2.153671769599
H	2.981815004144	4.338650849341	-0.480737207572
H	3.935149838029	4.278836131091	-1.984961830627
H	3.641381052482	2.790541292195	-1.057958734659
H	2.833166298924	1.614571071255	-3.712430980388
H	2.997664622339	3.205871418441	-4.472535610811
H	1.490048398108	2.310670026654	-4.699410019309
H	3.343853059929	-2.523175982327	4.405600509293
H	3.394448198882	1.766287237356	4.547454777475
H	3.329042283103	-3.672860025452	2.381773846937
H	4.515428546314	-2.979809338182	1.250597429857
H	2.789467803334	-2.780184847107	0.930011856876
H	2.123212139908	-0.430028039126	6.431614983250
H	3.654260346958	0.439369223780	6.638937930052
H	3.636855723981	-1.340788683449	6.580822557970
H	4.680770995692	2.364025048255	1.470299504779
H	3.497988050085	3.051934648715	2.610768872165
H	2.960017636706	2.299023164466	1.080887456147
H	5.603918742008	-2.774059630249	-0.755866096297
H	6.824686657533	-1.821588378744	-1.639000671070
H	5.115267175923	-1.763420416221	-2.138307206491
H	6.490276381075	0.398703666509	2.041517547853
H	7.674314152693	-0.509598505239	1.061442654071
H	6.398197064611	-1.369924845380	1.966542801420
H	5.330252442520	1.296739071536	-2.008085406002
H	7.023590005489	1.093128277436	-1.492409355977
H	5.915373097332	2.109636725442	-0.535144114289
N	-2.379168669121	0.087520370985	-0.974537688632
H	-2.858867297205	-0.805892754168	-1.086843370708
H	-3.095018243197	0.799512361144	-0.837953344009
H	0.141491502342	-0.787721260292	-2.902021607217
H	0.197278973362	0.934010183763	-2.856883114606
H	1.588916278864	0.028986371893	-2.945428755227
H	0.550205160480	0.105925977265	-4.310309253396
N	0.594487944759	0.074175521305	-3.292812467119

148

NH3_Int_3, G=-4518.932264

C	3.556472657893	0.926527325771	2.588804644714
C	3.598453453449	-0.271114890981	1.845930762772

C	3.498472009568	-1.503319393425	2.524795415371
C	3.381673097797	-1.515301191612	3.916001096178
C	3.337783038145	-0.340106999821	4.666943131976
C	3.421163627221	0.870076061213	3.977949535553
N	3.826675644719	-0.240839721911	0.441047500596
Si	5.510304677656	-0.330667396469	-0.119022322920
C	5.991736189198	1.186337917393	-1.128117625488
C	3.524829135342	-2.788852100483	1.759025469308
C	3.155694647417	-0.374991759935	6.156583094834
C	3.687383874036	2.247733018198	1.898733118024
C	5.792248947475	-1.818540226732	-1.241769653698
C	6.617160551119	-0.451200736981	1.392554636486
Si	2.565184322112	-0.121948641768	-0.834239826809
Si	0.730759702061	1.250599668288	-0.323365828324
N	0.364323310195	2.531331767900	-1.574904729878
C	-0.831937914386	3.294691890710	-1.353312005247
C	-0.917485215718	4.202090651619	-0.272756688431
C	-2.067637545756	4.979560467907	-0.117841391218
C	-3.149540169665	4.893822838400	-0.993464030988
C	-3.044983264797	3.998890334967	-2.058365883426
C	-1.910456605468	3.208087783191	-2.257358085709
C	0.218384700573	4.382469397115	0.687060676788
C	-4.393592270254	5.703638053860	-0.775414863537
C	-1.855989518800	2.278896432933	-3.430525389647
Si	0.590307394699	-1.323730391218	-0.419481154272
N	0.061077355616	-2.418658675672	-1.801842066573
C	-1.258915650338	-2.967416301912	-1.672303783823
C	-2.255921882851	-2.677629254500	-2.628025743921
C	-3.550876607585	-3.172621723967	-2.449447740560
C	-3.896978716072	-3.976792980575	-1.363966633988
C	-2.879481707929	-4.320761038290	-0.475126074602
C	-1.574726698666	-3.840582553744	-0.605901994586
C	-1.969510256871	-1.828780014703	-3.829974500023
C	-5.309579298939	-4.429928201779	-1.142493861941
C	-0.531507712295	-4.289389956179	0.372364704998
Si	-1.144316803785	0.041291066970	0.276343490311
N	-1.952716549664	0.080410728601	1.847528053232
C	-3.370154644465	-0.068135383442	1.836870439172
C	-4.185991046263	1.061060072992	1.614171437509
C	-5.570762875696	0.904842899684	1.546441749053
C	-6.182830451630	-0.342415245428	1.702207381126
C	-5.361033961112	-1.439932904273	1.957473140463
C	-3.968798332313	-1.325975848931	2.034773766810
C	-3.560561860497	2.412952268054	1.465337024132
C	-7.672772694068	-0.490300032360	1.586735749254
C	-3.133573928840	-2.522384370852	2.369520930904
Si	1.246493962541	-3.455301520885	-2.667489565547
C	2.061063532780	-2.545286665893	-4.107306962978
C	0.304262405622	-4.923684507693	-3.363390891424
C	2.652545339415	-4.053454754709	-1.582206906170
Si	-1.111962196870	0.231658586819	3.408639012019
C	-2.436144032587	0.357015844283	4.736687242114
C	-0.006457736204	-1.251442295066	3.698529292269
C	-0.054277696368	1.780638745120	3.450271567541
Si	1.663557398633	3.428436172097	-2.433877106489
C	2.279001175680	2.527055644152	-3.976453095826
C	0.933380123071	5.061888149167	-3.007475062990
C	3.189932126395	3.738757582928	-1.391029006026
H	-4.314515698628	-2.917198430588	-3.190437035370
H	-3.105315970748	-4.989238822058	0.360366275306
H	-1.027814124448	-2.116035019115	-4.321480176902
H	-1.910092233659	-0.767867202311	-3.542032686343
H	-2.774063358931	-1.921089767636	-4.572109296667

H	-5.347087456352	-5.419025481400	-0.662662024791
H	-5.872307194231	-4.483974653318	-2.085707131127
H	-5.840796422937	-3.723961573413	-0.481395426658
H	-0.979197971678	-4.894684352018	1.171912586104
H	-0.009812943278	-3.437187754760	0.844966704017
H	0.242529667998	-4.899667966175	-0.118399102399
H	-0.157471501839	-5.526461375881	-2.566984530402
H	0.999339731955	-5.569499038387	-3.923606406083
H	-0.500525103654	-4.613358753123	-4.046754390583
H	3.249831425100	-3.207847672968	-1.214413474913
H	3.315087144820	-4.698924715494	-2.181822207985
H	2.308177013001	-4.631607874444	-0.713310939040
H	1.348010016388	-2.174691327756	-4.861042980268
H	2.725761872681	-3.258094806408	-4.622296103647
H	2.692099243466	-1.716575512690	-3.747300116731
H	-6.191572082190	1.788974972396	1.369467874312
H	-5.815206307997	-2.424584813803	2.108197306858
H	-2.846104441893	2.458076820418	0.629420025773
H	-2.981255313715	2.685158001933	2.361550176356
H	-4.320183479020	3.188092468430	1.297636706055
H	-8.013551522097	-1.458688066545	1.980333608245
H	-8.000025948907	-0.426783180084	0.535314881178
H	-8.200747883294	0.304177272171	2.136246732719
H	-2.833871327483	-2.505178312943	3.430352325902
H	-2.209567524006	-2.543293115062	1.777991888216
H	-3.688704635460	-3.453643860062	2.197864735621
H	-0.575229613673	-2.191457467851	3.733521127876
H	0.549699337818	-1.144076173580	4.641940450234
H	0.737295309837	-1.328270205631	2.890313626542
H	0.764964678339	1.708391738087	2.719900430299
H	0.398669358278	1.895732648576	4.448168886669
H	-0.645610525425	2.681102674427	3.225860187756
H	-3.087669072174	1.228714179984	4.569485123802
H	-1.957889630053	0.472629013811	5.722619896387
H	-3.079956334683	-0.534317172873	4.771657791968
H	-2.115676577902	5.676042267995	0.724670739607
H	-3.871063744640	3.916800972486	-2.771404961631
H	0.620618707725	3.417861293920	1.038130022927
H	-0.100612539005	4.953330315079	1.569388124382
H	1.055033645786	4.927136201288	0.221810082354
H	-4.909363712211	5.917169250908	-1.722956435522
H	-4.171234874003	6.661206608430	-0.282371359314
H	-5.107817893682	5.163606460477	-0.130200627380
H	-1.876739150753	1.240379990321	-3.061754552484
H	-0.948308075557	2.436689368020	-4.034515415419
H	-2.724026041334	2.421767833926	-4.088273937068
H	0.061406606051	4.913224146469	-3.662506561869
H	1.696949954682	5.619080552879	-3.573810337384
H	0.605385151518	5.688307638424	-2.164807628053
H	2.984223131025	4.341549512279	-0.495829812751
H	3.933010405248	4.276279497361	-2.002514984272
H	3.643003184711	2.791416131682	-1.068842328436
H	2.832133591947	1.609654469364	-3.719802993509
H	2.989849728015	3.199374822146	-4.484498060328
H	1.484011929107	2.299447768772	-4.704448255089
H	3.309448350850	-2.480455532975	4.427125668402
H	3.390186842303	1.809444586361	4.538990600183
H	3.310672318031	-3.645660626650	2.413113603104
H	4.501067313282	-2.963158668323	1.279438802085
H	2.776291416288	-2.762891910413	0.953360349358
H	2.085133681499	-0.362240641736	6.427093858749
H	3.621901222744	0.495041545904	6.642120445817
H	3.589329039766	-1.285263797644	6.596349244176

H	4.687336855980	2.376829459078	1.454296473402
H	3.513784910564	3.080249729090	2.594904732426
H	2.964683911662	2.320338185985	1.072645641271
H	5.607152874363	-2.771443529723	-0.723850871698
H	6.831208553226	-1.826293876326	-1.610250337642
H	5.123569795330	-1.771512732228	-2.115847507029
H	6.487969307703	0.420733446498	2.051970838744
H	7.673220167731	-0.496660322099	1.081823080310
H	6.392872297091	-1.348221242448	1.989266262585
H	5.336333283856	1.290160710797	-2.007062183142
H	7.029021292911	1.088526569940	-1.488468973055
H	5.921212336131	2.113584428567	-0.539948402590
N	-2.379788570950	0.075898804582	-0.978721665118
H	-2.856826063303	-0.819091588646	-1.090104193539
H	-3.098168557358	0.785489764047	-0.842526414965
H	0.154189311049	-0.802919034379	-2.902402016093
H	0.198520313177	0.918686643586	-2.865712308487
H	1.596782764689	0.022988019655	-2.942442164094
H	0.564403813715	0.086953174741	-4.313132950937
N	0.603720185733	0.060116943537	-3.295259931195

148
NH3_TS_2, G=-4518.935745

C	3.486843962219	0.983721571498	2.627904429653
C	3.578943298037	-0.200774312005	1.871326971596
C	3.549702377143	-1.442427417200	2.538512776666
C	3.478370530020	-1.475892790628	3.931753783860
C	3.403563286801	-0.311828343666	4.697916298436
C	3.399539096660	0.907214128571	4.020216205188
N	3.777335285615	-0.145770219466	0.460726840316
Si	5.447550764353	-0.164566621913	-0.140918589496
C	5.839102221092	1.398822881916	-1.117899367552
C	3.574414439995	-2.714668061419	1.753420809461
C	3.270288168519	-0.373407003194	6.191877380200
C	3.498095782149	2.314088974871	1.945542676490
C	5.735369911341	-1.620553801478	-1.303770345879
C	6.598743540026	-0.282555348334	1.335911861668
Si	2.475520490201	-0.050092119354	-0.765138175060
Si	0.549481140371	1.229775154470	-0.624983744420
N	0.383232000246	2.826588314378	-1.396445030683
C	-0.917225466894	3.415359479409	-1.346964811853
C	-1.238946218623	4.340533410551	-0.334013015973
C	-2.498334924508	4.944514528977	-0.341398749288
C	-3.463423597202	4.636102217279	-1.301198234527
C	-3.129714833056	3.702021254219	-2.284537720801
C	-1.873164303531	3.094579496011	-2.329912217952
C	-0.246927834915	4.673187252052	0.738707761379
C	-4.833747794751	5.245711791469	-1.244655601098
C	-1.536238318297	2.109789710815	-3.406299258901
Si	0.493745396661	-1.238152357284	-0.742990877056
N	0.199581167584	-2.713460320292	-1.707978765501
C	-1.162851801390	-3.143525264188	-1.674431013818
C	-2.059685195589	-2.743414558936	-2.681603171876
C	-3.400128827997	-3.130007709355	-2.597163415250
C	-3.877869435045	-3.912641812340	-1.544139238312
C	-2.961688606761	-4.329317910401	-0.576032517098
C	-1.617563585439	-3.955464831837	-0.615548389870
C	-1.588975384125	-1.870693465224	-3.803136740895
C	-5.333664178338	-4.256770067525	-1.423344612328
C	-0.670600295465	-4.394466749284	0.460086577600
Si	-1.235044663355	-0.006295431284	0.176077310087
N	-1.977003369059	0.002417648832	1.786947765236
C	-3.394634369906	-0.116772457403	1.853336590228

C	-4.201953684063	1.025645126086	1.673090971111
C	-5.591152935334	0.895312853161	1.687540642533
C	-6.216546810726	-0.339202214637	1.883824697977
C	-5.401348214209	-1.451748425718	2.092207317526
C	-4.005501276038	-1.362846636812	2.087183336393
C	-3.563166680669	2.364376282806	1.474685402956
C	-7.713119623428	-0.460552593598	1.854206204049
C	-3.171727953843	-2.575182074040	2.360955424098
Si	1.393637412666	-3.804988903362	-2.444288894697
C	2.312540200098	-3.035727511510	-3.905704471333
C	0.447785443884	-5.294989500715	-3.084860478760
C	2.742121481678	-4.329958112527	-1.254635403548
Si	-1.049976686921	0.152454013348	3.293469519000
C	-2.281968291498	0.246741452722	4.709644898424
C	0.093313412212	-1.317597728530	3.491724173440
C	-0.025269450273	1.725003163137	3.276714174703
Si	1.674237279258	3.812330239843	-2.125857411224
C	2.504934703342	2.980627929792	-3.603420740076
C	0.875607931803	5.390132572593	-2.759281607438
C	3.069300084603	4.207461571616	-0.938333379050
H	-4.093673804206	-2.798409695552	-3.375880132943
H	-3.307695666610	-4.955671725681	0.251066511098
H	-0.736396816852	-2.320306371455	-4.336807445060
H	-1.275487959559	-0.894072784708	-3.401180696273
H	-2.390671365372	-1.690085840293	-4.531993828941
H	-5.477672117245	-5.280682872680	-1.047181626537
H	-5.853028965467	-4.170005292457	-2.388455399065
H	-5.834139853703	-3.575035774641	-0.714635528087
H	-1.200227521244	-4.930128951167	1.259250405041
H	-0.146983071079	-3.533756774300	0.909262488305
H	0.109458527830	-5.063822474989	0.064619640104
H	-0.070104315742	-5.833062962520	-2.277013720212
H	1.144944093304	-5.994963033912	-3.572437449402
H	-0.315292987725	-5.000607494341	-3.821734594606
H	3.359556680619	-3.465287330368	-0.973771384556
H	3.398494191515	-5.064017683353	-1.749604199861
H	2.350346040993	-4.784455307679	-0.333748601330
H	1.649351841028	-2.689302022028	-4.714512312186
H	2.966003833827	-3.811733292216	-4.336970428216
H	2.964803373676	-2.211924736048	-3.577462236232
H	-6.204502439235	1.790654659344	1.543542472986
H	-5.863779761630	-2.428156854479	2.269331047664
H	-2.908905854748	2.390309968333	0.589866528391
H	-2.919182978878	2.631313071544	2.327217628986
H	-4.317213523176	3.153366836379	1.352078451249
H	-8.051473238187	-1.395510324433	2.323729556938
H	-8.093810452326	-0.455151723554	0.818794231378
H	-8.196424308454	0.377564671996	2.379095763578
H	-2.785916750899	-2.564259994677	3.393712788632
H	-2.299547956438	-2.612357230585	1.697065509708
H	-3.755953696647	-3.496434729286	2.235095691779
H	-0.462927934354	-2.265680267020	3.524630047320
H	0.694394962714	-1.229845774978	4.409024041116
H	0.798185137549	-1.358308264539	2.646953032187
H	0.694308204476	1.705780222968	2.445136042155
H	0.550479297040	1.815843511732	4.211090097890
H	-0.662333458956	2.615432862055	3.166462546636
H	-2.960590875791	1.105123643936	4.587177663908
H	-1.741354058165	0.371526903843	5.661702907273
H	-2.904100664277	-0.657333374722	4.785530671571
H	-2.739504131059	5.663595446476	0.447503144291
H	-3.867121258673	3.443063941977	-3.050573171111
H	0.249794956340	3.768415672116	1.117611102691

H	-0.734945430632	5.181704009927	1.581512579689
H	0.547910663576	5.339560421619	0.365876041729
H	-5.285313238040	5.322095913398	-2.244586823734
H	-4.811176555663	6.251706810247	-0.800821389517
H	-5.511979301777	4.631607335850	-0.627270242917
H	-1.343357278015	1.125227783335	-2.948564281687
H	-0.644824372412	2.428694880800	-3.971857986735
H	-2.367485024026	1.994050571941	-4.115139298593
H	0.075143811579	5.169184015494	-3.482229824477
H	1.636422405925	6.005466808614	-3.265863539016
H	0.429772673584	5.990748771750	-1.953203782116
H	2.718127165380	4.658041995749	0.000380583371
H	3.765458981649	4.912326742843	-1.421400701416
H	3.633471895462	3.298081369985	-0.691384149033
H	3.051335700956	2.077316318631	-3.292971990750
H	3.244718251902	3.686379755607	-4.015568007170
H	1.803201194885	2.737544048888	-4.417465495924
H	3.462557826467	-2.449151454443	4.432193911388
H	3.332789442841	1.838213480288	4.591904762988
H	3.431442965496	-3.588559322152	2.404086926816
H	4.521321118020	-2.848171792650	1.207298463951
H	2.775682289007	-2.705547908871	0.997818649059
H	2.214267821809	-0.492601701835	6.491297251251
H	3.641805179536	0.544843104963	6.669703488340
H	3.823281811658	-1.226171545893	6.612952323548
H	4.471497766679	2.519657228987	1.472346167860
H	3.284189713066	3.128203822299	2.651883780697
H	2.748757593831	2.341841945887	1.141168819934
H	5.626828077578	-2.586254141752	-0.787385983433
H	6.748585012311	-1.577417450993	-1.735462182064
H	5.011021502029	-1.598144148081	-2.133188342460
H	6.454740963365	0.568029345774	2.019777151347
H	7.647425473272	-0.281567034440	0.998138691923
H	6.422281431924	-1.201476045467	1.915021925926
H	5.124455316546	1.530295064267	-1.945315455699
H	6.851749959890	1.341054146397	-1.549378661558
H	5.791505506998	2.297014989325	-0.483693666855
N	-2.540321459254	-0.016363638554	-0.998530088037
H	-2.985317829729	-0.924907048203	-1.117872015587
H	-3.271487524203	0.675817600227	-0.852332694888
H	0.937036638947	-0.832603487567	-3.591512614776
H	0.943003571985	0.824382095150	-3.581686613625
H	2.284779393125	-0.020590500892	-2.671535049935
H	2.013929906252	-0.000894298787	-4.579722700476
N	1.530680436188	-0.004970967043	-3.681827060824

148

NH3_Int_4, G=-4518.993097

C	-3.345463815927	-1.821548061923	2.520511921700
C	-3.354686453425	-0.460207257741	2.157182816853
C	-3.200451821970	0.527209284218	3.147546004831
C	-3.061766619217	0.138254248251	4.482190902567
C	-3.067449049383	-1.203320433740	4.866586244459
C	-3.210926163778	-2.166836732951	3.865998436381
N	-3.594992105577	-0.085767011727	0.805059783946
Si	-5.294684834937	0.149959993290	0.309734166424
C	-5.817082301639	-1.031730904107	-1.054296170877
C	-3.212162285521	1.978210648395	2.773760100618
C	-2.872079604375	-1.601237550900	6.300975458023
C	-3.484542429457	-2.883287548363	1.472853259929
C	-5.581914033811	1.887948444549	-0.352279569231
C	-6.354829027723	-0.132391288761	1.833160744959
Si	-2.304685386998	0.119651382308	-0.378154663009

Si	-0.393871184477	-1.020451380859	-0.960554878656
N	-0.290197692692	-2.461012470548	-1.944281141648
C	0.904138854890	-3.238516625686	-1.868838358652
C	0.971018223417	-4.315454196486	-0.964335752331
C	2.125868841710	-5.099700593872	-0.927474264824
C	3.224924199632	-4.833589994688	-1.746456674191
C	3.141099071083	-3.748919051697	-2.621875363251
C	2.000759665689	-2.944847570521	-2.702856871824
C	-0.177283209802	-4.598422502704	-0.044142989784
C	4.477953239859	-5.654606859544	-1.650580408087
C	1.947775216462	-1.785258552046	-3.650839583156
Si	-0.436371723047	1.440262359169	-0.580165534592
N	-0.265319620346	3.108581278696	-1.084699587773
C	1.076715576758	3.590228546366	-1.125944176787
C	1.883561149238	3.336481252235	-2.252614625932
C	3.208680595428	3.781218006677	-2.247720073362
C	3.752847684713	4.477849436984	-1.165616384065
C	2.924500999650	4.735830290621	-0.071857180270
C	1.600065712410	4.295142801931	-0.025668424446
C	1.331684973115	2.582164033670	-3.424137946741
C	5.195931243914	4.891170381340	-1.154623207539
C	0.751848228218	4.539129343846	1.185511155978
Si	1.329409978468	0.111325725054	0.074472705365
N	2.112405613890	-0.154424097309	1.632573266207
C	3.538426591262	-0.175661804021	1.633042623721
C	4.221867941858	-1.363117578049	1.304535843720
C	5.618120424148	-1.361456253634	1.272634023772
C	6.363729987139	-0.216016082754	1.561714346929
C	5.666147263384	0.948005832673	1.888807649147
C	4.269872049451	0.989103211411	1.931133228018
C	3.452763216347	-2.605501134997	0.978731615522
C	7.865071769103	-0.246370906016	1.550285428667
C	3.557468991206	2.256048300500	2.288891914443
Si	-1.607824651219	4.120604882568	-1.659843586520
C	-2.501248123881	3.272585025899	-3.077829325119
C	-0.864889130167	5.744071219228	-2.232554282951
C	-2.827392979001	4.399601541336	-0.261796249165
Si	1.253142230397	-0.420342246097	3.161665541676
C	2.540537947927	-0.737011989045	4.490985445768
C	0.234922685615	1.099221859237	3.578930914518
C	0.131721758419	-1.912037844077	3.004593864926
Si	-1.539531840432	-2.885635113198	-3.142588531515
C	-1.908252775334	-1.406293759276	-4.240319681282
C	-0.877342746830	-4.328232912525	-4.141536203086
C	-3.135576608382	-3.382647682573	-2.288854608268
H	3.835722461573	3.571309936139	-3.119623226840
H	3.327248539685	5.279648724690	0.787909365706
H	0.507306391665	3.133050593463	-3.903833512558
H	0.928989467576	1.605357186173	-3.110937775360
H	2.109405667388	2.404464813833	-4.179137432584
H	5.343543230176	5.830303183924	-0.601314674303
H	5.586342675228	5.028414583249	-2.173371639198
H	5.818066124838	4.122210725685	-0.665478232479
H	1.340117708816	4.990807992858	1.995957838952
H	0.310938062462	3.600502988367	1.557551922269
H	-0.090951220291	5.212194265195	0.962197823797
H	-0.332269063830	6.253974538997	-1.415420914909
H	-1.657665054411	6.416191934746	-2.597617464088
H	-0.143880742133	5.587400943277	-3.049459150728
H	-3.177233152945	3.436494449264	0.136746853396
H	-3.711379196887	4.955752750719	-0.612171565520
H	-2.370802106822	4.962001242088	0.566343845459
H	-1.829582146005	3.119406270639	-3.936418972725

H	-3.352530253776	3.888532394831	-3.411706863970
H	-2.890387815088	2.288970492918	-2.772882697805
H	6.138127599720	-2.288328555534	1.010533953593
H	6.225291545045	1.861540902426	2.115005901119
H	2.800558169513	-2.474569579456	0.099982290065
H	2.788387116372	-2.897310062499	1.806052445038
H	4.125592987724	-3.445951181786	0.764684854304
H	8.287789046719	0.753115119168	1.371670078793
H	8.249546351591	-0.923554752070	0.773046956626
H	8.262856365695	-0.601833285836	2.515949530611
H	3.118063650542	2.197278274576	3.297857347711
H	2.730468219615	2.457099790688	1.594437527636
H	4.241643820125	3.115172109424	2.270270374700
H	0.875860882182	1.979632404328	3.734095819901
H	-0.369826336003	0.935332570311	4.483426995540
H	-0.458382024779	1.320552221810	2.753858779653
H	-0.610680387729	-1.755983283710	2.208080578446
H	-0.426606978018	-2.085313730424	3.936805190538
H	0.709253122425	-2.817986202684	2.766018604497
H	3.151711314347	-1.620003853750	4.248722832877
H	2.040175047377	-0.920255785321	5.455493424860
H	3.225307208761	0.115137024463	4.615869539348
H	2.170426560886	-5.937971310804	-0.225431365551
H	3.990828612827	-3.519250378710	-3.272163194705
H	-0.408801131426	-3.721569576176	0.581309112688
H	0.047921856722	-5.442540089472	0.621947193552
H	-1.097737795487	-4.834048025302	-0.599575588665
H	5.011410069722	-5.693192973529	-2.611578553240
H	4.263040953971	-6.686258090661	-1.335913992682
H	5.174648969302	-5.226032330514	-0.909312807438
H	1.861518523543	-0.836257262405	-3.094842346029
H	1.085136080778	-1.854480859910	-4.330128529507
H	2.860153101662	-1.738431757864	-4.261608230702
H	0.044304956676	-4.067484289767	-4.682788949575
H	-1.631067478592	-4.649121106780	-4.878227074314
H	-0.646835607805	-5.185570171704	-3.490528977596
H	-3.017798673038	-4.298078940885	-1.689933401941
H	-3.927549494441	-3.560747867791	-3.034155699326
H	-3.484776381103	-2.580122884510	-1.623830964408
H	-2.478572320057	-0.641830701439	-3.689920675399
H	-2.521474670495	-1.724699589378	-5.099886237523
H	-0.987915255978	-0.944064290434	-4.627440130466
H	-2.949122444691	0.913809988339	5.246254132151
H	-3.207961402363	-3.226794165672	4.138418253102
H	-2.898996998210	2.608462713950	3.617305121274
H	-4.216835716406	2.306168351844	2.460576489672
H	-2.541647712192	2.175391054768	1.924045064808
H	-1.807785861902	-1.801548440178	6.514332052998
H	-3.428941992044	-2.517689730322	6.546121423896
H	-3.198275086088	-0.807982311831	6.989187102969
H	-4.484827620935	-2.872314701775	1.011654449662
H	-3.318203408523	-3.882855675883	1.897517705911
H	-2.764363010971	-2.730509703782	0.654960340443
H	-5.385195764115	2.663431988859	0.402313622513
H	-6.630874975957	1.988708798668	-0.676518521652
H	-4.940588392628	2.082169175957	-1.225296501307
H	-6.232119352842	-1.153107394554	2.226204790593
H	-7.416231370913	0.011506975283	1.574812092391
H	-6.100239152748	0.564598319759	2.645715340519
H	-5.267582969010	-0.805815605768	-1.981426191626
H	-6.892482778528	-0.893626523381	-1.256212426858
H	-5.656365992063	-2.086767644581	-0.789249378099
N	2.593759976844	0.359798605649	-1.109411872890

H	2.963677220625	1.307293102556	-1.156028222247
H	3.375482114132	-0.289547644781	-1.062783349676
H	-5.472423798792	1.360076097762	-3.789897645200
H	-4.049211473312	1.177727181597	-4.566246946589
H	-3.090597842634	0.319778297008	-1.632720996937
H	-5.101278138664	-0.067205129287	-4.489290312160
N	-4.709408888894	0.704377454805	-3.951517607826

148
NH3_Int_5, G=-4518.996278

C	-3.497904555176	-0.961508772106	2.439406283187
C	-3.609448896782	0.286388305898	1.786260709429
C	-3.678592744234	1.464248646117	2.561204853431
C	-3.640886672968	1.374829892444	3.957034496643
C	-3.535389245647	0.151092734973	4.616288898725
C	-3.462208309316	-1.004436690450	3.833538932361
N	-3.723803403790	0.346693497653	0.375160071415
Si	-5.354608483630	0.426534936646	-0.332827410725
C	-5.674311590497	-1.001494724996	-1.513867704793
C	-3.861893909080	2.802373352470	1.906532471515
C	-3.456081368260	0.076549474338	6.112835497034
C	-3.486953533220	-2.225901195145	1.637714245943
C	-5.642665090731	2.019775878750	-1.293298277584
C	-6.586173143129	0.314272168358	1.082731652662
Si	-2.287677455989	0.260793406261	-0.692767827504
Si	-0.534199084679	-1.125115946587	-1.123279728286
N	-0.540973034299	-2.810238427164	-1.682675379433
C	0.663713371118	-3.545670663014	-1.500024375123
C	0.738885831293	-4.559744327391	-0.526261936295
C	1.937355813302	-5.263277969230	-0.363896205594
C	3.064754936780	-4.995575864592	-1.138871132484
C	2.963664286358	-3.997749412551	-2.113100955297
C	1.786605826246	-3.275120094082	-2.315740940480
C	-0.461836454619	-4.917095508797	0.295954977198
C	4.353656951789	-5.735922775662	-0.928884176056
C	1.699983179403	-2.253548077071	-3.410517245897
Si	-0.350450735146	1.537688287786	-0.517872581727
N	0.053169525289	2.976984563843	-1.478380434925
C	1.428708007856	3.357934192492	-1.464395225456
C	2.348356727947	2.711522954468	-2.324230546784
C	3.707877105063	2.993931780647	-2.198753817665
C	4.196013959729	3.926487135155	-1.277061840312
C	3.265415401999	4.627260460707	-0.514941922833
C	1.892010693871	4.366357401429	-0.595612578568
C	1.867273025958	1.765876075161	-3.381088475438
C	5.671188066853	4.142028652214	-1.106287716887
C	0.954327594280	5.188596204327	0.242878360195
Si	1.156809483145	-0.149552988246	0.055494743711
N	1.826396068999	-0.341589949502	1.711413449661
C	3.237029543510	-0.162221360907	1.810231598737
C	4.105941823556	-1.261588689509	1.650356649489
C	5.485272470623	-1.052849784555	1.699132354017
C	6.039535990297	0.214442271828	1.900058985244
C	5.162843660192	1.289222097169	2.054941659470
C	3.775852324446	1.121143503448	2.015177003528
C	3.550925777240	-2.630646322871	1.404078218303
C	7.527242769042	0.405987506952	1.971668344528
C	2.866670243854	2.304967494990	2.150801493130
Si	-1.084853556361	3.709030018951	-2.620030457566
C	-1.615078714535	2.527713418120	-3.979916382503
C	-0.240607606253	5.211224820592	-3.362569417054
C	-2.611161065314	4.229266169374	-1.647780734438
Si	0.972186774081	-0.862202048547	3.166756180620

C	2.238059519394	-1.105477733486	4.534822318391
C	-0.250734758351	0.434546593377	3.774258601873
C	0.002603342720	-2.430842985850	2.844096375950
Si	-1.813688946861	-3.448059866159	-2.739245722634
C	-2.288004062998	-2.140067289815	-4.004229388193
C	-1.134953571266	-4.975641489868	-3.592794799271
C	-3.348126397590	-3.894043563340	-1.751388232833
H	4.412752753391	2.462749550497	-2.845769832372
H	3.613976959233	5.399690126334	0.178238425284
H	1.446907578178	2.329444977561	-4.231456069144
H	1.083802781255	1.096659466021	-2.999652479747
H	2.691425540600	1.147259972899	-3.758282900959
H	5.887471097554	5.107463525141	-0.626477158039
H	6.200234893696	4.111810657166	-2.070556690128
H	6.106097757444	3.350726316455	-0.471888575781
H	1.181005110655	6.260362440986	0.137762911118
H	1.049922037321	4.960777645388	1.319541777373
H	-0.089703115948	5.032966803054	-0.056224884548
H	0.029330353301	5.950967978023	-2.593768264647
H	-0.908129284650	5.699756314426	-4.090077137847
H	0.683747643009	4.924106482928	-3.887326656843
H	-3.074703879657	3.355717636406	-1.164830829910
H	-3.372873260861	4.674570448516	-2.307188811341
H	-2.366662212021	4.971381179032	-0.870631526412
H	-0.813377962961	2.379072562980	-4.717921851347
H	-2.499368662904	2.918934839537	-4.508549507364
H	-1.872299123167	1.543267659339	-3.560876600104
H	6.150666728332	-1.911611257560	1.564545721699
H	5.567909922644	2.294415186736	2.209018884734
H	2.826328956606	-2.641073338810	0.575588311596
H	3.008820351607	-3.014889753694	2.282402865180
H	4.349527178547	-3.346266862744	1.167766010410
H	7.811299449474	1.445704839766	1.752755700850
H	8.052920461155	-0.247879049473	1.259665710743
H	7.912385513763	0.165527877928	2.977040465554
H	2.115183217898	2.141508742385	2.938579980436
H	2.337138580131	2.491843749700	1.201238821813
H	3.432236273340	3.216263011645	2.387943314350
H	0.198971112852	1.435259769154	3.865978947650
H	-0.620111447931	0.150054285685	4.771636528037
H	-1.138505877662	0.475958039814	3.124745336800
H	-0.701367732325	-2.256012574748	2.017738711469
H	-0.577960751536	-2.729956034562	3.731193289403
H	0.661939110441	-3.261258905703	2.553349560015
H	2.991527181796	-1.866357990378	4.284726074701
H	1.724730137994	-1.420807895749	5.457598168776
H	2.775861624962	-0.168098014124	4.745887942754
H	1.986716376469	-6.046523380262	0.399003349938
H	3.826811080534	-3.783112227750	-2.751279237521
H	-1.055524324843	-4.027785357728	0.541544744214
H	-0.172287529000	-5.422718046478	1.227670788179
H	-1.125825315081	-5.602663515918	-0.257246960026
H	4.784835623719	-6.074023092387	-1.883763786652
H	4.212769553075	-6.618024820674	-0.288199879061
H	5.108661147205	-5.092675188578	-0.445976232258
H	1.496268814072	-1.247407546591	-3.010290650203
H	0.886088511290	-2.493393754229	-4.112292342027
H	2.640371030963	-2.206478484247	-3.977286935152
H	-0.240813859235	-4.740187803176	-4.189921780794
H	-1.895702004345	-5.402575302056	-4.265599662874
H	-0.850579808506	-5.749247229560	-2.863164582418
H	-3.162874853386	-4.736378169351	-1.068956331623
H	-4.180526356234	-4.166050661175	-2.419945260782

```
H        -3.671164230395     -3.034551157596     -1.147824612278
H        -2.661166841893     -1.227661829407     -3.513286628272
H        -3.081767973267     -2.516228535095     -4.669784858589
H        -1.424884319511     -1.851417526136     -4.623355733618
H        -3.702543684832      2.295617921569      4.545869800675
H        -3.383703900266     -1.979034477137      4.325201822680
H        -3.445923665866      3.612967748605      2.524116943752
H        -4.930918834036      3.030845715870      1.760160144418
H        -3.411089675299      2.826442864094      0.905865777225
H        -2.406249034539      0.051718762649      6.453005026091
H        -3.942011387883     -0.831877334364      6.498228050170
H        -3.931490454950      0.946944972792      6.587641417842
H        -4.458800020815     -2.386357533643      1.143263401383
H        -3.278436296514     -3.099159626992      2.270293177226
H        -2.733444079196     -2.184597971225      0.836923505944
H        -5.505556609229      2.915658778779     -0.670045166407
H        -6.674465319209      2.032446896784     -1.681126143450
H        -4.961613536603      2.095652230237     -2.154375156575
H        -6.443360519426     -0.612225051232      1.660114215903
H        -7.612184199608      0.311890154555      0.681129159980
H        -6.496802348998      1.156056145354      1.785407844469
H        -4.920540816819     -1.052575796642     -2.313640234505
H        -6.660149855690     -0.872226261890     -1.989736644899
H        -5.678421753626     -1.968542252756     -0.989466503057
N         2.564825433003     -0.100624820993     -0.988586954447
H         3.258174284882      0.613954245101     -0.781820925118
H         3.043861944629     -0.992006453894     -1.092591333820
H        -2.993228851366      0.551572081263     -1.985820882058
H        -1.206266750076      3.374543054249      1.101251188910
H        -1.036145729175      1.938003923307      1.911896024897
H         0.291985472522      2.861835715013      1.582541929546
N        -0.609747067424      2.554238025513      1.213937819885

152
NH3_Int_6, G=-4575.420987
C        -3.565918845784     -2.075967309615      1.695025492111
C        -3.767724172292     -0.679685010003      1.593877138905
C        -4.019999563410      0.059010543723      2.767549416570
C        -4.055863705500     -0.597054470366      4.005045080556
C        -3.829171166536     -1.967603019059      4.123202516588
C        -3.585644124356     -2.687289433292      2.947963766972
N        -3.762014638627     -0.055450590132      0.323146276097
Si       -5.328516660869      0.319792299577     -0.433108196094
C        -5.506736763559     -0.454248389036     -2.137217600316
C        -4.258562018140      1.537393333938      2.710846872134
C        -3.838024926537     -2.655421540284      5.457696379645
C        -3.374216293494     -2.887033521108      0.453461771554
C        -5.597111297174      2.175349515420     -0.621004119830
C        -6.672927868916     -0.402356871097      0.665730809762
Si       -2.239054215251      0.362206365932     -0.543206360182
Si       -0.325356278156     -0.661708354964     -1.242481790069
N        -0.185008218691     -2.050795143004     -2.349337556233
C         0.927560287156     -2.934246601441     -2.242584511229
C         0.757158569027     -4.243050223962     -1.741007589875
C         1.866220543452     -5.084855576805     -1.625964970248
C         3.145792995893     -4.682313605498     -2.010177793343
C         3.284911994712     -3.404480954772     -2.552298656454
C         2.206749332455     -2.524883561509     -2.680730563473
C        -0.600609878159     -4.764258602785     -1.376920475270
C         4.334269282971     -5.577564069401     -1.813172293899
C         2.427120346278     -1.167293750868     -3.274678016297
Si       -0.518589285436      1.729047845514      0.205950571367
N        -0.233031599704      3.354957754608     -0.500772934019
```

C	1.077275030637	3.611528662479	-1.003693995001
C	1.409092204652	3.368044488823	-2.353113625934
C	2.716511879616	3.620515203694	-2.789842561441
C	3.710180461282	4.100074021904	-1.939197808465
C	3.362725054697	4.330092499908	-0.604917904816
C	2.075381795166	4.092805105657	-0.127146708070
C	0.411450777177	2.810102529488	-3.323602419150
C	5.111168482687	4.339905085574	-2.421500186149
C	1.779216423500	4.296914567558	1.327657182173
Si	1.220748700022	0.111348278296	0.226323161130
N	2.035379564213	-0.748484422600	1.585604204637
C	3.323898392970	-0.262804348115	1.982756762720
C	4.505715550855	-0.674084802885	1.322473576001
C	5.740285025705	-0.189316117847	1.770763043206
C	5.858400373087	0.708787269358	2.828635909689
C	4.678253480782	1.143722888334	3.435679158962
C	3.426147482736	0.678760577038	3.033837543357
C	4.496781514962	-1.552228381576	0.105165170799
C	7.199020899470	1.187543407619	3.304865137621
C	2.198495944726	1.197816377573	3.717918157287
Si	-1.495236429947	4.540885952853	-0.862320700012
C	-2.560407639612	4.144590611064	-2.362838482035
C	-0.657096400561	6.200877210152	-1.117275251888
C	-2.688530041828	4.665360082374	0.596942040206
Si	1.640646344159	-2.354422905290	2.243241754615
C	2.824678607153	-3.715516996322	1.719241485820
C	1.755945607293	-2.297674844156	4.129056529065
C	-0.074962429518	-2.883512125648	1.720496905690
Si	-1.237009396727	-2.161726471298	-3.775008447589
C	-1.471446983500	-0.450509531107	-4.520086384133
C	-0.407369391468	-3.301945365424	-5.013061588626
C	-2.935202044584	-2.826623978721	-3.319416052099
H	2.964837970986	3.411607075707	-3.834806958359
H	4.123922513973	4.695357892844	0.091610393385
H	-0.149577440902	3.610436310326	-3.831764320276
H	-0.307053995741	2.151435986310	-2.817248146828
H	0.919664366094	2.225006351512	-4.103081554104
H	5.429424460097	5.376923975050	-2.229713176115
H	5.202864600686	4.152556647483	-3.500468899121
H	5.827759491145	3.681526307252	-1.904364862240
H	2.557837878771	4.901792384032	1.812138673851
H	1.756149823840	3.325523551036	1.850917764268
H	0.807112090890	4.791106412422	1.476144616453
H	-0.074174125238	6.504123257824	-0.233994711381
H	-1.408813751053	6.980399280272	-1.319732506657
H	0.034508342677	6.163106727572	-1.972903663114
H	-3.260479380556	3.734792252932	0.740634749793
H	-3.429757633655	5.450608610219	0.378005251372
H	-2.189620609708	4.942525414456	1.539139520573
H	-2.063292658735	4.413690605463	-3.305289085237
H	-3.505064011801	4.709443269396	-2.307896336158
H	-2.803451435287	3.072382459577	-2.402203667611
H	6.644493454133	-0.520106229815	1.249576837454
H	4.731175049584	1.875092062031	4.248471258692
H	3.488276717274	-1.856238478918	-0.184426809803
H	5.103633535515	-2.457642409753	0.259071142730
H	4.923268286522	-1.007499972753	-0.750408844003
H	7.159611712082	2.236245652619	3.635689729417
H	7.960320224160	1.106289854060	2.515581668192
H	7.551521427549	0.590086075232	4.162594945828
H	1.391604215638	1.372922192195	2.995037225466
H	2.411605552506	2.134486835279	4.252187580700
H	1.814163787000	0.474067740453	4.453467310992

H	1.701782829086	-3.325296613210	4.523003317667
H	0.940881976792	-1.724377518886	4.593703736001
H	2.716334449710	-1.866818331354	4.453433322237
H	-0.082705866109	-3.098300883057	0.643903429382
H	-0.863129624902	-2.145318100343	1.911689291365
H	-0.332244449155	-3.811590913589	2.258001251533
H	2.804898587882	-3.885853468266	0.633515538012
H	2.500390716968	-4.646865430226	2.212948091397
H	3.858851837567	-3.512665354007	2.033531831246
H	1.720749219810	-6.090508875822	-1.218903267937
H	4.271692524270	-3.072187197316	-2.889362621466
H	-1.262862571279	-3.961141785511	-1.035322846624
H	-0.535121740337	-5.531994217003	-0.592651647134
H	-1.087426454782	-5.230660961724	-2.250422349276
H	5.131515423522	-5.356812043122	-2.537957984792
H	4.064214133617	-6.639320511900	-1.912894466678
H	4.762165644548	-5.443781888920	-0.804589990659
H	2.580964353651	-0.419237279353	-2.477160953406
H	1.564048166985	-0.840403026004	-3.871181377036
H	3.322281791990	-1.162944548810	-3.913264062739
H	0.582305695299	-2.918587453628	-5.304816032124
H	-1.026091071920	-3.389981716900	-5.920361794798
H	-0.261461563410	-4.310569393976	-4.597452522801
H	-2.898671779577	-3.886556025533	-3.029301142392
H	-3.635255028833	-2.723797125554	-4.163948368769
H	-3.346836123592	-2.257479668145	-2.474058344226
H	-1.937473562124	0.238102096388	-3.798008417469
H	-2.123225465406	-0.507406311688	-5.407088354104
H	-0.509811113762	-0.011234928918	-4.826448256999
H	-4.252976071754	-0.008318074506	4.906758236590
H	-3.414822069247	-3.766544586038	3.006597810330
H	-3.828874324654	2.046995715000	3.586477064202
H	-5.335504094904	1.773937773740	2.696401886835
H	-3.836069183820	1.957947297932	1.789837136667
H	-2.849568361084	-3.081724379790	5.694850401886
H	-4.557793068214	-3.488938420031	5.473297643028
H	-4.108252988320	-1.962693299476	6.267247964128
H	-4.282398278426	-2.867490751142	-0.170365903704
H	-3.139917408455	-3.933370641749	0.690269671793
H	-2.562423185535	-2.476327720793	-0.164898264512
H	-5.648721329530	2.688342189596	0.350783348890
H	-6.545877170884	2.363189002302	-1.149581678507
H	-4.791184213576	2.636951393745	-1.210312686110
H	-6.532270185756	-1.486501126295	0.797937948351
H	-7.656868515477	-0.241270747018	0.196620862661
H	-6.694247634011	0.051557911700	1.667270635515
H	-4.714885502902	-0.132104014570	-2.828990244612
H	-6.475684578671	-0.156202458947	-2.570373910497
H	-5.486293272073	-1.553047059978	-2.084324387992
N	2.612153953264	0.808879936662	-0.578332167732
H	2.403992529675	1.538919771727	-1.251853064365
H	3.398253574230	1.094694656374	-0.002669197340
H	-2.886443545149	1.006730209122	-1.737776833094
H	-1.887736302256	2.819910014372	2.023473849920
H	-1.004335761863	1.526262262488	2.684586643376
H	-0.286861497549	3.025018758512	2.319278517708
N	-0.982964350348	2.349460473938	1.999951274371
H	-1.708439131533	-0.479299493687	3.500176099501
H	-0.115421165517	-0.366929359299	3.091582325809
H	-0.619772549187	0.109260050896	4.578837315164
N	-0.872513526487	0.099208917294	3.592636764643

152

NH3_TS_3, G=-4575.415884

C	4.593037343890	0.244068280975	1.338013199478
C	3.996874869986	-0.991126729838	0.980688649660
C	3.909418716471	-2.002340852932	1.963324317408
C	4.431864555603	-1.783828182332	3.244271577146
C	5.041462119937	-0.582145216563	3.601649646692
C	5.097370814113	0.420516169418	2.628890160938
N	3.505057749219	-1.212078839417	-0.341321099126
Si	4.681963167177	-1.915637176271	-1.482173259841
C	3.976128157759	-2.089360142754	-3.212507327982
C	3.240933772024	-3.311550806777	1.682811920935
C	5.631596822432	-0.374699498573	4.965960004723
C	4.679179923219	1.373635363064	0.364924401797
C	5.279232262481	-3.613442273168	-0.917023738745
C	6.254975679629	-0.885343182829	-1.607527122618
Si	1.805231643030	-0.753936260870	-0.739727934542
Si	0.986197503543	1.348365258447	-0.116080290820
N	1.250428825938	2.707835296093	-1.303655744111
C	0.792226107232	3.999168373850	-0.914111385886
C	1.541642586609	4.777817602122	-0.005358912347
C	1.064111337376	6.025040924564	0.399555340001
C	-0.142374885304	6.545589440506	-0.070659582947
C	-0.858328888343	5.778307245755	-0.989416369367
C	-0.417589205702	4.523034582302	-1.421276470820
C	2.841010347827	4.264330828520	0.525998581851
C	-0.663502748935	7.865724848424	0.419057716712
C	-1.234266131031	3.764134226055	-2.421372774587
Si	-0.111146523207	-1.650282902172	0.369909845729
N	-0.810084443259	-3.177075900854	-0.220054807877
C	-2.175496394448	-3.126768397795	-0.650761164560
C	-2.480533302559	-2.677640148942	-1.952523745455
C	-3.817546634074	-2.577324786914	-2.341274915334
C	-4.865817164243	-2.936253132134	-1.490556725476
C	-4.535731288553	-3.464622014081	-0.243102573782
C	-3.211373474276	-3.581108773761	0.186827119802
C	-1.386725470663	-2.377078669520	-2.933789423827
C	-6.293259998908	-2.733945083290	-1.901735849749
C	-2.908545412508	-4.257853434333	1.490900024875
Si	-1.180125501312	0.460514368025	-0.087350348884
N	-2.361727960758	1.103235416322	1.089983671683
C	-3.737568074571	0.778098413668	0.862880227744
C	-4.547226461068	1.583130101691	0.033147652223
C	-5.894665963046	1.247166911107	-0.136770732079
C	-6.480746820459	0.152958900851	0.498663398489
C	-5.668466108144	-0.621021146509	1.328706690886
C	-4.319846633045	-0.322649276224	1.524151037348
C	-4.010385461117	2.797588105635	-0.659346473973
C	-7.943260899326	-0.150851068730	0.341059711997
C	-3.504521390378	-1.145558560375	2.470598426789
Si	0.027045046005	-4.717023079893	-0.511746126581
C	1.562522923301	-4.460893426326	-1.556049857790
C	-1.141427927599	-5.865518117467	-1.428719552097
C	0.505717477778	-5.566021318530	1.103963891619
Si	-2.064625467883	2.155872136668	2.487624072744
C	-3.712694488709	2.902293874454	3.000101606221
C	-1.418733274883	1.199200286392	4.000236156305
C	-0.858740598772	3.540425273971	2.139354202109
Si	1.875392558734	2.461780264432	-2.933661164395
C	0.811406155914	1.285875659010	-3.956565719160
C	2.050184338970	4.137533078868	-3.772671980786
C	3.609672475068	1.725864283316	-2.901377201551
H	-4.047114485720	-2.208248668098	-3.345939387987
H	-5.336758478966	-3.797133438380	0.423692939244

H	-0.958502500360	-3.311847671788	-3.333509535457
H	-0.553131988380	-1.826551388533	-2.479036640658
H	-1.764731202415	-1.801020579595	-3.789858458369
H	-6.978847797471	-3.353474785705	-1.305838790094
H	-6.448128927877	-2.973566165930	-2.964431671594
H	-6.585811528203	-1.681458406349	-1.753591626041
H	-3.782233764405	-4.256820726406	2.156420120631
H	-2.062957918508	-3.790994070833	2.012057433187
H	-2.630201545654	-5.311873213407	1.323532034507
H	-2.052180287935	-6.083270893408	-0.851863593421
H	-0.625499283778	-6.818988693662	-1.626913675847
H	-1.456446694883	-5.441551997943	-2.393533865817
H	1.165082384317	-4.957454810619	1.737250498392
H	1.047079477576	-6.500132911625	0.881864396910
H	-0.386199620697	-5.827203588825	1.692191670262
H	1.281399967294	-4.120059718486	-2.563454934371
H	2.124667405718	-5.403463477160	-1.654212591205
H	2.236153685737	-3.704602806098	-1.131881580627
H	-6.511356550091	1.882577686211	-0.780653539860
H	-6.097723698745	-1.482105960035	1.849958264458
H	-3.109838750405	3.183819660911	-0.165892593410
H	-4.768622086593	3.593803803203	-0.699087022128
H	-3.713241599905	2.548252617662	-1.687526681271
H	-8.539606702124	0.357046181817	1.117899738614
H	-8.144222584109	-1.228756830977	0.430321746439
H	-8.325100726615	0.187761940440	-0.633472186284
H	-2.555015287745	-1.438234140675	1.999368495472
H	-4.043979845820	-2.051524110239	2.772240791223
H	-3.257468764524	-0.580833132357	3.381601612031
H	-0.746009824941	1.836250624928	4.598356706348
H	-0.872123979313	0.293292508208	3.707594868852
H	-2.245205948747	0.889404977538	4.658481451105
H	-1.132347396021	4.116596277617	1.242893636764
H	0.163745710791	3.165008903408	1.985992846249
H	-0.851956103804	4.230558140917	2.999615646416
H	-4.131416299861	3.544493112940	2.211013612132
H	-3.559076177576	3.522020085494	3.898746317564
H	-4.461361344129	2.131158730209	3.236931614360
H	1.659541111147	6.608661365377	1.108961726093
H	-1.803060230310	6.163154941672	-1.386388937490
H	2.686996726744	3.338107388877	1.105750479198
H	3.329557393888	5.006998719211	1.172483606414
H	3.527365516347	4.001991279064	-0.293235819229
H	-1.342486473504	8.328767219192	-0.311870826833
H	0.154539265669	8.573463215139	0.620876647918
H	-1.228082835510	7.745359161943	1.359437285957
H	-1.375719539407	2.715300665008	-2.125043836388
H	-0.755491243850	3.761030410096	-3.412733294209
H	-2.223003367348	4.225844960818	-2.543587005818
H	1.097566766482	4.663469835194	-3.924798795798
H	2.532181563105	4.007276347074	-4.755103784097
H	2.693498015947	4.792238837486	-3.163682713428
H	4.328541926051	2.438301286556	-2.468771808235
H	3.935601668874	1.492713899896	-3.928252976252
H	3.658947225423	0.800595496348	-2.311981969049
H	0.766085167503	0.299434339125	-3.469823699048
H	1.254593670577	1.144016160987	-4.955706273258
H	-0.215029958593	1.657250273755	-4.086519970176
H	4.349077548746	-2.583639330347	3.987115552317
H	5.547474916931	1.385143909830	2.881886494180
H	2.758869059500	-3.701825776937	2.592602608588
H	3.956329134059	-4.080201044052	1.355595894985
H	2.499173467035	-3.211276991811	0.881108607481

H	5.470176750210	0.652694247666	5.325007307501
H	6.721223011139	-0.543792135424	4.951710378317
H	5.202576488776	-1.068367955511	5.703350175457
H	5.361816024610	1.144557217769	-0.461900882063
H	5.036116213114	2.286160115959	0.859320883699
H	3.697117416348	1.584569365261	-0.078485460461
H	5.764252220742	-3.561159747304	0.070063992438
H	6.034782475913	-3.969500391132	-1.636314364555
H	4.475492554743	-4.361864122679	-0.878712788654
H	6.089297663305	0.073654275991	-2.119710967135
H	6.991118087469	-1.456069702503	-2.197036761483
H	6.701610256563	-0.689398785418	-0.620708503705
H	3.147418453052	-2.807921806380	-3.262677422312
H	4.790125348628	-2.462886534272	-3.856135752485
H	3.630489548697	-1.132333240656	-3.627050197937
N	-2.074613128572	0.422021011654	-1.586646766367
H	-1.514004881771	0.282159387190	-2.419245804939
H	-2.937018859154	-0.114103632936	-1.640723229722
H	1.682508677494	-1.297975431470	-2.132999384247
H	0.763270341212	-2.751910090211	2.383208662915
H	0.905966509053	-0.948069771800	2.660945268501
H	-0.628953030749	-1.955991377054	2.732282743186
N	0.243698662615	-1.890457650285	2.202799964525
H	2.619390887633	-0.078894056692	3.234035375885
H	1.582085094205	0.760281415062	2.228722368522
H	1.265491688911	0.644122881176	3.867461583353
N	1.633620229436	0.134878169547	3.065666786225

152

NH3_Int_7, G=-4575.420270

C	4.480821011299	-0.662210473909	1.486046559376
C	3.673627494698	-1.758404916612	1.087853704868
C	3.358000813394	-2.744416973299	2.051975716261
C	3.838039554184	-2.623351695822	3.362330896443
C	4.636150400985	-1.552675659345	3.766027113229
C	4.938428054531	-0.582202756377	2.804868268195
N	3.182865620998	-1.855122083518	-0.246467231434
Si	4.191311762894	-2.763945892368	-1.401274738934
C	3.453603687307	-2.765327791309	-3.126264318651
C	2.507344706758	-3.929358042940	1.716326415833
C	5.178683881898	-1.460549318436	5.162591057409
C	4.841320210777	0.425020552076	0.527579603752
C	4.435662818428	-4.557970585787	-0.872469397959
C	5.939912632240	-2.069536352457	-1.513818367290
Si	1.609743243155	-1.042064969770	-0.626616884762
Si	1.259870759080	1.209784134799	0.031636406846
N	1.754545210871	2.434273027527	-1.230496020809
C	1.619111989557	3.817859401707	-0.918483051802
C	2.548238847165	4.452367724941	-0.064024030602
C	2.414485825091	5.813344526744	0.217210733051
C	1.396610207444	6.589200217714	-0.339825678882
C	0.496457131731	5.952034534277	-1.192379196948
C	0.574198447831	4.586525711451	-1.478102829159
C	3.686677284894	3.675667113797	0.516975782220
C	1.250127187709	8.043984889164	0.001098743012
C	-0.475550395236	3.960369493504	-2.344777838977
Si	-0.469676518158	-1.613855529967	0.415775983024
N	-1.449159165493	-2.861264134458	-0.399973808333
C	-2.704093955908	-2.472059927248	-0.964271100459
C	-2.782564577727	-2.066707492768	-2.311428423664
C	-4.016592835530	-1.662261522834	-2.831134337166
C	-5.181819846966	-1.668131149114	-2.062749088405
C	-5.092474657434	-2.137436146351	-0.751420018953

C	-3.880594395564	-2.544435653975	-0.193349541437
C	-1.569380175519	-2.088599247765	-3.192193962793
C	-6.482760791899	-1.159927322007	-2.609862009333
C	-3.849534053632	-3.123366652607	1.187725183658
Si	-1.044150883746	0.673239373498	0.000342964240
N	-2.134637924835	1.520038995052	1.135420375129
C	-3.512995830881	1.136728048360	1.121629905808
C	-4.420985669902	1.746521708512	0.224404598974
C	-5.763984333823	1.366538690042	0.256420148574
C	-6.256925695756	0.430743482936	1.169043753644
C	-5.358423225649	-0.112542325247	2.084807500964
C	-4.001692757480	0.222924609918	2.076372029365
C	-3.986914773739	2.864943303921	-0.676263974371
C	-7.702687693302	0.028117676125	1.153698383312
C	-3.095439760548	-0.311362435839	3.143697799510
Si	-1.006342172091	-4.564626654088	-0.598337057511
C	0.603162369185	-4.727888237825	-1.551505703694
C	-2.378707950113	-5.432437341735	-1.539911677857
C	-0.817977942472	-5.432229406229	1.067212792204
Si	-1.742348972627	2.846740695681	2.246700730233
C	-3.333602039442	3.346098573418	3.121288568284
C	-0.477283436926	2.348127643195	3.560573715098
C	-1.037859481269	4.359298509275	1.410116240357
Si	2.328980017670	2.027496197076	-2.849079479880
C	1.064658056700	1.079189862568	-3.879183254279
C	2.819592038020	3.613360206182	-3.735741297473
C	3.891665450762	0.978159420456	-2.775441099857
H	-4.063743862923	-1.327114656289	-3.871999796637
H	-5.994886285206	-2.186547669716	-0.136989234763
H	-1.391320113344	-3.101650072563	-3.591650316474
H	-0.662419903170	-1.806063168873	-2.642956324823
H	-1.688186045690	-1.417200996221	-4.054348193403
H	-7.330801075160	-1.776500657116	-2.275171785270
H	-6.484001527887	-1.143537805864	-3.709170894760
H	-6.671137660061	-0.130648737419	-2.260734956063
H	-4.760032173617	-2.867942840433	1.744599721772
H	-2.978050110893	-2.773483545253	1.753903578722
H	-3.776969005888	-4.223341131637	1.147083911040
H	-3.337136302303	-5.386675162525	-1.001498902483
H	-2.112475505706	-6.492819468450	-1.678095359949
H	-2.538108074794	-4.986656237366	-2.532741394019
H	0.038051184242	-5.067081384602	1.650511834005
H	-0.660971750735	-6.510872233225	0.903292601154
H	-1.724743462662	-5.311584680715	1.678599426952
H	0.452973708755	-4.447015037406	-2.604518506654
H	0.982419826486	-5.761789623652	-1.520772793608
H	1.380580217927	-4.065586162623	-1.145683163850
H	-6.457213471891	1.846772953157	-0.441743080037
H	-5.723932377988	-0.814921527434	2.840874899689
H	-3.777488041914	3.772911711250	-0.086158020765
H	-4.778262456347	3.114703960709	-1.397133635774
H	-3.069050368709	2.609635990343	-1.221274318626
H	-7.981540937984	-0.515125628919	2.068069184582
H	-7.922427114241	-0.630550612550	0.296122729769
H	-8.364888821329	0.902678911972	1.061744596277
H	-2.072360548627	-0.414548753887	2.762914973905
H	-3.453039052339	-1.274296132555	3.535004216473
H	-3.041301266172	0.386768105799	3.996393244652
H	0.514342778052	2.311126285222	3.085003433955
H	-0.720344267745	1.380023284465	4.025754131059
H	-0.447371373874	3.114252070442	4.352003464476
H	-1.668090377486	4.706499247348	0.579422633230
H	-0.032586117111	4.171029791739	1.008558750498

H	-0.955343116964	5.178276661056	2.143363484163
H	-4.096783986362	3.683523444746	2.403739310624
H	-3.111292849126	4.186023100894	3.799437274411
H	-3.779825801439	2.535389965400	3.715170679383
H	3.144360133803	6.284788359610	0.883226360802
H	-0.321190598074	6.530738915981	-1.633858440491
H	3.317040494939	2.833790418882	1.125814553585
H	4.325492389087	4.313790055768	1.143927719984
H	4.306564133018	3.232235281861	-0.277677183431
H	0.805952148778	8.611068236565	-0.830323406515
H	2.219895192407	8.501716142517	0.246706882576
H	0.592862652285	8.181719681714	0.876728717392
H	-0.769634939131	2.967736410325	-1.976987930088
H	-0.128430351713	3.828808115123	-3.382586960633
H	-1.373376737809	4.593303595089	-2.384704737317
H	1.974578554249	4.287709891339	-3.932717928147
H	3.288725376039	3.358426956725	-4.699989293017
H	3.554682676223	4.173407500149	-3.136643576019
H	4.725280836834	1.557579784061	-2.349641803452
H	4.181019850472	0.659691129338	-3.790166244299
H	3.760852525686	0.078159477746	-2.160666989584
H	0.822997198353	0.112642057153	-3.413149600564
H	1.480357454823	0.868777516865	-4.878177037559
H	0.133703665715	1.649002464164	-4.012702671985
H	3.572945870035	-3.396806963167	4.090154985594
H	5.552922594031	0.277267662658	3.089169040579
H	1.902823357033	-4.233703524263	2.584334852499
H	3.119389682947	-4.801324484483	1.443557163997
H	1.854310414448	-3.721064215927	0.860685156618
H	5.257017210628	-0.416462534867	5.499935466411
H	6.191209025112	-1.893983261239	5.217074957881
H	4.550844946102	-2.009138389414	5.879592495459
H	5.504538490277	0.058807635206	-0.265388762333
H	5.349867486336	1.248085964510	1.045722932832
H	3.945668006126	0.828456724129	0.037347618761
H	4.931424148502	-4.628302052517	0.108092910571
H	5.096319781377	-5.043584950775	-1.609279790815
H	3.496808867551	-5.127885728304	-0.838553137556
H	5.969530026278	-1.080210103263	-1.992949733506
H	6.545107789594	-2.757057013094	-2.127108726815
H	6.419532276169	-2.000543612589	-0.525172170091
H	2.486373101929	-3.283365342049	-3.171061570567
H	4.159298000351	-3.306258500475	-3.778655945834
H	3.323827368352	-1.753772831827	-3.534057252171
N	-1.776107437228	0.771108058789	-1.586770385199
H	-1.141021190452	0.615186740911	-2.362218178757
H	-2.655223310715	0.287573301699	-1.754313562602
H	1.407014192301	-1.447873324697	-2.054099592263
H	0.302287373218	-2.934406690228	2.261856382971
H	0.913506535470	-0.886990210019	2.797479281489
H	-1.034512353307	-2.099376726430	2.700967470955
N	-0.175642327229	-2.040881142914	2.153360094836
H	2.534795408845	-0.508420941447	3.284426890594
H	1.692340713267	0.497771934536	2.162982356174
H	1.261300999948	0.465725203911	3.815085780054
N	1.616700846170	-0.112246880112	3.053688952937

152
NH3_TS_4, G=-4575.417987

C	4.640559870599	-0.021410087511	1.014602982607
C	3.874842901156	-1.211521458512	1.062600852269
C	3.728516920767	-1.873492576498	2.298275848709
C	4.308753124809	-1.327691991043	3.451992158135

C	5.040865971577	-0.141220102824	3.424889472197
C	5.193755565089	0.494245744535	2.186484136491
N	3.319442494460	-1.736540048665	-0.140217506658
Si	4.307401862030	-2.968741301331	-0.966058448575
C	4.116846275656	-2.870286319271	-2.831593819667
C	2.971239161601	-3.159540475068	2.408939075560
C	5.676423662608	0.419213769914	4.664218447578
C	4.871150738347	0.665222869458	-0.289634453365
C	3.918784732061	-4.737354564603	-0.430325792500
C	6.114996755247	-2.695952303459	-0.518559687590
Si	1.711532621579	-1.142949306210	-0.691297630440
Si	1.163171232439	1.124083820495	-0.309721611330
N	1.602727747190	2.372358237417	-1.545147442426
C	1.358586367450	3.725932876669	-1.161627066103
C	2.259564222553	4.394808211906	-0.304428018639
C	1.988772867969	5.701782098737	0.104155956718
C	0.849687089387	6.390053913535	-0.314685250478
C	-0.017180168216	5.726649975732	-1.182542342309
C	0.210499778482	4.415158047425	-1.611990691903
C	3.507498574387	3.714551705643	0.160674685849
C	0.552645425920	7.776975659637	0.177025921988
C	-0.771105879306	3.773607234334	-2.543057599501
Si	-0.333034415018	-1.680526958344	0.434334642411
N	-1.218938086797	-3.082143111335	-0.227653822660
C	-2.609082357328	-2.895923212968	-0.500485239995
C	-3.026580826292	-2.458176692099	-1.775356815313
C	-4.385529856921	-2.238609871733	-2.008101246121
C	-5.352684608265	-2.473766926423	-1.028001120171
C	-4.924348346562	-2.983176538006	0.197374667998
C	-3.573892296591	-3.208807931480	0.476264335949
C	-2.039002877357	-2.307712485342	-2.894744175689
C	-6.798151113243	-2.175590904837	-1.292992920369
C	-3.168695678990	-3.842522658539	1.775145440709
Si	-1.121084673625	0.519984765052	-0.122832979009
N	-2.168611765456	1.380375367712	1.045188582215
C	-3.584662682094	1.249359596803	0.853799245787
C	-4.284346963188	2.137662958401	0.007203601958
C	-5.670846544347	2.012281700825	-0.117841813600
C	-6.402051015352	1.048315863645	0.576352556573
C	-5.696181967703	0.187580142603	1.416127361301
C	-4.311419258941	0.277771274995	1.571243621245
C	-3.588159366668	3.226438070041	-0.748492227102
C	-7.898167439173	0.978119770798	0.463506863506
C	-3.618061047090	-0.625046772593	2.541049920062
Si	-0.568750906087	-4.688529551288	-0.603422573801
C	0.658456985710	-4.652129072096	-2.025694523726
C	-1.989108808302	-5.801485761140	-1.128290725838
C	0.252249966581	-5.425382236197	0.922016146714
Si	-1.704484945854	2.436031182757	2.390978280797
C	-3.197824473795	3.473620680207	2.865888182893
C	-1.216333123306	1.471811908460	3.956006167597
C	-0.297892776772	3.593252982602	1.956986526862
Si	2.014930436326	1.986080107113	-3.221279431010
C	0.679653771469	0.959778168104	-4.073463083461
C	2.380990702260	3.592162364297	-4.130056604543
C	3.584254752291	0.956901151908	-3.358629583833
H	-4.700162523156	-1.880677803515	-2.993803541582
H	-5.667143483629	-3.213367510775	0.967293237511
H	-1.864080408102	-3.281738186955	-3.382466834693
H	-1.063938235609	-1.954678787431	-2.536182490279
H	-2.409392151457	-1.622498259690	-3.670237713210
H	-7.446406499709	-2.583244339031	-0.504231692840
H	-7.127924149893	-2.593933769386	-2.256559537135

H	-6.965626713526	-1.087473520770	-1.331897051518
H	-3.976213627691	-3.785384895753	2.517736649384
H	-2.265069809546	-3.381149840701	2.193538756372
H	-2.932745008995	-4.910018862658	1.630775637300
H	-2.747026021425	-5.929002587065	-0.342216710304
H	-1.588800435923	-6.796448701346	-1.382442737301
H	-2.500468045148	-5.404161564834	-2.018104500623
H	1.094659419607	-4.816883487780	1.278221668056
H	0.645294426658	-6.430781941634	0.701592391343
H	-0.472956850067	-5.514368642035	1.745297445341
H	0.151530863688	-4.385187830837	-2.964710580641
H	1.125940994394	-5.640956895585	-2.157777038962
H	1.454760642363	-3.915688587714	-1.863002593425
H	-6.198798328657	2.713001854891	-0.772780690057
H	-6.238371312287	-0.577429349894	1.979985332549
H	-2.633371425520	3.499806512928	-0.282690170497
H	-4.222974680450	4.122060573000	-0.820627887415
H	-3.347348800839	2.885998051001	-1.764955336814
H	-8.379693269480	1.727499162337	1.114213344970
H	-8.280263263097	-0.008780751799	0.762097309420
H	-8.237842857284	1.178974878157	-0.563928172244
H	-2.694350379015	-1.024382523379	2.102961422898
H	-4.262612949909	-1.464657591498	2.827982977728
H	-3.339216892942	-0.086858315177	3.459499155155
H	-0.518348024018	2.060848079250	4.575403641306
H	-0.754400309572	0.500323314517	3.731190814137
H	-2.101852598910	1.275408328368	4.579846994767
H	-0.534205921447	4.202074137885	1.071170336962
H	0.644895558563	3.073137627967	1.738449516017
H	-0.127469631134	4.280479568750	2.802493167322
H	-3.510409939339	4.137967175151	2.046778133638
H	-2.935954530134	4.100597388545	3.733958629795
H	-4.061648122343	2.849256581187	3.139939972579
H	2.699487455026	6.198637261805	0.772191442089
H	-0.915908000526	6.240853043405	-1.537289376080
H	3.276464824199	2.798722621920	0.729821084441
H	4.108050887305	4.379490478227	0.797212349477
H	4.122647778886	3.398744773793	-0.694663160733
H	-0.066537218761	8.336161607503	-0.539588505972
H	1.475328141798	8.350348310491	0.351331047402
H	0.001661967768	7.748966203083	1.132607545380
H	-1.060983661073	2.768769262562	-2.205231304318
H	-0.359756636389	3.670591014820	-3.558498114101
H	-1.679538061591	4.384212039366	-2.625571771752
H	1.532725186836	4.287322306862	-4.187623133802
H	2.711022134204	3.363869806848	-5.156286261520
H	3.203732102075	4.119193789846	-3.621084165915
H	4.471491097401	1.514353930013	-3.023835717986
H	3.732996661454	0.681367466247	-4.415706016409
H	3.518523175966	0.027697657266	-2.777423604728
H	0.553721484103	0.005648690089	-3.536362703044
H	0.989765458534	0.716440035833	-5.102482277527
H	-0.293101340964	1.468427505672	-4.119677570170
H	4.175805183795	-1.852155657324	4.403514899807
H	5.773967631387	1.420031451640	2.127314070510
H	2.370210307361	-3.189449026769	3.330384285508
H	3.652626453091	-4.023875952549	2.447099963427
H	2.321343073501	-3.297619115064	1.537623221519
H	5.553464804435	1.511262277384	4.723706234837
H	6.760205183352	0.216867558863	4.676076826284
H	5.248802816536	-0.024256415583	5.574686577211
H	5.275558396098	-0.028586356265	-1.038195519819
H	5.567731197476	1.506307850011	-0.177781530912

H	3.929979430673	1.052551357067	-0.702724134011
H	4.362652957741	-4.964222032923	0.550215220740
H	4.359705411260	-5.435052782757	-1.161274294292
H	2.843322407347	-4.950234639097	-0.376074562911
H	6.526060432599	-1.759802626398	-0.923073148396
H	6.706097930978	-3.529254131383	-0.931929018657
H	6.262796472966	-2.687987650091	0.572200651361
H	3.111365341448	-3.145190415299	-3.180151282024
H	4.831120629294	-3.578642963771	-3.282610547166
H	4.348448135315	-1.866091355008	-3.214675849942
N	-2.009158211166	0.584003027850	-1.626936734392
H	-1.469543403419	0.369940790105	-2.457817044641
H	-2.909020808875	0.110313104619	-1.661678365259
H	1.602459823882	-1.693433831310	-2.081689679504
H	0.443836511666	-2.584282903317	2.531785949702
H	1.202220955509	-0.215541264079	2.727998022820
H	-0.867514363601	-1.620177361869	2.764711690022
N	-0.028289825939	-1.745303492229	2.200177975608
H	2.727127657050	0.454304326942	2.995711986015
H	1.644569625334	1.053934712321	1.513008334740
H	1.351803749804	1.312018772332	3.421916351382
N	1.756506319223	0.662396073251	2.751001792980

148
1, G=-4519.059844

Si	0.737014663504	-0.961997736085	-0.951258136042
Si	-0.736824493557	-0.962042144049	0.951318609023
Si	-0.644061200895	1.440040548607	1.025096767994
H	0.238942126462	1.861912827788	2.159702989502
Si	0.643629130183	1.440035644688	-1.025404407434
H	-0.239465807010	1.861494070543	-2.160090126454
Si	0.719160361500	-1.808555320445	-3.956219406684
Si	-0.718739776151	-1.808240765705	3.956368634296
Si	-2.198847027806	3.871762511616	2.235405744028
Si	2.197970533456	3.871974647718	-2.235919023124
N	0.000395188810	-1.797746158089	-2.326470146959
N	0.000046182627	-1.797409553694	2.326627519299
N	-2.115926703657	2.418315492509	1.219022743978
N	2.115288398818	2.418597275098	-1.219408459263
N	2.385771745010	-1.501290869893	-0.821402192410
H	2.542505753574	-2.430637619060	-0.436706254997
H	3.049613650464	-0.855845473162	-0.401380338168
N	-2.385429497532	-1.501795188422	0.821587732187
H	-2.541949751540	-2.431251536950	0.437070774703
H	-3.049474336321	-0.856601474158	0.401502721390
C	-1.225157066854	-2.494435519319	-2.135238529899
C	-2.454833879213	-1.828569868182	-2.300557395034
C	-3.646216141263	-2.524549907259	-2.085761423487
H	-4.594542764468	-1.990725776625	-2.201819638795
C	-3.658075265254	-3.866017896192	-1.696777201500
C	-2.427548188511	-4.512290366778	-1.549429127235
H	-2.408682643343	-5.566294119735	-1.255201920933
C	-1.213485771354	-3.857005261013	-1.778634886140
C	-2.479718107176	-0.371967346570	-2.634083772035
H	-3.490152202353	-0.037764136212	-2.901262640982
H	-1.788750348578	-0.122974440741	-3.450551041967
H	-2.164709734198	0.234997649728	-1.767568659351
C	-4.950793256830	-4.597731558460	-1.478078737001
H	-5.322597834785	-5.036227862776	-2.419501163552
H	-5.735132837284	-3.925306177390	-1.100401312577
H	-4.831950576600	-5.421384707015	-0.759143134897
C	0.084950319005	-4.597260315480	-1.670583586758
H	-0.070551054976	-5.617416933585	-1.295030241656

H	0.796256610533	-4.087859262736	-1.004580652833
H	0.577536273001	-4.670845050303	-2.652746348283
C	0.935333159691	-0.047734957121	-4.579438183807
H	-0.034519591342	0.451974897715	-4.723409827449
H	1.465115265363	-0.047632958924	-5.545712125802
H	1.520156877035	0.566987602368	-3.881239772867
C	-0.472127798223	-2.720175917967	-5.085961272567
H	-0.628346258444	-3.759761474845	-4.760013712225
H	-0.066066115003	-2.740176938043	-6.110096669060
H	-1.457661305876	-2.231369630954	-5.115712620175
C	2.397370250365	-2.652990166269	-3.980204830280
H	3.042491873988	-2.220697662597	-3.201631108918
H	2.878031046866	-2.525831682634	-4.963659094468
H	2.309640454829	-3.731800170678	-3.780735504605
C	1.225839104489	-2.493701947421	2.135501060824
C	2.455320843159	-1.827287126236	2.300687807099
C	3.646922453586	-2.522788548953	2.086016259395
H	4.595036573488	-1.988552062457	2.201982163198
C	3.659300286243	-3.864382478688	1.697233868139
C	2.429089431028	-4.511140840686	1.550063367051
H	2.410665787969	-5.565199539903	1.256030351667
C	1.214702382927	-3.856277172298	1.779177496192
C	2.479565867945	-0.370620703275	2.633987640412
H	3.489881659585	-0.035899264280	2.900964626925
H	1.788592585387	-0.121826468333	3.450512060310
H	2.164139728619	0.236052971345	1.767422085895
C	4.952531503353	-4.595167980520	1.478458670343
H	4.830636084768	-5.428962731141	0.771895360424
H	5.333227807266	-5.019243901441	2.422918674004
H	5.731808598684	-3.925621479930	1.085359850644
C	-0.083400297452	-4.597145294800	1.671301998073
H	0.072527719930	-5.617209440963	1.295673163515
H	-0.795065950324	-4.088049992150	1.005450497165
H	-0.575777023903	-4.671036300096	2.653549323900
C	0.472846023187	-2.719295283246	5.086256016067
H	0.629380865339	-3.758894874191	4.760507817360
H	0.066813886927	-2.739221259278	6.110404645999
H	1.458229745568	-2.230179103446	5.115894380473
C	-2.396672935444	-2.653217717036	3.980475206029
H	-3.042045836309	-2.221033460206	3.202050946664
H	-2.877231138726	-2.526344584120	4.964016806770
H	-2.308619378894	-3.731970630431	3.780845378295
C	-0.935485277378	-0.047399295073	4.579331816518
H	0.034215489883	0.452559178839	4.723470336918
H	-1.465496398898	-0.047296089943	5.545479440811
H	-1.520286349211	0.567098508580	3.880915468868
C	-3.266023025476	2.051260336720	0.459822601387
C	-3.484342784923	2.581305409189	-0.827606529658
C	-4.606161201381	2.173601476023	-1.555538918657
H	-4.758022673694	2.586380943231	-2.557832506902
C	-5.525793005436	1.256142970158	-1.049345743618
C	-5.321242641537	0.781849606533	0.249180326559
H	-6.047532115865	0.090997453932	0.688245713406
C	-4.224461203588	1.176572009611	1.019019793579
C	-2.564767428161	3.617403677104	-1.397955181736
H	-1.553464620185	3.515998306679	-0.989707961875
H	-2.917488753001	4.632024673361	-1.145311896078
H	-2.511561571504	3.550957877604	-2.494020984559
C	-6.682305602485	0.776927932914	-1.877435865116
H	-6.401042668562	-0.107123034724	-2.475727052803
H	-7.022726604386	1.549041868182	-2.582730374986
H	-7.536629937092	0.485015799992	-1.249531772525
C	-4.091508310618	0.711773143911	2.436894600635

H	-4.176539803045	1.556740796772	3.138478439527
H	-3.118284796832	0.239220446694	2.615763763338
H	-4.871572319511	-0.019438435584	2.687974049086
C	-0.980527598777	5.182363198828	1.660773559022
H	0.056352569771	4.823210357856	1.735398410299
H	-1.066817883936	6.078899230096	2.295877804565
H	-1.161589894174	5.483374440416	0.618952414318
C	-1.800308562203	3.484308146480	4.030568604038
H	-2.516050200265	2.772810246736	4.468231839646
H	-1.833938196441	4.411873802323	4.624967598442
H	-0.792915429490	3.054415199523	4.136063857274
C	-3.945744017961	4.547048139083	2.100817649205
H	-4.192268520157	4.829768191892	1.066296616256
H	-4.043148372960	5.444492333143	2.732385965699
H	-4.697119014282	3.814177525017	2.431094458207
C	3.265396994064	2.051866438191	-0.460068858571
C	4.223978658040	1.177160616669	-1.019001267136
C	5.320757621852	0.782752146803	-0.249000832961
H	6.047168307051	0.091899365763	-0.687866161151
C	5.525153119510	1.257348928989	1.049441433749
C	4.605349759100	2.174771998868	1.555381380179
H	4.757069628791	2.587764723579	2.557608493400
C	3.483529914122	2.582176460238	0.827279056269
C	4.091116352103	0.711926950775	-2.436739470179
H	4.175661211548	1.556737272055	-3.138565354716
H	3.118104590707	0.238841617801	-2.615372748733
H	4.871503980144	-0.018983831928	-2.687691149910
C	6.681678220778	0.778466632743	1.877706642872
H	7.021737720155	1.550662386323	2.583084383138
H	7.536199447539	0.486846452983	1.249933553886
H	6.400599435375	-0.105699658712	2.475912600457
C	2.563734455786	3.618217514217	1.397380337074
H	2.510397348189	3.551898930682	2.493447807055
H	1.552495401329	3.516624427831	0.989018456640
H	2.916352206122	4.632855055528	1.144660280956
C	3.944762904560	4.547547185333	-2.101404032435
H	4.191245762325	4.830403984296	-1.066910294288
H	4.042019409555	5.444948712760	-2.733055855094
H	4.696255169223	3.814767794038	-2.431615634154
C	0.979468456579	5.182453506115	-1.661396601329
H	-0.057358529205	4.823138566914	-1.735958864258
H	1.065610711635	6.078931482711	-2.296603026494
H	1.160508650373	5.483608832824	-0.619613251686
C	1.799465550018	3.484312261642	-4.031044663158
H	2.515328923040	2.772919642829	-4.468679236065
H	1.832905433182	4.411843157502	-4.625508662089
H	0.792151325623	3.054221924432	-4.136484680722

143
H2O_Int_1, G=-4482.363876

Si	-1.596012476996	0.513386710775	-0.677976319866
Si	0.250284758063	0.414573786713	0.607049052813
Si	0.097815304001	-1.405589520741	-0.677503744058
Si	-2.236447819095	-1.752019320028	-0.695046115023
Si	-3.982939758028	1.739729888270	-2.267013910942
Si	1.064703523667	2.950415679039	2.243480903295
Si	0.986874890679	-4.053012136427	-2.034354329320
Si	-4.148504571044	-3.327538628189	1.085836401308
N	-2.410500965853	1.874596569713	-1.390941948249
N	1.437591134297	1.420992680233	1.371826985096
N	1.323092312748	-2.422473499160	-1.364575040903
N	-2.795532721982	-2.190812422425	0.978970857567
C	-1.735123476713	3.135762612104	-1.408093963909

C	-0.636515595441	3.332551269364	-2.272235161755
C	0.004699134844	4.573402522275	-2.275671114921
H	0.854816060869	4.718900690761	-2.949095306445
C	-0.411014989859	5.630227961845	-1.462004531267
C	-1.509648497558	5.414941201681	-0.629874566904
H	-1.858446977257	6.226136960595	0.016465496025
C	-2.182383339376	4.190390205173	-0.587613880260
C	-0.175726208513	2.242440406221	-3.189965031909
H	0.594863586734	2.610784845003	-3.880901535318
H	-1.001899002026	1.816080977042	-3.776883918959
H	0.250709933996	1.392747356034	-2.633015429362
C	0.326096879746	6.937487126467	-1.454906072287
H	-0.325681459633	7.767841944720	-1.147156098348
H	0.738156443702	7.174151984534	-2.446847941533
H	1.173277865020	6.905364801916	-0.748588838438
C	-3.375241513815	4.028793043696	0.308495658537
H	-3.350597777942	4.758088654133	1.129794422480
H	-3.427568315453	3.018597987482	0.736479217332
H	-4.314959693746	4.190085546282	-0.244704622847
C	-4.212706044449	3.364752063556	-3.177799303126
H	-4.275224604602	4.238344416085	-2.514032547714
H	-5.140418265481	3.315366352098	-3.770106472945
H	-3.375224177304	3.535469063621	-3.872068140055
C	-5.407129568067	1.395701449822	-1.090827198603
H	-5.322336557594	0.379623611684	-0.676491974355
H	-6.351605573205	1.439106620952	-1.657754110510
H	-5.477052234936	2.105850921292	-0.255801019719
C	-3.994673557527	0.351125700614	-3.522014413884
H	-3.132662761969	0.380215022420	-4.202386227473
H	-4.922299652926	0.430364800748	-4.113000959183
H	-3.992706582526	-0.629170867277	-3.019973076816
C	2.835738575149	1.106031010550	1.307613759881
C	3.466884486494	0.425779567111	2.367913118710
C	4.859967472832	0.316656297519	2.359074105685
H	5.348681733985	-0.194272273576	3.195154728561
C	5.641386972522	0.851698617247	1.335413152310
C	4.983013813571	1.442450953481	0.255638050444
H	5.567795021687	1.826242762773	-0.585188832693
C	3.592956048334	1.554196381736	0.204601139709
C	2.691853010982	-0.196604597419	3.492459996609
H	2.925141721195	-1.270281935187	3.563936116203
H	1.609674008201	-0.097610771158	3.349391735823
H	2.953172320334	0.255042901990	4.461735342178
C	7.137988357306	0.746403543102	1.380806247127
H	7.612154659007	1.396727371471	0.632277607181
H	7.472323708268	-0.285063915181	1.180748853304
H	7.529508716068	1.025824775514	2.370825810150
C	2.922753279822	2.087200629444	-1.025441377142
H	3.639933640903	2.609588381051	-1.673436212718
H	2.089851601589	2.764554384205	-0.800205333191
H	2.506364028448	1.247694213911	-1.607447883114
C	1.708571619130	4.417640350336	1.272389450095
H	2.790050067498	4.330841472953	1.086016349030
H	1.535334107055	5.342225957195	1.846757844070
H	1.186075055254	4.514059107066	0.309397323172
C	-0.787016739847	3.039409368929	2.441571084936
H	-1.275590130044	3.115317218420	1.462423821539
H	-1.055232687205	3.936375970352	3.022488397447
H	-1.178996925546	2.152871706900	2.961426193999
C	1.888814753851	2.953830614108	3.928815027301
H	1.463788206012	2.189980963978	4.595220047915
H	1.723139746037	3.939998594406	4.392644090943
H	2.975040853698	2.794760260071	3.855674391889

C	2.673554843992	-1.952980376891	-1.441409940149
C	3.552352722030	-2.198434340391	-0.369029458119
C	4.900344597395	-1.868000044140	-0.509725764897
H	5.581688782596	-2.081658638959	0.318324671561
C	5.398023400732	-1.278485404214	-1.673380273675
C	4.495116575876	-0.993114774539	-2.697572270108
H	4.858007496376	-0.516502006499	-3.613763003016
C	3.139510991378	-1.321135173440	-2.609061591426
C	3.049342061467	-2.841970306125	0.886872268783
H	3.843348182430	-2.896274942731	1.643948192418
H	2.678212401666	-3.862686781025	0.701157257445
H	2.206984501980	-2.276240663893	1.314398324460
C	6.851917859965	-0.936186488581	-1.817286035384
H	7.033335649950	0.131199735009	-1.609849470289
H	7.211242173920	-1.133543881707	-2.838310962300
H	7.473982028165	-1.514746254313	-1.119594851539
C	2.229487212292	-1.055821208861	-3.770718313044
H	2.608733863273	-0.223032272720	-4.380190371271
H	1.197523607473	-0.830646359583	-3.468356043578
H	2.176098581483	-1.938224017970	-4.432409361944
C	2.641972769594	-4.773640988110	-2.540801842773
H	3.132705391405	-4.165906481819	-3.315686450963
H	2.492336933055	-5.789216990317	-2.940909782645
H	3.333419616846	-4.838054929859	-1.687160190649
C	0.165575393274	-5.162922340655	-0.765596998141
H	0.777085640606	-5.293466396545	0.139339273344
H	0.003224377861	-6.156809841421	-1.213831823033
H	-0.816705543168	-4.768811470172	-0.467046192391
C	-0.157900584316	-3.981570755242	-3.522044415575
H	-1.146933924773	-3.602145168022	-3.222349955670
H	-0.292385926810	-4.998193008577	-3.925744722186
H	0.255689984441	-3.348118073731	-4.319995517840
C	-2.256859463673	-1.622223263866	2.158909673547
C	-2.823546764499	-0.459569583258	2.722591406124
C	-2.275907100668	0.080600953279	3.888578644447
H	-2.735223715379	0.978022694512	4.315018695574
C	-1.164804724521	-0.482390386730	4.519106770942
C	-0.614314455605	-1.630772762435	3.948037652393
H	0.254762238397	-2.099737170529	4.419264716275
C	-1.141815410652	-2.211175911582	2.791815580135
C	-3.999184690000	0.196385173886	2.070740948352
H	-4.914597288204	-0.408400208592	2.171218371015
H	-3.828552352327	0.317101407291	0.990829248261
H	-4.198924753336	1.184824776268	2.507492507971
C	-0.600667152512	0.112624918150	5.776958203831
H	-0.705205569732	1.208111423522	5.786627121931
H	0.466174805449	-0.128719213928	5.893367456744
H	-1.121571205241	-0.270597358022	6.670598020683
C	-0.527590417687	-3.446333539468	2.219838698639
H	0.338471565615	-3.777494453712	2.809258777295
H	-0.193163215417	-3.268297369716	1.185654071597
H	-1.250985006500	-4.274007048653	2.164444302272
C	-5.653469650587	-2.659184815509	0.170808701625
H	-6.036387213992	-1.736137779621	0.631808535420
H	-6.469338532138	-3.400014644767	0.160417427850
H	-5.392079510414	-2.430515485613	-0.875109384645
C	-4.543790981712	-3.591220856800	2.901889832175
H	-3.671804116645	-3.991181919940	3.442065044531
H	-5.374569617259	-4.306353002061	3.011354542592
H	-4.832243774006	-2.650120463044	3.394280977181
C	-3.692178983233	-4.968042112833	0.282462518050
H	-3.387030632976	-4.812110881500	-0.764654837656
H	-4.552619766684	-5.656798414921	0.284011517335

H	-2.859830174275	-5.460666556698	0.808077650589
H	-1.667142542033	-1.226601509906	-3.153355696858
O	-1.105465247072	-0.886903930024	-3.870909527475
H	-1.049020933695	-1.636422966542	-4.472675309785

146
H2O_Int_2, G=-4558.644982

Si	-1.553733646168	0.672734078776	-0.576288712892
Si	0.330605914636	0.453052414674	0.628516535335
Si	-0.043959772149	-1.389278839095	-0.590519401147
Si	-2.391731154303	-1.510773732874	-0.369988244046
Si	-3.906728392515	1.971560402955	-2.104489685669
Si	1.374777271705	2.984361009014	2.122412433942
Si	0.717443717250	-4.092460161938	-2.013707621915
Si	-4.282148985051	-2.853351810167	1.599857520601
N	-2.298251265206	2.065168096749	-1.301954938995
N	1.618119086360	1.399064273147	1.303469016577
N	1.093774574122	-2.484385845575	-1.294547506285
N	-2.838443010474	-1.857322518301	1.350379150391
C	-1.542775826271	3.272733645414	-1.414039239381
C	-0.477259758890	3.350915737172	-2.335819828935
C	0.250669903034	4.539585904538	-2.424728517113
H	1.076378352501	4.593759683652	-3.140757678265
C	-0.049280219531	5.657281776606	-1.641812979748
C	-1.121742957100	5.560498649504	-0.754645883567
H	-1.381024787192	6.422766043753	-0.132766618313
C	-1.877517787312	4.391752772479	-0.625954407047
C	-0.144090363787	2.189453108145	-3.221103520009
H	0.624151640002	2.464087458296	-3.956886351778
H	-1.025162466768	1.808050242158	-3.757103494259
H	0.234141548905	1.330491892741	-2.644442677820
C	0.781206647725	6.904679383066	-1.723859224492
H	0.203381824052	7.795209827054	-1.437223272874
H	1.174014310257	7.062599609810	-2.738975469288
H	1.648254705542	6.842907202544	-1.044090039387
C	-3.035904495469	4.353736453702	0.327780512167
H	-2.927717686764	5.120008415478	1.107608575732
H	-3.132380686197	3.372062429567	0.810972289484
H	-3.988695638147	4.546889654631	-0.191768253759
C	-4.137712023889	3.581469790243	-3.040528946608
H	-4.139432840347	4.472232941650	-2.397245321654
H	-5.096477956764	3.547189688285	-3.582409134024
H	-3.333642481670	3.708664384304	-3.782070753547
C	-5.256456397904	1.688920726188	-0.831207816245
H	-5.107623581837	0.717222586462	-0.335386382954
H	-6.238500087938	1.657464300243	-1.330499466218
H	-5.289729786779	2.469061041959	-0.057638424916
C	-3.979168997844	0.561265144147	-3.331459974928
H	-3.128817730949	0.560878888715	-4.028462054761
H	-4.918233875627	0.630393643513	-3.905348367868
H	-3.960505434289	-0.405189209247	-2.808267534294
C	2.992160496137	0.998863934415	1.197370849165
C	3.634386467396	0.336049005409	2.261339664192
C	5.017490291568	0.147365071614	2.194939338178
H	5.516684987891	-0.348982159844	3.033600199673
C	5.777061117162	0.582954537308	1.109888933285
C	5.101074038139	1.154749783255	0.030458905071
H	5.664240676039	1.457407313132	-0.856924223439
C	3.719284609003	1.347763781566	0.039826338391
C	2.882175947884	-0.187364122547	3.449272591286
H	3.055385319920	-1.268950110957	3.560018632810
H	1.801745601666	-0.030404260258	3.353745716947
H	3.218607522707	0.290366961223	4.382034500699

C	7.266549254274	0.397445059518	1.096984697328
H	7.734952671001	0.941050610521	0.264546134729
H	7.536303047846	-0.666162246071	0.991763864322
H	7.721722268066	0.754209987487	2.033743621586
C	3.015561467618	1.870883691543	-1.175138928693
H	3.735128414937	2.173816385169	-1.947921228548
H	2.354156698198	2.718847333752	-0.955773088973
H	2.382182329993	1.078857919696	-1.606652391890
C	-0.459034157273	3.207732775419	2.375482356937
H	-0.977840039546	3.269238584372	1.411226649341
H	-0.644668505513	4.147979304185	2.919038046547
H	-0.886103212877	2.376400046483	2.954427084350
C	2.250795896815	3.001077814986	3.781073619666
H	1.805726137241	2.285770257092	4.487158625774
H	2.153554617123	4.010884079831	4.212279632911
H	3.323883152343	2.779270002921	3.682458949283
C	2.075182895245	4.371674608178	1.075723168193
H	3.140575195921	4.208494217286	0.851999059477
H	1.985110110962	5.322940583769	1.625102442142
H	1.520581206692	4.473246753195	0.131244212077
C	2.468894427476	-2.093609936622	-1.404338084584
C	3.346782769268	-2.397745816988	-0.341549237578
C	4.715728024522	-2.205622200209	-0.518025433063
H	5.392544545484	-2.467205887714	0.300006593206
C	5.241106953510	-1.691361997923	-1.706175649401
C	4.344095598965	-1.307234313205	-2.700133240128
H	4.727501802592	-0.856968674174	-3.621700692377
C	2.961898414791	-1.486060511933	-2.574518250762
C	2.811767994462	-2.922638040595	0.955962865883
H	3.623201727500	-3.067484461479	1.682167045895
H	2.281797543997	-3.877537478506	0.829215747592
H	2.085162607581	-2.219616009925	1.394078391759
C	6.718955854695	-1.516543486732	-1.898949769705
H	7.013253108217	-0.459200878462	-1.797643190434
H	7.033226505046	-1.845217860587	-2.901144868578
H	7.293532359209	-2.089925732998	-1.157913931729
C	2.066556448389	-1.002379293624	-3.678347376935
H	2.286145179084	0.054216343111	-3.899403960008
H	0.998202224791	-1.082962895144	-3.443669483659
H	2.243896110511	-1.561885721631	-4.610534388958
C	2.123819612065	-5.271221133701	-1.606074428773
H	3.104351018998	-4.844159844623	-1.865054840090
H	1.986779160011	-6.189959235259	-2.199428194792
H	2.141866588500	-5.560074239183	-0.544767955881
C	-0.890676599740	-4.771648702824	-1.339248437133
H	-0.987765498015	-4.667391163646	-0.249786424648
H	-0.923549422506	-5.847429396164	-1.579959890764
H	-1.757088516026	-4.296056259083	-1.818537688928
C	0.582867619778	-3.969563517211	-3.879613999126
H	-0.095889928321	-3.149402787939	-4.150234559107
H	0.174327903156	-4.911964219938	-4.278404983037
H	1.561400947554	-3.792858070993	-4.349468629096
C	-2.120644106109	-1.368159017742	2.466678280310
C	-2.469233589738	-0.137570800250	3.060074611538
C	-1.762343275616	0.311198205399	4.179378017280
H	-2.055441994773	1.260251099864	4.639407955057
C	-0.703517185026	-0.412769083788	4.729388970184
C	-0.360363307143	-1.620981063310	4.118231093159
H	0.469976296637	-2.208543047220	4.521908009601
C	-1.048930065940	-2.111305605701	3.006751041729
C	-3.590375323565	0.677260061943	2.495355353662
H	-4.568430919290	0.200397060020	2.667919720406
H	-3.488440499177	0.778659040591	1.403529100951

H	-3.619852694774	1.681515227863	2.940679598194
C	0.019493535435	0.074813170439	5.951727549784
H	-0.019249899688	1.171286424711	6.032871457125
H	1.076487932269	-0.229590648150	5.944532248971
H	-0.430577349071	-0.335615154268	6.871424634695
C	-0.663321279801	-3.419836658713	2.392779341853
H	0.223836436782	-3.846265147301	2.880423821832
H	-0.444834875337	-3.298460498445	1.319740992387
H	-1.481380158043	-4.154095477003	2.457673167140
C	-5.798469215425	-2.025755655941	0.846902265811
H	-6.018628985251	-1.059906482762	1.326304055002
H	-6.687682133173	-2.668962486742	0.948686268783
H	-5.638094130152	-1.838801306584	-0.227039893692
C	-4.516603029286	-3.076256180487	3.449096212815
H	-3.644194134324	-3.569575743892	3.904573755436
H	-5.405648917748	-3.695178523919	3.649298380689
H	-4.648943975950	-2.108466952862	3.956596030359
C	-4.091100769779	-4.526832430586	0.760340195227
H	-3.898903176591	-4.401463263580	-0.316793074878
H	-5.009336085421	-5.125668699667	0.874943866353
H	-3.252788162930	-5.102255738940	1.181578246637
H	-1.900380230033	-1.636077665748	-3.954039419539
O	-1.220784565226	-0.979058077006	-4.218360739237
H	-2.996224370562	-2.512547451370	-2.366151764030
O	-3.039077928568	-2.835218389672	-3.302174586601
H	-0.996767386376	-1.229241179808	-5.120336543979
H	-3.966820503415	-2.732594695971	-3.541339297800

149
H2O_Int_3, G=-4634.92542

C	3.333795768715	1.831156524910	2.137642770003
C	3.319842162154	0.463914653436	1.788427729687
C	2.930216536386	-0.480751648229	2.761311705857
C	2.551139603758	-0.048063892140	4.033569572306
C	2.544928306024	1.301585342333	4.388511235535
C	2.942250960222	2.223624963510	3.420435652718
N	3.739028126434	0.042594142759	0.499272092647
Si	5.481248449469	-0.082585363529	0.182349995722
C	6.062302409344	1.108719992152	-1.157472956977
C	2.967177527878	-1.940755723896	2.442731416504
C	2.143898752393	1.738435639379	5.767718701781
C	3.812385482464	2.851478251264	1.153950395909
C	5.977557251748	-1.793097425777	-0.429818771335
C	6.386426762372	0.244119235038	1.797633836061
Si	2.607502865270	-0.202047073533	-0.906155038462
Si	0.665034678631	1.107952771992	-0.834834440005
N	0.419205778810	2.692814798433	-1.589275613345
C	-0.655815718574	3.532713485921	-1.167782467622
C	-0.480859643383	4.354788170092	-0.031021260312
C	-1.464440455263	5.285268335906	0.300620780511
C	-2.629576565768	5.436973374561	-0.457488037128
C	-2.802203244948	4.592981162189	-1.551286747984
C	-1.845920961493	3.637405872963	-1.911669768388
C	0.776332710957	4.258385629760	0.771905930560
C	-3.678565335880	6.436314716695	-0.066442203332
C	-2.094898111173	2.762596743668	-3.101966318048
Si	0.677932132289	-1.465947118440	-0.468207758302
N	0.564580168355	-3.204828459500	-0.766547354181
C	-0.664751262365	-3.900040471212	-0.559977810801
C	-1.573535490738	-4.085034084965	-1.618686001794
C	-2.729632910965	-4.842889056006	-1.400754131161
C	-3.003226972683	-5.441078338504	-0.172587144596
C	-2.083315061306	-5.251426470904	0.863203060590

C	-0.927459639481	-4.490296954232	0.695693005299
C	-1.288175761357	-3.547457336759	-2.987752746515
C	-4.262020058431	-6.227364517967	0.049930008876
C	0.047297187557	-4.330968120234	1.820393114809
Si	-1.036953909009	-0.080398174343	0.094660025164
N	-2.278802600459	0.116346947804	1.340190000563
C	-3.665477029862	0.147157126843	1.016924708447
C	-4.340082025153	1.377161658779	0.859384091922
C	-5.683061956808	1.372923195155	0.479031556152
C	-6.390696390035	0.188440793774	0.253300184374
C	-5.712651065742	-1.016939454208	0.436195358991
C	-4.368162485654	-1.060749623953	0.824307048807
C	-3.626844526879	2.665077252232	1.111952949731
C	-7.838843085982	0.219678495626	-0.141419665748
C	-3.680915048311	-2.369964338659	1.044819718279
Si	1.916039145218	-4.208017017167	-1.373998017652
C	2.535419223656	-3.612407168340	-3.049415384370
C	1.277225368651	-5.964188613291	-1.559696025078
C	3.363035167348	-4.195054394378	-0.189952759095
Si	-1.788205791013	0.308919302922	3.047128058250
C	-3.364419477107	0.411586976407	4.062585484106
C	-0.801519977659	-1.185147938347	3.603945390850
C	-0.719510529423	1.832993639714	3.250421726625
Si	1.453020983151	3.371342818743	-2.889406658494
C	1.170914731976	2.497408561691	-4.530632663852
C	0.990532855132	5.182603724964	-3.078428961992
C	3.275293209976	3.249303815164	-2.479174988033
H	-3.429301190545	-4.980973919827	-2.231227177876
H	-2.272214857860	-5.711033114477	1.838292682776
H	-0.919948156875	-4.350116090478	-3.649783580904
H	-0.517756832436	-2.770258815863	-2.957286676206
H	-2.187672586164	-3.124720573945	-3.455925959781
H	-4.084148246568	-7.100162631870	0.695727960473
H	-4.689261873995	-6.584333589668	-0.898107210212
H	-5.032316024685	-5.611691322120	0.546098248151
H	-0.359482021011	-4.733020536385	2.758322693913
H	0.305069288371	-3.275312747805	1.978974063304
H	0.993510079462	-4.853925964985	1.608915604691
H	0.900875785261	-6.363436165042	-0.605778855104
H	2.102134078234	-6.610899165741	-1.899998151669
H	0.460018004000	-6.036731105764	-2.292568099281
H	3.703543023453	-3.167539491551	-0.012228546149
H	4.205924806259	-4.759923128889	-0.618980220406
H	3.104539336905	-4.644215034462	0.780234410769
H	1.732665915114	-3.644007556213	-3.803855614010
H	3.344529204256	-4.273719581421	-3.399093280866
H	2.945947698455	-2.593609643250	-2.962210783351
H	-6.191861976044	2.333522176475	0.351470866854
H	-6.243731518610	-1.961316756506	0.283331959112
H	-2.658263733832	2.700996330180	0.594787677408
H	-3.416152840723	2.797771640682	2.184731582537
H	-4.228305618211	3.520043889943	0.781706670075
H	-8.179123725538	-0.759583813130	-0.506760482200
H	-8.025409116638	0.962756068397	-0.931822773429
H	-8.476828811995	0.493775977361	0.715121111985
H	-3.246819372925	-2.426057067876	2.054026959954
H	-2.844530077198	-2.525394160026	0.346409313343
H	-4.377104109856	-3.208113824499	0.919553128713
H	-1.376612717234	-2.116910691094	3.498672843684
H	-0.507498665343	-1.073050115214	4.659932317666
H	0.120306244452	-1.272147547732	3.010881027288
H	0.247693674937	1.681780228953	2.746948114002
H	-0.507181621198	2.017135797460	4.315488008986

H	-1.191513523280	2.731215426637	2.827097024626
H	-3.985403841163	1.281353946440	3.803448875074
H	-3.103804091568	0.484480004470	5.130625675195
H	-3.980452701413	-0.490461298904	3.924094755629
H	-1.306279488956	5.925000177220	1.174318808788
H	-3.715372999246	4.672013575752	-2.149839259686
H	0.735617369391	4.908131629737	1.656795140866
H	1.650902504010	4.546258483704	0.168951919798
H	0.962737804792	3.229262102868	1.115585542618
H	-3.228764821513	7.382760089479	0.269137155434
H	-4.294460766538	6.059433214312	0.768310022279
H	-4.357401076812	6.658296986629	-0.902288590939
H	-3.115097813180	2.349212961503	-3.075446081306
H	-1.369754518348	1.941933840202	-3.113483895284
H	-1.990589184703	3.324999228644	-4.044673245371
H	-0.054640902487	5.317332897007	-3.394379461086
H	1.641973350370	5.643081179865	-3.838741815537
H	1.124134434738	5.733939730871	-2.135085174410
H	3.548069663219	3.889682677694	-1.627982249302
H	3.861561303723	3.578097179187	-3.352585615411
H	3.566140676666	2.214345642727	-2.246568762538
H	1.322320417914	1.410185607835	-4.456687376377
H	1.888235795623	2.885835022777	-5.272140963107
H	0.159558323381	2.681223940757	-4.922796669979
H	2.253443694506	-0.797685278976	4.773278728119
H	2.949558427882	3.290053674341	3.667248877074
H	2.590367521428	-2.545575486663	3.279285759943
H	3.991383652894	-2.272873487595	2.210263921156
H	2.365135965171	-2.165264594896	1.549469558793
H	1.322178795405	1.121336558492	6.162376980575
H	1.815622865636	2.788087937935	5.777727224819
H	2.983855673791	1.650191998373	6.477466887920
H	4.904948425788	2.796383677543	1.023197217372
H	3.572591493174	3.870079862451	1.486782196948
H	3.369433014103	2.680825958882	0.164899881586
H	5.829092388530	-2.569680407624	0.334830925724
H	7.045840662834	-1.784783552028	-0.702462076914
H	5.404196267836	-2.077555143526	-1.325765701305
H	6.199702802969	1.254107313970	2.191039446306
H	7.471610603063	0.127995163061	1.646361218911
H	6.069921085632	-0.472258935313	2.571763353106
H	5.577557821538	0.865526256602	-2.116146645783
H	7.151284000035	1.008495716043	-1.297567284061
H	5.840476536502	2.160076870006	-0.926860760931
H	-3.218487979286	-0.345045347839	-1.058644543698
H	-1.987762531437	-0.361554833485	-2.280485452926
O	-2.272623474059	-0.253716216853	-1.271361567417
O	-1.557070736369	-0.479453846136	-3.630063482888
H	-1.710071413892	0.320289692198	-4.145739119268
H	-0.574152620932	-0.655318885093	-3.704712769723
O	1.036629874157	-0.832140456514	-3.905490635537
H	1.435299195786	-1.660612430606	-4.198148288722
H	1.573453797124	-0.580254973504	-3.119501776155

149
H2O_TS_1, G=-4634.924778

C	-3.368019011821	-1.804510980419	2.156248024164
C	-3.326621988856	-0.441707981116	1.792217024164
C	-2.920773972898	0.505302012045	2.756746024164
C	-2.547441980097	0.078047005753	4.032403024164
C	-2.563957002783	-1.268137993968	4.400168024164
C	-2.980845018345	-2.191637986943	3.442116024164
N	-3.736548981837	-0.025188974208	0.498292024164

Si	-5.475144979311	0.124729055089	0.173094024164
C	-6.071355999545	-1.075996934864	-1.151478975836
C	-2.938552948326	1.963451012344	2.428221024164
C	-2.167187010040	-1.698791000654	5.782555024164
C	-3.873636029031	-2.825744971898	1.187107024164
C	-5.934685950475	1.835888062833	-0.465409975836
C	-6.392313984133	-0.161449929455	1.789249024164
Si	-2.597270977760	0.216734006593	-0.900607975836
Si	-0.647636999726	-1.086739026261	-0.852237975836
N	-0.399062026249	-2.660666030450	-1.635316975836
C	0.641601959101	-3.530059047987	-1.185252975836
C	0.419819945530	-4.335359044249	-0.045374975836
C	1.369435929449	-5.289667060252	0.319613024164
C	2.541016926082	-5.489470079995	-0.414712975836
C	2.756280939899	-4.669507083622	-1.520291975836
C	1.841121956482	-3.685488068200	-1.906782975836
C	-0.850234052125	-4.196224022847	0.730290024164
C	3.552867908810	-6.514384097046	0.006406024164
C	2.169985971257	-2.808665073742	-3.077029975836
Si	-0.672260956553	1.475240974154	-0.459900975836
N	-0.537893927287	3.211938971890	-0.760203975836
C	0.708581083989	3.881095950885	-0.571259975836
C	1.620996086275	4.016720935509	-1.634345975836
C	2.792756098733	4.755996915763	-1.437202975836
C	3.079226109284	5.382109910936	-0.225948975836
C	2.158290106833	5.236684926455	0.816043024164
C	0.987430094321	4.494205946186	0.669973024164
C	1.325418076649	3.445494940490	-2.987945975836
C	4.353141122222	6.149917889469	-0.026157975836
C	0.015141092357	4.377655962570	1.802424024164
Si	1.045941020190	0.095096945200	0.100848024164
N	2.281180016558	-0.120424075616	1.347765024164
C	3.666304015697	-0.171532098957	1.021490024164
C	4.320478994817	-1.410574109981	0.851159024164
C	5.662087994587	-1.424225132589	0.465844024164
C	6.387821014388	-0.249204144819	0.248509024164
C	5.730374034852	0.965156866260	0.446967024164
C	4.388292035887	1.026589888876	0.840663024164
C	3.589204973263	-2.689642097658	1.099074024164
C	7.833297013529	-0.300159169177	-0.153960975836
C	3.723179058084	2.343831900084	1.081042024164
Si	-1.873081909955	4.240430994389	-1.359510975836
C	-2.516686919786	3.657062005235	-3.030102975836
C	-1.197931880617	5.981409983012	-1.558677975836
C	-3.313406909661	4.257878018661	-0.167044975836
Si	1.782240013360	-0.310179067208	3.051966024164
C	3.353303011121	-0.443081093683	4.071566024164
C	0.817186038797	1.199306949055	3.606597024164
C	0.688811987948	-1.818202048782	3.245588024164
Si	-1.468303037677	-3.338828012432	-2.907561975836
C	-1.245242022767	-2.454077016191	-4.554341975836
C	-0.997901068096	-5.143941020359	-3.128444975836
C	-3.280403035919	-3.234535981895	-2.449458975836
H	3.494584100439	4.857234903936	-2.271190975836
H	2.359226114906	5.715753923069	1.779285024164
H	1.002427090045	4.240427945933	-3.681999975836
H	0.518373064203	2.706902954090	-2.939609975836
H	2.210319068596	2.967605925578	-3.431268975836
H	4.194063137222	7.040010892149	0.600610024164
H	4.782637127746	6.477706882231	-0.983668975836
H	5.114356111800	5.531435876641	0.480439024164
H	0.440200099113	4.778528955407	2.732739024164
H	-0.273629925262	3.332119967436	1.974920024164

H	-0.916023898380	4.927305978262	1.591360024164
H	-0.812197873863	6.382239976512	-0.609301975836
H	-2.008096869471	6.642820996665	-1.905995975836
H	-0.379210879843	6.027325969216	-2.292278975836
H	-3.679925926841	3.238413024837	0.005142024164
H	-4.143177899724	4.847557032644	-0.588274975836
H	-3.038655902330	4.692908014031	0.805022024164
H	-1.718832919515	3.673122991790	-3.790280975836
H	-3.314227908350	4.335657018675	-3.373233975836
H	-2.947192936810	2.646839012490	-2.942466975836
H	6.155211978286	-2.391561140899	0.327902024164
H	6.276506050643	1.902254857057	0.302484024164
H	2.615514972950	-2.708190081250	0.590552024164
H	3.384977970948	-2.827024094217	2.172621024164
H	4.174938958742	-3.551327107529	0.758088024164
H	8.191936030061	0.680846824779	-0.496284975836
H	8.000787001291	-1.026381172000	-0.964139975836
H	8.469463008321	-0.609200179898	0.691917024164
H	3.308292059017	2.399171907076	2.098553024164
H	2.876916060959	2.514436914345	0.398763024164
H	4.428827072058	3.173071888193	0.949395024164
H	1.407938054341	2.121705939099	3.504944024164
H	0.515342036981	1.091490954141	4.660800024164
H	-0.099506959465	1.302445964502	3.008064024164
H	-0.272873009257	-1.652322032576	2.736052024164
H	0.466949984853	-2.001857045044	4.308768024164
H	1.150727972713	-2.722257056566	2.823235024164
H	3.960058996314	-1.321729103907	3.808435024164
H	3.089376009860	-0.517919089235	5.138637024164
H	3.984808026157	0.449174895675	3.939495024164
H	1.174331918959	-5.912171056964	1.198196024164
H	3.672528937841	-4.791634099062	-2.106527975836
H	-0.844550062892	-4.835181022943	1.623826024164
H	-1.719867056735	-4.469803008192	0.113871024164
H	-1.017063034637	-3.158439020036	1.058443024164
H	3.070044893597	-7.417157088909	0.408937024164
H	4.210140915472	-6.119091108122	0.800206024164
H	4.196698903732	-6.815751107895	-0.832416975836
H	2.768646985793	-1.946065083830	-2.741803975836
H	1.250880977554	-2.435003058254	-3.545936975836
H	2.758347962183	-3.347175083657	-3.833290975836
H	0.038468929861	-5.265199037823	-3.477009975836
H	-1.667753075790	-5.600540009071	-3.874972975836
H	-1.098132077590	-5.707360018670	-2.188174975836
H	-3.524225046766	-3.878195977786	-1.592065975836
H	-3.886671041551	-3.568749971679	-3.306983975836
H	-3.575090018531	-2.202704976929	-2.208282975836
H	-1.372091004406	-1.364494014053	-4.469024975836
H	-2.006659028992	-2.823458003360	-5.260756975836
H	-0.261939026209	-2.658295032761	-5.006476975836
H	-2.236565967435	0.829453000515	4.764816024164
H	-3.008215036268	-3.255249986482	3.699515024164
H	-2.555240938119	2.569106005885	3.261200024164
H	-3.958489942546	2.306421029532	2.192826024164
H	-2.333242944752	2.175509002144	1.533933024164
H	-1.331040999869	-1.095215014745	6.167740024164
H	-1.861311027839	-2.755036005809	5.802670024164
H	-3.002378008123	-1.585025986580	6.494284024164
H	-4.970029028123	-2.771882953422	1.089689024164
H	-3.623807046194	-3.844285976108	1.512877024164
H	-3.461251026129	-2.653586978848	0.185793024164
H	-5.786423937309	2.617201060335	0.294461024164
H	-6.998064950355	1.843024080753	-0.756456975836

H	-5.340786945992	2.101909052825	-1.353678975836
H	-6.225728001103	-1.168489932262	2.198810024164
H	-7.474630981883	-0.027913911216	1.631888024164
H	-6.065813971960	0.560928065043	2.553617024164
H	-5.580125995805	-0.854085943141	-2.112025975836
H	-7.158187997604	-0.960847916549	-1.296627975836
H	-5.866827017259	-2.127173938310	-0.904575975836
H	3.213489025209	0.392948908673	-1.071416975836
H	1.933544024562	0.354553930242	-2.288021975836
O	2.262305024029	0.322942924702	-1.264095975836
O	1.464375024006	0.321557938148	-3.577191975836
H	1.486770008627	-0.591069062229	-3.890172975836
H	0.502865028568	0.592300954351	-3.652812975836
O	-1.093553967103	0.849183981253	-3.886054975836
H	-1.453985952980	1.687260987327	-4.199217975836
H	-1.660788970930	0.622073990812	-3.111626975836

149
H2O_Int_4, G=-4634.926965

C	-3.444271088884	-1.911680242811	2.129903672607
C	-3.391872854743	-0.535736642929	1.824029101611
C	-3.000136121427	0.371826454703	2.832924599482
C	-2.647789197602	-0.109003242867	4.094429528979
C	-2.672440800147	-1.470192406325	4.405637334616
C	-3.076824300081	-2.352189468631	3.405259797411
N	-3.771628932377	-0.064777039275	0.539422656979
Si	-5.500322261968	0.089737527611	0.165544793549
C	-6.038279691617	-1.069419577789	-1.219839916432
C	-3.007581511891	1.843203282016	2.564548093556
C	-2.289717187821	-1.955189694579	5.773898337919
C	-3.943688284439	-2.893854166062	1.116078951288
C	-5.940738737679	1.823253228117	-0.423480720296
C	-6.465152708383	-0.256638814730	1.740932619306
Si	-2.598562788933	0.239009106323	-0.810506030591
Si	-0.627746610507	-1.019488022963	-0.745858656946
N	-0.115344213873	-2.362283485061	-1.924420896781
C	0.812591390517	-3.328133548989	-1.372819029302
C	0.428282278314	-4.118589291837	-0.264838713932
C	1.277570204840	-5.124803861977	0.199488105328
C	2.499616418757	-5.405844767380	-0.408967968227
C	2.861159931424	-4.621600437409	-1.501829640970
C	2.056910176243	-3.586371796459	-1.988057513743
C	-0.907249737362	-3.947829370866	0.383917127404
C	3.407222925085	-6.476282661790	0.120129548645
C	2.562803264353	-2.795790141599	-3.157446295651
Si	-0.687780326059	1.489576083084	-0.289445076264
N	-0.536443239230	3.190424153853	-0.756871829822
C	0.727281378004	3.839396158614	-0.624608401190
C	1.633176499473	3.868646865562	-1.700698305975
C	2.821724915932	4.594341321947	-1.572790676413
C	3.132663454460	5.306173353531	-0.415141411697
C	2.224388888833	5.254197669935	0.645928747443
C	1.038859919304	4.523684243467	0.570005073838
C	1.310827718274	3.181426451645	-2.992534145141
C	4.425993640254	6.056519185316	-0.286889152946
C	0.095500897266	4.481918757680	1.732837090494
Si	1.127680818546	0.164001715980	0.100184113052
N	2.270338004569	-0.094946170107	1.414218149466
C	3.667046663940	-0.134403076058	1.135671954896
C	4.335090779702	-1.367113715434	0.979379506448
C	5.695799750151	-1.369130590585	0.667032987475
C	6.424807621938	-0.187677457774	0.506129714778
C	5.750555589686	1.020375449337	0.686488753433

C	4.389321246915	1.069000412381	1.004901052954
C	3.599317977891	-2.654254972987	1.168362642935
C	7.879830430809	-0.223659392971	0.137419737244
C	3.700178657574	2.378781427366	1.213604008207
Si	-1.881164147747	4.222911381347	-1.312874381894
C	-2.574784502887	3.657635324372	-2.970987925244
C	-1.206632828869	5.960732617653	-1.539583377962
C	-3.284565792488	4.224987771571	-0.076054837729
Si	1.691279128745	-0.328954416208	3.083852133253
C	3.209874932113	-0.491914983632	4.176059433401
C	0.702166242515	1.167935814620	3.636028089807
C	0.586592809490	-1.839439000258	3.180461123825
Si	-1.240095880568	-3.122837050197	-3.144303284368
C	-1.045628478614	-2.279861283323	-4.812664151836
C	-0.756712995588	-4.925686931140	-3.337255924895
C	-3.046035384863	-3.010561503480	-2.665116705331
H	3.519823050539	4.611946618078	-2.415680519529
H	2.451514825862	5.791770446657	1.571700004213
H	0.864084219657	3.887899464289	-3.713578995883
H	0.583283521125	2.378335001901	-2.832378251377
H	2.213518466178	2.763128037160	-3.460172146325
H	4.306171638744	6.968203592380	0.316855936419
H	4.825449053807	6.346150624747	-1.269481921429
H	5.194446945500	5.438432001787	0.209106866855
H	0.555935778451	4.917415747839	2.630184544568
H	-0.206453151511	3.450118478449	1.964788480617
H	-0.831042185847	5.040606709952	1.525273049984
H	-0.807787749723	6.376913894405	-0.602728455436
H	-2.017158501914	6.619929890470	-1.890188448287
H	-0.395340875516	5.989309498061	-2.282958697781
H	-3.629223086372	3.199600993957	0.110055123993
H	-4.137906155039	4.801470407482	-0.466989860338
H	-2.981742751312	4.663723327291	0.886007145290
H	-1.798930552522	3.662738400241	-3.755610226268
H	-3.361770759893	4.356972379844	-3.296782402475
H	-3.032833002496	2.660144692995	-2.882973653356
H	6.201943499425	-2.331906933901	0.544992818989
H	6.298985061693	1.962073019803	0.586953611447
H	2.643639426377	-2.658203762657	0.626956422247
H	3.359163470023	-2.823664703040	2.230068231559
H	4.198700971863	-3.504902274270	0.822289378015
H	8.363996268582	0.747825852924	0.311441988507
H	8.014766769300	-0.474384385310	-0.928100058735
H	8.423547508699	-0.984140022357	0.718329060773
H	3.194602071366	2.414595988981	2.190004103396
H	2.919711404668	2.553814301886	0.458101322095
H	4.409320501365	3.214119852177	1.157558057216
H	1.301791765843	2.089633468963	3.594649353594
H	0.346991440231	1.028733508621	4.669678812561
H	-0.181258009907	1.297178929004	2.994006619257
H	-0.354423391206	-1.649992318250	2.641223369111
H	0.324997295583	-2.065677741479	4.226047542118
H	1.063590319882	-2.726584747501	2.738904725845
H	3.828514912655	-1.364348584342	3.920061699307
H	2.895980618096	-0.592178767120	5.227396116905
H	3.847156654292	0.402384223736	4.094912705644
H	0.953890695259	-5.722571079927	1.056647884970
H	3.814873799218	-4.812971676754	-2.002811473311
H	-1.013744432466	-4.613553362473	1.250631268365
H	-1.725110047016	-4.167728162408	-0.317824618475
H	-1.073417526773	-2.917898103585	0.746884828815
H	2.838098404926	-7.304448892284	0.567062930714
H	4.070406855912	-6.076893258369	0.906819182928

H	4.050372896294	-6.888836771626	-0.670699077824
H	2.872022447687	-1.787953633186	-2.843954162301
H	1.811231801914	-2.676800695771	-3.949770399777
H	3.440666172093	-3.285082683183	-3.599714970936
H	0.279088696480	-5.041486213162	-3.689127491267
H	-1.426152642826	-5.388280044262	-4.080708045519
H	-0.850752671275	-5.485633512530	-2.395109567291
H	-3.292202814833	-3.629900857753	-1.791160510743
H	-3.652073331816	-3.369609146200	-3.512999756772
H	-3.342473653077	-1.973654689194	-2.445734750563
H	-1.386069396427	-1.234608092777	-4.803859812588
H	-1.670851539776	-2.828360969483	-5.536200426239
H	-0.010935213998	-2.304862010727	-5.186841012042
H	-2.347734280761	0.610555306553	4.862555056389
H	-3.111554358689	-3.425048988562	3.619953821645
H	-2.668408470678	2.413376841101	3.440468816050
H	-4.014541787737	2.194438623375	2.289484299605
H	-2.354607409741	2.090992684461	1.712816577757
H	-1.362841225034	-1.475572571264	6.125972835438
H	-2.133023228952	-3.043385636890	5.786142756892
H	-3.072379347916	-1.724305248864	6.515786869378
H	-5.045365265653	-2.891968144636	1.072417464586
H	-3.630488198667	-3.917457261864	1.362264849042
H	-3.588865954328	-2.635216209343	0.110944992958
H	-5.795346638923	2.576951355419	0.364523393172
H	-6.999963223317	1.849677728308	-0.728225169865
H	-5.332219618518	2.114492626775	-1.293696876544
H	-6.308837125843	-1.277428611185	2.119009508148
H	-7.542535809285	-0.118787143222	1.556025135914
H	-6.163274067218	0.438440825510	2.539910279200
H	-5.508337535067	-0.814674394105	-2.151225711986
H	-7.118876978124	-0.957382481517	-1.407275540132
H	-5.835930892554	-2.126410827404	-0.996390690585
H	3.154617050191	0.405355217946	-1.037189573614
H	1.512052585416	0.105557708887	-2.572420753762
O	2.209201318187	0.390373903722	-1.252262021332
O	0.939589586384	-0.304522480672	-3.322047228866
H	0.592362669045	-1.173143346520	-2.869476031363
H	0.091481358880	0.253548261114	-3.486136870684
O	-1.295307387998	0.811650770125	-3.757496819185
H	-1.435892686851	1.754315071836	-3.912955459197
H	-1.906792245074	0.603602561995	-2.998327515291

149
H2O_TS_2, G=-4634.924488

C	-3.398143409816	-1.859747871107	2.194153177954
C	-3.362611772144	-0.491882269790	1.855843944552
C	-2.982702037031	0.445240148489	2.841436522816
C	-2.625200645786	-0.000223483011	4.114298869721
C	-2.633418134858	-1.353694740919	4.458549917359
C	-3.027964575278	-2.264399162577	3.480683792290
N	-3.741506995095	-0.058186772911	0.553022719502
Si	-5.468605907094	0.099081350787	0.173593849509
C	-6.009545092199	-1.082850451175	-1.189735789975
C	-3.002957589191	1.909590775786	2.538462719308
C	-2.229444101919	-1.799535412908	5.833736428764
C	-3.875469958178	-2.872365575036	1.201142321025
C	-5.890670779801	1.824342617370	-0.452216297093
C	-6.435386341938	-0.214702330025	1.752933139798
Si	-2.518246266767	0.199538318029	-0.747258031774
Si	-0.585402687492	-1.086631274483	-0.733435972875
N	-0.245958217211	-2.519322047301	-1.773366889160
C	0.763413768288	-3.424834909455	-1.290916817666

C	0.484016822151	-4.248532235432	-0.178280800791
C	1.408076870047	-5.220917843420	0.209643849431
C	2.606450797634	-5.419384883229	-0.476221215850
C	2.875130553260	-4.581262626245	-1.557707204139
C	1.990138668040	-3.580685912756	-1.967964784071
C	-0.815048062902	-4.125370947590	0.552614466460
C	3.594974111911	-6.456417709525	-0.030740547867
C	2.370820268642	-2.692170922843	-3.111704205719
Si	-0.641464236953	1.491476150790	-0.299377486157
N	-0.525699656652	3.204166960791	-0.751635104204
C	0.739587244688	3.857330917588	-0.637653903943
C	1.621958274971	3.899634194308	-1.732772006764
C	2.810911891485	4.626934875899	-1.621982371683
C	3.144493520539	5.326823766495	-0.463047812914
C	2.257328221749	5.264563457598	0.614952481214
C	1.070457944190	4.534342244638	0.555098005081
C	1.272959905947	3.226736530694	-3.025043656385
C	4.440111658336	6.075994264807	-0.353280085923
C	0.141611645676	4.494651530881	1.729683862174
Si	1.176183101823	0.154190389639	0.010655633088
N	2.308166459216	-0.082261345459	1.335896694694
C	3.704822219551	-0.134720941045	1.055435516985
C	4.365637169646	-1.373445565736	0.924144722779
C	5.726914011810	-1.388664772202	0.613367094946
C	6.462240317482	-0.214788712599	0.430907746673
C	5.794686400379	1.000408620189	0.589218835610
C	4.433784494276	1.062086367573	0.904692278630
C	3.624353928310	-2.653246545369	1.140853169990
C	7.916049717858	-0.263488293737	0.058798623727
C	3.752509856246	2.379259905736	1.092969211425
Si	-1.864400032896	4.244462090457	-1.293660023465
C	-2.592539599478	3.669073098104	-2.939233582702
C	-1.182106461666	5.973156126647	-1.554944860176
C	-3.264466232056	4.278431489155	-0.054559872067
Si	1.737728549151	-0.288968021255	3.011589847369
C	3.262615662582	-0.439441695419	4.097580199658
C	0.756409948273	1.214756065102	3.560300594111
C	0.624860428410	-1.790668427372	3.148862833003
Si	-1.357839952822	-3.265280643601	-2.978402093669
C	-1.264489600570	-2.416165070658	-4.657870318776
C	-0.834811980356	-5.052538409492	-3.212141136471
C	-3.153381815234	-3.203658128943	-2.447031999530
H	3.491714049525	4.654201496236	-2.478521366425
H	2.501161379877	5.795562078903	1.540239668998
H	0.778905544697	3.931571174173	-3.716244695611
H	0.580367135157	2.395556395700	-2.854275692287
H	2.168261046353	2.841513009659	-3.532357098667
H	4.336697355222	6.972668378937	0.275366163011
H	4.811749433836	6.389317253420	-1.339523415258
H	5.222350416977	5.446786236494	0.105712952778
H	0.622811053999	4.905723436721	2.627651996770
H	-0.182470421809	3.467446330251	1.949443069737
H	-0.773211217740	5.079899014999	1.542168860172
H	-0.756718962010	6.391891177766	-0.630974908379
H	-1.995764602247	6.636628386161	-1.889770268349
H	-0.388499045803	5.989669742862	-2.317299331955
H	-3.632547129874	3.262221146114	0.135088695801
H	-4.104346278424	4.873960043771	-0.446460303349
H	-2.950305319755	4.712467513728	0.905874793685
H	-1.809297776918	3.548536365278	-3.708188472676
H	-3.290591230012	4.431541015934	-3.320690548744
H	-3.170368381309	2.741054446264	-2.808678214797
H	6.228065608629	-2.356257648508	0.510552613820

H	6.348879835299	1.937084495124	0.474504807261
H	2.668036070417	-2.666048470582	0.600716443625
H	3.385525617234	-2.799720476825	2.206279237507
H	4.219628329911	-3.513502489318	0.812179828371
H	8.425445809131	0.681124784730	0.297896133809
H	8.044167152407	-0.441765254571	-1.022255237855
H	8.439712678394	-1.076194497654	0.584195709624
H	3.245616714074	2.432873674703	2.067863631627
H	2.974599567855	2.548045054674	0.333400743292
H	4.467515416277	3.208923870895	1.026524501426
H	1.351021945737	2.137970532809	3.492791170782
H	0.425414128194	1.088140358451	4.603750104796
H	-0.141991574904	1.330066713444	2.936796425859
H	-0.325387319733	-1.600820430749	2.626108509453
H	0.384235357414	-2.000228857777	4.203100199727
H	1.085482814459	-2.687841939229	2.710562496746
H	3.877815745121	-1.317442043628	3.852964331441
H	2.953926490214	-0.521028987278	5.152099062515
H	3.901955041869	0.451535359458	3.997924601695
H	1.169201629199	-5.855164563767	1.068667764272
H	3.815660317203	-4.699199239021	-2.104393325071
H	-0.835974456380	-4.775247367157	1.437814729218
H	-1.664297673682	-4.397233941104	-0.092576293096
H	-1.000360160347	-3.093527804565	0.895545889403
H	3.094220094401	-7.323440122913	0.424327906993
H	4.284164118482	-6.045441715190	0.727288878597
H	4.209393810764	-6.815855108988	-0.868975291670
H	2.478461226811	-1.653421287072	-2.766731910960
H	1.622852278456	-2.701551860961	-3.918481227625
H	3.330489049944	-3.003393423615	-3.545563070529
H	0.192379825362	-5.134493621221	-3.597765820807
H	-1.514060194482	-5.529569808848	-3.937140724387
H	-0.879697609160	-5.621940562997	-2.271874449939
H	-3.360847467855	-3.875192356618	-1.601906575060
H	-3.787403228208	-3.517710347387	-3.291948301041
H	-3.454030413420	-2.184745352105	-2.161371585957
H	-1.715794575183	-1.414410543985	-4.660261280062
H	-1.842742885035	-3.033386186432	-5.365306067122
H	-0.241893003517	-2.336212180427	-5.056184367402
H	-2.333184595141	0.741524897090	4.864185269926
H	-3.050016002340	-3.331878487081	3.721170905345
H	-2.661940220645	2.502029498775	3.398683733779
H	-4.014642866028	2.247830496207	2.264219726418
H	-2.356536112849	2.142162174618	1.678104300756
H	-1.216670512358	-1.446570027536	6.087412413630
H	-2.234101031652	-2.895392693259	5.919487229395
H	-2.909964757651	-1.399395693617	6.602814162556
H	-4.974660156322	-2.858981661358	1.116696884203
H	-3.582738793153	-3.889234914139	1.495432256670
H	-3.480743359287	-2.657645817227	0.201176611442
H	-5.757252510687	2.590993930290	0.325185847689
H	-6.943056920888	1.851766165980	-0.779214399406
H	-5.263999005086	2.098718677909	-1.315123611404
H	-6.280958682245	-1.229919935257	2.146731179821
H	-7.512353325349	-0.077958220458	1.565016546772
H	-6.132327037507	0.492183594499	2.540886119152
H	-5.481137987271	-0.850632276920	-2.127910859806
H	-7.089847133756	-0.969746887515	-1.378006958333
H	-5.811931681225	-2.135940625912	-0.944631234373
H	3.150840295302	0.412458225127	-1.152733475933
H	1.186315871037	0.288757885954	-2.856802294951
O	2.202980982469	0.389289071673	-1.350049543390
O	0.568979364165	-0.081583089849	-3.531918188921

```
 H         0.439677831347     -0.989754707178     -3.192447614960
 H        -0.744282408836      0.438480116344     -3.555352717810
 O        -1.771994392035      0.729945800467     -3.641729546678
 H        -1.824168411694      1.697497773093     -3.706998144906
 H        -2.317487144551      0.493201760494     -2.679278105589

149
H2O_Int_5, G=-4634.985989
 C        -3.572167414085     -1.088162248269      2.411991288681
 C        -3.331486972554      0.224546105660      1.959216856485
 C        -2.872468316139      1.199616242444      2.865874727321
 C        -2.648784603625      0.843292713447      4.197332935710
 C        -2.872828420315     -0.453009021117      4.664562347213
 C        -3.338917817382     -1.401415991709      3.753246679386
 N        -3.612614071526      0.583691961480      0.610283562723
 Si       -5.308389417502      0.957730729293      0.194458220513
 C        -6.041871934240     -0.255359674772     -1.041380757155
 C        -2.679523124700      2.613040083365      2.410728922670
 C        -2.583143969511     -0.822214304410      6.090147339169
 C        -4.127909693003     -2.122910248077      1.481980979858
 C        -5.509759863215      2.659440919783     -0.577710476539
 C        -6.286459058927      0.921335825236      1.797742520002
 Si       -2.330554142855      0.537468474507     -0.608429300633
 Si       -0.729360596411     -1.070601520333     -0.949625731598
 N        -0.829480952561     -2.701469278663     -1.579961106604
 C         0.098966226034     -3.691262093956     -1.122055636431
 C        -0.217139083879     -4.428406688122      0.043663182190
 C         0.596391742130     -5.493872699053      0.422024889345
 C         1.728882924404     -5.859010851765     -0.313413338699
 C         2.051310921386     -5.088395444689     -1.425997040131
 C         1.271544195233     -3.999244777706     -1.836068199865
 C        -1.417063832700     -4.066320267007      0.859824933452
 C         2.595053926583     -7.003189773821      0.125723052947
 C         1.746419488318     -3.165550613199     -2.986834667882
 Si       -0.268115823418      1.563754062720     -0.675335548072
 N         0.093005038244      3.235762258376     -1.039154079952
 C         1.381190330528      3.780853290158     -0.748577949556
 C         2.414195038628      3.711081671757     -1.703842033796
 C         3.645129581410      4.305948505593     -1.415022298074
 C         3.884870618635      4.970292205357     -0.211577603218
 C         2.843969049424      5.031489185525      0.716706475935
 C         1.596987530536      4.452783918863      0.470766315956
 C         2.188539769881      3.042079362298     -3.022816286372
 C         5.230580452357      5.560269337476      0.094289699115
 C         0.493412333142      4.581906281087      1.474635992031
 Si        1.175138835861     -0.110690712180     -0.126412714144
 N         2.147742340988     -0.381578062349      1.313387159186
 C         3.536651179702     -0.662244331945      1.138472713300
 C         3.990119983797     -1.996950107858      1.081955116806
 C         5.347309738267     -2.245349505742      0.873239181810
 C         6.276850967416     -1.212043874058      0.720987861300
 C         5.809363249796      0.099831369635      0.796635371932
 C         4.457743008573      0.395533913809      1.007711719299
 C         3.027340354961     -3.126263143815      1.262380603440
 C         7.728125577810     -1.514076601524      0.482201225876
 C         3.996015322913      1.814774237502      1.108647522745
 Si       -1.076208572573      4.276343944304     -1.907034758031
 C        -1.586950682581      3.461407602965     -3.517640139614
 C        -0.235890383883      5.921407545740     -2.232319724117
 C        -2.572890481805      4.546247236082     -0.812063874943
 Si        1.500618846568     -0.309507478851      2.969781209216
 C         2.933901265290     -0.638199242759      4.136790614346
 C         0.786585331024      1.388695742696      3.318575378277
```

C	0.141567244357	-1.577578266388	3.186996409482
Si	-1.998404012455	-3.281265420725	-2.813263184418
C	-1.265027766340	-3.237431861163	-4.540270798123
C	-2.453802028723	-5.063592658352	-2.422549533468
C	-3.507189020841	-2.183849218725	-2.777770270429
H	4.441964474638	4.247377448102	-2.162755274421
H	3.002544797681	5.551402404481	1.666532786472
H	1.508717037464	3.631967015100	-3.659153151946
H	1.728932816393	2.052945607147	-2.915133655364
H	3.132487787145	2.918879864838	-3.570858799702
H	5.147652339561	6.439560473174	0.749743730425
H	5.756911152273	5.863949828395	-0.822274188160
H	5.874632006622	4.828866226048	0.612821345923
H	0.870054610315	4.974313646534	2.429131657219
H	0.006708137892	3.617143434126	1.666359680464
H	-0.293798104256	5.264880242499	1.116748922563
H	0.073352931235	6.408251021291	-1.294907790176
H	-0.937380106983	6.593312654044	-2.752709662317
H	0.660719149891	5.809631365361	-2.859947238700
H	-2.977745087544	3.586897536776	-0.463368946511
H	-3.374949542346	5.068605836440	-1.356592956564
H	-2.313000720548	5.142918414596	0.075014077682
H	-0.807028319148	3.606278836787	-4.281930521824
H	-2.520858399026	3.905720152007	-3.897932944226
H	-1.735725230861	2.374578181084	-3.408757911149
H	5.687934571990	-3.284525042452	0.827734190718
H	6.516640519880	0.928381675661	0.694228137458
H	2.154466030943	-3.027772626075	0.601721382155
H	2.637308792811	-3.156490899584	2.291955093979
H	3.505661587061	-4.091447191515	1.055616963788
H	8.334083772174	-0.597214752299	0.470576282802
H	7.876303534543	-2.026007819051	-0.482596168170
H	8.132862765667	-2.177236072487	1.262914611897
H	3.525311302015	2.014185079988	2.082941592631
H	3.241589636888	2.060607287005	0.345995534619
H	4.833099804602	2.512939234194	0.984354740832
H	1.532064289942	2.185206650704	3.180066763446
H	0.403073874495	1.439087142649	4.349899375118
H	-0.058422344040	1.581956063300	2.642545412497
H	-0.740046195138	-1.293059355168	2.592569984151
H	-0.179057255809	-1.628956594659	4.238952871370
H	0.463965136945	-2.579966850559	2.870257886706
H	3.378044293778	-1.633023356588	3.984221632088
H	2.578962935264	-0.577775932223	5.178125300218
H	3.734360322604	0.105984544157	4.005319747898
H	0.332704414899	-6.062081260877	1.319312756059
H	2.953150695268	-5.327039125335	-1.998646729672
H	-1.487709588999	-4.692483774748	1.759811576766
H	-2.345911047921	-4.179692717875	0.284072678384
H	-1.378143802140	-3.014967456122	1.182469710440
H	1.992123418864	-7.872602066593	0.428638374937
H	3.212405235190	-6.721256010615	0.995796620101
H	3.277995795623	-7.323183754846	-0.673997127925
H	2.678735114492	-2.650476115752	-2.706423028759
H	1.011723890623	-2.400669756267	-3.254259004988
H	1.957959430568	-3.784339693715	-3.872720175155
H	-1.560116640634	-5.699502222733	-2.330421552151
H	-3.065409011707	-5.458632171102	-3.249912269507
H	-3.040758362924	-5.162031593468	-1.497523568457
H	-3.944437451112	-2.113547213289	-1.771861022331
H	-4.274334446195	-2.597751625850	-3.452132002925
H	-3.240871927852	-1.175989308094	-3.129024191106
H	-0.983586491186	-2.201207577639	-4.778279625135

H	-2.035171898486	-3.557121178866	-5.261626640262
H	-0.397416703207	-3.902244550844	-4.655582358737
H	-2.296139810228	1.610491921504	4.893445437439
H	-3.526755207852	-2.424290239782	4.093826817030
H	-2.220345335130	3.227842289691	3.196786179490
H	-3.639524729233	3.075998311594	2.129633992535
H	-2.044890951857	2.663193645073	1.513720936707
H	-1.532090033462	-1.136956450348	6.211848515854
H	-3.212953536032	-1.657995629288	6.428046607763
H	-2.747255134226	0.027136196848	6.769466525476
H	-5.205924494836	-1.966122586604	1.314846614333
H	-4.003057027156	-3.133275940272	1.892990933673
H	-3.644092697455	-2.078169381147	0.496540822075
H	-5.202199408865	3.468389038661	0.100665535784
H	-6.574842215388	2.807743117886	-0.820960400376
H	-4.938194346195	2.757981839649	-1.512570483354
H	-6.271137788886	-0.066845993101	2.280206330879
H	-7.335667825705	1.187162727367	1.591610399706
H	-5.886138573299	1.646458836034	2.522925505699
H	-5.505248863966	-0.217737803385	-2.001030387313
H	-7.091964256705	0.019763832889	-1.233503965737
H	-6.024271580566	-1.295252154744	-0.685686140777
H	3.253599800050	-0.289584359938	-1.049349842418
H	1.756631780056	-0.129218596209	-3.022868956078
O	2.342098748195	-0.229122403075	-1.371041301239
O	1.357641409576	-0.033398351806	-3.916977668023
H	1.760668271260	-0.733335025522	-4.439327386402
H	-0.415460402861	0.094121468192	-3.960233888090
O	-1.387648844764	0.095836915381	-4.093365618061
H	-1.496709633004	0.442144422574	-4.984639201737
H	-3.081271664235	0.868379056101	-1.853113532145

149
H2O_Int_6, G=-4634.985405

C	4.006457681324	-0.562266368723	1.489696831298
C	3.655291207528	-1.527410126820	0.516962424231
C	4.590223304953	-1.831884821580	-0.499219359770
C	5.861708324091	-1.246826529161	-0.458252709019
C	6.234238017708	-0.325951215035	0.515607763608
C	5.276490094254	0.013375757994	1.472375843300
N	2.373170968497	-2.190355504740	0.596606142135
Si	2.499753139241	-3.832921515493	1.301935899628
C	3.551538603193	-5.053404077380	0.329709568037
C	4.275796731249	-2.689524674689	-1.692018948066
C	7.583332853491	0.330003522205	0.496269384477
C	3.022086879322	-0.115797839868	2.523966951064
C	0.787896031047	-4.603241929831	1.458682825072
C	3.269376181863	-3.682855111198	3.009106129670
Si	1.175529879615	-1.571580507770	-0.643160183398
Si	0.605743188836	0.558661654226	-1.185660844018
N	1.607880871531	1.814666843008	-1.917651043549
C	1.409424408198	3.146533239800	-1.448052126653
C	2.214820127732	3.662818411337	-0.415131743232
C	1.999484599742	4.974001253236	0.023604538401
C	1.020594021743	5.790649936924	-0.540502138401
C	0.235480681277	5.256351549941	-1.566086322951
C	0.396337385275	3.947746338201	-2.020398079686
C	3.290162429614	2.826388697180	0.211357660153
C	0.781312262510	7.187103104400	-0.043689678337
C	-0.527314340752	3.390586987426	-3.062336247490
Si	-1.127371586614	-1.426833749233	-0.411145283102
N	-2.313315544824	-2.315595293008	-1.379206412962
C	-3.619081867163	-1.734822980511	-1.327929227946

C	-3.968453248426	-0.683689577956	-2.211580891188
C	-5.202946798936	-0.047768579029	-2.059469123826
C	-6.123982732254	-0.431105205404	-1.078686693956
C	-5.791508651611	-1.518120341978	-0.274009680118
C	-4.565941294138	-2.186216213206	-0.387136951094
C	-3.048691150016	-0.267706964795	-3.322178207577
C	-7.413036912316	0.314616076451	-0.899310086727
C	-4.306543230272	-3.379346824322	0.486073376107
Si	-1.318156992392	0.848059491191	-0.009262280658
N	-1.833044244973	1.537847881467	1.534870747149
C	-1.370442322320	1.004807220518	2.759240946418
C	-2.146665670317	0.057375358531	3.461725979634
C	-1.672853888102	-0.450539822751	4.674444940981
C	-0.446851137827	-0.054422921636	5.216063448442
C	0.296680652478	0.901966756201	4.515699223153
C	-0.148992478853	1.448744310726	3.306192820960
C	-3.475636434400	-0.377528077081	2.924358363860
C	0.077070398688	-0.669528906449	6.480734722448
C	0.626393530206	2.525024135342	2.613086238000
Si	-1.899293168556	-3.495499684070	-2.640807615891
C	-0.807702145150	-2.786725501610	-3.997527457010
C	-3.524335247962	-4.117560238939	-3.342899542167
C	-0.955719748899	-4.927932185113	-1.867204921000
Si	-2.972410927869	2.912936722104	1.577094033648
C	-3.148543715263	3.367385438510	3.398398691889
C	-2.300378460700	4.399734338567	0.657157784278
C	-4.686716009393	2.482094247869	0.907344792485
Si	2.734580300317	1.521810180641	-3.253458336742
C	2.100076636783	0.050911580044	-4.234884552067
C	2.830380611460	3.054973208628	-4.335526736547
C	4.474496541859	1.153137001342	-2.646590376070
H	-5.454892292551	0.773770864479	-2.737508704054
H	-6.511878135580	-1.869821787327	0.471126089807
H	-3.119345798300	-0.976663694204	-4.162946093903
H	-1.998772923004	-0.239320121093	-2.996900423323
H	-3.317680814946	0.725710434157	-3.705200089301
H	-8.149842582485	-0.279638378050	-0.340401405222
H	-7.857773706586	0.591807118630	-1.866756710928
H	-7.244622669002	1.249207727057	-0.338287920772
H	-5.233962059655	-3.946723233813	0.649657668321
H	-3.925546018720	-3.078189276142	1.474374492150
H	-3.566333213430	-4.050146802679	0.028177752350
H	-4.145182755922	-4.587355185172	-2.564525314680
H	-3.334412546426	-4.865695747715	-4.128843922601
H	-4.111850091726	-3.299379894530	-3.786542976410
H	0.050717685095	-4.611960436068	-1.553300231387
H	-0.826620953265	-5.728506810601	-2.613593364609
H	-1.478402412198	-5.360925179839	-1.000142686491
H	-1.386390238221	-2.258685427041	-4.768686543954
H	-0.241127090231	-3.593116640969	-4.489988670735
H	-0.084666343040	-2.073477402681	-3.572794610047
H	-2.276968803083	-1.191105951734	5.207295167894
H	1.250889798819	1.244049176360	4.927512681862
H	-3.428636071967	-0.570193319981	1.844004043903
H	-4.236913414952	0.404979343917	3.074111716283
H	-3.829487550522	-1.285940395418	3.431502963639
H	-0.738567168282	-0.924841437101	7.172872980569
H	0.770518671251	0.006137534627	7.001811824976
H	0.625875954190	-1.602120274359	6.265093783099
H	0.782814011296	2.293222649265	1.549649119613
H	1.602783104404	2.687300787257	3.088359159915
H	0.074429365370	3.478167474694	2.635080623046
H	-1.292888303253	4.673622982750	1.004430886543

H	-2.965301335500	5.267387199703	0.799928809045
H	-2.234165813604	4.183411895730	-0.417678633832
H	-4.776749591664	2.772381375695	-0.149869167904
H	-5.465622738619	3.016319973857	1.475241216191
H	-4.894005755244	1.403905277028	0.979285498422
H	-3.581919091933	2.553453195606	3.999202458856
H	-3.809454287109	4.244666782123	3.489297789168
H	-2.177844133193	3.627505364845	3.847887753981
H	2.621991862602	5.365688147439	0.834370809707
H	-0.547698510906	5.874591548933	-2.015669255143
H	3.508025178910	3.164067901916	1.234619961969
H	4.231201563900	2.882561690179	-0.358381321737
H	3.012051732320	1.764127473383	0.239422565729
H	1.569139813900	7.509888482992	0.651783024702
H	-0.183046338280	7.256913215113	0.486974753033
H	0.743906022555	7.909515798907	-0.873922907338
H	-1.078258167339	4.196529341770	-3.567739259409
H	-1.264446639359	2.714489564402	-2.597136246966
H	0.010615585967	2.804401565385	-3.819274225172
H	1.893404638012	3.260704003449	-4.872912755024
H	3.630598981557	2.923402317529	-5.081736554897
H	3.074332451926	3.942446061448	-3.730943495785
H	4.982352427547	2.071806348932	-2.315230355689
H	5.078900537067	0.711826139795	-3.456212839925
H	4.468962632973	0.450120750979	-1.801731741349
H	2.118684782993	-0.875205383687	-3.640105700454
H	2.728246262127	-0.106988843788	-5.126598816247
H	1.062856916122	0.212734799003	-4.566991018326
H	6.574812211729	-1.506338145033	-1.246886104731
H	5.521285476376	0.758762996705	2.234979593031
H	5.109629378104	-3.368763305770	-1.922905723384
H	4.123948959263	-2.047916185761	-2.575851127066
H	3.366368805810	-3.281307617941	-1.566926861869
H	7.924196968833	0.585620583907	1.510236897714
H	7.553387225226	1.267120639124	-0.085046208496
H	8.340984541084	-0.317680412762	0.031959012565
H	2.080484905325	0.197984699488	2.048688255899
H	3.412759692407	0.742774650841	3.086153649454
H	2.791932862580	-0.910296399538	3.248748656471
H	0.240891640531	-4.184634898833	2.314089412214
H	0.911993142720	-5.683998043809	1.631131331943
H	0.197742266217	-4.490718933602	0.538192478779
H	4.223834236788	-3.135377393283	2.969908105029
H	3.471596353371	-4.685960228555	3.417119461647
H	2.591374555291	-3.163361463398	3.702230072928
H	3.130484565699	-5.297152594562	-0.656837818893
H	3.594219444795	-5.987194509834	0.914805619919
H	4.583241287236	-4.698805615092	0.192788825351
H	-3.260869748561	1.169867434667	-1.131863047842
O	-2.438146372251	1.659062107585	-0.996065761352
H	1.464463515396	-2.368247524915	-1.878275270998
H	-1.603833366394	-3.265710189168	1.164012783992
H	-0.767377772329	-2.087494317000	1.933892576431
O	-1.537268274247	-2.304959617947	1.252373620631
H	0.360321752551	-0.961437162375	3.174605328154
H	1.151871566986	-1.719106287275	2.046434669141
O	0.410324350165	-1.805747845743	2.695795345362

149
H2O_TS_3, G=-4634.983928

C	3.941920149254	-0.973376997072	1.678451402266
C	3.433296268056	-1.774617771031	0.626952077978
C	4.289321224016	-2.062466914053	-0.462088088579

C	5.608732531697	-1.589290508985	-0.445648585158
C	6.124887843996	-0.822017255387	0.591029679090
C	5.261292339518	-0.525339937081	1.644845275385
N	2.083187387353	-2.309134267714	0.713497367104
Si	2.098683482969	-4.032936880312	1.267017209737
C	2.732034152549	-5.289893012558	0.021829996269
C	3.880294567537	-2.802331601376	-1.704583402151
C	7.527205057683	-0.291192453965	0.557774942286
C	3.083438663808	-0.525700218546	2.818322428379
C	0.349088095034	-4.561413471985	1.715585683504
C	3.181264248291	-4.142955030422	2.796002813610
Si	0.928353846004	-1.654846677482	-0.626339760394
Si	0.594896757990	0.470873307162	-1.325281141659
N	1.751982620490	1.593716023821	-2.060943945830
C	1.840776539062	2.902879611238	-1.500313749856
C	2.777912226867	3.187074312309	-0.488516662614
C	2.843175277157	4.484277458022	0.032811331980
C	2.011062206525	5.506718663652	-0.419177298544
C	1.076309341602	5.196207699282	-1.411426259671
C	0.961377216005	3.914586085352	-1.948907429500
C	3.673921489713	2.115921564728	0.055689500920
C	2.088877589960	6.893954796353	0.150499507727
C	-0.116626959297	3.606884602988	-2.945110686398
Si	-1.355047929620	-1.360216745964	-0.250969053096
N	-2.612816794108	-2.157233762775	-1.211045581616
C	-3.851678929075	-1.446023422217	-1.189158723827
C	-4.107906049963	-0.425152000039	-2.137296676687
C	-5.268035737338	0.343167911777	-2.013471670857
C	-6.203979763411	0.123835268187	-0.996806968955
C	-5.968785528225	-0.934921008794	-0.122924666095
C	-4.820196764182	-1.731502115553	-0.206255141391
C	-3.165991588819	-0.177565723779	-3.278738674091
C	-7.405173144294	1.010624658610	-0.851985164549
C	-4.657588252289	-2.878360942905	0.748758566516
Si	-1.272329259965	0.967552262460	-0.120415280386
N	-1.664274460112	1.777200039008	1.408375626356
C	-1.242794559364	1.234661885871	2.642099257868
C	-2.109705059035	0.412836649016	3.395869070136
C	-1.673242427618	-0.104705836384	4.618824141121
C	-0.402400081828	0.168050629723	5.130031129791
C	0.433699806102	1.003290706034	4.381797167764
C	0.035849567680	1.546383744600	3.152795627038
C	-3.492089992186	0.121753001973	2.901258040961
C	0.069375203788	-0.450518614019	6.413109132086
C	0.926879976687	2.481945807391	2.397372073395
Si	-2.316465508019	-3.443849334951	-2.395684157151
C	-1.180657015959	-2.928446594092	-3.803737920185
C	-3.996884029151	-3.963856446041	-3.049998740265
C	-1.497108027064	-4.899139638577	-1.527828651873
Si	-2.648171121095	3.266148476137	1.418086212863
C	-2.723709934962	3.810054923754	3.222286407946
C	-1.848129216924	4.640255932732	0.427064006712
C	-4.416487894372	3.002903190877	0.802581182611
Si	2.730811430820	1.188305819507	-3.479362706668
C	1.939747950615	-0.293580206055	-4.320589209739
C	2.804560714944	2.640049014861	-4.673643401570
C	4.511740291620	0.783910577126	-3.022924286731
H	-5.447559318234	1.139902890294	-2.742321146268
H	-6.705385883607	-1.157967041251	0.655224004946
H	-3.307718857274	-0.937219412640	-4.064462107645
H	-2.114526740396	-0.219864887499	-2.958510725535
H	-3.347709452697	0.805603285274	-3.732793270041
H	-8.187276086211	0.536188697985	-0.242329680070

H	-7.839653602782	1.265950312854	-1.830081469746
H	-7.129496101955	1.958792177047	-0.360396725033
H	-5.634266023079	-3.316396418239	0.999330353330
H	-4.194196408995	-2.550270951607	1.692837795562
H	-4.024987780435	-3.665810633303	0.315275204461
H	-4.648895539239	-4.326605889754	-2.240397217628
H	-3.882863861117	-4.773259678801	-3.788472065466
H	-4.513824264228	-3.125389092430	-3.541288048642
H	-0.472458191621	-4.644480348153	-1.217841048436
H	-1.425614005162	-5.751718460519	-2.222564411891
H	-2.060174745602	-5.233891577692	-0.642731823313
H	-1.726054496656	-2.434240686012	-4.620444160763
H	-0.668347176373	-3.809503661554	-4.222151111519
H	-0.413283778707	-2.227585950413	-3.440191219240
H	-2.350407142229	-0.748708813417	5.188132863732
H	1.426283196280	1.252164951000	4.768688213155
H	-3.490700803968	-0.118822554273	1.829897685642
H	-4.149764332535	0.996219220033	3.030856670444
H	-3.941037258957	-0.715851610474	3.452801194189
H	-0.762376650140	-0.607921824193	7.115078499530
H	0.824023946544	0.176008004825	6.910247302726
H	0.531007144126	-1.435402885770	6.227561968982
H	1.059150374695	2.159326005752	1.354229021435
H	1.915063837038	2.568734741422	2.868890898511
H	0.484918796280	3.489285114596	2.345416289071
H	-0.813935994825	4.829104388350	0.751311200879
H	-2.424375296546	5.572870416072	0.543592742251
H	-1.823877269558	4.377823267454	-0.639374035268
H	-4.500750638898	3.270169850254	-0.261191527391
H	-5.118463754235	3.635422015899	1.369889682672
H	-4.737680997875	1.956382938671	0.913220082702
H	-3.225375362555	3.074041414822	3.868721919238
H	-3.281705128747	4.757932657662	3.291748045885
H	-1.717275590353	3.979623666471	3.635328232437
H	3.567839915384	4.694873072801	0.825833559585
H	0.393406447174	5.974925565351	-1.764616753951
H	3.992345433770	2.352766742998	1.080815474726
H	4.586067349728	1.991672138412	-0.547756652487
H	3.173231370760	1.137453449907	0.063004419070
H	2.854447750906	6.965236993926	0.936172923416
H	1.125164605525	7.199356844554	0.589515348004
H	2.335552522291	7.634303691879	-0.627564719530
H	-0.606360921213	4.529830629783	-3.286507362388
H	-0.890478647968	2.957916085251	-2.501781355312
H	0.274302216059	3.075283264365	-3.822316160222
H	1.870238817777	2.773472647565	-5.238494203197
H	3.616991027525	2.472952096337	-5.399189312435
H	3.018002183104	3.579178826624	-4.139246990209
H	5.060284229847	1.700117966798	-2.753863787361
H	5.028579717383	0.328102894175	-3.883589039271
H	4.574900686607	0.091896989943	-2.171354879647
H	1.926518380647	-1.186772063459	-3.678012921389
H	2.499089276191	-0.541472546191	-5.237542410626
H	0.897659318458	-0.074552367568	-4.600853771043
H	6.250842570818	-1.832930292773	-1.297475195168
H	5.620310131298	0.091982745186	2.473598592097
H	4.690168699365	-3.466270844181	-2.041034909552
H	3.689279801740	-2.087188158764	-2.520869989325
H	2.972992488704	-3.394540914980	-1.584563173779
H	7.989391653972	-0.306644628816	1.556080440081
H	7.540685305317	0.755248109384	0.209272000875
H	8.163372898580	-0.872568772158	-0.124628378760
H	2.257097185397	0.095822701323	2.444921833570

H	3.662184848429	0.093098168128	3.516759335241
H	2.655882444338	-1.360074744246	3.390295878229
H	0.005104619578	-4.104959659175	2.653653955907
H	0.349266611337	-5.654833670365	1.848778410170
H	-0.366707816109	-4.333606162521	0.914377081147
H	4.195626127565	-3.762810094136	2.602282430711
H	3.265153428034	-5.196916632220	3.106050144411
H	2.754616419248	-3.579385545849	3.639130727488
H	2.142890035499	-5.307744162109	-0.907441362780
H	2.641367441576	-6.283444050376	0.491515412976
H	3.789619752838	-5.136741749674	-0.234228409269
H	-3.207584474103	1.448724090287	-1.202979383635
O	-2.333439605147	1.850976805908	-1.112057891470
H	1.126542042854	-2.638309106562	-1.735831951612
H	-2.056752346731	-2.919078949990	1.379736076734
H	-0.636827570017	-1.848770577087	2.113834212234
O	-1.704744328708	-2.020615114741	1.391834750781
H	0.366221533856	-0.745089863056	2.935045131576
H	1.110660904675	-1.799321649520	1.891643605881
O	0.345980465585	-1.665892616084	2.607190004321

149
H2O_Int_7, G=-4635.012237

C	4.170213085255	-0.623763669236	0.968030299651
C	3.639108850113	-1.711483409655	0.238855797786
C	4.356692274513	-2.221794291496	-0.859639524188
C	5.592921632116	-1.651356162675	-1.185015888326
C	6.146703873154	-0.594320325400	-0.472369724587
C	5.410042651578	-0.100845076362	0.604206501386
N	2.358788839569	-2.277543383835	0.640312205202
Si	2.381848338246	-4.057101674122	1.212510592754
C	1.660421855113	-5.229989906033	-0.057022809968
C	3.897552321464	-3.368900030710	-1.712657294648
C	7.456187289454	0.024114196348	-0.863586321928
C	3.428352726566	0.017580676001	2.100068231135
C	1.308521903470	-4.211276373470	2.730069012838
C	4.155297348926	-4.446985682469	1.667564294662
Si	0.840348682957	-1.606158885954	-0.588501062461
Si	0.357139012690	0.492174147795	-1.153015654569
N	1.256785393418	1.637934032844	-2.157651129293
C	1.360670769375	2.996805008938	-1.736647521325
C	2.513620087582	3.438444509613	-1.062010894273
C	2.593342368574	4.769743971796	-0.645898779255
C	1.559947287998	5.678191860590	-0.877365593565
C	0.431356231281	5.221942854061	-1.563499179161
C	0.311658367876	3.900920055258	-1.999897216005
C	3.628352072015	2.480192610687	-0.781237393957
C	1.638157971075	7.092727809782	-0.379577319741
C	-0.911476773669	3.450263598140	-2.739479377858
Si	-1.330402707336	-1.594594658497	0.260116320903
N	-2.635739893847	-2.409690239595	-0.630909462695
C	-3.659489998990	-1.566081948852	-1.152934613793
C	-3.592411885410	-1.063758351023	-2.469672257945
C	-4.587323592090	-0.182392705365	-2.913144827152
C	-5.659020521794	0.199953727892	-2.104585003512
C	-5.729979721912	-0.346348681635	-0.821347076698
C	-4.752119761784	-1.213124591595	-0.333215374693
C	-2.485674408634	-1.465875426181	-3.395098417809
C	-6.679512945819	1.192842329863	-2.577574779551
C	-4.851298375909	-1.742268426009	1.064656014051
Si	-1.443603553523	0.770985647360	0.228956291449
N	-1.640151619379	1.724332915682	1.702528799261
C	-0.824890100792	1.490305127909	2.840562250486

C	-1.292761919819	0.682788696010	3.901188964959
C	-0.498151846574	0.499938937962	5.035337952376
C	0.762779651570	1.085341799494	5.156213638312
C	1.205423985401	1.892403003164	4.106022316750
C	0.435970088667	2.114394487587	2.958957863852
C	-2.642313614388	0.040577404861	3.824459816486
C	1.632893446532	0.810065738757	6.346758348914
C	0.922155909826	3.041536639793	1.891642627335
Si	-2.676459637711	-4.142167872729	-1.005081406302
C	-1.400795606716	-4.636022015735	-2.301396939785
C	-4.395659371290	-4.541021424832	-1.642296706995
C	-2.327664729482	-5.131564010327	0.556988929404
Si	-2.856156943803	3.033920940245	1.817003022087
C	-2.587587568570	3.845299940416	3.496807338030
C	-2.603451160970	4.341095892571	0.494527992567
C	-4.618681341104	2.382723083702	1.734010792806
Si	1.922787261970	1.161334941914	-3.732687746669
C	0.574212789883	0.411019722082	-4.806806729435
C	2.625819324475	2.703089165113	-4.537446708714
C	3.251096789913	-0.148437462502	-3.519106963335
H	-4.513665252461	0.218728338983	-3.928485653559
H	-6.565399118379	-0.074629966990	-0.169357490705
H	-2.673047162323	-2.464039305894	-3.822923369561
H	-1.523140604675	-1.499031855321	-2.864602115066
H	-2.397010338216	-0.760116662031	-4.232025895871
H	-7.692113566493	0.920415760053	-2.244463956949
H	-6.688765210878	1.273385597254	-3.673850561401
H	-6.463294163015	2.195906911121	-2.173183492051
H	-5.839762013131	-1.537162147834	1.497591927199
H	-4.107236170256	-1.259906310439	1.718110962590
H	-4.666220802269	-2.826074260079	1.103276345644
H	-5.172053684512	-4.293991889806	-0.902250894588
H	-4.471541498511	-5.615294675229	-1.874724149779
H	-4.621347831253	-3.976308504222	-2.559850453972
H	-1.336131823852	-4.906921198051	0.974967319924
H	-2.368373410157	-6.209474591180	0.331324533627
H	-3.075069618537	-4.922748973765	1.338465890315
H	-1.755873167354	-4.416884345957	-3.318881478820
H	-1.186219651501	-5.715107811118	-2.242999094580
H	-0.456307251667	-4.090038047054	-2.158914313955
H	-0.870412158959	-0.140264948025	5.840780762109
H	2.184478239187	2.375614375100	4.179029272723
H	-2.887323111228	-0.209539821135	2.785551469088
H	-3.431394147014	0.725542004421	4.175897694567
H	-2.690394882778	-0.866138350636	4.444512123712
H	1.040513436774	0.721801155080	7.269497786058
H	2.380894757111	1.602516187916	6.493531636446
H	2.177960638638	-0.140256363576	6.216829535245
H	0.808274716551	2.603873119769	0.890817441216
H	1.978330876938	3.304339867011	2.036553719443
H	0.341485815056	3.978262846889	1.882655031867
H	-1.550099056992	4.655892214479	0.440653543788
H	-3.218976558152	5.228029019007	0.716651936762
H	-2.891091127038	3.946914268534	-0.489290866198
H	-4.759797664312	1.829594859628	0.794594023769
H	-5.333241029284	3.221659118052	1.761148199232
H	-4.851504389865	1.713648966916	2.576082218333
H	-2.735688666323	3.146393194446	4.333685472998
H	-3.305244232930	4.673497860912	3.614616279152
H	-1.572314777011	4.260700794107	3.589949117100
H	3.490218073755	5.102297123286	-0.113727082706
H	-0.388554367625	5.917196841348	-1.768018847475
H	4.319332931625	2.880114155293	-0.025820468110

```
 H          4.217954287664      2.262546769063     -1.687318190900
 H          3.241944647014      1.512697517127     -0.431917493296
 H          2.675296441559      7.390040876046     -0.167523303545
 H          1.062424953587      7.216486958673      0.553322109716
 H          1.222303823624      7.801981606020     -1.111058700451
 H         -1.591264442753      4.293789421145     -2.922074592623
 H         -1.467400388699      2.688179141061     -2.170857401689
 H         -0.649256983704      2.999600849652     -3.708665857400
 H          1.850407575403      3.473976686932     -4.664246012115
 H          3.033480944388      2.457035190121     -5.530875642427
 H          3.432928310916      3.144311507428     -3.933343684440
 H          4.064646319935      0.185015116491     -2.859005560306
 H          3.682113352242     -0.429191185771     -4.493883306581
 H          2.815232039312     -1.054221246455     -3.071039633459
 H          0.134433962289     -0.463832003764     -4.303915807048
 H          0.987035752058      0.077785988519     -5.772685993937
 H         -0.237987017766      1.126372491558     -5.006596304660
 H          6.132320192675     -2.054934751182     -2.046753953141
 H          5.802853832748      0.740862570120      1.181540847316
 H          4.298424079702     -4.328452318237     -1.347581583534
 H          4.260875788468     -3.238827937368     -2.741300428793
 H          2.807459166400     -3.452258688205     -1.757638692480
 H          8.105845452445      0.177705392667      0.011521715966
 H          7.301047838624      1.011979449401     -1.328252367299
 H          7.998718621559     -0.601273422118     -1.586360978559
 H          2.465040036406      0.424309907618      1.754155883281
 H          4.010854053765      0.850373248257      2.514414213833
 H          3.205187018733     -0.683617626178      2.917089826894
 H          1.641549481328     -3.566850805392      3.553776199689
 H          1.352271781155     -5.264397996960      3.053315476033
 H          0.261706204373     -3.958755400986      2.505406706542
 H          4.854843098624     -4.404826861949      0.822070341787
 H          4.196248221764     -5.459077284345      2.101787027845
 H          4.510301309359     -3.737423507001      2.431174623016
 H          0.688066576202     -4.866998460651     -0.418914489302
 H          1.476029749512     -6.185061253888      0.461594778416
 H          2.297353786724     -5.434621742976     -0.925463403374
 H         -3.067317816552      0.909791433794     -1.350190706741
 O         -2.755166230274      1.438797445642     -0.603836542802
 H          1.140506206758     -2.569578726123     -1.688186861900
 H         -2.203381203527     -2.513716633223      2.166019920108
 H          0.054597568760     -1.722236683405      2.699660808056
 O         -1.320598975589     -2.370537534611      1.806340049508
 H          0.790596271734     -0.408747746688      2.984607686412
 H          2.002893441931     -1.786060328672      1.495504926573
 O          0.944253087250     -1.357655011314      2.885837673222

114
H2O_Int_8, G=-3822.205703
 Si         0.550029100860     -1.453566746232     -2.608278043691
 Si         1.952937401443     -0.043980212713     -1.350079965382
 Si         0.265957016611      0.477491025375      0.291267895794
 Si        -1.062253528005     -1.010959098828     -1.014143178216
 Si         4.909167020907     -0.780272418491     -1.655336971814
 Si        -0.312571999313      2.762927693392      2.347768964180
 Si        -2.451493999638     -3.740721654574     -1.015093598818
 O          2.417555707512      1.197550423872     -2.413724709050
 H          1.898453658012      1.137960720818     -3.224733714662
 O          0.584065258678     -0.219055061885      1.786068548076
 O         -1.936169159154      0.256012787120     -2.257899246694
 N          3.487939861078     -0.508356500087     -0.619883866423
 N         -0.322652087809      2.079998050872      0.704829639931
 N         -2.392725933943     -2.019656065001     -0.532646673254
```

C	3.560951270854	-0.723540663005	0.779839265510
C	4.017226732235	0.300554653518	1.635670817889
C	4.050152615381	0.075410961976	3.015333905912
H	4.395431880583	0.881617674023	3.669515537523
C	3.638463635721	-1.131897740977	3.581497964046
C	3.202534218971	-2.140445579999	2.717254388804
H	2.878870573328	-3.099534567056	3.132420674922
C	3.160678618299	-1.962522934994	1.330447488444
C	4.465577246315	1.616558671797	1.077862656331
H	4.407432418967	2.408480877144	1.836859354734
H	3.866449119058	1.906172327626	0.203965297471
H	5.511389939820	1.562301122335	0.733991835945
C	3.622706229301	-1.326461257017	5.069540075054
H	2.653773979335	-1.016282482688	5.495793066817
H	4.402592081665	-0.728426568812	5.562812436555
H	3.776020817468	-2.381126925162	5.340692523795
C	2.676991049200	-3.063715723900	0.436881438763
H	2.430878619249	-3.965405560819	1.013822226678
H	3.431874790436	-3.326317255557	-0.319904724167
H	1.781195346455	-2.758854061778	-0.126364238719
C	4.346932589844	-1.742772360238	-3.167553893123
H	3.867880454690	-2.693490764087	-2.888278414921
H	5.202221886220	-1.963052008580	-3.826242804423
H	3.617762359937	-1.158581027768	-3.751335604109
C	6.148822971537	-1.762906499502	-0.642901429725
H	6.443859647430	-1.209668606491	0.262443197703
H	7.057483082420	-1.949031889576	-1.237314511599
H	5.744448230993	-2.734702537371	-0.321940577557
C	5.693949308002	0.824258725626	-2.236801624709
H	4.908677995661	1.473671411491	-2.651344821192
H	6.440895645543	0.623939398655	-3.022078630568
H	6.196819658741	1.363919265926	-1.420873340777
C	-0.802850622116	2.804350669752	-0.415919547635
C	-2.183740709367	2.785426705238	-0.728876960181
C	-2.623998749804	3.388608335032	-1.913815070398
H	-3.692118755116	3.360518576305	-2.150765721260
C	-1.740853124271	4.028143215019	-2.791478625304
C	-0.395597271250	4.089809900419	-2.427171341883
H	0.309086548870	4.617113746000	-3.077321325788
C	0.090580961276	3.507843065332	-1.250813217857
C	-3.166093089459	2.152317684754	0.209795471331
H	-3.307367323900	2.781792676417	1.102693173557
H	-2.824361784809	1.173202304425	0.573989152289
H	-4.147504204220	2.020334662286	-0.262773804341
C	-2.228264071771	4.619040708316	-4.082284185081
H	-1.566549083356	5.424443795826	-4.431365291519
H	-3.243585564704	5.029359939060	-3.980104716708
H	-2.264323081670	3.856951717261	-4.878971978534
C	1.521615760226	3.702078822862	-0.853756534001
H	2.174299052078	3.797937675545	-1.729748646666
H	1.888268527733	2.867668100321	-0.246175075522
H	1.616614917148	4.616966634409	-0.243758782845
C	-1.386612340167	1.749847770966	3.504666748192
H	-2.421830385694	1.670034263201	3.140568093756
H	-1.405974572543	2.199454685580	4.510553493744
H	-0.974943905077	0.732861501917	3.584270523200
C	1.441225681487	2.818431181130	3.010167907204
H	1.874760948406	1.807672432347	3.025565165216
H	1.458165907379	3.216508185707	4.037545317591
H	2.079291852120	3.460248052977	2.383559002109
C	-0.968558259074	4.517807235476	2.186224119766
H	-0.344815601327	5.113414587850	1.501675651231
H	-0.954634319651	5.011246887338	3.171223801741

```
 H        -2.001505256929      4.548610047071      1.808357467122
 C        -3.424685179727     -1.467780756795      0.289635494839
 C        -3.256543351105     -1.416666634614      1.688908934093
 C        -4.245280907664     -0.809574904958      2.466909675861
 H        -4.100329089293     -0.760685599732      3.550273787979
 C        -5.410415147430     -0.280793926850      1.907579288291
 C        -5.583846962036     -0.406608141091      0.528806076375
 H        -6.503731873571     -0.031703561210      0.069312437161
 C        -4.616223832593     -0.991381634454     -0.293062202576
 C        -2.056360414156     -2.031663807705      2.341198251475
 H        -2.139634918983     -1.974551395802      3.435222415537
 H        -1.118368241761     -1.533096024183      2.054359392569
 H        -1.957581387246     -3.091961881607      2.061550652100
 C        -6.432722273678      0.411981811794      2.761020481647
 H        -6.503210696854     -0.045745454834      3.758795175513
 H        -7.430681144752      0.384272870481      2.300063275025
 H        -6.169114933421      1.473020163394      2.909170033573
 C        -4.873772237959     -1.128188016393     -1.764771566064
 H        -5.753158908359     -0.540650743941     -2.063371260935
 H        -5.074604883841     -2.177715156872     -2.035308183823
 H        -4.012177329201     -0.804089690576     -2.361907199814
 C        -3.965665083722     -4.481287723736     -0.192409187269
 H        -3.906920010000     -4.390006470795      0.903026200539
 H        -4.036371818018     -5.551815398017     -0.442630229821
 H        -4.896193262319     -3.991877300274     -0.515918949892
 C        -2.552063070461     -3.883195758882     -2.883508968958
 H        -3.487252541127     -3.455294800829     -3.274088019703
 H        -2.500226544808     -4.939177874027     -3.193694831789
 H        -1.711919927931     -3.351726202673     -3.359198999320
 C        -0.908422983966     -4.630682403743     -0.421342854746
 H        -0.007874894247     -4.208378241924     -0.891509763904
 H        -0.959336199992     -5.699036247416     -0.687093500509
 H        -0.791686832463     -4.553610999736      0.670275677777
 H         1.009020192632     -2.885985754584     -2.606288284540
 H        -1.323526603304      0.156977535585     -3.028070685664
 H         1.437862279203     -0.668498441745      1.875748029928
 H        -1.979743284007      1.206281931923     -1.993398746923

114
H2O_TS_4, G=-3822.200721
 Si        0.545520017523     -1.368169066997     -2.612188066870
 Si        1.952936742572      0.003075938677     -1.307574393206
 Si        0.238581258166      0.424296731920      0.342400579153
 Si       -1.145312572152     -0.904250187305     -1.062314164717
 Si        4.890802957430     -0.810667021709     -1.622121486595
 Si       -0.335462788455      2.640297551708      2.469649357815
 Si       -2.586217200068     -3.600099512523     -1.204287125874
 O         2.462756887337      1.285460607393     -2.290496104926
 H         1.881758215650      1.395518358326     -3.052153778260
 O         0.506423811789     -0.366233182274      1.797714306585
 O        -1.714247895265      0.273592979803     -2.358497585402
 N         3.457889985781     -0.555911912431     -0.594662266123
 N        -0.271600194140      2.036375145275      0.799478384083
 N        -2.485307807380     -1.917018477568     -0.623249850159
 C         3.505456676205     -0.847822847264      0.793517989211
 C         3.990582243993      0.105433267087      1.712219318181
 C         3.992861388962     -0.201014577212      3.077258616399
 H         4.358824603394      0.551939877383      3.781685523098
 C         3.528080557762     -1.421654779355      3.567109915938
 C         3.063921170011     -2.360192876480      2.640095431433
 H         2.695311974612     -3.327340909861      2.994590987999
 C         3.045901289624     -2.098584844558      1.266825476245
 C         4.504615194169      1.433078534238      1.245295236987
```

H	4.371034985188	2.201900782680	2.018817770244
H	4.003658934871	1.755750953344	0.323696135174
H	5.582177995413	1.378216131984	1.018170931369
C	3.485135549108	-1.704166359350	5.040337275036
H	2.499929487072	-1.442152559399	5.461345994095
H	4.240063139954	-1.119596438602	5.585354382809
H	3.656985142354	-2.769636245710	5.252237275110
C	2.506741453019	-3.116359725477	0.308314481829
H	2.308980948447	-4.072407161173	0.811563608060
H	3.198562824479	-3.290821060461	-0.528645239037
H	1.562429048133	-2.773916530975	-0.145513998133
C	4.323524433838	-1.631429452600	-3.213375984930
H	3.806204561506	-2.583604649422	-3.021098690261
H	5.185175814446	-1.830635090283	-3.870467809327
H	3.630578247711	-0.974125374242	-3.762245280918
C	6.064213257105	-1.917964493657	-0.662421824582
H	6.366363894880	-1.444749528265	0.284865806391
H	6.974806552361	-2.105157507791	-1.253462010392
H	5.608029792398	-2.889355067776	-0.418672541788
C	5.745445034480	0.801145373899	-2.066356587026
H	4.995898282093	1.505821874080	-2.456525189634
H	6.507347205558	0.628978729382	-2.843864090692
H	6.242412424388	1.264288391734	-1.201607893594
C	-0.637268230661	2.828591630674	-0.322303239184
C	-1.983346824228	2.862192341201	-0.755216777445
C	-2.299453343090	3.528052252969	-1.944710534715
H	-3.341706090510	3.539284313391	-2.278397831518
C	-1.326155457855	4.181894659949	-2.708697160198
C	-0.018287388037	4.193650381945	-2.223206642009
H	0.753822344630	4.731796738418	-2.781424394244
C	0.344189235544	3.544676382548	-1.037706028947
C	-3.062844229772	2.226534490258	0.067205320615
H	-3.285610569478	2.842108442151	0.953858977200
H	-2.774260072103	1.236327358075	0.445698821570
H	-3.993903543270	2.118813982659	-0.503412548922
C	-1.682911987113	4.840540388016	-4.009319502143
H	-0.937774881284	5.595954662166	-4.296331030311
H	-2.665958671498	5.331526243946	-3.956258317282
H	-1.733841108412	4.100999271163	-4.826008324829
C	1.737249078384	3.671880437296	-0.503124370854
H	2.461721784393	3.857673962678	-1.305295885845
H	2.044393521575	2.769060132321	0.036500800400
H	1.788166241949	4.509111097435	0.214103067130
C	-1.509662254514	1.606088994178	3.503433675536
H	-2.520110022491	1.579382739472	3.068266438988
H	-1.582996365753	2.005034010825	4.528018768614
H	-1.136930574534	0.572425478319	3.557783016005
C	1.377664010519	2.599080860761	3.235873933162
H	1.781943113266	1.576162455839	3.210223589869
H	1.345198065166	2.930121257184	4.286391542000
H	2.069817352781	3.258758752070	2.690382914446
C	-0.931000213571	4.419421604703	2.358573927663
H	-0.253441405562	5.030495580541	1.742442397909
H	-0.966927694039	4.864795956460	3.365618782355
H	-1.937706928552	4.493186777014	1.920380778273
C	-3.512608223260	-1.371253266224	0.209397495701
C	-3.380190624470	-1.423610732886	1.611932752317
C	-4.362777030056	-0.827147263929	2.405664922799
H	-4.246740094126	-0.859466817704	3.493159909471
C	-5.485179070334	-0.204525817666	1.855181528738
C	-5.620460629399	-0.217036429493	0.466349424549
H	-6.504007645570	0.240870415031	0.010907884029
C	-4.656933155860	-0.788426420394	-0.369219131018

C	-2.221146088537	-2.129229097519	2.247576286352
H	-2.313410809160	-2.115924535964	3.342348072014
H	-1.256381137109	-1.667741891399	1.988636948982
H	-2.174208807222	-3.180667941893	1.923463029911
C	-6.501164679206	0.469542361212	2.731141424778
H	-6.658902135918	-0.085799873876	3.667709661374
H	-7.471633644424	0.567168915013	2.223598259054
H	-6.171507862181	1.485033001955	3.008862881671
C	-4.853984167551	-0.783710490379	-1.856143193579
H	-5.715273633223	-0.160392499067	-2.134160220609
H	-5.048236419564	-1.800768338366	-2.233944780732
H	-3.962564487285	-0.415418766055	-2.382642402290
C	-4.140480087316	-4.337298034963	-0.458151895867
H	-4.105259230726	-4.314551338135	0.641764312559
H	-4.247551109887	-5.386474455730	-0.776251849630
H	-5.042283313375	-3.790125016181	-0.771287201721
C	-2.656855598026	-3.635589601933	-3.080392699501
H	-3.569227526629	-3.153143617480	-3.461232936475
H	-2.635779148623	-4.673664145391	-3.449331121590
H	-1.792449066275	-3.107505988330	-3.514267644456
C	-1.077252406283	-4.570389434722	-0.645896563987
H	-0.160396862721	-4.160801091257	-1.095927779319
H	-1.163299790166	-5.623654001715	-0.958608770327
H	-0.960829614499	-4.544843077435	0.448125506656
H	1.005291326154	-2.760202366561	-2.934915277318
H	-0.794119388055	-0.130334012615	-3.076191341089
H	1.361120071268	-0.809812015352	1.901367482944
H	-1.734705674294	1.230440183068	-2.144804462675

114
2, G=-3822.26692

Si	0.396865556968	-2.730728520365	-1.168429855625
Si	1.774995648974	-0.800651304478	-1.043611667406
Si	0.189531551909	0.276956738278	0.413757802552
Si	-1.331476592502	-1.247956736721	-0.593240847187
Si	4.648119676138	-1.769143655702	-1.545861413340
Si	0.021330788201	3.155975463210	1.622718034661
Si	-3.090923521222	-3.585159860989	0.440881222055
O	1.807395090111	-0.183444860307	-2.611986376616
H	0.926286677198	-0.166703640869	-3.011165372315
O	0.458802490193	-0.066647400227	2.037655083714
O	-1.509366367063	-0.555811840364	-2.138373610660
N	3.444832395410	-0.920199470518	-0.538080567293
N	-0.101414478866	2.002150136656	0.280492120630
N	-2.800255129551	-1.857301965435	0.122697875578
C	3.777197048309	-0.433922669940	0.757709054490
C	4.497763647796	0.768724369524	0.916893647051
C	4.759138606345	1.242796757020	2.205010891459
H	5.302131677876	2.186671042275	2.313166196945
C	4.332680602320	0.570417698080	3.350137995956
C	3.658280647874	-0.640416291801	3.174887355703
H	3.344696956907	-1.211008569146	4.054540048354
C	3.388991258503	-1.163199274666	1.904761755737
C	5.010190791599	1.530961080731	-0.267633907785
H	5.036587991992	2.610864161754	-0.063500444087
H	4.395279659262	1.350767731500	-1.156121844373
H	6.039770223737	1.221230478125	-0.514567782037
C	4.565707057584	1.144817182436	4.716430170940
H	3.819466733093	1.924821022246	4.943771822977
H	5.557790504541	1.613886826611	4.793384774115
H	4.488123489656	0.375811143104	5.498103807895
C	2.695251434300	-2.487705982310	1.775191724512
H	2.759557024147	-3.056913621069	2.712824812649

H	3.125720068363	-3.089128870672	0.962763286319
H	1.623766887466	-2.365619008380	1.548314746999
C	3.866679122140	-3.345068921892	-2.205933754797
H	3.490932590790	-3.993114971431	-1.399193877827
H	4.610135974338	-3.915711303023	-2.785826326096
H	3.025944849944	-3.116468901539	-2.879797002900
C	6.106806862427	-2.164400705362	-0.435346746209
H	6.567269069395	-1.250622212537	-0.030146518467
H	6.877190677361	-2.711436547089	-1.001509944600
H	5.799828488100	-2.788549247215	0.418041248235
C	5.161879816437	-0.733908931804	-3.024579287443
H	4.254396693930	-0.359186460490	-3.523268714629
H	5.728173415454	-1.346660533035	-3.744315175986
H	5.785669578730	0.127178476224	-2.745314268191
C	-0.442848113211	2.408602729754	-1.042015544829
C	-1.800806095875	2.517603092296	-1.420637901870
C	-2.115106540194	2.776114048806	-2.757815274170
H	-3.169145766732	2.842471652593	-3.044335211352
C	-1.125570518309	2.953005540594	-3.730997266096
C	0.205795380673	2.915913686554	-3.317722685730
H	0.999242959057	3.087004349171	-4.051295227188
C	0.566443464581	2.664718118564	-1.989599799978
C	-2.885831819385	2.398662730509	-0.396917538064
H	-2.916272525705	3.296200165559	0.241755721040
H	-2.730411048343	1.545621146244	0.276164730929
H	-3.871278465094	2.288424059719	-0.865331474608
C	-1.491508403646	3.165262095863	-5.170946459755
H	-0.666252798604	3.624220118959	-5.733662912233
H	-2.376339176517	3.811448905693	-5.271268848387
H	-1.732553069025	2.207174398851	-5.661143746288
C	2.003458621616	2.726113915904	-1.576558160161
H	2.669513067860	2.504954424042	-2.419602786031
H	2.220717944189	2.022266384736	-0.763717761258
H	2.248566721713	3.732919643382	-1.196486600086
C	-1.198694328363	2.721196453312	2.979614737688
H	-2.233279514365	2.707260762833	2.604622376891
H	-1.139451595436	3.441844031184	3.810939171408
H	-0.964924852573	1.719107860501	3.369234020693
C	1.760754937087	3.145888702815	2.332148651401
H	2.040040496733	2.150974482932	2.707924917002
H	1.841339487542	3.857141811869	3.170281499328
H	2.501723426999	3.430435671885	1.569681684307
C	-0.330492464796	4.854359371703	0.902092728833
H	0.387162514402	5.101995937380	0.104727418235
H	-0.240324062299	5.619516907256	1.689569780622
H	-1.341135657263	4.926127026857	0.473671742816
C	-3.805133077289	-0.907654732611	0.483105170124
C	-3.819379716481	-0.343632548074	1.774406257005
C	-4.801328051326	0.600342260623	2.088283694391
H	-4.801565098961	1.042031590550	3.089508774669
C	-5.778223555897	0.988268604813	1.169582210279
C	-5.762465870281	0.389006072789	-0.091802789819
H	-6.528139793351	0.663606572785	-0.824161103637
C	-4.792588633342	-0.547693436200	-0.456264161141
C	-2.824036997915	-0.766499801512	2.812403185200
H	-2.883069257602	-0.117036122063	3.696179347929
H	-1.788407466165	-0.747568082490	2.444175498790
H	-3.021330178163	-1.798659759061	3.144564509464
C	-6.792843140431	2.039566446028	1.514412023387
H	-7.004083349659	2.059302190212	2.593472279221
H	-7.741495992437	1.875977684498	0.982386266933
H	-6.430949632093	3.043647344353	1.234511846788
C	-4.816961708014	-1.175331891174	-1.818751113939

H	-5.575817386319	-0.699998922820	-2.455634370662
H	-5.058682757422	-2.248969729788	-1.756173643767
H	-3.837884391809	-1.102112859035	-2.313773924315
C	-4.836597968131	-3.728816519873	1.113973416338
H	-4.959323929086	-3.149745681690	2.041823388818
H	-5.063073771795	-4.783810624175	1.336256692162
H	-5.583864698298	-3.361888333700	0.394398639880
C	-2.929462477839	-4.580998209851	-1.144110415093
H	-3.626635903823	-4.217094246173	-1.913915231362
H	-3.153814500055	-5.642266574187	-0.949414743881
H	-1.912734488778	-4.526540382045	-1.562256691532
C	-1.878171168801	-4.256410844134	1.711748022293
H	-0.836475793757	-4.110856816172	1.389961945658
H	-2.036435353759	-5.337682932283	1.855126206489
H	-2.003351808446	-3.760609064318	2.685727762695
H	0.721166148482	-3.866062102841	-0.252236810401
H	0.207216264556	-3.258889056616	-2.556724262583
H	1.388391283858	-0.101740011094	2.306663512182
H	-1.704100097711	0.394270173100	-2.144449807645

114
H2O_Int_8', G=-3822.204285

Si	0.813663514929	-0.268837043774	-2.401287880531
Si	2.106634095473	-0.199577424653	-0.490742192301
Si	0.161079173307	0.498759489152	0.710558834225
Si	-0.929711046388	-0.473884433395	-1.105409417262
Si	4.641453467527	-1.941884862100	0.032285462712
Si	-0.836738291037	2.903090958785	2.437837588410
Si	-3.107784941706	-1.870275019723	-2.749411679504
O	3.413057321184	0.860843804373	-0.774201563922
H	3.259237274759	1.763020933172	-0.454979651258
O	-0.284236087379	-0.172693433948	2.185324400862
N	2.899835694883	-1.625621208440	0.165825933636
N	0.105324601841	2.211642156026	1.091762893879
N	-2.614079052434	-0.817544921466	-1.389250609928
C	2.011455252078	-2.537643526347	0.798579250513
C	1.746399055621	-2.413225237750	2.178198291336
C	0.778250260202	-3.236881362894	2.766075166010
H	0.570172993760	-3.124871356005	3.834960683311
C	0.076478205408	-4.194372819624	2.028854676580
C	0.393955734129	-4.335934018102	0.676107714290
H	-0.126715208949	-5.092160518267	0.080697649260
C	1.356254035222	-3.539725444794	0.050852248798
C	2.496114702746	-1.413681210478	3.006331800240
H	2.021698116245	-1.269466318450	3.986039497403
H	2.559274641266	-0.439246012779	2.502127275646
H	3.532522791237	-1.748088944474	3.175231832839
C	-0.958410109951	-5.069061259658	2.674558402801
H	-0.535716775720	-6.051641766212	2.943083350989
H	-1.805897367233	-5.254405186274	1.997971076594
H	-1.350189789790	-4.617043169204	3.597095453788
C	1.712592128223	-3.771772589118	-1.384526984465
H	1.002892079670	-4.458598859589	-1.864806377091
H	2.720133472212	-4.209997301581	-1.470916451612
H	1.728024529540	-2.829523297410	-1.950962509709
C	5.144381789984	-1.843326496218	-1.773251817724
H	4.587605784095	-2.570543384540	-2.383750199105
H	6.220950133833	-2.041487569718	-1.897493571839
H	4.938736015250	-0.835901798172	-2.166588526020
C	4.927060687228	-3.666449866584	0.721762650797
H	4.608888950135	-3.732817907514	1.773818961849
H	5.999307996416	-3.915300601349	0.676165996655
H	4.375213078323	-4.434581744842	0.159562412946

C	5.651913897992	-0.703190278821	1.019857171116
H	5.449578238726	0.312228873299	0.649935983010
H	6.729271146533	-0.907287685179	0.909537419309
H	5.406607095010	-0.738737937139	2.092016322243
C	0.869410907755	3.035848719890	0.227026745599
C	0.319020081613	3.527389099518	-0.974321934543
C	1.135978030889	4.246698714884	-1.854048170134
H	0.702518937316	4.611709446864	-2.790964256689
C	2.481561635567	4.505724412297	-1.580778797846
C	2.991940139928	4.063591294063	-0.356547957601
H	4.034531310384	4.279002447039	-0.103025925068
C	2.211014978122	3.339567932209	0.551897975573
C	-1.129414499707	3.323083738742	-1.295872279835
H	-1.730925158227	4.166115400312	-0.915130580967
H	-1.517159426317	2.411073271312	-0.826133072028
H	-1.293548711731	3.259959425162	-2.380463564181
C	3.357078618421	5.180261030986	-2.594589125383
H	4.265767952657	5.595028857724	-2.136359960362
H	2.827456350178	5.993763698880	-3.111891373752
H	3.669603624125	4.451061921918	-3.361076570541
C	2.795206499340	2.878161560091	1.854575749833
H	3.888873399378	2.982574434634	1.863060329949
H	2.535993127211	1.830014599888	2.065487005458
H	2.393986437278	3.467270775533	2.694201637471
C	-2.602464453521	2.300916899882	2.308147254151
H	-3.074728409394	2.624641330131	1.368826795610
H	-3.211917161785	2.677018882295	3.145562424956
H	-2.633347534333	1.201128401682	2.333304125108
C	-0.131468622293	2.411129748444	4.107639367407
H	-0.063246983152	1.314873502330	4.159746958283
H	-0.789617972624	2.762656040498	4.918719056494
H	0.870605349112	2.832102290982	4.278219609680
C	-0.741620449223	4.769889544328	2.238528375469
H	0.298651129435	5.129443850710	2.269362316085
H	-1.292769448061	5.259893096040	3.057076910433
H	-1.181745754809	5.102495783219	1.286305227177
C	-3.582108691503	-0.372191542027	-0.438076303051
C	-3.749523814376	-1.079844588708	0.769243202631
C	-4.703490439738	-0.635841222409	1.685839467007
H	-4.825911874569	-1.185071796176	2.624274566906
C	-5.499514534538	0.485777980205	1.441801150424
C	-5.322959323691	1.160794196718	0.233957913698
H	-5.935661628304	2.041948473713	0.019511135911
C	-4.376400270165	0.756288033061	-0.711467658994
C	-2.929428648549	-2.301855358278	1.053858347269
H	-3.132878733410	-2.688127058028	2.061715917724
H	-1.849235267838	-2.102898052482	0.981170290522
H	-3.139704957492	-3.105648099904	0.329993905692
C	-6.473697754984	0.979490587727	2.470677374100
H	-6.889742113139	0.152703104999	3.064861798958
H	-7.309508410735	1.525205042700	2.009277699424
H	-5.978654794875	1.670709754052	3.173926946030
C	-4.235853251116	1.499522831412	-2.006431104341
H	-4.597190774427	2.533117394312	-1.911294326198
H	-4.824953704669	1.019683031114	-2.806293960270
H	-3.193054346249	1.517193481621	-2.349376177152
C	-4.926092220334	-2.257250261627	-2.516777995339
H	-5.103649307252	-2.754386688112	-1.550766480708
H	-5.273312371112	-2.928674821468	-3.318042486315
H	-5.544666059387	-1.347688777013	-2.539145926538
C	-2.788653125955	-0.996969256275	-4.379400750493
H	-3.419269137026	-0.103040159070	-4.493733781462
H	-2.988446093175	-1.670006846901	-5.228486848992

H	-1.735869680816	-0.678348657404	-4.443728677584
C	-2.082112094156	-3.443045599645	-2.699321404331
H	-1.011037875962	-3.208593433239	-2.799879931799
H	-2.362975877328	-4.118491097107	-3.523284964312
H	-2.219167492951	-3.979539333540	-1.747963977360
H	1.047163227060	-0.931413336502	-3.716605091685
H	-0.184394373833	-1.133215772899	2.239387249120
O	2.488051492329	1.641266695294	-3.345061461587
H	3.048265128783	1.384434386923	-2.589005653942
H	1.943260118158	2.345375036193	-2.964321564089

114

H2O_TS_4', G=-3822.185677

Si	0.395401401051	0.456914802749	-2.831646794610
Si	1.880128988225	0.406962222031	-1.019950892508
Si	0.014234469056	0.058580675647	0.444738188138
Si	-1.396287845487	0.754802776858	-1.326481276260
Si	4.741886996668	-0.496391371323	-1.730891919720
Si	-0.360750505703	0.415966117949	3.520868616008
Si	-4.027520639359	0.463793408217	-2.876104271937
O	2.508771857144	1.990935627956	-1.106592969478
H	2.446508568879	2.506090750606	-0.284345912619
O	-0.142773297635	-1.493023244620	1.063090232830
N	3.186589900093	-0.761849947706	-0.909844612462
N	0.016231232722	1.042851965345	1.901252215677
N	-3.043663991025	0.117933447287	-1.438981634215
C	2.827382457491	-2.000989414013	-0.306706349886
C	3.256529727162	-2.318950949794	1.000543110396
C	2.765796623029	-3.469284992385	1.621558887834
H	3.082123181062	-3.684647609189	2.646466406450
C	1.869247315203	-4.333585693588	0.990624189902
C	1.513462544407	-4.044041239055	-0.328352284424
H	0.847857659431	-4.726267168893	-0.865973241623
C	1.987021296967	-2.906600587103	-0.993696563419
C	4.241258760414	-1.453855363756	1.725646823225
H	4.073672308977	-1.483797537338	2.811321755953
H	4.186089262444	-0.415170278473	1.384228481045
H	5.272054297626	-1.801520084161	1.541252974513
C	1.286717792290	-5.512415621277	1.712625259932
H	0.383147162972	-5.216506136128	2.271791250237
H	1.996780017097	-5.932721539297	2.439510185814
H	0.994723722855	-6.310731612902	1.015203238207
C	1.589707854896	-2.678963702220	-2.422739181464
H	1.437890929760	-3.635780159214	-2.941917654991
H	2.345862537493	-2.096742945040	-2.965197808065
H	0.633213225193	-2.129351632358	-2.499195889718
C	4.396351854019	0.227926862470	-3.428998263132
H	3.776451428568	-0.440678405050	-4.045978108655
H	5.339256228974	0.414355243301	-3.967684657080
H	3.868747575655	1.190096548951	-3.333888933545
C	5.584152915324	-2.166528220795	-1.858659944702
H	5.766108284789	-2.602685177266	-0.864536722298
H	6.554644370523	-2.068564422244	-2.370196633224
H	4.967193008696	-2.879495410141	-2.426985062997
C	5.801282969218	0.730855763890	-0.782674606456
H	5.225923291764	1.656824820142	-0.628296875251
H	6.708754527106	0.984221783670	-1.353976562030
H	6.113484489504	0.345075442104	0.198791619279
C	0.390465799979	2.393305516157	1.671143739133
C	-0.561895818253	3.350823282491	1.273064115494
C	-0.134066686653	4.640852504736	0.935412418981
H	-0.881990048609	5.369960547209	0.609073581747
C	1.203269079525	5.024643524968	0.999869537126

C	2.130375697772	4.077090342246	1.451294981086
H	3.183637223419	4.360227538574	1.548056113142
C	1.747837134431	2.777728502595	1.792631244747
C	-2.023967522889	3.026566334280	1.251254823711
H	-2.517245851096	3.415190647599	2.158978110690
H	-2.194312608042	1.943434789587	1.227320845156
H	-2.520371386138	3.482461827409	0.382807831750
C	1.646427221956	6.400783948065	0.596594693186
H	0.791737696266	7.082590787778	0.485346949889
H	2.181051033023	6.378613104605	-0.367477787862
H	2.335244812594	6.835014906141	1.337296699424
C	2.762901524191	1.798901974801	2.298700262138
H	3.784649188313	2.090576514332	2.016916442200
H	2.558171937081	0.788493023411	1.922021159917
H	2.728649355612	1.730841617517	3.398455406367
C	-2.051576205650	-0.386995126618	3.500598503863
H	-2.837340821942	0.343318979284	3.255144756479
H	-2.290750369832	-0.830747306719	4.480632783905
H	-2.086045606492	-1.183162359336	2.741560374003
C	0.919548835981	-0.838640953561	4.089456626624
H	1.006158330281	-1.632071184410	3.332127361319
H	0.613832399521	-1.297079806286	5.043833085259
H	1.910135996287	-0.381913565746	4.234020111399
C	-0.354716371271	1.908556159329	4.664216045620
H	0.622578440695	2.415361196929	4.674808489564
H	-0.585698150893	1.595150175093	5.694845123902
H	-1.109930503299	2.648269327713	4.356596436510
C	-3.532946978644	-0.735307724290	-0.407618293239
C	-3.103899346771	-2.077449598209	-0.340653414519
C	-3.564000061992	-2.893617940395	0.692704150573
H	-3.210398804370	-3.928203976252	0.741500985993
C	-4.460890523915	-2.431007869644	1.657551819886
C	-4.903921955177	-1.113052045096	1.551460540631
H	-5.611446705977	-0.726815614935	2.291909890947
C	-4.456063567058	-0.254850412887	0.543749097352
C	-2.180030247992	-2.623097197109	-1.383729026174
H	-1.934864458634	-3.675978989781	-1.186740395805
H	-1.240938870707	-2.049048620321	-1.419844033153
H	-2.621780096717	-2.548654158469	-2.390167658913
C	-4.894895544113	-3.304708681152	2.797907050293
H	-4.861521614364	-4.370446937228	2.527990872883
H	-5.917506861058	-3.066145830328	3.125578582430
H	-4.232135636206	-3.165330241630	3.669126828449
C	-4.980246627091	1.149056375645	0.479377987631
H	-5.249564472650	1.517464500466	1.479708547393
H	-5.889312749524	1.207754244923	-0.143170113289
H	-4.245012080048	1.830385898944	0.035504784593
C	-5.557401937671	-0.617927953702	-2.792042262106
H	-5.280113650403	-1.682847723811	-2.754043138163
H	-6.184308149161	-0.457370795006	-3.683430214707
H	-6.165702564803	-0.403904913499	-1.901044000544
C	-4.469851636253	2.289405606012	-2.955852047613
H	-5.162731269975	2.583412065634	-2.153970958098
H	-4.937401673488	2.537039617070	-3.922355821647
H	-3.556374879053	2.898198314122	-2.857268530817
C	-3.046881068267	0.053109021324	-4.428922033445
H	-2.197589172953	0.741339372626	-4.563022113927
H	-3.691519715788	0.144597176361	-5.318377461403
H	-2.647268247505	-0.972152397808	-4.397139245351
H	0.644061758592	0.004944481923	-4.232655141888
H	0.289757261077	-2.209422770810	0.580520255960
O	0.398025344165	2.293613875703	-2.936302649332
H	1.187679816002	2.596494341817	-2.432408152788

H	-0.537157460208	2.268191953490	-2.148991107713

114
2', G=-3822.253534

Si	0.429441617976	2.459430523644	1.415916340049
Si	1.824979347715	0.566082881322	1.073228255687
Si	0.119394069329	-0.396751604835	-0.340207614651
Si	-1.371964727893	1.143583679251	0.690889635816
Si	4.637530069305	1.783272237461	1.214409772065
Si	-0.328315308439	-3.241040352328	-1.553729929063
Si	-2.936919486228	3.617379372313	-0.313980357242
O	2.164919923612	-0.209939087697	2.527435959183
H	1.491955518576	-0.827942124904	2.843747449606
O	0.316380386270	-0.067120722701	-1.974498154940
N	3.378961689060	0.897813952554	0.323995033607
N	-0.212284225762	-2.110365383003	-0.191884667227
N	-2.778717474619	1.868511487312	-0.068013513078
C	3.589013387979	0.430423906459	-1.003114681315
C	4.267701652506	-0.781114835157	-1.252889733602
C	4.383379030185	-1.239049027651	-2.568274594164
H	4.891768578489	-2.191532105795	-2.746671056609
C	3.861140713860	-0.534475862741	-3.653372043993
C	3.232339011442	0.685756913223	-3.391294476235
H	2.834428857852	1.273410820183	-4.224104298856
C	3.094952812371	1.185071434268	-2.091030375799
C	4.886700862173	-1.570133734791	-0.138527510041
H	4.850914379607	-2.648658623200	-0.348319129791
H	4.388254424239	-1.374946067002	0.817779382419
H	5.948661091881	-1.297689689511	-0.017298999047
C	3.945986384985	-1.086470281043	-5.046072794856
H	3.184226756670	-1.868682609172	-5.202804873158
H	4.926490534374	-1.546850870996	-5.238571169328
H	3.779145489087	-0.306049251271	-5.801852173298
C	2.388959534873	2.487078679557	-1.857848104021
H	2.305603236806	3.063748234053	-2.789241161939
H	2.901221812595	3.098035794860	-1.102573480489
H	1.362608689965	2.322417950760	-1.490087126544
C	3.799547369366	3.159873613511	2.188187770238
H	3.242104772633	3.848000401842	1.534225791154
H	4.556771974809	3.741653734368	2.738007607190
H	3.105791914593	2.734777826540	2.932482879165
C	5.845132685219	2.465693162152	-0.046777425461
H	6.298499626644	1.651320902415	-0.633187118321
H	6.656990936394	3.013354365555	0.457230418274
H	5.353998180327	3.151687278208	-0.753503436235
C	5.520200292865	0.698935976644	2.467989421406
H	4.767775316576	0.162840065581	3.067331200402
H	6.129232903527	1.318641769042	3.145960805189
H	6.182953832582	-0.042097730887	1.999018393871
C	-0.373058989590	-2.525236545675	1.161435086581
C	-1.647955257818	-2.513606664195	1.763709238914
C	-1.756836124509	-2.809349013528	3.124649947148
H	-2.747744442936	-2.781629519703	3.587899457142
C	-0.646614255636	-3.132878337915	3.907483436482
C	0.598428082559	-3.201068748046	3.276734604859
H	1.478730342362	-3.496503488435	3.856236369132
C	0.752634126973	-2.918844477134	1.915080806541
C	-2.874123760640	-2.239544484968	0.950234883747
H	-3.165096602309	-3.133287345353	0.373125913161
H	-2.719025777850	-1.437785955040	0.216369831962
H	-3.726158962686	-1.964369063238	1.584678881609
C	-0.786070834429	-3.386993213821	5.380228585317
H	0.031414030395	-4.015762435610	5.761446697615

H	-1.739042031682	-3.883635528668	5.614942747083
H	-0.764735522736	-2.440940146891	5.946919357003
C	2.084789855974	-3.088845949177	1.251840741574
H	2.905210888455	-3.052799460097	1.980083731290
H	2.255898003965	-2.320334851626	0.487373742310
H	2.133608072156	-4.062134954669	0.734699366621
C	-1.667223819872	-2.695517464613	-2.746114260721
H	-2.634975253277	-2.576039789840	-2.235400124932
H	-1.790477435859	-3.426518506759	-3.561307952280
H	-1.395115039512	-1.725433945711	-3.187462486670
C	1.309585360104	-3.343675803523	-2.473237032049
H	1.616520815768	-2.355943712711	-2.847558387864
H	1.225083485120	-4.022353458391	-3.337572748935
H	2.114141164993	-3.718370232833	-1.822555549840
C	-0.734269421502	-4.918166069234	-0.812253215961
H	0.038774355223	-5.242434662920	-0.098801061346
H	-0.799985612016	-5.675024363535	-1.610048959343
H	-1.695608734348	-4.904331429172	-0.276947703396
C	-3.816569814110	0.969304390043	-0.455681785167
C	-3.841802368025	0.434299447738	-1.758542419094
C	-4.832557118780	-0.494889294906	-2.088549866488
H	-4.840402955020	-0.914697206859	-3.099050733626
C	-5.807958240443	-0.896540895615	-1.174010445397
C	-5.786498030003	-0.320217392438	0.098008896148
H	-6.552135559915	-0.602963463394	0.827259106954
C	-4.809587542148	0.603238672132	0.474972527409
C	-2.842107072106	0.872715752952	-2.785315065879
H	-2.939103552726	0.277071946677	-3.703149361421
H	-1.805435010593	0.785594467156	-2.428915562482
H	-2.997827911605	1.929518912260	-3.055973265201
C	-6.831393513680	-1.933402146458	-1.537114034088
H	-7.037581732252	-1.936985185642	-2.617343316585
H	-7.781117462162	-1.766842136883	-1.007772802461
H	-6.481746047558	-2.944716431312	-1.268220937446
C	-4.813964209347	1.196325387166	1.851846101185
H	-5.634375025287	0.784663336044	2.455739021287
H	-4.932744134863	2.290560767092	1.817637396501
H	-3.865886573175	1.003870727721	2.377566263305
C	-4.648392080792	3.913269672042	-1.023895721913
H	-4.780433244444	3.396520163679	-1.986701657246
H	-4.807444333121	4.990807924521	-1.188602901511
H	-5.434308685881	3.550854061750	-0.343855890147
C	-2.751212517771	4.519635855031	1.325543283905
H	-3.601763388535	4.296664245875	1.988365895448
H	-2.724217972493	5.609471020519	1.163764018646
H	-1.834486330952	4.235917386624	1.866910078798
C	-1.634582226875	4.247228262340	-1.516686160202
H	-0.618102255263	4.049244066243	-1.145342292708
H	-1.733490218749	5.336405548787	-1.653229900710
H	-1.734573189536	3.766982453235	-2.501408987575
H	0.809384433132	3.606558083128	0.525900407158
H	1.227792074996	-0.016995682551	-2.298727913396
O	0.173484610953	3.042000168860	2.978366564527
H	0.888084461756	3.590406572425	3.318174351605
H	-1.707221181496	0.450658326334	1.988748088254

114
H2O_Int_8'', G=-3822.205809

Si	0.422244413400	-0.652312991183	-2.404211273234
Si	2.002826633172	0.029195427448	-0.865959037181
Si	0.170785542409	0.613535690872	0.565839618820
Si	4.986245271321	-0.774243522015	-0.372077949304
Si	-1.178115485514	2.955566498373	2.132675776435

Si	-3.083810528495	-2.742677904704	-1.982870470202
O	3.011871011217	1.284286591768	-1.368382629291
H	2.546291455644	2.042742132980	-1.740312548212
O	0.058063986618	-0.199968200155	2.091907709010
N	3.239205735024	-1.011271900457	-0.127341216730
N	-0.093497769323	2.307334761903	0.876775510542
N	-2.680158080364	-1.376015339953	-0.910059050684
C	2.757418017823	-2.139008086349	0.577274558440
C	2.594720816410	-2.081277693228	1.978037199596
C	2.032867412650	-3.165878009243	2.659020363929
H	1.899456979311	-3.097368190625	3.743022139822
C	1.627498933805	-4.324940791663	1.995570263406
C	1.845417774408	-4.390784425366	0.614279568517
H	1.571403369184	-5.301610392138	0.073124859675
C	2.419798529373	-3.333498068639	-0.102964398661
C	3.050765942436	-0.871291028234	2.736155605897
H	2.594613710296	-0.823964266655	3.736879282940
H	2.845292126379	0.054601392838	2.179987375348
H	4.142917821707	-0.889712553641	2.884144940318
C	0.948661144685	-5.446465649938	2.724700818252
H	1.217260953651	-5.456482567671	3.790635385155
H	1.209604205236	-6.424440376204	2.294713688415
H	-0.148018377343	-5.344427861225	2.662274154489
C	2.737463899940	-3.503066320153	-1.556210966677
H	2.129005334055	-4.297970068283	-2.009293098426
H	3.797759128503	-3.782002543319	-1.678376078679
H	2.575179459030	-2.574853940298	-2.116226968953
C	5.389393151114	-0.734363304680	-2.203812687252
H	5.088901778727	-1.669854286572	-2.699968229422
H	6.469887049272	-0.591610804422	-2.365756328239
H	4.852298894428	0.100132349631	-2.677926392887
C	5.831446499400	-2.252863915276	0.431484296614
H	5.619622620531	-2.317831541745	1.509810821015
H	6.922766176064	-2.164292595546	0.307799629860
H	5.516416093956	-3.203208826011	-0.025863288521
C	5.597556949669	0.806904929647	0.436423913125
H	5.079082218201	1.665305494657	-0.014139831921
H	6.681583820659	0.925347417435	0.276987741849
H	5.411413954833	0.813959168109	1.521277807078
C	0.611071951288	3.198788372311	0.007837102111
C	0.023388564880	3.613474968984	-1.203618068466
C	0.739828491695	4.469628917449	-2.045203480269
H	0.281532448922	4.780582628944	-2.988761999921
C	2.017951598281	4.931120330411	-1.721801785996
C	2.584318898702	4.495996256371	-0.521087251577
H	3.595702088971	4.818751677968	-0.256798537778
C	1.908263786545	3.633834124430	0.345047583223
C	-1.315026627572	3.090170854017	-1.623598772255
H	-1.737847361738	3.690150695144	-2.441339723107
H	-2.034825021671	3.062787086337	-0.793940856487
H	-1.209965636895	2.053886944310	-1.987528539996
C	2.751735213969	5.879916395931	-2.624497694266
H	2.551627555438	6.927290341985	-2.342131650373
H	2.442672353784	5.759663026731	-3.672909863551
H	3.839850899423	5.729837419192	-2.568472901010
C	2.596495199327	3.101666966935	1.563214949050
H	3.525926994386	3.649833111431	1.767905380342
H	2.870013011270	2.045767300383	1.397844384512
H	1.957660919763	3.137418539159	2.454514743683
C	-2.724766209156	1.910974211031	2.174930164690
H	-3.236156671432	1.884130377256	1.201298333061
H	-3.436094397392	2.292704280273	2.924277105136
H	-2.477206220689	0.874520663575	2.448741010884

C	-0.383938092512	2.890220822355	3.841672352202
H	-0.051754902962	1.869231896981	4.084672522749
H	-1.123386721735	3.186385598544	4.603710398236
H	0.477149224096	3.569278886610	3.934482098712
C	-1.533035819992	4.748469637587	1.713183692206
H	-0.600281037979	5.330430636814	1.655044867664
H	-2.166797982294	5.195581915606	2.495668607668
H	-2.053250766474	4.852949480852	0.749710584577
C	-3.732557560145	-0.627294918037	-0.303247116282
C	-4.202494515593	-0.972935723235	0.980685055998
C	-5.271712216145	-0.262962744816	1.528392108089
H	-5.631671096712	-0.542597614980	2.523388216211
C	-5.880621019966	0.798542797264	0.856549690581
C	-5.394265029073	1.129758938388	-0.407850336632
H	-5.852963305916	1.957596204171	-0.957647691272
C	-4.338545193563	0.433465778406	-1.005781175323
C	-3.548092120303	-2.061858588119	1.775870000884
H	-4.211316758163	-2.416802674625	2.577004528703
H	-2.621836578682	-1.696571775350	2.249553724414
H	-3.254367269545	-2.912900324513	1.147330289553
C	-6.987006434075	1.585241106221	1.496577345887
H	-7.629699720932	0.945690728982	2.119579106963
H	-7.620200334832	2.079287395370	0.745550110748
H	-6.579496351321	2.373681465290	2.152124294813
C	-3.898635179787	0.805682815677	-2.390749946959
H	-4.043587022534	1.879730706614	-2.573652979437
H	-4.491290948351	0.265541433606	-3.147777448381
H	-2.844499554768	0.555431541241	-2.568731635713
C	-4.855087672245	-3.236365150154	-1.619970553467
H	-4.987604162950	-3.540210532708	-0.570820848991
H	-5.151103911065	-4.079208611767	-2.264534349976
H	-5.542890418963	-2.398863284734	-1.813665139231
C	-2.894268123031	-2.304398475373	-3.799052309009
H	-3.683715664937	-1.620191516390	-4.141836406532
H	-2.943962042378	-3.217393649740	-4.414295775488
H	-1.922036613585	-1.821952680887	-3.986374528014
C	-1.895598784141	-4.153225290577	-1.602158518930
H	-0.857956503262	-3.871807528057	-1.847053052803
H	-2.150838120366	-5.034127613413	-2.212929754762
H	-1.934423584721	-4.442507163990	-0.541114345087
H	0.212805131602	-2.887107479856	0.461577923910
H	0.786164067057	-0.033175993135	2.701735608169
O	-0.561120843743	-2.341508201417	0.712798411269
H	-0.256780784036	-1.768441960639	1.470677316699
Si	-1.000568301839	-0.979870789706	-0.692819559223
H	0.709622875191	-1.786526861099	-3.352971729624

114
H2O_TS_4'', G=-3822.188723

Si	0.737377241575	-0.418080931605	-2.629145319866
Si	2.096460819736	0.199532743540	-0.764831960042
Si	0.199768927437	0.336581341042	0.762111585450
Si	5.046953208938	-0.774256347756	-0.607121614009
Si	-1.135819128798	2.532196858115	2.506542193886
Si	-3.192642721836	-1.753695674570	-2.820548299870
O	3.054158574570	1.556729538420	-1.016264349056
H	2.594387592606	2.334629224337	-1.356609058944
O	0.049272457284	-0.397536352421	2.271258483181
N	3.320186951389	-0.948683554045	-0.205768138128
N	-0.180328527155	2.012609849064	1.089914847523
N	-2.653537388871	-0.983357320492	-1.292639379458
C	2.857263037422	-1.995038551023	0.629073347723
C	2.739313108470	-1.789452275748	2.023280617398

C	2.159866271799	-2.783884383252	2.820079367791
H	2.064644607683	-2.606077465099	3.896083151517
C	1.702670853725	-3.991835120585	2.284374184657
C	1.902988521774	-4.210045608114	0.919273852209
H	1.598147333454	-5.165135744259	0.480714662722
C	2.493232411228	-3.249404483990	0.086869375383
C	3.247650505632	-0.525378207279	2.645951652603
H	2.822952283246	-0.368543241532	3.646379581661
H	3.024325272635	0.350443613797	2.022403624110
H	4.344780914091	-0.562925134987	2.744460690922
C	1.010678934313	-5.009892208889	3.142083462463
H	1.367838742167	-4.971955876346	4.181284533228
H	1.164879347931	-6.029949748826	2.762030632853
H	-0.077492706628	-4.829760138938	3.163638903927
C	2.750437327811	-3.568127360160	-1.355489545793
H	2.095184432370	-4.375987472254	-1.709827059333
H	3.791453827482	-3.902274716474	-1.492876415755
H	2.606232763988	-2.688605848271	-1.997732389948
C	5.204964735576	-0.605156213979	-2.467887260922
H	4.784667988911	-1.480365478623	-2.986625664388
H	6.258137730874	-0.494986097059	-2.771230856821
H	4.657928330765	0.289919034545	-2.801782277054
C	5.916521349144	-2.317128671917	0.026316357597
H	5.747257288132	-2.451349916123	1.106398301697
H	7.002054236623	-2.213295993721	-0.131261041210
H	5.588829985508	-3.235480000157	-0.481758687524
C	5.823782877459	0.716812964392	0.230591854907
H	5.244531466363	1.617432223872	-0.016919472110
H	6.861066427588	0.852374330030	-0.116562230695
H	5.845848526026	0.603616129568	1.325129601689
C	0.337622303942	2.984853723871	0.185185602372
C	-0.347172623347	3.288953116194	-1.009294478070
C	0.206353980578	4.209864194886	-1.904640137072
H	-0.330323547365	4.423657612273	-2.833983029124
C	1.411774015474	4.862043678911	-1.647310068528
C	2.064366020493	4.567023371782	-0.446402170506
H	3.017998986114	5.054513930599	-0.222044447353
C	1.553691730893	3.645564486444	0.470670156520
C	-1.653736972095	2.641274336768	-1.346187914206
H	-2.361143207849	3.372432799924	-1.764298562942
H	-2.112111411498	2.172885297262	-0.465968145003
H	-1.513914099822	1.855406237093	-2.108489785006
C	1.974949258921	5.869793073024	-2.606615551665
H	1.675263519578	6.892829988984	-2.323638803506
H	1.617748481246	5.694945269557	-3.631543237301
H	3.074615151300	5.845508173465	-2.617790527436
C	2.322580578566	3.346823895662	1.720179605600
H	3.398199100761	3.509198388965	1.567611470070
H	2.163445701919	2.311957154739	2.047664623261
H	1.999472340627	3.996728248802	2.549854480085
C	-2.658748921107	1.457545258522	2.643669338747
H	-3.276598723833	1.487293957210	1.734034538104
H	-3.283820366739	1.784536899991	3.490290956421
H	-2.371753162597	0.410623813659	2.819195489129
C	-0.161061418418	2.379531188112	4.106675301058
H	0.227104565321	1.353502098583	4.191191421337
H	-0.819554477562	2.572244265626	4.969250209033
H	0.683602110956	3.080952509312	4.160955106339
C	-1.597644680616	4.324950095074	2.193224843329
H	-0.706283533434	4.962460331702	2.089097361652
H	-2.197214780806	4.709952500491	3.033463295873
H	-2.191646741456	4.428447271328	1.272169781454
C	-3.609752892046	-0.771882843546	-0.251156534510

C	-3.614068954210	-1.598655961312	0.895013192413
C	-4.582673682603	-1.398822881498	1.878921919877
H	-4.572550397383	-2.047438025200	2.760302556445
C	-5.556601893102	-0.404406539637	1.776674135682
C	-5.540705092122	0.395628708692	0.636412152959
H	-6.291372011933	1.184566609726	0.526413304597
C	-4.590199666551	0.235047642321	-0.376757471296
C	-2.589352577711	-2.677371721151	1.083959143655
H	-2.924062624404	-3.404184246182	1.837397642957
H	-1.638618352499	-2.247926931020	1.442742042216
H	-2.372686940019	-3.207868328750	0.146861289831
C	-6.550207835333	-0.176904134016	2.877610141725
H	-6.839376094646	-1.120753639118	3.363172406094
H	-7.462812717132	0.311037462298	2.505921217818
H	-6.124045369839	0.474343206298	3.659645400370
C	-4.662683716430	1.128601476725	-1.579643553801
H	-4.949364898523	2.150368070440	-1.291598740701
H	-5.419695542285	0.772268620711	-2.298055695992
H	-3.705303303450	1.169360413470	-2.107670656740
C	-4.969263210684	-2.304420198619	-2.577299864441
H	-5.042536523512	-2.990766825515	-1.719378889983
H	-5.307587036261	-2.843767883940	-3.476479712629
H	-5.662429236884	-1.471532092311	-2.395959534346
C	-3.011011491995	-0.571827400595	-4.269894965204
H	-3.714206412655	0.271695955883	-4.223607448199
H	-3.184467999434	-1.109999592337	-5.215652994654
H	-1.986946404830	-0.166339608570	-4.301917520718
C	-2.159213188173	-3.275159408696	-3.204697910432
H	-1.123695930608	-3.012498309273	-3.468251399678
H	-2.605873373457	-3.795096250780	-4.068260196878
H	-2.128770952753	-3.974637544284	-2.356314724782
H	0.460925978798	-2.117995060599	-2.001309059236
H	0.572995087695	-1.203319677697	2.381767069498
O	-0.180376873311	-2.372792871266	-0.964539627773
H	0.374477682958	-2.670252333437	-0.218697427387
Si	-0.955377291326	-0.693494063887	-1.022198016748
H	0.664831740262	0.623675065047	-3.711810058473

114

2''', G=-3822.26227

Si	0.428670265952	-1.227258063546	-2.399007223287
Si	1.955529266365	-0.073444696614	-0.978433087663
Si	0.172789884180	0.367393082291	0.604639363612
Si	4.811516550757	-1.193235235615	-1.224996016892
Si	-1.040456655722	2.762430502963	2.173718010249
Si	-3.312992680347	-2.675102253274	-2.072014754352
O	2.721659279905	1.223969470344	-1.714451869263
H	2.153899872863	1.912659849745	-2.085956129996
O	0.199888042672	-0.385100022400	2.120690929045
N	3.282339308036	-1.012213778257	-0.333008844149
N	-0.073860395794	2.089238403852	0.832607528537
N	-2.793813668565	-1.381320131573	-0.949659552015
C	3.076172238439	-1.586308640795	0.950745818414
C	3.330050853724	-0.811598185711	2.107458744731
C	3.015329230008	-1.333894697666	3.364570999516
H	3.205000191603	-0.722834444070	4.252846880055
C	2.476869000072	-2.618917416512	3.515968949444
C	2.290331854756	-3.383444582275	2.366646757910
H	1.889263068660	-4.397328747914	2.460123429334
C	2.581013583054	-2.897346068859	1.085022145513
C	3.943298867040	0.550656006872	1.987236063005
H	3.793103342285	1.138053165174	2.903574863622
H	3.542271417172	1.109880586715	1.130940489830

H	5.029460337295	0.471460087842	1.818482846170
C	2.098210536112	-3.137056290846	4.872524862083
H	2.853223619805	-2.876777069188	5.629582852457
H	1.980268468006	-4.229726926277	4.867846223156
H	1.141011613760	-2.702624721285	5.206070218829
C	2.333133081312	-3.777316100724	-0.103322559614
H	1.251314546746	-3.935949380702	-0.226024064401
H	2.814000456912	-4.758374253969	0.031329692739
H	2.710039741905	-3.322110022631	-1.026371938316
C	4.381930175073	-1.509217209128	-3.024815465728
H	3.771040809878	-2.416724173424	-3.147492720948
H	5.296925885172	-1.625920633930	-3.627065502483
H	3.817723012394	-0.655645167711	-3.432755875213
C	5.739744052811	-2.634534593262	-0.461902742708
H	5.903644265390	-2.469270232884	0.614584872888
H	6.725903159888	-2.740045486823	-0.941504250256
H	5.198179002636	-3.584610532115	-0.578471708674
C	5.861545383765	0.360717089600	-1.123116559735
H	5.246490454206	1.228509988741	-1.404700565455
H	6.716851354184	0.294182034508	-1.815001969529
H	6.259056167664	0.526298647134	-0.110486790983
C	0.476766629734	2.934781213553	-0.171880256400
C	-0.111699079133	2.979911701688	-1.452062196674
C	0.499427204848	3.731056266944	-2.464155268980
H	0.039635329782	3.745377376368	-3.456819725471
C	1.662514635765	4.469049511023	-2.238900831948
C	2.201526729485	4.449040295347	-0.949488823700
H	3.113154056428	5.018191016004	-0.744019462251
C	1.635464495425	3.701842069475	0.083174915622
C	-1.380490205868	2.237838616825	-1.743030347116
H	-1.890047660760	2.662084502291	-2.618572949638
H	-2.068461744128	2.255090732213	-0.886186854102
H	-1.197951695370	1.174890984233	-1.973387429358
C	2.316927153603	5.258792790077	-3.334654276568
H	2.386822448256	6.325728207001	-3.070076276604
H	1.757271971317	5.181020967808	-4.277137635384
H	3.343271475485	4.904706322781	-3.521531218348
C	2.268340760407	3.716005926802	1.439851776839
H	3.341539952850	3.941889804358	1.374111717180
H	2.137152002769	2.753810218715	1.947980955098
H	1.808332110712	4.484878178644	2.082807272218
C	-2.612256760410	1.766371273643	2.327188785418
H	-3.183646347988	1.737998478504	1.387478803806
H	-3.264066326954	2.187810628425	3.109105607055
H	-2.384352232012	0.728844311449	2.612898447295
C	-0.134530596386	2.653489751262	3.818264370780
H	0.180980586378	1.612462606295	3.987214995052
H	-0.814718758637	2.936981149136	4.637816423024
H	0.751428656529	3.301276926418	3.873208237695
C	-1.419935224724	4.543467915616	1.722055522386
H	-0.508965206368	5.149368177062	1.605864115965
H	-2.039747358030	5.005765481478	2.506676774756
H	-1.976538347995	4.593724464677	0.773357281653
C	-3.795088571857	-0.686464656301	-0.208272467938
C	-4.085086741566	-1.048424855778	1.126088655829
C	-5.114049143827	-0.393384301582	1.802974141167
H	-5.330935536712	-0.688415482408	2.834291735880
C	-5.858690989927	0.631818808324	1.217077960901
C	-5.555409110445	0.977556409452	-0.098535257614
H	-6.126480982844	1.772691697454	-0.588166638252
C	-4.549926036342	0.331653516137	-0.825803751359
C	-3.288944754209	-2.100358222441	1.837662630799
H	-3.826625249943	-2.464226119275	2.724160734303

H	-2.322428846763	-1.695039163718	2.182224350338
H	-3.049558492334	-2.951198992267	1.185756191940
C	-6.916841179858	1.359085154917	1.994024671908
H	-7.482087371396	0.674724687731	2.644318356601
H	-7.630554812903	1.867111666877	1.329647753324
H	-6.467971333129	2.128230685653	2.645332760535
C	-4.333778003058	0.703255554977	-2.262430863554
H	-4.523929215674	1.773415746612	-2.427737459795
H	-5.024553505414	0.147612505228	-2.919169994115
H	-3.315865081070	0.464920952661	-2.590179328795
C	-5.130826986197	-2.997600465661	-1.744931672254
H	-5.294752137891	-3.284213584023	-0.694820624788
H	-5.485751911913	-3.819342825818	-2.387050598661
H	-5.750557371713	-2.111222801095	-1.946349779942
C	-3.042064130141	-2.209421911604	-3.873872390238
H	-3.684850272235	-1.378064758165	-4.195907924682
H	-3.263260022618	-3.078904278386	-4.514099967069
H	-1.995606682300	-1.920174901634	-4.056889262219
C	-2.314620515701	-4.230494925337	-1.733713286588
H	-1.242064173872	-4.082337956951	-1.931471101379
H	-2.669305055762	-5.043136819647	-2.388533546178
H	-2.412851048113	-4.553874174664	-0.687227560960
H	0.854579538561	-2.555261430038	-2.942579776425
H	1.076288150660	-0.501959680515	2.523083871032
O	-0.515962888191	-2.562153397717	0.322750293536
H	-0.259452981750	-2.274666828900	1.212926533686
Si	-1.087687345739	-1.253560931635	-0.592232897672
H	0.002157904675	-0.360085257779	-3.548707447229

114

H2O_Int_8''', G=-3822.198076

Si	0.651187318267	-0.130285339845	-2.549619850981
Si	2.071220479744	0.242329324172	-0.750784358901
Si	0.118481258925	0.621315931486	0.571211866258
Si	4.592127580814	-1.551838330136	-0.823751769364
Si	-1.252035429469	2.897399816014	2.172530538421
Si	-2.789043007451	-2.670045293286	-2.478243629961
O	3.164268449956	1.498420192405	-1.006652954780
H	2.750801476868	2.301301606439	-1.355472798385
O	-0.100360008820	-0.068467758310	2.081999216616
N	3.051268639263	-1.052187585110	-0.076857447450
N	-0.325544848956	2.302085527565	0.772600650007
N	-2.457021211310	-1.417254667584	-1.231042266809
C	2.721374944400	-1.461012237059	1.251071493507
C	3.096095989926	-0.678011883280	2.366332138247
C	2.695869081583	-1.078290628900	3.650250079889
H	2.976031821763	-0.450056253097	4.501366105947
C	1.951189503090	-2.235298309545	3.874997643074
C	1.587125077887	-2.993348344144	2.758432612068
H	0.974016648159	-3.888652026411	2.897217296858
C	1.941584252771	-2.625047748374	1.460439566572
C	3.889302137977	0.590043240257	2.235055541713
H	3.516363270543	1.349424134791	2.937986210409
H	3.860588480967	1.005196620270	1.220707028728
H	4.947568404075	0.414145945125	2.485063777638
C	1.547983391082	-2.653724963407	5.258075059491
H	1.675641867254	-1.835334945235	5.980482005272
H	2.158163305204	-3.503392545029	5.606388336137
H	0.496249937082	-2.976224390021	5.288683574887
C	1.423958827645	-3.425878090986	0.307175092602
H	0.793365865659	-2.796231010665	-0.340965844408
H	0.809392141929	-4.266364941987	0.655933990296
H	2.228377914022	-3.828517601271	-0.320685903566

C	4.538750757013	-1.022236699145	-2.621200580412
H	3.753960875090	-1.575138848371	-3.156914145738
H	5.503226868186	-1.260071883691	-3.097580444663
H	4.363438519357	0.059430677310	-2.717726222970
C	4.796529852499	-3.413732836127	-0.706328404668
H	4.610810568330	-3.783129079833	0.314418617391
H	5.828782395627	-3.687454063171	-0.977939510424
H	4.114495941646	-3.922611941289	-1.404213448024
C	6.056941173756	-0.740316895158	0.029005296935
H	5.981537371790	0.356183712129	-0.014175546596
H	6.989466644009	-1.042454641419	-0.475196106729
H	6.134075668170	-1.047183142766	1.083270938549
C	0.077573260502	3.171179833980	-0.280380395205
C	-0.735735848280	3.356838665048	-1.414436757400
C	-0.262348197362	4.136598850906	-2.474380860141
H	-0.895777781821	4.258725353619	-3.358224326137
C	0.987003710817	4.753645990854	-2.442483400570
C	1.759265254534	4.599926852158	-1.285873832605
H	2.729130983012	5.102969660010	-1.217604391074
C	1.324194590471	3.830969551032	-0.203277514551
C	-2.110748225118	2.767800448674	-1.487285763713
H	-2.867172429891	3.500461271880	-1.157027587520
H	-2.212371205488	1.891507639630	-0.833793655313
H	-2.361540820852	2.474261770271	-2.515948485529
C	1.498470337414	5.541901964176	-3.613012414642
H	2.098489078872	6.404691257215	-3.287873069047
H	0.675057035623	5.913700491044	-4.239448358359
H	2.144436286154	4.920278966310	-4.255626473364
C	2.160126754483	3.728582402743	1.035483689145
H	3.195142041256	4.043478359799	0.846859833618
H	2.173148639144	2.704209674445	1.427544703550
H	1.750922090357	4.369852861036	1.833447388957
C	-2.815935497659	1.884872296235	2.357572838812
H	-3.450158138506	1.942929210339	1.460003184463
H	-3.409235731570	2.233803500458	3.218275789780
H	-2.569732032725	0.825182931738	2.523209048572
C	-0.247089301482	2.781880106908	3.756874280924
H	0.032282867706	1.734535394864	3.942102762109
H	-0.839212645625	3.138425575256	4.615264784292
H	0.672367456473	3.383846442892	3.704241024053
C	-1.652204787870	4.696103911767	1.808191844313
H	-0.738624727500	5.292169129897	1.659479999236
H	-2.211023288713	5.134004709739	2.650630514985
H	-2.267329650426	4.798238876863	0.901428057018
C	-3.428927775609	-1.146415839039	-0.212529436333
C	-3.305230021813	-1.750015201868	1.055203626368
C	-4.265677092074	-1.468954879874	2.028412262080
H	-4.165380844097	-1.936655288065	3.012461193759
C	-5.337739264660	-0.607056082655	1.788378592341
C	-5.441914557299	-0.032412224906	0.521693393181
H	-6.275724999666	0.643099232916	0.307145064106
C	-4.508477712081	-0.285889515704	-0.487052939109
C	-2.152694359232	-2.655832779020	1.369510894020
H	-2.341323152886	-3.221642568723	2.292384719681
H	-1.231930718579	-2.069713315870	1.528242712423
H	-1.956819862076	-3.368079600671	0.555868180779
C	-6.316024754322	-0.271751372726	2.875483228684
H	-6.475670228984	-1.122988880770	3.553489627559
H	-7.291154904225	0.028849418429	2.465811579305
H	-5.943101019648	0.567398737820	3.487040175119
C	-4.683763707630	0.332563780479	-1.842414306434
H	-5.255197814552	1.269112468259	-1.778365433992
H	-5.236145481093	-0.341001741894	-2.519152340211

H	-3.718229879585	0.540229906280	-2.319911099346
C	-4.480909109229	-3.380306320692	-2.102359165331
H	-4.505513211190	-3.806597782631	-1.087666204115
H	-4.709330658721	-4.186438505438	-2.817624061805
H	-5.279952081597	-2.627834279561	-2.166521688922
C	-2.721536824991	-1.872875138241	-4.175438118582
H	-3.511426515647	-1.117707204572	-4.301485440536
H	-2.841477265192	-2.631854346324	-4.964928351821
H	-1.750772363210	-1.376035028467	-4.332222579483
C	-1.505128552108	-4.032399223289	-2.378063865811
H	-0.474895372308	-3.682360248042	-2.546309486215
H	-1.721326014566	-4.795764551966	-3.143726147313
H	-1.533450398722	-4.532206352900	-1.397284309040
H	0.691028145457	-0.425919804332	2.509544775622
Si	-0.929517260199	-0.611388384266	-1.130400481190
H	0.907607486959	-0.691698454230	-3.915192033767
H	1.788236025700	-2.676960440476	-2.186276594451
O	1.957240773752	-3.341303316314	-2.869980129882
H	1.705272322101	-2.882497918639	-3.678667428806

114

H2O_TS_4''', G=-3822.176728

Si	0.771321017885	0.259397247537	-2.418482962465
Si	2.137696551155	0.405118520545	-0.482216821504
Si	0.110421497879	0.508320840033	0.768871225773
Si	4.794426049862	-1.162191549828	-0.691181756819
Si	-1.380123699807	2.763481546304	2.349845884675
Si	-2.633429356101	-2.615264978987	-2.529047351639
O	3.261895802242	1.657138625377	-0.586369250904
H	2.883684684084	2.495403351796	-0.889663271144
O	-0.101477258715	-0.130766421825	2.308479532079
N	3.148975192234	-0.974752830919	-0.051185376155
N	-0.384864938485	2.183327625480	0.994238305087
N	-2.334559153427	-1.396873491754	-1.265238237590
C	2.691407578092	-1.757042977408	1.047842472977
C	2.765092727410	-1.248537591393	2.364936426911
C	2.234014293328	-1.998687505278	3.422194698302
H	2.290124072536	-1.585881931512	4.434325803062
C	1.625707936096	-3.239489974171	3.224057929495
C	1.591731816125	-3.738427659006	1.921027234581
H	1.133058088195	-4.714567665281	1.734765979352
C	2.117553461720	-3.032665463266	0.836839282441
C	3.340367721998	0.104038666456	2.672199110094
H	3.778753819174	0.585910410634	1.790961848258
H	4.117288210961	0.030588627144	3.448972027496
H	2.553031401683	0.767902112576	3.064769763937
C	1.004024262400	-3.997089757529	4.360568210177
H	1.209603504914	-5.075366948079	4.286174691493
H	-0.092091894428	-3.875018558187	4.360636932432
H	1.375132726963	-3.643507533598	5.332885160266
C	2.063523266103	-3.667335699517	-0.519427688457
H	1.036128878041	-3.962314330384	-0.774727258838
H	2.688216648390	-4.574358234140	-0.550505257393
H	2.424411115604	-2.975583114674	-1.286619024864
C	4.692386805492	-0.861689245854	-2.548730756654
H	4.191486592814	-1.696048653517	-3.069430467789
H	5.699418151186	-0.777782830239	-2.986812734560
H	4.161160434929	0.078743342585	-2.766309013829
C	5.401115774774	-2.902948477646	-0.336663207782
H	5.290591164584	-3.145602572526	0.731896224279
H	6.471402614857	-2.974088338977	-0.588156363796
H	4.864313390087	-3.666858399750	-0.916490996431
C	5.995167883078	0.055262143950	0.079605843048

H	5.623937999481	1.080771074158	-0.059894713619
H	6.991392105420	-0.033528165719	-0.383174693191
H	6.106578743168	-0.132236246958	1.158939421018
C	0.091462056939	3.076047930796	-0.003002954187
C	-0.625215970669	3.257803096338	-1.205856045149
C	-0.076565581031	4.052931832199	-2.217080272065
H	-0.633606988565	4.167791045524	-3.151927216432
C	1.145471432000	4.709288955037	-2.065844757671
C	1.806527827567	4.575512359392	-0.841245907848
H	2.745952597147	5.113642532679	-0.678276130155
C	1.302393924167	3.776168172098	0.191450730638
C	-1.992690180296	2.674236580167	-1.376737988438
H	-2.753319715888	3.356950509234	-0.960263399840
H	-2.100577237987	1.719572433928	-0.847859620658
H	-2.229814950494	2.522315366655	-2.437754818780
C	1.733976009829	5.518901740261	-3.184222535088
H	0.955909764657	6.061164605646	-3.741625591578
H	2.257893046446	4.871001544684	-3.907041215505
H	2.463043996258	6.252798472262	-2.812021641978
C	2.039388256627	3.674952267152	1.491792154798
H	3.073514379150	4.030838225997	1.392772523866
H	2.058018117102	2.641396068261	1.860313000689
H	1.545168942521	4.281836562037	2.267562063689
C	-2.939850239029	1.737523448800	2.444777219249
H	-3.501585650664	1.756075780645	1.498622836254
H	-3.602697592562	2.096428669992	3.248651609017
H	-2.685834928502	0.688548744437	2.656702056133
C	-0.472938340689	2.635759082664	3.991164834437
H	-0.171309841506	1.590047509393	4.150174897119
H	-1.134669384351	2.938792111844	4.818849744870
H	0.426189875062	3.268510018756	4.025160354079
C	-1.776009720387	4.564083664709	1.980883673291
H	-0.863197563885	5.172152327114	1.886061320084
H	-2.381552391445	4.986771171234	2.798525136559
H	-2.346395939271	4.670999068811	1.045760913659
C	-3.395730790193	-1.033296164566	-0.386195211334
C	-3.469095876139	-1.618255807040	0.897270256678
C	-4.531055073269	-1.283581881442	1.736752798525
H	-4.580001703391	-1.741595182277	2.729462857809
C	-5.524191095022	-0.378778427943	1.353191091339
C	-5.438453065872	0.177710645796	0.078060313541
H	-6.208647658253	0.882723458874	-0.250575296889
C	-4.397902943287	-0.136229291472	-0.802674913715
C	-2.422497669539	-2.592231342676	1.350613084975
H	-2.706174533821	-3.057756700057	2.304804258017
H	-1.452380363116	-2.092082059630	1.501522133514
H	-2.259974591058	-3.386657430653	0.607274553020
C	-6.616188906057	0.014929478788	2.303980695125
H	-6.933878245175	-0.832744308461	2.929309336906
H	-7.498971715968	0.398493030940	1.772278506938
H	-6.270140711165	0.810471320205	2.985769084580
C	-4.391088003311	0.446557350912	-2.184810488839
H	-4.829580595641	1.454683308821	-2.192870528486
H	-4.986249628034	-0.171493115784	-2.878325532399
H	-3.373651475946	0.497443586737	-2.589897259128
C	-4.398961412146	-3.219008050332	-2.337124547101
H	-4.557272298238	-3.635813142783	-1.330344612732
H	-4.601647444463	-4.012820796851	-3.073591446725
H	-5.138695984176	-2.419034154414	-2.482870148722
C	-2.322877503056	-1.885136264123	-4.232305924433
H	-2.982497005878	-1.030784150660	-4.443166562489
H	-2.485630349528	-2.646254048647	-5.012512853095
H	-1.278629443448	-1.543505656290	-4.315040198788

C	-1.475540229284	-4.089208400882	-2.337038706101
H	-0.434663065718	-3.818793923355	-2.567013058052
H	-1.774883919345	-4.885231476704	-3.038455856087
H	-1.507286304775	-4.503869493971	-1.317845579191
H	0.459238935218	-0.888809729492	2.526196210820
Si	-0.689780176223	-0.818485317852	-0.988866882459
H	0.969295556447	1.094358372179	-3.647694018966
H	0.642521560670	-1.748802986199	-2.099044303341
O	1.204120292949	-1.422804577442	-3.109554378041
H	2.153672612947	-1.624455926892	-3.093826260634

114
2''', G=-3822.257573

Si	0.870337493277	-0.017804980787	-2.376810277807
Si	2.168719732027	0.380406466148	-0.412792760367
Si	0.113562434682	0.522764823007	0.864807051822
Si	4.891806565253	-1.031497326349	-0.561470841609
Si	-1.459855756370	2.714736074760	2.411365995875
Si	-2.610970380633	-2.244694429632	-2.770278496383
O	3.202760927267	1.701471486803	-0.516074210915
H	2.782407525073	2.522411379598	-0.810249387926
O	-0.069487896054	-0.186302999099	2.373207038090
N	3.229974289394	-0.934275931990	0.065125361786
N	-0.375245097624	2.183679143290	1.102503108158
N	-2.395321246083	-1.223347492185	-1.324694929113
C	2.711672380222	-1.866726628512	1.005453431184
C	2.695938067048	-1.528461771278	2.377599842229
C	2.100779233325	-2.402395413319	3.293300878405
H	2.089053660208	-2.127038377585	4.352449154350
C	1.526044636849	-3.613329023645	2.891782100074
C	1.591752052758	-3.944756894740	1.539040305528
H	1.157379814916	-4.890373646543	1.200730840061
C	2.175727577256	-3.102420239452	0.586620309181
C	3.289215018269	-0.233159109103	2.842485258491
H	3.193008069072	-0.121915741493	3.930820861171
H	2.796644330064	0.631074602032	2.368617066569
H	4.352828599573	-0.159700520732	2.573578515729
C	0.832030884902	-4.506352909835	3.878335123068
H	1.255468520720	-4.400845602131	4.887690658474
H	0.902684251254	-5.563678998988	3.584736045407
H	-0.240530698746	-4.256961166633	3.945476984555
C	2.214059184778	-3.531530524851	-0.849378800561
H	1.362338791786	-4.183483528835	-1.086798643289
H	3.131530932826	-4.103951352984	-1.061903820934
H	2.190484732863	-2.678013093326	-1.537968514715
C	4.792379781832	-0.760360900031	-2.420341641699
H	4.182514466388	-1.548340418952	-2.888888239577
H	5.791690617114	-0.775275260094	-2.882612543149
H	4.339211681409	0.221981248207	-2.630717506811
C	5.599002702019	-2.718437998794	-0.143495597576
H	5.468483986986	-2.940527462222	0.927326986033
H	6.679572527096	-2.721856100563	-0.358201963381
H	5.136043668014	-3.532542435624	-0.718162437102
C	5.999924262233	0.279689387594	0.198860002161
H	5.510616176456	1.261565896003	0.127194223780
H	6.966530617703	0.321618150168	-0.329044433326
H	6.207258046648	0.066058675418	1.259081202763
C	0.115152303342	3.084438042324	0.117517429043
C	-0.536329363209	3.231495268278	-1.122291559398
C	0.020166970564	4.075286401394	-2.093632634017
H	-0.489553333998	4.168568899669	-3.057347355408
C	1.190302761034	4.793790013497	-1.871668827303
C	1.807964487855	4.661063044138	-0.620247547004

H	2.722622101967	5.225127700319	-0.411461265911
C	1.293088325197	3.828746450519	0.374263206908
C	-1.824600705229	2.529379437453	-1.427003024990
H	-2.619161013910	3.256945398541	-1.656990629865
H	-2.162267218755	1.908980385619	-0.588105412792
H	-1.716431903421	1.881980592885	-2.311074022852
C	1.772945391693	5.695035589137	-2.920706053914
H	1.747152021470	6.748496675051	-2.598180146744
H	1.220609407429	5.620347664000	-3.867659068485
H	2.826775253033	5.446374463179	-3.122050084987
C	1.987712901399	3.716521449337	1.696157252722
H	3.014729568661	4.100658052969	1.638465212641
H	2.019369511957	2.674832347417	2.042579058707
H	1.453496119261	4.290183330932	2.470428470942
C	-2.943877845321	1.576530462676	2.462510834653
H	-3.487980401341	1.551500648111	1.505946498153
H	-3.649860779480	1.891361441254	3.247952536336
H	-2.624705313159	0.548375751432	2.689546654666
C	-0.597520998408	2.648519180509	4.078692663017
H	-0.184860393728	1.638352873242	4.221284394582
H	-1.313226592053	2.849610209396	4.892133395680
H	0.223788924385	3.375466220960	4.159736383112
C	-1.960557083005	4.473798507642	1.986671449345
H	-1.086202839864	5.138484682238	1.909613172956
H	-2.626686586754	4.876265665498	2.766246579565
H	-2.496192285229	4.512800286172	1.025647402881
C	-3.463718871555	-1.016274849759	-0.404819013756
C	-3.490987326699	-1.711652144278	0.825462275943
C	-4.547292263244	-1.493255951775	1.710481956923
H	-4.553765459165	-2.036740090719	2.660401051283
C	-5.587984846493	-0.608028821732	1.424844918193
C	-5.555252087854	0.050942573185	0.197088858148
H	-6.362316587214	0.744583592439	-0.059018641658
C	-4.519839500969	-0.137239866268	-0.723724822482
C	-2.402466009345	-2.675699575996	1.195417884658
H	-2.727411931556	-3.335411804020	2.012220063899
H	-1.500531099193	-2.145083661448	1.541865918750
H	-2.100211469746	-3.297397333262	0.340382824312
C	-6.676860189777	-0.345693832270	2.423454765238
H	-6.929061813848	-1.251581254460	2.994568193562
H	-7.593499169471	0.019353808621	1.937954994743
H	-6.362503262124	0.420996701709	3.152039104911
C	-4.571485474218	0.578539918997	-2.040345581348
H	-5.080883054616	1.547998265318	-1.943869631752
H	-5.128427677431	-0.006608182191	-2.791559847161
H	-3.566284845923	0.743049734306	-2.442521703188
C	-4.296327335793	-3.056141912580	-2.641137513146
H	-4.369383124423	-3.642553874419	-1.712099863181
H	-4.450175857434	-3.738653574539	-3.491933118841
H	-5.117110997301	-2.324492904916	-2.635633761877
C	-2.432625320469	-1.243253282824	-4.351416757265
H	-3.171414191869	-0.433616222909	-4.433712725270
H	-2.550197093474	-1.904033048042	-5.225723404700
H	-1.423188766759	-0.805306541829	-4.408566451448
C	-1.263185036337	-3.553669081847	-2.803920553313
H	-0.271145400649	-3.097370947115	-2.951168875238
H	-1.440075834083	-4.243273698349	-3.645585492384
H	-1.243560743477	-4.144531791813	-1.875097223637
H	0.506756984019	-0.945520644529	2.548711512649
Si	-0.755195864945	-0.776996323765	-0.887537141220
H	0.599366056516	1.224691332172	-3.174825388992
H	0.064062271440	-1.970831700981	-0.453020778598
O	1.366303907409	-1.252797623280	-3.426648080587

H	2.204506261078	-1.090352851922	-3.871520664990

146
MeOH_Cluster_Int_1, G=-4521.554649

Si	-1.064072544570	1.171417409794	-0.262729279476
Si	0.901548344164	0.233986531464	0.477774345320
Si	-0.020377966372	-0.670084084266	-1.404997559675
Si	-2.354611271318	-0.525233058086	-1.255995544663
Si	-3.039581537165	3.346412671181	-1.184569977781
Si	1.348984852819	0.163475006660	3.587236081017
Si	0.372322017045	-2.088740675047	-4.143535148543
Si	-4.760358131015	-2.291179559746	-0.897732874413
N	-1.600485387122	2.852677209366	-0.236910155373
N	1.788338587238	-0.267651157658	1.911560971478
N	0.982836559925	-1.116154545001	-2.788895762108
N	-3.249682203327	-1.678705845490	-0.185097935656
C	-0.840422510588	3.877649300925	0.388223539794
C	0.180386326598	4.539970303464	-0.319140951362
C	0.894337463773	5.568138911889	0.305086409054
H	1.682726183208	6.079512162008	-0.256816908529
C	0.633160223276	5.955501945995	1.619630822990
C	-0.397957213471	5.298968315154	2.297614414898
H	-0.639118828811	5.598348410123	3.322482675756
C	-1.150710305798	4.283969443997	1.703994575047
C	0.487977677348	4.144431798085	-1.731688243587
H	1.423382537489	4.603634581635	-2.083229793691
H	-0.313961811249	4.454621001570	-2.420035182975
H	0.564215123448	3.050960397791	-1.830073956577
C	1.423242956742	7.043067502261	2.288798649759
H	0.764171493126	7.829969322423	2.687962853641
H	2.128627132591	7.516765981639	1.591446803720
H	2.004730167964	6.650417106390	3.138928605446
C	-2.315077115244	3.682406223586	2.430336757656
H	-2.250227100176	3.868368874535	3.511171159408
H	-2.388750538401	2.601855991058	2.260454778094
H	-3.261253293965	4.121655724314	2.072017364987
C	-3.148782090070	5.219431303447	-1.055419158567
H	-3.261121700624	5.553572402611	-0.012841934676
H	-4.028115530931	5.567910655731	-1.620823011663
H	-2.259125400656	5.718243724886	-1.468368149659
C	-4.603723983774	2.589571846999	-0.482419479028
H	-4.563186712119	1.493190833790	-0.537811058969
H	-5.476606984707	2.923372880299	-1.066509633449
H	-4.759722198972	2.879110666253	0.567486247756
C	-2.858944974685	2.848781924127	-2.986294059153
H	-1.988454258238	3.327127742037	-3.460290024955
H	-3.759689830151	3.141109332763	-3.550353745434
H	-2.745539455127	1.755420782887	-3.067604808576
C	2.738464075157	-1.338861022126	1.764065371515
C	2.281144871673	-2.676252292081	1.708833671023
C	3.206198043974	-3.720647856207	1.664162967901
H	2.828143920855	-4.747444300817	1.635775267886
C	4.582988943233	-3.498330284749	1.647894435232
C	5.015687118626	-2.174378757040	1.667974953388
H	6.089613245188	-1.963545983641	1.649390623196
C	4.127768266899	-1.093675953615	1.722034064797
C	0.826465995258	-3.014537303837	1.649980754095
H	0.510979444950	-3.181151580091	0.607400721833
H	0.179598346982	-2.222744412761	2.039289638629
H	0.613487650816	-3.937475749830	2.205572399289
C	5.552024950598	-4.642699667026	1.582526990392
H	6.583680386555	-4.309640183110	1.764192498896
H	5.527960365009	-5.127841302470	0.592532529792

H	5.308619545992	-5.417784202420	2.325356036812
C	4.706242821275	0.287599783750	1.685477153211
H	5.705894246289	0.307114138201	2.142289225441
H	4.073724301541	1.012028298199	2.204377076850
H	4.809001662961	0.620658567047	0.640303466247
C	-0.171596459925	1.247821540612	3.537315136649
H	-0.006957418915	2.169181995813	2.958821695259
H	-0.441663539185	1.538118256781	4.565767282999
H	-1.024024003584	0.708774050301	3.097564743062
C	1.020264503625	-1.380485295775	4.599015348041
H	0.100465275554	-1.884407903086	4.269439094065
H	0.897502628992	-1.102781612406	5.658284219741
H	1.854267072610	-2.095663183988	4.530471118323
C	2.743885023591	1.106282714845	4.432992932048
H	3.669289450961	0.512525569472	4.482076640975
H	2.440966461063	1.336401697139	5.467715383909
H	2.969537697386	2.062908706529	3.935837328959
C	2.260589956872	-0.507683233513	-2.766950824722
C	3.347915633992	-1.171414418807	-2.157741426012
C	4.565203701434	-0.501739336033	-2.021960479334
H	5.390709122114	-1.019060434880	-1.523917924564
C	4.748354691357	0.809047836004	-2.473720887360
C	3.670957229060	1.439086017225	-3.105083237362
H	3.793187903446	2.457055828582	-3.488087273794
C	2.432185822545	0.801030017955	-3.273416818479
C	3.180079694001	-2.569837787836	-1.656411010798
H	4.041874971861	-2.884058032709	-1.054515047990
H	3.057659113664	-3.275837375529	-2.493592660063
H	2.278783582644	-2.658516049008	-1.034574553674
C	6.066834705832	1.505380926039	-2.298248632243
H	5.993234282808	2.576678652185	-2.533403964991
H	6.835324491400	1.072878828489	-2.959460561286
H	6.440341540228	1.406184585794	-1.266873224502
C	1.311865280913	1.477536691328	-4.004481264352
H	1.523137752682	2.543574847941	-4.162457519007
H	0.357142543717	1.375882602567	-3.465710738426
H	1.166467156716	1.015027938558	-4.993911606284
C	1.820993010288	-2.346218715249	-5.310805873758
H	2.211257893176	-1.384087928955	-5.678005815561
H	1.503981865454	-2.937288630838	-6.184549209931
H	2.651451006007	-2.878725418783	-4.822698297791
C	-0.263548765272	-3.716960043228	-3.463324288820
H	0.502002338931	-4.232242152142	-2.863346211976
H	-0.580014440455	-4.389028640939	-4.276925103814
H	-1.139846698985	-3.536884763378	-2.821161556608
C	-1.039363299756	-1.221906723819	-5.024383357716
H	-1.811218883004	-0.935885676912	-4.290207982481
H	-1.500953248753	-1.892810405023	-5.766981620164
H	-0.710164025343	-0.310234320294	-5.544994533926
C	-2.899453186150	-2.113965962144	1.115428748891
C	-3.148650014275	-1.293227188711	2.235672209980
C	-2.854942000701	-1.768252710870	3.517261363531
H	-3.051282346291	-1.117740169716	4.375637133294
C	-2.358861665101	-3.054690711469	3.736646870084
C	-2.128236190760	-3.856156892919	2.617020743678
H	-1.729507320534	-4.866311311313	2.756914655696
C	-2.364929632829	-3.404476675638	1.317764556039
C	-3.786974630276	0.048656266478	2.056664725548
H	-4.798471969102	-0.048010275341	1.630683724847
H	-3.219997421200	0.670338862970	1.347283193211
H	-3.866846718290	0.585844321282	3.012165323980
C	-2.100427180885	-3.570807785806	5.123435737705
H	-2.120571715967	-2.759249788073	5.864968084993

H	-1.119926519774	-4.067163923588	5.194804009191
H	-2.860518738593	-4.311894540818	5.422161279169
C	-2.053066277638	-4.282269895006	0.144008533079
H	-1.359322120055	-5.087694550674	0.423362908368
H	-1.608711656847	-3.692548272389	-0.670372441479
H	-2.958660717224	-4.756619005714	-0.267635054426
C	-5.927992186356	-0.882534896746	-1.344784433226
H	-6.214817798927	-0.297386843165	-0.457593492377
H	-6.848047531650	-1.285639245562	-1.799457722012
H	-5.462060060621	-0.197484531343	-2.069574374136
C	-5.619431684119	-3.374560131196	0.375794534894
H	-5.028845072366	-4.259175034193	0.654469681798
H	-6.587144824767	-3.717684984871	-0.024094160526
H	-5.812958486059	-2.804807399823	1.298167103475
C	-4.425652548317	-3.247054083024	-2.487115895019
H	-3.915616556286	-2.594157563725	-3.213683772347
H	-5.369097556988	-3.589306722274	-2.942909725860
H	-3.786533269416	-4.126293333125	-2.318503300697
O	2.372954945088	1.328816726795	-0.213149332636
H	2.475710028863	1.309871342501	-1.190680172413
C	2.815974378624	2.551879182330	0.366698334513
H	3.884976957818	2.687862279576	0.157354838199
H	2.229343774009	3.397404621834	-0.005426829139
H	2.662141784870	2.464865310829	1.448189584694

152
MeOH_Cluster_Int_2, G=-4637.020157

Si	-1.739408598010	0.005822505192	-0.519630024691
Si	0.188455958497	-1.172288379032	-0.116840957382
Si	0.524183914881	0.832103989684	-1.134748700065
Si	-1.250925045114	2.312538005675	-0.738472421929
Si	-4.705939487426	0.311145142655	-1.359200219866
Si	-0.065551656580	-3.089549382705	2.293935404858
Si	2.108256928452	2.496209797104	-3.229645046720
Si	-1.857613219404	4.961601610675	0.624779729838
N	-3.363479684505	-0.679215638711	-0.703102732177
N	0.929391972517	-2.194894954680	1.100049373821
N	1.932939976275	0.999913185451	-2.242306864091
N	-1.179385770776	3.322616801856	0.775309015121
C	-3.644275162090	-2.049565903154	-0.437782013465
C	-3.447317205103	-3.014798850914	-1.446831252707
C	-3.745158507678	-4.355117264971	-1.193394878598
H	-3.584569354566	-5.091235584166	-1.987619156556
C	-4.252568402505	-4.776032148457	0.037966704050
C	-4.486140681927	-3.800842870978	1.007527453414
H	-4.909082022955	-4.098701829731	1.971970088822
C	-4.205063119981	-2.448305588016	0.793146089337
C	-2.970907530498	-2.578239090221	-2.796126836206
H	-2.718453605825	-3.435897122235	-3.435784349144
H	-3.741733125624	-1.986398031303	-3.315384534776
H	-2.096219907651	-1.922035315393	-2.705652594841
C	-4.501738445292	-6.228122765107	0.320256063220
H	-5.321043806990	-6.365742898648	1.040835615183
H	-4.754058361323	-6.782141630886	-0.595737806691
H	-3.603102168683	-6.700826511854	0.752391906836
C	-4.578532739253	-1.440222759761	1.836059659701
H	-4.598958406226	-1.890196917512	2.838030434197
H	-3.893110682285	-0.586158999575	1.844975980344
H	-5.585220487307	-1.035676254440	1.635954999915
C	-6.164453661129	-0.842925375289	-1.632564158912
H	-6.499120574446	-1.310735978576	-0.694521897573
H	-7.007280683451	-0.263561777467	-2.042951001046
H	-5.931671798582	-1.652411941368	-2.340596942551

C	-5.193875044019	1.655647070644	-0.151940923258
H	-4.329866143816	2.294686358662	0.072153134857
H	-5.979027607269	2.294232948048	-0.587274670121
H	-5.570510399410	1.239601867685	0.793810268874
C	-4.214488428371	1.101739602740	-2.990051550496
H	-4.008069612878	0.348078734585	-3.765186353431
H	-5.030224804200	1.747730821193	-3.353409116545
H	-3.318604435856	1.729512962246	-2.852586190958
C	2.319142929098	-2.029944109148	1.435369728485
C	2.705510378606	-1.036147403405	2.356158962864
C	4.036431813261	-0.971226921542	2.777560554308
H	4.315422359386	-0.196535185107	3.498825186987
C	5.003837194293	-1.868938450897	2.329455282855
C	4.603661125967	-2.833491701734	1.405650143859
H	5.336474423530	-3.555130087643	1.032387648969
C	3.291264607645	-2.920069638884	0.937807868085
C	1.715471513837	-0.083698802055	2.944746808203
H	2.220145797829	0.804465902308	3.340816760273
H	0.958161956690	0.258390970192	2.224371761325
H	1.168157283261	-0.543471907984	3.783190861562
C	6.428883959755	-1.770898192958	2.788766161447
H	6.495052405320	-1.426527607043	3.831332220160
H	6.945000165956	-2.739201883056	2.714320196350
H	6.996515753039	-1.052654134350	2.172373594583
C	2.946439072313	-3.971803120412	-0.069333630100
H	2.624042243778	-3.509526020045	-1.013731829639
H	3.816320273724	-4.607038508867	-0.284085392720
H	2.130433619487	-4.620341249594	0.285553003679
C	-1.335112915423	-1.968314539221	3.099381529320
H	-1.914825556921	-1.442652035500	2.328131605632
H	-2.038748613169	-2.562569911175	3.703977924877
H	-0.875727603111	-1.205067459171	3.742662087767
C	1.101473224246	-3.809579749356	3.572643068226
H	1.644460500130	-3.028015857134	4.124108634179
H	0.523654219460	-4.405900833121	4.297056530126
H	1.851782598649	-4.466502080825	3.106739670713
C	-0.990741024327	-4.489701303119	1.451449607777
H	-0.306465121434	-5.243086183378	1.031076206715
H	-1.632576350061	-4.995064598573	2.191188963912
H	-1.653019470063	-4.123974860695	0.653008610886
C	3.153318936929	0.310010638905	-1.919038300330
C	3.837828048437	0.622956496186	-0.720414076117
C	5.064368974870	0.020285842776	-0.447413187556
H	5.579791711856	0.291606470166	0.477055633098
C	5.656342931026	-0.892172366715	-1.321204070270
C	4.969832884005	-1.188639132928	-2.494770048728
H	5.417420705254	-1.884366730927	-3.211419110872
C	3.739240729081	-0.605505647952	-2.818443976313
C	3.305956716661	1.648464069331	0.231408157410
H	3.925638158290	1.700020499976	1.136198776349
H	3.282725446384	2.647095220386	-0.227269001056
H	2.275647765981	1.431377765325	0.560667989148
C	6.980466990834	-1.522849235707	-1.007664671865
H	6.865225119642	-2.344379002069	-0.282145485669
H	7.453088611579	-1.939323645588	-1.908757764949
H	7.676546984387	-0.795881046516	-0.562347811740
C	3.125134770474	-0.971345084760	-4.138423997482
H	2.404764289189	-1.796921329468	-4.042139559000
H	2.582024135115	-0.135927319893	-4.598921052765
H	3.904243996085	-1.292622386842	-4.843930283671
C	3.818512660194	2.503832476139	-4.002110491571
H	3.990423009736	1.668212539916	-4.694755559795
H	3.946207207716	3.444080323755	-4.562687433113

H	4.597539465156	2.461648792937	-3.225402424216
C	1.919625579185	4.077898707960	-2.240347550231
H	2.741560968079	4.232708979804	-1.526470305419
H	1.914748892669	4.933030873592	-2.935916121201
H	0.966917497067	4.090439370885	-1.692209724727
C	0.769299926996	2.498721529236	-4.544836785333
H	-0.225580902529	2.412922221851	-4.078194679333
H	0.794384913053	3.450762279585	-5.098596015523
H	0.898872004279	1.686036271237	-5.275694889556
C	-0.640037108960	2.961689460295	2.035791122549
C	-1.414332641277	2.218137789607	2.952312847499
C	-0.905664847499	1.935564852206	4.221634623722
H	-1.524900661695	1.361531728764	4.918560861441
C	0.347407248510	2.390200508091	4.635960132791
C	1.095746639608	3.135169482477	3.724121320904
H	2.081471490716	3.509164127652	4.020564378570
C	0.634574614266	3.418102230880	2.436155805712
C	-2.804057711885	1.803583896400	2.589525708539
H	-3.457964052216	2.680398208086	2.455337907978
H	-2.818460720093	1.266353172361	1.629696718829
H	-3.245300630783	1.160665676363	3.363406462314
C	0.892784324421	2.045287540819	5.990783279709
H	1.497101275817	1.121809961112	5.951082177748
H	1.544000659151	2.841777913637	6.380589858952
H	0.087437056942	1.875116408420	6.720384804622
C	1.469660651476	4.249869050309	1.512743112286
H	2.534296411184	4.193797016222	1.779714781760
H	1.341869940269	3.928074355883	0.473175484935
H	1.177515975901	5.312528596791	1.554041540742
C	-3.652654762074	4.941718486043	0.055979434503
H	-4.312645259493	4.452049565535	0.787515471686
H	-4.004383878516	5.978116169390	-0.078266400352
H	-3.759771257813	4.420124938587	-0.907391487859
C	-1.799645807121	5.775855785078	2.319012661051
H	-0.774848060601	5.898178318995	2.698549584654
H	-2.268348732463	6.771648022926	2.264848022879
H	-2.353195571367	5.176499055797	3.058590599947
C	-0.930558483700	6.009178310963	-0.637850025910
H	-1.006901593704	5.552495039584	-1.637210041597
H	-1.378607025231	7.015057382108	-0.692266468434
H	0.136135833615	6.121451801443	-0.396645267403
O	0.366549526164	-2.333032188954	-1.644191677487
C	0.033028880609	-3.713360561496	-1.744521524229
H	0.185297157186	-1.824193199794	-2.510952837479
H	0.476115317824	-4.225348470462	-0.888367521107
H	0.462593410724	-4.111600616159	-2.674218268710
H	-1.053981697074	-3.860018145247	-1.728225812925
C	-0.936242512484	-0.508075103971	-4.521018676368
H	-1.502432322109	-1.380459119326	-4.870351287707
H	-1.591587515642	0.123836501557	-3.899350569854
H	-0.611258924816	0.066056121057	-5.401312123535
O	0.175981130154	-0.977388382509	-3.790559611621
H	0.717213155868	-0.222968146794	-3.469155334337

152

MeOH_Cluster_TS_1, G=-4637.019285

Si	-1.704924015964	-0.013989068817	-0.533822968750
Si	0.159560037808	-1.311540991549	-0.088509968750
Si	0.540946954836	0.690601024256	-1.129044968750
Si	-1.135307111115	2.282028954789	-0.750641968750
Si	-4.559048033384	0.406350812904	-1.636546968750
Si	-0.248195896546	-2.895600008447	2.532010031250
Si	1.977542889427	2.268942083791	-3.377924968750

Si	-1.623148224342	5.014228934572	0.455809031250
N	-3.342330990781	-0.621669136674	-0.820616968750
N	0.817492072546	-2.149783964284	1.306678031250
N	1.914327953482	0.723290081171	-2.404700968750
N	-1.036212155483	3.352640958896	0.717645031250
C	-3.711543935320	-1.959972151974	-0.508347968750
C	-3.497975892782	-2.986417143124	-1.450359968750
C	-3.870365838473	-4.296901158556	-1.143455968750
H	-3.691407805869	-5.083653151140	-1.883196968750
C	-4.465350824811	-4.626570183213	0.076092031250
C	-4.711823867778	-3.589778193428	0.976782031250
H	-5.199878858399	-3.816096213653	1.929806031250
C	-4.358865922691	-2.264701178800	0.707049031250
C	-2.901196906720	-2.650101118392	-2.780774968750
H	-2.639232869177	-3.556021107536	-3.345188968750
H	-3.600349931218	-2.058947147366	-3.393744968750
H	-2.004720932411	-2.030166081241	-2.651428968750
C	-4.794257765865	-6.048969196844	0.422077031250
H	-5.681098763197	-6.113334233596	1.069452031250
H	-4.982440740861	-6.652317204642	-0.477944968750
H	-3.958302746286	-6.521402162200	0.965658031250
C	-4.735927967461	-1.184394194426	1.673543031250
H	-4.841824951084	-1.579578198815	2.693148031250
H	-4.003991001225	-0.369662164094	1.683848031250
H	-5.703234986072	-0.735290234513	1.390950031250
C	-6.055122988504	-0.676616249096	-1.986305968750
H	-6.487744971762	-1.080603267025	-1.058414968750
H	-6.829022013525	-0.072846281168	-2.487227968750
H	-5.814345953133	-1.530120239118	-2.637541968750
C	-5.072921092344	1.829071791608	-0.533038968750
H	-4.193086116601	2.414408828070	-0.234373968750
H	-5.760336120345	2.504750763120	-1.066573968750
H	-5.576301077536	1.471750770747	0.377456031250
C	-3.856561061075	1.074533842016	-3.245444968750
H	-3.605755027453	0.263224852410	-3.946329968750
H	-4.585973088569	1.737986811788	-3.737567968750
H	-2.946265085404	1.661608879740	-3.039268968750
C	2.207984066761	-2.010182906659	1.636711031250
C	2.623287023508	-0.966466889448	2.487565031250
C	3.959727020606	-0.896443834064	2.889373031250
H	4.259512986904	-0.083205821640	3.558180031250
C	4.908949059442	-1.833557794727	2.482458031250
C	4.481489101549	-2.849632812441	1.628858031250
H	5.198586132865	-3.605290782724	1.293710031250
C	3.158687105712	-2.950067867261	1.191471031250
C	1.651418981789	0.040231070276	3.010176031250
H	2.171137944749	0.934003091814	3.372478031250
H	0.916537968614	0.358148039821	2.256295031250
H	1.075395998462	-0.362093953596	3.858753031250
C	6.341422055048	-1.727546735363	2.917400031250
H	6.423319040350	-1.372861731969	3.955586031250
H	6.858117095166	-2.695602713950	2.845061031250
H	6.898790025470	-1.013803712264	2.286528031250
C	2.776339152047	-4.068149883106	0.273675031250
H	2.472538135771	-3.675408895696	-0.707791968750
H	3.618404180583	-4.756743848209	0.121058031250
H	1.928609175885	-4.643370918237	0.676565031250
C	-1.505876947385	-1.668831060568	3.190483031250
H	-2.075667965159	-1.239933084181	2.354149031250
H	-2.220023926381	-2.175652090163	3.859368031250
H	-1.043665982001	-0.833528041413	3.734486031250
C	0.850893130479	-3.547702962899	3.905203031250
H	1.408118097136	-2.743143939807	4.407157031250

H	0.231715151730	-4.060514988559	4.658595031250
H	1.587990160318	-4.267730932353	3.517646031250
C	-1.209253837292	-4.325415048275	1.781058031250
H	-0.546158805044	-5.103559020795	1.373296031250
H	-1.836384818092	-4.788699074265	2.560424031250
H	-1.889884851345	-3.986296076482	0.985744031250
C	3.198695976837	0.159714134397	-2.032905968750
C	3.806437963332	0.485599159583	-0.794911968750
C	5.021784987744	-0.103463790051	-0.450770968750
H	5.464164976866	0.159010228282	0.512715031250
C	5.687371024740	-0.996206762468	-1.290335968750
C	5.103037035711	-1.260938786683	-2.523140968750
H	5.617881063214	-1.924580765347	-3.224997968750
C	3.884950012357	-0.697396837163	-2.922684968750
C	3.235463921129	1.503974135921	0.142899031250
H	3.797973920629	1.516039159233	1.085052031250
H	3.269466879315	2.512951137330	-0.288589968750
H	2.182377929058	1.312642092280	0.413823031250
C	6.978915051234	-1.635502708944	-0.876677968750
H	6.799216084499	-2.438191716391	-0.143128968750
H	7.505124069567	-2.077885687137	-1.734499968750
H	7.653460021114	-0.908699680990	-0.398977968750
C	3.429238026348	-1.034999856048	-4.317868968750
H	3.005198068362	-2.048822873621	-4.366480968750
H	2.668924997956	-0.349891887557	-4.704984968750
H	4.288002024746	-0.996340820460	-5.004018968750
C	3.481686889742	2.261340146125	-4.503572968750
H	3.381532917016	1.603214141975	-5.377322968750
H	3.622403847122	3.289782151957	-4.874003968750
H	4.392863901631	1.974470183886	-3.956528968750
C	2.127076826326	3.791596089988	-2.297610968750
H	3.122208822687	3.879396131228	-1.837369968750
H	1.973254789497	4.680297083613	-2.931055968750
H	1.358826825557	3.810159058150	-1.512859968750
C	0.398900884745	2.381925018369	-4.379521968750
H	-0.484588112082	2.305359981756	-3.724903968750
H	0.364899843835	3.369083016960	-4.868106968750
H	0.339431916359	1.619071015905	-5.168952968750
C	-0.595038142005	3.027405977179	2.024656031250
C	-1.459455114204	2.356572941356	2.916682031250
C	-1.044742103780	2.105022958542	4.225845031250
H	-1.730684082237	1.585185930116	4.902422031250
C	0.199050878874	2.523603010087	4.702680031250
C	1.033015850667	3.204241044648	3.815724031250
H	2.013119836374	3.549129085265	4.162048031250
C	0.668832840469	3.450309029556	2.489624031250
C	-2.840260098802	1.984899884133	2.478639031250
H	-3.444366135980	2.882034859098	2.266801031250
H	-2.816170074911	1.408403885131	1.541876031250
H	-3.358473074305	1.393785862658	3.246018031250
C	0.645381892056	2.205510028584	6.099664031250
H	1.213335931287	1.258842052121	6.126938031250
H	1.303245859575	2.989284055847	6.503860031250
H	-0.209443103213	2.091340993159	6.782377031250
C	1.604015809458	4.198619068311	1.590093031250
H	2.648615814701	4.072116111601	1.908145031250
H	1.502021823486	3.860125064085	0.552947031250
H	1.389725764621	5.280545059431	1.593707031250
C	-3.387239226021	5.054737861465	-0.201726968750
H	-4.100906207597	4.610167831890	0.508100031250
H	-3.688462269383	6.101084848982	-0.376062968750
H	-3.469856203566	4.512888858042	-1.156006968750
C	-1.611698261393	5.908268935047	2.110501031250

H	-0.603959265336	6.003421976809	2.539817031250
H	-2.029000303327	6.920162917753	1.983117031250
H	-2.231790239186	5.372422909349	2.846076031250
C	-0.575933263031	5.947805977971	-0.802562968750
H	-0.613116241793	5.435331976430	-1.776779968750
H	-0.971571305288	6.967468961575	-0.940971968750
H	0.478203733683	6.027089021656	-0.499961968750
O	0.339608086602	-2.488957984088	-1.438489968750
C	-0.134767858171	-3.821599003747	-1.472811968750
H	0.500796062076	-1.897128977408	-2.547731968750
H	0.462401168213	-4.458257978999	-0.809871968750
H	-0.037938842618	-4.196893999734	-2.502717968750
H	-1.187636855860	-3.877346047379	-1.166820968750
C	-0.274628972422	-1.064688009543	-4.411356968750
H	-0.743524932742	-2.022180028975	-4.672500968750
H	-1.035356000885	-0.377856041069	-4.007979968750
H	0.165571009753	-0.634562991300	-5.322071968750
O	0.755365037349	-1.300457966858	-3.473417968750
H	1.244756999328	-0.383001946577	-3.119472968750

152
MeOH_Cluster_Int_3, G=-4637.032681

Si	-1.748811980117	0.084540309721	-0.487906969165
Si	-0.019260755178	-1.445890702102	-0.165097440089
Si	0.519516488477	0.544684439406	-1.117267529204
Si	-0.955496718027	2.327538440956	-0.821816144960
Si	-4.613745577346	0.786139569259	-1.465475623528
Si	-0.458790344913	-2.937528211736	2.512804163777
Si	2.297924702698	1.906600812405	-3.465037337196
Si	-1.156843337475	5.135153348006	0.258994744291
N	-3.446513264014	-0.340110328644	-0.706210807126
N	0.614261942194	-2.253970139518	1.261249333163
N	2.007189600123	0.393015200830	-2.405521516751
N	-0.698457598539	3.445218454545	0.596271847156
C	-3.919717904164	-1.636158205473	-0.345969120028
C	-3.829465639748	-2.696648931964	-1.269845815427
C	-4.303550087738	-3.961435124972	-0.916555235150
H	-4.221528850792	-4.776254405519	-1.642698509787
C	-4.877251122191	-4.211484074805	0.332055110432
C	-4.994926024189	-3.139396310800	1.216626656345
H	-5.460755919266	-3.302081702457	2.193469439459
C	-4.539680464392	-1.855930330308	0.900708900808
C	-3.251561767555	-2.445520662844	-2.626103126182
H	-3.143106507707	-3.378498506632	-3.196035390980
H	-3.884997506006	-1.759283087918	-3.210241520481
H	-2.268624835351	-1.964317342791	-2.541811538872
C	-5.312533352842	-5.592865938133	0.723368604757
H	-6.142555697775	-5.567171539554	1.444395901363
H	-5.634341769226	-6.179296818380	-0.149699991459
H	-4.482084923127	-6.142188293155	1.198998314666
C	-4.779039746726	-0.728743049001	1.857958470491
H	-4.876144835171	-1.095321665351	2.888996965067
H	-3.977348733298	0.017015210823	1.822896611845
H	-5.714110919928	-0.200231191037	1.606179144599
C	-6.210985731041	-0.168461552778	-1.726902593708
H	-6.630451823040	-0.530217613939	-0.776002094338
H	-6.955545371171	0.493397663351	-2.198034528081
H	-6.070182026065	-1.042840798202	-2.379924791448
C	-4.937040843989	2.253041596208	-0.347086111658
H	-3.991566800431	2.754291110944	-0.101155770160
H	-5.584930097390	2.987724284763	-0.851141988001
H	-5.422261925890	1.949508878001	0.591991138856
C	-3.953734894285	1.393271642768	-3.114192483876

H	-3.836366286875	0.568007722791	-3.832975562834
H	-4.645814991963	2.131401524031	-3.550983332048
H	-2.976301382690	1.883282249764	-2.972476606680
C	2.009535323846	-2.137159289529	1.569810164333
C	2.455983175134	-1.113364974795	2.429952560825
C	3.796819152630	-1.080242466227	2.821116406833
H	4.120218000588	-0.286754784438	3.502868231395
C	4.721603575816	-2.030289176521	2.387412282840
C	4.268514899444	-3.012306716429	1.507644705150
H	4.969123873313	-3.768759924096	1.141114842096
C	2.938835377436	-3.077993771407	1.083292575192
C	1.512209320231	-0.084277338308	2.961102312419
H	2.053445383400	0.801303916124	3.313157232351
H	0.782419548331	0.244130755602	2.207286082482
H	0.937306729865	-0.469352282982	3.817816683086
C	6.151896436542	-1.975815388069	2.839559293361
H	6.223452952224	-1.803446317831	3.924348978523
H	6.684767473451	-2.909448880128	2.609144993063
H	6.699333854931	-1.154365802256	2.347413004568
C	2.518602010207	-4.160263603971	0.139151471685
H	2.139391612262	-3.732127336401	-0.800425995911
H	3.361018708772	-4.823798130545	-0.098954796951
H	1.708050066166	-4.769869860127	0.568137049326
C	-1.623635899885	-1.646982138090	3.226645188018
H	-2.089006628440	-1.080836116278	2.406994108561
H	-2.429971074940	-2.137122800032	3.795234415238
H	-1.119739518190	-0.924597076785	3.883181811990
C	0.637652875597	-3.674719799864	3.844638180807
H	1.264256960098	-2.913508115422	4.332419122938
H	0.012767291907	-4.153673885449	4.615366696240
H	1.311467699627	-4.437417805217	3.424575928641
C	-1.534771126268	-4.286268058878	1.772160769654
H	-0.937515205295	-5.103352758413	1.340402180153
H	-2.177283929066	-4.711065860632	2.560748489800
H	-2.204256909895	-3.888902330699	0.994537103856
C	3.234018192946	-0.227267529079	-1.914342112895
C	3.903700829743	0.303634385618	-0.798870728835
C	5.140944033630	-0.240594804161	-0.437964284965
H	5.653648477451	0.186449229888	0.427919351896
C	5.726673043987	-1.295328954683	-1.127675601363
C	5.016331898892	-1.829306439655	-2.203066509373
H	5.439832484611	-2.670913578928	-2.758728463901
C	3.784000942163	-1.323328443051	-2.614884724298
C	3.383010521951	1.418810308759	0.056282238592
H	3.338430099601	1.087817073096	1.103145840473
H	4.046687968076	2.295188930473	0.012540888345
H	2.370886065355	1.755366679444	-0.204724838715
C	7.048053543435	-1.870405706763	-0.714742824914
H	6.905083826959	-2.748619719032	-0.064044216334
H	7.633601480211	-2.200040887538	-1.585587552542
H	7.648663229392	-1.141755006957	-0.152210573159
C	3.097506833873	-1.984633872731	-3.772666659243
H	2.223859097636	-2.564299983125	-3.441404492612
H	2.717078848125	-1.275718293901	-4.520638405198
H	3.789293198847	-2.670801523889	-4.278699889508
C	3.798169645468	1.533751010931	-4.517888638559
H	3.639035037005	0.678399975013	-5.190467747712
H	4.019046110950	2.415476764347	-5.141043539305
H	4.682099792273	1.324097666596	-3.896927328456
C	2.598334229511	3.423325228254	-2.428730823962
H	3.545726769781	3.392664443802	-1.875459170444
H	2.635837878126	4.281983798296	-3.119204907700
H	1.767662827421	3.599314867408	-1.730935186613

C 0.751660631053 2.110467645947 -4.482574046742
H -0.135790157914 2.165170727090 -3.830907456401
H 0.819433552055 3.057084327858 -5.042390779965
H 0.623590540075 1.294274099359 -5.206210422877
C -0.236108550350 3.152033084302 1.904456856786
C -1.110418675291 2.568622966881 2.848650101338
C -0.671590720098 2.339735902645 4.153335330666
H -1.366436528873 1.886273549570 4.867511962184
C 0.609740999650 2.696208396331 4.578487488348
C 1.453625634550 3.294182948053 3.643421522537
H 2.462081864576 3.593060587217 3.948481517660
C 1.061712387184 3.520721412915 2.320672694512
C -2.525510326043 2.268135941877 2.470495946384
H -3.073540549952 3.191041807673 2.220797430227
H -2.570918589094 1.637077004521 1.570348245006
H -3.058454355289 1.759225012738 3.285146456352
C 1.078572122569 2.395049043786 5.971656921931
H 1.527193975048 1.387522895729 6.027272026718
H 1.843858224375 3.110936778646 6.306205300067
H 0.248427646502 2.419924787647 6.693038669517
C 2.008445576167 4.208303524790 1.384726487589
H 3.053566097533 4.018155499916 1.666794775859
H 1.850978373486 3.881723221467 0.351252856670
H 1.862533373833 5.301429085873 1.397478554299
C -2.923309753822 5.288328172782 -0.374635018776
H -3.656664282803 4.959769098163 0.376967000331
H -3.133057049134 6.344186939389 -0.613939774659
H -3.077753861128 4.694027787745 -1.287801779241
C -1.053564718945 6.106997346060 1.865796379500
H -0.038006165019 6.149325201804 2.284184954497
H -1.397828424728 7.139109340634 1.691103082877
H -1.704294500650 5.654916521907 2.630667463468
C -0.060175917700 5.914126215474 -1.063015780014
H -0.152062444592 5.347985122947 -2.003654533190
H -0.381219825076 6.950157654292 -1.260182353501
H 1.001385886418 5.933849835700 -0.777367741083
O -0.005599789573 -2.570002556642 -1.486000315701
C -0.500628495394 -3.886460969991 -1.481089394418
H -0.012188821694 -1.761646629269 -3.014736108041
H 0.078924007451 -4.532710093552 -0.806839223712
H -0.417959160186 -4.290581357430 -2.502481170368
H -1.555889475447 -3.928138011170 -1.172731810078
C -0.914235598922 -0.925712406775 -4.570858884821
H -1.531687148155 -1.802067784654 -4.833323469680
H -1.544114377007 -0.200660900702 -4.025577177598
H -0.593414129961 -0.457881770038 -5.513068533244
O 0.227428855409 -1.288870278895 -3.846713400326
H 1.513420336439 -0.262817234575 -3.055412762122

111
MeOH_Cluster_Int_4, G=-3708.789349
Si 2.250505942545 -0.972599208379 -0.952015424872
Si 1.038555596546 0.029811195876 -2.694863626488
Si -0.234357945232 0.821758418845 -1.050333949837
Si -1.350153079256 3.492913017287 -2.022676874666
Si 5.191343759375 -0.893244019709 0.023487285716
Si 0.152599090222 -1.074684221896 0.052595227758
Si -0.352761390093 -3.861853671385 1.275833571901
O 2.970322970409 -2.428069207325 -1.394364535402
N 3.543436928746 -0.213410205793 -0.063655753081
N -1.428380367524 2.061757940371 -0.935381013199
N -0.924681976035 -2.396008346220 0.410669452157
C 2.407777737118 4.737286997532 2.637606651414

H	1.551208071471	5.252697889402	2.173559430387
H	3.264693462986	5.427449534019	2.585404951590
H	2.167056040548	4.578789231353	3.698314280809
C	2.705989499913	3.441162275290	1.941099158446
C	2.607824151951	2.218806936453	2.603738108094
H	2.314083799694	2.204066410789	3.657832932639
C	2.869878978907	1.003561628037	1.962844776006
C	3.246766202261	1.008838422931	0.606563304257
C	-2.409218162513	2.018524106484	0.106188374631
C	-3.694126548801	1.518541882386	-0.166073578765
C	-4.653269459735	1.545986675281	0.848817789852
H	-5.655976357908	1.167605370245	0.629820176495
C	-4.366654353908	2.026316383436	2.127661812311
C	-5.402219761984	1.994767259328	3.214122670317
H	-6.418132747946	2.105349047077	2.807563028024
H	-5.239776794961	2.793734983999	3.952123579065
H	-5.372720677689	1.036440142999	3.760976446476
C	-2.088516329773	2.503180054486	1.388069855356
C	-0.712947277841	3.017094869315	1.678628515867
H	0.061548074116	2.261137381684	1.471323533406
H	-0.614053078461	3.316119627521	2.730922362045
H	-0.463926915646	3.888029753550	1.052515603907
C	-3.074876336515	2.495378668088	2.376799035109
H	-2.823531442668	2.873764148808	3.372274345566
C	0.365828381701	4.244046515689	-1.906924139170
H	1.131062951426	3.511725461216	-2.208626160983
H	0.601498424450	4.565992157404	-0.881842572555
H	0.450270896581	5.116155158120	-2.575082255057
C	-2.658876006808	4.691433145852	-1.427142576707
H	-3.665095594535	4.249153347789	-1.480226903684
H	-2.647297734162	5.598578034158	-2.051912338602
H	-2.482354354337	4.989213496611	-0.382403259477
C	-1.646561051677	2.968990954466	-3.798927444735
H	-0.929817023053	2.184528092472	-4.091056296475
H	-1.504795401167	3.825740493063	-4.477280968484
H	-2.661499324241	2.574382801524	-3.954714725477
C	2.798329640446	-0.280219043817	2.732395877454
H	2.481227032643	-1.109589630306	2.086205488169
H	2.101593472450	-0.200282760359	3.578790312058
H	3.786012191920	-0.552341780762	3.141596967192
C	3.085254249725	3.424689823547	0.595944842169
H	3.180948442115	4.370675037965	0.054210058968
C	3.363817465765	2.236840938705	-0.080863543099
C	3.819351195119	2.266521360730	-1.506039857908
H	3.165909943637	1.652883951415	-2.150230114031
H	4.832929809641	1.849190839045	-1.611361443178
H	3.827468760198	3.292241197787	-1.899061652033
C	5.920519884550	-1.097101636447	-1.695649290814
H	5.323665496762	-1.808035501434	-2.284434663213
H	6.950987260493	-1.482467344741	-1.631991705809
H	5.948745062861	-0.139487332145	-2.237468205980
C	6.216213530069	0.349377304984	0.991301637922
H	6.226793512529	1.335704047544	0.502868493325
H	7.256272369432	-0.006871532184	1.065308928353
H	5.830744664161	0.491334719218	2.012283452263
C	5.204317063064	-2.549578284386	0.908083788758
H	4.562684242968	-3.263744695738	0.372010715335
H	4.837257610933	-2.458496598979	1.941468370755
H	6.227058173143	-2.958361982767	0.944784083232
C	2.591887232959	-3.173148337831	-2.518546936478
H	2.892610990165	-2.672113061869	-3.455761489269
H	1.500965611637	-3.340360388956	-2.560612616139
H	3.086544375375	-4.155448673829	-2.470897823352

C	-2.296826225041	-2.325379577664	0.009289038533
C	-3.265166378712	-1.817128911726	0.892767510510
C	-2.863392290949	-1.251347132617	2.219998904794
H	-2.189870686508	-0.388484944294	2.091561112938
H	-2.327795215960	-1.990001665919	2.835019950029
H	-3.741074224763	-0.905146910940	2.781061967284
C	-4.607155140955	-1.839578744051	0.505667423619
H	-5.357302803748	-1.454092657085	1.201889565599
C	-5.012523028604	-2.325442821902	-0.737710830976
C	-4.027352854723	-2.798322526781	-1.608128829105
H	-4.318055037741	-3.178591645141	-2.592129482823
C	-2.675495057383	-2.808656992296	-1.258877268221
C	-1.644198453428	-3.336621158882	-2.209126565370
H	-0.873583877050	-2.575807778277	-2.415456416116
H	-2.100969708019	-3.631636833407	-3.163434525188
H	-1.122324762498	-4.213480013732	-1.794546485508
C	-6.456242498120	-2.295703509610	-1.147470970685
H	-6.688823159731	-1.375369910729	-1.710638400698
H	-7.124554088247	-2.321732736558	-0.274600839175
H	-6.710235845175	-3.144824274515	-1.798871413614
C	-1.822535083573	-5.010643612889	1.433453970409
H	-2.221504593789	-5.287970296032	0.445960191228
H	-2.641214321449	-4.543956742321	2.001615873977
H	-1.520671143794	-5.932705931283	1.955196869432
C	1.042592253156	-4.670980819325	0.319643055358
H	1.848663003162	-3.951846568358	0.104021887330
H	0.693643041335	-5.081585032766	-0.639869431099
H	1.472706146033	-5.498442038153	0.906666356993
C	0.308034038108	-3.380382510726	2.967290941919
H	1.132922741444	-2.657609904089	2.874827112560
H	0.699763312789	-4.267555888907	3.490349854241
H	-0.468002399375	-2.925250313387	3.600503881623
C	-4.019333102212	0.954342465197	-1.514154180905
H	-3.856148626959	1.689554671440	-2.316000427146
H	-5.064614384676	0.622334215052	-1.558949103641
H	-3.380646769253	0.085160634227	-1.740296076597

222
MeOH_Cluster_Int_5, G=-7417.602156

Si	-2.508127818812	2.287183243747	-0.257149560897
Si	-0.949992542967	0.586967233732	-0.767876436716
Si	-2.836969376408	-0.693886662583	-0.714542156013
Si	-2.085285994332	-2.998918283899	-2.562585611388
Si	-3.515777816360	4.932349748264	-1.524334929914
Si	-3.521580953374	0.579802790957	0.936255636199
Si	-5.114650713064	1.615708647794	3.392098826323
O	-1.963681712314	3.588899770437	0.657017895052
N	-3.306136136718	3.156787599062	-1.545845318057
N	-3.113420381284	-2.317661979136	-1.262415899002
N	-4.241946239792	0.328095658272	2.503794351944
C	-5.587960073513	0.207135717118	-5.929616854135
H	-5.033941931180	0.363875947756	-6.866390733627
H	-6.640579898077	0.474182826422	-6.105529764585
H	-5.561264881805	-0.874060585445	-5.709842027662
C	-4.998260373873	1.000095211923	-4.799600566848
C	-5.763644015619	1.340843917935	-3.683297555999
H	-6.818435546375	1.051371261785	-3.648614865236
C	-5.223011603769	2.030188904595	-2.596230645312
C	-3.867335279577	2.413641440473	-2.626232796000
C	-4.267796493460	-3.026968209200	-0.803361871410
C	-4.154954658601	-3.913569283562	0.282617489955
C	-5.282401560900	-4.629332523919	0.689826411333
H	-5.183879483991	-5.326061094312	1.527427324386

C	-6.523609695373	-4.472197683341	0.070498942271
C	-7.732175467424	-5.213336079674	0.564322916092
H	-7.462787009782	-6.197868889540	0.974208017100
H	-8.469514639624	-5.366217559615	-0.237091444085
H	-8.238497548706	-4.653209658268	1.369450205885
C	-5.506958467274	-2.850599005655	-1.447696406336
C	-5.629832518826	-1.914997665325	-2.609928427801
H	-5.202543617955	-0.924787554135	-2.390978686553
H	-6.680569154514	-1.776867877210	-2.899459687637
H	-5.086975665934	-2.295201855975	-3.490651371904
C	-6.613294748775	-3.573643742725	-0.994384473618
H	-7.574737685262	-3.431914754502	-1.497408458366
C	-2.039253792248	-1.798025471028	-4.006107127711
H	-1.638368945407	-0.824110365535	-3.682812522595
H	-3.038563372703	-1.615516948724	-4.427312444144
H	-1.386799991014	-2.183644943056	-4.806510613655
C	-2.845265730488	-4.639180200647	-3.052271019144
H	-2.887193298475	-5.326692012512	-2.193665323515
H	-2.241448434401	-5.112119916572	-3.842955050133
H	-3.871894900797	-4.516556092166	-3.428822950641
C	-0.337342946927	-3.227276651561	-1.933160925050
H	0.046668577114	-2.290703560609	-1.496978196592
H	0.341584561704	-3.512116338487	-2.751822992821
H	-0.283458055992	-4.005576509426	-1.158752821628
C	-6.081622499145	2.367849098019	-1.416959249476
H	-5.539555476442	2.179523016292	-0.478380995806
H	-7.005952333731	1.773759585017	-1.413673848066
H	-6.366612958691	3.432566990702	-1.412081075467
C	-3.657876624798	1.387580422256	-4.809324291935
H	-3.035475830868	1.139218978138	-5.674318244239
C	-3.079901893638	2.090098144898	-3.749108719821
C	-1.640132276624	2.495371636417	-3.813802277696
H	-1.514118708484	3.576083867343	-3.649601697950
H	-1.196696845987	2.233738990615	-4.784435841764
H	-1.053237111756	1.991746581197	-3.025287005683
C	-1.878890408853	5.853187220338	-1.598084184894
H	-1.256310568770	5.612082120897	-0.725374054161
H	-2.066761233643	6.939350314028	-1.601418729904
H	-1.312971853321	5.609261603961	-2.510090425554
C	-4.494048152940	5.363543158786	-3.070961123541
H	-3.965122427277	5.036664253734	-3.979546953314
H	-4.625618144772	6.456204204358	-3.127002903727
H	-5.490286776620	4.897458144088	-3.085341029035
C	-4.452522284290	5.491436303079	0.006429722349
H	-3.844676799361	5.313766391288	0.904987260572
H	-5.406381365620	4.955172874566	0.122380970586
H	-4.673134465724	6.569417094017	-0.054198052646
C	-1.037016428451	3.490267748054	1.700714245742
H	-1.166159572779	2.562871174813	2.285783239478
H	-1.167394307888	4.348244567792	2.378598799712
H	-0.004992563035	3.505834606091	1.315064892852
C	-3.980138539787	-0.927644282666	3.139198012927
C	-4.959640882954	-1.936723107965	3.139759892376
C	-6.257954544841	-1.735631810472	2.420543735230
H	-6.086318243048	-1.516434443877	1.354415540132
H	-6.830033611834	-0.892034481823	2.836021129645
H	-6.883130223637	-2.635656188745	2.481394563045
C	-4.690300134194	-3.135232337974	3.804558791699
H	-5.458472354277	-3.913887998974	3.809350323660
C	-3.471875032421	-3.371399497004	4.443424994063
C	-2.506980152181	-2.362491240565	4.406247104322
H	-1.542761290418	-2.522672824478	4.898444891277
C	-2.733890658527	-1.143941216900	3.762080329781

C	-1.664470800638	-0.095286785342	3.724324138866
H	-1.283388584014	0.059785952906	2.695783178932
H	-0.807696116248	-0.382315288338	4.348355677235
H	-2.036214318461	0.880470099463	4.071449154216
C	-3.185339047651	-4.688994860681	5.103059433162
H	-2.692569521719	-5.382863480013	4.400054822822
H	-4.107445337629	-5.177362664652	5.450156531025
H	-2.513799138930	-4.572334633985	5.966389744541
C	-5.473036977182	0.949895293443	5.104825254113
H	-4.540300895064	0.673802507189	5.620183564290
H	-6.110876458684	0.054068383179	5.072687737132
H	-5.985259628168	1.717642186704	5.706181495807
C	-4.042965262146	3.152764221065	3.483219604657
H	-3.727781351304	3.475673888807	2.478699095252
H	-3.137167050120	2.984729128244	4.085056910483
H	-4.607038010247	3.981584329423	3.940447578224
C	-6.692134718133	2.077970744022	2.484287922261
H	-6.460229083902	2.486162722340	1.488625491543
H	-7.236236753103	2.856598119251	3.042767117579
H	-7.365371569884	1.219182313926	2.347493891387
C	-2.848005913672	-4.069995537113	0.990956097125
H	-2.039350883853	-4.340952548713	0.297202788793
H	-2.909104963161	-4.837240079463	1.773787602036
H	-2.546806608300	-3.125318191379	1.470111083664
Si	2.508339441012	2.287496260913	0.256950699441
Si	0.950146783271	0.586885762504	0.765938928889
Si	2.837185964849	-0.693782151269	0.714789077920
Si	2.084928057080	-2.999100739619	2.562305128240
Si	3.515036410264	4.932550600749	1.525052175008
Si	3.522426893281	0.580000296035	-0.935791860954
Si	5.114836551073	1.615697804242	-3.392034207561
O	1.964412173696	3.589254400874	-0.657359690695
N	3.305934131460	3.156897155960	1.546005087460
N	3.113384520644	-2.317640185253	1.262454687303
N	4.242203372118	0.328131156043	-2.503536970105
C	5.589354595269	0.207328441080	5.929013856946
H	5.580725913155	-0.872486941229	5.701296302721
H	5.024433615255	0.349450309517	6.861551335329
H	6.636325848502	0.488378814687	6.116790006610
C	4.998787927754	0.999805634167	4.799103103400
C	5.764138222047	1.341255727643	3.682899994448
H	6.819058361622	1.052222671617	3.648163644582
C	5.223308848913	2.030743587937	2.596109639767
C	3.867382018116	2.413581767002	2.626138156312
C	4.267659217982	-3.027096552536	0.803371810299
C	4.154646224659	-3.913768918058	-0.282524008058
C	5.281993228320	-4.629693303512	-0.689730984491
H	5.183345769278	-5.326492915618	-1.527257134813
C	6.523256380278	-4.472635610131	-0.070498486295
C	7.731719948876	-5.213930139565	-0.564336480022
H	7.462167617055	-6.198304600784	-0.974490417216
H	8.468923655211	-5.367163420472	0.237136692115
H	8.238268173413	-4.653715125707	-1.369259482267
C	5.506880733658	-2.850794642488	1.447612921809
C	5.629903885612	-1.915066404638	2.609727983191
H	5.203118326411	-0.924683327647	2.390534196406
H	6.680648175230	-1.777378141750	2.899460202591
H	5.086622508273	-2.294871655461	3.490358407439
C	6.613112417202	-3.573991425305	0.994298079174
H	7.574605738050	-3.432321344806	1.497242501211
C	2.039114007012	-1.798686779000	4.006214620108
H	1.639238273622	-0.824315279731	3.683057411838
H	3.038341944066	-1.617188345693	4.428053465191

H	1.385899999223	-2.184050847615	4.806119667800
C	2.844497799600	-4.639684464002	3.051533105746
H	2.886280854037	-5.326992905063	2.192759391837
H	2.240555828815	-5.112677279781	3.842091005788
H	3.871146574429	-4.517389741898	3.428140383632
C	0.337013607701	-3.226779936938	1.932589024340
H	-0.046820616209	-2.289917618225	1.496863573360
H	-0.342024151876	-3.511944366761	2.751046849333
H	0.283049658657	-4.004642274778	1.157754014479
C	6.081891027072	2.369392336143	1.417089192241
H	5.539310095057	2.183283942449	0.478389922435
H	7.005583190106	1.774313731709	1.412534970496
H	6.368072072324	3.433806655879	1.413958184318
C	3.658267293886	1.386546748223	4.808794977491
H	3.035914288845	1.137504320750	5.673617955560
C	3.080006496861	2.089166265755	3.748713790399
C	1.639980716241	2.493505540317	3.813363114751
H	1.053461371141	1.989640534962	3.024707766442
H	1.513270761721	3.574157836583	3.649306453981
H	1.196622527311	2.231425755673	4.783911039856
C	1.877842665720	5.852838567594	1.598639348729
H	1.255464224162	5.611797061287	0.725770551830
H	2.065406560324	6.939054897580	1.602270519833
H	1.311842357897	5.608538699714	2.510495442709
C	4.492666622652	5.363550843136	3.072138599877
H	3.963701024064	5.035919500350	3.980429756544
H	4.623594586149	6.456259457858	3.128748272755
H	5.489175413568	4.898043290931	3.086538031422
C	4.452155493273	5.492395033370	-0.005199389692
H	3.844607982734	5.315081404865	-0.904029457259
H	5.406094913982	4.956266139141	-0.121090200692
H	4.672657573787	6.570367144923	0.056002184051
C	1.037254246830	3.491181218745	-1.700694240307
H	0.005401526653	3.506244260051	-1.314563745100
H	1.166351327360	2.564262469267	-2.286513298350
H	1.167162404877	4.349703539268	-2.377971538893
C	3.980287911170	-0.927608555801	-3.138904833280
C	4.959627429987	-1.936855582801	-3.139258715978
C	6.257871326408	-1.735932893883	-2.419871384644
H	6.086162750536	-1.517032832947	-1.353691424834
H	6.829948574593	-0.892203169173	-2.835070882871
H	6.883076031472	-2.635924725756	-2.480913459085
C	4.690194030841	-3.135340184308	-3.804037609735
H	5.458241066359	-3.914121199948	-3.808663443599
C	3.471827332183	-3.371330352507	-4.443109935773
C	2.507125281989	-2.362246466796	-4.406183338945
H	1.542979969894	-2.522259311227	-4.898574501488
C	2.734149520389	-1.143683195677	-3.762051465583
C	1.664971407256	-0.094782902334	-3.724683977245
H	1.283664039508	0.060519911761	-2.696268096588
H	0.808270594650	-0.381698222197	-4.348871110012
H	2.037013821423	0.880837354424	-4.071875002044
C	3.185245133244	-4.688954149091	-5.102662722263
H	2.695113629440	-5.383770007621	-4.398747114975
H	4.107077069017	-5.176053824833	-5.452285376756
H	2.511415387411	-4.572695978933	-5.964249762998
C	5.472404740790	0.949981771763	-5.104963065690
H	4.539431443579	0.674323397697	-5.620120293832
H	6.109865242468	0.053873680307	-5.073039542169
H	5.984823726845	1.717556535369	-5.706369982745
C	4.043470622357	3.153008920879	-3.482491149031
H	3.728679076862	3.475984079189	-2.477869932012
H	3.137417820845	2.985222042938	-4.084016122699

```
 H        4.607586764422      3.981689401675     -3.939920945212
 C        6.692871109125      2.077349939335     -2.484869644129
 H        6.461692694756      2.483250501436     -1.488103151484
 H        7.235808987336      2.857551638147     -3.042279506791
 H        7.366921017617      1.218803828776     -2.350556252058
 C        2.847626141440     -4.070134308862     -0.990745443451
 H        2.039051911126     -4.341231673012     -0.296948785800
 H        2.908665072654     -4.837277821935     -1.773678289470
 H        2.546322631614     -3.125413906769     -1.469744588799

222
MeOH_Cluster_TS_2, G=-7417.549725
 Si      -2.899549859067      2.196849696717      0.546299275039
 Si      -0.853351199390      1.204203286159      0.051881767429
 Si      -1.605768388764     -0.909341224904     -0.363442935158
 Si      -0.664065097397     -2.517539415817     -2.880792461056
 Si      -3.253068775960      5.044055953897     -0.391401339411
 Si      -3.586511457092     -0.085369841546      0.534920470819
 Si      -6.118595474525      0.181040617403      2.362937848782
 O       -2.863026850528      3.091264518265      1.973215430496
 N       -3.745769267260      3.323702142091     -0.487501738370
 N       -1.854438576078     -2.126115225740     -1.593906245214
 N       -4.678834151095     -0.712005906846      1.749026522588
 C       -7.691426044043      1.725380127608     -4.362021480937
 H       -7.347899962512      1.875460132673     -5.396348005232
 H       -8.642897237390      2.262034975339     -4.236237126362
 H       -7.904531554679      0.649317859574     -4.243009079719
 C       -6.663397817139      2.182091584883     -3.368162180191
 C       -7.033727954484      2.740778442289     -2.145428730287
 H       -8.095475441717      2.882725437990     -1.921450367134
 C       -6.091525029006      3.128197142573     -1.187753588288
 C       -4.720038525875      2.931399747962     -1.448779879997
 C       -3.059096081992     -2.898184306421     -1.653194652813
 C       -3.165669645401     -4.104131470804     -0.936090283883
 C       -4.247213613690     -4.950265710182     -1.198083286279
 H       -4.308187756481     -5.900901328279     -0.659640309793
 C       -5.243918694046     -4.618031466928     -2.115362625796
 C       -6.408093056473     -5.532363465705     -2.362467817851
 H       -6.158244301825     -6.577105583146     -2.127742095386
 H       -6.743057522992     -5.486975777432     -3.409252642772
 H       -7.271868699306     -5.252397595720     -1.734765631506
 C       -4.092129451666     -2.503780630062     -2.529176665262
 C       -4.046984317046     -1.172106410118     -3.211134249408
 H       -3.953500394660     -0.361595732653     -2.471795133511
 H       -4.962769229985     -0.994379935876     -3.790154272427
 H       -3.187009589005     -1.076828019283     -3.891244558783
 C       -5.160268845003     -3.375136015610     -2.748194018736
 H       -5.948868730498     -3.072296858216     -3.443827253087
 C       -0.073986136453     -0.867843081255     -3.560718386903
 H        0.565169672133     -0.341782675888     -2.835277174451
 H       -0.951253523489     -0.229810508207     -3.750436108988
 H        0.480314235587     -0.982673128796     -4.504122235338
 C       -1.522924645247     -3.485433478372     -4.241584657108
 H       -1.972552144356     -4.409738787236     -3.848216440276
 H       -0.762110379779     -3.768509256180     -4.987987507654
 H       -2.310386096073     -2.919277547619     -4.757691226659
 C        0.675347985593     -3.651472430548     -2.211414686627
 H        1.233892254190     -3.210340183157     -1.373523159412
 H        1.393382671129     -3.934806830741     -2.994335775686
 H        0.198353072534     -4.578021665265     -1.855186126613
 C       -6.550453887593      3.783507534208      0.079555761003
 H       -5.916223763552      3.511765284752      0.932752298966
 H       -7.592079303221      3.519794392871      0.307110824490
```

H	-6.505594512688	4.880695302630	-0.011965660411
C	-5.299067510686	2.042807607841	-3.627982379005
H	-4.976133297532	1.639159298173	-4.592466269164
C	-4.324205421337	2.409148215904	-2.698633331277
C	-2.874475374822	2.280610374374	-3.045996117413
H	-2.347675827214	3.236386177120	-2.905340236909
H	-2.743539998506	1.961511846160	-4.089382220907
H	-2.365448217111	1.548571929198	-2.397344940705
C	-1.384280240271	5.114583582804	-0.238943936641
H	-1.034861142716	4.619872441230	0.680220121585
H	-1.052927141745	6.163931678918	-0.193866668951
H	-0.877961107802	4.638424663810	-1.090013987206
C	-3.817874719009	5.856136060936	-1.987467968132
H	-3.369157811667	5.382175561021	-2.873335862701
H	-3.529664625436	6.919499245636	-1.983807018734
H	-4.911589947113	5.800286320754	-2.099680742596
C	-3.983161725389	5.968578694161	1.072756603976
H	-3.836283506444	5.378001209912	1.988268283067
H	-5.055714486919	6.179028328748	0.957881960600
H	-3.461263700177	6.932308570269	1.190928574748
C	-2.103701839503	2.794008360355	3.115258534069
H	-2.643137629533	2.094623701081	3.776499987852
H	-1.930763006087	3.728126065609	3.672698830244
H	-1.123842232960	2.348474201451	2.866824490903
C	-4.466532957210	-2.022830700595	2.283469609554
C	-5.158364816409	-3.129880264872	1.759839932978
C	-6.139466187411	-2.955601771822	0.644468251923
H	-5.690468118428	-2.437595940830	-0.215415416254
H	-7.003636211841	-2.355928601332	0.968444723039
H	-6.516964828039	-3.925571931552	0.300048099376
C	-4.939295311101	-4.387560049048	2.326101877086
H	-5.469168773735	-5.248478059998	1.907797193312
C	-4.069114867407	-4.574824622927	3.402074651269
C	-3.432128076747	-3.450348240457	3.931624955708
H	-2.774386635744	-3.566708877968	4.798320824503
C	-3.614179955107	-2.174780691745	3.392570991550
C	-2.955054452667	-0.974213980667	3.999599498629
H	-2.344179890306	-0.430818591805	3.261256789607
H	-2.304069689160	-1.257068825686	4.836736210100
H	-3.705542883430	-0.260046144672	4.374023011422
C	-3.801465389624	-5.945296085380	3.952482651732
H	-2.942471333468	-6.412064665766	3.440512224382
H	-4.664499857828	-6.612986440934	3.815217381154
H	-3.560827869757	-5.911397341590	5.025153617099
C	-6.962623445005	-0.915590848628	3.628670413159
H	-6.281554146995	-1.160153443092	4.458040453590
H	-7.322999137439	-1.863380313613	3.204257617666
H	-7.827329721097	-0.374470198549	4.046098047117
C	-5.624409466852	1.775645952905	3.217468668195
H	-4.909526284914	2.384557820899	2.644978403236
H	-5.167422742437	1.559970953237	4.195700828308
H	-6.523670562072	2.386623338982	3.397709920903
C	-7.255625704620	0.538821458566	0.915938311230
H	-6.710431798207	1.016365593937	0.087675758111
H	-8.072413906845	1.213828223357	1.215783518016
H	-7.702640719398	-0.386152988254	0.524274748812
C	-2.161502735403	-4.459837847969	0.112955188025
H	-1.160591478726	-4.100882341055	-0.147311715507
H	-2.120306543410	-5.544118466230	0.285991578855
H	-2.433065555891	-3.986434629691	1.072031595988
Si	2.236709271784	2.322701307365	-0.281684398627
Si	1.031754820497	1.166290060466	1.391066926952
Si	1.519131250617	-1.001679371355	0.344740586716

Si	1.339607737687	-3.308739409923	2.429430346442
Si	3.680748186429	5.118369963737	-0.256616476220
Si	2.953097460685	0.137071836680	-1.126095463716
Si	5.023288745774	0.919149428916	-3.347917106667
O	1.594379533251	3.268333995081	-1.527115575529
N	3.372061226713	3.478932542903	0.402109943646
N	2.296541385267	-2.266167550626	1.337201198946
N	4.328174176033	-0.247161020088	-2.141515718716
C	5.079153834715	2.550432628439	5.831205207956
H	4.824832976486	1.518752805894	6.129665162044
H	4.588990431433	3.226821522737	6.546134560744
H	6.167824044878	2.661219111400	5.945055913435
C	4.641558586448	2.823727817203	4.421925450720
C	5.428945346322	2.434993138630	3.336641970942
H	6.394112939426	1.951690467251	3.516788512464
C	5.012500407972	2.624828774453	2.019699809678
C	3.775274748194	3.249808901627	1.757726984927
C	3.700364757460	-2.327957742636	1.601178095707
C	4.473612073212	-3.360914749960	1.032902783996
C	5.797211502022	-3.528533344307	1.447409643348
H	6.379165906349	-4.346073960184	1.011880615133
C	6.395722356768	-2.686619868278	2.385076071650
C	7.834218222201	-2.858803595834	2.776862637883
H	8.156252928748	-3.906391890110	2.683397909069
H	8.012966111847	-2.536862389345	3.813444057453
H	8.496900358119	-2.255343255886	2.132123937078
C	4.284029783399	-1.465368332220	2.557033025355
C	3.489993807462	-0.389763499734	3.229548797661
H	3.448423547843	0.527545240076	2.621574050477
H	3.941339096640	-0.108549785613	4.190399519301
H	2.448324716395	-0.692048020393	3.400441167053
C	5.620913769419	-1.655019253369	2.917527625866
H	6.057282340446	-0.987165476326	3.666883125669
C	2.344214561531	-3.681557978173	3.973882764401
H	2.580899574462	-2.777885754426	4.553567347202
H	3.292282530495	-4.183017669824	3.726930543668
H	1.757268745603	-4.358066123534	4.617042109596
C	0.873569516817	-4.979702618309	1.687900902849
H	0.641301520081	-4.915569178607	0.616630245819
H	-0.017149976379	-5.373683772748	2.204030826460
H	1.686808373577	-5.709659778615	1.819262670195
C	-0.207247652771	-2.379644688356	2.913009594602
H	0.039104276228	-1.367643014363	3.269413450179
H	-0.722008517941	-2.922903621227	3.718927588822
H	-0.919468308633	-2.273743179714	2.081306252374
C	5.881344427197	2.173208734732	0.891531111954
H	5.321426767276	1.550284804798	0.180327597465
H	6.735446629771	1.587880181876	1.257312994032
H	6.271361469017	3.027947424505	0.317013774287
C	3.417434746567	3.428962267310	4.146654489419
H	2.777779156271	3.741200947872	4.977928110787
C	2.969342872248	3.658820708462	2.841169977674
C	1.666948201393	4.377227653022	2.648861959718
H	1.232643161580	4.194712585993	1.658602571965
H	1.804739904287	5.466507899113	2.756424567131
H	0.935190929971	4.059962814532	3.404927151147
C	2.148535212350	6.204868397515	-0.300333028233
H	1.361834214201	5.718049473646	-0.891903149054
H	2.402382955323	7.165484527968	-0.777735264693
H	1.747281316798	6.419566257541	0.699320257800
C	4.948176242836	5.913460821881	0.881550621832
H	4.560415192710	6.026395858519	1.905198812183
H	5.201097340033	6.915309884651	0.498272289900

H	5.877274826978	5.326741561016	0.942069536489
C	4.365302138653	5.088689868531	-2.004591589151
H	3.755981695590	4.457810317332	-2.665985787326
H	5.406661538774	4.742789976659	-2.043635097724
H	4.338046633788	6.116280109012	-2.401851145532
C	0.679997974535	2.817788543365	-2.475887728536
H	-0.308967484148	2.612021547771	-2.024027295732
H	1.010505263773	1.885815461637	-2.969193617636
H	0.549205371369	3.594218986274	-3.246109662417
C	4.875791855173	-1.568052248470	-2.272778493402
C	6.025035491919	-1.932504063624	-1.551103120161
C	6.613440457616	-1.007041952979	-0.535560488524
H	5.904416173832	-0.828408590121	0.286840167235
H	6.855445481067	-0.027650747365	-0.969576487620
H	7.528240789006	-1.430065125271	-0.101612943576
C	6.609718447279	-3.175814050734	-1.803524406212
H	7.508801852547	-3.449720207867	-1.244088669366
C	6.082480137671	-4.073626051094	-2.732165361693
C	4.937230745852	-3.689212965120	-3.431974149200
H	4.504646729344	-4.369983355270	-4.171231975928
C	4.329152881673	-2.450344197473	-3.225015783925
C	3.131182782222	-2.048505723821	-4.024766717151
H	2.317563464281	-1.714200748307	-3.370811446007
H	2.763553617460	-2.882440247876	-4.637988279056
H	3.352022119462	-1.207871338015	-4.701515343121
C	6.700191863387	-5.425530944247	-2.937730176885
H	6.269887742789	-6.163851247300	-2.239362499731
H	7.785541313982	-5.405279087027	-2.761209718073
H	6.524574747276	-5.801156017975	-3.956272540372
C	6.007594563228	-0.043443517283	-4.623143527555
H	5.407540656269	-0.740936479626	-5.222873876129
H	6.814582509685	-0.619909550646	-4.146170350128
H	6.469797969359	0.686069793858	-5.308505957549
C	3.626619925307	1.873873566050	-4.149367026290
H	3.125361911837	2.514877136126	-3.410737895447
H	2.874731487318	1.206284484969	-4.596214015906
H	4.029414111703	2.524201350946	-4.942334719451
C	6.205640675598	2.134994427645	-2.558522251877
H	5.724598282782	2.676871514575	-1.737023086162
H	6.512894494370	2.871438329580	-3.318365489599
H	7.112818657803	1.646684770711	-2.174452088451
C	3.892206141331	-4.268977694406	-0.006115592326
H	2.985096693799	-4.775991814724	0.343818276597
H	4.620379640816	-5.031879988443	-0.311023530948
H	3.608708851434	-3.698841393001	-0.903274228766

222
3, G=-7417.682400

Si	-2.084117248261	-0.028412575992	2.148648930047
Si	-0.575048995763	1.100893592710	0.716062081647
Si	-1.181991527434	0.033296475957	-1.298301676046
Si	-2.326347273907	2.349822319273	-3.035368337897
Si	-3.782245652859	0.842392858748	4.582725537188
Si	-1.717864900835	-1.592685962464	0.369822979802
Si	-3.813692164999	-3.868764345682	0.924436307965
O	-1.534649527704	-0.593050117621	3.628513510924
N	-3.484197278317	0.802180973058	2.804909998760
N	-2.278433175282	0.635026534107	-2.539146930686
N	-2.256754338098	-3.268890233796	0.300195077118
C	-7.127686728628	4.084569162391	-0.214112904181
H	-7.006601016410	3.877622082477	-1.291209702632
H	-6.945207860320	5.158708031075	-0.065653025535
H	-8.177509452012	3.876218849998	0.040072841579

C	-6.185225397023	3.246779123660	0.599525936615
C	-6.515697089365	1.940399853184	0.968347483014
H	-7.495503600566	1.538095894759	0.692493886832
C	-5.628743283199	1.122721983136	1.665779575868
C	-4.356570364118	1.619577738105	2.025757985899
C	-3.217653140482	-0.256204327232	-3.143822834097
C	-2.947659678122	-0.866399970010	-4.383811148936
C	-3.893909131606	-1.729481865619	-4.942080899027
H	-3.664993197472	-2.207685081791	-5.899555966241
C	-5.110738620766	-2.001333778674	-4.316451466609
C	-6.086296399250	-2.979905079770	-4.902560648640
H	-6.002025304966	-3.029719678479	-5.997982910840
H	-7.124044723297	-2.717001682748	-4.649941423436
H	-5.904697791931	-3.997097019166	-4.514999225654
C	-4.458446176141	-0.482479590327	-2.519397742417
C	-4.798232819694	0.230518627086	-1.253230326374
H	-4.102751908590	-0.022849352212	-0.437835985028
H	-5.813893172066	-0.014466676161	-0.922210740902
H	-4.736848615465	1.323556045459	-1.362862166251
C	-5.377671618145	-1.351141290289	-3.110354810801
H	-6.336136891931	-1.519008332316	-2.610369935412
C	-2.394046327973	3.445831342899	-1.525440179457
H	-1.418213397627	3.481933757620	-1.018644066543
H	-3.150153550920	3.094258831518	-0.809708341259
H	-2.648141229047	4.476166797479	-1.820504171016
C	-3.864526297929	2.578291012523	-4.088693177277
H	-3.852416507155	1.929452814407	-4.977489020250
H	-3.918838795243	3.625005500899	-4.428906526460
H	-4.782790493143	2.353084829438	-3.525712353388
C	-0.813617804908	2.838346872704	-4.031317314157
H	0.108189086379	2.607512988846	-3.477436704495
H	-0.830469202036	3.926538616329	-4.199967574721
H	-0.768704326819	2.336930311126	-5.007577818448
C	-6.038121534801	-0.264711009458	2.052914946071
H	-5.209258733872	-0.972790245479	1.936501479906
H	-6.889211185662	-0.612473890744	1.451227461122
H	-6.341249487625	-0.312256326895	3.111724761532
C	-4.938441054441	3.729599748240	0.989054252088
H	-4.660964410664	4.758152291601	0.737979323682
C	-4.023463099877	2.949544569505	1.706120129790
C	-2.737589630289	3.581162892491	2.143953466562
H	-2.128885711429	2.893475043132	2.740872663702
H	-2.933783277946	4.479830862558	2.750918100014
H	-2.142302493784	3.898661500321	1.275011444090
C	-2.380371483388	1.614680904653	5.569313031649
H	-1.504156062626	0.955523868023	5.605373216149
H	-2.728994876681	1.795909767180	6.599215190181
H	-2.074986953566	2.584291013370	5.146898077314
C	-5.265265873843	1.977146924339	4.836848121584
H	-5.061912056359	2.993077565169	4.465224279580
H	-5.465144320366	2.045377187484	5.918796514466
H	-6.182200480001	1.624736845884	4.343565606146
C	-4.201032895779	-0.831600730054	5.328532557676
H	-3.348175216587	-1.522800641447	5.300367508683
H	-5.048530926088	-1.311117396040	4.817762073392
H	-4.485185104622	-0.683783311593	6.383440351252
C	-0.823366686351	-1.735709292727	3.982775218261
H	0.166768390251	-1.770275268488	3.499207208478
H	-1.375148856756	-2.653188689056	3.723997554402
H	-0.662309754097	-1.722972563006	5.071231911821
C	-1.394515607174	-4.233810174804	-0.310568430842
C	-1.495853044493	-4.517154828010	-1.684940035461
C	-2.509473885186	-3.823446482687	-2.540467254547

H	-2.606926903517	-2.757957119412	-2.293518866690
H	-3.510012419659	-4.266342212106	-2.403672669219
H	-2.259288601827	-3.908700439805	-3.605780849547
C	-0.678673516545	-5.510556387632	-2.233798125863
H	-0.767931536028	-5.726299767913	-3.302781438047
C	0.230429187370	-6.236246109302	-1.465138811004
C	0.297527940585	-5.954096899669	-0.098873041487
H	0.989525882524	-6.519141508908	0.533058472144
C	-0.497978064112	-4.971446480792	0.491428826066
C	-0.435031895158	-4.733650755650	1.967482011463
H	-0.218912013214	-3.679634643058	2.184803675818
H	0.340980637367	-5.351859589420	2.437479445206
H	-1.395139857913	-4.966722394669	2.453776848229
C	1.132534779205	-7.260029066053	-2.089214792180
H	2.099044122696	-6.810381887220	-2.375855555810
H	0.690011080654	-7.688196168426	-3.000293261211
H	1.352638941256	-8.083817637733	-1.394348312620
C	-3.831448456903	-5.734369003880	0.727115380715
H	-3.016010352266	-6.225954272363	1.277369608406
H	-3.738754549034	-6.023548690712	-0.330773627086
H	-4.788448832954	-6.127681827005	1.106554404301
C	-3.995039880301	-3.411150711082	2.738269715501
H	-3.753680330964	-2.354237452430	2.922370384912
H	-3.329185474214	-4.018315006228	3.370050111880
H	-5.028543459477	-3.577986738613	3.080921799728
C	-5.255066881651	-3.160490591869	-0.042306705356
H	-5.233923912848	-2.063946605771	-0.059789917783
H	-6.209590141869	-3.473775322494	0.410699458674
H	-5.234305573304	-3.506621657285	-1.085516001660
C	-1.676483113083	-0.576843850477	-5.114190846298
H	-1.729384715672	0.392527175230	-5.636599949900
H	-1.461660206975	-1.348225068460	-5.866052546367
H	-0.831156492478	-0.517375755496	-4.420406384101
Si	2.084057843590	0.028463951479	2.148704580350
Si	0.575024717421	-1.100873563958	0.716116312696
Si	1.182031349730	-0.033347101296	-1.298271269441
Si	2.326525397232	-2.349914422861	-3.035238720020
Si	3.782079306362	-0.842253135165	4.582862420870
Si	1.717854743468	1.592683757674	0.369828513360
Si	3.813676778880	3.868763751538	0.924479595799
O	1.534566509695	0.593159283602	3.628538984656
N	3.484118960784	-0.802099411856	2.805032480274
N	2.278487823835	-0.635101342217	-2.539105345275
N	2.256769374364	3.268878195044	0.300169165698
C	7.127647660318	-4.084499364413	-0.213929706006
H	7.006557810774	-3.877560280444	-1.291027659880
H	6.945175228128	-5.158638165418	-0.065461872479
H	8.177469772710	-3.876140931131	0.040251920031
C	6.185182887746	-3.246708540922	0.599704114068
C	6.515635173107	-1.940313356604	0.968487504634
H	7.495429200970	-1.537996594283	0.692607952971
C	5.628675710919	-1.122635248774	1.665913427806
C	4.356519800928	-1.619508939560	2.025924340911
C	3.217689654877	0.256138372408	-3.143800991338
C	2.947718182028	0.866212466742	-4.383852855208
C	3.893948730548	1.729295949227	-4.942157622367
H	3.665046047961	2.207405305953	-5.899682663513
C	5.110743290323	2.001251940247	-4.316511974958
C	6.086285227935	2.979812383933	-4.902665689620
H	6.001942935941	3.029650436525	-5.998080903358
H	7.124044619584	2.716879406755	-4.650120204406
H	5.904738578585	3.997000681072	-4.515070151437
C	4.458465672120	0.482504682485	-2.519362300383

C	4.798274988498	-0.230412306192	-1.253154838212
H	4.102819201484	0.023009432573	-0.437756848338
H	5.813944689099	0.014590764067	-0.922176463811
H	4.736889223091	-1.323457218879	-1.362715812588
C	5.377670218793	1.351157992220	-3.110359174483
H	6.336119005441	1.519094544667	-2.610366666691
C	2.394130178713	-3.445855808374	-1.525256317270
H	1.418255838016	-3.481970777451	-1.018540741782
H	3.150165495607	-3.094226820521	-0.809476942032
H	2.648288097741	-4.476193844708	-1.820256815218
C	3.864812561226	-2.578366351005	-4.088407708836
H	3.852771492549	-1.929560686949	-4.977228306356
H	3.919187535044	-3.625091979273	-4.428578976037
H	4.783016665909	-2.353117354632	-3.525345408830
C	0.813920181461	-2.838557689823	-4.031319502114
H	-0.107948345917	-2.607871732540	-3.477479785999
H	0.830917932551	-3.926734733720	-4.200045543062
H	0.769008252114	-2.337080251161	-5.007548564596
C	6.038027651704	0.264820598553	2.052996638170
H	5.209131734526	0.972867085341	1.936622721323
H	6.889067910473	0.612603370251	1.451250404246
H	6.341222626517	0.312394679057	3.111786564683
C	4.938410304841	-3.729541727787	0.989256698001
H	4.660944664005	-4.758101792303	0.738200925683
C	4.023427144422	-2.949485451578	1.706314740431
C	2.737563162627	-3.581110802093	2.144168970966
H	2.128769531095	-2.893353325950	2.740917307761
H	2.933778455712	-4.479647356449	2.751322489202
H	2.142359355642	-3.898818952970	1.275247466554
C	2.380158659386	-1.614524080837	5.569395815441
H	1.503924990956	-0.955386392211	5.605374856724
H	2.728720784538	-1.795705541666	6.599327178705
H	2.074824260935	-2.584156873632	5.146997980317
C	5.265105741899	-1.976971836173	4.837113695170
H	5.061795250664	-2.992922059270	4.465520114763
H	5.464924832689	-2.045155968283	5.919076371945
H	6.182061801808	-1.624561634019	4.343871227581
C	4.200797207855	0.831780008777	5.328616685791
H	3.347938804446	1.522975669707	5.300354701651
H	5.048332052314	1.311271806452	4.817884524095
H	4.484871464752	0.684014657247	6.383553305486
C	0.823260662010	1.735821373179	3.982743511970
H	-0.166853689647	1.770371167589	3.499132020056
H	1.375048906828	2.653297904354	3.723968203741
H	0.662158050058	1.723109992683	5.071193697911
C	1.394556540900	4.233787945845	-0.310649633258
C	1.495937331040	4.517083016682	-1.685029797197
C	2.509588068482	3.823342299559	-2.540495708094
H	2.607011867724	2.757855905759	-2.293523599372
H	3.510127193603	4.266223098620	-2.403657850025
H	2.259455549317	3.908580824761	-3.605823035829
C	0.678770485037	5.510458169144	-2.233949593134
H	0.768065308719	5.726166273002	-3.302937114156
C	-0.230366408666	6.236168942514	-1.465345939921
C	-0.297512328085	5.954065547926	-0.099075364381
H	-0.989537839275	6.519124707360	0.532812618299
C	0.497984573261	4.971442339744	0.491289782621
C	0.434984820251	4.733700988833	1.967348976202
H	0.218886814030	3.679687158725	2.184704281582
H	-0.341064214793	5.351906171858	2.437290251504
H	1.395065373742	4.966819519878	2.453674812433
C	-1.132457156857	7.259916362459	-2.089501185272
H	-2.098813949980	6.810164924597	-2.376493386584

H	-0.689745781961	7.688305887811	-3.000385074739
H	-1.352884369039	8.083552887331	-1.394558569991
C	3.831444842246	5.734368530413	0.727154296604
H	3.015998605479	6.225961078349	1.277389478237
H	3.738780137269	6.023550667791	-0.330736439364
H	4.788438970967	6.127673860251	1.106618371877
C	3.994946624621	3.411152141557	2.738322442920
H	3.753555126036	2.354244480690	2.922415530952
H	3.329072278114	4.018330963874	3.370067807410
H	5.028435800555	3.577967800165	3.081024785142
C	5.255102747046	3.160497502178	-0.042193352316
H	5.233971827900	2.063953322912	-0.059673001396
H	6.209597758238	3.473796027553	0.410863612460
H	5.234392604660	3.506629359253	-1.085403346828
C	1.676605721508	0.576510301086	-5.114290614691
H	1.729668225114	-0.392824454222	-5.636752885911
H	1.461700981367	1.347898564322	-5.866123116312
H	0.831264552613	0.516885072486	-4.420540532560

146
MeOH_Int_1, G=-4521.560257

Si	1.591125354442	0.495725438932	0.604747275381
Si	-0.286322690982	0.464338879444	-0.638266477755
Si	-0.119156488347	-1.422230766817	0.547952920150
Si	2.217353644599	-1.771261255612	0.502103702259
Si	4.050825045843	1.670367130126	2.121320742485
Si	-1.128298578489	3.069617373922	-2.150532245231
Si	-1.032164567893	-4.150448183199	1.732483604618
Si	4.050636560593	-3.296130185081	-1.399912141055
N	2.448343074124	1.835565315048	1.314340410588
N	-1.486408751387	1.509021715273	-1.329969300359
N	-1.346575272455	-2.469869730049	1.180096315726
N	2.726373602900	-2.137245608611	-1.207073970704
C	1.785883188023	3.100218004806	1.396960011007
C	0.718846309523	3.276358979516	2.303978031255
C	0.094019722776	4.523450762583	2.375801984480
H	-0.731363370661	4.653465132384	3.082376892893
C	0.496093985871	5.605264627185	1.588500806845
C	1.562955793721	5.408753554936	0.711362001407
H	1.899295514949	6.239537064667	0.083573936929
C	2.218213980701	4.179178860444	0.600263132137
C	0.272808471936	2.155794342262	3.192511033510
H	-0.436957196808	2.514144233828	3.950633832692
H	1.117817058603	1.666039901222	3.696748586433
H	-0.222024976588	1.354791216304	2.619702930962
C	-0.224123260866	6.920214377711	1.654786412416
H	0.428361906764	7.754360834224	1.359028062369
H	-0.602621457095	7.122913234287	2.667402943128
H	-1.092830911910	6.925122426599	0.974386609767
C	3.375116398422	4.035146974349	-0.345193073183
H	3.330915862586	4.795188822433	-1.137224475572
H	3.395148045494	3.041568409622	-0.812887764521
H	4.337941654755	4.161563874780	0.176272265547
C	4.358615756806	3.277866157126	3.040838955321
H	4.437725124593	4.157533157631	2.387369618008
H	5.298077097605	3.187382869564	3.609529873402
H	3.544965950967	3.469863702076	3.757471330487
C	5.425017733397	1.296717923781	0.898063443365
H	5.313517096501	0.279869613437	0.492448082840
H	6.389716085684	1.333621665199	1.430272984482
H	5.471581371827	2.002569605533	0.057529738658
C	4.054914851295	0.285447999763	3.379203479044
H	3.277024022225	0.434846261970	4.139433747771

H	5.037575062960	0.263695539605	3.878271607535
H	3.891792253521	-0.690064947321	2.895922914185
C	-2.884676176865	1.199791546944	-1.245459813949
C	-3.544923321753	0.566873778524	-2.317084183730
C	-4.938343144106	0.466219639005	-2.279890257971
H	-5.450001682423	-0.008033882360	-3.123812054748
C	-5.691863574744	0.964507498822	-1.217648172805
C	-5.004448540654	1.509179245267	-0.131590252164
H	-5.566790637451	1.863604771221	0.736960324452
C	-3.613032235052	1.609679080329	-0.108611370929
C	-2.800479838197	-0.013564305345	-3.483964438036
H	-3.047281382422	-1.080495617961	-3.597454376591
H	-1.714370850327	0.067962775885	-3.360037315074
H	-3.076810176346	0.482845749265	-4.426827717551
C	-7.189449090470	0.864792158543	-1.227722769615
H	-7.643350381757	1.517903273844	-0.468966628259
H	-7.522138931611	-0.165133928407	-1.016390234033
H	-7.603824974270	1.142976429419	-2.208568110822
C	-2.910541151348	2.089398030041	1.125407016497
H	-3.610755387745	2.581834642382	1.814117504292
H	-2.084147487893	2.777341999446	0.908116212386
H	-2.479161152264	1.225598644701	1.658970602969
C	-1.726394439238	4.502130750042	-1.101334772245
H	-2.802669083510	4.418093907008	-0.885876551270
H	-1.560527296625	5.447566902201	-1.643018051666
H	-1.174114240259	4.554327757945	-0.151714377253
C	0.716175067798	3.148009025567	-2.405973351176
H	1.237516419110	3.165749315255	-1.441240827655
H	0.979522178557	4.069676661912	-2.949062237542
H	1.076482642300	2.283289420606	-2.982158960096
C	-2.005226006093	3.151001224935	-3.807045750953
H	-1.607465676428	2.413694958110	-4.518652641578
H	-1.844869931291	4.155031951800	-4.232934292334
H	-3.090078538520	2.997210258418	-3.707258875842
C	-2.682590003082	-1.978969104895	1.332442498405
C	-3.610576919527	-2.160964389702	0.289796003227
C	-4.944928656180	-1.815041308965	0.504211533096
H	-5.665370531098	-1.980809153591	-0.301427401231
C	-5.380615505824	-1.268899991136	1.713011961651
C	-4.429108131723	-1.044043950802	2.708155238843
H	-4.743343359924	-0.602932604365	3.659419740925
C	-3.085181588039	-1.391451670833	2.546122809367
C	-3.174077946199	-2.757409155975	-1.013448934038
H	-3.989874538044	-2.730202377945	-1.748505493823
H	-2.852509658917	-3.804831543583	-0.894909291380
H	-2.314813687984	-2.212073956674	-1.432882548903
C	-6.820343843446	-0.907697685816	1.933673717127
H	-6.996993491383	0.164190512476	1.745496072361
H	-7.130863499809	-1.110663398123	2.969400622885
H	-7.485069333909	-1.470164924244	1.262434411698
C	-2.118382816306	-1.196972528239	3.675285998231
H	-2.475079747015	-0.416945109255	4.363142835265
H	-1.105395858450	-0.937437899299	3.337549335443
H	-2.013636503738	-2.124379972891	4.264512896365
C	-2.685968841737	-4.852340623575	2.270889499415
H	-3.124439133114	-4.273561857083	3.097617206731
H	-2.546548988278	-5.890630278710	2.612258061935
H	-3.416372754120	-4.855592121987	1.447916403352
C	-0.308135066722	-5.206851461433	0.361807812363
H	-0.947647626301	-5.243907150084	-0.532137714539
H	-0.186912209501	-6.235969380016	0.737924295378
H	0.686164523567	-4.844257835203	0.063656129044
C	0.179332385639	-4.190890419439	3.159486550039

H	1.153767674048	-3.787687584284	2.843431422640
H	0.328349666923	-5.232517633510	3.487171190031
H	-0.182733200871	-3.603071728170	4.013353207815
C	2.167332425151	-1.511696227200	-2.348391346664
C	2.736887260001	-0.333114800115	-2.875250821166
C	2.172453052160	0.263722723806	-4.004848933528
H	2.634566853372	1.173267668221	-4.401563608425
C	1.040855038722	-0.257685029496	-4.634848594651
C	0.488643264243	-1.423237976410	-4.102231355700
H	-0.395735921430	-1.861481132014	-4.574538181068
C	1.032581714104	-2.059367262883	-2.983316911910
C	3.931970195473	0.280758841737	-2.218993796812
H	4.831086671447	-0.345339463249	-2.332522048228
H	3.767415716511	0.386548786501	-1.136698810781
H	4.154460847729	1.271753358577	-2.638591883819
C	0.456349711914	0.399812666869	-5.851603255306
H	0.556175717836	1.494882332107	-5.804966671257
H	-0.611126986111	0.159894923312	-5.964942014527
H	0.965848688206	0.067017232611	-6.771614206826
C	0.413651844122	-3.310751970575	-2.454423098282
H	-0.466671267928	-3.606181975204	-3.041674562857
H	0.098984212317	-3.174420144814	-1.407913176170
H	1.127287192245	-4.148502918170	-2.447771537659
C	5.590998634536	-2.703031386628	-0.491503769026
H	5.982255056235	-1.766967796255	-0.918103036085
H	6.390859998394	-3.459838713286	-0.536781262811
H	5.359295261499	-2.519479560194	0.570100965616
C	4.400193998056	-3.480555628929	-3.235146573433
H	3.508661265345	-3.837239542260	-3.773691531358
H	5.213765453967	-4.205339165865	-3.397631229686
H	4.696565184177	-2.522427632726	-3.688533500058
C	3.577784284742	-4.963758501595	-0.664362326653
H	3.285059160876	-4.849498593335	0.391686588464
H	4.427090617613	-5.664984639519	-0.705395242707
H	2.732863485480	-5.420313998683	-1.202411049404
H	1.759836248903	-1.513191805792	3.101314693111
O	1.186415007974	-1.077506627824	3.752629599978
C	1.511091635880	-1.511989124182	5.041347347239
H	2.525552790298	-1.201624346151	5.354380406744
H	0.791705875099	-1.055733892263	5.738187180094
H	1.441016317185	-2.608384081148	5.156326795211

152
MeOH_Int_2, G=-4637.042902

Si	1.414883574163	0.844551182209	0.418447505961
Si	-0.535385817278	0.563762009036	-0.661796073579
Si	0.155057001095	-1.377443783995	0.214934787386
Si	2.476801594954	-1.164318020682	-0.171612860212
Si	3.721451999549	2.195959884585	1.970638023792
Si	-1.988176966819	3.134175426300	-1.665107575855
Si	-0.154736537312	-4.335813093243	1.252289822189
Si	4.341046956670	-1.954306987270	-2.447209235050
N	2.051195245850	2.201987905282	1.295772951394
N	-1.976235626619	1.428624659426	-1.086940191060
N	-0.776860126237	-2.699561468822	0.825433403025
N	2.819579263185	-1.195612168339	-1.948635719868
C	1.172578303112	3.271656686265	1.650522238310
C	0.192768103354	3.074614626936	2.646961162338
C	-0.656534943280	4.133673413560	2.975011711896
H	-1.414791355609	3.974375389161	3.747731239109
C	-0.559549044514	5.384761421666	2.360375690994
C	0.433227106557	5.559707709550	1.395992948147
H	0.534970022492	6.531393443877	0.903100146030

C	1.304534874766	4.529671255194	1.030097989109
C	0.080737885911	1.763538192382	3.362507769188
H	-0.658838148728	1.820047561098	4.172900324894
H	1.041161550326	1.436267293556	3.786469093844
H	-0.224626080819	0.950045383890	2.685612106761
C	-1.518254419656	6.488803713654	2.698554832978
H	-1.078762249598	7.478749482484	2.509084059323
H	-1.827217903653	6.446678485926	3.753314484166
H	-2.433603222254	6.413158925606	2.086920527919
C	2.372713540191	4.784915632780	0.007076489441
H	2.104343684367	5.632848403378	-0.637947599831
H	2.549078058792	3.904161533681	-0.624974877214
H	3.334597342103	5.029734025816	0.486816734434
C	3.830647243292	3.653375540530	3.147050605033
H	3.672674676768	4.626025001625	2.660677456833
H	4.827378294816	3.661712043679	3.616591403949
H	3.079774111514	3.555490064304	3.946469803891
C	4.990805594241	2.304311066304	0.591719370869
H	4.928605652976	1.410780953606	-0.048407119254
H	6.004067545351	2.328139401101	1.024821662444
H	4.868279744794	3.193186145933	-0.042939944483
C	4.052686801767	0.622810702710	2.923883321474
H	3.252274193699	0.391290295410	3.641457565410
H	5.009596205187	0.713817977429	3.463597544870
H	4.122653496447	-0.230794156812	2.234257750043
C	-3.278632627924	0.842213405954	-0.948471009412
C	-3.929009743955	0.262199079214	-2.054895203936
C	-5.267666179390	-0.116391619482	-1.921381179039
H	-5.776580875014	-0.547733347930	-2.789614666499
C	-5.973459262294	0.051576268553	-0.730765636465
C	-5.277267844142	0.545391333480	0.374065925534
H	-5.790686894515	0.637824676854	1.335315571539
C	-3.935350523189	0.919288303922	0.297778201920
C	-3.228630118678	0.022002973019	-3.359978885556
H	-3.287884400807	-1.043574001877	-3.630435586293
H	-2.168610357699	0.297123003409	-3.314792880149
H	-3.696552744954	0.588980198049	-4.179218969424
C	-7.421193556262	-0.334155553695	-0.639894737132
H	-7.880380278439	0.033329722963	0.288556851568
H	-7.544811324037	-1.429428090859	-0.659133104038
H	-7.996831118220	0.070199736484	-1.486594179247
C	-3.193693318384	1.347568020180	1.527330131935
H	-3.870100601592	1.419788120887	2.389820464572
H	-2.675512961667	2.307191648901	1.403387207687
H	-2.422106387927	0.600677690980	1.775638327486
C	-0.221745088880	3.620728885698	-2.012226036921
H	0.373153056972	3.602525597424	-1.090988650171
H	-0.194846889572	4.647858951854	-2.410150189933
H	0.243350147354	2.947143401319	-2.746391255003
C	-3.006369945908	3.282765597035	-3.233655022117
H	-2.546789939915	2.743263601436	-4.073889463735
H	-3.065682322691	4.349357806838	-3.505697860587
H	-4.033503552133	2.913501980669	-3.095577499120
C	-2.743741912223	4.254352668762	-0.367328324912
H	-3.760528733821	3.929623199398	-0.098110011114
H	-2.808741858962	5.280227976628	-0.765028863963
H	-2.122595904038	4.282693498655	0.540028324271
C	-2.170004881236	-2.505406998857	1.102897789479
C	-3.106023111287	-2.760875683753	0.077794607553
C	-4.464202355845	-2.772177689894	0.389291689028
H	-5.183105262480	-2.996812000401	-0.403448990574
C	-4.926653800464	-2.506572450826	1.680816485105
C	-3.986781332690	-2.161239010962	2.649249326649

H	-4.327552306478	-1.903598587617	3.657460601900
C	-2.611719995527	-2.144056685516	2.389551260460
C	-2.642719356414	-3.020599014871	-1.323592217126
H	-3.498654173054	-3.165506720768	-1.996803343114
H	-1.993247152714	-3.905676243815	-1.388760090379
H	-2.051182743094	-2.172853110532	-1.705081608224
C	-6.389299827699	-2.549461552587	2.013780990722
H	-6.812765212180	-1.534499871588	2.088061179650
H	-6.565143602435	-3.044773371895	2.980635603178
H	-6.962531255094	-3.088935914351	1.246626336636
C	-1.672825187262	-1.724401412356	3.483080914033
H	-1.993034671903	-0.756189762423	3.899265694463
H	-0.631823986542	-1.620144758740	3.154957390300
H	-1.681105067937	-2.442787272726	4.317970272689
C	-1.491317876908	-5.596958216729	0.859292470702
H	-2.448157116742	-5.336805267622	1.336456753603
H	-1.170308055540	-6.574778896395	1.253949021094
H	-1.665986955132	-5.713390179228	-0.220355190890
C	1.395278872590	-4.726836334592	0.278859769171
H	1.281546944438	-4.567073007796	-0.802299386543
H	1.638424954092	-5.789843428112	0.441793695135
H	2.243614248499	-4.128453186039	0.637396372947
C	0.233662939079	-4.452722993062	3.081042413837
H	0.891668146293	-3.628329997822	3.385842257053
H	0.755246857212	-5.403132681257	3.279026513807
H	-0.678557615781	-4.429405274188	3.694315596548
C	1.964063351652	-0.628921559224	-2.922945565812
C	2.130368412509	0.710526658656	-3.330636755328
C	1.288278919325	1.240415612637	-4.312241145420
H	1.440765343434	2.276481614461	-4.630801309300
C	0.265893673630	0.492155463309	-4.897745989352
C	0.104323741816	-0.827380901449	-4.469469616408
H	-0.692307579055	-1.438256104891	-4.905130302319
C	0.934184519114	-1.399975363093	-3.503170966083
C	3.201921816178	1.555401889641	-2.715885015916
H	4.208003933565	1.224782896837	-3.019670910976
H	3.175217467524	1.482533744514	-1.617337864075
H	3.092009305355	2.611201804786	-3.000259761575
C	-0.608641653121	1.076005829443	-5.969619584082
H	-0.695216819724	2.168136593239	-5.869098729820
H	-1.622168063984	0.649449626418	-5.940989999460
H	-0.198925169492	0.873075157179	-6.973505451938
C	0.740918562561	-2.822734715021	-3.084425844694
H	-0.140128698898	-3.267538961004	-3.566787530198
H	0.612923214617	-2.892974254904	-1.992375201090
H	1.619525408792	-3.439652912348	-3.329578226563
C	5.805465951920	-1.094103175469	-1.632285975170
H	5.884562125151	-0.040511981545	-1.940412279948
H	6.750890062628	-1.598298077911	-1.890453990808
H	5.699048263599	-1.116973381105	-0.535685469399
C	4.462256768854	-1.828425093346	-4.316133166585
H	3.617453819594	-2.338905204898	-4.803710741305
H	5.396167697591	-2.293184727704	-4.670147287452
H	4.450250033944	-0.780025083761	-4.651076331228
C	4.394308324814	-3.759757465028	-1.917004218366
H	4.280369722130	-3.847160178767	-0.825175835401
H	5.357306658072	-4.217778189922	-2.195737843918
H	3.587967055220	-4.347195785837	-2.381558933472
H	2.238630137769	-1.787388228193	3.543132750188
O	1.568595102839	-1.248776069663	4.014309178706
C	1.567324013654	-1.610303820507	5.360745055281
H	2.536187701823	-1.412613935101	5.860783258013
H	0.801353704477	-1.014452395916	5.881626360140

```
 H          1.318186848960     -2.677921972531      5.518561447193
 H          3.192028093710     -2.334654620958      1.676246090270
 O          3.285507022768     -2.782180875788      2.554998686444
 C          4.627130803231     -3.123863771274      2.764218619380
 H          5.290947380533     -2.240158429765      2.788051566802
 H          4.705726251323     -3.633636625852      3.735895871339
 H          5.002001580304     -3.816088661085      1.988668387177

158
MeOH_Int_3, G=-4752.503694
 Si        -1.807022739112      0.026979732334     -0.132825889563
 Si         0.111012249651      1.265032426930      0.060108051169
 Si         0.520784620033     -0.750489780564     -0.906137125393
 Si        -1.218059707134     -2.230103861132     -0.412972089212
 Si        -4.863217393045     -0.516412663931     -0.448282434154
 Si         0.431074760108      3.620662048989      2.189323348675
 Si         2.067658984550     -2.230507207698     -3.233462102746
 Si        -1.712836915485     -4.961752803817      0.862646173699
 N         -3.483350698867      0.593707219759     -0.156349336370
 N          1.035167253354      2.380370089674      1.058612876302
 N          1.924064364961     -0.989255796904     -1.951804436129
 N         -1.016673080874     -3.330226125202      1.011846557493
 C         -3.838837640999      1.962152287625      0.021756996914
 C         -3.899929368152      2.832226938954     -1.084424949659
 C         -4.291029403697      4.160862219844     -0.890233832221
 H         -4.331415291173      4.828648405066     -1.756843154726
 C         -4.663690294116      4.647318935084      0.363888547019
 C         -4.639501890699      3.755730063403      1.437522494725
 H         -4.933256312329      4.107715035874      2.431256976357
 C         -4.225803635433      2.430834364911      1.293605528985
 C         -3.629171832306      2.321055976005     -2.465998743528
 H         -3.500860447203      3.149249043325     -3.178149322787
 H         -4.472498879749      1.706791550801     -2.823848490080
 H         -2.741968312512      1.677512276053     -2.505344878023
 C         -5.052925853480      6.083595192897      0.561009518186
 H         -5.881610638422      6.181787676583      1.278104545792
 H         -5.362375425889      6.552678681378     -0.384093981769
 H         -4.208879797751      6.671468620020      0.959953071872
 C         -4.202005608777      1.518500229028      2.482007131690
 H         -4.252220425557      2.089881433691      3.419185835310
 H         -3.296788553399      0.894245092829      2.495057533209
 H         -5.056250091341      0.822828154559      2.471347632425
 C         -6.417209998299      0.538100041645     -0.540381679373
 H         -6.593364691814      1.100032097829      0.388834972694
 H         -7.281911485160     -0.124250994500     -0.708770342817
 H         -6.380933122717      1.266307989700     -1.364444152961
 C         -5.036874045336     -1.748236919046      0.951259269902
 H         -4.091768909125     -2.285625880301      1.100671135744
 H         -5.810738188884     -2.493194979406      0.707380225520
 H         -5.309981785337     -1.264436917778      1.900031832420
 C         -4.673853314591     -1.464511555411     -2.055492376639
 H         -4.382312412594     -0.807345601063     -2.887331297366
 H         -5.627564693200     -1.956743394903     -2.306681411004
 H         -3.897196498377     -2.236392293683     -1.944661444536
 C          2.424730739700      2.084391126864      1.289106832015
 C          2.795702957258      1.136317452706      2.263904101500
 C          4.146327428386      1.013365328205      2.605656061540
 H          4.423447997580      0.287671849108      3.377228759264
 C          5.136921353141      1.794438728258      2.013659163050
 C          4.750488917098      2.676530876532      1.003062773420
 H          5.512031520004      3.269348550884      0.488596884293
 C          3.420023361449      2.816334569738      0.608500553292
 C          1.795044991757      0.249531707652      2.938236554067
```

H	1.869377367850	-0.781925641394	2.562469487933
H	0.761460546508	0.568297807063	2.766738763386
H	1.968464551107	0.206962397835	4.022833397190
C	6.578023948783	1.645682151245	2.406342095121
H	7.160331243021	2.543795880297	2.153841486028
H	7.047230994950	0.793820921678	1.884692412838
H	6.686382858968	1.460702693590	3.485429278591
C	3.074368241193	3.710422143190	-0.544501898423
H	3.985539248952	4.035945169719	-1.063987552162
H	2.523727576174	4.607829397783	-0.229886587944
H	2.446125364920	3.171636164592	-1.266400981572
C	-1.415873802580	3.753058115892	1.990224717216
H	-1.757249869312	3.984646498089	0.971860454328
H	-1.803619390243	4.540996073152	2.655250049149
H	-1.887837313197	2.807997789185	2.287925886195
C	0.806364238042	3.181992084756	3.977714437568
H	0.256242826709	2.289550426173	4.308741185714
H	0.501534548298	4.026260004988	4.617462667532
H	1.880249954566	3.005605058518	4.139449687432
C	1.232059432024	5.297834505914	1.884288291659
H	2.327980363834	5.245008885932	1.970206942114
H	0.869912819655	5.995738830779	2.657050230130
H	0.977584001563	5.731394269826	0.905140061969
C	3.115921637305	-0.213752471503	-1.778888606329
C	4.045430768721	-0.621389521240	-0.792759688349
C	5.300069502428	-0.024749682405	-0.747319808962
H	6.015201505162	-0.369158743474	0.004856358519
C	5.671137139654	0.992108405314	-1.633285889615
C	4.719054044631	1.427288802058	-2.547925902326
H	4.968814835496	2.245338375817	-3.231506360726
C	3.444876488560	0.849874641620	-2.637166937108
C	3.696260187904	-1.720245264181	0.159297804572
H	4.470645442694	-1.837237726374	0.929517396587
H	3.576119760701	-2.680777237612	-0.364106498266
H	2.740287436881	-1.527131433448	0.670523855152
C	7.047455876287	1.588006809712	-1.584992757420
H	7.115753091978	2.497754759714	-2.198662961148
H	7.802579205732	0.877172025200	-1.959467574210
H	7.336324322952	1.849134500142	-0.555265183012
C	2.482099915685	1.383890862818	-3.654582312818
H	2.374448611299	2.475066322964	-3.541937719329
H	1.494343729273	0.925724149818	-3.549848727180
H	2.841088120668	1.206072992809	-4.681090235944
C	3.886160846225	-2.646322902155	-3.456229638055
H	4.473712904557	-1.760364091180	-3.740448866863
H	3.986746675041	-3.397807381074	-4.256303521362
H	4.330638328362	-3.063527947193	-2.540372371896
C	1.129165115207	-3.788332508866	-2.789197450335
H	1.543329292636	-4.266826378223	-1.890048641984
H	1.211366127890	-4.505646439154	-3.622194913568
H	0.064324067059	-3.592126429829	-2.595588880749
C	1.408126418380	-1.600111015022	-4.881410077523
H	0.423706733614	-1.135113124135	-4.737643500336
H	1.296083650662	-2.440681289424	-5.585238171962
H	2.082911365487	-0.862766277408	-5.338184241955
C	-0.231937614021	-3.050640555647	2.161832612060
C	-0.788308022612	-2.351125923114	3.253161683090
C	-0.030470480041	-2.161582130567	4.410836696410
H	-0.482982515955	-1.625782205856	5.251417485362
C	1.274608943775	-2.641659233894	4.531381181027
C	1.816129790285	-3.319522111169	3.438654288646
H	2.839511202421	-3.703620775438	3.499723853705
C	1.092217905802	-3.529747476579	2.262030831985

C	-2.200614274471	-1.863377416801	3.186112306232
H	-2.910406295718	-2.702588740757	3.111257826752
H	-2.359562146132	-1.246813218748	2.288186950978
H	-2.463065996768	-1.268912856423	4.072461208300
C	2.086722991351	-2.390925015278	5.768183234830
H	2.694993258817	-1.475112652367	5.665102280133
H	2.782055540575	-3.218993392376	5.971058534271
H	1.446636766647	-2.257692074456	6.652619703803
C	1.705106236865	-4.305382700851	1.137421216448
H	2.798244935622	-4.347424613834	1.234088229488
H	1.446080048786	-3.859203905562	0.168311432011
H	1.336508713166	-5.343734959324	1.119245707492
C	-3.577803835987	-4.925345295290	0.610128559487
H	-4.107971632855	-4.521725155980	1.485068710927
H	-3.937332291919	-5.953560003334	0.439041103623
H	-3.853357826988	-4.320953808755	-0.267597130299
C	-1.365497637268	-5.885463822721	2.462833766026
H	-0.291079533547	-6.021872070011	2.653407952036
H	-1.838430878719	-6.879962351368	2.425654558581
H	-1.783494856314	-5.339352789772	3.322940274611
C	-1.029052850915	-5.910406355156	-0.615639189051
H	-1.271997466753	-5.380915867920	-1.549834644715
H	-1.493103545658	-6.909356628532	-0.662753197219
H	0.061514637732	-6.040332826979	-0.579945518987
O	0.027777448174	2.366152248212	-1.517517458446
H	-0.318724615826	1.845338091934	-2.328749623313
C	-0.354210346194	3.733089967424	-1.554090344733
H	-0.057204095194	4.162240916039	-2.521550718560
H	-1.438693510474	3.844507709526	-1.403248131007
H	0.187613810443	4.249686242417	-0.754070978812
H	-1.143943890119	0.180888661460	-3.515720747701
O	-0.795564083100	1.113348531687	-3.570901877142
C	-0.955557094725	1.630792623218	-4.864569177452
H	-2.020973557758	1.726660722517	-5.138007500405
H	-0.499837364267	2.631160512205	-4.901104348935
H	-0.448951000925	1.001779746414	-5.616350584948
H	-1.708241787279	-1.776552221632	-2.632277197756
O	-1.747095089074	-1.331201990839	-3.517549547962
C	-2.133528057910	-2.249343684674	-4.505507333588
H	-3.162226275277	-2.613844101621	-4.341242374556
H	-2.099921053625	-1.741290925022	-5.480490479665
H	-1.453493437349	-3.117392153236	-4.550013218998

158
MeOH_TS_1, G=-4752.500312

Si	-1.797188266895	-0.003859416537	0.087089846161
Si	0.032431573860	-1.387851379960	0.084847565028
Si	0.608899629561	0.683593731920	0.836842295018
Si	-1.110736948381	2.203189299823	0.441149418587
Si	-4.850545934001	0.680073107342	0.413984851853
Si	0.264524717813	-3.731567039775	-2.072785022252
Si	2.230108777356	2.111413223528	3.171870810652
Si	-1.587734944900	5.014204603530	-0.721987446282
N	-3.508818786233	-0.475393865713	0.142303275935
N	0.911048407248	-2.472562400442	-0.994139152182
N	2.033773656224	0.903307163772	1.875136113332
N	-0.931044501839	3.375711720911	-0.929033866061
C	-3.916603624116	-1.830888775223	-0.024715344814
C	-3.989238003718	-2.690697121789	1.087556636603
C	-4.442839015688	-4.001369514714	0.915423436516
H	-4.491840686982	-4.660922297343	1.787832231831
C	-4.860395806945	-4.480597721448	-0.327364496971
C	-4.809090303345	-3.602591516313	-1.411051147460

H	-5.127948369350	-3.952821815700	-2.397642180723
C	-4.334402662576	-2.296024560129	-1.287566789349
C	-3.650552147817	-2.179587132610	2.453474450101
H	-3.541368624284	-3.007389063732	3.169045926584
H	-4.444405209611	-1.513598468416	2.831447713703
H	-2.730241014693	-1.583682906792	2.439405295285
C	-5.313731158280	-5.900555611450	-0.503869012710
H	-6.130590023338	-5.975116879684	-1.237021349362
H	-5.664548199119	-6.334718621328	0.443586191136
H	-4.489455255878	-6.535293558205	-0.871266536252
C	-4.268082234892	-1.402939591355	-2.489123546735
H	-4.352766099892	-1.984765403936	-3.417337384481
H	-3.328395623947	-0.831156605152	-2.514492455314
H	-5.082434759713	-0.661001855735	-2.486809228067
C	-6.441043613965	-0.318273268417	0.513870279466
H	-6.639096223956	-0.879927354904	-0.410980870530
H	-7.282406970303	0.373647241260	0.681646610585
H	-6.425494152230	-1.043373771941	1.341646123077
C	-4.977122040380	1.910732585554	-0.991282620726
H	-4.020943629260	2.432517620161	-1.122164197201
H	-5.744745459725	2.667424477007	-0.764539270263
H	-5.237025986565	1.429112978046	-1.944685829439
C	-4.664072575700	1.650133842705	2.011792789840
H	-4.479789147856	0.994149442491	2.874755320952
H	-5.588938156872	2.221868897817	2.193319492254
H	-3.824715252392	2.356888079200	1.927063717291
C	2.278862092930	-2.157472792183	-1.300413044858
C	2.588804097877	-1.201372411287	-2.288483514487
C	3.918802697309	-1.061546813716	-2.699318899850
H	4.147278269683	-0.329431556834	-3.480850667051
C	4.947524189850	-1.834331583418	-2.164315964003
C	4.623967933031	-2.725441399419	-1.139286510067
H	5.417244079648	-3.313689824307	-0.669216202889
C	3.317542046811	-2.881215504611	-0.677220157508
C	1.543617431904	-0.326149244194	-2.908675524409
H	1.623125285502	0.704424704199	-2.530961179685
H	0.525622969976	-0.661493739791	-2.682155417316
H	1.662361073099	-0.277182755682	-4.000709780415
C	6.365441972087	-1.670369501016	-2.629012732961
H	6.945403243030	-2.594656654616	-2.489533255125
H	6.881154725577	-0.874615480496	-2.064269298880
H	6.413607808872	-1.394317530056	-3.692706214205
C	3.038962090043	-3.788361224403	0.483963821874
H	3.971323815503	-4.034753926196	1.009426064689
H	2.562323305341	-4.730092311504	0.177880653545
H	2.363336092962	-3.297146622160	1.196551030213
C	-1.579269409416	-3.831376092438	-1.828836678869
H	-1.902136552881	-4.033753660319	-0.797895077587
H	-1.995972147835	-4.625151057105	-2.468852005550
H	-2.038839053404	-2.883676107024	-2.136427741562
C	0.600409909250	-3.331273832612	-3.879346626764
H	0.053583779361	-2.436815698878	-4.210729381437
H	0.270514812992	-4.181180815122	-4.498863733528
H	1.672100890963	-3.169209752271	-4.069106170723
C	1.041006058941	-5.420567153150	-1.764492839269
H	2.130503370550	-5.400489979340	-1.918801649064
H	0.614627489487	-6.131396691842	-2.491543306099
H	0.835388214015	-5.813816107170	-0.757389657905
C	3.210219684756	0.108049651886	1.678757177151
C	4.138380055309	0.510057075886	0.689219329113
C	5.382638977909	-0.106219049645	0.629108407227
H	6.098030445090	0.235171927377	-0.124312976243
C	5.744519717793	-1.138385524882	1.501080342139

C	4.792570274501	-1.567872684661	2.418433647022
H	5.033534674320	-2.397836989942	3.090697037237
C	3.527725270080	-0.972307285709	2.520390936827
C	3.799405295201	1.620340245730	-0.252439584335
H	4.567102701714	1.726929276352	-1.030718688568
H	3.703831897358	2.579683764792	0.277630275200
H	2.834198905353	1.446409723480	-0.753273085379
C	7.105250542806	-1.765726516137	1.421472891942
H	7.203428003467	-2.608847195700	2.120118452273
H	7.896564833133	-1.037112744534	1.661599629364
H	7.313522530646	-2.140752560300	0.406576328457
C	2.555792505666	-1.510549360611	3.526898731899
H	2.420147521650	-2.594387725459	3.381830848463
H	1.583162563515	-1.021631387800	3.427610540298
H	2.917796343451	-1.368236569623	4.557839476823
C	4.053969206971	2.518248653116	3.360613965940
H	4.645870899830	1.623827134361	3.606629739654
H	4.177877339021	3.250056146562	4.175561310383
H	4.474620498099	2.956708907940	2.443443297492
C	1.288794641085	3.691015418967	2.813980776767
H	1.729104522842	4.229977143557	1.962892104771
H	1.346894072105	4.351430373847	3.694733156869
H	0.230200876100	3.513337818038	2.571865520999
C	1.630515693714	1.448749136335	4.836935448262
H	0.659236129522	0.946322849789	4.736597875639
H	1.527401709774	2.276867469982	5.556981956133
H	2.340606298140	0.723769697041	5.259162192958
C	-0.154352260680	3.112065864363	-2.090898303723
C	-0.729361052112	2.470383058813	-3.208001010622
C	0.032609088662	2.279446041473	-4.362199012877
H	-0.432832127084	1.785511173683	-5.221064635075
C	1.362725973530	2.693531735147	-4.451795333345
C	1.919697584394	3.320434893637	-3.337195527848
H	2.960883311439	3.656290494562	-3.375161156469
C	1.188395275324	3.538949235893	-2.166330079091
C	-2.158024651400	2.030703284139	-3.166413078322
H	-2.839178712807	2.889336945317	-3.056608745610
H	-2.339499867859	1.379242343992	-2.297776035268
H	-2.436749833285	1.484852963085	-4.078619964664
C	2.180711841867	2.420334697973	-5.679810679859
H	2.672920144286	1.433975265775	-5.614887430546
H	2.973793869887	3.170583531857	-5.814186006699
H	1.559257396063	2.412709656620	-6.587471573841
C	1.815845584610	4.263087177306	-1.016431821125
H	2.907118708922	4.313594520676	-1.126936805497
H	1.572656642168	3.769197891490	-0.066378681450
H	1.443393637348	5.297394333210	-0.943098053551
C	-3.455441358702	5.022166082567	-0.484556836531
H	-3.989762929083	4.676757756449	-1.381487147454
H	-3.785575050559	6.051623720521	-0.267753722846
H	-3.758181868747	4.385696808643	0.360871107086
C	-1.202176374690	5.992320620927	-2.280331968525
H	-0.122722527313	6.104077552138	-2.458307421168
H	-1.645908614510	6.998133110945	-2.205905589262
H	-1.629400304777	5.494024479052	-3.164568857303
C	-0.895467628633	5.887691573009	0.799020331353
H	-1.175343388170	5.337713043083	1.710874225321
H	-1.322509439429	6.901528305579	0.871451930412
H	0.199676586763	5.976385790882	0.786166450565
O	-0.158913551841	-2.323690032032	1.582957381023
H	-0.520400685811	-1.458248708925	2.711615042085
C	-0.404242239925	-3.705796578846	1.659704839406
H	-0.091593303184	-4.075108605368	2.649883106470

H	-1.474459238056	-3.934444812566	1.515591340337
H	0.179184424574	-4.244092501721	0.897997304344
H	-1.252134390215	0.215204081066	3.431449465703
O	-0.724178745454	-0.870829299856	3.553589171127
C	-0.896439482798	-1.610905368643	4.740863819971
H	-1.863518210150	-2.138580306565	4.753666142643
H	-0.080327787840	-2.340973081347	4.842939072138
H	-0.847192517912	-0.924016904570	5.597874310125
H	-1.642580448952	1.767004898274	2.442903248533
O	-1.796739688970	1.263120423450	3.339400573872
C	-1.950154734959	2.145801066924	4.433243286869
H	-2.731189883881	2.879890137436	4.190049051890
H	-2.272990955626	1.564164772175	5.307468912966
H	-1.011676148136	2.669633593898	4.667515775059

158

MeOH_Int_4, G=-4752.561463

Si	-1.836426748018	0.055953179067	0.172966285501
Si	-0.081557799838	-1.398343385923	-0.125219542030
Si	0.604371100259	0.376921366850	1.138730720185
Si	-0.767651364640	2.084112749946	0.464224386035
Si	-4.743919609512	1.038754676770	0.616865466612
Si	-0.021666589281	-3.036161165899	-2.843697661636
Si	2.181377691842	1.304043423110	3.684951189296
Si	-0.996389520036	5.120173736834	-0.041934216440
N	-3.566753347219	-0.210430395042	0.089612204471
N	0.717090559692	-2.135836853303	-1.498058501135
N	1.993419545127	0.321874890269	2.195436526544
N	-0.484185206954	3.488954228054	-0.573998244310
C	-4.111705174049	-1.471411348957	-0.295739561433
C	-4.232813541338	-2.502076194809	0.656388168094
C	-4.789789531979	-3.723587630278	0.269391652679
H	-4.877531586230	-4.520191169338	1.014856409884
C	-5.257598053811	-3.946315531725	-1.027740616317
C	-5.163649141513	-2.896040181042	-1.941271298687
H	-5.529305837001	-3.041388401369	-2.962427270937
C	-4.596161625837	-1.665707041107	-1.603053863081
C	-3.820323861249	-2.267961129390	2.076100217482
H	-3.926666434813	-3.184634549530	2.672799535092
H	-4.441675028693	-1.487714498946	2.545062007773
H	-2.784034893416	-1.913937513830	2.163056781994
C	-5.817365163900	-5.278698583070	-1.433792907681
H	-6.601915210225	-5.171318202201	-2.197160488965
H	-6.246617650303	-5.815701258265	-0.575435277939
H	-5.031569264680	-5.923053391979	-1.863307990365
C	-4.512770441641	-0.573418883582	-2.626298608292
H	-4.581499533864	-0.980957541373	-3.644402137503
H	-3.578030626516	-0.002288870432	-2.534858732911
H	-5.334589589223	0.151305037856	-2.505940983557
C	-6.448864623810	0.254327876963	0.588486332176
H	-6.741123990071	-0.077828273834	-0.418561284455
H	-7.187999073959	0.995450012870	0.933865132984
H	-6.504984375167	-0.618861018279	1.256032999616
C	-4.692249076902	2.468517395689	-0.593084556257
H	-3.664848787318	2.835938525900	-0.713894696170
H	-5.309317826735	3.307417173122	-0.236037286016
H	-5.060160885602	2.164947206086	-1.583719227306
C	-4.350864665044	1.620254179567	2.354445275935
H	-4.799882067585	0.935384907389	3.091410810461
H	-4.772243378628	2.623086921158	2.530267144762
H	-3.269792642965	1.650045879167	2.562260682804
C	2.131356585250	-1.939150217997	-1.664779312829
C	2.608858450494	-0.855610196548	-2.426262632948

C	3.972759653688	-0.789203603513	-2.732132799386
H	4.329403845629	0.049475322623	-3.339066711914
C	4.876169441855	-1.759439993702	-2.305876900558
C	4.389552042801	-2.785967087801	-1.493074059194
H	5.082862417447	-3.537952489777	-1.105939093801
C	3.044076558497	-2.881181382589	-1.140932211314
C	1.704617520658	0.233262169543	-2.912074772837
H	1.989928969320	1.197861136196	-2.469101934686
H	0.657410780370	0.051600011091	-2.648559282979
H	1.771903503331	0.355450796034	-4.002779849277
C	6.333436367987	-1.672992286300	-2.654885322241
H	6.798811673538	-2.668994216073	-2.693260821165
H	6.889065803622	-1.083878640602	-1.905201019354
H	6.488109736597	-1.185869268116	-3.628776575615
C	2.595878070732	-3.962319925021	-0.203765480956
H	3.463088299723	-4.440988442220	0.270443498130
H	2.014003027599	-4.741916091959	-0.715943672264
H	1.958575199248	-3.547245238430	0.588410729297
C	-1.872006602950	-3.032126104000	-2.608942701262
H	-2.220090165770	-3.453661029064	-1.655802106394
H	-2.346803527814	-3.610661438842	-3.417338540209
H	-2.251322390429	-2.003818422446	-2.674451502779
C	0.360626135452	-2.246186195979	-4.505791954545
H	-0.095370765913	-1.250786818087	-4.605030606066
H	-0.047647718351	-2.889693868417	-5.302214645302
H	1.443963320490	-2.149934586691	-4.672305575197
C	0.628273768548	-4.800136452606	-2.943631760436
H	1.725260730618	-4.815382155163	-3.034608042667
H	0.211286783942	-5.278921303455	-3.844699402194
H	0.340893594923	-5.414961806174	-2.077576144244
C	3.127536964552	-0.490799764126	1.866490673927
C	4.092957189345	0.035684893918	0.976023814044
C	5.294877621100	-0.638620298068	0.797010525319
H	6.042075084416	-0.205112870977	0.126500524623
C	5.572053808311	-1.846111004141	1.446914413058
C	4.580572250401	-2.381366273710	2.261072860446
H	4.756389829499	-3.341347074714	2.757215235749
C	3.357916762649	-1.731819000033	2.483335372648
C	3.831878503669	1.319784403271	0.255058052216
H	4.650341508475	1.555972974669	-0.438436603962
H	3.711906503536	2.159877859738	0.954389938554
H	2.902429558716	1.267997708257	-0.333851105049
C	6.886450502342	-2.540924816443	1.244043592864
H	6.938802491812	-3.480255898890	1.812565653216
H	7.728166161729	-1.906115793434	1.564439469100
H	7.052858402995	-2.779685638230	0.181202927384
C	2.339061031738	-2.393904221305	3.360581725625
H	2.072728991320	-3.381221287468	2.949420377328
H	1.424169787621	-1.799496459394	3.440710834684
H	2.733749790475	-2.565347358243	4.374574041769
C	4.001108203226	1.726621440437	3.887225630221
H	4.621083917805	0.818392888917	3.933491276591
H	4.135271072267	2.279403185052	4.831281327274
H	4.383364016797	2.355939101594	3.070035683662
C	1.148821266839	2.851850516675	3.521876761216
H	1.378955182476	3.406473552974	2.600765375132
H	1.354251456102	3.515406197218	4.377645498380
H	0.079168232665	2.592750056998	3.534372639664
C	1.621717232697	0.363514108951	5.209969080961
H	0.607304330714	-0.026121341705	5.048630976008
H	1.595031366846	1.051130864867	6.071063151702
H	2.292533527384	-0.470453050063	5.459453858633
C	0.247377503852	3.401165188872	-1.792755786799

C	-0.400047644153	2.998853815614	-2.979157720860
C	0.311932096763	2.989321436603	-4.179312642694
H	-0.204117712585	2.677846221502	-5.092828769283
C	1.655697762855	3.363415624173	-4.247940050933
C	2.280666669056	3.752973944028	-3.063964510206
H	3.335234560734	4.044951239921	-3.086108144110
C	1.605316976771	3.776160510528	-1.839608601780
C	-1.847681729582	2.617677074978	-2.955630202068
H	-2.478316695392	3.460427131077	-2.631484299557
H	-2.033394586667	1.800917892140	-2.240395480781
H	-2.190921591779	2.292656339049	-3.947468619770
C	2.414672509025	3.287381536842	-5.540083636954
H	2.817968172149	2.272155724878	-5.698888608231
H	3.266744030311	3.982388894041	-5.551481761678
H	1.771654366254	3.518772133843	-6.402065057487
C	2.322393510257	4.234904174700	-0.606492613582
H	3.407953445413	4.106622928716	-0.711351535395
H	1.984732796624	3.683442068591	0.280651376823
H	2.136893279735	5.303769450056	-0.411174273546
C	-2.841755988681	5.229520746945	0.296017277934
H	-3.441003144888	5.089171011870	-0.614794233463
H	-3.059180105089	6.235432973263	0.691588379656
H	-3.173604199356	4.496920118369	1.046466920718
C	-0.604172558413	6.304460543402	-1.446280526415
H	0.471479989924	6.378475284681	-1.661008666649
H	-0.973274555657	7.309641801334	-1.186546654726
H	-1.103425839765	5.986478409986	-2.374776468400
C	-0.156721334883	5.675293489051	1.547286728693
H	-0.441567691383	5.028584072894	2.390538313941
H	-0.488640172736	6.699420892887	1.784811070774
H	0.939970832273	5.682080534513	1.481871181511
O	-0.324428103043	-2.652575093159	1.039484150106
H	-0.651465666647	-2.079512279440	2.621030433047
C	-0.797848002414	-3.942916979765	0.756147776464
H	-0.568928903963	-4.599521262159	1.610533334533
H	-1.889606962889	-3.948689194334	0.592105880847
H	-0.305777163975	-4.364474623307	-0.134535405718
H	-1.321833454994	-0.040378768697	3.678861994422
O	-0.887439617794	-1.746107017660	3.518475250851
C	-1.046228308980	-2.811188596239	4.410241149655
H	-1.857914486452	-3.496547157718	4.104039899765
H	-0.118681578856	-3.400783829411	4.524775760078
H	-1.306749361020	-2.402014695281	5.397695136517
H	-1.404104532261	2.736615235401	1.643611628371
O	-1.560949910912	0.870734637875	3.941240077898
C	-2.221479805474	0.806426496695	5.166467590112
H	-2.561998261876	1.818491965614	5.435440194367
H	-3.116726933602	0.154567427723	5.138866097360
H	-1.569000080579	0.448620717800	5.988229594547

158
MeOH_Int_5, G=-4752.563906

Si	-0.091273969171	1.192729691682	1.125559802570
Si	0.722719113453	-0.975361974035	0.845481065221
Si	1.032635571447	0.176155049575	-1.123778774635
Si	-0.763833738939	1.583132898027	-1.054316535976
Si	0.013163168011	3.980660941743	2.693124258320
Si	1.207035767343	-3.821372870841	1.776974972290
Si	2.085418245409	0.873295933417	-4.010639545648
Si	-2.781804374918	3.180457659629	-2.728383603845
N	0.682230705067	2.423583670807	2.151430812947
N	0.038695366597	-2.506608074978	1.413156356609
N	2.053575262064	-0.135117029202	-2.546484761090

N	-2.392658750495	1.706340900111	-1.799692512450
C	2.102806148670	2.297218049352	2.310181985504
C	2.994514071034	2.853388445425	1.369600212829
C	4.373500360501	2.784479288013	1.611053532542
H	5.052210127822	3.225724142253	0.874278855956
C	4.903742188738	2.167180295989	2.739430438510
C	4.006004039509	1.573793011097	3.631682885786
H	4.392769559529	1.041298312115	4.506070596257
C	2.627508809959	1.613320891918	3.432434552023
C	2.538066119212	3.481894720813	0.087473643710
H	2.917029318255	2.902417762546	-0.766275990345
H	2.922673706735	4.508173136893	-0.015481290139
H	1.447465082097	3.502205216516	0.000573848522
C	6.383715043529	2.103717982368	2.981154881642
H	6.659031546208	2.614766562718	3.917792577471
H	6.945921171321	2.575920019639	2.163210584209
H	6.729032870702	1.061221059787	3.070535256079
C	1.727581536391	0.873755137959	4.374404710644
H	2.314962176658	0.337043724379	5.131642457086
H	1.133443926259	0.127925115490	3.823918354349
H	1.032593602536	1.544879394571	4.898621561681
C	0.114507337577	4.121362952489	4.571359310657
H	-0.514991147214	3.391923148959	5.102325871449
H	-0.210776228198	5.127390045923	4.882198377872
H	1.154598963577	3.989631598450	4.909520432097
C	-1.763818108423	4.156692505649	2.111415964769
H	-1.842016186144	3.926760438125	1.038111640412
H	-2.074695611954	5.204322732736	2.251398808224
H	-2.472371484130	3.514457714013	2.650350970578
C	0.963835444440	5.461324957440	2.029617705625
H	2.041664211833	5.386616644371	2.236520219910
H	0.585394543850	6.364721783228	2.535481926913
H	0.824828688219	5.596807065819	0.947825485888
C	-1.339906273106	-2.822826761067	1.494561279088
C	-1.969007457645	-2.940554988633	2.750367708543
C	-3.295361466743	-3.378773332846	2.819841755097
H	-3.761984755101	-3.486956780817	3.804774327434
C	-4.036021077566	-3.683920652181	1.680075558831
C	-3.403418517334	-3.544134266121	0.441290468432
H	-3.948620595994	-3.797143883504	-0.472772385886
C	-2.074507164710	-3.136043948317	0.329641728342
C	-1.220035259276	-2.663005122369	4.020125832618
H	-1.889969400812	-2.272879476959	4.801406859863
H	-0.395759515253	-1.956497173290	3.853876924898
H	-0.765455376536	-3.582471249362	4.423779196501
C	-5.463641277474	-4.142399366301	1.770898415516
H	-5.641235157928	-5.032863794975	1.147860597838
H	-6.164033174181	-3.366313502350	1.417833355613
H	-5.741242520495	-4.394188783600	2.804585719049
C	-1.401483881835	-3.102270469689	-1.005332600048
H	-2.078341520509	-3.436610444100	-1.803139074080
H	-0.506298039792	-3.743950753448	-1.009463139783
H	-1.053686444302	-2.086829179715	-1.246113278898
C	2.490725137820	-3.970803307102	0.407150366788
H	2.211383859514	-3.385176075051	-0.480968202252
H	2.594803069911	-5.020159253902	0.088121043264
H	3.481064770528	-3.616877104962	0.727670682785
C	2.069516845502	-3.703537577535	3.448617975299
H	2.458344509535	-2.687807944477	3.604156809474
H	2.919424130723	-4.406043457899	3.459905041983
H	1.409973760792	-3.967563647697	4.287748513782
C	0.190736397733	-5.412071541995	1.779129784218
H	-0.588664016643	-5.419327751321	2.555882762077

H	0.862725749066	-6.266618416636	1.962733145452
H	-0.308451144207	-5.574248539930	0.810931706667
C	3.011405042632	-1.189318037478	-2.482816860383
C	2.665097957750	-2.479503569994	-2.932458657157
C	3.621098045536	-3.495304878791	-2.891122605087
H	3.340441517186	-4.495803082680	-3.234510485297
C	4.913342908581	-3.275908868745	-2.409673141309
C	5.240652470541	-1.987202793972	-1.986191595375
H	6.250104807708	-1.786855482813	-1.613592716201
C	4.317182898994	-0.938385293610	-2.017696574606
C	1.293355562715	-2.747068081573	-3.472633747271
H	1.095796566605	-3.825944366803	-3.539631777922
H	1.173027189229	-2.320110623445	-4.481894821407
H	0.520144967958	-2.284823291043	-2.844727955573
C	5.900735259064	-4.402152729948	-2.312142477192
H	6.936210382259	-4.032743688282	-2.290338081533
H	5.803665844575	-5.099681209870	-3.157486726537
H	5.738966670640	-4.985212680105	-1.389602030479
C	4.722858865015	0.439714469008	-1.586697824392
H	5.619797149200	0.410396555295	-0.952384732431
H	3.919413747308	0.939474947779	-1.029027407319
H	4.952564357025	1.077394582633	-2.456746097760
C	3.496212319967	0.276954337634	-5.097722371925
H	4.477801006643	0.373983771789	-4.611781551207
H	3.512412593206	0.869994529479	-6.026484604771
H	3.366301317421	-0.781579984958	-5.370570032639
C	0.490496136340	0.706942055790	-4.981673175497
H	0.326099073744	-0.329556366654	-5.311287817927
H	0.516340143602	1.355563997166	-5.871839794169
H	-0.367097075622	1.010116904096	-4.369801843742
C	2.324763504730	2.685449551758	-3.573269966732
H	1.567778410232	3.021330042609	-2.848515493738
H	2.233494484639	3.310404511820	-4.476235615846
H	3.316910406535	2.864884723762	-3.132773278794
C	-3.405802645585	0.725759758301	-1.657597165817
C	-4.486589378242	0.942339412581	-0.776628678582
C	-5.522237208990	-0.001128477917	-0.705284619190
H	-6.362206646690	0.191508367916	-0.030205415352
C	-5.515640238407	-1.169560221021	-1.467834189000
C	-4.430213667790	-1.373090026477	-2.323653438488
H	-4.398731055211	-2.276199748952	-2.940893114466
C	-3.390487474798	-0.450494617591	-2.441536832903
C	-4.533902976611	2.167949085238	0.084856827236
H	-4.809641770871	3.060362801930	-0.497322353789
H	-3.554515864554	2.377232423325	0.536243295667
H	-5.281945931672	2.063558005600	0.882934629610
C	-6.638293333481	-2.162291189951	-1.395367373104
H	-6.264573610528	-3.180854107332	-1.208542762288
H	-7.200940276099	-2.194186330295	-2.342421418790
H	-7.349226907241	-1.911014120593	-0.595364159101
C	-2.300665374578	-0.671452342600	-3.439741151059
H	-2.358642397888	-1.674606161954	-3.883212479965
H	-1.306630642431	-0.544504900566	-2.987489421472
H	-2.369152965834	0.066682297287	-4.254370139173
C	-2.703023801996	4.746441574220	-1.682153855103
H	-3.354107848892	4.708641914639	-0.797600175373
H	-3.012480334970	5.608736181273	-2.295247381677
H	-1.675274720371	4.936283364818	-1.337131447137
C	-4.505481584445	2.958728155150	-3.449131847643
H	-4.551337700474	2.045100124541	-4.062452400780
H	-4.735179290854	3.816277129290	-4.101912461588
H	-5.297732685061	2.886211686443	-2.690592620453
C	-1.620290326433	3.451210913267	-4.181374441274

H	-0.563190979130	3.477522628026	-3.881287180089
H	-1.861100815079	4.420616010416	-4.648129721771
H	-1.740418100261	2.671161650457	-4.947186636575
O	2.187306544429	-1.240197457450	1.652224256445
C	3.470416866234	-0.822761676260	1.295488643835
H	4.155888900649	-1.058494164398	2.124955999137
H	3.823788842400	-1.345639198187	0.392290000391
H	3.519006921737	0.261518174360	1.117372264335
H	-0.283213068194	2.960236589540	-1.417776136723
O	-1.699244011524	1.114192364708	2.203171819775
H	-2.436001001212	0.506676899019	1.842788980574
C	-1.711517331961	0.984340427606	3.618020905299
H	-1.635101255260	-0.071943014040	3.908666559730
H	-0.852190477390	1.531485720873	4.011545702239
H	-2.639506506116	1.420936610511	4.016171775555
O	-3.602531641671	-0.431002004200	1.680123226063
H	-3.902677719889	-0.551070374907	0.763475733416
C	-4.700176169466	-0.440007839402	2.556566102911
H	-5.396361178385	-1.252309852046	2.303884280908
H	-4.323291364772	-0.629300271716	3.570716438764
H	-5.247346236116	0.519443372593	2.550318931835

158
MeOH_TS_2, G=-4752.544123

Si	-0.314872816560	1.699717907678	-0.810202371470
Si	-0.820520092262	-0.571800375116	-1.241058447623
Si	-0.836480399522	-0.293624345793	1.068841424168
Si	0.916619183562	1.160274602464	1.123873614410
Si	-1.098769653652	4.710867020866	-1.160254761531
Si	-1.115973092023	-2.968329467947	-3.104682436254
Si	-1.456690833858	-0.945732697442	4.124139942892
Si	3.173084932462	2.339178535142	2.880435720923
N	-1.525275800565	2.991188170753	-0.989522268694
N	-0.070620468723	-1.703048188426	-2.381812106266
N	-1.453646291942	-1.330622251420	2.382352986907
N	2.853009578758	1.283429356201	1.414452248123
C	-2.905136078480	2.696871872239	-0.791058800931
C	-3.478263022809	2.771006726281	0.495592232965
C	-4.844184096941	2.522059112387	0.648924255836
H	-5.277053687458	2.574291707922	1.652893180031
C	-5.671138466285	2.226852026827	-0.437110274929
C	-5.087990452970	2.180858903582	-1.703804870736
H	-5.712584134465	1.950673456596	-2.572542154681
C	-3.720951019762	2.392353876682	-1.898313721543
C	-2.644181586115	3.151270297767	1.679294282299
H	-3.200940191369	3.016192698773	2.615752738075
H	-2.330243698701	4.205849907646	1.627403003009
H	-1.727979175226	2.544746170203	1.722778464414
C	-7.129705146503	1.930836335490	-0.239205542342
H	-7.705442780226	2.101567442744	-1.160329726667
H	-7.564925218638	2.553452346798	0.556720832740
H	-7.283745210842	0.877830936761	0.052810381368
C	-3.124212797888	2.252646039333	-3.265438308125
H	-3.903300168319	2.080396183694	-4.020801478641
H	-2.435154581296	1.392359933074	-3.287915728336
H	-2.550974720404	3.144951037303	-3.556074232331
C	-0.458237055987	5.240157865193	-2.854471382671
H	0.577916729860	4.919801951124	-3.026281559145
H	-0.476947528774	6.341822468026	-2.893516241020
H	-1.081662382799	4.872475521384	-3.683436018062
C	0.224939489131	5.165001825433	0.093391126067
H	-0.103630677576	4.940344062827	1.118828798177
H	0.471195414568	6.237156303608	0.033395543147

H	1.148312904971	4.598477944855	-0.098562092152
C	-2.662257387509	5.719310577063	-0.882303915881
H	-3.421002501804	5.472936090078	-1.641407321792
H	-2.421533052711	6.790477305102	-0.977876822595
H	-3.120957818259	5.556494263274	0.102740553876
C	1.321759750663	-1.764611104763	-2.633048260872
C	1.855812971828	-1.300935833964	-3.853645595713
C	3.239464840710	-1.359400302689	-4.064280762713
H	3.642812613639	-0.978028937605	-5.008070726071
C	4.112827020373	-1.888709246817	-3.115637982379
C	3.558339809698	-2.406766503962	-1.941498987664
H	4.212737585121	-2.855756328825	-1.190526381384
C	2.188522855439	-2.365330997593	-1.690421761760
C	0.963638598102	-0.808220192526	-4.955226371683
H	1.466269889522	-0.049190748339	-5.572673237891
H	0.024136024015	-0.398128234992	-4.563622020439
H	0.689899574253	-1.638348743855	-5.627915406579
C	5.598676049349	-1.906584595738	-3.329372301444
H	6.004375161932	-2.927447381595	-3.244979573769
H	6.114474464421	-1.294653346709	-2.570359984797
H	5.871500587427	-1.517460777753	-4.320611642619
C	1.624877856293	-2.992411152377	-0.455989983973
H	2.405200825209	-3.504393682376	0.122172381844
H	0.833601763030	-3.716486337447	-0.704924487329
H	1.154868263017	-2.235095804134	0.187820069266
C	-2.172856629608	-3.810374201664	-1.791327783676
H	-1.896454710583	-3.493846830472	-0.774636078693
H	-2.050560187915	-4.904220541357	-1.838100900092
H	-3.241407529460	-3.586249481892	-1.923132258219
C	-2.203374971624	-2.383830064174	-4.526006521727
H	-2.801652161752	-1.515932482018	-4.216372511151
H	-2.890564176857	-3.198004038573	-4.810395903018
H	-1.620553979868	-2.113233288124	-5.418286616729
C	0.069230980699	-4.263710945023	-3.798397748974
H	0.727829400426	-3.864890926735	-4.584318167529
H	-0.519012373545	-5.087474645758	-4.235279177156
H	0.713683870507	-4.687930320161	-3.012734732260
C	-2.292329347265	-2.428674461649	2.010530767878
C	-1.763355450280	-3.733200303195	1.936193546956
C	-2.604815648072	-4.791799685745	1.589211771515
H	-2.181134185084	-5.798609000252	1.521750702362
C	-3.959944289347	-4.601907999549	1.314860869027
C	-4.471308969260	-3.308875666336	1.431425747463
H	-5.535305979931	-3.135807783869	1.241774130827
C	-3.668162837575	-2.219813066484	1.780608476869
C	-0.328901796796	-3.997479105020	2.278189459179
H	0.035073924967	-4.914024623195	1.793091917678
H	-0.205067688417	-4.127928044535	3.366953655365
H	0.311592613223	-3.159260103271	1.984726374621
C	-4.825551057103	-5.743661977784	0.869119854228
H	-5.885328676393	-5.564270000934	1.101488511133
H	-4.525213970334	-6.689353678032	1.344124319717
H	-4.747421209063	-5.887717678731	-0.221971155132
C	-4.283562784961	-0.862360207736	1.954508031382
H	-5.227268160735	-0.777991287733	1.398072153172
H	-3.616976185877	-0.056331346391	1.619608893843
H	-4.510490667513	-0.665564781505	3.015653129852
C	-2.710673528612	-2.058865407673	4.974373523742
H	-3.736626384893	-1.931333670385	4.602968770389
H	-2.705628197385	-1.834146569047	6.053398240502
H	-2.443296565412	-3.119414931503	4.848231734604
C	0.185296144710	-1.280898045706	4.971462409909
H	0.561630217893	-2.291304545804	4.756291026463

H	0.041547961984	-1.193527281535	6.060844107546
H	0.951510544291	-0.554466983866	4.679679415044
C	-1.885579775576	0.862523227135	4.386452130968
H	-1.205665425806	1.517674013570	3.820936876113
H	-1.803133236597	1.124966156703	5.453294251731
H	-2.910473182499	1.080944481406	4.053087533970
C	3.866069164640	0.261051401627	1.255411536743
C	5.125507108739	0.603065643973	0.699058792056
C	6.075443130096	-0.399880406370	0.477784334159
H	7.033574856626	-0.113258481972	0.032036181636
C	5.859619119408	-1.730741519694	0.822515713902
C	4.664443586528	-2.024310882460	1.477248680714
H	4.487668790147	-3.042255568967	1.837549658620
C	3.685704756567	-1.059631650631	1.723956000888
C	5.563804129291	2.008110291373	0.364104854200
H	6.525257369586	2.216430414876	0.856174373162
H	4.853865675983	2.780435526219	0.673723491858
H	5.726339414771	2.138726065134	-0.716578365957
C	6.866406683947	-2.797279896816	0.509956707790
H	6.671442565726	-3.243529459719	-0.480492946926
H	6.833594290884	-3.613453628499	1.246263286928
H	7.889550832334	-2.394662280292	0.487588728076
C	2.541961886155	-1.429688108071	2.609549453026
H	2.491888432718	-2.516134920626	2.758120092189
H	1.567409426516	-1.089832907181	2.234564312806
H	2.678908060441	-0.971578422806	3.600549840825
C	2.941489429554	4.137249074718	2.404019304559
H	3.530598620039	4.410262507814	1.514894334289
H	3.274595873487	4.777870238226	3.236374251241
H	1.889557339786	4.371771394737	2.193581282417
C	4.919587247191	2.064634089478	3.527593400351
H	5.131248136205	0.994611346625	3.675386448725
H	4.971661589460	2.555171789956	4.513653512429
H	5.715150140253	2.488458593162	2.903247617447
C	2.115050850078	1.924992183358	4.382340454130
H	1.037108819156	1.857244113498	4.195064077536
H	2.279073598856	2.723654201778	5.125018963557
H	2.453038078519	0.984760516116	4.843314299891
O	-2.364365580940	-0.794843066452	-1.892634690104
C	-3.610033837386	-0.775438586397	-1.262448549728
H	-4.390697622538	-0.672621060284	-2.032428477180
H	-3.786029422407	-1.710254098929	-0.707551061822
H	-3.711405506989	0.072069572547	-0.570343847834
H	0.637382624391	2.268227958731	2.090691154052
O	0.870579028761	2.468173425016	-1.898048421718
H	2.107495770972	2.409690592947	-1.346958828539
C	0.768268453263	2.268001878855	-3.291879478898
H	1.042231108356	1.237234956357	-3.554384192249
H	-0.259601301556	2.461850888451	-3.629783685609
H	1.441904211474	2.965848205012	-3.811892020637
O	3.085370824026	2.148778119526	-0.989909325865
H	2.999836333226	1.854772357192	0.092110238776
C	3.447370620657	1.065818437786	-1.857384212334
H	2.740566506794	0.232013304188	-1.752606864267
H	3.430609600991	1.436693694887	-2.889219818717
H	4.452386334415	0.714834036413	-1.614585096150

158
MeOH_Int_6, G=-4752.585748

Si	-0.371826934197	1.894936091081	-0.643744411056
Si	-1.421518702362	-0.150815580213	-1.338110899659
Si	-1.136448970266	-0.623426804744	0.969778289155
Si	0.609011186109	0.702159046249	1.065125514584

Si	-0.746610716336	4.864325242343	0.059938578753
Si	-1.883204842566	-2.247244222581	-3.499093591794
Si	-1.052317807542	-1.979170445828	3.748424938047
Si	4.323226749722	0.995580466925	3.311323426627
N	-1.433046850368	3.258676662388	-0.312408557529
N	-0.773440535435	-1.146868958853	-2.616508318490
N	-1.743829805849	-1.804040545267	2.095104097612
N	4.242590762334	0.617679003176	1.598209183762
C	-2.829721345777	3.056839076258	-0.125424401742
C	-3.376222811002	2.870405254997	1.161400939842
C	-4.765262299856	2.760375823933	1.299654541896
H	-5.180798211978	2.621224230109	2.302474423332
C	-5.630147080417	2.830387095919	0.208658754427
C	-5.066173475317	3.007390514295	-1.058000675590
H	-5.720993007051	3.059887836715	-1.933271075204
C	-3.688411222274	3.110563703403	-1.245686248043
C	-2.507719269708	2.835129100283	2.384180596405
H	-3.012508317895	2.315029496084	3.210422274015
H	-2.267179719743	3.850916568396	2.737807489803
H	-1.556766822323	2.324928292787	2.182821607975
C	-7.113452701338	2.674415508512	0.377460698018
H	-7.668971653628	3.359744597838	-0.280156377508
H	-7.425015801443	2.868152876269	1.413863830893
H	-7.434937180363	1.650314728802	0.122060897604
C	-3.121935428812	3.266503862084	-2.623461908580
H	-3.919117084551	3.411418413359	-3.365176360361
H	-2.561527713280	2.361403322559	-2.910900867010
H	-2.425268146010	4.115038230624	-2.682412912499
C	0.009597253399	5.727198197415	-1.431102153133
H	0.989996965815	5.303390101912	-1.686935954190
H	0.156171907279	6.792514209525	-1.189436837463
H	-0.637511654247	5.672239598269	-2.319688657913
C	0.601246505652	4.682051724559	1.358132341133
H	0.221618790581	4.226260178979	2.284500995045
H	1.029189329062	5.665945522801	1.608853894482
H	1.426112451714	4.053667159417	0.985996898028
C	-2.155105525711	5.958088242379	0.650424427775
H	-2.925049665119	6.042884971085	-0.132569760506
H	-1.764744111187	6.968632509448	0.851513587257
H	-2.649835805463	5.594733912108	1.561240506778
C	0.622242397435	-1.217654778361	-2.902711692441
C	1.145048455713	-0.543621360073	-4.024522255535
C	2.514571575435	-0.628992792722	-4.298225673095
H	2.911821558350	-0.087933013255	-5.162844522963
C	3.384384178553	-1.377660979003	-3.505758047732
C	2.837084994158	-2.083638739901	-2.432341258169
H	3.495153903338	-2.693986130263	-1.807133144389
C	1.476831145503	-2.032081745571	-2.126916030811
C	0.240807053845	0.195695671659	-4.966777648803
H	0.791344672558	0.955955654816	-5.538884803081
H	-0.596286477350	0.674371315148	-4.441438252585
H	-0.203072523194	-0.498960715390	-5.699153970493
C	4.859189017614	-1.430980169188	-3.778858086185
H	5.182928285108	-2.456024172582	-4.021895821165
H	5.433412699395	-1.115480758959	-2.892980906217
H	5.138477790467	-0.781061911648	-4.620084291180
C	0.938681279748	-2.874031031501	-1.012065915468
H	1.587711412148	-3.741025062532	-0.825224042094
H	-0.074208902890	-3.233663551555	-1.236962619765
H	0.878922204351	-2.297954561208	-0.074268230321
C	-2.964071383129	-3.199370607259	-2.283712831135
H	-2.559458188596	-3.176359900939	-1.261009242279
H	-3.038962967923	-4.256681149049	-2.583950643560

H	-3.984209280293	-2.791877955560	-2.238219603835
C	-2.988415526713	-1.357874633792	-4.730660477921
H	-3.519967447758	-0.540178013177	-4.223101433720
H	-3.734669865127	-2.063832397866	-5.130846823902
H	-2.428442612562	-0.941292562127	-5.580003943368
C	-0.779450972856	-3.463054489598	-4.414302671049
H	-0.103169289541	-2.962320726329	-5.123103717692
H	-1.408771996962	-4.167901643059	-4.981598003476
H	-0.155390948049	-4.044941654583	-3.718869201664
C	-2.905039987675	-2.575684959855	1.770774753354
C	-2.781339067093	-3.892281022552	1.291344355210
C	-3.940578201720	-4.609221441952	0.981097075628
H	-3.837613470580	-5.628197245286	0.595887588469
C	-5.215168328127	-4.065869593371	1.139232418898
C	-5.312074802401	-2.768216748750	1.648335516248
H	-6.300595204428	-2.324024614036	1.799368982361
C	-4.182745316888	-2.014766835342	1.971442880209
C	-1.438998811658	-4.543322249363	1.146221267563
H	-1.444283509395	-5.291381471818	0.340966519425
H	-1.155653640516	-5.066055566055	2.075622144420
H	-0.653214675824	-3.807768604702	0.941494466047
C	-6.441146833797	-4.837822019737	0.746920474418
H	-7.315686447657	-4.537828531435	1.342317358956
H	-6.294860506341	-5.920340599418	0.873762342506
H	-6.691562257717	-4.664645121603	-0.313297343800
C	-4.327150211559	-0.636787246260	2.542139340154
H	-5.371204662375	-0.298393871939	2.500162381945
H	-3.714228379738	0.101459219789	2.003776985016
H	-4.000854372857	-0.604510946707	3.594471629720
C	-2.180729522556	-3.134231855595	4.698292035455
H	-3.205965892021	-2.740008607846	4.764095701874
H	-1.795423286946	-3.260826467088	5.722727420760
H	-2.236086371712	-4.127534540799	4.228604133749
C	0.671727688564	-2.694875462926	3.610209632031
H	0.647397955765	-3.723761376089	3.221992004834
H	1.171969232824	-2.705514946081	4.591196089780
H	1.289435992430	-2.090775045893	2.930123626658
C	-0.988029256567	-0.300931991297	4.585425511823
H	-0.417948531568	0.428778513878	3.991396027845
H	-0.499493909882	-0.387876504026	5.569080352452
H	-1.998768130461	0.106916210591	4.737849910282
C	5.143186433316	-0.227880868373	0.914811543845
C	6.271184089499	0.316501421476	0.259447709546
C	7.125086031691	-0.530122298438	-0.449179369998
H	7.993635058910	-0.096430290077	-0.955139579746
C	6.911039770355	-1.910192965003	-0.521417992333
C	5.799165676635	-2.428871998625	0.142395409331
H	5.602991228260	-3.505091744476	0.096731466903
C	4.909919140084	-1.614158558972	0.850769895096
C	6.534331732298	1.792475063141	0.318275826136
H	6.643804286547	2.133323820584	1.360104094504
H	5.701280432508	2.371665720711	-0.111129626943
H	7.454545567185	2.050833409047	-0.223903296356
C	7.824374150686	-2.794067510710	-1.321388120348
H	7.540413935039	-2.798342366831	-2.387879759479
H	7.788588621703	-3.835563231786	-0.970177019196
H	8.868592912170	-2.451639084115	-1.266498396319
C	3.693460427425	-2.198996393700	1.498225588425
H	3.474074427257	-3.202739534306	1.107848576343
H	2.824817124385	-1.545119405763	1.323788567122
H	3.808647066312	-2.287776876711	2.589996337405
C	3.864236939112	2.812349723945	3.459580614742
H	4.575322649282	3.445221192983	2.906136667478

H	3.859957160649	3.137951232357	4.511708237822
H	2.859815163580	2.999969372585	3.047146680904
C	6.057545213020	0.648427404074	3.949427496169
H	6.340330042194	-0.394314911315	3.732405804272
H	6.112706559248	0.793323782253	5.040015726249
H	6.809305261343	1.298990203966	3.477680073571
C	3.139225805194	-0.006770537329	4.382024460873
H	2.102859399013	0.054857817295	4.020453373736
H	3.160962067364	0.379681116516	5.414390466661
H	3.421982147296	-1.069519423951	4.419773810558
O	-3.024929677281	-0.088660235872	-1.815765175709
C	-4.157298505848	-0.212341830748	-0.997815089272
H	-5.052419139686	-0.084724567672	-1.625048662624
H	-4.202448333426	-1.205831535426	-0.522327887536
H	-4.185466256005	0.559073639879	-0.214072909769
H	1.191155390765	1.266386038174	2.316881568097
O	0.867772581287	2.619956274810	-1.549017349816
H	2.495223920851	2.626083759475	-0.813276646104
C	0.820496154822	2.865519109928	-2.932486859973
H	1.268352291942	2.024175099404	-3.479448124335
H	-0.211580349154	3.018619401451	-3.288347560656
H	1.396682189099	3.774819168632	-3.159288779473
O	3.404884646759	2.299254654272	-0.683412075647
H	3.928737028981	1.389779918101	0.998950902170
C	3.561525537186	1.307251041658	-1.669046497444
H	2.760182909380	0.551804118580	-1.625103852842
H	3.577280473982	1.733402061382	-2.689247108295
H	4.517402830767	0.793751643309	-1.507024904634

123
MeOH_Int_7, G=-3939.775504

Si	1.853843960969	-0.179118148091	1.138549744431
Si	0.397574877142	-0.650672056141	-0.641242118467
Si	-0.756196867471	1.188499969717	0.133860772720
Si	-0.012893244355	0.748529993878	2.165999361516
Si	3.763030295935	2.176669374441	1.836182207449
Si	0.476838457080	-3.526413420782	-1.730762403797
Si	-2.067208857931	3.904798837073	-0.083728839997
N	3.264309081888	0.818233656776	0.814992661450
N	-0.259669257519	-2.298583531230	-0.672340241916
N	-2.189606079863	2.136303838937	-0.292841797588
C	3.952826806035	0.534880153943	-0.410280379468
C	3.694548753100	1.302052244844	-1.564125039526
C	4.337187478619	0.975559111871	-2.759490386392
H	4.122219697529	1.574618539075	-3.650120222464
C	5.234583006578	-0.088863285109	-2.853198674749
C	5.504221864041	-0.811814439184	-1.691221993782
H	6.224152298589	-1.635251629476	-1.728091878308
C	4.889128215612	-0.516990919073	-0.471153915603
C	2.756438701449	2.468481546472	-1.515864266129
H	2.436330651046	2.761156100783	-2.525085317058
H	3.240829084150	3.348919519297	-1.062694362493
H	1.865248981946	2.242512548691	-0.910177193572
C	5.867404359119	-0.458564476172	-4.163412492130
H	6.853145282997	-0.924868370249	-4.019235938834
H	5.996233059079	0.419911048020	-4.812868567242
H	5.241732566360	-1.182151052211	-4.713156154066
C	5.238939353658	-1.317523322254	0.747568761460
H	4.420179393364	-2.000204082066	1.018990294062
H	5.405618459386	-0.663492022240	1.617110765685
H	6.147616358996	-1.911120105346	0.576843712912
C	5.468964124187	2.705710220651	1.265259072447
H	6.188232749768	1.875812606637	1.341359328873

H	5.836768550690	3.541058779768	1.881989454099
H	5.454843901143	3.034372514427	0.214948041093
C	3.830796427405	1.613265165578	3.631614443614
H	2.833329391843	1.320879926458	3.996106957509
H	4.184220289459	2.440702767169	4.268078278954
H	4.512714917385	0.760677636663	3.768584576042
C	2.565164266116	3.630018515008	1.771290701613
H	2.715432674249	4.255779827626	0.879805575026
H	2.684977740168	4.269028414817	2.660728623833
H	1.525219548079	3.267769682500	1.743333676572
C	-1.367048076418	-2.580088801771	0.162769526884
C	-1.218516592110	-3.214812351799	1.417856208936
C	-2.345252555142	-3.360755183005	2.245315029951
H	-2.219268019794	-3.854137392736	3.215211368358
C	-3.615015946387	-2.923837309191	1.864024962206
C	-3.750002084155	-2.369531172650	0.589350908193
H	-4.734967375104	-2.044264469250	0.244498989009
C	-2.660091698678	-2.200891292235	-0.263951646072
C	0.099919639359	-3.768821985146	1.868129008666
H	0.116563201687	-3.905961087434	2.959935806426
H	0.937251480239	-3.122118760485	1.577291810645
H	0.276299827804	-4.761085739924	1.424487635238
C	-4.795733806494	-3.037888171981	2.783248715979
H	-5.664401772910	-3.476739681727	2.268926622614
H	-5.108176277373	-2.045666382768	3.149926473332
H	-4.569742149865	-3.662424307615	3.659452991985
C	-2.859191497220	-1.661830430389	-1.644847177951
H	-3.887879139998	-1.310940664769	-1.792354256830
H	-2.637234655265	-2.437668842529	-2.395177129923
H	-2.181379458221	-0.821783767633	-1.854865819813
C	2.314829959319	-3.592179298804	-1.360864226226
H	2.769123193211	-2.608308138921	-1.564282531433
H	2.825657007559	-4.335504155177	-1.993463703933
H	2.502367226783	-3.841400596200	-0.305566167790
C	-0.395082080097	-5.159451042338	-1.389667552640
H	-0.244860564562	-5.539698457459	-0.370162234495
H	-0.022105416418	-5.921878173142	-2.092187391224
H	-1.479835728639	-5.056641666990	-1.552730778123
C	0.233739388667	-3.178925967733	-3.564534075308
H	-0.812377321482	-2.930566467095	-3.800316454104
H	0.506351139288	-4.074106973071	-4.147253367574
H	0.871638913766	-2.347590639293	-3.892474033016
C	-3.434952935034	1.581127045603	-0.700243846382
C	-4.332599891921	1.064170248601	0.254070564112
C	-5.575223584771	0.584624796041	-0.170160659237
H	-6.271107140919	0.197088260010	0.580921784358
C	-5.957970795017	0.595799131080	-1.512507920078
C	-5.048144062085	1.104614579314	-2.442786097682
H	-5.317843373997	1.117574592547	-3.503442587937
C	-3.795877072551	1.590887003118	-2.063419404698
C	-3.964280184729	1.036380258342	1.705356613379
H	-4.802087784440	0.675979479492	2.318772964329
H	-3.670994862982	2.034012037454	2.065421232444
H	-3.099026476412	0.377145317531	1.882904898374
C	-7.285551840185	0.045922402267	-1.948639832162
H	-7.711347143601	0.629409758327	-2.778501637588
H	-8.012731426482	0.045968358685	-1.123755311732
H	-7.189316642585	-0.995103311077	-2.301227072564
C	-2.839519367742	2.104559392900	-3.096772828922
H	-3.174545041090	1.842240073979	-4.109802511125
H	-1.831569429287	1.694661664663	-2.941517134573
H	-2.738131170039	3.200841467763	-3.052994315600
C	-3.721598769808	4.657153063799	-0.552253180146

H	-3.992469962211	4.473603489196	-1.601950028170
H	-3.687470436357	5.746583357571	-0.391486310715
H	-4.527207845714	4.243808878080	0.074161213935
C	-1.696960146649	4.347602424071	1.707968328704
H	-2.473563084998	3.970843258601	2.391082499711
H	-1.646732716860	5.442956302814	1.821416111527
H	-0.729857219028	3.933207465419	2.030100853780
C	-0.666183216948	4.576925774471	-1.140237406122
H	0.285708555824	4.107990656006	-0.844985398503
H	-0.563365475130	5.665774888237	-1.006213982573
H	-0.825276672105	4.373424735187	-2.209869574182
O	1.125150032067	-0.512540061291	-2.150927226713
C	0.689482061872	0.309232098377	-3.188331171903
H	0.366174724866	1.300354684880	-2.819515859340
H	1.521669167182	0.468714443396	-3.892285486772
H	-0.152459655492	-0.141479939979	-3.746121733196
H	-0.074520551857	1.633513754634	3.371450335884
O	2.313883699947	-1.612955743411	1.921842655851
C	2.703225031560	-1.726404433288	3.253133860610
H	3.790551005971	-1.576782001958	3.376195188652
H	2.191482511882	-0.993661091778	3.906260164898
H	2.458642709008	-2.738960271090	3.617703549957
O	-0.934527596899	-0.719794294876	3.194857263547
H	-1.219657271332	-1.461084052879	2.609591825167
C	-0.819179371268	-1.108771751396	4.555119602010
H	-1.784644119582	-1.488911289307	4.918419108347
H	-0.039388313260	-1.873731463951	4.685147276432
H	-0.546653725247	-0.211207701663	5.125221615019

123
MeOH_TS_3, G=-3939.759413

Si	-1.914912306238	-0.133295760616	1.252022452620
Si	-0.429343797305	0.724318640795	-0.389274454589
Si	1.134000618466	-0.881447539527	0.293248814530
Si	-0.287778944013	-1.709912105906	1.896626065213
Si	-3.908763537749	-2.524213607476	1.168039879253
Si	-0.730434425667	3.777306024750	-0.631392384507
Si	2.035237816001	-3.524301043352	-1.090374479244
N	-3.331395849384	-0.942619836473	0.597494178237
N	0.216889189141	2.348858230956	-0.162097488140
N	2.338455450056	-1.855244348719	-0.568125866545
C	-4.017344379669	-0.315724785143	-0.495065413570
C	-3.791527451155	-0.753808195820	-1.814679098173
C	-4.444449251959	-0.113210148573	-2.868239193918
H	-4.253759578359	-0.456876196620	-3.889840294566
C	-5.322106226170	0.951549786683	-2.658495998969
C	-5.559940020297	1.344757174248	-1.341486972444
H	-6.265101556476	2.158037947994	-1.144600024843
C	-4.933467216057	0.726643865322	-0.255353387793
C	-2.868379546359	-1.898376928324	-2.092125004621
H	-2.562937660636	-1.916329649505	-3.146715296858
H	-3.347054924704	-2.866835966519	-1.875582993065
H	-1.967835727726	-1.835651482315	-1.465523915851
C	-5.968182153035	1.663499031356	-3.811447611590
H	-6.928859883284	2.113749542001	-3.521683632276
H	-6.150061031979	0.983179761618	-4.656554124814
H	-5.323864887548	2.478809769758	-4.181908117776
C	-5.254545591096	1.171999995865	1.139702341303
H	-4.408959345144	1.713107038032	1.589244184831
H	-5.466548809283	0.310588089807	1.791791573454
H	-6.130061593102	1.835482729491	1.145597880708
C	-5.623165033041	-2.784476058530	0.456494986452
H	-6.310514871573	-1.985146481527	0.773128355449

H	-6.031129283220	-3.748359348502	0.800400411526
H	-5.607771987060	-2.789517976587	-0.643720386299
C	-4.008863934981	-2.531453770718	3.046865237601
H	-3.019966901243	-2.409150567393	3.514368204907
H	-4.413964564525	-3.499556877162	3.383960161201
H	-4.670655804772	-1.736285159491	3.421454223254
C	-2.768847368372	-3.940445691065	0.643743046371
H	-3.170470848384	-4.472340552568	-0.232208916141
H	-2.646106588215	-4.673539731687	1.456170371856
H	-1.766993639736	-3.575490754270	0.365855010581
C	1.498030992296	2.458084656031	0.451627161595
C	1.624180847564	2.615617427840	1.846991629333
C	2.895636692708	2.557497087565	2.422381432892
H	2.984382846288	2.664288234821	3.508722745891
C	4.049896926235	2.361576996070	1.659652826205
C	3.907570667886	2.284929216671	0.274749233847
H	4.793965896971	2.155145229193	-0.351369305494
C	2.655533053641	2.343876071454	-0.340600244087
C	0.418276406826	2.844680895365	2.705805274382
H	0.684497193955	2.814713889902	3.772054172838
H	-0.379513829605	2.114091119176	2.512905319767
H	-0.029632669801	3.831878539139	2.510448906784
C	5.394229690685	2.218342189549	2.311477980227
H	6.209207664323	2.411398645995	1.599197572869
H	5.535398182087	1.194205698968	2.696170225904
H	5.510347866295	2.906781600823	3.162399203857
C	2.541557112395	2.295803469844	-1.832497799851
H	3.526559645479	2.164573953766	-2.298211180581
H	2.088923006946	3.220718527419	-2.223519712284
H	1.895591352789	1.470706357331	-2.164212146879
C	-2.445008192615	3.620284116909	0.113512929151
H	-2.977647105810	2.776186927956	-0.355545521610
H	-3.041756102072	4.528424779207	-0.068194905995
H	-2.400123807394	3.430455625555	1.195939910695
C	0.198746954975	5.295219380979	-0.029924918166
H	0.315486239787	5.327677351718	1.062499079433
H	-0.336649589138	6.205248353225	-0.344433942503
H	1.207279027326	5.325898308751	-0.471537836727
C	-0.941122087247	3.936455224900	-2.494813128558
H	0.020803665512	3.931531805708	-3.029454652023
H	-1.451860799453	4.885296619406	-2.727672084783
H	-1.560321130773	3.112899482676	-2.877858385633
C	3.638078915045	-1.290187463614	-0.748992265077
C	4.559907329531	-1.274778955327	0.320006109543
C	5.824792494195	-0.723002505673	0.120020848740
H	6.532409228015	-0.715945981972	0.954841997412
C	6.214719036136	-0.185527239656	-1.109765859350
C	5.296337192233	-0.229857580325	-2.157818685929
H	5.579787780905	0.174301049060	-3.134843658950
C	4.017024195569	-0.773918497196	-2.002052774770
C	4.196743330633	-1.874951960513	1.643662992562
H	5.014847244019	-1.758744914839	2.367971060077
H	3.975099185126	-2.949643846945	1.545171236175
H	3.291235417751	-1.410176847990	2.063945937831
C	7.566026125937	0.446468485307	-1.278582322629
H	8.352970126920	-0.145313912087	-0.786886154226
H	7.592122347460	1.453569073473	-0.827970697406
H	7.833090161678	0.554538594409	-2.339528374092
C	3.079302110205	-0.829897674819	-3.170785543406
H	3.315976631120	-0.051753562111	-3.909754435055
H	2.036538568859	-0.712093422454	-2.849917227932
H	3.143399694309	-1.802174776713	-3.687755697653
C	3.645916285876	-4.204098477103	-1.774581558807

H	4.013980380840	-3.635142259653	-2.640254998264
H	3.503342716194	-5.251027542309	-2.086633953075
H	4.434168815518	-4.181261122350	-1.006051763223
C	1.506182118504	-4.619176912522	0.346836104470
H	2.199359815562	-4.535333634226	1.197854082653
H	1.493849984144	-5.672029607653	0.019997912933
H	0.497121820326	-4.372413430825	0.707163651785
C	0.666017408468	-3.567023995213	-2.376531060120
H	-0.236015171421	-3.069693046130	-1.988073557550
H	0.394799512366	-4.605089901451	-2.626672395557
H	0.960659401913	-3.054878202997	-3.304650433967
O	-1.255697097855	0.742754185428	-1.848960900740
C	-0.719729973690	0.460615970954	-3.105670714544
H	-0.220581102167	-0.525376687661	-3.126274771273
H	-1.540098334860	0.441313997968	-3.840589960469
H	0.008843602852	1.224441936100	-3.430568398672
H	-0.532599839231	-3.020653205008	2.570676211450
O	-2.291296828421	1.004647650000	2.444236366929
C	-2.837913494144	0.755027114562	3.703965061969
H	-3.937037461127	0.665510031488	3.661264453189
H	-2.443940942305	-0.172718596695	4.158312215966
H	-2.591604177391	1.595310946639	4.373550449649
O	0.839070459314	-0.924942042910	3.137364923141
H	1.334517564891	-0.389150455027	2.206802351836
C	0.576986083405	-0.324290313893	4.397020427829
H	1.529047681232	0.038147088812	4.808161227367
H	-0.123881255308	0.515505906980	4.294975937650
H	0.157342506749	-1.079346055663	5.077179818647

123
4, G=-3939.840329

Si	-1.880785908771	0.810708979576	-0.637742478469
Si	-0.066482783640	-0.464472131729	0.221180552803
Si	1.360850145607	1.252643459294	-0.605935412730
Si	-0.479952412441	2.697757982410	-0.408122162952
Si	-4.461703343536	2.357059138233	0.029468401591
Si	-0.886696180422	-3.365526235917	-0.497517323189
Si	3.375875856929	3.469712362972	-0.040142289796
N	-3.485289646027	0.868695512810	0.050664842537
N	0.312192710458	-2.036618566989	-0.436962795989
N	2.798635425351	1.811478038866	0.234058016023
C	-4.085632474961	-0.329480764111	0.551323705843
C	-4.028922888160	-0.614980129223	1.930860788453
C	-4.641889455666	-1.775417061982	2.407518455364
H	-4.588669509922	-1.994054375183	3.478397074302
C	-5.300990003450	-2.667939063817	1.559696170783
C	-5.358698006988	-2.353936433256	0.201562241157
H	-5.885954440218	-3.027128002591	-0.481525054583
C	-4.775714678406	-1.194814739762	-0.319384994680
C	-3.327043210533	0.314874340862	2.872949904376
H	-3.272776673575	-0.120134653205	3.880679924369
H	-3.854101425115	1.278420931481	2.953170516357
H	-2.304284073463	0.524476058557	2.527541148231
C	-5.892303859749	-3.941587570462	2.089606208447
H	-6.689065682766	-4.322063443185	1.434243694610
H	-6.313008147927	-3.803912452685	3.096720654847
H	-5.123949312352	-4.730119631295	2.164798680724
C	-4.939522690887	-0.859698968996	-1.772936173618
H	-4.000150832087	-0.512121588938	-2.222617529631
H	-5.676814837047	-0.049736700906	-1.905152738769
H	-5.299379002511	-1.730035628962	-2.338346294251
C	-6.208850907395	1.898660386489	0.531254145535
H	-6.657484211104	1.164165686178	-0.153583198116

H	-6.843209901660	2.799364461364	0.535297387177
H	-6.227101295535	1.462365382958	1.541701297692
C	-4.432401308717	3.076455615278	-1.707748044467
H	-3.405702555707	3.315424837666	-2.025373114951
H	-5.020226106422	4.007427235123	-1.752512438241
H	-4.853983504000	2.368198439055	-2.437029445846
C	-3.822542113198	3.669872785388	1.215905013934
H	-3.923369421250	3.351284223417	2.263962234767
H	-4.404171730629	4.597289478689	1.086556683735
H	-2.763395821991	3.909840585286	1.045938596023
C	1.569463475180	-2.286819506370	-1.074621721450
C	1.723799993085	-2.112738021573	-2.465568451777
C	2.938352935012	-2.466062945760	-3.064388409941
H	3.047012426258	-2.323954398585	-4.144187143009
C	4.001185397961	-2.998795845951	-2.337128924268
C	3.829290451266	-3.154968997091	-0.960648060394
H	4.646497462479	-3.566024102433	-0.360584411990
C	2.644443997763	-2.798975827969	-0.316374866123
C	0.613648782256	-1.592209671087	-3.327801285636
H	1.017680242890	-0.982386451286	-4.149184346620
H	-0.109693966964	-0.991705065362	-2.763231645235
H	0.051568995070	-2.421619762192	-3.787967529068
C	5.302772711290	-3.345655653720	-2.998680282590
H	5.791072204925	-4.199580101046	-2.506250411269
H	6.007429316628	-2.497416917096	-2.953215931306
H	5.163699318004	-3.595816734188	-4.060404185904
C	2.530743062941	-2.952138517558	1.167192970262
H	3.344012067089	-3.575885572784	1.562606164654
H	1.568305652648	-3.392244301346	1.462401170945
H	2.606494311149	-1.969962095947	1.659647214419
C	-2.142531386734	-3.097372071519	-1.867879233402
H	-2.359181069857	-2.027849442320	-1.998374129086
H	-3.085156043334	-3.614422296605	-1.629801074581
H	-1.769889823080	-3.481514240919	-2.829222535125
C	0.043818166473	-4.964289081298	-0.812149566574
H	0.587862788823	-4.927561288757	-1.768312394715
H	-0.673888119303	-5.799415847663	-0.858387990529
H	0.774102907518	-5.183174314645	-0.018952642913
C	-1.764187379349	-3.431495346747	1.154563606589
H	-1.073816562964	-3.721671514503	1.961310693156
H	-2.578162764931	-4.172260783618	1.117835778226
H	-2.218254876099	-2.464597191290	1.416689547299
C	3.561597937337	0.904325620352	1.021398743196
C	4.528747405175	0.083267669017	0.409414491559
C	5.286921499694	-0.778179776273	1.204114381364
H	6.038506918731	-1.410280391404	0.721814588849
C	5.106908175080	-0.861537784363	2.586658644218
C	4.142978796578	-0.037580851090	3.171610415043
H	3.985789394812	-0.081678343075	4.253917439811
C	3.368528836729	0.847278325030	2.414877991669
C	4.729701114621	0.137723744275	-1.073645316963
H	5.580867839193	-0.485107103805	-1.378936211006
H	4.907397238048	1.166709579535	-1.420823099473
H	3.840438083104	-0.231345598328	-1.608794269479
C	5.894921951570	-1.839754720069	3.408562971426
H	6.917233628518	-1.961816025879	3.021033809537
H	5.421618463345	-2.836605539528	3.395714368152
H	5.962920605741	-1.524337841310	4.459766317610
C	2.351164011869	1.730701410806	3.072879851539
H	2.170178349469	1.421898040713	4.111666895612
H	1.395596300971	1.715052221894	2.527878447774
H	2.686764514233	2.779444115457	3.091171887772
C	5.156739701502	3.548965071275	0.542407681208

```
 H          5.227480608352      3.307311207025      1.614375054098
 H          5.557163655784      4.564252933162      0.393650459681
 H          5.801415809122      2.841032026267      0.000944552381
 C          3.196448970272      3.860281258379     -1.869464936815
 H          3.710543736364      3.122552612935     -2.503979436111
 H          3.613070779173      4.856633802894     -2.088963096285
 H          2.130879690860      3.878087023285     -2.152853330276
 C          2.371530644451      4.766986898885      0.884300760599
 H          1.331144501261      4.789464790865      0.528162780596
 H          2.806267772679      5.761975594281      0.693150050781
 H          2.357343631251      4.605034159550      1.971532849929
 O         -0.323067113064     -0.400151978720      1.876176532804
 C          0.297015353255     -1.006362291750      2.971866540938
 H          1.386567932503     -0.845649586718      2.981642702669
 H         -0.125061653626     -0.569565460925      3.890574573281
 H          0.105828266358     -2.091493650661      2.991527445175
 H         -0.550954819186      3.199336814699      1.007784989871
 O         -1.839443373928      0.384926674140     -2.275029455902
 C         -1.853377514579      1.240936075993     -3.384824879931
 H         -2.877228971375      1.367441402327     -3.777105294105
 H         -1.444574780527      2.238954149566     -3.145807134718
 H         -1.232796168210      0.800664145582     -4.181600520115
 O         -0.517886461031      4.023762821342     -1.451343066628
 H          1.585578617539      0.981446848874     -2.066278282506
 C         -1.164828606596      5.236442157254     -1.185141382596
 H         -0.808785584693      5.991692548889     -1.902759722876
 H         -2.261443596385      5.154598127945     -1.290500875942
 H         -0.949437137475      5.606975876112     -0.165427671936

123
MeOH_Int_7', G=-3939.772177
 Si        -2.019595869238     -0.347613434276     -1.261805196437
 Si        -0.167413051451     -0.882473957730      0.141823388353
 Si         0.761051473074      1.111412843973     -0.612076409409
 Si        -0.654922359006      1.268070149369     -2.238418528656
 Si        -4.787762991653      0.991130383024     -1.317184116761
 Si         0.157713376123     -3.958037401144     -0.409448724081
 Si         1.843312673932      3.920282311993     -0.000319888671
 N         -3.480513282349      0.140724458782     -0.443855159094
 N          0.779741954841     -2.296324776665     -0.278481740503
 N          2.019413614082      2.135233830153     -0.003228009342
 C         -3.744896679123     -0.238475760685      0.908110842732
 C         -3.366603967245      0.628612066945      1.960953356683
 C         -3.696934206269      0.297868790237      3.273233484151
 H         -3.400129453948      0.980129174493      4.075912188878
 C         -4.380542319474     -0.879943934530      3.593324165298
 C         -4.712764786928     -1.736959138970      2.548461845629
 H         -5.229232168064     -2.676056581541      2.771224738768
 C         -4.410279758814     -1.443863781047      1.210486833253
 C         -2.568142081380      1.864259295611      1.682035792451
 H         -1.521523050177      1.586606225422      1.475575744100
 H         -2.573199709002      2.543665292848      2.545949239087
 H         -2.940244457963      2.398352435140      0.799487760875
 C         -4.723882824754     -1.205028051723      5.018152735043
 H         -5.410440536113     -0.455549812082      5.443991448603
 H         -3.824962319856     -1.214795267402      5.655158998366
 H         -5.207651629548     -2.188354807681      5.102233297660
 C         -4.805637834735     -2.436518888543      0.155024931777
 H         -4.337244198330     -2.219051986269     -0.811667145241
 H         -5.896777206473     -2.441992409443      0.008480051196
 H         -4.518795421093     -3.453429270287      0.459642542694
 C         -6.235283543471     -0.146327145313     -1.700350774803
 H         -5.921347146004     -1.021059250744     -2.288468507931
```

H	-6.978446896446	0.415773392076	-2.289740307239
H	-6.734236816177	-0.498977266139	-0.785759311318
C	-4.115815467290	1.662114799834	-2.933422575869
H	-3.406386700267	2.482944310899	-2.750997429969
H	-4.962019185203	2.068705942908	-3.511502722669
H	-3.637728056407	0.885972693783	-3.550511813713
C	-5.436248775765	2.404265311283	-0.264366191414
H	-5.625671329781	2.086955482635	0.772681679979
H	-6.385970014697	2.771655167287	-0.684963726091
H	-4.719207041569	3.237651155878	-0.259501685176
C	2.187287123579	-2.137767121148	-0.507125670912
C	2.684558271915	-1.962261402866	-1.815846395005
C	4.069153337893	-1.918411516425	-2.017146556820
H	4.442489937961	-1.785023272704	-3.037651719889
C	4.977062054521	-2.041225151077	-0.969543550795
C	4.461729181701	-2.156917385958	0.323078752542
H	5.150528575121	-2.206863130807	1.170464546607
C	3.092300222929	-2.186847992597	0.578965002937
C	1.784311363069	-1.809287503003	-3.006326405967
H	1.991430313701	-0.860916260801	-3.525710654171
H	0.730779481477	-1.796433104016	-2.712454308440
H	1.937237569459	-2.623816801199	-3.731415045357
C	6.456989499033	-2.049238776766	-1.217844870408
H	6.811634838837	-3.062956192850	-1.469587379204
H	7.015496810952	-1.718055652649	-0.330549969654
H	6.728975939144	-1.393057668829	-2.058289750829
C	2.607962996354	-2.242350920194	1.997102325517
H	3.451220832800	-2.143771554349	2.693578252454
H	2.089254697926	-3.185237515430	2.218576732102
H	1.896428915320	-1.431587428766	2.204807696193
C	0.603790230595	-4.726819551142	-2.064994927468
H	0.112528877229	-4.224211350095	-2.909930434739
H	0.284918694045	-5.781682230850	-2.066066471746
H	1.691153567962	-4.704749686961	-2.234399736401
C	0.902057501715	-5.083746811922	0.902285372690
H	2.002085214903	-5.080245527251	0.855649284209
H	0.563211947969	-6.117066838509	0.722154007398
H	0.595045123728	-4.805147865283	1.921642480859
C	-1.701213360255	-3.906104818757	-0.213299433327
H	-2.013490337607	-3.493176115465	0.757292083383
H	-2.094199373373	-4.933890924166	-0.275162449556
H	-2.176346633799	-3.313147352277	-1.008855244299
C	3.222242054208	1.582676849029	0.544184311597
C	4.335741173196	1.418186556507	-0.298830595814
C	5.547524179629	1.009234247957	0.258963993688
H	6.417246492693	0.901957537236	-0.395368701109
C	5.674031152888	0.721276461114	1.618317201010
C	4.538604809110	0.842842738498	2.421977210937
H	4.608729529393	0.605413216046	3.488089120468
C	3.309743103939	1.270539197867	1.912981963567
C	4.217249709935	1.679858962384	-1.769846611687
H	5.146925558123	1.414632150139	-2.291292774736
H	3.992937871912	2.736696976076	-1.986001033428
H	3.398960417841	1.087612108879	-2.209400413781
C	6.975722300490	0.240489431689	2.190818561845
H	7.146784430372	0.639862773840	3.201587898943
H	7.827024725240	0.532503710097	1.559193008659
H	6.990941968912	-0.860178448281	2.272668319421
C	2.118433723933	1.405819583776	2.813986728248
H	2.334095277185	0.986329720610	3.806345774064
H	1.233287984918	0.887363259214	2.412827975639
H	1.833998243322	2.460062599983	2.952362893150
C	3.512899380208	4.651988160795	0.428957905903

H	3.871430579092	4.273400634667	1.398238540958
H	3.424629215816	5.748087939453	0.498468903619
H	4.278620717270	4.416115934321	-0.324410443836
C	1.250332666603	4.508768337964	-1.681639883633
H	1.827383441414	4.047612295559	-2.497610643385
H	1.375169994286	5.601946821232	-1.751351227622
H	0.185506264112	4.284665944902	-1.847975897263
C	0.588569881526	4.434815161551	1.304261334496
H	-0.323800764049	3.820899827370	1.248818493726
H	0.298907242301	5.489236421936	1.173135963022
H	0.999185823067	4.323645790948	2.318735410336
O	-0.409027393095	-0.745890709619	1.798577328068
C	-0.937931758437	-1.711363313053	2.665321772547
H	-2.001721205437	-1.908126485494	2.454146797264
H	-0.377017590986	-2.661072557858	2.606108417413
H	-0.862987072637	-1.333582379525	3.695822720942
H	-0.871761480527	2.326911960393	-3.264250078692
O	-2.559506599942	-1.470050028891	-2.406395450483
C	-1.971320200181	-1.771514428130	-3.634293523653
H	-2.759837786238	-1.937396590499	-4.386117708496
H	-1.315062234561	-0.955340196816	-3.994711215010
H	-1.358757274295	-2.687803476706	-3.572673115157
O	-2.338204207430	3.962248389805	-1.162264666141
H	-1.707465854971	3.365643517190	-0.734569941503
C	-2.438875693621	5.162668305756	-0.449496565296
H	-2.691930532509	5.007910085233	0.615621240452
H	-1.513738072538	5.765959631678	-0.499972471771
H	-3.248349459748	5.752367610801	-0.903824791785

123
MeOH_TS_3', G=-3939.757511

Si	-1.987152886312	0.032899511562	-1.087067794911
Si	-0.214238069184	-0.663327663780	0.323936740196
Si	0.657878619984	1.478214249328	0.035513764409
Si	-0.581171791874	1.746654737163	-1.900344535288
Si	-4.596803533561	1.829430857662	-0.937673476557
Si	-0.243681045444	-3.619468047065	-0.795030739052
Si	2.522045262181	3.835048485962	0.579409469106
N	-3.530376605992	0.510636191323	-0.402285019306
N	0.556114858427	-2.081246790908	-0.365694264339
N	2.255488390625	2.079730206133	0.458863088300
C	-3.956024134501	-0.206014163949	0.767480639230
C	-3.629020423223	0.253818011262	2.057773138857
C	-4.113798082509	-0.448700960677	3.168990489763
H	-3.853609271613	-0.083818614179	4.167544592648
C	-4.885732042419	-1.599848445784	3.046277388387
C	-5.167771377619	-2.058059733063	1.754835217608
H	-5.754645673101	-2.972827946673	1.626849193767
C	-4.723645260454	-1.386334948818	0.617053624361
C	-2.744292226712	1.441756148436	2.295210965575
H	-1.886601166444	1.149109118511	2.917389725644
H	-3.284878437405	2.248204653654	2.815520377792
H	-2.339155097786	1.842856439676	1.360596999305
C	-5.393272236203	-2.338863387850	4.250438060822
H	-6.493847778311	-2.309177307053	4.302045372032
H	-5.003150625933	-1.906527701528	5.182583802964
H	-5.101988403260	-3.400729762928	4.221217321704
C	-5.056612681782	-1.926584577059	-0.743064796462
H	-4.159226209431	-2.016058083890	-1.370225097151
H	-5.755645221141	-1.266702140430	-1.276833091380
H	-5.524038348478	-2.917405000243	-0.661423276073
C	-6.397611483792	1.289612491928	-0.852915351076
H	-6.672779022083	0.604948005626	-1.668781774621

H	-7.040785655466	2.180784392264	-0.934289601898
H	-6.626741353726	0.797731636348	0.105102987463
C	-4.210280823982	2.290153189888	-2.718042317799
H	-3.173293148600	2.623071174712	-2.863765046615
H	-4.875181684980	3.117545677408	-3.015449312668
H	-4.400363156822	1.449297765835	-3.401086956739
C	-4.462613344656	3.348750307000	0.162521902833
H	-4.848129831734	3.135880388044	1.170853261444
H	-5.072328050218	4.162636866160	-0.263125510216
H	-3.426025034314	3.697921846546	0.255138780604
C	1.945899544730	-2.056327936950	-0.711808740396
C	2.361805765735	-1.704899096517	-2.010884176090
C	3.709175115682	-1.867185585649	-2.358026024342
H	4.021182361024	-1.603182825658	-3.373769667760
C	4.654235787865	-2.353632046239	-1.460093067271
C	4.231191996027	-2.623908871299	-0.155848870310
H	4.962188393174	-2.958113937229	0.585305053319
C	2.906987912108	-2.463268700744	0.241827758412
C	1.419098116267	-1.131526009751	-3.029470461890
H	1.675510107653	-0.077114208589	-3.225904241601
H	0.377983886838	-1.167642451535	-2.689325121525
H	1.487732999586	-1.667388543210	-3.988629503139
C	6.082413195571	-2.572377734063	-1.866599100681
H	6.253442988017	-3.616957230349	-2.177305714243
H	6.772540997255	-2.365324460806	-1.034896558070
H	6.365068818530	-1.929822597379	-2.713602266296
C	2.517800340165	-2.706024701132	1.669129920400
H	3.410174204939	-2.851509206212	2.292503140019
H	1.876780217430	-3.592750125992	1.774358072006
H	1.953778853228	-1.854170567191	2.074476840518
C	-0.299399381461	-3.891954873532	-2.652713634450
H	-0.895371145283	-3.112954206837	-3.145260401169
H	-0.765738039011	-4.870456061490	-2.853156045138
H	0.709940699065	-3.905296711093	-3.089970193868
C	0.716382079773	-5.080152459966	-0.091990393304
H	1.782945792572	-5.035798629379	-0.360718157801
H	0.300861600624	-6.008856063910	-0.516432198017
H	0.634898029386	-5.151293064192	1.003015535579
C	-1.981880048522	-3.663845201854	-0.083825245279
H	-1.995679514110	-4.106126065107	0.922332675486
H	-2.619275294799	-4.281977491968	-0.734986066272
H	-2.452912901476	-2.674400389286	-0.021595889593
C	3.356742184189	1.235903213026	0.791175091847
C	4.375723919698	0.999272587947	-0.147289030026
C	5.522723025066	0.314233186999	0.259295559561
H	6.320761452535	0.149243205936	-0.470527284565
C	5.673176214255	-0.171829631810	1.557298984412
C	4.623359058793	0.030160400721	2.456939143333
H	4.711507800434	-0.352758562338	3.478580030547
C	3.467268989838	0.725869038530	2.100390584648
C	4.251810131146	1.503311794287	-1.553200346509
H	4.877775929217	0.911558073369	-2.234957064555
H	4.571030205558	2.555528021233	-1.637798050875
H	3.209586106855	1.453966507267	-1.899706264556
C	6.901154231649	-0.933548460041	1.963924205753
H	7.203762172778	-0.694958323801	2.994649117184
H	7.750066197567	-0.714695148256	1.299975907523
H	6.726632330392	-2.022720437487	1.923878622169
C	2.372820823172	0.957424824469	3.099684208123
H	2.627744421718	0.508865602457	4.070011817473
H	1.414133571149	0.523582968406	2.768986984714
H	2.192397898281	2.032882583877	3.256145053824
C	4.186732392420	4.118105758816	1.391256667041

H	4.217897741367	3.672994601058	2.397390038372
H	4.379853017104	5.198874334484	1.483856532974
H	5.003296621169	3.668097035050	0.807090182082
C	2.500402060582	4.623069967993	-1.136462861978
H	3.482727050929	4.538601960173	-1.624630799864
H	2.238550363180	5.692202975642	-1.087667661423
H	1.772966181460	4.116050127542	-1.790137191363
C	1.181606262127	4.641518116995	1.628067065394
H	0.179954356708	4.559878766395	1.181163358590
H	1.408484880637	5.712323897833	1.759225429310
H	1.133847358236	4.176331733308	2.624664486362
O	-0.465176800840	-0.845890473245	1.976842258820
C	-0.967629580584	-1.961704332831	2.655022557186
H	-2.038629482403	-2.117954989891	2.445833937121
H	-0.415802851196	-2.881724931314	2.393918386635
H	-0.853582418542	-1.792252546537	3.737156565682
H	-0.553193644101	2.229432038859	-3.322699615100
O	-2.150028412555	-1.144893565949	-2.291813804393
C	-2.696544150196	-0.928000787161	-3.559486885734
H	-3.800619806051	-0.887249283538	-3.525147532831
H	-2.332013416609	0.011645389217	-4.014817876048
H	-2.409072744690	-1.761712732431	-4.220213303850
O	-1.348050111837	3.257245626167	-1.105902602209
H	-0.745460958513	2.901286128672	-0.164393658293
C	-1.282066237699	4.601717088543	-1.557942435586
H	-1.832899692788	5.235819596188	-0.848953313829
H	-0.241506954824	4.951306435174	-1.635613071287
H	-1.764543035274	4.671587749138	-2.542743849708

123
4', G=-3939.836711

Si	2.000129546634	0.312164457729	0.946291272723
Si	0.220320698833	-0.751991365410	-0.237965734144
Si	-0.746459742229	1.377622370389	-0.263070684396
Si	0.550915558963	2.131184923121	1.519909239701
Si	4.437888991586	2.153690802378	0.287658861319
Si	0.296065809921	-3.438170475102	1.401366108968
Si	-2.615289888721	3.723265202638	-0.632327871721
N	3.491906032006	0.653831937928	0.103678391997
N	-0.532471903103	-2.030027650368	0.676093634097
N	-2.359526355431	1.956000400213	-0.602833447033
C	3.995036761382	-0.341825456551	-0.795395764252
C	3.721464579256	-0.265217540170	-2.173903676793
C	4.253999004686	-1.240742493873	-3.024616857719
H	4.032306304103	-1.176674656836	-4.094423058509
C	5.039499594978	-2.291195887562	-2.554368954271
C	5.307418765228	-2.339130095677	-1.183214337373
H	5.934257345669	-3.144046782270	-0.787411156924
C	4.810155026671	-1.382586731246	-0.298286906179
C	2.899233698473	0.845282573371	-2.754376206271
H	2.337164799972	0.499103715312	-3.632417969767
H	3.541994869686	1.680234143700	-3.080778699998
H	2.183075528891	1.246162581122	-2.028126663298
C	5.563394593237	-3.351272157821	-3.479398696940
H	6.606642733720	-3.611456919210	-3.244501753098
H	5.520634437503	-3.027792754195	-4.529158747226
H	4.972873032224	-4.278968481428	-3.394841366119
C	5.165976496967	-1.449570855767	1.157657883788
H	4.270223536137	-1.536084407367	1.788876525997
H	5.691659359069	-0.537220738942	1.481190178947
H	5.818764839099	-2.309181572584	1.361266965318
C	6.121618463544	1.859897382643	-0.485686422411
H	6.655426487946	1.018639377296	-0.019354740992

H	6.740597581377	2.764662676041	-0.374458385732
H	6.030031575157	1.640007569446	-1.560295970421
C	4.631716611540	2.518399190713	2.123885210283
H	3.659510502930	2.586763372325	2.634952101799
H	5.153548580333	3.477357650337	2.273223601758
H	5.218059055856	1.730731128533	2.621475200247
C	3.625101912793	3.617352065131	-0.556670958657
H	3.616654093786	3.489138577624	-1.648622889077
H	4.185716412366	4.539165896834	-0.329992722618
H	2.586989543620	3.754974029907	-0.218524183358
C	-1.939446340320	-1.976735549564	0.949430765861
C	-2.414409085410	-1.402074753699	2.143881618936
C	-3.772814419650	-1.519579608617	2.460223467138
H	-4.130477804071	-1.078968394042	3.396358793322
C	-4.676504569073	-2.164379651612	1.620580326108
C	-4.196607464185	-2.650406705717	0.401828517780
H	-4.895519036890	-3.108404334880	-0.303401377086
C	-2.854957976051	-2.553301104429	0.040757123248
C	-1.521837559203	-0.617578283661	3.060757213711
H	-1.841313623913	0.436731132699	3.084225253535
H	-0.473922666412	-0.648641702064	2.739874685338
H	-1.569393718974	-0.990121446415	4.095436339130
C	-6.120203154808	-2.320690964504	1.999632045933
H	-6.283376209998	-3.248414824097	2.573685760330
H	-6.765495249481	-2.371034877607	1.110228883439
H	-6.466916571885	-1.486175328117	2.627540607663
C	-2.406489541551	-3.029848484748	-1.307599752025
H	-3.267414564437	-3.348294211341	-1.910470498193
H	-1.706564621570	-3.874229634218	-1.233007686364
H	-1.889313209815	-2.227607279497	-1.853685915220
C	0.327690654864	-3.331513434139	3.275250760928
H	0.886710280053	-2.445172349573	3.603735043134
H	0.825533529629	-4.228171088614	3.678868505126
H	-0.687952811260	-3.292967555354	3.696038198848
C	-0.625839614112	-5.018676383292	0.961906968047
H	-1.700886407834	-4.936351323590	1.184128205401
H	-0.219779033962	-5.847525402780	1.564230096696
H	-0.510035718506	-5.290409265731	-0.098068852674
C	2.047962930928	-3.557183205740	0.747362248298
H	2.088660169561	-3.889111478077	-0.298765750439
H	2.591762393185	-4.298689894294	1.354886020302
H	2.591126146017	-2.607741336114	0.834020339326
C	-3.381309105731	1.079552624493	-1.069411395964
C	-4.525765964166	0.850824169620	-0.282406176870
C	-5.549934834388	0.048889460171	-0.789874846362
H	-6.437748709945	-0.117517745211	-0.172683012287
C	-5.464071585479	-0.562304773501	-2.039733044396
C	-4.312444208056	-0.335907293518	-2.796120276632
H	-4.222100684958	-0.795589844197	-3.785373701252
C	-3.274485558564	0.479917613748	-2.342247678188
C	-4.658438966298	1.461042095936	1.079549734060
H	-5.397721854088	0.915518756213	1.680796137276
H	-4.986753758132	2.512286022679	1.026107980412
H	-3.696153280663	1.449893877241	1.609602949444
C	-6.550960305094	-1.469549024841	-2.538623815664
H	-6.704889143825	-1.361162019393	-3.622776614044
H	-7.508225380971	-1.267843435570	-2.036280166722
H	-6.300678185159	-2.528691791448	-2.353470766842
C	-2.076246576381	0.729756275431	-3.209901227896
H	-2.255481473068	0.372650699805	-4.233701642329
H	-1.175884623302	0.211800141976	-2.836271357691
H	-1.831851819434	1.802063424925	-3.253979352230
C	-4.214484560977	4.043578870910	-1.556013065330

H	-4.158966962081	3.627172439713	-2.573745605834
H	-4.387771224895	5.128438610892	-1.636506466711
H	-5.085918147446	3.590390993006	-1.061830429208
C	-2.640328425366	4.445620193732	1.103980653033
H	-3.420085128489	4.012484062017	1.745223172244
H	-2.802103307487	5.534703440080	1.051042845990
H	-1.661608658002	4.282761874805	1.582122947367
C	-1.177369672408	4.543291296376	-1.522562232713
H	-0.248501786777	4.446023348865	-0.938742095401
H	-1.386235459403	5.619350301830	-1.640043030060
H	-1.008348430662	4.115297272414	-2.522457172381
O	0.469684806912	-1.152635982849	-1.848482468504
C	0.998774689036	-2.341548235892	-2.365654501287
H	2.061515129410	-2.464256355434	-2.101984664296
H	0.438003145342	-3.224497497608	-2.013278335506
H	0.923132377352	-2.305716293610	-3.463436905119
H	0.095823902867	1.993130075299	2.943479318868
O	2.169055158155	-0.607076038247	2.359748911765
C	2.634632201106	-0.134292542501	3.590945248627
H	3.738025861559	-0.125972137414	3.632086634024
H	2.275610037888	0.889184836606	3.805100196714
H	2.267695508512	-0.797463260847	4.390731208216
O	0.926162112287	3.762859224928	1.308451875335
H	0.256372587904	1.895659146213	-1.275483366829
C	1.485333621223	4.598296755428	2.282009391663
H	1.003110746722	5.587725916272	2.231051391995
H	1.345935981479	4.202993627145	3.305336795212
H	2.567315456089	4.734898340959	2.111622592452

123
MeOH_Int_7'', G=-3939.774443

Si	-1.889047392789	0.511876420058	-1.002549814337
Si	-0.267190394581	-0.515699391463	0.451484581399
Si	0.858884469732	1.459783399768	-0.117472613315
Si	-0.590419759344	2.409403950740	-1.671970349660
Si	-4.261055168229	2.519392781096	-0.631790038960
Si	-0.639709548712	-3.439757308362	-0.572171527255
Si	2.964630381453	3.683326741661	-0.171396142394
N	-3.483863806123	0.940010646100	-0.368891023281
N	0.362471153745	-2.004724959320	-0.199349389676
N	2.494910805507	1.977150032826	0.153731002160
C	-4.231125757936	-0.060771195393	0.327503998014
C	-4.164534924116	-0.134919304299	1.733484752719
C	-4.870638658385	-1.133805267529	2.408352200240
H	-4.800853542499	-1.181062607376	3.499831920931
C	-5.671019092300	-2.056411923714	1.733675274893
C	-5.769685575589	-1.931695883823	0.347797761282
H	-6.414142154332	-2.622055005991	-0.205276087336
C	-5.071324587903	-0.956017445809	-0.367698540131
C	-3.371625355073	0.874372858773	2.503314164529
H	-3.263035459396	0.577884174598	3.555982222927
H	-3.865062127696	1.859797408551	2.482750265548
H	-2.373991741441	0.996327566062	2.066786848049
C	-6.388045969556	-3.152165706273	2.467293689129
H	-7.361736279030	-3.376341911605	2.006873851920
H	-6.560683753819	-2.888695837161	3.520935297873
H	-5.800578654659	-4.085874353242	2.454211631744
C	-5.221344146888	-0.893976074096	-1.857512251036
H	-4.370370659099	-1.384927507639	-2.352696996098
H	-5.239734838020	0.142092778801	-2.221321054521
H	-6.144207065575	-1.396172280729	-2.179155242355
C	-6.022647776949	2.372541565855	0.006039149230
H	-6.584550296220	1.589883659686	-0.525391555535

H	-6.547783848536	3.330402180144	-0.137927871864
H	-6.049698134466	2.124607596360	1.078176254280
C	-4.285518346028	2.971871087166	-2.455822504982
H	-3.261711988848	3.047426954357	-2.854308487628
H	-4.771882180034	3.951239573851	-2.594295728407
H	-4.838672487645	2.230973496816	-3.053339115320
C	-3.413256378180	3.925159891256	0.299642452774
H	-3.249147503478	3.675592867602	1.358824006047
H	-4.042731262344	4.829315984865	0.261317713141
H	-2.450016393986	4.167710539968	-0.177081743480
C	1.745575090599	-2.165607451958	-0.538772288322
C	2.188525230497	-1.934897568050	-1.856227072026
C	3.496723867788	-2.294677508959	-2.203655887078
H	3.824386614257	-2.130873090059	-3.235310572000
C	4.384670695397	-2.846688901677	-1.285626737619
C	3.950670189504	-2.978595261278	0.036138698659
H	4.645154868076	-3.354726890257	0.792565239857
C	2.661345940674	-2.632316342134	0.431810766734
C	1.317826034766	-1.277684101447	-2.886868314465
H	1.721652175130	-0.285666700635	-3.147862270921
H	0.293155658461	-1.132539203466	-2.529067476106
H	1.281398159452	-1.863678329659	-3.817707982487
C	5.766287129371	-3.272303921902	-1.688655320149
H	5.800869940184	-4.351370382039	-1.914881920955
H	6.494574239833	-3.087475517515	-0.884572162765
H	6.107531763323	-2.739576087939	-2.588534217415
C	2.270983301137	-2.734538989420	1.875475575007
H	3.145596176342	-2.974449828864	2.495114273973
H	1.509974315497	-3.509997751035	2.041912192447
H	1.847988072225	-1.785822590165	2.235542470089
C	-0.601220936678	-3.863736315411	-2.399572655960
H	-1.054703524369	-3.056990295064	-2.991614058870
H	-1.183382916977	-4.785142633735	-2.563722709330
H	0.422539614831	-4.042212387668	-2.760369376123
C	0.030856979714	-4.936328713885	0.354214242069
H	1.107370586316	-5.077241701720	0.171711772593
H	-0.491273031667	-5.839446250241	-0.001549033634
H	-0.132911119648	-4.865147328501	1.440375267444
C	-2.413423912693	-3.130124326969	-0.071227355040
H	-2.561652063953	-2.827175240935	0.974682289576
H	-2.979549807132	-4.063501640815	-0.224659587397
H	-2.857163513964	-2.362862480879	-0.718171268152
C	3.527076448355	1.079625019578	0.580432170249
C	4.487232426613	0.630022302064	-0.346341848845
C	5.564604042725	-0.132744956666	0.108932889098
H	6.312080586984	-0.463199176638	-0.617818632797
C	5.707805833388	-0.495846827357	1.446438576765
C	4.720597485980	-0.076882077667	2.339481261021
H	4.799967753903	-0.352376609871	3.395691525560
C	3.638455738968	0.705509826428	1.933968526393
C	4.394844731074	0.977846073496	-1.800881487757
H	4.931028137786	0.237324348234	-2.408639544248
H	4.841622140173	1.963376116630	-2.012014745297
H	3.351104970574	1.017761814115	-2.140187101366
C	6.854274054171	-1.349629244395	1.902960194141
H	7.194592373371	-1.067348555578	2.910409693579
H	7.711761892708	-1.273480201733	1.218768446160
H	6.563859574182	-2.413487846969	1.946292984471
C	2.618787592719	1.151089238916	2.934223513037
H	2.928220637852	0.891190240243	3.955963392629
H	1.644730228122	0.670112153255	2.746977906055
H	2.465187372773	2.239249067875	2.884756642932
C	4.724697352987	3.894500151002	0.439763394707

```
 H         4.804505400877      3.617357315637      1.502367781507
 H         5.022152034315      4.950211170022      0.335659317549
 H         5.445204395220      3.277125484976     -0.115298345718
 C         2.783294687403      4.156790365256     -1.976624257695
 H         3.406393925189      3.550996235408     -2.648125908603
 H         3.067895681389      5.214243713026     -2.103047195953
 H         1.732983139625      4.051745148814     -2.292576274581
 C         1.859806598540      4.854273345893      0.806062806466
 H         0.827702864156      4.847346039801      0.423850440505
 H         2.241932022759      5.881418815058      0.688580729877
 H         1.853986288003      4.623389437511      1.882034066589
 O        -0.429394505338     -0.564538872378      2.139289767332
 C        -0.981488614266     -1.598997109162      2.907848905602
 H        -2.081927968328     -1.618195194788      2.834345628787
 H        -0.596591345341     -2.586135191909      2.601257970672
 H        -0.706594900644     -1.440799921464      3.962582174011
 H         0.045359502390      1.858194526699     -2.935434608796
 O        -2.051116488425     -0.644099623089     -2.240629678844
 C        -2.205073018688     -0.305004411800     -3.590509473828
 H        -3.153386093939      0.229853404774     -3.779418830441
 H        -1.377893704613      0.333438246727     -3.948502060555
 H        -2.211451813949     -1.229612490404     -4.190078486756
 H        -0.681634873613      3.160460507785      0.690090157008
 O        -0.263611572617      2.452388625699      1.226155582093
 C        -0.380077609047      2.628908619816      2.638025441040
 H        -0.309617086224      1.634480545961      3.090248775891
 H         0.415104075409      3.289284570199      3.008494677437
 H        -1.363530565080      3.063362865223      2.857849145185

123
MeOH_TS_3'', G=-3939.773111
 Si       -1.940664432617      0.519703272218     -0.984764693490
 Si       -0.273274752120     -0.473381751099      0.446031836473
 Si        0.907424453022      1.495302888872     -0.027791627137
 Si       -0.629554485833      2.416759162580     -1.546292850247
 Si       -4.379615694558      2.463565455689     -0.703684287226
 Si       -0.651528712104     -3.398671077433     -0.614679201052
 Si        3.095804699270      3.644042927152     -0.112446837711
 N        -3.544074524418      0.923544134011     -0.374682696825
 N         0.340328834528     -1.962539326887     -0.217975218891
 N         2.576368890223      1.948758326088      0.176831049168
 C        -4.256955607597     -0.065439813503      0.373372435673
 C        -4.169670661211     -0.083526683923      1.778976067620
 C        -4.855990048130     -1.066484532070      2.498579954613
 H        -4.772305739590     -1.072749622013      3.590050857951
 C        -5.651262388823     -2.023815813599      1.869174164127
 C        -5.761679063429     -1.959960525635      0.478925336536
 H        -6.396683613811     -2.684646668286     -0.039996653398
 C        -5.083041207107     -1.005139600630     -0.280056428707
 C        -3.384160922460      0.964335097735      2.504420946166
 H        -3.141125714749      0.645881377560      3.527651313487
 H        -3.957797432758      1.902995212559      2.576685100968
 H        -2.453093051929      1.192889243256      1.975133100719
 C        -6.350472121087     -3.096880011127      2.652083092730
 H        -7.348736050092     -3.310278177572      2.241457609155
 H        -6.468725268966     -2.815363123248      3.708379966437
 H        -5.780683654965     -4.041038701283      2.624428580461
 C        -5.227169252230     -1.009690669891     -1.772065974631
 H        -4.320557070657     -1.413113043458     -2.247584004135
 H        -5.364127890097      0.004790081349     -2.171994620097
 H        -6.083049643438     -1.625130175437     -2.081284082369
 C        -6.179952946729      2.234362215228     -0.216185565313
 H        -6.673042297495      1.431564111636     -0.783549442877
```

H	-6.728778607510	3.172087974570	-0.399587910186
H	-6.273751599100	1.988691618080	0.852914373818
C	-4.257990288410	2.883381157916	-2.533511642579
H	-3.212594171575	3.031293549983	-2.847081535643
H	-4.801151658617	3.819555013730	-2.741162734676
H	-4.693700876229	2.090877423108	-3.161224963543
C	-3.707589742341	3.918446496938	0.282267651643
H	-3.762924813393	3.738257769517	1.366097000861
H	-4.306093310933	4.816789567081	0.058125029209
H	-2.662848421788	4.128728921741	0.010594916831
C	1.720808594994	-2.122796620223	-0.569317217365
C	2.157224888460	-1.858498775684	-1.882729726788
C	3.454624578904	-2.232314478382	-2.252094527856
H	3.777715685590	-2.044291237007	-3.280944206318
C	4.339278439538	-2.827021520118	-1.356824675151
C	3.915316487401	-2.986366822671	-0.035185928982
H	4.610392973518	-3.393125723062	0.704531440841
C	2.635278154108	-2.628630783436	0.381344580348
C	1.288721663837	-1.145465919182	-2.877273634836
H	1.641497751651	-0.108755157593	-3.014960584468
H	0.245737869381	-1.091250947387	-2.548802355406
H	1.323378710191	-1.629640152726	-3.864716856404
C	5.708455669726	-3.267239432471	-1.785817520573
H	5.705089766593	-4.322053523691	-2.108629850608
H	6.434131980567	-3.182220112599	-0.963180875662
H	6.079107158182	-2.669892610720	-2.632162823908
C	2.253908297185	-2.758138609960	1.825380550529
H	3.133737483080	-3.003554252892	2.435282875177
H	1.498026051594	-3.540134766043	1.984140823340
H	1.828934436245	-1.817653447416	2.204271515370
C	-0.618234830356	-3.777361177402	-2.452592823394
H	-1.094368569975	-2.966997748081	-3.021458593071
H	-1.179955074682	-4.707612934222	-2.636551856743
H	0.406925966271	-3.922446548116	-2.824399515400
C	0.028995902683	-4.914764891352	0.270699065006
H	1.104336360420	-5.051106389529	0.079039290755
H	-0.495316892363	-5.808011985457	-0.106275845408
H	-0.130338530508	-4.872012844615	1.358907081243
C	-2.423422621713	-3.116144359211	-0.093646705562
H	-2.563758369764	-2.851644551370	0.963532631418
H	-2.987098864164	-4.045555979449	-0.276607421864
H	-2.877340664732	-2.330226922820	-0.709194832314
C	3.581469995316	1.022301172433	0.598454815182
C	4.547412420434	0.571789139191	-0.321849911992
C	5.606315530915	-0.214010047618	0.136744805244
H	6.360371380142	-0.544470872046	-0.583317383779
C	5.721601409026	-0.602159645037	1.470331882498
C	4.726763709562	-0.183610951459	2.355404179342
H	4.786138373985	-0.478367197097	3.407775753812
C	3.663549489025	0.623760161129	1.947662763186
C	4.471638736933	0.940100789238	-1.772574145865
H	5.034202233791	0.221999154564	-2.383327656495
H	4.894452320657	1.939631616330	-1.965491280226
H	3.431533455224	0.958397368274	-2.124531171490
C	6.846654524955	-1.483094425189	1.928984214075
H	7.162643212148	-1.237688903980	2.953823822230
H	7.723061317108	-1.394368552053	1.270662766105
H	6.544797268141	-2.544811892488	1.929536317464
C	2.638988931059	1.077167821202	2.941500429562
H	2.922413564674	0.781904305881	3.961170548295
H	1.649223888250	0.638037163025	2.731831863866
H	2.520449232495	2.170655031346	2.918145189376
C	4.898829020617	3.783691347638	0.385849007664

H	5.033441785823	3.464562033614	1.431049688992
H	5.209115965643	4.838097591921	0.307402734984
H	5.575562014393	3.178718429003	-0.232755972857
C	2.806576030745	4.153717667675	-1.895742202905
H	3.327716085498	3.504959586778	-2.613323875595
H	3.157205169642	5.187641553048	-2.046447127584
H	1.731538247908	4.128276729494	-2.133882940988
C	2.127191636039	4.842334658918	0.966841697675
H	1.059237201322	4.862189250415	0.707766458180
H	2.528891883575	5.857715425001	0.816540836425
H	2.227745496293	4.599369095044	2.035722901914
O	-0.474559769121	-0.542072033752	2.121914041058
C	-1.022101887006	-1.583281255583	2.882762895764
H	-2.120761107578	-1.618579038288	2.792330952170
H	-0.616610660754	-2.564812258583	2.584001236198
H	-0.765995488012	-1.418078952161	3.940981749623
H	-0.254271946079	2.521412802631	-2.998874576463
O	-2.047106127379	-0.597535423573	-2.260473443139
C	-2.228253918235	-0.238698271861	-3.601279530685
H	-3.281696750535	0.012560338892	-3.819210474977
H	-1.604435620079	0.628261148334	-3.884546690366
H	-1.942464729798	-1.089632321788	-4.240604591886
H	-0.623219340534	3.158200547763	0.154912143313
O	-0.097870284891	2.574714816898	1.079512776579
C	-0.326459803918	2.741247059340	2.470673493328
H	-0.403420662823	1.756287898077	2.949063237900
H	0.485462799686	3.327366983055	2.924012783069
H	-1.274919735737	3.282383432966	2.593543938582

123
4'', G=-3939.840187

Si	-1.952161366863	0.308118270766	-1.062276409271
Si	-0.255000304386	-0.484357477976	0.443448081609
Si	0.764369561530	1.627479129091	0.092283975757
Si	-0.477458379571	1.999070732167	-1.863067011433
Si	-4.209526531350	2.438856521638	-0.913295511162
Si	-0.611912070853	-3.454292001701	-0.443204959169
Si	2.951995948797	3.761096342236	-0.047837499687
N	-3.522734705248	0.844644561483	-0.508610810704
N	0.381745829736	-1.988246663490	-0.165054628723
N	2.444228419705	2.065823382276	0.247012295903
C	-4.259963171379	-0.026627642401	0.355042343325
C	-4.150516863882	0.103438454401	1.752415088175
C	-4.854345387272	-0.778925045500	2.578643785937
H	-4.754468664628	-0.673174881039	3.663532514778
C	-5.681756452831	-1.775899656584	2.063027856044
C	-5.812607065879	-1.855690588684	0.674702549068
H	-6.475230990953	-2.611919508897	0.242702722109
C	-5.122996366648	-1.002106955798	-0.187234833809
C	-3.325500008759	1.198200476005	2.352261577073
H	-3.042417745211	0.965066992833	3.387903647605
H	-3.888084436514	2.146688464384	2.369436938510
H	-2.416065202721	1.378040628212	1.769556941851
C	-6.397637469260	-2.740919752057	2.962902067309
H	-7.418157346540	-2.944765852962	2.605280649080
H	-6.466315641305	-2.359487597706	3.991765656014
H	-5.870298350778	-3.708833842419	3.004123975294
C	-5.301320451328	-1.137059257790	-1.669144907843
H	-4.397505734720	-1.555834871393	-2.136023871649
H	-5.478261572083	-0.161390170411	-2.145014200173
H	-6.147057305262	-1.797549681895	-1.904029957086
C	-5.991469687180	2.435311663208	-0.324222819918
H	-6.585039899883	1.646611217156	-0.810454955501

H	-6.458071867339	3.407190604961	-0.551951954828
H	-6.052658442357	2.274279283998	0.762972159707
C	-4.173718908655	2.672686754847	-2.780219936745
H	-3.152055923909	2.626773868378	-3.186086646322
H	-4.591240656370	3.657552954183	-3.045273890969
H	-4.776657292780	1.901843232686	-3.284868228260
C	-3.276294962408	3.836488270529	-0.064857906504
H	-3.704335313764	4.032051995519	0.930729969247
H	-3.346966614699	4.767542432189	-0.649366601742
H	-2.211287388024	3.595630396847	0.075856057038
C	1.765711835691	-2.184168812863	-0.481456401617
C	2.216053875374	-2.055326522260	-1.808731415578
C	3.517859276465	-2.465136917753	-2.125609332378
H	3.851868438502	-2.383790832363	-3.165055973306
C	4.389368992111	-2.973348580870	-1.168420516430
C	3.949654337330	-3.001918939137	0.158825889579
H	4.632552756497	-3.342772631647	0.942113558978
C	2.668790245359	-2.598924935289	0.524634436187
C	1.359722887737	-1.460406733583	-2.888109169675
H	1.805643427191	-0.518130146558	-3.244505909045
H	0.344972480684	-1.246206645459	-2.535176763661
H	1.283884277160	-2.128056755569	-3.759687686372
C	5.765273411596	-3.450228140204	-1.530636746573
H	5.849842036299	-4.544058908012	-1.422067447465
H	6.529868060781	-3.003323602727	-0.876319098258
H	6.018933041307	-3.199366936883	-2.570525228542
C	2.268242990656	-2.594011349644	1.969217147516
H	3.138552660445	-2.786461118413	2.610872674994
H	1.506260164718	-3.355604668757	2.187396418339
H	1.841450145037	-1.623041358526	2.257932193323
C	-0.557724225640	-4.005662211580	-2.236133877943
H	-1.000610383461	-3.242575181870	-2.890398421039
H	-1.144591465789	-4.933117154953	-2.337821203684
H	0.468328378259	-4.217281708056	-2.571365555535
C	0.061213113771	-4.883248146694	0.582702480996
H	1.140046581563	-5.027873230457	0.418670743129
H	-0.452274878424	-5.809271972727	0.276531689968
H	-0.113505442817	-4.749129073484	1.661054021948
C	-2.399670769539	-3.150629482194	0.025107054895
H	-2.563472065244	-2.718314533388	1.021277236396
H	-2.918270688713	-4.123389639341	0.003125609607
H	-2.882216338744	-2.501689233603	-0.716039734486
C	3.477365155092	1.131034418407	0.565375660984
C	4.343798812697	0.669385110305	-0.442901906696
C	5.460067121477	-0.089671551082	-0.084207906474
H	6.140040266923	-0.423165820040	-0.873323779013
C	5.726944938248	-0.437394751401	1.238567463933
C	4.814094078289	-0.029742681540	2.213561396842
H	4.985637668419	-0.306544521308	3.258557270968
C	3.696051250983	0.745170409229	1.903299294373
C	4.105493492458	1.008059775318	-1.883511695611
H	4.542209269900	0.241152557020	-2.537404886834
H	4.558497036839	1.975267694602	-2.158216754571
H	3.031735374557	1.084946006425	-2.101163918917
C	6.926677590829	-1.262356333520	1.602979674501
H	7.380550199678	-0.920932953540	2.545502585080
H	7.697591208604	-1.220966199347	0.819974837161
H	6.655691393877	-2.322934478824	1.741780423659
C	2.754046888929	1.180712787651	2.982654690732
H	3.097215749719	0.836407158727	3.967954022437
H	1.741826257432	0.774926323757	2.820594027467
H	2.656457818389	2.276294734141	3.012645596265
C	4.795895394696	3.876042410761	0.271281624547

H	5.035026349898	3.522378616083	1.286142060139
H	5.109059612231	4.929509863716	0.192100998968
H	5.395434441193	3.283708440858	-0.433959841663
C	2.518644362278	4.297172514411	-1.797688987327
H	2.926979272453	3.619020821238	-2.560182917960
H	2.921818296483	5.306104903993	-1.983490561869
H	1.427848433556	4.348791916131	-1.938029180981
C	2.068251290146	4.938869756941	1.117833542942
H	0.977799872569	4.860046338723	0.999128789770
H	2.368075310033	5.974782431732	0.890794554229
H	2.321121661025	4.738241565793	2.170268266993
O	-0.509876017794	-0.502089757083	2.108019030842
C	-1.086173504442	-1.493429405924	2.909663336618
H	-2.185179266626	-1.503931123179	2.815827886572
H	-0.704414872173	-2.498058835214	2.659759441523
H	-0.831182923066	-1.286334207263	3.960769798874
H	0.182655939425	1.543566031904	-3.128866872313
O	-2.053240576170	-0.867471520418	-2.278568963349
C	-2.320970975598	-0.594800153558	-3.625227241205
H	-3.389184635660	-0.372512784753	-3.793869183431
H	-1.731857819834	0.263284810589	-3.997082982346
H	-2.055191303875	-1.478708142576	-4.226665329224
H	-0.968534624886	3.395753647059	-2.065065424293
O	-0.230496066679	2.616547141065	1.046327867913
C	-0.293147192554	2.738041055617	2.443713197748
H	-0.373070277558	1.755507659334	2.934290964845
H	0.587731884471	3.267975972953	2.839186119120
H	-1.185991189665	3.330648283632	2.696301785772

123

MeOH_Int_7''', G=-3939.775414

Si	1.778120814224	-0.208433902417	-1.271955043064
Si	0.050204955742	0.576790204003	0.158647495521
Si	-1.245745005387	-1.044855089890	-0.978150196763
Si	0.329668373170	-1.531749744704	-2.607869866834
Si	3.882211584017	-2.482120901306	-1.232477023889
Si	0.715024034037	3.603240048584	0.397884305178
Si	-2.886808257995	-3.631722423386	-0.893702750118
N	3.240469122626	-0.941364539673	-0.614662623822
N	-0.397505358863	2.264736657371	-0.000713529186
N	-2.414961457501	-2.061784502085	-0.177442339971
C	3.959836192255	-0.270621765505	0.421728765853
C	3.733760030399	-0.614532185626	1.770228296906
C	4.417887612258	0.070589041586	2.778022964543
H	4.225121637476	-0.201451065078	3.820764218329
C	5.345267660594	1.073982205882	2.493534818954
C	5.596874162976	1.358549926551	1.151157958021
H	6.341964650492	2.118834748844	0.896803251029
C	4.928345634126	0.706677876115	0.112109307829
C	2.794696775651	-1.725734446462	2.122106243445
H	2.604687331732	-1.754346547155	3.204309470936
H	3.209887771265	-2.703494963615	1.832441107327
H	1.835350027111	-1.615547244478	1.602702833618
C	6.035256139906	1.831273996580	3.590783716961
H	7.049964474495	2.136171958661	3.294901425957
H	6.112678946197	1.231945425875	4.509763084059
H	5.481062040064	2.750695664963	3.845603783888
C	5.242706229256	1.066108129721	-1.308110275478
H	4.444693861541	1.691036688272	-1.735876936957
H	5.313601510712	0.172424111069	-1.944447324010
H	6.190161715186	1.618759779524	-1.372423557233
C	5.620059217063	-2.675064046945	-0.545677669720
H	6.282421599076	-1.856340519460	-0.864599695696

H	6.053270231503	-3.625546813586	-0.896660351908
H	5.615731903209	-2.687619929561	0.555108200740
C	3.960702820644	-2.420395381566	-3.111391201648
H	2.968625085638	-2.274822526559	-3.566037245602
H	4.371108991104	-3.364957011942	-3.503853157796
H	4.612463337072	-1.600001628218	-3.450099129372
C	2.855572919637	-3.967054011948	-0.691574209006
H	3.134452328775	-4.299835538356	0.319712175432
H	3.008757238417	-4.813808816491	-1.379668242378
H	1.783521101127	-3.723745988023	-0.691324006907
C	-1.699292737766	2.645453053712	-0.441487103767
C	-1.913822222061	3.043385750473	-1.778331017359
C	-3.162701820639	3.563979403406	-2.139955779768
H	-3.310084952884	3.894266279786	-3.173674687881
C	-4.225800785848	3.644558346249	-1.242924192562
C	-4.019409695480	3.152101139328	0.050367788923
H	-4.845715500085	3.167297636971	0.768015433729
C	-2.781929308543	2.658153252677	0.468905368868
C	-0.867476376511	2.858804298034	-2.839547016664
H	-1.274721309223	2.261527356182	-3.671027639903
H	0.019658444519	2.342087281781	-2.453746659238
H	-0.548115190097	3.819897169343	-3.269537730255
C	-5.547006394980	4.235114783759	-1.642238266805
H	-5.641540445748	5.272738214887	-1.280718768084
H	-6.389101135562	3.668608263576	-1.216485572343
H	-5.666932122346	4.257611100218	-2.735036528697
C	-2.612582299827	2.143572758405	1.866663899335
H	-3.567419194769	2.161050020079	2.408953862468
H	-1.887616072855	2.749846871131	2.427965766568
H	-2.240768604313	1.108291065487	1.878844515379
C	0.934765904129	4.805418267443	-1.027386728051
H	1.432434740801	4.321736002802	-1.879792903359
H	1.573180936818	5.636059591669	-0.684375779367
H	-0.020053905878	5.234654298600	-1.365473032504
C	0.012099507720	4.595346388655	1.836661766230
H	-1.015293110149	4.928811339447	1.620763673203
H	0.627861721487	5.494408352253	2.000987621641
H	-0.000771842475	4.023886705991	2.777046995368
C	2.392871391906	2.894731965601	0.808853734643
H	2.412979121463	2.146044477570	1.613100921932
H	3.058628403501	3.718617317015	1.112869615475
H	2.820596075545	2.435711258299	-0.093444015006
C	-2.752680204316	-1.795793725199	1.190989520982
C	-3.957195464212	-1.142945603541	1.514930574409
C	-4.263339787697	-0.897698581759	2.856920703916
H	-5.204505825416	-0.392187771426	3.094927715252
C	-3.406934320258	-1.265850950037	3.892268007099
C	-2.226787401089	-1.928838302676	3.549901294801
H	-1.542109438228	-2.245906010460	4.342769075568
C	-1.888644895062	-2.213018353492	2.226054241565
C	-4.913829405848	-0.675852558093	0.460371153562
H	-5.951453930935	-0.732353588082	0.819713930956
H	-4.827592686556	-1.266169935259	-0.459560509681
H	-4.718156280406	0.377384075602	0.198928494167
C	-3.724706768141	-0.940476567104	5.322766393573
H	-4.800387121029	-0.765376248737	5.468150403599
H	-3.196364710368	-0.027655595288	5.646061483638
H	-3.415266065606	-1.750388693119	6.000150583755
C	-0.633277047952	-2.981365094189	1.945589729906
H	0.108864267951	-2.814342780818	2.736109460660
H	-0.174901169375	-2.707193866747	0.985699230933
H	-0.836915533221	-4.063626011619	1.905291696281
C	-4.345651017022	-4.286142928918	0.084883526939

H	-4.078189440656	-4.415526690035	1.144931278801
H	-4.647755300489	-5.268017834373	-0.313114591513
H	-5.215637062559	-3.614597276215	0.038470728810
C	-3.357965317157	-3.336033576772	-2.689482007407
H	-4.240401778418	-2.684630557829	-2.781515309302
H	-3.601053166700	-4.296642312356	-3.170894368291
H	-2.524543511038	-2.888348894109	-3.255512696083
C	-1.496610430557	-4.892588324843	-0.866280380238
H	-0.566408336123	-4.461585929716	-1.266261338352
H	-1.768841622012	-5.754107216671	-1.497397708622
H	-1.296105416659	-5.267621120282	0.147397387031
O	0.085095685010	0.053432980429	1.749675684716
C	0.483649671415	0.678588448545	2.929686076590
H	-0.008534205355	0.172967657217	3.776399069412
H	1.576345180254	0.620499358126	3.073392490078
H	0.190223612040	1.741696439071	2.953632238609
H	0.666095196136	-2.996492972461	-2.524199986231
O	2.150905962687	1.189312015140	-2.177268007871
C	2.466682162416	1.178801623419	-3.539775734743
H	3.527642470889	0.922658824085	-3.713583570242
H	1.842866079860	0.452330242534	-4.095222232036
H	2.285553124404	2.182439872402	-3.958495332525
O	-2.269366357459	-0.111927456531	-2.348654305910
C	-3.598720923153	0.355434550254	-2.556261550853
H	-3.604517126181	1.022730988184	-3.426435881483
H	-4.283004316747	-0.489136694460	-2.714437370815
H	-3.889173612527	0.930793912891	-1.672622375419
H	-1.737089999300	-0.360534862103	-3.149077705281

123
MeOH_TS_3''', G=-3939.775124

Si	1.821922933248	-0.137233000118	-1.268962114224
Si	0.046036065914	0.570488773483	0.150461300126
Si	-1.280841081864	-1.032404125105	-0.984665472253
Si	0.377125929974	-1.479265264090	-2.575463033102
Si	3.898623107269	-2.437949989680	-1.251860810392
Si	0.708085456333	3.600086395016	0.406201720077
Si	-2.810612530332	-3.677710098648	-0.839954078931
N	3.259308231036	-0.906497155438	-0.607937448570
N	-0.393346467089	2.257299302615	-0.005380644944
N	-2.398930826934	-2.079296478267	-0.155692570064
C	3.954577433451	-0.277092727366	0.471150764900
C	3.694637831304	-0.668597081887	1.799782635241
C	4.356608021870	-0.020560111213	2.846598594272
H	4.138932260109	-0.328150420155	3.874388574393
C	5.291925568077	0.989788519681	2.619318431894
C	5.576657158173	1.320890042433	1.293931834600
H	6.328942494417	2.087639354271	1.084386943407
C	4.932227571306	0.707116866097	0.217606097209
C	2.747974020546	-1.791198175854	2.091024433640
H	2.526485281785	-1.851289708079	3.165808633921
H	3.174836526298	-2.759667635294	1.786652264642
H	1.802717762247	-1.667465911022	1.549395114317
C	5.957446469111	1.706447087557	3.758153431626
H	6.987623716225	1.997879541292	3.505121687241
H	5.990954498710	1.083783335198	4.664057785953
H	5.412633857271	2.630600035788	4.015773695516
C	5.282284791741	1.108518263670	-1.183201512312
H	4.474629220033	1.706218085463	-1.631121259960
H	5.417311826382	0.230742261512	-1.831928529536
H	6.206781649612	1.701762927561	-1.201666283900
C	5.601868718814	-2.688956132644	-0.502784761710
H	6.290037831967	-1.871705359366	-0.766021756203

H	6.032543751671	-3.634220268515	-0.870492408926
H	5.553331966429	-2.739988421515	0.595689257409
C	4.059184551686	-2.314707795158	-3.122769051567
H	3.092873110893	-2.123490358386	-3.613464152867
H	4.457685139393	-3.257929812920	-3.530417669857
H	4.749760349025	-1.504608915367	-3.404442777671
C	2.810231109685	-3.909765516894	-0.800751852542
H	3.085308815552	-4.320245304840	0.182775775385
H	2.912197332762	-4.716505746928	-1.543892621440
H	1.749953349835	-3.620553162339	-0.763686455855
C	-1.692596973233	2.628698826359	-0.466291840641
C	-1.892889452308	3.013362081099	-1.808389150354
C	-3.145500364097	3.509109107683	-2.191504365911
H	-3.285588773629	3.823370893118	-3.230960087754
C	-4.221881367623	3.578089395519	-1.309940267947
C	-4.024287692834	3.105991343341	-0.007615906083
H	-4.860779275878	3.113033649255	0.698107128523
C	-2.785153932386	2.637108394256	0.431844685780
C	-0.830444950483	2.837455623578	-2.854392983632
H	-1.210217152573	2.206135071872	-3.673061483304
H	0.067136168900	2.356706808900	-2.448504399763
H	-0.536726636326	3.798655181908	-3.302426053402
C	-5.549884731607	4.134772800650	-1.734502863549
H	-5.684459943716	5.164421784524	-1.363112022538
H	-6.384352170654	3.537668307676	-1.336246974806
H	-5.643846960737	4.166085435898	-2.829505396643
C	-2.624007324411	2.141245159066	1.837471836004
H	-3.582170977853	2.165319430899	2.373211446838
H	-1.901789135484	2.753626658606	2.395304209341
H	-2.255073342188	1.105095767454	1.865139993427
C	0.949472297928	4.793759976641	-1.022229186712
H	1.461131032185	4.304864693936	-1.863254985022
H	1.579566105089	5.629195362421	-0.675567741796
H	-0.001635253513	5.216346017166	-1.378618045116
C	-0.019814792996	4.597244311397	1.828608826276
H	-1.045693067714	4.924070560398	1.596278870458
H	0.589390431185	5.500361044536	1.995176756759
H	-0.042884695652	4.031999881354	2.772575790074
C	2.381754478599	2.900656648235	0.855354205321
H	2.384702945672	2.131915021004	1.640738572334
H	3.025193217421	3.725024312861	1.203168488005
H	2.848277621441	2.471563327013	-0.042175885922
C	-2.803371135597	-1.764448712816	1.182072720487
C	-4.019102028156	-1.092114371296	1.413702448839
C	-4.398781053287	-0.799965373853	2.726104465868
H	-5.347879602538	-0.281317456827	2.893733756489
C	-3.602695153715	-1.137035417613	3.820250954671
C	-2.410606108896	-1.817966521005	3.568475323725
H	-1.774924620639	-2.112246890579	4.409523248572
C	-2.001972000204	-2.151404896270	2.275236674106
C	-4.897836985299	-0.657352737086	0.281290630695
H	-5.939462964287	-0.543679279642	0.613185563833
H	-4.868858859013	-1.366124704988	-0.556499013745
H	-4.571043964065	0.320563489381	-0.107632166426
C	-3.998836346432	-0.755406304562	5.216966115959
H	-5.091049101488	-0.679240845745	5.320684539396
H	-3.575368686075	0.225412699003	5.492481833484
H	-3.634973302711	-1.485370626299	5.954918746455
C	-0.744886887296	-2.943812773252	2.085348245902
H	-0.033866757338	-2.750615806353	2.898825456817
H	-0.247349288352	-2.713929310889	1.134490741070
H	-0.962168612902	-4.024904881144	2.084910937507
C	-4.187727798946	-4.394117712482	0.209690067893

H	-3.860721846634	-4.521659956152	1.253075280039
H	-4.481967887273	-5.381396646194	-0.180442078777
H	-5.077500161258	-3.746962806619	0.216806461653
C	-3.368733335593	-3.427931815224	-2.617018931972
H	-4.301738763409	-2.846468565900	-2.672332455994
H	-3.549790256091	-4.402496084909	-3.097471945035
H	-2.598449191766	-2.905323351927	-3.206639040416
C	-1.343289743549	-4.849139747641	-0.863025779791
H	-0.478943792030	-4.385424495931	-1.362066152733
H	-1.606633735199	-5.760508019510	-1.424045025564
H	-1.033562385115	-5.150682069331	0.147496082006
O	0.102728284938	0.037199694397	1.735502682385
C	0.460462044511	0.663351542291	2.928134597838
H	-0.072427822686	0.169475485920	3.756992167189
H	1.545868273018	0.589963877427	3.114070090480
H	0.181453490873	1.730403177817	2.935368091656
H	0.743123325627	-2.909660388786	-2.845797402170
O	2.209654780057	1.256740181387	-2.162305886058
C	2.540839600226	1.249064489069	-3.521315486642
H	3.597221215256	0.968628435370	-3.684673667908
H	1.905545572807	0.542729383677	-4.089744661489
H	2.386747338191	2.259125341740	-3.934170425399
O	-2.119667223110	-0.252792392159	-2.413836905430
C	-3.404111595151	0.248616357534	-2.737102131502
H	-3.323697390157	0.813473723728	-3.675681892159
H	-4.119722409450	-0.576460076655	-2.864787843261
H	-3.745286990418	0.931584970057	-1.950585595321
H	-1.202432800806	-0.547326742522	-3.121278965558

123
4''', G=-3939.846666

Si	-1.898885673831	0.931703820346	-0.774178932962
Si	-0.137272880607	-0.350251436291	0.183057110687
Si	1.319439351051	1.340853791030	-0.656599047274
Si	-0.514430409713	2.841372361327	-0.669609053551
Si	-4.445451086776	2.615131279096	-0.159788768775
Si	-1.051265056476	-3.266731777218	-0.380665264576
Si	3.387916856321	3.512194603220	0.046843793628
N	-3.491885726784	1.110997080703	-0.074725222449
N	0.186483488431	-1.973199950277	-0.369286535814
N	2.695418891545	1.885207725752	0.267882417055
C	-4.095890663209	-0.022422880789	0.558483803283
C	-4.008002895147	-0.171969726434	1.957962697371
C	-4.619618992301	-1.273028746186	2.560780945263
H	-4.540746389197	-1.386614963701	3.646281747336
C	-5.315551394661	-2.232503616026	1.822644268289
C	-5.412300910753	-2.047657883614	0.443314284351
H	-5.972490592967	-2.772012568782	-0.156086169649
C	-4.827452322184	-0.954365302416	-0.202536971135
C	-3.295637947982	0.847870360993	2.792756798597
H	-3.181931656696	0.493711374338	3.826769122644
H	-3.856439076526	1.795699506288	2.827490531616
H	-2.298710529753	1.062787679087	2.384643073342
C	-5.905062687926	-3.440822834643	2.489139516632
H	-6.732629450420	-3.864537056072	1.901995550518
H	-6.283446568475	-3.204249802161	3.494630113376
H	-5.145720110882	-4.232902038791	2.606688841575
C	-5.038448014902	-0.749548241064	-1.673432892477
H	-4.103240691477	-0.495942851057	-2.188244600330
H	-5.740687861188	0.081894591441	-1.853542381728
H	-5.461494208265	-1.649475058655	-2.139940177230
C	-6.139801977028	2.233801090652	0.549933984806
H	-6.638369521242	1.419923295628	0.002744440900

H	-6.775725551194	3.131227670735	0.487318976792
H	-6.078745802939	1.930220252453	1.605875525878
C	-4.610522394440	3.182115875988	-1.945215679831
H	-3.629080598584	3.428868931826	-2.378878937445
H	-5.228648617795	4.093476121040	-1.991131826697
H	-5.083889610418	2.415744655828	-2.577090704164
C	-3.677861702324	4.040371485323	0.798389810645
H	-3.369434713169	3.746618038643	1.811934901818
H	-4.420519460447	4.850514724001	0.886137704344
H	-2.796904176201	4.454530956085	0.286521732329
C	1.461418149398	-2.300052642287	-0.937102314235
C	1.661554725246	-2.247400652051	-2.331150359453
C	2.898582246480	-2.634333505218	-2.853355822762
H	3.047524130984	-2.580136404484	-3.936277063873
C	3.938358503181	-3.093063864083	-2.044451777901
C	3.711892630978	-3.152969543482	-0.669415059895
H	4.504443100006	-3.517277391712	-0.009151376727
C	2.499494648498	-2.757763359047	-0.101251880243
C	0.565796477992	-1.829612294061	-3.262427138625
H	0.984993017102	-1.462805985769	-4.209938329643
H	-0.075180760421	-1.052074877802	-2.827922102586
H	-0.087265438898	-2.683102487605	-3.507431443128
C	5.268619602555	-3.469092202944	-2.628932753842
H	5.792091713425	-4.205478058004	-2.001672940571
H	5.925822281235	-2.586507303132	-2.714884270522
H	5.162915753045	-3.892655596813	-3.638587579268
C	2.314832071953	-2.820318831833	1.381812280189
H	3.120250804824	-3.398179813015	1.855090465749
H	1.348707288105	-3.267789107853	1.655076538608
H	2.341276544286	-1.809945243182	1.817825907984
C	-2.314563696077	-3.016226307616	-1.750484802898
H	-2.425527087227	-1.948815787992	-1.988063983963
H	-3.296399227779	-3.408008847826	-1.443213775174
H	-2.010060857917	-3.533105886215	-2.673041475370
C	-0.158738007251	-4.896426542321	-0.640868180953
H	0.377938022442	-4.909403304005	-1.601769780323
H	-0.893884716055	-5.717493786533	-0.647054132057
H	0.575202587111	-5.097225164684	0.153821050511
C	-1.933236916766	-3.263552573767	1.271483460624
H	-1.255147054931	-3.556298780712	2.087537083092
H	-2.768238770349	-3.981198689353	1.248323926530
H	-2.358692002397	-2.276763502042	1.507745654774
C	3.376514991230	0.975363997721	1.131578137671
C	4.365464408909	0.115403986340	0.614654570988
C	5.054935667613	-0.722674373367	1.493091460213
H	5.825630934835	-1.384366828589	1.086956726497
C	4.787753161420	-0.744529678554	2.863585620127
C	3.804353177868	0.118050435675	3.351779459163
H	3.580261428990	0.124651278814	4.423070026373
C	3.095224790598	0.981315045153	2.510901105713
C	4.684166356813	0.116785507046	-0.849157658950
H	5.494012225324	-0.589907958598	-1.072290771510
H	4.995671085201	1.117959322871	-1.186859780589
H	3.809576577321	-0.160258959120	-1.457086294278
C	5.505153809715	-1.698492197914	3.773542188092
H	6.541615577345	-1.867182873104	3.445920523688
H	5.004342192652	-2.681839606704	3.786565464230
H	5.528838170735	-1.330912914774	4.809665611774
C	2.064132774852	1.915916142033	3.069323334037
H	1.828349506093	1.666679419854	4.112993548276
H	1.135467009653	1.885175222042	2.480807193169
H	2.419067894683	2.958421476531	3.048820303787
C	5.055086500926	3.532146518702	0.904402361454

H	4.951006149707	3.332826313200	1.981948603148
H	5.525894570414	4.520497329368	0.781876587464
H	5.735431748000	2.771582451580	0.493320717336
C	3.571651058915	3.866832738300	-1.790287948592
H	4.131705762018	3.072469767607	-2.305590203241
H	4.103348963572	4.820396256657	-1.939728975411
H	2.587934893314	3.960437448131	-2.275749224977
C	2.307329642323	4.878241568971	0.757973304174
H	1.335363231680	4.939896851488	0.246101401635
H	2.816495685555	5.846297980514	0.619683106148
H	2.112110818771	4.747431177325	1.832082779572
O	-0.457746663535	-0.185855926953	1.819780314854
C	0.028224666855	-0.777033904840	2.987684972212
H	1.118032241818	-0.662198487186	3.095905868747
H	-0.457240354865	-0.288192705477	3.847092949065
H	-0.213496281551	-1.850909180212	3.026251397855
H	-0.613600074093	3.410046072744	0.711584305945
O	-1.887907392542	0.379847410624	-2.375203021233
C	-2.186193941277	1.100324879737	-3.533969300621
H	-3.271603557638	1.116123592172	-3.734917234859
H	-1.835219097672	2.147181605470	-3.475696032253
H	-1.687048286816	0.619677360800	-4.390403118238
O	1.704824612774	0.927580948448	-2.236464709062
C	1.439051489285	1.602023121790	-3.425174423845
H	0.823799716434	2.508059284104	-3.268380579380
H	2.378389252262	1.912263430148	-3.913423023060
H	0.893908271253	0.941383818400	-4.118488823601
H	-0.638399355487	3.963457883972	-1.657416286890

153
PhOH_Int_1(THF), G=-4712.939660

Si	-0.494140769267	1.367059623300	-0.512949268518
Si	1.223557824885	0.370647521139	0.534135658904
Si	-0.321381261996	-1.119382727423	-0.083066558267
Si	-2.294872993496	0.109697245516	0.347172059130
Si	-2.072935800645	3.041519737913	-2.500975564674
Si	3.781128853237	2.046992088424	1.171655754322
Si	-1.655549723339	-3.726588975861	-0.962236399212
Si	-4.083177598846	0.423239241524	2.844309244108
N	-0.538023768678	2.691662175857	-1.630020782001
N	2.928177282728	0.506951103277	0.777422588774
N	-0.184166954508	-2.734998348833	-0.679212203822
N	-2.461046497339	0.434269283505	2.120524908197
C	0.670313090153	3.342689407420	-2.020712462733
C	1.547345188195	2.732583718567	-2.940315489020
C	2.728252129187	3.393834878636	-3.288229169717
H	3.407681947332	2.912993351833	-3.998648862946
C	3.060047859629	4.647015973096	-2.764509055381
C	2.162011575545	5.240173029926	-1.874828583065
H	2.395831701094	6.222455005696	-1.453285085388
C	0.972902487820	4.612244924762	-1.491586460351
C	1.202806389428	1.416569530577	-3.568772835592
H	2.038744786932	1.034571008278	-4.169850975063
H	0.324815567401	1.509518828246	-4.227604062339
H	0.948358074992	0.657453936726	-2.813526282888
C	4.358342469434	5.312084920676	-3.118249781887
H	4.304220407629	6.401799818687	-2.983785656505
H	4.648031102134	5.105191194855	-4.158953508399
H	5.174553081179	4.941645511339	-2.474893737405
C	0.037922301891	5.279529614221	-0.527899408589
H	0.512399215956	6.151864126430	-0.058477422632
H	-0.285571597576	4.586922050878	0.261941346253
H	-0.878907180324	5.628007806875	-1.030098928356

C	-1.696014564966	4.383815440207	-3.752920414255
H	-1.372835935833	5.322667425510	-3.279751313154
H	-2.603489006053	4.591725829486	-4.342129876549
H	-0.904575545143	4.061708510138	-4.447175036026
C	-3.385088625512	3.587199952367	-1.279821035951
H	-3.599292992359	2.788025314184	-0.554371142754
H	-4.322079371359	3.812555979192	-1.813880936348
H	-3.073253645047	4.486588648309	-0.727874004965
C	-2.627396457969	1.486421113395	-3.395357923227
H	-1.963304332928	1.258643853943	-4.242992730768
H	-3.653713409676	1.604689862932	-3.775997019492
H	-2.627843284211	0.621434364725	-2.713928004491
C	3.776377710409	-0.648387001902	0.674691853538
C	4.113376395388	-1.400788482368	1.816600574341
C	5.072236430493	-2.411459048940	1.694380418314
H	5.343047246491	-2.983852189302	2.587434271162
C	5.698222834195	-2.701170252362	0.482302920872
C	5.289774694314	-1.988923835793	-0.647859308779
H	5.730681871313	-2.225687921220	-1.620294655440
C	4.319873245044	-0.988013154184	-0.582675192597
C	3.461251761344	-1.171391733998	3.147760488464
H	2.953708769230	-2.089324859322	3.483808245973
H	2.713772156857	-0.371760857107	3.107207868602
H	4.200575490766	-0.911384516566	3.920712660098
C	6.735204798414	-3.782537040757	0.393402730331
H	7.342749678643	-3.686716628888	-0.517649316796
H	6.265234167543	-4.779763883160	0.371342194310
H	7.410633068682	-3.764297318158	1.261737318681
C	3.822531211422	-0.336683998763	-1.837066289988
H	4.484347508310	-0.554289279281	-2.686568813646
H	3.713016581078	0.750142118605	-1.740483301784
H	2.826220302223	-0.737051597692	-2.086477764642
C	2.479223031609	3.354706799273	1.443525805579
H	1.958083384752	3.591807352191	0.506155228396
H	2.966537294451	4.276737694633	1.799738718214
H	1.738692375209	3.041914038239	2.194363500615
C	4.812372579899	1.814434158747	2.719422005542
H	4.187521047436	1.628527721773	3.604004183349
H	5.387380316851	2.738352253063	2.894162867459
H	5.527961058743	0.985296245347	2.613272260174
C	4.923788492574	2.538304865998	-0.230202792627
H	5.642206241598	1.739304265011	-0.469147518596
H	5.495785102929	3.430115625465	0.073890205093
H	4.355817336134	2.791788785600	-1.137728159843
C	1.084126572068	-3.265137367572	-1.067944827767
C	1.874107958426	-3.931989626884	-0.111554747975
C	3.045217926162	-4.566932716173	-0.527220600926
H	3.644400244920	-5.101476971067	0.215297416729
C	3.468413238692	-4.539409603032	-1.858629914924
C	2.700717916268	-3.816782900124	-2.773197129244
H	3.025970006377	-3.757795745147	-3.816738453213
C	1.514917949281	-3.174721729486	-2.404513006458
C	1.442567807015	-3.974853111555	1.322782078218
H	2.191543842683	-4.483778071209	1.944461052520
H	0.478745738255	-4.494531097824	1.444134628783
H	1.301499473909	-2.958482679716	1.723682506759
C	4.726175960055	-5.233408850255	-2.293432938462
H	5.571204187568	-4.527647348059	-2.349100646137
H	4.613241805141	-5.680413811235	-3.292298360911
H	5.011849041222	-6.029226889316	-1.590894498266
C	0.710610803404	-2.438802090016	-3.434989653350
H	1.356544152639	-2.063354676988	-4.241219028265
H	0.166255936023	-1.590283403829	-2.996715786908

H	-0.042608150961	-3.095530139297	-3.900620579057
C	-1.067456348034	-5.430003076134	-1.472368205240
H	-0.486632619206	-5.392373686269	-2.406379792941
H	-1.938370983859	-6.085172781947	-1.633730202019
H	-0.433009450465	-5.883840997145	-0.695752908940
C	-2.646607997378	-3.831743062768	0.627396814048
H	-2.064414803876	-4.316477661068	1.426190083256
H	-3.560938841074	-4.424268554079	0.461097009109
H	-2.948512018018	-2.835177070967	0.983236312297
C	-2.699361723292	-2.958760988870	-2.320248323492
H	-2.907814643292	-1.900176831855	-2.099222237048
H	-3.664939245397	-3.481498232483	-2.405978338756
H	-2.191079035833	-3.012825745104	-3.293650213902
C	-1.362653403343	0.767011816498	2.948654427706
C	-0.992640524960	2.116810586591	3.129182735128
C	0.074951767993	2.429598916460	3.974521479090
H	0.345255924114	3.481340614807	4.112057470706
C	0.804531559651	1.444003693836	4.644986233259
C	0.430956951860	0.113260356191	4.444751041790
H	0.984253296463	-0.680360435417	4.955613729608
C	-0.636061680930	-0.242285216402	3.613971712188
C	-1.747008246879	3.203926571472	2.427567963429
H	-2.761120225291	3.326973810726	2.840561524660
H	-1.880292462808	2.969009589184	1.359542352420
H	-1.229198101254	4.168806783813	2.516579172366
C	1.934809972851	1.805784520157	5.565130067556
H	2.465244405874	2.704621368639	5.217198675166
H	2.663855443279	0.986409903622	5.648007638125
H	1.568985890542	2.020184677052	6.583519251065
C	-1.019744478083	-1.678816590662	3.445690670826
H	-0.274148253041	-2.348328734077	3.896117021326
H	-1.125793599314	-1.936784668889	2.379852682309
H	-1.996423698989	-1.892384909825	3.908466481654
C	-5.230008684017	1.601698219810	1.930488979007
H	-4.867688203983	2.639462771312	1.990100741351
H	-6.241022395863	1.563983967370	2.368095920228
H	-5.311564342237	1.333192887204	0.866111698122
C	-3.913162621662	0.946657602063	4.640995454679
H	-3.261874545766	0.252234793310	5.194252180898
H	-4.904983931873	0.943714726972	5.120997369926
H	-3.486709039621	1.956489854422	4.737535843141
C	-4.855964515436	-1.291169526998	2.791403631457
H	-5.014610282657	-1.628042706241	1.756826357836
H	-5.840280515914	-1.272415172254	3.287700151662
H	-4.227694653404	-2.035516500576	3.303706273682
H	-4.288284347326	0.186325808155	-0.962752620200
O	-5.136770285754	0.414965838217	-1.420487381741
C	-6.026064552812	-0.588474761771	-1.334985860365
C	-7.326818529884	-0.359634578450	-1.804968714815
C	-5.703668760006	-1.843688880969	-0.800888603512
C	-8.282053264981	-1.370182350525	-1.739212976110
H	-7.568372885825	0.622048322770	-2.218682095636
C	-6.668953467886	-2.846668883353	-0.736906650140
H	-4.688477026884	-2.024494378200	-0.445154856032
C	-7.963770119823	-2.620551399879	-1.203826645353
H	-9.291834374865	-1.175952215110	-2.110313627126
H	-6.398548094313	-3.818538335087	-0.315160007396
H	-8.717273729617	-3.409390172407	-1.152796030151

153
PhOH_TS_1(THF), G=-4712.918739

Si	-0.499802538829	1.391838071390	-0.583184407001
Si	1.141213417887	0.390014341216	0.584393196516

Si	-0.230048450610	-1.158706400049	-0.251141108839
Si	-2.179340880270	0.015223626842	0.265514563449
Si	-2.065395672517	3.226231761389	-2.463542470830
Si	3.603213525333	2.108029927792	1.433742208350
Si	-1.480750744093	-3.747828254567	-1.283038263647
Si	-4.245789867972	0.205430624896	2.534932526167
N	-0.545759780162	2.789764657135	-1.583377302072
N	2.814438573992	0.560984080104	0.923440794045
N	-0.035840412030	-2.740332698977	-0.881102321129
N	-2.548216897846	0.226242772240	1.978314257507
C	0.673813305246	3.471462649103	-1.890699071576
C	1.580129863119	2.921801367876	-2.819774431684
C	2.768793517985	3.605658124656	-3.087200835659
H	3.471463736488	3.172873236795	-3.805575408907
C	3.079117582627	4.823301130161	-2.474296496959
C	2.151295038600	5.357495091988	-1.578131135508
H	2.367383374631	6.312217166324	-1.089400898511
C	0.952172254396	4.705945002250	-1.273386520945
C	1.259998246231	1.651234664998	-3.548093466563
H	2.114033632023	1.317226242431	-4.152078570150
H	0.398869565534	1.787185018076	-4.221751067325
H	0.992608324431	0.835326106340	-2.859270770239
C	4.384996221485	5.512151060949	-2.743402947953
H	4.316167149463	6.594152201867	-2.561922320972
H	4.719014459488	5.354276942045	-3.779233631417
H	5.176285170655	5.118086419338	-2.083245609764
C	-0.020268492507	5.323221459974	-0.312811246190
H	0.458708145556	6.117026657028	0.275970729091
H	-0.439313335440	4.577201722328	0.376466641663
H	-0.874654580520	5.774304894586	-0.843197653409
C	-1.604382035193	4.610913484194	-3.637687825182
H	-1.218681154948	5.500248575639	-3.118672255409
H	-2.500114184897	4.906756230818	-4.206812848288
H	-0.839828223141	4.277353764283	-4.356343691687
C	-3.362815959258	3.754568610204	-1.220429557061
H	-3.597199660298	2.927193421265	-0.533097009838
H	-4.294902874479	4.019411181105	-1.745260521169
H	-3.040975882117	4.621540953793	-0.625120026666
C	-2.684384337333	1.742974322538	-3.423694521728
H	-1.860818091795	1.204204922751	-3.916687659217
H	-3.374115362679	2.093942351776	-4.209797087971
H	-3.250499834780	1.042671738707	-2.787130468945
C	3.703892008908	-0.562998201823	0.794911339480
C	4.028985731958	-1.355977979874	1.912027943485
C	5.029440196502	-2.323110684921	1.775750496644
H	5.292862585930	-2.926934079736	2.649963929000
C	5.704649593345	-2.531828268777	0.573372148668
C	5.304392055401	-1.785183933410	-0.537308032243
H	5.783379406297	-1.962114516536	-1.504287737111
C	4.297802408288	-0.821906810993	-0.458547266622
C	3.321201390858	-1.219508698208	3.227308326874
H	2.826813620396	-2.168142291866	3.489683208676
H	2.553048173578	-0.439128976925	3.204597313964
H	4.023753640745	-0.987232050829	4.041968988039
C	6.790113679136	-3.563295386498	0.474954436654
H	7.392122955927	-3.432200411170	-0.435056077815
H	6.366861914169	-4.580790315623	0.447476326994
H	7.464413312558	-3.519328565570	1.343382204288
C	3.820491049666	-0.126034041093	-1.697111854992
H	4.516357593213	-0.281953534371	-2.532600496036
H	3.674005591749	0.951567053790	-1.554589332331
H	2.846909865453	-0.544002494847	-2.002180462629
C	2.239685552110	3.348925215453	1.711239033139

H	1.731264751922	3.593432683772	0.769262184800
H	2.677510830481	4.280242713136	2.105082705423
H	1.498050782497	2.983477645928	2.436497862955
C	4.569918001146	1.825851964762	3.012219796575
H	3.912316258379	1.582520100096	3.858407061573
H	5.109111837776	2.756141479304	3.254299100683
H	5.313993608494	1.023174860956	2.900256820400
C	4.780146564559	2.694876642729	0.101529244832
H	5.530547693768	1.928091337354	-0.144353548738
H	5.313447161405	3.587295976278	0.467467878682
H	4.239488022615	2.974125223399	-0.814963796152
C	1.265781261669	-3.231342501553	-1.217620131427
C	2.013087059936	-3.920444253197	-0.244370633251
C	3.221414065051	-4.511586700749	-0.617455453297
H	3.789676964138	-5.064199804393	0.135976708581
C	3.719862714265	-4.416263575577	-1.918842293696
C	2.988300012380	-3.672795051690	-2.846778922069
H	3.371713777743	-3.561850001939	-3.865870854237
C	1.767440686524	-3.075173184863	-2.522971394634
C	1.504251157406	-4.030884302871	1.160725430831
H	2.224795936139	-4.558890314971	1.799569702671
H	0.542398183919	-4.565583709801	1.206705405928
H	1.330310713295	-3.035223499344	1.599374127351
C	5.017359525942	-5.060890808465	-2.310016605386
H	5.831065234857	-4.318769015490	-2.357242981998
H	4.949278808984	-5.526609326391	-3.304565076554
H	5.318707707396	-5.833392550213	-1.588725571629
C	1.004338533289	-2.318474781509	-3.569567224687
H	1.681301616955	-1.930004454786	-4.343073498462
H	0.442914376847	-1.475781139327	-3.140940751350
H	0.268117636358	-2.965010371198	-4.074880250360
C	-0.816018465537	-5.395456476704	-1.871415703817
H	-0.212834011618	-5.287342617973	-2.785382668623
H	-1.659686933254	-6.068267572102	-2.093055755046
H	-0.187472915035	-5.871140370993	-1.103208133268
C	-2.511462480393	-3.974074574885	0.267131254857
H	-1.931671774746	-4.460896691150	1.065983293526
H	-3.383856868284	-4.608749896536	0.042300722229
H	-2.890654660731	-3.015298581143	0.651752672468
C	-2.495107452500	-2.913378840692	-2.617465923547
H	-2.944444378312	-1.965837105825	-2.276738158710
H	-3.323199164046	-3.578641779327	-2.912107059042
H	-1.889030091117	-2.713725220044	-3.512765418521
C	-1.511298681339	0.504282442892	2.905401916613
C	-1.156653001091	1.838356654511	3.192041538623
C	-0.151288106018	2.091007435762	4.130299226232
H	0.108866532513	3.130048750757	4.354356906105
C	0.528756791386	1.061449845205	4.784873931643
C	0.173932639014	-0.253458452270	4.472301896983
H	0.695009171775	-1.080050500421	4.964065995224
C	-0.830685041440	-0.551016533215	3.547944133211
C	-1.850661239273	2.972142164221	2.502029207884
H	-2.890477920396	3.085727949413	2.847552533069
H	-1.910227841322	2.802079976710	1.415056275203
H	-1.331619881914	3.923585677062	2.680564404767
C	1.591436969684	1.355791623209	5.803746948684
H	2.061836629548	2.333362455664	5.624136449953
H	2.378418879741	0.587195134559	5.797273394202
H	1.170107593196	1.378350007109	6.822797030791
C	-1.187968503548	-1.972252149788	3.245380154264
H	-0.500724644190	-2.671549308347	3.740334844269
H	-1.159298824933	-2.167289806038	2.161429106654
H	-2.213847131243	-2.209037552909	3.567334669472

C	-5.244836258108	1.406422578238	1.496543014714
H	-4.901664959077	2.442903130332	1.635736747488
H	-6.311854303995	1.354836304030	1.766022169784
H	-5.155898333311	1.146780909332	0.429222393512
C	-4.244055340283	0.686779633575	4.348563559902
H	-3.651294937410	-0.025893811452	4.942921144470
H	-5.278032861730	0.671711304417	4.728932299682
H	-3.831443829603	1.692805251198	4.515419464173
C	-4.982082852573	-1.509449338698	2.352264843146
H	-5.008279024149	-1.821600214197	1.297253259845
H	-6.022504414313	-1.508722462232	2.716840663119
H	-4.418095380221	-2.259051509734	2.927918832346
H	-3.459954424298	-0.232546741604	-0.681892077448
O	-4.600853303668	-0.214509693626	-1.633753920334
C	-5.714621523600	-0.845097393145	-1.407149088641
C	-6.966319280280	-0.168382155308	-1.446235633754
C	-5.765627936635	-2.233361032898	-1.100869061610
C	-8.164064665360	-0.828216264208	-1.193992806379
H	-6.958376218565	0.899471525870	-1.684100721976
C	-6.970668085296	-2.882863012679	-0.846835805522
H	-4.826725767090	-2.788651969221	-1.067517912041
C	-8.185263730982	-2.193335433927	-0.886603299459
H	-9.103079590261	-0.266724614774	-1.234808505088
H	-6.960066093455	-3.951863450868	-0.610505918410
H	-9.128372826044	-2.707343974538	-0.686735755115

153

PhOH_Int_2(THF), G=-4712.921049

Si	-0.439499753061	1.417727401119	-0.587530432976
Si	1.181895966510	0.384700711930	0.579842279579
Si	-0.191474698935	-1.161837567489	-0.258423736034
Si	-2.080631690224	0.059565609149	0.323555865829
Si	-2.059265492411	3.242529104029	-2.415533913879
Si	3.650162643854	2.097108910775	1.397713557579
Si	-1.519523196212	-3.721063552903	-1.271740448254
Si	-4.223897272555	0.220017425715	2.515984433340
N	-0.510558968346	2.817687927454	-1.572507768685
N	2.853573729416	0.546234226732	0.902065879396
N	-0.043062510699	-2.745374132308	-0.879851004590
N	-2.506678099363	0.258465351109	2.007801253890
C	0.705388319159	3.494076064831	-1.908479555858
C	1.590984458500	2.931328870681	-2.849581304921
C	2.781677321776	3.602662813207	-3.138272487624
H	3.469004585536	3.160401537472	-3.865550385280
C	3.113055566635	4.819336208456	-2.534461315686
C	2.203566112850	5.367117975557	-1.627728479564
H	2.435877168303	6.322183013676	-1.147314953459
C	1.002636326116	4.729111134630	-1.302074069823
C	1.248529785119	1.658911112421	-3.564958346752
H	2.079749704379	1.329577512333	-4.202316972341
H	0.359544579052	1.788055862013	-4.202781387006
H	1.015374565714	0.839850143064	-2.866983778276
C	4.422079440551	5.493283014562	-2.824659576607
H	4.366834724696	6.576852236770	-2.648178576584
H	4.740753029040	5.326208611431	-3.863827363842
H	5.217622811810	5.094368874303	-2.172606038321
C	0.049135204845	5.362210745437	-0.332960770111
H	0.544579812718	6.152712581726	0.246464737112
H	-0.371787929843	4.625288009514	0.365125896338
H	-0.804678308313	5.822007904152	-0.856809534607
C	-1.613782010103	4.580028550517	-3.647074753166
H	-1.195750242386	5.475918960700	-3.165360920600
H	-2.522335315280	4.876699929251	-4.194909583549

H	-0.879137848048	4.212420750363	-4.380068809866
C	-3.305233808158	3.832918609415	-1.149046416147
H	-3.521985681473	3.036909928543	-0.420280108629
H	-4.253458058388	4.080758084361	-1.652908531948
H	-2.959986698818	4.721954824636	-0.601697452466
C	-2.725601019611	1.727208251107	-3.291660805424
H	-1.916426894944	1.115719315699	-3.720544349583
H	-3.366302455630	2.060247308283	-4.125730736191
H	-3.351503054106	1.092029311800	-2.640795936754
C	3.730342052771	-0.589099770492	0.778116786524
C	4.048854805752	-1.377274635064	1.900405240980
C	5.032313938971	-2.361778691053	1.765857427291
H	5.291118986857	-2.962948542049	2.643194404549
C	5.695921077539	-2.591743856604	0.560779917005
C	5.301890343625	-1.847278986096	-0.553537905415
H	5.771968642728	-2.040153410295	-1.521789609851
C	4.312480732668	-0.866069807335	-0.476768505221
C	3.350825976273	-1.219774110439	3.218740453858
H	2.844162470894	-2.158954009849	3.491464685560
H	2.594028256298	-0.428263645511	3.196131466840
H	4.061983280375	-0.991865244713	4.027109735753
C	6.763041924033	-3.642226552260	0.464273715855
H	7.360069276286	-3.530294559029	-0.451484558882
H	6.322270978153	-4.652480925819	0.450349620467
H	7.444497231287	-3.601072425588	1.327190202796
C	3.840571111733	-0.172811980598	-1.719308435646
H	4.541173038777	-0.329449140046	-2.550629763830
H	3.692844416477	0.904940076549	-1.579891701480
H	2.869654549715	-0.593115819639	-2.030494313448
C	2.284350046494	3.334148700887	1.684650407936
H	1.772188644961	3.583574735743	0.745713008347
H	2.722567646592	4.264495592634	2.080359969486
H	1.545746975888	2.966802861320	2.412533998005
C	4.631751220754	1.815393779887	2.965884712735
H	3.981541411010	1.580959675985	3.820341641516
H	5.180696904203	2.742517102949	3.197691149146
H	5.368340482127	1.006500797183	2.849133337489
C	4.807092960007	2.676267847458	0.046002705607
H	5.555875006868	1.909327187089	-0.204239663491
H	5.343377580058	3.572405722435	0.398175190914
H	4.253572861633	2.947982337699	-0.865182984572
C	1.248218918463	-3.264583242018	-1.218038144639
C	1.987895512658	-3.957029208197	-0.242002132362
C	3.187928168307	-4.565563927508	-0.614817124313
H	3.751379221082	-5.120208524577	0.140694508313
C	3.683807701757	-4.484815809533	-1.917985735437
C	2.958110505969	-3.739923670716	-2.849801999926
H	3.339425743111	-3.641620079369	-3.870876526044
C	1.746196874474	-3.124658179978	-2.526597100786
C	1.482618069053	-4.051242009325	1.165624662630
H	2.194844018711	-4.591336711657	1.803589002400
H	0.510229441403	-4.565757423429	1.217667081197
H	1.331469198497	-3.050873864373	1.602442075167
C	4.972665475296	-5.146940093239	-2.308293197277
H	5.792562713279	-4.412858340705	-2.371370926150
H	4.893656889261	-5.625664482305	-3.295821528095
H	5.271562862701	-5.912119547822	-1.578323781262
C	0.987710178011	-2.365197453538	-3.574355924506
H	1.658277240591	-2.031075328322	-4.377932825041
H	0.477268209712	-1.484677290188	-3.157224759866
H	0.207268737393	-2.990891111971	-4.037545230843
C	-0.885488843314	-5.374593583120	-1.876368175802
H	-0.296889325222	-5.273067171813	-2.800511331915

H	-1.742345360573	-6.034680800980	-2.085180035747
H	-0.250807492018	-5.861248795637	-1.120171542590
C	-2.531410248009	-3.933857641029	0.291250432491
H	-1.957193894126	-4.438040009484	1.083165920015
H	-3.421827500751	-4.544491008026	0.070436726775
H	-2.887706000717	-2.969180565552	0.682247577119
C	-2.542292331432	-2.857792689811	-2.578513562695
H	-3.062088552650	-1.959227222747	-2.201326987050
H	-3.323695103387	-3.554329516795	-2.926016463155
H	-1.932903314795	-2.578653511366	-3.450241666686
C	-1.476195147232	0.531536277224	2.946511263649
C	-1.112997459012	1.863456336573	3.230495974396
C	-0.098739258435	2.109165374730	4.160954394232
H	0.171523534416	3.146266389885	4.381570966802
C	0.579483628860	1.074539425401	4.809656291835
C	0.211705722543	-0.238108906571	4.502639958581
H	0.730762460443	-1.067919690749	4.990940858722
C	-0.801523918091	-0.529116034182	3.585581634502
C	-1.801798315350	3.001463593734	2.542138371010
H	-2.851497456175	3.097006231469	2.861532370007
H	-1.830131356025	2.851872964610	1.450819122556
H	-1.297570247905	3.954979498553	2.749096222995
C	1.655198804171	1.361836892811	5.816618969574
H	2.133364309798	2.333987662948	5.628545712873
H	2.434097319082	0.585192323561	5.805162362092
H	1.243823669985	1.392383477561	6.839452312643
C	-1.170042242388	-1.948045809748	3.284484640918
H	-0.489901682562	-2.651738233560	3.782764444908
H	-1.140046057596	-2.147620681178	2.201387080153
H	-2.198491616866	-2.175482659885	3.605016830276
C	-5.194284712565	1.431629825202	1.465938401111
H	-4.846342104832	2.466440632948	1.604563613088
H	-6.264576232949	1.388252475598	1.723426022038
H	-5.098945371354	1.159899917585	0.401353222731
C	-4.256816602372	0.668373986246	4.336127205636
H	-3.674106255873	-0.053283927787	4.929531801602
H	-5.297090994706	0.647746425705	4.698141889456
H	-3.847473265510	1.672049349004	4.524895604792
C	-4.943059754195	-1.491595856566	2.262115330493
H	-4.957394762005	-1.760023901972	1.194308349777
H	-5.988315528452	-1.508837459075	2.612038892692
H	-4.384329904985	-2.261830147663	2.814896633942
H	-3.288701507019	-0.205088291923	-0.543171504733
O	-4.723601432086	-0.335093842229	-1.675166164206
C	-5.862047026090	-0.872582147807	-1.426221212715
C	-7.073337562834	-0.110188340704	-1.377893397849
C	-6.017483806137	-2.271847103397	-1.164318756335
C	-8.302557431355	-0.689536618753	-1.085317592207
H	-7.001613952998	0.963508733486	-1.579839850539
C	-7.254441398831	-2.838135429333	-0.874014292272
H	-5.121350035957	-2.897495810225	-1.194511692897
C	-8.416832458980	-2.060863924616	-0.825325704690
H	-9.197146854680	-0.057707928625	-1.058007074276
H	-7.314326136374	-3.914208982068	-0.677127079081
H	-9.385591120952	-2.510611521905	-0.594935810064

153
PhOH_Int_3(THF), G=-4712.982197

Si	-1.918423796943	0.165386274233	0.191210664394
Si	0.038195109684	-0.778370495074	-0.266688299260
Si	0.840422064875	0.675781061314	1.365988717188
Si	-0.754922877143	2.179928919090	0.482467857698
Si	-4.907778754002	1.108104728092	0.309346010741

Si	-0.548845194123	-3.727365620096	-1.139493753551
Si	3.153274190163	2.365827380349	2.472850265839
Si	-1.109665048177	5.026495277822	-0.681868211570
N	-3.628239118947	-0.114373221175	-0.058174651190
N	0.560735276996	-2.363298609917	-0.780326566713
N	2.555879810540	0.975334704455	1.521774692024
N	-0.518771937661	3.347373536113	-0.826764805367
C	-4.146153827274	-1.429116024046	-0.314830197719
C	-4.318178446098	-2.325683367314	0.760202255194
C	-4.905903287393	-3.568300335960	0.521515355138
H	-5.032204925075	-4.258040771327	1.361594307107
C	-5.336632741215	-3.952521295032	-0.751720256092
C	-5.166144788495	-3.043614567262	-1.795707102772
H	-5.494036486265	-3.318136346050	-2.803116154231
C	-4.577547926059	-1.788782333745	-1.603931563849
C	-3.908309226966	-1.940524757458	2.147153760383
H	-4.000228822589	-2.789255509302	2.837039350184
H	-4.528433288157	-1.119997325037	2.541396565926
H	-2.868431008378	-1.579534777159	2.193791086751
C	-5.926813522010	-5.312332283275	-0.989015423253
H	-6.528058451790	-5.337183676676	-1.909042020429
H	-6.565001395245	-5.627261828202	-0.150024406055
H	-5.133812224775	-6.072050500341	-1.093604534680
C	-4.438037641914	-0.853978077677	-2.767134766748
H	-4.325218911437	-1.412832844925	-3.706755459977
H	-3.578134759616	-0.184024376040	-2.647029298010
H	-5.330386315385	-0.215564501119	-2.873796259049
C	-6.523458084790	0.191702435319	0.568729778456
H	-6.825197159723	-0.359262155576	-0.334648928949
H	-7.305791805536	0.934737246907	0.794359127373
H	-6.476669626076	-0.521617162202	1.404185292986
C	-5.141590564304	2.305592653191	-1.111599398590
H	-4.198129506152	2.786799781748	-1.393273818993
H	-5.847773038172	3.095552544867	-0.809340331745
H	-5.550153231263	1.800910350267	-1.998697370113
C	-4.459884055289	2.042023763136	1.870820398771
H	-4.381628435133	1.354724038355	2.725961243063
H	-5.246149968154	2.782578618681	2.089066516928
H	-3.503293278972	2.576135447857	1.781770540980
C	1.930303075207	-2.569890325351	-1.148045559956
C	2.401583008090	-2.113885755248	-2.395747579980
C	3.686273068215	-2.478128239113	-2.803197546466
H	4.043026098447	-2.136365278740	-3.780065222987
C	4.515394864215	-3.280046283385	-2.014283811136
C	4.040112318041	-3.678241394515	-0.764389229968
H	4.679777498194	-4.282133864693	-0.114432748134
C	2.768965965106	-3.322755916379	-0.307346763069
C	1.536939134189	-1.270224774392	-3.283240388173
H	2.037265764386	-1.064777513745	-4.239306037191
H	1.302292750542	-0.297789715401	-2.818785805405
H	0.570786342520	-1.752000741032	-3.493846622093
C	5.890689071122	-3.654347665979	-2.484567807772
H	6.272046569880	-4.540226452234	-1.956689296736
H	6.606606565956	-2.834441744373	-2.306492664621
H	5.903900338200	-3.864553155625	-3.564557255776
C	2.295834921962	-3.764951014856	1.043977244250
H	3.148353251376	-3.987618976211	1.699759678407
H	1.673190283508	-4.673015892823	0.984519318514
H	1.678799202170	-2.988504104356	1.517187328553
C	-1.712514227395	-3.193584269311	-2.508665561641
H	-2.072368605528	-2.167543269108	-2.350980892700
H	-2.594091672745	-3.848573093450	-2.571942647302
H	-1.182693377328	-3.226333394377	-3.473652042036

C	0.452193453013	-5.178988688101	-1.778718534737
H	1.028059156079	-4.895106568885	-2.672864990967
H	-0.248688671269	-5.980896793213	-2.063936712717
H	1.152012244377	-5.584402460527	-1.034520354719
C	-1.434298171201	-4.184565172128	0.445227927616
H	-0.731909818167	-4.668994567987	1.140694851395
H	-2.274680205593	-4.869463970508	0.256110361780
H	-1.838945900370	-3.289953799722	0.939064050106
C	3.573621980033	-0.006454528783	1.266656112702
C	4.275703304856	0.033455369274	0.041332347078
C	5.427868095985	-0.736977479232	-0.116886072483
H	5.971873613584	-0.673846652719	-1.063826160110
C	5.902529799847	-1.578199475664	0.890988561080
C	5.138455757638	-1.682512604976	2.051115532625
H	5.456305152315	-2.371637047495	2.840723797806
C	3.975540338955	-0.930997261108	2.256779000988
C	3.812240230464	0.904995895626	-1.082641202724
H	4.162995386082	0.510953389584	-2.046313727912
H	4.190549062660	1.933004345606	-0.980259881037
H	2.717060831106	0.972824208447	-1.115108322558
C	7.153466399943	-2.387948673309	0.713311316223
H	6.919436559276	-3.415905613124	0.390016182710
H	7.718756365334	-2.466800379320	1.653952383908
H	7.813491622673	-1.949902563775	-0.049264686122
C	3.198467846092	-1.173977345877	3.518778714103
H	2.778951629214	-2.193000431905	3.501183344367
H	2.366315424928	-0.474567545560	3.637254922758
H	3.845948302491	-1.113769487197	4.406128518279
C	4.658070312122	3.109854587626	1.627409370594
H	5.417209339747	2.333324694769	1.444333403944
H	5.104193230007	3.869747025634	2.288899161858
H	4.417327450195	3.589681299308	0.669375182212
C	1.767140892496	3.616670679413	2.645347825757
H	1.386919922830	3.975938049875	1.680780969343
H	2.150364754664	4.487593723182	3.201489636096
H	0.924941151820	3.195074328057	3.214949705710
C	3.706197938843	1.911905984579	4.211778074366
H	2.888172559728	1.482847655385	4.807720833928
H	4.045817683925	2.836019610314	4.708492874866
H	4.547893788942	1.204141902869	4.208040650115
C	0.140979855011	2.966948499924	-2.033720443060
C	-0.574536600606	2.365453678095	-3.086888426242
C	0.099441538413	2.001907693180	-4.257474654620
H	-0.467898751096	1.523682639085	-5.061831244532
C	1.462935899619	2.241250111406	-4.432180384715
C	2.146009222214	2.880479279207	-3.394512946791
H	3.212529144481	3.097904919867	-3.507750326938
C	1.512021497348	3.245411657695	-2.205374675208
C	-2.052054331705	2.167253636373	-2.982378033347
H	-2.577533901756	3.134604375928	-2.937478949090
H	-2.322725623151	1.623806118213	-2.065192441968
H	-2.443376219339	1.610267800370	-3.844181436996
C	2.180808588648	1.819261595319	-5.681153723093
H	1.477737565914	1.488427135594	-6.458542666119
H	2.869820080853	0.982873876258	-5.477196144381
H	2.788312662542	2.639343045321	-6.094073930139
C	2.277703523510	3.934471909989	-1.121783454872
H	3.325739710765	4.084600350645	-1.412845408543
H	2.255156961130	3.340570861811	-0.198053880855
H	1.851096522282	4.918926049928	-0.880767791955
C	-2.968760311939	5.099283591665	-0.417865998362
H	-3.520477328845	4.799468621612	-1.320293203632
H	-3.255072147670	6.136229806820	-0.177903621767

H	-3.298495637550	4.459844578185	0.414346009933
C	-0.701340445358	5.901181553758	-2.291593966511
H	0.381117092107	5.945205889192	-2.482305393004
H	-1.085402824260	6.933033092496	-2.248560878252
H	-1.173862051621	5.395277076995	-3.147637948416
C	-0.335259943400	5.948010442322	0.764239430133
H	-0.568587457271	5.462681347000	1.724014664276
H	-0.754591041021	6.967138056017	0.793386605444
H	0.757954649503	6.034798451336	0.682977077651
H	-1.369885763062	3.024645655046	1.556259870683
O	0.453428562175	0.427803051476	3.028058033171
C	-0.614618110232	-0.076420064054	3.666205616262
C	-0.633259935506	-1.430390607592	4.029852203335
C	-1.678424503677	0.751927660479	4.043630297726
C	-1.683196223153	-1.934093365495	4.793591504575
H	0.194947688096	-2.069981424600	3.718342742585
C	-2.725943177798	0.237168809267	4.806337547028
H	-1.659575855039	1.802697683921	3.747199849146
C	-2.731222843324	-1.102997103834	5.192658209587
H	-1.683353368576	-2.989711476493	5.077003782413
H	-3.545614377084	0.895386045546	5.105964463198
H	-3.553178957713	-1.500809242454	5.791785739208

166
PhOH_Int_4(THF), G=-5019.813080

Si	-1.631151892853	0.889241496152	-0.413324728132
Si	0.168489333265	-0.309259044798	-0.889508558678
Si	0.712576056034	0.200271617135	1.316001223579
Si	-0.219934231889	2.314506942682	0.769436956122
Si	-4.345516831219	2.497308093151	-0.472225328428
Si	-0.585271300995	-2.227330011205	-3.232302435217
Si	3.000065803417	0.738835478130	3.338291580912
Si	0.326197878561	5.338890737939	0.617927049329
N	-3.290575056261	1.102789157962	-0.919395187680
N	0.539904108411	-1.633636638261	-1.959145192022
N	2.344342113047	-0.076976541646	1.887369576753
N	0.592950476616	3.681280862729	0.002876945185
C	-3.982566452671	-0.031620371816	-1.465110443176
C	-4.399264371342	-1.065906111666	-0.599575036497
C	-5.104653377471	-2.148122321342	-1.127123005244
H	-5.413755890019	-2.950466798857	-0.450555496802
C	-5.410350972967	-2.237272265926	-2.488378371614
C	-5.016070155793	-1.185594610442	-3.316334362765
H	-5.263817709530	-1.221803066585	-4.381773513287
C	-4.319082741724	-0.073875146769	-2.829659244985
C	-4.118854652768	-0.990761922968	0.869785140111
H	-4.515168637147	-1.869837602560	1.392902838979
H	-4.563277238400	-0.089427793058	1.319856054396
H	-3.036286499975	-0.944287126096	1.070800008939
C	-6.106565193147	-3.445529914601	-3.042506365438
H	-6.624571078999	-3.219058024834	-3.985443439519
H	-6.841226001705	-3.850397914110	-2.330963383098
H	-5.381413223386	-4.250722146497	-3.251031766452
C	-4.016189479720	1.075973798323	-3.743694552882
H	-3.916699338181	0.741315896023	-4.785092798559
H	-3.099695815714	1.604154439214	-3.450898424677
H	-4.834483965370	1.815198330693	-3.717008773615
C	-6.121450787177	1.958764316332	-0.734814745202
H	-6.337072296535	1.722762257419	-1.786999254014
H	-6.783447557388	2.783923671816	-0.425783023190
H	-6.370646385402	1.073926678191	-0.130024429539
C	-3.970884951863	3.996653317452	-1.528241293512
H	-2.929530257706	4.317749219424	-1.403922170434

H	-4.620244282983	4.831509114494	-1.219335955724
H	-4.146019049981	3.798608030606	-2.595162983441
C	-4.119881822223	2.943091375207	1.337529727876
H	-4.475375651988	2.132784311177	1.991742269848
H	-4.713712233116	3.844041073037	1.561021638737
H	-3.073102629326	3.159304666216	1.594014839416
C	1.881082569478	-2.135011025925	-2.070428725761
C	2.821105391372	-1.426377607471	-2.846895278645
C	4.059950386643	-2.010799363315	-3.109591439813
H	4.778537024213	-1.462243150901	-3.726901611069
C	4.397389640713	-3.278449949702	-2.627111786302
C	3.465946325015	-3.938211922196	-1.826888893427
H	3.712729113854	-4.922363420857	-1.417640672476
C	2.215533491962	-3.387364569756	-1.527718098861
C	2.483635254759	-0.085891003864	-3.422684518798
H	3.352077553349	0.352745402654	-3.931236978976
H	2.155371294719	0.628512221363	-2.650552926575
H	1.660627940589	-0.150102745999	-4.151472665701
C	5.736584517826	-3.881028452121	-2.936667938054
H	5.753477331203	-4.960159158885	-2.727825696858
H	6.530850521164	-3.415706050837	-2.329630519705
H	6.010187490240	-3.730780689765	-3.992151091256
C	1.237312609130	-4.159592303313	-0.696201340265
H	1.765051924879	-4.822807864682	0.003769826057
H	0.593845883063	-4.795854379847	-1.328318577743
H	0.568490197062	-3.502656913931	-0.126438001411
C	-1.216315864966	-0.754091166497	-4.205244624895
H	-1.527192806564	0.059944180509	-3.535603385802
H	-2.086943951067	-1.035338695050	-4.817003104512
H	-0.433230202853	-0.362502128323	-4.872248347473
C	0.373481749427	-3.358534431291	-4.378975483168
H	1.227062980947	-2.839206045029	-4.840035613241
H	-0.301531497616	-3.695952832149	-5.182520165228
H	0.757227957626	-4.246907536332	-3.856030631491
C	-1.989712826341	-3.164759304693	-2.436134080367
H	-1.610229364953	-4.075505753899	-1.949876151163
H	-2.738419953749	-3.454134513859	-3.189608997907
H	-2.499980769118	-2.554250377421	-1.677816370670
C	3.173099193676	-1.165543501554	1.442824663564
C	4.186164221303	-0.920779594021	0.491239563234
C	5.150152777017	-1.901956356168	0.248032843893
H	5.942787598298	-1.685610634912	-0.473942134490
C	5.130064623713	-3.138534450266	0.892178333687
C	4.061643610581	-3.399218084651	1.749483915539
H	3.991783443365	-4.377769311317	2.235428263676
C	3.075066949431	-2.448828626038	2.027475778153
C	4.255666573108	0.375005948640	-0.254004063047
H	4.731199089129	0.227765764924	-1.232980732659
H	4.841102220162	1.128571913027	0.292667576954
H	3.257421014139	0.800859160887	-0.416059826780
C	6.183450668230	-4.176607203114	0.638672162768
H	5.827696957700	-4.937055789851	-0.075960966717
H	6.455223096394	-4.706666184341	1.563947619313
H	7.096025837345	-3.732472727695	0.216194814539
C	1.931282134241	-2.845422263766	2.910894487310
H	1.118968750701	-3.280277476810	2.306496716545
H	1.501762595599	-1.988939359214	3.434875727238
H	2.240694051711	-3.603377458393	3.644471829587
C	4.754061971323	1.343232450056	3.038517705966
H	5.395967983323	0.515490300821	2.699200024809
H	5.160233673474	1.714567224378	3.993211463694
H	4.814877663481	2.156320571167	2.304036364895
C	1.871027945912	2.168118279449	3.785422130762

H	1.807872652928	2.921362620501	2.988387457209
H	2.284779973679	2.662381822131	4.679653397851
H	0.850074127192	1.838199902056	4.032630010272
C	3.135065720421	-0.407198888889	4.824665405717
H	2.158432382278	-0.719768420414	5.217732473515
H	3.658627882681	0.140659445222	5.625376277353
H	3.725355062801	-1.305952278978	4.590851803801
C	1.436133461900	3.525855785374	-1.136956726894
C	0.899490018750	3.481105459027	-2.439107926635
C	1.764518105873	3.365901443524	-3.532311411063
H	1.334026857628	3.320668638916	-4.537396612262
C	3.150574725555	3.312665933732	-3.380861267648
C	3.663988577338	3.380408077669	-2.083575446680
H	4.747073889089	3.342326517752	-1.931496606254
C	2.834548300663	3.488639434157	-0.965869918621
C	-0.572342903870	3.616548985835	-2.666697482919
H	-0.922458990435	4.625784957168	-2.397060338652
H	-1.144533596568	2.910684637552	-2.046098731379
H	-0.833919121610	3.437798352650	-3.718655565583
C	4.063829480135	3.201293987349	-4.567342650342
H	4.873755558346	2.478947027930	-4.383179790980
H	4.542485284313	4.168257433719	-4.795362939616
H	3.518300982737	2.881986272141	-5.466658927748
C	3.420667288384	3.587626580954	0.406569689312
H	4.510487812639	3.458029887851	0.380574354159
H	2.991095347378	2.825036722928	1.070289207530
H	3.212163760465	4.564758989468	0.867832881724
C	-1.484287479899	5.835507351941	0.528915589523
H	-1.820996266597	5.945960886602	-0.512233729264
H	-1.614407734910	6.809207995925	1.028802171893
H	-2.144830255797	5.112252941311	1.028420149271
C	1.302314273695	6.510644971428	-0.475982361009
H	2.385469036150	6.323927119146	-0.439413201598
H	1.119967274834	7.545309244177	-0.143725225824
H	0.980692409252	6.426576680806	-1.525611045123
C	0.859648694547	5.523415459889	2.411909749302
H	0.278343759716	4.865732262094	3.075866835198
H	0.681233163293	6.563363937304	2.730843552196
H	1.926578617934	5.301665613055	2.560444655764
H	-0.892309804741	2.978023205058	1.927973720586
O	-0.277546081595	-0.622295553260	2.521168685057
C	-0.965653955764	-0.141456916704	3.585645713169
C	-0.680346871341	-0.635581938804	4.864251345680
C	-2.000694181002	0.785169387557	3.425581142609
C	-1.403552155693	-0.184272272215	5.966788594120
H	0.102966710511	-1.385667508848	4.981027032532
C	-2.714726015464	1.236843784666	4.532907556948
H	-2.249443968332	1.131280254522	2.423282921108
C	-2.418514985371	0.760087419097	5.810038342834
H	-1.168372348083	-0.577717395174	6.958768176416
H	-3.517089821989	1.965045863717	4.389784852974
H	-2.981065078355	1.114887641939	6.676215611924
O	-1.206524642170	-2.884692336496	1.343826066506
H	-0.906206009761	-2.117245571709	1.878955642659
C	-2.238772650383	-3.530845020976	1.912112096106
C	-2.905037581630	-4.500161297368	1.150830354519
C	-2.676949721797	-3.260268500026	3.215076460584
C	-4.004452193447	-5.169881580922	1.680184986324
H	-2.559386728905	-4.697302600401	0.134621169060
C	-3.780587595227	-3.935802099252	3.732901374621
H	-2.161770493361	-2.507025859945	3.815282793021
C	-4.455259346055	-4.891300816872	2.972546336471
H	-4.519591459105	-5.916633014575	1.070214604251

H	-4.115337938214	-3.706206836718	4.747909125107
H	-5.321182721082	-5.415421460312	3.382569063546

166

PhOH_TS_2(THF), G=-5019.785222

Si	-1.772458426256	0.717079973160	0.091366263478
Si	-0.332885462207	-0.965415649474	-0.265147492527
Si	0.947808928473	0.378140559137	1.138162243726
Si	-0.233073033704	2.327150896057	0.544634857037
Si	-4.483848215527	2.225979503324	0.434847167974
Si	-0.996426242764	-2.054151141266	-3.114840788440
Si	3.347454750612	1.246166381063	2.826866563083
Si	0.096664136902	5.379896769458	0.081883157055
N	-3.517995365824	0.864260668126	-0.205176049839
N	0.036597430616	-1.968352785974	-1.643129480170
N	2.670257776565	0.277459576140	1.476140849933
N	0.332991221918	3.692236914614	-0.443404850084
C	-4.266506351968	-0.270098989520	-0.639966866761
C	-4.513702949332	-1.329452768456	0.261474632610
C	-5.283686354884	-2.416145346559	-0.158154143438
H	-5.463267438720	-3.232813063714	0.546694625816
C	-5.834174352505	-2.484049402595	-1.440783132857
C	-5.612669938853	-1.407333382759	-2.300619260352
H	-6.047516711726	-1.427051570642	-3.304960031684
C	-4.844730449924	-0.299957259515	-1.925946054734
C	-4.032029290744	-1.252137335621	1.679768269040
H	-4.149298923404	-2.216456786380	2.191613496720
H	-4.600024743088	-0.492510964718	2.242773912407
H	-2.974103748188	-0.952085233711	1.744418616962
C	-6.600950252646	-3.693669557907	-1.889545597946
H	-7.343793378529	-3.440770072946	-2.660033777236
H	-7.122573111125	-4.176568817989	-1.050204681700
H	-5.921787210674	-4.445949984726	-2.326185869173
C	-4.714909767267	0.858490501386	-2.869736014126
H	-4.691195817775	0.518707590170	-3.914722057594
H	-3.814883446554	1.450335727314	-2.666066523168
H	-5.578834601004	1.537080096144	-2.769259964883
C	-6.267670599419	1.658098400081	0.576399820042
H	-6.685960723845	1.381234880289	-0.402924508298
H	-6.871595040665	2.484739225900	0.984595966118
H	-6.372731734293	0.792177637633	1.247092075688
C	-4.409929715053	3.709603585882	-0.711135196006
H	-3.372819128719	3.939021325591	-0.987228633627
H	-4.834154798842	4.597519132406	-0.216072857136
H	-4.976618075642	3.525870156226	-1.635349918664
C	-3.858441821005	2.718855899436	2.136709854004
H	-3.909917025745	1.868872297874	2.833981409669
H	-4.485110605700	3.532757311116	2.536196354535
H	-2.818216356140	3.075619396572	2.110388367850
C	1.374394599837	-2.465206405587	-1.825008939534
C	2.338378295284	-1.638280115566	-2.440022647476
C	3.597230804993	-2.163006710026	-2.734030301158
H	4.335267850377	-1.513253693268	-3.213693025652
C	3.937013974013	-3.485818099336	-2.445967306423
C	2.975792218118	-4.279114083260	-1.820010818901
H	3.216318082094	-5.318332469767	-1.575362640030
C	1.700678660133	-3.796440195696	-1.504092103946
C	2.010891707949	-0.236177109231	-2.850213556692
H	2.916751332739	0.318142716273	-3.119321854640
H	1.487483312338	0.330647127487	-2.065032644770
H	1.349338655590	-0.222672312542	-3.730176331587
C	5.279773883726	-4.034286319875	-2.828867915389
H	5.518866901068	-4.949218496270	-2.268773530497

H	6.077177779840	-3.299926317033	-2.644208260020
H	5.312439854065	-4.285626264249	-3.902352215700
C	0.704534045028	-4.711823558190	-0.861712967867
H	1.063217307004	-5.750104212762	-0.871214773616
H	-0.268176023341	-4.675228397532	-1.371909922832
H	0.515272710603	-4.431249985164	0.184644252527
C	-1.544257384912	-0.350736144952	-3.668602661930
H	-2.098458220530	0.151693187593	-2.864798415659
H	-2.214583559127	-0.442830782401	-4.538233943992
H	-0.698073070893	0.294595287990	-3.942393253636
C	0.007214055068	-2.894297522917	-4.459933093012
H	0.907083439231	-2.327984659141	-4.739349817487
H	-0.629982487179	-2.994692819953	-5.353640882616
H	0.323598293879	-3.901921237691	-4.149818779968
C	-2.538694616311	-3.089560797658	-2.844747983513
H	-2.300169109096	-4.129715213872	-2.574009439808
H	-3.099692428333	-3.111585707838	-3.793528408079
H	-3.212102131622	-2.664085624416	-2.087256110043
C	3.605752829452	-0.722119975683	1.027780258936
C	4.566573874692	-0.378471673939	0.053685739450
C	5.638050137687	-1.241801592610	-0.202303165934
H	6.380537446162	-0.941489118308	-0.948301877334
C	5.782960021264	-2.461020596399	0.452186908209
C	4.766663717053	-2.834427783274	1.333728018086
H	4.817288373596	-3.811306135679	1.824605204401
C	3.681081601318	-2.005586251732	1.624683302014
C	4.472740366487	0.892424654281	-0.728310580730
H	4.836267768120	0.736824060583	-1.753838721480
H	5.086673291851	1.689346455719	-0.285227684472
H	3.442400166967	1.258496058550	-0.776525079858
C	6.971409227386	-3.349161403153	0.226673648129
H	6.665459269711	-4.383502750413	0.007479645519
H	7.610707033380	-3.387341537750	1.124104390766
H	7.590752913845	-2.992324199804	-0.608111528279
C	2.613795530711	-2.535177613579	2.531069105890
H	1.726260982209	-2.823133208274	1.946074045487
H	2.281285168929	-1.789524643129	3.257923247626
H	2.963995381642	-3.428777661094	3.065424098402
C	4.992267421649	2.032731842797	2.379286066849
H	5.723468545683	1.285309629604	2.036809805123
H	5.395448843713	2.509589116213	3.287465486225
H	4.889705548046	2.808073320144	1.607284835517
C	2.140483262461	2.601920562139	3.290932625355
H	1.953044872226	3.294897231872	2.459733175066
H	2.598946951451	3.179427169435	4.110813491498
H	1.172182717945	2.226428264168	3.653500614769
C	3.656718930164	0.181975675442	4.345771990282
H	2.719974990435	-0.197096170826	4.779951191203
H	4.154599616260	0.802043740610	5.109042091287
H	4.314189706163	-0.671649698400	4.122185394338
C	0.959001219626	3.447677137689	-1.698123687954
C	0.187094414484	3.171119107200	-2.845980546794
C	0.834713748713	2.904436510169	-4.055373058715
H	0.226369530888	2.681353957034	-4.937591336790
C	2.226528088355	2.928849339281	-4.173972066381
C	2.969080226579	3.263846532691	-3.040073627158
H	4.060165082946	3.314943850801	-3.109117736714
C	2.361867596975	3.526575880269	-1.809020104390
C	-1.308176602917	3.230655830178	-2.788642498055
H	-1.648299370638	4.249270139840	-2.540426541391
H	-1.709180911687	2.565034966814	-2.009680170907
H	-1.755463717761	2.946406744388	-3.750655512312
C	2.900134616770	2.570449258315	-5.466296369418

H	3.877266525170	3.065199528314	-5.565887047113
H	2.283373863492	2.843711865333	-6.335082010719
H	3.077922541956	1.482573959307	-5.524296235580
C	3.189029182768	3.926519779686	-0.625490758535
H	4.259736181884	3.796570757410	-0.827330200617
H	2.921120734254	3.339593406918	0.264374150602
H	3.028749706381	4.984312765875	-0.366584241981
C	-1.711275210579	5.771856819420	0.414698323474
H	-2.301999990798	5.767913562338	-0.512497708689
H	-1.782159842000	6.779509763333	0.856137839273
H	-2.173214558275	5.063525713825	1.117770605438
C	0.663774333180	6.491476618122	-1.323644411144
H	1.733374330331	6.384470098611	-1.555216902546
H	0.475108304977	7.542122467956	-1.050011646046
H	0.097535353102	6.271997534636	-2.242325422123
C	1.026551848805	5.797512211258	1.664598325372
H	0.624559971715	5.231587273949	2.518736671815
H	0.892909139062	6.869952898844	1.882430467491
H	2.105136855152	5.595601696240	1.597833100733
H	-0.625913995901	3.099621899479	1.773539258558
O	0.328730274314	-0.570534190284	2.680260616706
C	-0.263719229239	-0.194254178339	3.858291836889
C	0.019663957123	-0.938903502324	5.006045773565
C	-1.135873289368	0.893009032460	3.921417208786
C	-0.571782580200	-0.590020514248	6.219094405822
H	0.707697117713	-1.784319255318	4.942539025454
C	-1.715708449652	1.237012515023	5.141513180656
H	-1.359542362722	1.446883584262	3.010442435839
C	-1.439149457408	0.500112507009	6.293068199577
H	-0.345478053070	-1.174383293834	7.114049270020
H	-2.397010469108	2.090337227221	5.184926675432
H	-1.898183685014	0.773810167160	7.245505409123
O	-0.536173169907	-2.183694023752	1.170751419241
H	-0.161954918050	-1.473546655960	2.083713398964
C	-1.372241823894	-3.213530744555	1.496385667367
C	-2.195811001259	-3.795540194388	0.538937400014
C	-1.382274313499	-3.678906559255	2.813875440039
C	-3.030042188739	-4.850860255672	0.897481329254
H	-2.184182021884	-3.414378601148	-0.477968111698
C	-2.228103482577	-4.728743835341	3.164090244339
H	-0.739097939103	-3.214191343538	3.563276408320
C	-3.054319960656	-5.323523239861	2.209147146333
H	-3.674473976648	-5.299447996921	0.137601876428
H	-2.237031426441	-5.084491508867	4.197106918122
H	-3.712969610403	-6.148897567678	2.487258608773

153
6_syn(THF), G=-4712.995998

Si	-0.164640793721	1.489243690904	-0.517130242708
Si	0.781615838855	-0.342021561949	0.514539667688
Si	-0.436524144214	-0.805493791772	-1.365539769960
Si	-2.215082899499	0.624416520788	-1.093538006125
Si	-0.386545509866	4.183930256244	-1.966359187645
Si	0.377048405132	-0.222323059678	3.650795950581
Si	-1.212009410899	-2.541674408198	-3.809022867328
Si	-5.243961059134	0.948040062601	-1.358021696677
N	0.393232043612	3.123772953584	-0.751337829105
N	0.693760926527	-1.052760584640	2.107789342995
N	-0.045229868815	-1.982107632766	-2.589000159595
N	-3.808717098011	0.599979276721	-0.348547170905
C	1.618166779495	3.569399456802	-0.165640130044
C	2.849748512536	3.259694359642	-0.780356851246
C	4.024468077874	3.780609379872	-0.240752026297

H	4.976308491545	3.533364201774	-0.719795855418
C	4.022705970144	4.579401994817	0.906536878545
C	2.795029170462	4.852401087090	1.510045998946
H	2.767807574621	5.472828272443	2.411326841290
C	1.587351251172	4.371904607813	0.988602146480
C	2.892835085046	2.392104893869	-2.001133653541
H	3.929408981574	2.197676463963	-2.307280302581
H	2.365222976412	2.856254935085	-2.848903381882
H	2.407491126904	1.420745737381	-1.820211111813
C	5.309701370287	5.103103452532	1.473443703781
H	5.855681678980	5.710624782926	0.734260230758
H	5.976379254297	4.274311791528	1.761283033424
H	5.136850473431	5.725348540679	2.362819697732
C	0.284345594160	4.762872370965	1.620595488503
H	0.431213491328	5.092649704455	2.658462415751
H	-0.439313265536	3.937623703520	1.611207548596
H	-0.183844168477	5.598358175492	1.073013628711
C	0.689171468655	5.707100653700	-2.149838940860
H	0.779619687541	6.256825784086	-1.200861284165
H	0.239880627783	6.380774296253	-2.897002255688
H	1.703240655598	5.444429399656	-2.487223565573
C	-2.103054684520	4.662422394547	-1.386619882659
H	-2.729013292362	3.773048988110	-1.231417075785
H	-2.598594320861	5.292045987268	-2.142883432653
H	-2.063586121294	5.221505265698	-0.439980569028
C	-0.517290079952	3.291599012762	-3.615623605331
H	0.476714818214	3.088729437054	-4.042163000462
H	-1.077582841420	3.913337080284	-4.332663523033
H	-1.046336112828	2.331602635872	-3.517040189680
C	0.777881125186	-2.488114997033	2.164242986367
C	-0.395691431239	-3.265172402470	2.042115952025
C	-0.321497633353	-4.654042921431	2.180531100970
H	-1.244040667238	-5.236396027933	2.090008379699
C	0.884417101068	-5.315116347852	2.409145767303
C	2.037894049173	-4.534933830491	2.495272895212
H	3.002107785239	-5.024195303087	2.666076581862
C	2.013691436248	-3.141565748297	2.374669328852
C	-1.725192743815	-2.659290546093	1.718451359940
H	-2.053309097105	-2.984787687914	0.718831997022
H	-1.700812803167	-1.565355475706	1.715233693081
H	-2.497215418270	-2.983428832229	2.431292949051
C	0.946961813439	-6.811646530585	2.507328398444
H	1.097186500863	-7.263703338103	1.512094339670
H	0.015179106682	-7.229328828771	2.915621198131
H	1.782114338187	-7.140919528126	3.142492755996
C	3.315607139412	-2.402560404246	2.436024617973
H	4.002135923439	-2.874979186548	3.153392806068
H	3.184648150281	-1.351831551633	2.708093657280
H	3.808684557777	-2.414755544875	1.450924964457
C	-0.114970270074	1.549506636962	3.291072971611
H	0.669665621571	2.128496331803	2.782197930047
H	-0.336062091849	2.055007160455	4.245162387088
H	-1.023552385041	1.580336353884	2.672128617526
C	-1.014375029250	-1.083895472775	4.567330504387
H	-1.970208407451	-0.989972233430	4.033192440768
H	-1.127621179983	-0.621030461517	5.561100612554
H	-0.797920164096	-2.153170459033	4.714189198886
C	1.885012257531	-0.256345071404	4.773932880507
H	2.184086308797	-1.291101313036	5.002334483596
H	1.633226796792	0.238700758833	5.726218024551
H	2.749291948877	0.263739337930	4.334972215481
C	1.330805938065	-2.370547292487	-2.607654011447
C	1.745919873741	-3.505468853268	-1.884356114818

C	3.103518862444	-3.833170940481	-1.859236532585
H	3.421140859876	-4.710817505596	-1.287565901731
C	4.061197113612	-3.071499512814	-2.536230042397
C	3.620264817719	-1.964315239505	-3.265226418947
H	4.349537710172	-1.356929917635	-3.810273545543
C	2.271670960854	-1.596957204987	-3.312487597006
C	0.742004893521	-4.342810551615	-1.155774622197
H	1.230933816050	-5.142400397192	-0.583988892193
H	0.021484828415	-4.804319558267	-1.849587364420
H	0.157876334274	-3.737194033794	-0.448190654191
C	5.520939933865	-3.413202196428	-2.447886391954
H	6.088423376200	-2.983364632726	-3.285698667965
H	5.682217378202	-4.501410296590	-2.443156881344
H	5.961772382514	-3.019095219544	-1.516109484052
C	1.835035474818	-0.406710460838	-4.113498027802
H	2.696578736831	0.203488956191	-4.416840322253
H	1.139680869705	0.229527018737	-3.544349711826
H	1.304188361600	-0.716145510736	-5.028652868463
C	-0.332295103356	-3.810651004566	-4.868953436692
H	0.538077941844	-3.363766737350	-5.374008093381
H	-1.017793302765	-4.193065101455	-5.641684690946
H	0.023231654494	-4.663643367172	-4.271428959680
C	-2.686569447784	-3.278742636931	-2.912489365009
H	-2.383356405179	-4.117107612431	-2.266897682690
H	-3.443273965808	-3.641494825915	-3.626185203830
H	-3.165179812984	-2.516295870011	-2.278181183191
C	-1.807135587745	-1.106273402965	-4.865078057682
H	-2.208261808127	-0.297822159199	-4.234512607456
H	-2.610119393945	-1.438234901352	-5.542890016643
H	-0.993056802863	-0.687143532083	-5.475164066667
C	-3.999035482110	0.350737478554	1.040445062045
C	-3.780749865307	1.381919694314	1.978810362307
C	-4.021163847423	1.135896833855	3.333065604073
H	-3.849692127198	1.943491855677	4.051393127687
C	-4.493282150167	-0.097477918187	3.790590422443
C	-4.712945480513	-1.099612490084	2.843613484126
H	-5.082659106837	-2.075367568854	3.173978502292
C	-4.470933346003	-0.901499277412	1.481004908741
C	-3.358218796079	2.746246306128	1.525171566249
H	-4.127366731595	3.206199446881	0.884258644913
H	-2.438801023907	2.711043670914	0.921330570836
H	-3.182450575946	3.412653358366	2.380584038543
C	-4.726338575426	-0.345008435255	5.252759567692
H	-3.810948946795	-0.724964952449	5.736876747263
H	-5.515414211111	-1.093601840705	5.414630894984
H	-5.012407195261	0.578176126280	5.777497291715
C	-4.738203992194	-2.006098871028	0.503941863288
H	-4.757407783832	-2.982969189942	1.005417806970
H	-3.977463738023	-2.028181838788	-0.287591773057
H	-5.711839604275	-1.875132052885	0.004265786157
C	-5.133883166753	2.635389065669	-2.180991078834
H	-5.071674812357	3.444056081939	-1.437138296374
H	-6.045691027699	2.796277509296	-2.779518895774
H	-4.269825993201	2.720872062971	-2.856124966560
C	-6.740475286628	0.969103611398	-0.226180603618
H	-6.923898446124	0.003709986034	0.266329731725
H	-7.633156524559	1.229333833456	-0.817306054910
H	-6.617630948711	1.731299426594	0.559218988536
C	-5.426722789384	-0.338935430110	-2.716333847837
H	-4.540339930869	-0.350540609049	-3.369672754333
H	-6.301180675680	-0.101061426984	-3.343390215661
H	-5.561140898604	-1.351997333068	-2.310083949817
O	2.410400367910	-0.403197135276	-0.005195549352

C	3.498990071754	0.174081588754	0.532377454532
C	3.436892308944	1.049426851454	1.621312086453
C	4.744632904436	-0.141445585409	-0.025275631291
C	4.606980121729	1.565655011866	2.171235375538
H	2.467724355417	1.341275882338	2.027464947846
C	5.908556949337	0.390777432760	0.522411848967
H	4.768148123209	-0.816632350050	-0.883162954012
C	5.850280599847	1.237041763685	1.632627886938
H	4.538785877508	2.245654162482	3.023700427801
H	6.874932399275	0.132777676063	0.081070596055
H	6.766369980610	1.643289015368	2.067323715914
H	-2.533264096318	1.128199807910	-2.463952618899

153
6_anti(THF), G=-4712.990853

Si	0.650682871142	1.711736466627	0.028647622527
Si	-0.656861758828	-0.159075090365	-0.341780954150
Si	0.572314022534	-0.157135602280	1.648630501108
Si	2.513896981615	0.483931912952	0.594009116749
Si	1.949841033064	4.501122370793	-0.168222719428
Si	-3.604460060444	0.819906437667	0.407026568673
Si	1.735762853156	-1.048432086808	4.382192857141
Si	5.436805819179	-0.280506066550	0.036992109761
N	0.569378082842	3.392669661891	-0.445118437611
N	-2.408995885328	-0.214256944836	-0.415088622104
N	0.364268654893	-0.800743719871	3.260451443360
N	3.700654261792	-0.387357563616	-0.364756831458
C	-0.617523573997	3.890365653633	-1.060721057826
C	-1.531171101262	4.680243405367	-0.333268092141
C	-2.675941123129	5.155959326894	-0.980729351519
H	-3.387465903104	5.759887762342	-0.409176918184
C	-2.943269323977	4.876280513687	-2.322100241309
C	-2.014298928840	4.103968423661	-3.025369112312
H	-2.194971624885	3.877318555842	-4.080436690737
C	-0.856774905066	3.608397954036	-2.421949013448
C	-1.291066216571	5.036830510777	1.105255940059
H	-2.241073813474	5.202313925911	1.631620512617
H	-0.703571273620	5.965104559646	1.195244129822
H	-0.731091722592	4.252586075731	1.631234959187
C	-4.210027251077	5.347151609916	-2.975042576496
H	-4.072099390789	5.512781128864	-4.053413351863
H	-4.575065136886	6.281401637729	-2.524436917869
H	-5.009371927797	4.595195053670	-2.860295951479
C	0.134210860061	2.808870296312	-3.212901024479
H	-0.194395794435	2.691703617792	-4.254253823446
H	0.277437053586	1.801078260396	-2.789617374650
H	1.124641888877	3.289654225562	-3.212022767754
C	1.487480089623	6.182385229342	-0.857414193177
H	1.236070439257	6.098890626723	-1.926351459407
H	2.357230540611	6.852603886835	-0.764971287160
H	0.637897649451	6.652172193304	-0.343423436588
C	3.469081383866	3.909946338197	-1.092345099727
H	3.761251710665	2.893450365188	-0.798316904747
H	4.315498844830	4.579433555158	-0.868625539584
H	3.303956528159	3.918743789908	-2.180337672870
C	2.341184791644	4.560893655693	1.666683091767
H	1.515952712836	4.999711253598	2.246559636199
H	3.247278580541	5.161757535635	1.845106881095
H	2.528776019597	3.546104832639	2.052917213328
C	-3.000563773782	-1.120636752405	-1.358580441653
C	-3.145960872341	-0.745385858382	-2.711747020244
C	-3.774123933193	-1.621969044199	-3.599254257823
H	-3.880977591782	-1.319014345123	-4.645866606553

C	-4.248552582361	-2.872499108636	-3.199333318734
C	-4.071984102596	-3.234232932497	-1.864444314873
H	-4.411767152789	-4.218757391452	-1.526965440887
C	-3.447153041962	-2.392071027833	-0.939584697659
C	-2.567738971526	0.531079606097	-3.241685238549
H	-3.220405582361	0.983993971550	-4.002319047984
H	-1.594355411169	0.330287154920	-3.717512359687
H	-2.392098944107	1.268744245177	-2.452479703276
C	-4.867228066362	-3.821034135726	-4.184796945934
H	-5.637314787530	-4.449884325188	-3.714267709671
H	-4.104369403181	-4.500397759914	-4.602164059202
H	-5.325423760680	-3.286600630308	-5.029641512338
C	-3.231424991017	-2.892551026088	0.457352399445
H	-2.711778864850	-3.861727825057	0.432658367133
H	-4.185151092916	-3.044617959572	0.985656362950
H	-2.631327462361	-2.195046092413	1.051360641567
C	-4.545652614516	1.850440044367	-0.850099251070
H	-3.890934203698	2.583549803887	-1.343396980901
H	-5.346419328221	2.403671173448	-0.332517031461
H	-5.012888308296	1.219329300653	-1.621580382243
C	-4.856436822191	-0.207773251690	1.355570195661
H	-5.408130442698	-0.896096968331	0.697880841003
H	-5.586769933810	0.475382897751	1.819851810037
H	-4.375471642455	-0.789314889312	2.155140122428
C	-2.702157460761	1.931989163384	1.597723238079
H	-2.220568963246	1.343998710307	2.388150612988
H	-3.416390857802	2.624471906510	2.070886597348
H	-1.939413917913	2.531207575693	1.085951030982
C	-0.962924199467	-1.007979558791	3.745748242863
C	-1.571738363459	-2.270119521170	3.605880272006
C	-2.874650958037	-2.450801589811	4.077362490188
H	-3.346005423537	-3.430742749247	3.955608589769
C	-3.587871962136	-1.420440105931	4.695667533848
C	-2.948655386128	-0.188521262718	4.856134057959
H	-3.479985145355	0.629458208744	5.352216184904
C	-1.647227087656	0.037005853264	4.398730761078
C	-0.813427741064	-3.415280271540	3.007323141693
H	-1.481071195135	-4.250929768700	2.759269400894
H	-0.050000270232	-3.789977458329	3.708891575924
H	-0.274702175099	-3.112772371019	2.099664288032
C	-5.007428648879	-1.619923706160	5.140732960137
H	-5.201223607294	-2.666198938095	5.418123734841
H	-5.709448635868	-1.361860434528	4.329566445920
H	-5.255352528180	-0.981203851990	6.000813215389
C	-0.979117381796	1.359047618273	4.641599139545
H	-0.273509162178	1.292301999187	5.486121683945
H	-1.716879867719	2.134489478115	4.888607603196
H	-0.396648706211	1.692746692475	3.769719485783
C	2.893513464720	-2.341580720035	3.677377229919
H	2.375653074283	-3.300360846539	3.524167073121
H	3.742472567401	-2.508617912704	4.359478347086
H	3.300991576283	-2.015204634839	2.710649847186
C	2.684740737248	0.550643473011	4.642596160361
H	3.061258447309	0.949779332497	3.688932660135
H	3.551131135371	0.365643086347	5.298191168212
H	2.057173508158	1.322770826817	5.111717804720
C	1.015215015947	-1.646526362990	6.006904296293
H	0.333058125609	-0.906326247893	6.451255153559
H	1.835187845409	-1.828442987687	6.720006456580
H	0.458006217435	-2.587146168545	5.879638451197
C	3.323408670921	-1.195769286133	-1.480397710582
C	3.201131698924	-0.619961299206	-2.761325925191
C	2.930764756388	-1.441420789159	-3.856643564790

H	2.840194934720	-0.982936913315	-4.846704862205
C	2.782571663624	-2.825568430738	-3.726190172603
C	2.878706692742	-3.370167890572	-2.446925287662
H	2.725592618353	-4.444253952976	-2.310128341239
C	3.132138858285	-2.579901026240	-1.321592122286
C	3.378178757487	0.854936408214	-2.945663876962
H	4.404256240680	1.174922754329	-2.705284193909
H	2.713581527498	1.418661204632	-2.275480606739
H	3.160270901396	1.155866527682	-3.979721058458
C	2.520784103540	-3.685953352439	-4.927733885021
H	3.383405783533	-3.687573113561	-5.614377915582
H	1.653775863886	-3.318722324249	-5.498463978582
H	2.314292405623	-4.725573974888	-4.638736578689
C	3.203653416335	-3.204138261544	0.037629598733
H	2.577096966741	-2.652995536870	0.753866034033
H	4.228352595382	-3.192807672803	0.442972659518
H	2.861074960302	-4.247342625633	0.010353892556
C	6.080088069375	1.483205511766	-0.079795067803
H	5.966559469879	1.897706870747	-1.092573558750
H	7.153312739873	1.489455408747	0.172185500933
H	5.567370268109	2.154992768975	0.625144638913
C	6.363040755769	-1.349600772693	-1.194244309216
H	6.049693846967	-2.402869617363	-1.139881122195
H	7.443217304566	-1.297707748364	-0.983588814625
H	6.195262584326	-1.001113177444	-2.224956062466
C	5.772801258318	-0.870963465502	1.790755300341
H	5.191769314879	-0.297266642529	2.529402116440
H	6.841001560418	-0.732855013253	2.024788659594
H	5.531267783359	-1.936153240304	1.920742085456
H	3.347056661552	1.295137864996	1.532531284666
C	-0.134534831927	-3.352606336779	-0.877914770484
C	-0.212883438014	-4.716370099495	-1.146155667521
C	-0.457049332195	-5.167241141173	-2.443436287562
C	-0.619361892344	-4.232975439645	-3.469272789987
C	-0.537172313158	-2.869360521693	-3.208241083570
C	-0.296423888426	-2.420979017280	-1.906057003948
H	0.067409724110	-2.994279040772	0.132033006105
H	-0.079233549555	-5.431080113699	-0.329658562354
H	-0.521459369282	-6.237012856479	-2.654871380323
H	-0.811859353805	-4.571650583842	-4.490642643466
H	-0.658498763198	-2.131993980834	-4.003001731924
O	-0.198468346320	-1.092614014773	-1.685273547345

6. References

[28] Bruker SMART APEX (2012-10.6) and SAINT (Version 8.27a). Bruker AXS Inc., Madison, Wisconsin, USA.

[29] R. H. Blessing, An Empirical Correction for Absorption Anisotropy, *Acta Cryst.* **1995** *A51*, 33–38. https://doi.org/10.1107/S0108767394005726

[30] L. Krause, R. Herbst-Irmer, G. M. Sheldrick, D. Stalke, Comparison of silver and molybdenum microfocus X-ray sources for single-crystal structure determination. *J. Appl. Cryst.* **2015**, *48*, 3-10. https://doi.org/10.1107/S1600576714022985

[31] G. M. Sheldrick, SADABS, Program for Empirical Absorption Correction of Area Detector Data. University of Gottingen, Gottingen, Germany, 1996.

[32] G. M. Sheldrick, *SHELXTL Version 6.1*. Bruker AXS Inc., Madison, Wisconsin, USA, 2001.

[33] G. M. Sheldrick, SHELXT – Integrated space-group and crystal-structure determination, *Acta Cryst.* **2015**, *A71*, 3–8. https://doi.org/10.1107/S2053273314026370

[34] G. M. Sheldrick, Crystal structure refinement with SHELXL, *Acta Cryst.* **2015**, C*71*, 3–8. https://doi.org/10.1107/S2053229614024218

[35] H. Putz, K. Brandenburg, Diamond - Crystal and Molecular Structure Visualization, Crystal Impact GbR, Kreuzherrenstr. 102, 53227 Bonn, Germany. https://www.crystalimpact.de/diamond

[36] P. v. d. Sluis, A. L. Spek, BYPASS: an Effective Method for the Refinement of Crystal Structures Containing Disordered Solvent Regions, *Acta Cryst.* **1990**, *A46*, 194–201. https://doi.org/10.1107/S0108767389011189

[37] A. L. Spek, PLATON SQUEEZE: a tool for the calculation of the disordered solvent contribution to the calculated structure factors, *Acta Cryst.* **2015**, *C71*, 9–18. https://doi.org/10.1107/S2053229614024929

[38] Gaussian 16, Revision C.01, M. J. Frisch, G. W. Trucks, H. B. Schlegel, G. E. Scuseria, M. A. Robb, J. R. Cheeseman, G. Scalmani, V. Barone, G. A. Petersson, H. Nakatsuji, X. Li, M. Caricato, A. V. Marenich, J. Bloino, B. G. Janesko, R. Gomperts, B. Mennucci, H. P. Hratchian, J. V. Ortiz, A. F. Izmaylov, J. L. Sonnenberg, D. Williams-Young, F. Ding, F. Lipparini, F. Egidi, J. Goings, B. Peng, A. Petrone, T. Henderson, D. Ranasinghe, V. G. Zakrzewski, J. Gao, N. Rega, G. Zheng, W. Liang, M. Hada, M. Ehara, K. Toyota, R. Fukuda, J. Hasegawa, M. Ishida, T. Nakajima, Y. Honda, O. Kitao, H. Nakai, T. Vreven, K. Throssell, J. A. Montgomery, Jr., J. E. Peralta, F. Ogliaro, M. J. Bearpark, J. J. Heyd, E. N. Brothers, K. N. Kudin, V. N. Staroverov, T. A. Keith, R. Kobayashi, J. Normand, K. Raghavachari, A. P. Rendell, J. C. Burant, S. S. Iyengar, J. Tomasi, M. Cossi, J. M. Millam, M. Klene, C. Adamo, R. Cammi, J. W. Ochterski, R. L. Martin, K. Morokuma, O. Farkas, J. B. Foresman, and D. J. Fox, Gaussian, Inc., Wallingford CT, 2016.

[39] V. Barone, M. Cossi, Quantum Calculation of Molecular Energies and Energy Gradients in Solution by a Conductor Solvent Model, *J. Phys. Chem. A* **1998**, *102*, 1995–2001. https://doi.org/10.1021/jp9716997

[40] M. Cossi, N. Rega, G. Scalmani, V. Barone, Energies, structures, and electronic properties of molecules in solution with the C-PCM solvation model, *J. Comp. Chem.* **2003**, *24*, 669–681. https://doi.org/10.1002/jcc.10189
